# Dupilumab in patients with prurigo nodularis: two randomized, double-blind, placebo-controlled phase 3 trials

**DOI:** 10.1038/s41591-023-02320-9

**Published:** 2023-05-04

**Authors:** Gil Yosipovitch, Nicholas Mollanazar, Sonja Ständer, Shawn G. Kwatra, Brian S. Kim, Elizabeth Laws, Leda P. Mannent, Nikhil Amin, Bolanle Akinlade, Heribert W. Staudinger, Naimish Patel, George D. Yancopoulos, David M. Weinreich, Sheldon Wang, Genming Shi, Ashish Bansal, John T. O’Malley

**Affiliations:** 1 University of Miami, Miami, FL USA; 2 Perelman School of Medicine, University of Pennsylvania, Philadelphia, PA USA; 3 University Hospital Münster, Mϋnster, Germany; 4 Johns Hopkins University School of Medicine, Baltimore, MD USA; 5 Icahn School of Medicine at Mount Sinai, New York, NY USA; 6 Sanofi, Bridgewater, NJ USA; 7 Sanofi, Chilly-Mazarin, France; 8 Regeneron Pharmaceuticals Inc., Tarrytown, NY USA; 9 Sanofi, Cambridge, MA USA

**Keywords:** Inflammatory diseases, Drug development, Antibody therapy

## Abstract

Prurigo nodularis (PN) is a chronic inflammatory skin disease with intensely pruritic nodules. The LIBERTY-PN PRIME and PRIME2 phase 3 trials enrolled adults with PN with ≥20 nodules and severe itch uncontrolled with topical therapies. Dupilumab, a fully human monoclonal antibody, blocks the shared receptor component for interleukin (IL)-4 and IL-13. Patients were randomized 1:1 to 300 mg dupilumab or placebo subcutaneously every 2 weeks for 24 weeks. The primary endpoint was pruritus improvement, measured by proportion of patients with a ≥4-point reduction in Worst Itch Numeric Rating Scale (WI-NRS) from baseline at week 24 (PRIME) or week 12 (PRIME2). Key secondary endpoints included nodule number reduction to ≤5 at week 24. PRIME and PRIME2 enrolled 151 and 160 patients, respectively. Both trials met all the pre-specified primary and key secondary endpoints. A ≥4-point WI-NRS reduction at week 24 in the dupilumab and placebo arms was achieved by 60.0% and 18.4% of patients, respectively, in PRIME (95% confidence interval (CI), 27.8–57.7 for the difference, *P* < 0.001) and at week 12 by 37.2% and 22.0% of patients, respectively, in PRIME2 (95% CI, 2.3–31.2; *P* = 0.022). Dupilumab demonstrated clinically meaningful and statistically significant improvements in itch and skin lesions versus placebo in PN. Safety was consistent with the known dupilumab safety profile.

ClinicalTrials.gov identifiers: NCT04183335 and NCT04202679.

## Abstract (web summary)

In the LIBERTY-PN PRIME and PRIME2 phase 3 trials, dupilumab, a monoclonal antibody that blocks IL-4 and IL-13 signaling, significantly reduced itch and skin lesions in patients with prurigo nodularis, a chronic inflammatory skin disease.

## Main

Prurigo nodularis (PN), a chronic inflammatory skin condition characterized by intensely pruritic papulonodular lesions, substantially impacts quality of life (QoL)^1,2^. Variable prevalence was reported in several countries^3–6^; in the United States, it affects annually an estimated 18–72 adults per 100,000 population^7,8^.

PN is driven by an itch–scratch cycle with an intensity and frequency of chronic pruritus among the highest reported in dermatologic and other pruritic diseases^9–11^, and often is accompanied by skin pain, stinging and burning. The high symptom burden in PN causes sleep impairment and affects mental and emotional health^2,12^. The disease burden is frequently compounded by associated comorbidities, including infections, malignancies and renal, hepatic and neuropsychiatric disorders^13–15^; 18.7–46.3% of adult patients have a history of atopy or current atopic comorbidity, such as atopic dermatitis (AD)^7,8,11,13,16,17^.

PN represents a substantial therapeutic challenge, and inadequate disease control is common in this population^2,18–23^. Although topical treatments, UV light therapy, immunosuppressive agents and systemic neuromodulators are frequently prescribed, these therapies are limited by insufficient evidence for efficacy and/or associated side effects^18,23^. Recently, dupilumab was approved as the first systemic therapy indicated in PN^24,25^.

Case reports have shown successful treatment with dupilumab in PN^26–28^. Dupilumab, a fully human VelocImmune-derived monoclonal antibody^29,30^, blocks the shared receptor component (IL-4Rα) for interleukin (IL)−4 and IL-13, thus inhibiting signaling of these central drivers of type 2 inflammation.

**Extended Data Fig. 1:**
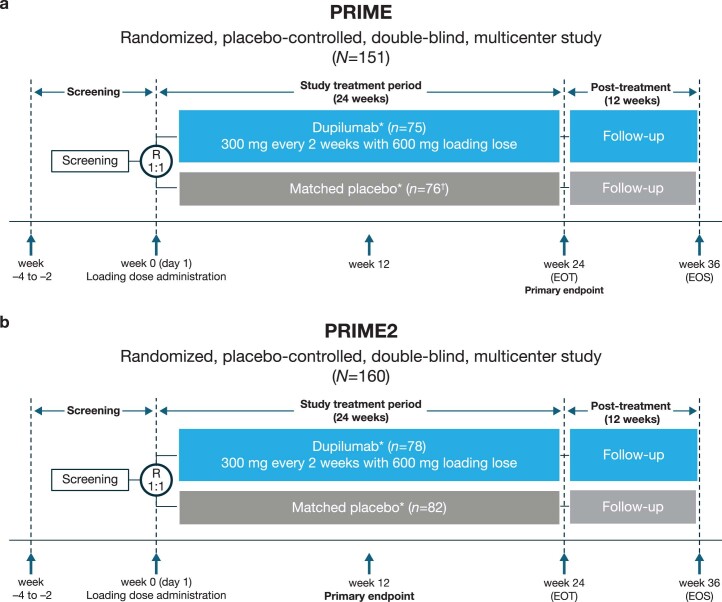
Overview of the trials design. **a**, Trial design of LIBERTY-PN PRIME and **b**, trial design of LIBERTY-PN PRIME2. ^*^Low-to-medium potency TCS/TCI as background therapy permitted (dose maintained from screening to EOT). †One patient in the placebo group was randomized but not exposed to study intervention due to patient’s decision (fear of being exposed to COVID-19). EOS, end of study; EOT, end of treatment; R, randomization; TCI, topical calcineurin inhibitors; TCS, topical corticosteroids.

In two parallel phase 3 trials of similar design, LIBERTY-PN PRIME and PRIME2, we assessed the efficacy and safety of dupilumab in adults with PN that was inadequately controlled with topical prescription therapies (Extended Data Fig. 1). Patients were randomized 1:1 to receive subcutaneous dupilumab 300 mg or matching placebo every 2 weeks for 24 weeks. Patients on a stable regimen of low-to-moderate potency topical corticosteroids (TCSs) and topical calcineurin inhibitors (TCIs) before screening were allowed to continue their use throughout the trial. The primary endpoint was the proportion of patients with a ≥4-point reduction in Worst Itch Numeric Rating Scale (WI-NRS) score (range 0 (‘no itch’) to 10 (‘worst imaginable itch’)) at week 24 (PRIME) or week 12 (PRIME2). WI-NRS is validated in PN, with research to date supporting a four-point reduction as clinically meaningful^31–33^. Key secondary endpoints in both trials included proportion of patients with reduction in skin lesion number to an Investigator Global Assessment for PN-Stage (IGA PN-S) score of 0 or 1 at week 24. IGA PN-S is also validated in PN, with scores ranging from 0 to 4 (0, ‘clear’ (no nodules); 1, ‘almost clear’ (≤5 nodules); 2, ‘mild’ (6–19 nodules); 3, ‘moderate’ (20–99 nodules); 4, ‘severe’ (≥100 nodules))^34^. Other pre-specified secondary and tertiary endpoints included assessment of QoL, skin pain, sleep and mental health.

## Results

### Patients

**Fig. 1:**
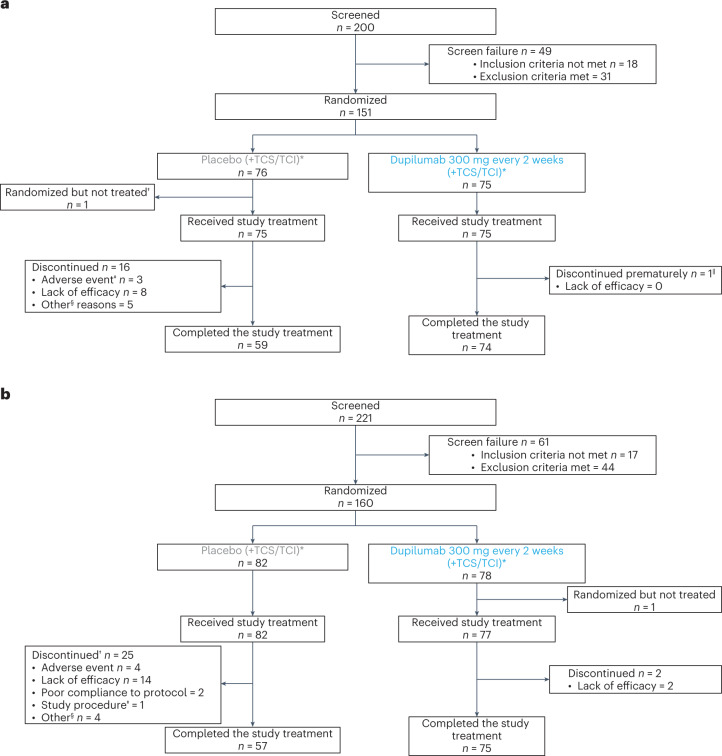
CONSORT diagrams of patient disposition. **a**, PRIME. No patients were lost to follow-up at the time of the cutoff date. ^*^Low-to-medium potency TCS/TCI as background therapy permitted (maintain dose from screening to end of treatment (EOT)). ^†^Patient’s decision (fear of being exposed to Coronavirus Disease 2019 (COVID-19)). ^‡^One patient experienced a serious adverse event (SAE) of Hodgkin’s disease; one patient experienced an SAE of duodenal ulcer perforation; and one patient experienced a non-serious event of neurodermatitis. ^§^None was related to safety issues, lack of efficacy or COVID-19. ^‖^Poor compliance to protocol. **b**, PRIME2. No patients were lost to follow-up at the time of the cutoff date. ^*^Low-to-medium potency TCS/TCI as background therapy permitted (maintain dose from screening to EOT). ^†^No discontinuations related to COVID-19. ^‡^Patient could not continue the self-administration of investigational medicinal product. ^§^None of the ‘other’ reasons for permanent study intervention discontinuation was related to safety or lack of efficacy. All were reported as the reason for withdrawal by the subject.

In PRIME, 200 patients were screened and 151 were randomized (75 dupilumab and 76 placebo) at 58 study sites in eight countries/regions. In PRIME2, 221 patients were screened and 160 were randomized (78 dupilumab and 82 placebo) at 55 study sites in 11 countries/regions (Fig. 1 for CONSORT diagrams and Supplementary Information for lists of investigators). The population sample was representative for the PN real-world sex^6,17^, age^11,16,17,35^, racial/ethnic background distribution^17^ and associated comorbidities^1,8,11,13,16,35^ (Supplementary Table 1).

Assessment scales used to measure disease severity and treatment outcomes (WI-NRS^31–33^, IGA PN-S^34^, Dermatology Life Quality Index (DLQI)^36,37^, Hospital Anxiety and Depression Scale (HADS)^38,39^, Sleep Numeric Rating Scale (NRS) and Skin Pain NRS) are detailed in Supplementary Table 2.

Baseline characteristics were generally balanced between intervention groups in both trials (Table 1). All patients had severe itch at baseline, as demonstrated by a mean (s.d.) WI-NRS score of 8.5 (1.0) in both trials and ≥20 nodules upon entry; 28.7% (PRIME) and 38.4% (PRIME2) of patients had ≥100 nodules (IGA PN-S = 4). Mean (s.d.) DLQI baseline scores were 16.7 (7.2) in PRIME and 18.2 (6.7) in PRIME2, corresponding to ‘very large’ impact of PN on life. All patients in both studies had used topical therapies in the past; 98.7% and 98.1% reported past use of TCS; and 69.5% and 63.1% had previously received systemic therapies (Table 1). The most common associated medical conditions in patients in both trials were hypertension, type 2 diabetes mellitus and hypothyroidism (Supplementary Table 3). Other baseline disease characteristics are summarized in Table 1.

**Table 1:** Demographic and clinical characteristics of the patient population at baseline

	PRIME			PRIME2		
	Placebo *n* = 76	Dupilumab *n* = 75	Overall *n* = 151	Placebo *n* = 82	Dupilumab *n* = 78	Overall *n* = 160
Mean age (s.d.), years	51.1 (15.8)	49.2 (17.4)	50.1 (16.6)	46.7 (15.2)	51.0 (15.8)	48.8 (15.6)
Female sex, *n* (%)	48 (63.2)	52 (69.3)	100 (66.2)	51 (62.2)	52 (66.7)	103 (64.4)
Mean weight (s.d.), kg	71.4 (17.0)	75.2 (17.3)	73.3 (17.2)	75.0 (19.7)	73.9 (17.5)	74.5 (18.6)
Race, *n* (%)						
White	45 (59.2)	35 (46.7)	80 (53.0)	48 (58.5)	48 (61.5)	90 (60.0)
Black or African American^a^	3 (3.9)	8 (10.7)	11 (7.3)	5 (6.1)	3 (3.8)	8 (5.0)
Asian	25 (32.9)	29 (38.7)	54 (35.8)	27 (32.9)	25 (32.1)	52 (32.5)
Others or missing data^b^	3 (4.0)	3 (4.0)	6 (3.9)	2 (2.4)	2 (2.6)	4 (2.5)
Region, *n* (%)^c^						
Asia	23 (30.3)	27 (36.0)	50 (33.1)	23 (28.0)	20 (25.6)	43 (26.9)
Eastern Europe	11 (14.5)	11 (14.7)	22 (14.6)	5 (6.1)	6 (7.7)	11 (6.9)
Latin America	22 (28.9)	19 (25.3)	41 (27.2)	8 (9.8)	6 (7.7)	14 (8.8)
Western countries	20 (26.3)	18 (24.0)	38 (25.2)	46 (56.1)	46 (59.0)	92 (57.5)
Mean duration of PN (s.d.), years	5.4 (6.2)	6.0 (7.6)	5.7 (6.9)	5.5 (7.0)	5.4 (6.9)	5.4 (6.9)
History of atopy^d^, *n* (%)	28 (38.6)	33 (44.0)	61 (40.4)	40 (48.8)	34 (43.6)	74 (46.3)
Ongoing mild AD	2 (2.6)	4 (5.3)	6 (4.0)	5 (6.1)	2 (2.6)	7 (4.4)
Stable use of TCS/TCI^e^, *n* (%)	45 (59.2)	47 (62.7)	92 (60.9)	46 (56.1)	44 (56.4)	90 (56.3)
Stable use of antidepressants, *n* (%)	9 (11.8)	9 (12.0)	18 (11.9)	8 (9.8)	7 (9.0)	15 (9.4)
Prior topical medication for PN	76 (100)	74 (98.7)	150 (99.3)	82 (100)	78 (100)	160 (100)
TCS	75 (98.7)	74 (98.7)	149 (98.7)	80 (97.6)	77 (98.7)	157 (98.1)
TCI	12 (15.8)	9 (12.0)	21 (13.9)	8 (9.8)	6 (7.7)	14 (8.8)
Prior systemic medication for PN	52 (68.4)	53 (70.7)	105 (69.5)	52 (63.4)	49 (62.8)	101 (63.1)
Antihistamines	44 (57.9)	45 (60.0)	89 (58.9)	40 (48.8)	36 (46.2)	76 (47.5)
Corticosteroids	13 (17.1)	17 (22.7)	30 (19.9)	15 (18.3)	9 (11.5)	24 (15.0)
Non-steroidal immunosuppressants	10 (13.2)	16 (21.3)	26 (17.2)	18 (22.0)	20 (25.6)	38 (23.8)
Gabapentinoids	2 (2.6)	5 (6.7)	7 (4.6)	1 (1.2)	0	1 (0.6)
Opioid receptor antagonists	2 (2.6)	2 (2.7)	4 (2.6)	1 (1.2)	2 (2.6)	3 (1.9)
Antidepressants	2 (2.6)	1 (1.3)	3 (2.0)	13 (15.9)	10 (12.8)	23 (14.4)
Mean WI-NRS (0–10) score (s.d.)	8.3 (1.1)	8.6 (0.9)	8.5 (1.0)	8.5 (1.0)	8.5 (1.0)	8.5 (1.0)
IGA PN-S (0–4), *n* (%)						
3	53 (70.7)	54 (72.0)	107 (71.3)	49 (60.5)	49 (62.8)	98 (61.6)
4	22 (29.3)	21 (28.0)	43 (28.7)	32 (39.5)	29 (37.2)	61 (38.4)
Mean Skin Pain NRS (0–10) score (s.d.)	7.2 (2.3)	7.2 (2.5)	7.2 (2.4)	7.1 (2.5)	7.3 (2.4)	7.2 (2.4)
Mean Sleep NRS (0–10) score (s.d.)	4.3 (2.2)	4.4 (2.4)	4.3 (2.3)	4.2 (2.5)	4.4 (2.3)	4.3 (2.4)
Mean DLQI (0–30) score (s.d.)	15.7 (7.3)	17.8 (7.1)	16.7 (7.2)	18.2 (7.0)	18.2 (6.5)	18.2 (6.7)
Mean total HADS (0–42) score (s.d.)	14.3 (8.0)	14.5 (8.2)	14.4 (8.1)	15.9 (8.4)	16.2 (7.7)	16.0 (8.0)
Anxiety (HADS-A)	8.3 (4.6)	8.5 (5.2)	8.4 (4.9)	9.5 (5.1)	9.3 (4.2)	9.4 (4.6)
Depression (HADS-D)	6.0 (4.1)	6.0 (3.8)	6.0 (3.9)	6.3 (4.0)	6.9 (4.0)	6.6 (4.0)
Mean baseline exact number of lesions in representative area from PAS (s.d.)	25.1 (16.7)	27.0 (26.7)	26.1 (22.2)	26.4 (18.8)	25.6 (18.7)	26.0 (18.7)

Note: Higher score indicates worse disease/larger impact, except for Patient Sleep Quality NRS, where higher score indicates better sleep quality.

^a^31.4% of PRIME patients from the United States and 55.6% of PRIME2 patients from the United States were Black or African American.

^b^Including American Indian, Alaska Native, Native Hawaiian or Pacific Islands, unknown.

^c^PRIME: Argentina, China, France, Japan, Mexico, Russia, South Korea, United States; PRIME2: Canada, Chile, France, Hungary, Italy, Portugal, South Korea, Spain, Taiwan, United Kingdom, United States.

^d^Defined as having a medical history of AD, allergic rhinitis/rhinoconjunctivitis, asthma or food allergy.

^e^Defined as maintaining the same medicine (low-to-medium-potency TCS or TCI) and maintaining the same frequency of treatment (once or twice daily) used from 2 weeks before screening.

### Efficacy analyses

A study-level multiplicity procedure was used to control for the overall type I error rate for testing primary, key secondary and selected other endpoints (Methods and Extended Data Table 1). *P* values less than 0.05 were considered statistically significant if the endpoint was included in the testing hierarchy.

### Primary endpoint

The primary endpoint in both trials addressed clinically meaningful itch improvement. A weekly WI-NRS score was calculated as the average of daily non-missing scores within the week window of each trial week. The trials were originally designed similarly, with week 12 as the timing for the primary endpoint. However, results from PRIME2, which preceded PRIME, showed continued improvement of itch after week 12. To represent more precisely the effect of the treatment on itch and to harmonize the assessment of the itch and lesion endpoint evaluations, a protocol amendment was submitted and approved while the PRIME study was ongoing to change the timing for the primary endpoint to week 24.

**Fig. 2:**
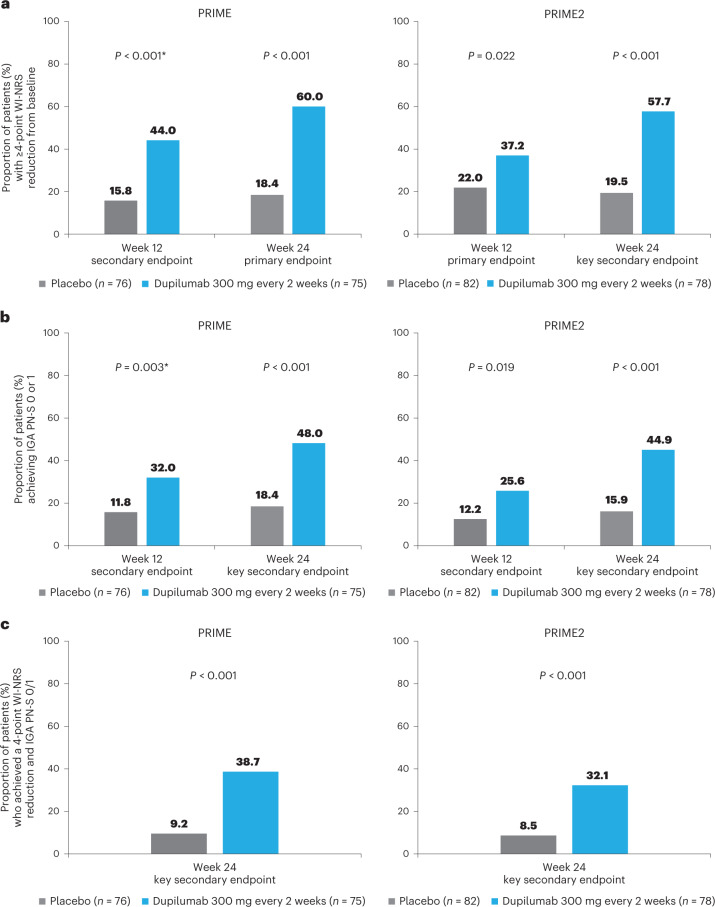
Efficacy outcomes. **a**, Proportion of patients who achieved WI-NRS improvement (reduction) by ≥4 points from baseline at week 12 and week 24**. b**, Proportion of patients who achieved IGA PN-S score of 0 or 1 (‘clear’ or ‘almost clear’) at week 12 and week 24. **c**, Proportion of patients who achieved concomitantly WI-NRS reduction from baseline by ≥4 points and IGA PN-S of score 0 or 1 at week 24 in PRIME and PRIME2. *Endpoint not in the testing hierarchy. Cochran–Mantel–Haenszel test was performed on the association between the responder status and intervention group, adjusted by documented history of atopy (atopic or non-atopic), stable use of TCS/TCI (yes or no), region and baseline antidepressant use (yes or no).

Significantly more dupilumab-treated patients achieved the primary endpoint of a ≥4-point reduction in WI-NRS compared to placebo-treated patients in both trials: 45/75 (60.0%) versus 14/76 (18.4%) at week 24 in PRIME (95% confidence interval (CI), 27.8–57.7 for the difference; *P* < 0.001); 29/78 (37.2%) versus 18/82 (22.0%) at week 12 in PRIME2 (95% CI, 2.3–31.2 for the difference; *P* = 0.022) (Table 2, Fig. 2a and Supplementary Table 4). Patients with missing data (PRIME, week 24: 1 (1.3%) in the dupilumab group and 16 (21.1%) in the placebo group; PRIME2, week 12: 2 (2.6%) and 6 (7.3%), respectively) were considered non-responders due to missing data (see Supplementary Table 5 for a summary of missing data).

**Table 2:** Efficacy outcomes

Efficacy endpoints	PRIME				PRIME2			
	Placebo *n* = 76	Dupilumab 300 mg every 2 weeks *n* = 75	Difference versus placebo, % (95% CI)	*P* value versus placebo (place in the testing hierarchy)	Placebo *n* = 82	Dupilumab 300 mg every 2 weeks *n* = 78	Difference versus placebo, % (95% CI)	*P* value versus placebo (place in the testing hierarchy)
Primary and key secondary outcomes								
WI-NRS improvement (reduction) by ≥4 from baseline to week 24^a^, *n* (%)	14 (18.4)	45 (60.0)	42.7 (27.8 to 57.7)	<0.001 (1)	16 (19.5)	45 (57.7)	42.6 (29.1 to 56.1)	<0.001 (2)
WI-NRS improvement (reduction) by ≥4 from baseline to week 12^b^, *n* (%)	12 (15.8)	33 (44.0)	29.2 (14.5 to 43.8)	<0.001 (not multiplicity-controlled)	18 (22.0)	29 (37.2)	16.8 (2.3 to 31.2)	0.022 (1)
IGA PN-S score of 0 or 1 (‘clear’ or ‘almost clear’) at week 24^c^, *n* (%)	14 (18.4)	36 (48.0)	28.3 (13.4 to 43.2)	<0.001 (2)	13 (15.9)	35 (44.9)	30.8 (16.4 to 45.2)	<0.001 (3)
Concomitant WI-NRS improvement (reduction) by ≥4 points from baseline and IGA PN-S score of 0 or 1 at week 24^d^, *n* (%)	7 (9.2)	29 (38.7)	29.6 (16.4 to 42.8)	<0.001 (3)	7 (8.5)	25 (32.1)	25.5 (13.1 to 37.9)	<0.001 (4)
Other multiplicity-controlled endpoints								
Percent change from baseline in WI-NRS at week 24, LS mean (s.e.)	−22.2 (5.7)	−48.9 (5.6)	−26.7 (−38.4 to −14.9)	<0.001 (4)	−36.2 (6.2)	−59.3 (6.4)	−23.2 (−33.8 to −12.5)	<0.001 (6)
IGA PN-S score of 0 or 1 (‘clear’ or ‘almost clear’) at week 12^ǁ^, *n* (%)	9 (11.8)	24 (32.0)	20.9 (7.8 to 34.0)	0.003 (not multiplicity-controlled)	10 (12.2)	20 (25.6)	14.8 (2.6 to 27.0)	0.019 (5)
Change from baseline in DLQI at week 24, LS mean (s.e.)	−5.8 (1.0)	−12.0 (1.0)	−6.1 (−8.3 to −4.0)	<0.001 (5)	−6.8 (1.2)	−13.2 (1.2)	−6.4 (−8.4 to −4.4)	<0.001 (7)
Change from baseline in Skin Pain NRS at week 24, LS mean (s.e.)	−2.2 (0.4)	−4.3 (0.4)	−2.2 (−3.1 to −1.3)	<0.001 (6)	−2.7 (0.5)	−4.4 (0.5)	−1.6 (−2.5 to −0.7)	<0.001 (8)
Change from baseline in total HADS score to week 24, LS mean (s.e.)	−2.0 (0.9)	−4.6 (0.9)	−2.6 (−4.5 to −0.7)	0.008 (7)	−2.6 (1.0)	−5.6 (1.1)	−3.0 (−4.7 to −1.2)	0.001 (10, tested after hierarchy broke)
Anxiety, HADS-A (0–21)	−1.2 (0.5)	−2.7 (0.6)	−1.5 (−2.7 to −0.4)	0.008 (non multiplicity-controlled)	−1.9 (0.9)	−3.3 (0.7)	−1.4 (−2.5 to −0.3)	0.012 (non multiplicity-controlled)
Depression, HADS-D (0–21)	−0.9 (0.5)	−1.9 (0.5)	−1.1 (−2.0 to −0.1)	0.033 (non-multiplicity-controlled)	−0.5 (0.5)	−2.1 (0.5)	−1.6 (−2.5 to −0.7)	<0.001 (non-multiplicity-controlled)
Change from baseline in Sleep NRS at week 24, LS mean (s.e.)	1.3 (0.3)	2.7 (0.3)	1.4 (0.8 to 2.1)	<0.001 (non multiplicity-controlled)	0.8 (0.5)	1.3 (0.5)	0.5 (−0.2 to 1.3)	0.165 (9; hierarchy broke here)
Non-multiplicity-controlled endpoints								
Use of concomitant medication/procedures or rescue medication through week 24, *n* (%)	22 (28.9)	8 (10.7)	N/A	N/A	26 (31.7)	11 (14.1)	N/A	N/A
Prohibited medications	13 (17.1)	4 (5.3)	N/A	N/A	9 (11.0)	3 (3.8)	N/A	N/A
Prohibited procedures	2 (2.6)	0	N/A	N/A	1 (1.2)	2 (2.6)	N/A	N/A
Rescue medication	16 (21.1)	5 (6.7)	0.2 (0.1 to 0.6)	0.004 (non multiplicity-controlled)	20 (24.4)	6 (7.7)	0.3 (0.1 to 0.7)	0.004 (non multiplicity-controlled)

Unless otherwise indicated, efficacy analyses were performed in the full analysis set, which included all patients who underwent randomization. The primary and secondary endpoints were tested with a hierarchical testing procedure in a pre-specified order, and inferential conclusions about successive endpoints required statistical significance of the previous endpoint at a two-sided significance level of 0.05. For endpoints that measured binary responses, *P* values were derived by a Cochran–Mantel–Haenszel test. The difference versus placebo is the response rate difference derived from the Mantel–Haenszel estimator. For continuous efficacy endpoints, *P* values and difference versus placebo were derived by an analysis of covariance (ANCOVA) model. For the endpoint of rescue medication, the difference versus placebo is based on the time to first use of rescue medication and was calculated as hazard ratio of dupilumab versus placebo; *P* values were derived from the Cox proportional hazard model.

^a^Primary endpoint in PRIME, key secondary endpoint in PRIME 2.

^b^Primary endpoint in PRIME 2, secondary endpoint in PRIME.

^c^Key secondary endpoint PRIME and PRIME2.

^d^Key secondary endpoint, United States and United States reference countries only.

N/A, not available.

### Secondary endpoints addressing itch

**Fig. 3:**
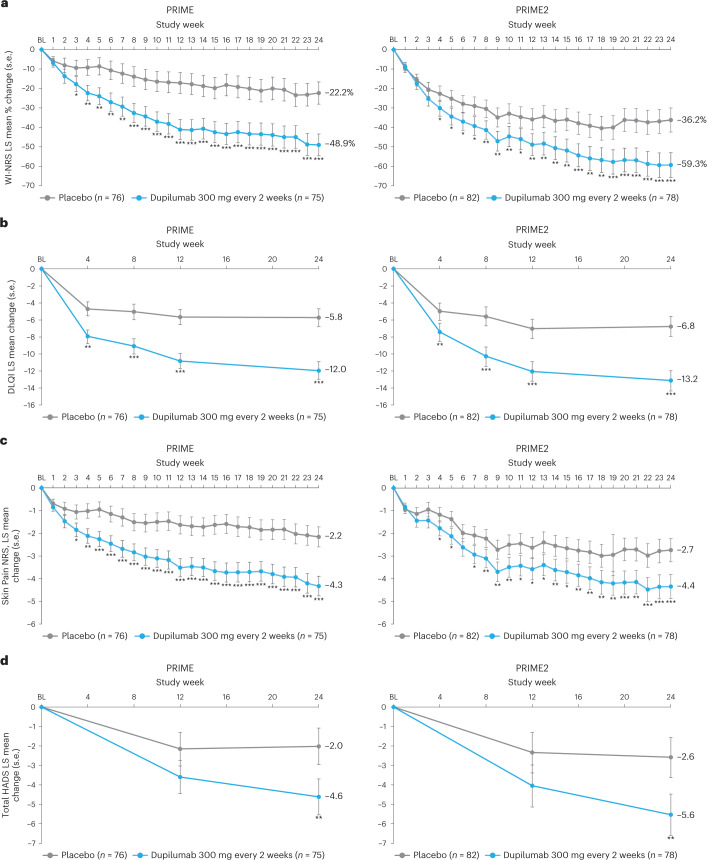
Patient-reported outcomes. **a**, LS mean percent change in WI-NRS (s.e.) from baseline (BL) through week 24. **b**, LS mean change (s.e.) in DLQI from BL to week 24**. c**, LS mean change (s.e.) in Skin Pain NRS from BL through week 24 in PRIME and PRIME2. **d**, LS mean change (s.e.) in HADS from BL through week 24 in PRIME and PRIME2. *P < 0.05, **P < 0.01, ***P < 0.001. Data were presented as mean ± s.e. The imputed complete data were analyzed by fitting an analysis of covariance (ANCOVA) model with the corresponding BL value, intervention group, documented history of atopy (atopic or non-atopic), stable use of TCS/TCI (yes or no), region and BL antidepressant use (yes or no) as covariates. *P* values at week 24 are multiplicity-controlled except for LS mean change in total HADS in PRIME2. *P* values for all the other timepoints are non-multiplicity-controlled.

**Extended Data Fig. 2:**
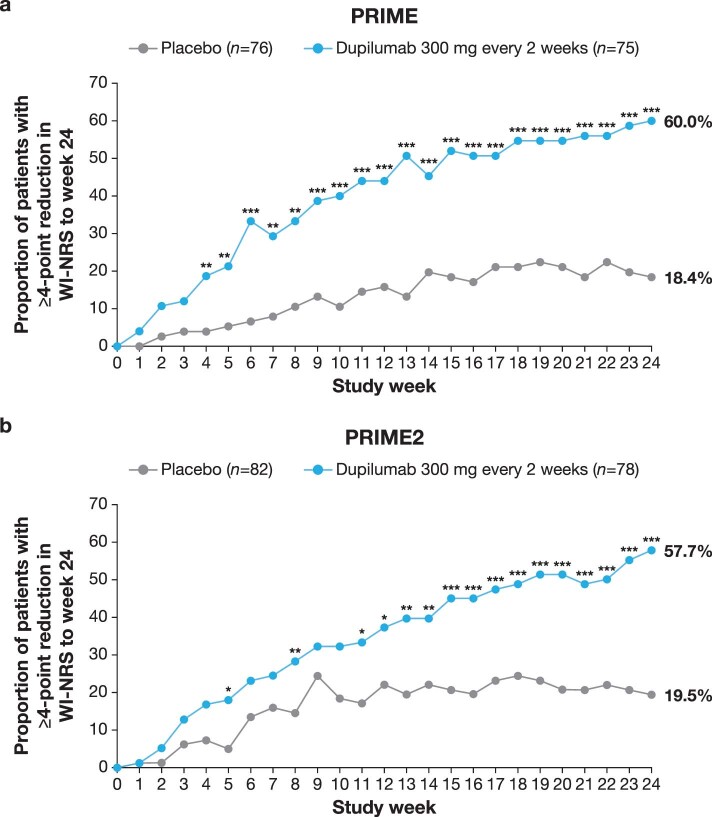
Proportion of patients with WI-NRS score reduction. Patients with WI-NRS score reduction by ≥4 points over time, by visit, to week 24 in PRIME (**a**) and PRIME2 (**b**). *P < 0.05; **P < 0.01; ***P < 0.001. ‘Responder’ was defined as a patient with WI-NRS reduction ≥ 4 from baseline. Cochran-Mantel Haenszel test was performed on the association between responder status and intervention group, adjusted by documented history of atopy (atopic/non-atopic), stable use of TCS/TCI (yes/no), region and baseline antidepressant use (yes/no). *P* values are non multiplicity-controlled. TCI, topical calcineurin inhibitor; TCS, topical corticosteroids; WI-NRS, Worst Itch Numerical Rating Scale.

Proportion of patients achieving a ≥4-point reduction in WI-NRS was also higher in the dupilumab group compared to placebo at week 12 in PRIME: 44% versus 15.8% (95% CI for the difference, 14.5–43.8; non-multiplicity-controlled *P* < 0.001) and at week 24 in PRIME2 (key secondary endpoint): 57.7% versus 19.5% (95% CI for the difference, 29.1–56.1; *P* < 0.001) (Table 2, Fig. 2a and Supplementary Table 5). Compared to placebo, non-multiplicity-controlled significant improvements in least squares (LS) mean percent change in weekly average WI-NRS with dupilumab were observed as early as week 3 in PRIME and week 4 in PRIME2 (Fig. 3a). The proportion of patients achieving a ≥4-point reduction in WI-NRS was significantly higher with dupilumab than with placebo starting at week 4 in PRIME and week 5 in PRIME2 (non-multiplicity-controlled *P* versus placebo <0.05) (Extended Data Fig. 2).

### Secondary endpoints addressing skin lesions

**Extended Data Fig. 3:**
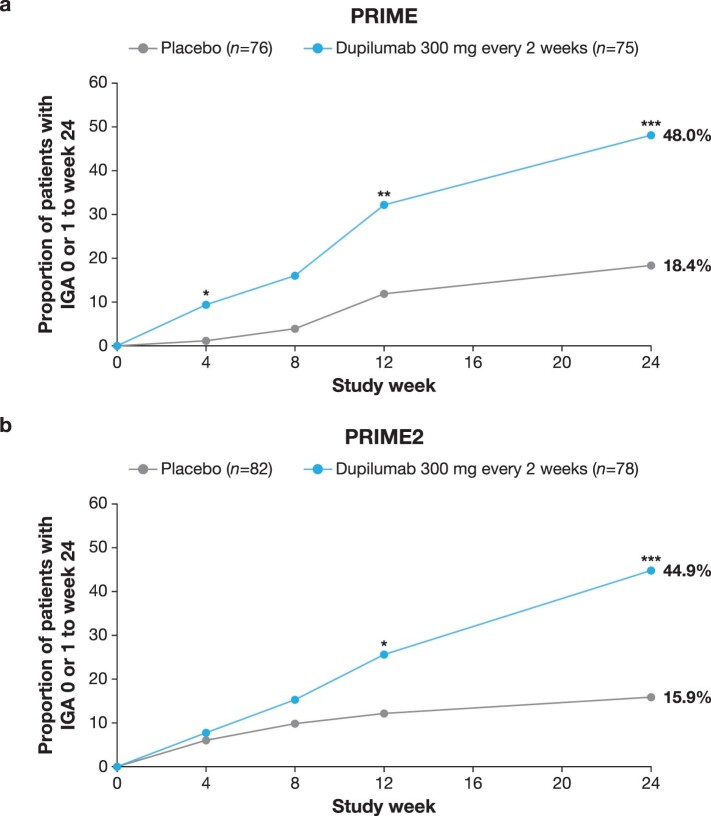
Proportion of patients with IGA PN-S score 0 or 1. Patients with IGA PN-S score 0/1 over time, by visit, to week 24 in PRIME (**a**) and PRIME2 (**b**). *P < 0.05; **P < 0.01; ***P < 0.001. ‘Responder’ was defined as a patient with IGA PN-S score of 0 or 1. Cochran-Mantel Haenszel test was performed on the association between responder status and intervention group, adjusted by documented history of atopy (atopic/non-atopic), stable use of TCS/TCI (yes/no), region and baseline antidepressant use (yes/no). *P* values are non multiplicity-controlled.IGA PN-S, Investigator’s Global Assessment for PN-Stage; TCI, topical calcineurin inhibitors; TCS, topical corticosteroids.

Significantly more dupilumab-treated patients achieved an IGA PN-S score of 0 or 1 (‘clear’ or ‘almost clear’, ≤5 nodules) in each trial at week 24: PRIME, 48.0% versus 18.4% (95% CI for the difference, 13.4–43.2; *P* < 0.001); PRIME2, 44.9% versus 15.9% (95% CI for the difference, 16.4–45.2; *P* < 0.001). At week 12, this endpoint was achieved by 32.0% versus 11.8% of patients in PRIME (95% CI for the difference, 7.8–34.0; non-multiplicity-controlled *P* = 0.003) and 25.6% versus 12.2% in PRIME2 (95% CI for the difference, 2.6–27.0; *P* = 0.019) (Fig. 2b, Extended Data Fig. 3 for proportions of patients achieving IGA 0/1 over time and Supplementary Table 5 for a summary of missing data). Significantly more patients achieved the composite endpoint (key secondary endpoint in both trials) of a concomitant ≥4-point reduction in WI-NRS from baseline and an IGA PN-S score of 0 or 1 at week 24 in the dupilumab group (29 (38.7%) and 25 (32.1%) in PRIME and PRIME 2, respectively) compared to seven patients in each placebo group (9.2% and 8.5%, 95% CI, 16.4–42.8 and 13.1–37.9 for the difference, respectively; *P* < 0.001 for both comparisons), demonstrating efficacy on pruritus and skin lesions within the same patient (Fig. 2c).

Supplementary (as-observed and hybrid method) analyses results for primary and key secondary endpoints in both trials were consistent with the primary analysis, confirming the robustness of results (Extended Data Table 2).

### Other multiplicity-controlled endpoints

**Extended Data Fig. 4:**
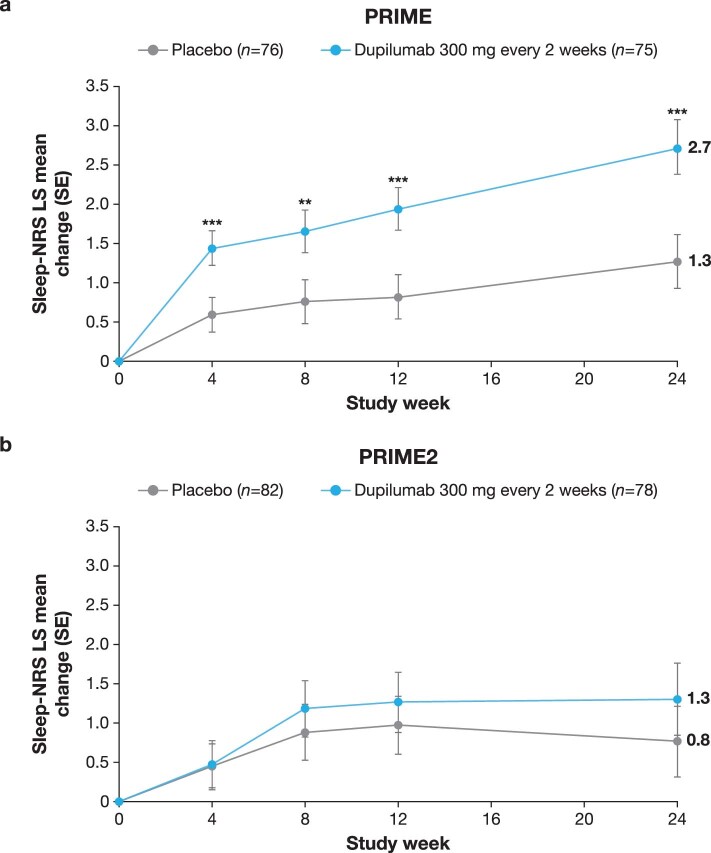
Least-squares mean change in Sleep NRS score over time to week 24 in PRIME (a) and PRIME2 (b). Data are presented as mean values ± SE. Each of the imputed complete data were analyzed by fitting an ANCOVA model with the corresponding baseline value, intervention group, documented history of atopy (atopic/non-atopic), stable TCS/TCI use (yes/no), region and baseline antidepressant use (yes/no) as covariates. *P* values are non multiplicity-controlled. LS, least squares; NRS, Numeric Rating Scale; SE, standard error; TCI, topical calcineurin inhibitors; TCS, topical corticosteroids.

Additional multiplicity-controlled endpoints in both trials included changes from baseline in DLQI, Skin Pain NRS and HADS at week 24 and, in PRIME2 only, change in Sleep NRS at week 24 (Extended Data Table 1 for the testing hierarchy). Dupilumab-treated patients showed significant improvements in QoL compared to placebo-treated patients, as measured by LS mean change (±s.e.) in DLQI score from baseline at week 24: PRIME, −12.0 (1.0) versus −5.8 (1.0); PRIME2, −13.2 (1.2) versus −6.8 (1.2) (95% CI, −8.3 to −4.0 and −8.4 to −4.4 for the difference, respectively; both *P* < 0.001) (Table 2 and Fig. 3b). Significant improvements in skin pain were also observed, as measured by LS mean change (s.e.) in Skin Pain NRS at week 24: PRIME, −4.3 (0.4) versus −2.2 (0.4); PRIME2, −4.4 (0.5) versus −2.7 (0.5) (95% CI, −3.1 to −1.3 and −2.5 to −0.7 for the difference, respectively; both *P* < 0.001) (Table 2 and Fig. 3c). Statistical (PRIME) or non-multiplicity-controlled (PRIME2) significant improvements in anxiety and depression, as measured by LS mean change (s.e.) from baseline in total HADS at week 24, were observed in both studies (Table 2 and Fig. 3d). Change from baseline in Sleep NRS at week 24 is shown in Table 2 and over time in Extended Data Fig. 4.

Efficacy outcomes were similar between atopic and non-atopic patients as well as those who used TCS/TCI throughout the trial compared to those who did not (Extended Data Tables 3 and 4).

### Use of rescue medication

**Extended Data Fig. 5:**
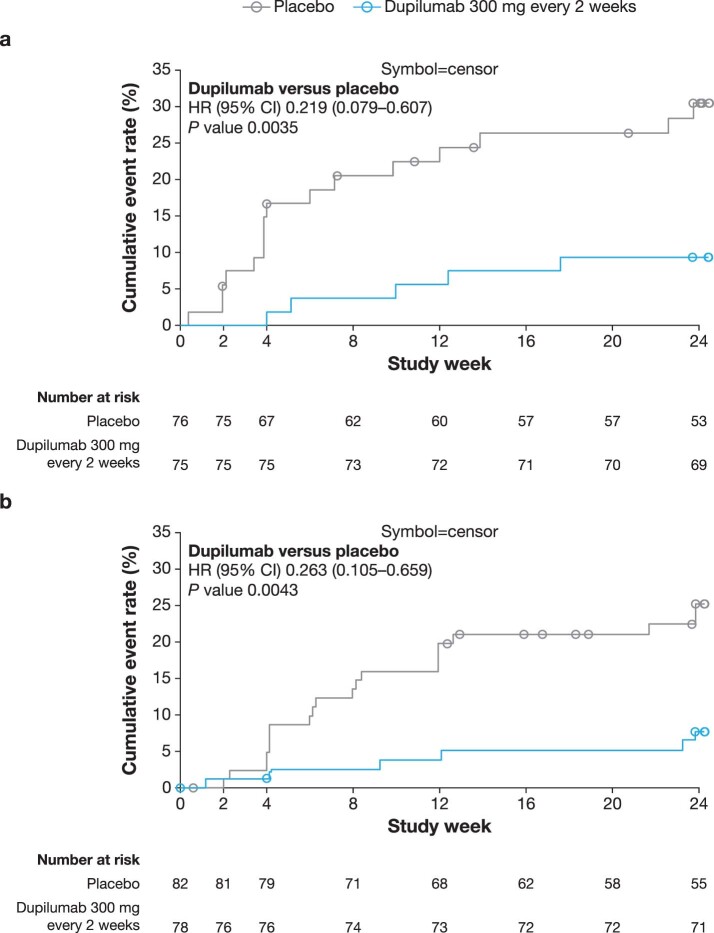
Time to first use of rescue medication. Kaplan–Meier curves of time to first use of rescue medication in PRIME (**a**) and PRIME2 (**b**). HR and non multiplicity-controlled *P* values were derived from Cox proportional hazards model, including intervention, documented history of atopy (atopic/non-atopic), stable use of TCS/TCI (yes/no), region and baseline antidepressant use (yes/no) as covariates. CI, confidence interval; HR, hazard ratio; TCI, topical calcineurin inhibitors; TCS, topical corticosteroids.

Fewer dupilumab-treated patients required rescue medication compared to those given placebo during the 24-week studies (PRIME, 6.7% versus 21.1%; PRIME2, 7.7% versus 24.4%; non-multiplicity-controlled *P* versus placebo = 0.004 in both trials) (Table 2 and Extended Data Fig. 5).

Results for additional efficacy endpoints are summarized in Extended Data Table 5.

This manuscript reports on all the multiplicity-controlled and pre-specified supportive endpoints included in the PRIME and PRIME2 trials. Additional pre-specified secondary and tertiary efficacy endpoints not reported in this manuscript are listed in the Methods section and will be reported in subsequent publications.

### Safety outcomes

In both trials, dupilumab was well tolerated and had an overall safety profile consistent with its known profile (Table 3 and Supplementary Tables 6–8). Treatment-emergent serious adverse events were reported in five (6.7%) and six (8.0%) patients in the dupilumab and placebo groups, respectively, in PRIME, and two (2.6%) and two (2.4%), respectively, in PRIME2. Except for two events of mesenteritis and sepsis experienced by one patient in the placebo group in PRIME, none were considered related to the study intervention. Two placebo-treated patients (2.7%) in PRIME and one placebo-treated patient (1.2%) in PRIME2 discontinued treatment due to a treatment-emergent adverse event (TEAE); no dupilumab-treated patients discontinued treatment. Conjunctivitis occurred equally in the dupilumab and placebo groups in PRIME (two (2.7%) in each) and was more frequent with dupilumab in PRIME2 (three (3.9%) versus zero). None were serious or severe, and none led to study drug discontinuation. Herpes viral infections were also more common with dupilumab in PRIME2: four (5.2%) versus zero, whereas no herpes infections occurred in either group in PRIME. Skin infections occurred less in dupilumab-treated patients than placebo-treated patients in both trials: PRIME, two (2.7%) versus seven (9.3%); PRIME2, four (5.2%) versus five (6.1%).

**Table 3:** Safety outcomes

TEAEs, *n* (%)	PRIME		PRIME2	
	Placebo *n* = 75	Dupilumab *n* = 75	Placebo *n* = 82	Dupilumab *n* = 77
Patients with ≥1 TEAE	42 (56.0)	49 (65.3)	38 (46.3)	42 (54.5)
Patients with any severe adverse event^a^	5 (6.7)	3 (4.0)	1 (1.2)	2 (2.6)
Patients with any treatment-emergent SAE	6 (8.0)	5 (6.7)	2 (2.4)	2 (2.6)
Deaths	0	0	0	0
Patients with TEAE leading to treatment discontinuation^b^	2 (2.7)	0	1 (1.2)	0
TEAEs reported in ≥5% of patients in any treatment group (MedDRA PT), *n* (%)				
Nasopharyngitis	3 (4.0)	4 (5.3)	0	2 (2.6)
Headache	4 (5.3)	4 (5.3)	5 (6.1)	4 (5.2)
COVID-19	4 (5.3)	0	1 (1.2)	1 (1.3)
Neurodermatitis	6 (8.0)	1 (1.3)	3 (3.7)	2 (2.6)
TEAE groups of interest, *n* (%)				
Herpes viral infections (HLT)^c^	0	0	0	4 (5.2)
Skin infections (excluding herpetic infections)^d^	7 (9.3)	2 (2.7)	5 (6.1)	4 (5.2)
Conjunctivitis (narrow)^e^	2 (2.7)	2 (2.7)	0	3 (3.9)
Coronavirus infections (HLT)^f^	4 (5.3)	1 (1.3)	3 (3.7)	1 (1.3)
COVID-19	4 (5.3)	0	1 (1.2)	1 (1.3)
Asymptomatic COVID-19	0	0	2 (2.4)	
COVID-19 pneumonia	0	1 (1.3)	0	0

Included in the analysis were all patients in the safety analysis set, which included all randomized patients who received ≥1 dose of dupilumab or placebo. Patients are listed according to the study drug they received, which may differ from the randomized group. Results are reported for the 24 weeks of treatment.

^a^Considered unrelated to the study intervention except for two events of sepsis and mesenteritis, experienced by one placebo-treated patient in PRIME.

^b^In PRIME, one event each of Hodgkin’s disease and neurodermatitis (MedDRA PTs), considered unrelated to the study drug. In PRIME2, one event of urticaria.

^c^Herpes viral infections (HLT) includes MedDRA PTs oral herpes, herpes zoster, ophthalmic herpes zoster and genital herpes simplex. None of these TEAEs was severe, and all patients recovered with corrective treatment while continuing dupilumab.

^d^Skin infection TEAEs (excluding herpetic infections) were identified based on blinded medical review of all reported TEAEs identified as possible skin infections using CMQ30067. This search included MedDRA PTs under HLGT Skin and subcutaneous tissue infections and infestations, all MedDRA PTs under HLT Skin structures and soft tissue infections, all MedDRA PTs of ‘wound infection’ and MedDRA PTs of chalazion, hordeolum and skin papilloma.

^e^Conjunctivitis (narrow term) includes MedDRA PTs conjunctivitis, conjunctivitis bacterial, conjunctivitis viral, conjunctivitis adenoviral, conjunctivitis allergic and atopic keratoconjunctivitis.

^f^Coronavirus infections (HLT) include MedDRA PTs COVID-19, asymptomatic COVID-19 and COVID-19 pneumonia.

COVID-19, Coronavirus Disease 2019; HLGT, MedDRA High-Level Group Term; HLT, MedDRA High-Level Term; MedDRA, Medical Dictionary for Regulatory Activities; PT, MedDRA Preferred Term.

## Discussion

Management of PN is challenging^2,18,19^, particularly for patients with moderate or severe PN for whom topical therapies are, in many cases, insufficient to control the disease^20–22^. Other treatments currently used for PN also have limitations, including unsatisfactory effectiveness and associated side effects and toxicities^23^. In PRIME and PRIME2, dupilumab compared to placebo significantly improved multiple measures of signs and symptoms as well as health-related QoL in patients with PN. These data represent the first replicate positive results from two phase 3, randomized, placebo-controlled global trials.

Given the seminal role of pruritus and resultant scratching in the patient experience with PN, as exemplified by the ‘butterfly sign’ where there is an absence of lesions in regions of the body that the patient cannot easily reach, this was chosen as the primary endpoint for the trial^40^. The 24-week treatment period was considered an appropriate duration to observe dupilumab’s effect in PN to ensure an adequate evaluation of PN lesions based on the observation that dupilumab has shown clinical efficacy and biomarker data, including thymus and activation-regulated chemokine (TARC) and eotaxin-3, plateauing before week 24 in all phase 3 clinical trials across all dupilumab indications^41^.

Despite previous use of topical and, in over 60% of patients, systemic therapies, the patients enrolled in PRIME and PRIME2 had a high disease burden at baseline, with severe itch and skin lesions impacting sleep, QoL and mental health. All primary and key secondary endpoints addressing itch and number of skin lesions were met in both trials. Non-multiplicity-controlled significant itch improvement started as early as week 3 or 4, with progressively larger treatment differences from placebo throughout the 24-week treatment period. From an initial baseline of 20 to >100 nodules, 32.0% (PRIME) and 25.6% (PRIME2) of dupilumab-treated patients showed a reduction to ≤5 nodules, corresponding to a response of ‘clear’ or ‘almost clear’ skin at week 12, compared to 11.8% and 12.2% of placebo-treated patients; the treatment effect on skin lesions continued to improve after week 12, with 48.0% (PRIME) and 44.9% (PRIME2) of patients achieving ≤5 nodules at week 24 with dupilumab versus 18.4% and 15.9%, respectively, with placebo. Approximately one-third of dupilumab-treated patients met the composite pruritus and skin lesion endpoint at the end of treatment. Dupilumab treatment also led to fewer patients using rescue medication compared to placebo in both trials.

Improvements in skin pain mirrored those in itch throughout both trials, with non-multiplicity-controlled significant improvement starting at week 3 and week 4 in PRIME and PRIME2, respectively. Dupilumab-treated patients experienced non-multiplicity-controlled significant improvement in DLQI from week 4 and, by the end of the treatment, achieved mean DLQI scores at the threshold between ‘small’ and ‘moderate’ impact on life in both trials, whereas the ‘very large’ impact of PN on QoL at baseline was maintained in placebo groups to week 24. Improvements (non-multiplicity-controlled significance in PRIME2) were also seen for HADS anxiety and depression.

The safety profile of dupilumab observed in PN was consistent with its known safety profile^42–45^. TEAEs most frequently reported with dupilumab versus placebo were conjunctivitis and herpes viral infection, none of which was serious/severe or led to treatment discontinuation. In contrast, non-herpetic skin infections occurred more often in placebo-treated patients.

More than 50% of patients enrolled did not have an atopic background, and only 4% had concomitant mild AD. Efficacy was consistent regardless of the assessed atopic or non-atopic status, although patients with moderate-to-severe atopic dermatitis were not included in this study. These results support PN as a disease independent from AD. Thus, whether one has concomitant AD or not, the pathophysiology of PN may be highly conserved in terms of nodules yet distinct from AD lesions.

Concomitant TCS/TCI therapy was allowed especially because topical treatments are the standard of care in clinical practice, and patients who experienced severe disease could continue with the standard of care, but higher placebo responses were observed in patients who did not use concomitant TCS/TCI compared to those who did. Possible reasons include the fact that patients who continued TCS/TCI use during the trials in each group were those with more severe disease. It is, therefore, not unexpected that improvements should come more easily in patients who started with less severe disease at baseline.

Dupilumab, through its blockade of IL-4 and IL-13 signaling, may impact PN pathogenesis in multiple ways^46^. Epithelial-derived cytokines are released in response to chronic scratching in PN, leading to upregulation of IL-4 and other type 2 cytokines in PN lesions that promote further inflammatory response^47–50^. Type 2 cytokines can also directly activate sensory neurons in the skin^51–53^, thus bridging the immune and neural dysregulation in PN^48,54–56^. IL-4Rα is increased in PN lesions^47^, and its activation sensitizes sensory neurons to the effects of other pruritogens, thereby amplifying the itch response^51^. IL-13 plasma levels are also increased in patients with PN compared to healthy control patients^57^. Treatment with dupilumab blocks this pathway, potentially breaking the pathologic itch–scratch cycle. Additionally, IL-4Rα is expressed on fibroblasts, and IL-4 and IL-13 have been implicated in promoting cutaneous fibrosis^58,59^.

Dupilumab specifically targets the IL-4/IL-13 cytokine axis and has not been associated with systemic immunosuppression, as suggested by the lower incidence of non-herpetic skin infections and absence of systemic infections in the dupilumab groups compared to placebo.

In our studies, improvements in WI-NRS, Skin Pain NRS and DLQI increased progressively over 24 weeks of treatment without plateauing, indicating that further treatment could lead to continued improvements. The relatively short duration of treatment in this study for this chronic disease precluded assessment of efficacy maintenance beyond 24 weeks. Also, of the atopic population enrolled, mild, active AD was capped at 10%, limiting the strength of statistical analysis for this subpopulation. Lastly, although patients had high compliance with the daily diary, with over 90% at week 12 and 85% at week 24 completing the majority of the days for both studies (Supplementary Table 4), missing data from a patient-reported outcome are acknowledged as a potential weakness.

In conclusion, dupilumab treatment in PRIME and PRIME2 led to significant improvements in multiple aspects of PN in patients with disease inadequately controlled with topical therapies, with a safety profile consistent with its known safety profile. These positive studies support the involvement of type 2 cytokines in driving PN disease pathogenesis and the targeting of the IL-4/IL-13 axis as a novel therapeutic paradigm for patients with PN.

## Methods

### Study design

PRIME and PRIME2 were phase 3, randomized, placebo-controlled, double-blind, multicenter, parallel-group, 24-week trials designed to evaluate efficacy and safety of dupilumab in adults with PN inadequately controlled with topical prescription therapies. Patients were enrolled in 16 countries in North and South America, Europe and Asia, from 12 December 2019 to 3 February 2022 (PRIME) and 16 January 2020 to 22 November 2021 (PRIME2). Each study had a 2–4-week screening period, followed by a 24-week intervention period and a 12-week post-treatment follow-up period (Extended Data Fig. 1).

The PRIME and PRIME2 protocols were developed by the sponsors, Sanofi and Regeneron Pharmaceuticals Inc. (redacted protocols are provided in Supplementary Data 1). Data were collected by the investigators and analyzed by the sponsors. The studies were conducted in accordance with the Declaration of Helsinki, the International Conference on Harmonisation Good Clinical Practice guideline and applicable regulatory requirements. The local institutional review board/ethics committee at each study center oversaw trial conduct and documentation (institutional review board from 7 February 2020; Supplementary Data 2; the complete list of investigators is provided in the Supplementary Information). All patients provided written informed consent before participating in the trial.

### Patients

Patients were enrolled in PRIME or PRIME2 if all the following inclusion criteria applied:

- Male or female, 18–80 years of age at the time of signing the informed consent
- PN diagnosed by a dermatologist for at least 3 months before the screening visit
- WI-NRS score of ≥7 in the 7 days before day 1 (on a scale of 0–10)
  Baseline pruritus NRS average score for maximum itch intensity was determined based on the average of daily NRS scores for maximum intensity (the daily score ranges from 0 to 10) during the 7 days immediately preceding randomization (a minimum of four daily scores out of the 7 days is required to calculate the baseline average score); for patients who did not have at least four daily scores reported during the 7 days immediately preceding the planned randomization date, randomization was postponed until this requirement was met but without exceeding the 28-day maximum duration of the screening period.
- ≥20 PN lesions in total on both legs, and/or both arms and/or trunk, at screening visit and on day 1
  Patients needed to have bilaterally symmetrical lesions on the extremities; presence of lesions on at least two body surface areas was required.
- History of failing a 2-week course of medium-to-super-potent TCS or when TCS were not medically advisable
  Failure was defined as patients who had been unable to achieve and/or maintain remission and low disease activity (similar to IGA PN-S score of ≤2 (≤19 nodules)) despite treatment with a daily regimen of medium-to-super-potent TCS (±TCI as appropriate), applied for at least 14 days or for the maximum duration recommended by the product prescribing information, whichever was shorter.
- Had applied a stable dose of topical emollient (moisturizer) once or twice daily for at least five out of the seven consecutive days immediately before day 1
- Was willing and able to complete a daily symptom eDiary for the duration of the study
- Contraceptive use by women was consistent with local regulations regarding the methods of contraception for those participating in clinical studies
- Female patients were eligible to participate if they were not pregnant or breastfeeding and at least one of the following conditions applied:
  Is not a woman of childbearing potential (WOCBP)
  OR
  Is a WOCBP and agreed to use a contraceptive method during the study (at a minimum until 12 weeks after the last dose of study intervention)
  A WOCBP must have had a negative highly sensitive pregnancy test (urine or serum as required by local regulations) on day 1 before the first dose of study intervention.
  If a urine test could not be confirmed as negative (for example, an ambiguous result), a serum pregnancy test would be required; in such cases, the patient would be excluded from participation if the serum pregnancy result was positive.
- The investigator was responsible for review of medical history, menstrual history and recent sexual activity to decrease the risk for inclusion of a woman with an early undetected pregnancy.
- Is capable of giving signed informed consent, which includes compliance with the requirements and restrictions listed in the informed consent form (ICF) and in the study protocol; in countries where legal age of majority is above 18 years, a specific ICF was signed by the patient’s legally authorized representative.
  Patients were not eligible if any of the following exclusion criteria applied:
- Presence of skin morbidities other than PN and mild AD that may interfere with the assessment of the study outcomes
  Conditions such as, but not limited to, the following: scabies, insect bite, lichen simplex chronicus, psoriasis, acne, folliculitis, habitual picking, lymphomatoid papulosis, chronic actinic dermatitis, dermatitis herpetiformis, sporotrichosis and bullous disease
  Patients with mild active AD will have represented up to 10% of the atopic PN study population.
- PN secondary to medications (for example, opioids and angiotensin-converting enzyme inhibitors)
- PN secondary to medical conditions such as neuropathy or psychiatric disease (for example, notalgia paresthetica, brachioradial pruritus, neurotic excoriations, obsessive compulsive disorder and delusions of parasitosis)
- Documented moderate-to-severe AD within 6 months before the screening visit or documented diagnosis of moderate-to-severe AD from screening visit to randomization visit (for example, Investigator Global Assessment (IGA) for AD of 3 or 4, Eczema Area and Severity Index (EASI) ≥16 and SCORing Atopic Dermatitis (SCORAD) ≥25)
- Severe concomitant illness(es) under poor control that, in the investigator’s judgment, would adversely affect the patient’s participation in the study
  Examples include, but are not limited to, patients with life expectancy shorter than 1 year; patients with uncontrolled diabetes (hemoglobin A1c ≥9% according to the laboratory results within 3 months before screening visit); patients with cardiovascular conditions (for example, class III or IV heart failure according to the New York Heart Association classification); hepatobiliary conditions (for example, Child–Pugh class B or C); neurologic conditions (for example, demyelinating diseases); active major autoimmune diseases (for example, lupus, inflammatory bowel disease and rheumatoid arthritis); and other severe endocrinological, gastrointestinal, metabolic, pulmonary or lymphatic diseases. The specific justification for patients excluded under this criterion would be noted in study documents (chart notes and electronic CRF (eCRF)).
- Severe renal conditions (for example, patients with uremia and/or on dialysis)
- Uncontrolled thyroid disease
- Active tuberculosis (TB) or non-tuberculous mycobacterial infection or a history of incompletely treated TB, unless it was well documented by a specialist that the patient had been adequately treated and can now start treatment with dupilumab in the medical judgment of the investigator and/or infectious disease specialist; TB testing was performed on a country-by-country basis, according to local guidelines if required by regulatory authorities or ethics boards.
- Diagnosed active endoparasitic infections; suspected or high risk of endoparasitic infection, unless clinical and (if necessary) laboratory assessments have ruled out active infection before randomization
- Active chronic or acute infection (except HIV infection) requiring treatment with systemic antibiotics, antivirals, antiprotozoals or antifungals within 2 weeks before screening visit or during the screening period
- Known or suspected immunodeficiency, including history of invasive opportunistic infections (for example, TB, histoplasmosis, listeriosis, coccidioidomycosis, pneumocystosis and aspergillosis) despite infection resolution or otherwise recurrent infections of abnormal frequency or prolonged duration suggesting an immune-compromised status, as judged by the investigator
- Active malignancy or history of malignancy within 5 years before the baseline visit, except completely treated in situ carcinoma of the cervix, completely treated and resolved non-metastatic squamous or basal cell carcinoma of the skin
- History of systemic hypersensitivity or anaphylaxis to any biologic therapy, including any excipients
- Any other medical or psychological condition, including relevant laboratory abnormalities at screening, that, in the opinion of the investigator, suggested a new and/or insufficiently understood disease, may have presented an unreasonable risk to the study patient as a result of his/her participation in this clinical trial, may have made the patient’s participation unreliable or may have interfered with study assessments. The specific justification for patients excluded under this criterion was to be noted in study documents (chart notes and eCRF).
- History of substance and/or alcohol abuse
- Planned major surgical procedure during the patient’s participation in this study
- Exposure to another systemic or topical investigative drug (monoclonal antibodies as well as small molecules) within a certain time period before visit 1 (screening), as follows: an interval of less than 6 months or <5 pharmacokinetic (PK) half-lives for investigative monoclonal antibodies, whichever was longer, and an interval of fewer than 30 days or <5 PK half-lives, whichever was longer, for investigative small molecules
- Had used any of the following treatments within 4 weeks before the screening visit:
  Systemic immunosuppressive/immunomodulating drugs (for example, systemic corticosteroids, cyclosporine, mycophenolate-mofetil, interferon-gamma, Janus kinase inhibitors, azathioprine, methotrexate, hydroxychloroquine, dapsone, sulfasalazine and colchicine)
  Intralesional corticosteroid injections and cryotherapy
  Phototherapy, including tanning beds
  Naltrexone or other opioid antagonists
  Gabapentin, pregabalin or thalidomide
- Started to use the following treatments or changed the dose of the following treatments in 3 months before the screening visit or expected the dose of the following treatments would be changed throughout the study:
  Paroxetine, fluvoxamine or other selective serotonin reuptake inhibitors (SSRIs)
  Serotonin and norepinephrine reuptake inhibitors (SNRIs)
  Amitriptyline or other tricyclic or tetracyclic antidepressants
- Previous treatment with biologic medicines within the following timeframe:
  Any cell-depleting agents including, but not limited to, rituximab: within 6 months before the screening visit
  Omalizumab: within 5 months before screening visit
  Other immunomodulatory biologics: within 5 half-lives (if known) or 16 weeks before the screening visit, whichever was longer
- Initiation of treatment with prescription moisturizers or moisturizers containing additives such as ceramide, hyaluronic acid, urea, menthol, polidocanol or filaggrin degradation products during the screening period (patients could continue using stable doses of such moisturizers if initiated before the screening visit)
- Initiation of treatment with TCS/TCI (any potency) during the screening period or treatment with high-potency or super-potent TCS/TCI during the screening period
- For patients who were on a stable regimen of TCS/TCI (maintain same medicine, same dose from 2 weeks before screening visit) at the screening visit:
  Application of TCS/TCI on fewer than 6 days during the 7 days immediately preceding randomization
  Application of TCS/TCI of incorrect potency within 7 days before day 1 (correct application was low potency if on low potency at screening visit and medium potency if on medium or higher potency at screening visit)
- Treatment with a live (attenuated) vaccine within 4 weeks before the screening visit
  For patients who had vaccination with live, attenuated vaccines planned during the study (based on national vaccination schedule/local guidelines), it was determined, after consultation with a physician, whether the administration of vaccine could be postponed until after the end of study or preponed to before the start of the study, without compromising the health of the patient.
  - Patients for whom administration of live (attenuated) vaccine could be safely postponed were eligible to enroll in the study.
  - Patients who had their vaccination preponed could enroll in the study only after a gap of 4 weeks after administration of the vaccine.
- Planned or anticipated use of any prohibited medications and procedures during screening and study treatment period
- Participation in a prior dupilumab clinical study, treated in the past with dupilumab or prior use of biologics for PN
- For patients without history of HIV infection before screening visit, positive HIV serology at screening; for patients with history of HIV infection, CD4^+^ counts ≤300 cells per microliter and/or detectable HIV viral load at screening
- Patients with any of the following results at screening:
  Positive (or indeterminate) HBs Ag
  Positive total HBc Ab confirmed by positive HBV DNA
  Positive HCV Ab confirmed by positive HCV RNA

### Treatment and procedures

Patients were randomized 1:1 to receive subcutaneous dupilumab 300 mg (loading dose of 600 mg on day 1) or matching placebo every 2 weeks for 24 weeks. Patients on a stable regimen of low-to-moderate potency TCS and TCI before screening were allowed to continue their use throughout the trial. Patients on stable doses of antidepressants for 3 months before enrollment were eligible, provided they planned to keep medication unchanged throughout.

### Prohibited concomitant medications and procedures

The concomitant use of the following therapies was prohibited during the entire study. Study treatment was to be discontinued in patients receiving these treatments*:

- Systemic immunosuppressive/immunomodulating drugs (for example, systemic corticosteroids, cyclosporine, mycophenolate mofetil, interferon-gamma, Janus kinase inhibitors, azathioprine, methotrexate, hydroxychloroquine, dapsone, sulfasalazine and colchicine)
- Other monoclonal antibodies (that are biological modifiers)
- Phototherapy, including tanning beds
- Naltrexone or other opioid antagonists
- Gabapentin, pregabalin, and thalidomide

*Although kappa-agonists were not specifically mentioned in the protocol, no patients in PRIME or PRIME2 used kappa-agonists.

The concomitant use of the following therapies was prohibited except if the dose had been stable for at least 3 months before screening, but study treatment was not to be discontinued in patients receiving the treatments listed below. The doses should have remained stable (could be reduced or discontinued if medically indicated) but were not to be initiated or increased throughout the study.

- Paroxetine, fluvoxamine or other SSRIs
- SNRIs
- Amitriptyline or other tricyclic or tetracyclic antidepressants

The concomitant use of the following therapies was also prohibited during the entire study, but study treatment did not need to be discontinued in patients receiving:

- Intralesional corticosteroid injections and cryotherapy
- Sedating antihistamine
- Non-sedating antihistamine used specifically for the treatment of itch secondary to AD or PN

### Rescue treatments

If medically necessary (that is, to control intolerable PN symptoms), rescue treatment for PN was provided to study patients at the discretion of the investigator. Although the use of rescue medications was allowed at any time during the study, the use of such medications was delayed, if possible, for at least 14 days after the initiation of the investigational treatment. The date and time of rescue medication administration, as well as the name and dosage regimen of the rescue medication, was recorded in the eCRF. For the efficacy responder analysis, a pre-specified algorithm was used to classify rescue. In addition, a blinded review of all post-baseline medications, based on medical judgment, was performed to adjudicate rescue treatment. Patients who received rescue treatment as per this adjudication during the study were considered as having treatment failure.

Dermatological preparations of high-potency or super-potent TCS and TCI could be used as rescue medications.

### Randomization

Randomization was performed centrally using a permuted block randomization schedule via interactive voice response system/interactive web response system and was stratified by documented history of atopy (atopic or non-atopic), stable use of TCS/TCI (yes or no) and country/territory code. Atopy was defined as having a medical history of AD, allergic rhinitis/rhinoconjunctivitis, asthma or food allergy. Atopic and non-atopic PN populations were to be capped at 60% of the total enrolled population. Of the atopic population enrolled, mild, active AD was capped at 10%.

### Endpoints

Instruments used to measure efficacy endpoints are described in Supplementary Table 2. The original primary endpoint in both trials was improvement (reduction) in WI-NRS by ≥4 points from baseline to week 12. Results from PRIME2, which preceded PRIME, showed that the effect of dupilumab over time continually improved after week 12 across all endpoints, with a similar timecourse of improvement in both itch and lesion endpoints through week 24. Based on these data, before the PRIME database lock, the timing for the primary efficacy endpoint in PRIME was moved from week 12 to week 24 in the protocol amendment 03 (Supplementary Data 1) to represent the overall treatment effect more accurately and to synchronize the primary itch assessment with the primary lesion assessment.

#### Primary endpoints

- Proportion of patients with improvement (reduction) in WI-NRS by ≥4 points from baseline
  to week 24 (PRIME)
  to week 12 (PRIME2)

#### Key secondary endpoints

- Proportion of patients with improvement (reduction) in WI-NRS by ≥4 points from baseline to week 24 (PRIME2 only)
- Proportion of patients with reduction in skin lesion number to an IGA PN-S score of 0 or 1 at week 24 (PRIME and PRIME2)
- Proportion of patients concomitantly achieving a ≥4-point reduction in WI-NRS with an IGA PN-S of 0 or 1 at week 24 (PRIME and PRIME2, United States and United States-reference countries only)

#### Other multiplicity-controlled endpoints

- Percent change from baseline in WI-NRS at week 24 (PRIME and PRIME2)
- Proportion of patients with IGA PN-S 0 or 1 at week 12 (only multiplicity-controlled in PRIME2, secondary non-multiplicity-controlled endpoint in PRIME)
- Change from baseline in health-related QoL as measured by DLQI at week 24 (PRIME and PRIME2)
- Change from baseline in Skin Pain NRS at week 24 (PRIME and PRIME2)
- Change from baseline in HADS total score at week 24 (PRIME and PRIME2)
- Change from baseline in Sleep NRS at week 24 (only multiplicity-controlled in PRIME2, secondary non-multiplicity-controlled endpoint in PRIME)

#### Supportive secondary endpoints

- Proportion of patients with WI-NRS reduction ≥4 over time until week 24
- Proportion of patients with WI-NRS reduction ≥4 at week 4
- Proportion of patients with IGA PN-S 0 or 1 score at week 12
- Proportion of patients with IGA PN-S 0 or 1 score at week 8
- Proportion of patients with IGA PN-S 0 or 1 score at week 4
- Proportion of patients with IGA PN-A (PN activity) 0 or 1 score at week 24
- Proportion of patients with IGA PN-A 0 or 1 score at week 12
- Proportion of patients with IGA PN-A 0 or 1 score at week 8
- Proportion of patients with IGA PN-A 0 or 1 score at week 4
- Time to onset of effect on pruritus as measured by proportion of patients with an improvement (reduction) in WI-NRS by ≥4 from baseline during the 24-week treatment period
- Change from baseline in WI-NRS at week 24
- Change from baseline in WI-NRS at week 12
- Percent change from baseline in WI-NRS at week 12
- Percent change from baseline in WI-NRS at week 4
- Percent change from baseline in WI-NRS at week 2
- Percent change from baseline in WI-NRS over time until week 24
- Onset of action in change from baseline in WI-NRS (first *P* < 0.05 difference from placebo in the daily WI-NRS that remains significant at subsequent measurements) until week 12
- Change from baseline in IGA PN-S score at week 24
- Change from baseline in IGA PN-S score at week 12
- Change from baseline in IGA PN-S score at week 8
- Change from baseline in IGA PN-S score at week 4
- Change from baseline in health-related QoL, as measured by DLQI to week 12

#### Tertiary endpoints

- Use of high-potency or super-potent TCS rescue medication through week 24
- Use of systemic immunosuppressant through week 24, constituting treatment failure
- Change from baseline in HADS total score to week 24
- Change from baseline in EuroQoL five-dimension questionnaire, five-level version (EQ-5D5L) single index score to week 24
- Change from baseline in EQ-5D visual analog scale to week 24
- Change from baseline in Skin Pain NRS to week 4, week 8, week 12 and week 24, respectively
- Change from baseline in Sleep NRS to week 4, week 8, week 12 and week 24, respectively
- Missed school/workdays through week 24
- Incidence of skin-infection TEAEs (excluding herpetic infections) through week 24
- Proportion of patients who achieve ≥75% healed lesions from Prurigo Activity Score (PAS) at week 4, week 8, week 12 and week 24, respectively
- Change from baseline in exact number of lesions in representative area (as determined from PAS) at week 4, week 8, week 12 and week 24, respectively
- Change from baseline in Patient Global Impression of Severity (PGIS) of PN to week 4, week 8, week 12 and week 24, respectively
- Proportion of patients with PGIS score of ‘none’ at week 4, week 8, week 12 and week 24, respectively
- Proportion of patients with PGIS score of ‘none’ or ‘mild’ at week 4, week 8, week 12 and week 24, respectively
- Patient Global Impression of Change (PGIC) of PN at week 4, week 8, week 12 and week 24, respectively
- Proportion of patients with PGIC score of ‘very much better’ at week 4, week 8, week 12 and week 24, respectively

### Statistical analysis

A sample size of 150 patients for PRIME and PRIME2 each was estimated to provide 90% power to detect a 28% difference in the primary endpoint between dupilumab and placebo with a Fisher exact test at a two-sided alpha of 0.05, assuming response rates of 39% and 11%, respectively. Efficacy analyses were performed in the intention-to-treat population, which included all randomized patients analyzed according to the intervention group allocated by randomization regardless of whether the treatment kit was used or not. Safety analyses were performed on all patients randomly assigned to study intervention and who received at least one dose of study intervention. Patients were analyzed according to the intervention they received.

A study-level multiplicity procedure was used to control for the overall type I error rate for testing primary, key secondary and selected other endpoints (Extended Data Table 1). Efficacy endpoints that measured binary responses were analyzed using a Cochran–Mantel–Haenszel test adjusted by stratification factors and baseline antidepressant use. Patients with missing data at the timepoint or who used rescue/prohibited medications/procedures before the timepoint were considered non-responders. Data collected after discontinuation were included in the analysis. Continuous efficacy endpoints were analyzed using an analysis of covariance (ANCOVA) model with intervention group, stratification factors, baseline antidepressant use and relevant baseline measurement as covariates. Data from patients who used rescue/prohibited medications/procedures were set to ‘missing’ after medication use, and the endpoint value was imputed by the worst post-baseline value available before medication use. For participants who discontinued due to lack of efficacy, a worst-observation carried forward (WOCF) approach was used to impute missing data if needed. For participants who discontinued due to other reasons or any other type of missing data, a multiple imputation (MI) approach was used to impute missing data. All data collected after treatment discontinuation were used in the analysis. Safety analyses were descriptive. All analyses were conducted using SAS software version 9.4.

### Sensitivity and supplementary analysis

For the primary estimand for the primary endpoint, and for the key secondary endpoints, sensitivity/supplementary analyses were performed in both PRIME and PRIME2.

#### As-observed analysis (included all data after taking selected prohibited and/or rescue medications)

Data collected after taking all prohibited medications and/or rescue medications were included in the supplementary analysis to evaluate the robustness of the primary analysis results with respect to the method of handling data while taking selected prohibited/rescue medications (for example, treatment policy strategy). In addition, for patients discontinuing the study treatment before week 12 (or 24), their off-study treatment values measured up to week 12 (or 24) were included in the analysis. The patients having missing data at week 12 (or 24) regardless of reason(s) were considered non-responders at that timepoint (Extended Data Table 2).

#### Hybrid method analysis (WOCF and MI)

In the primary analysis of change from baseline in WI-NRS (continuous variable) at week 12 (or 24), the hybrid method of the WOCF and MI was used. Similar to the continuous variable, the same imputation method was used in the analysis of the proportion of patients with improvement (reduction) in WI-NRS by ≥4 from baseline to week 12 (or 24), which is consistent for the intercurrent event strategy and missing data handling in the binary variables and continuous variable. That is, after the imputation of continuous WI-NRS data at week 12 (or 24) using the hybrid method of the WOCF and MI, responders were defined as patients with improvement in WI-NRS by ≥4 from baseline to week 12 (or 24) in each of the imputed datasets with about 40 imputations, and then the Cochran–Mantel–Haenszel test adjusted by documented history of atopy (atopic or non-atopic), stable use of TCS/TCI (yes or no), region and baseline antidepressant use (yes or no) was used. Statistical inference obtained from all imputed data was combined using Rubin’s rule (Extended Data Table 2).

### Reporting summary

Further information on research design is available in the Nature Portfolio Reporting Summary linked to this article.

## Online content

Any methods, additional references, Nature Portfolio reporting summaries, source data, extended data, supplementary information, acknowledgements, peer review information; details of author contributions and competing interests; and statements of data and code availability are available at 10.1038/s41591-023-02320-9.

### Supplementary information

Supplementary Information PRIME and PRIME2 study investigator list and Supplementary Tables 1–8.

Reporting Summary

## Figures and Tables

**Extended Data Table 1:**
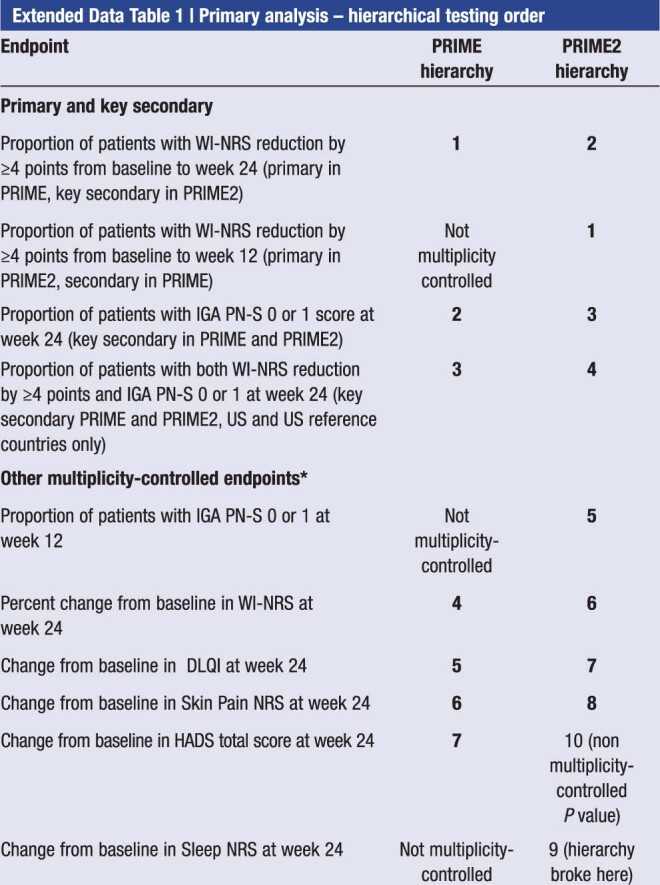
Primary analysis—hierarchical testing order

*Refers to endpoints that were multiplicity-controlled in at least one of the two trials. Testing was done in numerical order for endpoints 1–7 in PRIME and 1–10 in PRIME2; endpoints showed in bold represent statistically significant differences from placebo. In PRIME2, the hierarchy broke at the ninth tested endpoint, and, therefore, the *P* value for the tenth endpoint is non-multiplicity-controlled.

**Extended Data Table 2:**
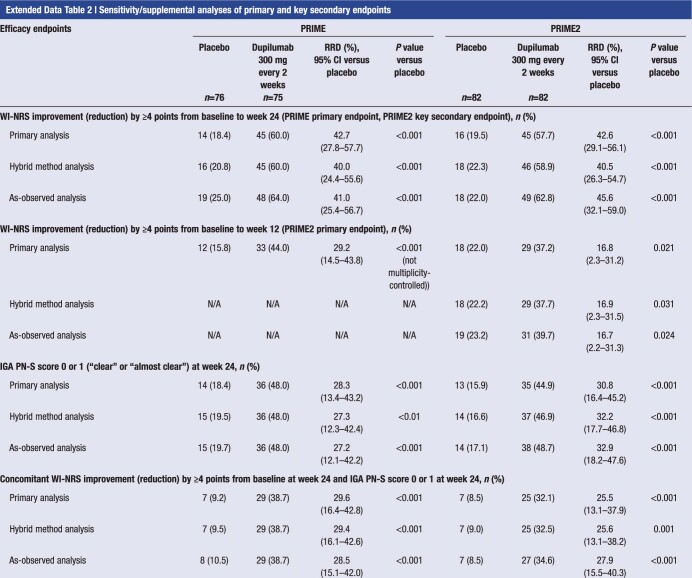
Sensitivity/supplemental analyses of primary and key secondary endpoints

*P* values were derived by a Cochran–Mantel–Haenszel test performed on the association between the responder status and intervention group, adjusted by documented history of atopy (atopic or non-atopic), stable use of TCS/TCI (yes or no), region and baseline antidepressant use (yes or no). RRD was derived from the Mantel–Haenszel estimator. For the primary analysis, patients who received prohibited medications/procedures and/or rescue medications that impacted efficacy before week 12/24, as well as those who had missing data at week 12/24, were considered non-responders. For the hybrid method analysis, data collected after study intervention discontinuation were included. Data after the selected prohibited medications/procedures and/or rescue medications that impacted efficacy were set to missing and imputed by WOCF. Missing data at week 12/24 after study intervention discontinuation for lack of efficacy were imputed by WOCF; other missing data at week 12/24 were imputed by multiple imputation. For the as-observed analysis, data collected after study intervention discontinuation and/or after taking the selected prohibited medications/procedures and/or rescue medications were included, and missing data at week 12/24 were considered non-responders. N/A, not available; RRD, response rate difference.

**Extended Data Table 3:**
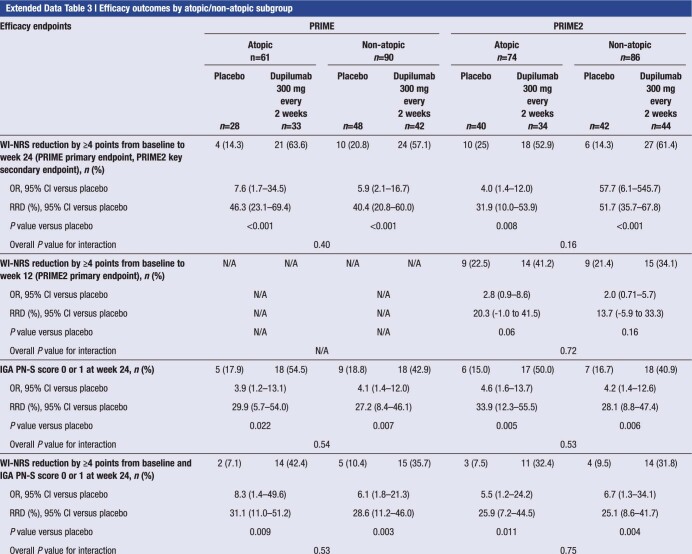
Efficacy outcomes by atopic or non-atopic subgroup

*P* values versus placebo (non-multiplicity-controlled) were obtained by a Cochran–Mantel–Haenszel test performed on the association between the responder status and intervention group, adjusted by documented history of atopy (atopic or non-atopic), stable use of TCS/TCI (yes or no), region and baseline antidepressant use (yes or no). Overall *P* values for interaction were calculated by a logistic regression model used for the interaction test including intervention group, adjusted documented history of atopy (atopic or non-atopic), stable use of TCS/TCI (yes or no), region and baseline antidepressant use (yes or no) plus the subgroup variable and the subgroup-by-intervention in the model. OR and RRD were derived from the Mantel–Haenszel estimator. Patients who received the prohibited medications/procedures and/or rescue medications that impacted efficacy before week 12/24, as well as those with missing data at week 12/24, were considered non-responders. N/A, not available; OR, odds ratio; RRD, response rate difference.

**Extended Data Table 4:**
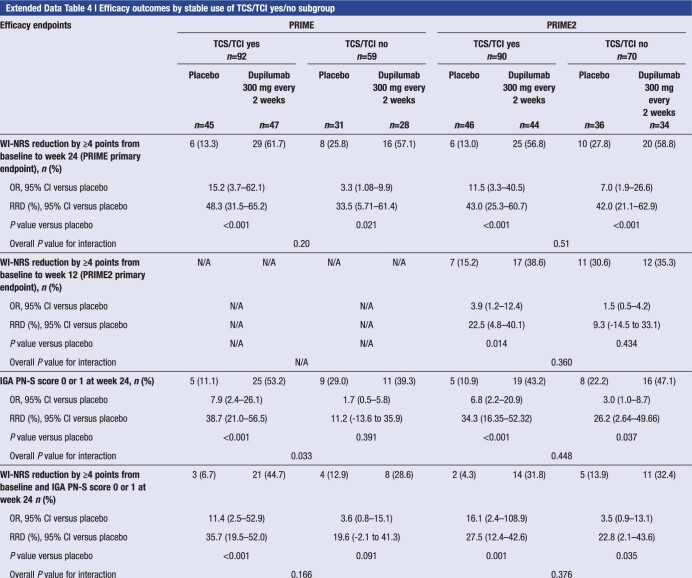
Efficacy outcomes by stable use of TCS/TCI yes or no subgroup

*P* values versus placebo (non-multiplicity-controlled) were obtained by a Cochran–Mantel–Haenszel test performed on the association between the responder status and intervention group, adjusted by documented history of atopy (atopic or non-atopic), stable use of TCS/TCI (yes or no), region and baseline antidepressant use (yes or no). Overall *P* values for interaction were calculated by a logistic regression model used for the interaction test including intervention group, adjusted documented history of atopy (atopic or non-atopic), stable use of TCS/TCI (yes or no), region and baseline antidepressant use (yes or no) plus the subgroup variable and the subgroup-by-intervention in the model. OR and RRD were derived from the Mantel–Haenszel estimator. Patients who received the prohibited medications/procedures and/or rescue medications that impacted efficacy before week 12/24, as well as those with missing data at week 12/24, were considered non-responders. N/A, not available; OR, odds ratio; RRD, response rate difference.

**Extended Data Table 5:**
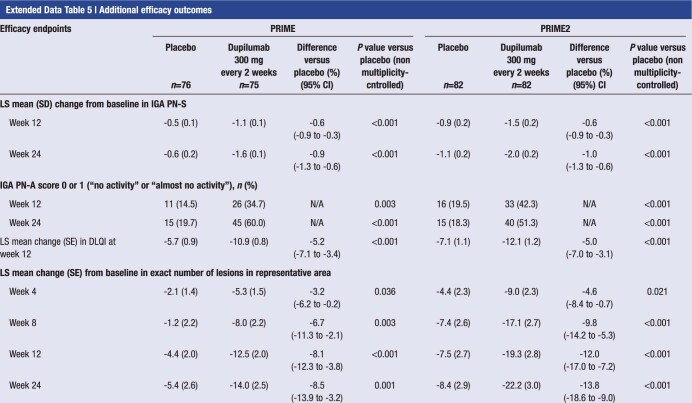
Additional efficacy outcomes

For endpoints that measured binary responses, *P* values were derived by a Cochran–Mantel–Haenszel test performed on the association between the responder status and the intervention group, adjusted by stratification factors and baseline antidepressant use (yes or no). Stratification factors were documented history of atopy (yes or no), stable TCS/TCI use (yes or no) and region. The difference versus placebo is the response rate difference derived from the Mantel–Haenszel estimator. Patients who received prohibited medications/procedures and/or rescue medications that impacted efficacy before week 12/24, as well as those with missing data at week 12/24, were considered non-responders. For continuous efficacy endpoints, *P* values and difference versus placebo were derived for each of the imputed complete data by fitting an ANCOVA model with the corresponding baseline value, intervention group, stratification factors and baseline antidepressant use as covariates. Data collected after study intervention discontinuation were included. Data after the selected prohibited medications/procedures and/or rescue medications that impacted efficacy were set to missing and imputed by WOCF. Missing data after study intervention discontinuation for lack of efficacy were imputed by WOCF, and other missing data were imputed by multiple imputation.

## Data Availability

Scientific and medical researchers may request access to study documents (including the clinical study report, study protocol with any amendments, blank case report form and statistical analysis plan) that support the methods and findings reported in this manuscript. Individual anonymized participant data will be considered for sharing once the product and indication have been approved by major health authorities (for example, the US Food and Drug Administration, the European Medicines Agency and the Pharmaceuticals and Medical Devices Agency), if there is legal authority to share the data, and there is not a reasonable likelihood of participant re-identification. Requests should be submitted to https://vivli.org/ and will be addressed within 60 days.
