# Supplementary material for: Dupilumab in patients with prurigo nodularis: two randomized, double-blind, placebo-controlled phase 3 trials
Source: Nat Med. 2023 May 4;29(5):1180–90. doi: 10.1038/s41591-023-02320-9 (PMC10202800; doi:10.1038/s41591-023-02320-9)
Supplement: Supplementary file 1 — PRIME and PRIME2 study investigator list and Supplementary Tables 1–8. [file 41591_2023_2320_MOESM1_ESM.pdf]

nature medicine

Article

https://doi.org/10.1038/s41591-023-02320-9

Dupilumab in patients with prurigo nodularis: two randomized, double-blind, placebo-controlled phase 3 trials

In the format provided by the authors and unedited

Supplementary Appendix

Table of Contents

Study Investigators	2
PRIME	2
PRIME2	3
Supplementary Table 1. Representativeness of the trial population	6
Supplementary Table 2. Instruments used to measure efficacy endpoints	8
Supplementary Table 3. Comorbid conditions associated with PN in patients enrolled in PRIME and PRIME2	11
Supplementary Table 4. WI-NRS daily score reporting in the week preceding the primary endpoint assessment	16
Supplementary Table 5. Summary of patients with WI-NRS and IGA PN-S missing data	17
Supplementary Table 6. Serious adverse events (MedDRA SOC and PT)	19
Supplementary Table 7. Adverse events leading to discontinuation by primary SOC and PT	21
Supplementary Table 8. Overview of skin infections (except herpes infections) by MedDRA SOC and PT	22
Supplementary Data 1 – Trial protocols and statistical analysis plans	24
Supplementary Data 2 – Institutional Review Boards/Independent Ethics Committees	708

Study investigators

Listed in alphabetical order by country/institution.

PRIME

ARGENTINA: Buenos Aires Skin (Pablo González, Matias Turienzo); Centro de Investigación Clínica CEDIC (Nora Kogan); Centro Medico Privado de Reumatologia (Cecilia Maria Noelia); Diagnostico Urquiza (Pablo González); Fundación Cidea (Jorge Maspero); Instituto de Neumologia y Dermatologia (Paula Luna); Investigaciones en Alergia y Enfermedades Respiratorias (Anahi Yañez); Parra Dermatología (Viviana Parra); STAT Research S.A. (Mariano Marini).

CHINA: Beijing Friendship Hospital, Capital Medical University (Linfeng Li); Hangzhou First People's Hospital (Liming Wu); West China Hospital, Sichuan University (Jingyi Li); Wuxi No. 2 People's Hospital (Xiaohong Zhu).

FRANCE: Polyclinique de Courlancy (Ziad Reguiai).

JAPAN: Fujita Health University Bantane Hospital (Akiko Yagami); Kyoto University Hospital (Saeko Nakajima); Nagasaki University Hospital (Motoi Takenaka); National Defense Medical College Hospital (Takahiro Satoh); Nihon University Itabashi Hospital (Hideki Fujita); NTT Medical Center Tokyo (Atsuyuki Igarashi); Shimane University Hospital (Yuko Chinuki); Tokyo Medical and Dental University, Medical Hospital (Takeshi Namiki, Hiroo Yokozeki); Yokohama City University Hospital (Naoko Inomata, Yuko Watanabe).

KOREA, REPUBLIC OF: Gachon University Gil Medical Center (Joo-Young Roh); Hallym University Medical Center (Chun Wook Park); Seoul National University Bundang Hospital (Jung-Im Na); Seoul National University Hospital (Kiu Han Kim); The Catholic University of Korea, Incheon St. Mary's Hospital (Sang Hyun Cho); Yonsei University Health System Severance Hospital (Chang Ook Park).

MEXICO: Arké SMO SA de CV (Claudia Bernabé del Río); Consultorio Médico (Raul Aguilar Orozco); Eukarya PharmaSite S.C. (Jorge Garza Gómez); Hospital Universitario Dr. José Eleuterio Gozález

(Jorge de Jesús Ocampo Candiani); Instituto de Jalisciense de Investigación Clinica, SA de CV (Dante Hernandez).

RUSSIA: Chelyabinsk Regional Dermatologic Dispensary (Oleg Ziganshin); Clinical Dermatovenerogic Dispensary (Alkes Khotko); Institute of Immunology (Elena Fedenko); Russian Medical Academy of Continuous Professional Education (Alexei Kubanov); Saratov State Medical University (Andrei Bakulev); State Scientific Center of Dermatovenerology and Cosmetology (Irina Kondrakhina); Stavropol Regional Clinical Hospital (Olga Ukhanova); Pavlov First Saint Petersburg State Medical University (Evgeny Sokolovskiy).

UNITED STATES OF AMERICA: Advanced Medical Research PC (Jamie Weisman); Arizona Allergy and Immunology Research (Neal Jain); Austin Institute for Clinical Research (Edward Lain); Aventiv Research (Jess DeMaria); Bellaire Dermatology Associates (Craig Teller); Center for Clinical Studies (Stephen Tyring); Clarkston Skin Research (Wendy McFalda); Columbus Regional Research Institute (William Paull); Dawes Fretzin Dermatology Group (Scott Fretzin); Dermatology and Laser Center (Todd Schlesinger); Johnson Dermatology (Sandra Johnson); MedaPhase (Mark Ling); Oakview Dermatology (Dawn Sammons); Psoriasis Treatment Center of Central New Jersey (Jerry Bagel); Stephen Miller MD, PA (William Chad Cragun); University of California, Davis Medical Center (Emanuel Maverakis); University of Miami (Gil Yosipovitch); University of Pennsylvania (Nicholas Mollanazar); Virginia Clinical Research (David Pariser); Vital Prospects Clinical Research Institute (Iftikhar Hussain); Washington University School of Medicine (Heather Jones).

PRIME2

CANADA: AvantDerm (Davindra Singh); Dr. Chih-ho Hong Medical (Chih-ho Hong); Dr. S.K. Siddha Medicine Professional Corporation (Sanjay Siddha); Kirk Barber Research (Kirk Barber); Lynderm Research (Charles Lynde); Skinsense Medical Research (Kristen Walker).

CHILE: Centro Internacional de Estudios Clínicos CIEC (Fernando Valenzuela); Centro Medico Skinmed (Walter Gubelin); Clínica Dermacross (Claudia De La Cruz); Clinical Research Chile (Álvaro San Martin); Hospital Basé de Osorno (Cristian Pizzaro); Hospital Clínico San Borja Arriarán (Javier Arellano).

FRANCE: APHP – Hôpital Saint-Louis (Jean-David Bouaziz); Centre Hospitalier du Mans (Nathalie Beneton); CHRU Brest – Hôpital Morvan (Laurent Misery); CHRU Lille – Hôpital Claude Huriez (Delphine Staumont-Salle); CHU Toulouse – Hôpital Larrey (Maria-Polina Konstantinou); HCL Lyon – Hôpital Edouard Herriot (Denis Jullien); Polyclinique de Courlancy (Ziad Reguiai); Hôpital Saint André-MIGR (Julien Senechal).

HUNGARY: Debreceni Egyetem Klinikai Központ (Andrea Szegedi); DermaMed Research Kft. (Dósa Piroska); PTE KK (Ágnes Kynió); Szegedi Tudományegyetem Börgyógyászati és Allergológiai Klinika (Kemény Lajos).

ITALY: Azienda Ospedaliero Universitaria Mater Domini (Cataldo Patruno); Azienda Ospedaliero-Universitaria Ospedale Riuniti Umberto I (Anna Maria Offidani); Istituto Clinico Humanitas (Antonio Costanzo); Ospedale Maggiore Policlinico (Silvia Ferrucci).

KOREA, REPUBLIC OF: Chung-Ang University Hospital (SeongJun Seo); Hallym University Medical Center (Chun Wook Park); Pusan National University Hospital (Byung Soo Kim); Soonchunhyang University Hospital Bucheon (YoungLip Park); The Catholic University of Korea, St. Mary's Hospital (Sang-Hyun Cho); Yongsei University Health System, Severance Hospital (Chang Ook Park).

PORTUGAL: Centro Hospitalar e Universitário de Coimbra (Margarida Gonçalo); Centro Hospitalar Universitário de Porto, EPE (Inês Lobo); CUF Descobertas (Pedro Bastos).

SPAIN: Complexo Hospitalario de Pontevedra (Ángeles Flórez Menéndez); Hospital Vital Álvarez Buylla (Pablo Coto Segura); Hospital Universitari Germans Trias I Pujol (Jose Manuel Carrascosa Carrillo); Hospital Universitario Miguel Servet (Yolanda Gilaberte Calzada, Maria Milagros Sanchez

Hernandez); Hospital Universitari i Politècnic La Fe (Rafael Botella Estrada); Hospital Universitario Ramón y Cajal (Maria Bibiana Pérez Garcia); Hospital Universitario Reina Sofía (Juan Alberto Ruano Ruiz).

TAIWAN: Chang Gung Memorial Hospital Kaoshiung (Chih-Hung Lee); Chung Shan Medical University Hospital (Po-Ju Lai); Mackay Memorial Hospital (Nan-Lin Wu); National Taiwan University Hospital (Hsien-Yi Chiu, Tsen-Fang Tsai).

UNITED KINGDOM: Surrey and Sussex Healthcare NHS Trust (Sandeep Cliff).

UNITED STATES OF AMERICA: Acclaim Dermatology, PLLC (Syed Omar Ali); Johns Hopkins University, School of Medicine (Shawn Kwatra); Johnson Dermatology (Sandra Johnson); Psoriasis Treatment Center of Central New Jersey (Jerry Bagel); Riverchase Dermatology and Cosmetic Surgery (Robert Snyder); The Indiana Clinical Trials Center, PC (Scott Guenthner); University of California, Davis Medical Center (Emanual Maverakis).

Supplementary Table 1 | Representativeness of the trial population

**Category**	
Disease, problem or condition under investigation	PN uncontrolled by topical therapies or in patients in whom topical therapies are not medically advisable.
Prevalence	Annual prevalence of PN was estimated to date at 32.7/100,000 in the UK[3], 40/100,000 in Germany[4], 6.52/100,000 population in Poland[5], and 4.82 per 1,000 dermatology outpatients in South Korea[6]; in the USA, it was estimated at 18–70 per 100,000[7] and in another study, at up to 72 per 100,000[8].
Special considerations related to	
Sex	In the USA, PN was diagnosed more frequently in women (54.6% in African American and 50.5% in White patients)[17]; in Asian patients, males with PN were predominant (58.1% in Asian American patients[17], 58.1% in Korean patients[6]).
Age	PN is predominantly observed in older patients with a median age of over 50 years[11,17,35], and occasionally in younger patients where an atopic diathesis was found to predict an earlier age of onset compared with non-atopic patients[16].
Race or ethnic group	In a US study, African American patients were 3.4 times more likely to have PN than White patients[17].
Geography	Data not available.
Other considerations	PN is associated with increased rates of obesity, mental health problems, endocrine, cardiovascular and renal disorders, HIV and malignancy[1,8,13]; approximately 20–60% of patients have either past or current history of AD or other atopic disorders[11,16,35].

**Category**	
Overall representativeness of this trial	In our studies, female sex was predominant with 66.2% of patients in PRIME and 64.4% in PRIME2. Approximately two-thirds of PRIME/PRIME2 patients were Black or White (in whom PN was found to predominate in females), while the Asian population accounted for approximately one-third (where PN was found to predominate in males). Thus, the gender distribution is largely consistent with that reported in the literature.<br>The overall median age of patients in PRIME was 51 years and in PRIME2 was 50 years, consistent with the literature.<br>PRIME enrolled 53% White patients and 7.3% Black or African American patients, and PRIME2 enrolled 60% and 5%, respectively. However, the Black or African American patients were enrolled predominantly from the USA and represented 31.4% (PRIME) and 55.6% (PRIME2) of US-enrolled patients. In both trials, this is higher than the incidence of the Black population in the USA (12.4% [United States Census Bureau 2021]), largely in line with the literature regarding the disproportionate impact in Black patients.<br>40.4% in PRIME and 46.3% in PRIME2 had a history of atopy, consistent with the literature.

Supplementary Table 2 | Instruments used to measure efficacy endpoints

Efficacy endpoint	Range	Description	Severity stratification	Minimum clinically important difference
Worst Itch Numerical Rating Scale (WI-NRS)[31, 32]	0–10	WI-NRS is a patient-reported outcome comprising 1 item, the worst itch over the past 24 hours, rated from 0 (“no itch”) to 10 (“worst imaginable itch”); weekly average WI-NRS, calculated as the average of non-missing WI-NRS scores per trial week, was used for analysis.	0 = no itch<br>1–2 = mild itch<br>3–6 = moderate itch<br>7–9 = severe itch<br>10 = very severe itch	In PN, the MCID reduction in WI-NRS appears to depend on the baseline score; for baseline WI-NRS scores between 7 and 9, reduction by ≥4 is clinically meaningful[33]
Investigator’s Global Assessment for PN Stage (IGA PN-S)[34]	0–4	IGA PN-S is a clinician-reported outcome that assesses the disease stage based on the number of pruriginous lesions:<br>0 = no nodules<br>1 = 1–5 nodules<br>2 = 6–19 nodules<br>3 = 20–99 nodules<br>4 = ≥100 nodules	0 = clear<br>1 = almost clear<br>2 = mild<br>3 = moderate<br>4 = severe	Not applicable

Investigator's Global Assessment for PN Activity (IGA PN-A)[34]	0–4	IGA PN-A is a clinician-reported outcome that assesses the disease activity based on the percentage of pruriginous lesions that have excoriations or scars:<br>0 = none of the lesions have excoriations or scars<br>1 = ≤10% of lesions have excoriations or scars<br>2 = 11–25% of lesions have excoriations or scars<br>3 = 26–75% of lesions have excoriations or scars<br>4 = ≥76% of lesions have excoriations or scars	0 = clear<br>1 = almost clear<br>2 = mild<br>3 = moderate<br>4 = severe	Not applicable
Dermatology Life Quality Index (DLQI)[36]	0–30	DLQI is a 10-item questionnaire that assesses impact of symptoms on the health-related quality of life over the past week.<br>DLQI covers symptoms, how skin disease affected daily activities, leisure activities, working and social life, personal relationships and potential impact of topical treatment.<br>All items are scored on a 4-point Likert scale:	Effect on patient's life[37]:<br>0–1 = no effect<br>2–5 = small effect<br>6–10 = moderate effect<br>11–20 = very large effect<br>21–30 = extremely large effect	Not established in PN patients

		• 9/10 items: from 0 = "not at all" to 3 = "very much"<br>• Work item (if "Yes"): 0 = "not at all", 3 = "a lot"		
Hospital Anxiety and Depression Scale (HADS)[38,39]	0–42	HADS is a patient-reported outcome for assessing anxiety and depression in non-psychiatric patients; the HADS consists of 14 items, 7 each for anxiety and depression symptoms, and scores range from 0 to 21 for each symptom subscale	For both subscale scores:<br>≤7 = normal<br>8–10 = borderline (possible case)<br>≥11 = probable case (increasing severity)	Not established in PN patients
Sleep Numeric Rating Scale (Sleep NRS)	0–10	Sleep NRS is a patient-reported outcome to rate sleep quality in the past 24 hours, from 0 ("worst possible sleep") to 10 ("best possible sleep").	Not established	Not established in PN patients
Skin Pain Numeric Rating Scale (Skin Pain NRS)	0–10	Skin Pain NRS is a patient-reported outcome to rate the worst skin pain the past 24 hours, from 0 ("no pain") to 10 ("worst imaginable pain")	Not established	Not established in PN patients

Supplementary Table 3 | Comorbid conditions associated with PN in patients enrolled in PRIME and PRIME2

	PRIME		PRIME2	
**Primary SOC**<br>**HLT, *n* (%)**	**Placebo**<br>***n*=75**	**Dupilumab 300 mg every 2 weeks**<br>***n*=75**	**Placebo**<br>***n*=82**	**Dupilumab 300 mg every 2 weeks**<br>***n*=77**
Any medical/surgical history for PN comorbidity	40 (52.6)	48 (64.0)	48 (58.5)	41 (52.6)
Infections and infestations	1 (1.3)	0	3 (3.7)	1 (1.3)
Hepatitis virus infections	1 (1.3)	0	2 (2.4)	0
Helicobacter infections	0	0	0	1 (1.3)
Retroviral infections	0	0	1 (1.2)	0
Neoplasms benign, malignant and unspecified (including cysts and polyps)	2 (2.6)	1 (1.3)	1 (1.2)	0
Breast and nipple neoplasms malignant	1 (1.3)	0	0	0
Cervix neoplasm malignant	0	1 (1.3)	0	0
Mantle cell lymphomas	0	0	1 (1.2)	0
Respiratory tract and pleural neoplasms malignant cell type unspecified NEC	1 (1.3)	0	0	0
Blood and lymphatic system disorders	6 (7.9)	4 (5.3)	1 (1.2)	3 (3.8)
Anemia deficiencies	4 (5.3)	1 (1.3)	0	0
Anemias NEC	1 (1.3)	3 (4.0)	1 (1.2)	3 (3.8)
Polycythemia (excl. rubra vera)	1 (1.3)	0	0	0
Immune system disorders	3 (3.9)	2 (2.7)	3 (3.7)	2 (2.6)
Atopic disorders	2 (2.6)	1 (1.3)	2 (2.4)	1 (1.3)

	PRIME		PRIME2	
**Primary SOC**<br>**HLT, *n* (%)**	**Placebo**<br>***n*=75**	**Dupilumab 300 mg every 2 weeks**<br>***n*=75**	**Placebo**<br>***n*=82**	**Dupilumab 300 mg every 2 weeks**<br>***n*=77**
Allergic conditions NEC	1 (1.3)	1 (1.3)	1 (1.2)	1 (1.3)
Endocrine disorders	10 (13.2)	8 (10.7)	8 (9.8)	7 (9.0)
Thyroid hypofunction disorders	8 (10.5)	7 (9.3)	5 (6.1)	7 (9.0)
Acute and chronic thyroiditis	0	1 (1.3)	2 (2.4)	0
Thyroid disorders NEC	1 (1.3)	0	0	0
Thyroid hyperfunction disorders	1 (1.3)	0	1 (1.2)	1 (1.3)
Metabolism and nutrition disorders	12 (15.8)	20 (26.7)	21 (25.6)	17 (21.8)
Diabetes mellitus (incl. subtypes)	5 (6.6)	13 (17.3)	8 (9.8)	5 (6.4)
Hyperlipidemias NEC	3 (3.9)	5 (6.7)	3 (3.7)	3 (3.8)
Lipid metabolism and deposit disorders NEC	4 (5.3)	4 (5.3)	7 (8.5)	4 (5.1)
General nutritional disorders NEC	3 (3.9)	2 (2.7)	1 (1.2)	5 (6.4)
Elevated cholesterol	0	4 (5.3)	4 (4.9)	3 (3.8)
Water soluble vitamin deficiencies	0	2 (2.7)	0	0
Elevated triglycerides	0	1 (1.3)	2 (2.4)	2 (2.6)
Psychiatric disorders	10 (13.2)	12 (16.0)	13 (15.9)	11 (14.1)
Depressive disorders	4 (5.3)	5 (6.7)	7 (8.5)	6 (7.7)
Anxiety symptoms	2 (2.6)	6 (8.0)	4 (4.9)	3 (3.8)
Disturbances in initiating and maintaining sleep	3 (3.9)	3 (4.0)	0	0
Anxiety disorders NEC	2 (2.6)	0	1 (1.2)	2 (2.6)
Bipolar disorders	0	0	2 (2.4)	0

	PRIME		PRIME2	
**Primary SOC**<br>**HLT, *n* (%)**	**Placebo**<br>***n*=75**	**Dupilumab 300 mg every 2 weeks**<br>***n*=75**	**Placebo**<br>***n*=82**	**Dupilumab 300 mg every 2 weeks**<br>***n*=77**
Attention deficit and disruptive behavior disorders	1 (1.3)	1 (1.3)	0	0
Adjustment disorders	0	1 (1.3)	0	0
Eating disorders	0	0	0	2 (2.6)
Panic attacks and disorders	0	0	1 (1.2)	0
Personality disorders with eccentric behavior (cluster a)	0	1 (1.3)	0	0
Sleep disorders NEC	1 (1.3)	0	0	0
Stress disorders	0	1 (1.3)	0	0
Nervous system disorders	4 (5.3)	2 (2.7)	0	1 (1.3)
Central nervous system vascular disorders NEC	2 (2.6)	1 (1.3)	0	0
Lumbar spinal cord and nerve root disorders	1 (1.3)	1 (1.3)	0	0
Central nervous system hemorrhages and cerebrovascular accidents	1 (1.3)	0	0	1 (1.3)
Mononeuropathies	1 (1.3)	0	0	0
Cardiac disorders	6 (7.9)	8 (10.7)	2 (2.4)	4 (5.1)
Ischemic coronary artery disorders	2 (2.6)	3 (4.0)	1 (1.2)	3 (3.8)
Supraventricular arrhythmias	1 (1.3)	4 (5.3)	0	0
Myocardial disorders NEC	1 (1.3)	2 (2.7)	0	0
Coronary artery disorders NEC	2 (2.6)	0	0	2 (2.6)

	PRIME		PRIME2	
**Primary SOC**<br>**HLT, *n* (%)**	**Placebo**<br>***n*=75**	**Dupilumab 300 mg every 2 weeks**<br>***n*=75**	**Placebo**<br>***n*=82**	**Dupilumab 300 mg every 2 weeks**<br>***n*=77**
Mitral valvular disorders	1 (1.3)	1 (1.3)	0	0
Cardiac hypertensive complications	0	0	1 (1.2)	0
Aortic valvular disorders	0	1 (1.3)	0	0
Cardiac conduction disorders	0	1 (1.3)	0	0
Cardiac signs and symptoms NEC	0	1 (1.3)	0	0
Cardiomyopathies	1 (1.3)	0	0	1 (1.3)
Heart failures NEC	0	1 (1.3)	0	0
Right ventricular failures	0	1 (1.3)	0	0
Tricuspid valvular disorders	0	1 (1.3)	0	0
Ventricular arrythmias and cardiac arrest	0	1 (1.3)	0	0
Vascular disorders	20 (26.3)	22 (29.3)	22 (26.8)	10 (12.8)
Vascular hypertensive disorders NEC	20 (26.3)	22 (29.3)	22 (26.8)	10 (12.8)
Peripheral embolism and thrombosis	1 (1.3)	1 (1.3)	0	0
Aortic necrosis and vascular insufficiency	0	0	1 (1.2)	0
Phlebitis NEC	1 (1.3)	0	0	0
Respiratory, thoracic and mediastinal disorders	2 (2.6)	2 (2.6)	1 (1.2)	0
Bronchospasm and obstruction	2 (2.6)	2 (2.7)	1 (1.2)	0
Gastrointestinal disorders	1 (1.3)	0	0	0
Malabsorption syndromes	1 (1.3)	0	0	0
Hepatobiliary disorders	0	3 (4.0)	5 (6.1)	2 (2.6)
Hepatocellular damage and hepatitis NEC	0	3 (4.0)	5 (6.1)	2 (2.6)

	PRIME		PRIME2	
**Primary SOC**<br>**HLT, *n* (%)**	**Placebo**<br>***n*=75**	**Dupilumab 300 mg every 2 weeks**<br>***n*=75**	**Placebo**<br>***n*=82**	**Dupilumab 300 mg every 2 weeks**<br>***n*=77**
Skin and subcutaneous tissue disorders	6 (7.9)	9 (12.0)	11 (13.4)	8 (10.3)
Dermatitis and eczema	6 (7.9)	7 (9.3)	6 (7.3)	6 (7.7)
Pruritus NEC	0	0	2 (2.4)	3 (3.8)
Exfoliative conditions	0	1 (1.3)	0	0
Psoriatic conditions	0	1 (1.3)	0	2 (2.6)
Skin and subcutaneous conditions NEC	0	1 (1.3)	0	0
Urticarias	0	1 (1.3)	2 (2.4)	0
Skin preneoplastic conditions NEC	0	0	1 (1.2)	0
Musculoskeletal and connective tissue disorders	3 (3.9)	1 (1.3)	1 (1.2)	4 (5.1)
Metabolic bone disorders	3 (3.9)	1 (1.3)	1 (1.2)	4 (5.1)
Renal and urinary disorders	1 (1.3)	4 (5.3)	0	2 (2.6)
Renal failure and impairment	1 (1.3)	4 (5.3)	0	2 (2.6)

HLT, MedDRA High Level Term; MedDRA, Medical Dictionary for Regulatory Affairs; NEC, not elsewhere classified; SOC, MedDRA System Organ Class.

Supplementary Table 4 | WI-NRS daily score reporting in the week preceding the primary endpoint assessment

WI-NRS	PRIME, week 24			PRIME2, week 12		
	**Placebo (n=76)**	**Dupilumab 300 mg every 2 weeks (n=75)**	**Total (n=151)**	**Placebo (n=82)**	**Dupilumab 300 mg every 2 weeks (n=78)**	**Total (n=160)**
Patients expected, *n*	68	74	142	77	76	153
Patients with, *n* (%)						
< 4 measurements	15 (22.1)	3 (4.1)	18 (12.7)	5 (6.5)	3 (3.9)	8 (5.2)
4 measurements	4 (5.9)	6 (8.1)	10 (7.0)	6 (7.8)	2 (2.6)	8 (5.2)
5 measurements	5 (7.4)	13 (17.6)	18 (12.7)	10 (13.0)	11 (14.5)	21 (13.7)
6 measurements	16 (23.5)	13 (17.6)	29 (20.4)	23 (29.9)	17 (22.4)	40 (26.1)
7 measurements	28 (41.2)	39 (52.7)	67 (47.2)	33 (42.9)	43 (56.6)	76 (49.7)

WI-NRS, Worst Itch Numeric Rating Scale.

Supplementary Table 5 | Summary of patients with WI-NRS and IGA PN-S missing data

			PRIME		PRIME2	
**Visit**	**Parameter**	**Reason for missing data**	**Placebo (n=76)**	**Dupilumab 300 mg every 2 weeks (n=75)**	**Placebo (n=82)**	**Dupilumab 300 mg every 2 weeks (n=78)**
Week 12	WI-NRS		7 (9.2%)	0	6 (7.3%)	2 (2.6%)
		Discontinuation due to lack of efficacy	1	0	2	1
		Discontinuation due to AE	1	0	1	0
		Discontinuation due to other reason	3	0	3	0
		Other*	2	0	0	1
Week 24	WI-NRS		16 (21.1%)	1 (1.3%)	24 (29.3%)	3 (3.8%)
		Discontinuation due to lack of efficacy	3	0	10	1
		Discontinuation due to AE	2	0	1	0
		Discontinuation due to other reason	6	1	12	0
		Other*	5	0	1	2
	IGA PN-S		11 (14.5%)	1 (1.3%)	23 (28.0%)	3 (3.8%)
		Discontinuation due to lack of efficacy	2	0	10	1

		Discontinuation due to AE	1	0	1	0
		Discontinuation due to other reason	6	1	10	0
		Other*	2	0	2	2
	WI-NRS or IGA PN-S		17 (22.4%)	1 (1.3%)	25 (30.5%)	4 (5.1%)
		Discontinuation due to lack of efficacy	3	0	10	1
		Discontinuation due to AE	2	0	1	0
		Discontinuation due to other reason	6	1	12	0
		Other*	6	0	2	3

*Including treatment completed but with missing data, and untreated patients (one placebo patient from PRIME and one dupilumab patient from PRIME2). AE, adverse event; IGA PN-S, Investigator's Global Assessment for PN Stage; WI-NRS, Worst Itch Numeric Rating Scale.

Supplementary Table 6 | Serious adverse events (MedDRA SOC and PT)

	PRIME		PRIME2	
**Primary SOC**<br>**PT *n* (%)***	**Placebo**<br>***n*=75**	**Dupilumab 300 mg every 2 weeks**<br>***n*=75**	**Placebo**<br>***n*=82**	**Dupilumab 300 mg every 2 weeks**<br>***n*=77**
Any event	6 (8.0)	5 (6.7)	2 (2.4)	2 (2.6)
Infections and infestations	2 (2.7)	1 (1.3)	0	1 (1.3)
COVID-19 pneumonia	0	1 (1.3)	0	0
COVID-19	1 (1.3)	0	0	0
Sepsis	1 (1.3)	0	0	0
Pelvic inflammatory disease	0	0	0	1 (1.3)
Pyelonephritis acute	0	0	0	1 (1.3)
Neoplasms benign, malignant and unspecified (including cysts and polyps)	1 (1.3)	1 (1.3)	1 (1.2)	2 (2.6)
Papillary thyroid cancer	0	1 (1.3)	0	0
Hodgkin's disease	1 (1.3)	0	0	0
Lipoma[†]	0	0	0	1 (1.3)
Uterine leiomyoma	0	0	0	1 (1.3)
Cutaneous T-cell lymphoma	0	0	1 (1.2)	0
Cardiac disorders	1 (1.3)	0	0	0
Acute myocardial infarction	1 (1.3)	0	0	0
Respiratory, thoracic, and mediastinal disorders	0	2 (2.7)	0	0
Asthma	0	1 (1.3)	0	0
Interstitial lung disease	0	1 (1.3)	0	0

	PRIME		PRIME2	
**Primary SOC**<br>**PT *n* (%)***	**Placebo**<br>***n*=75**	**Dupilumab 300 mg every 2 weeks**<br>***n*=75**	**Placebo**<br>***n*=82**	**Dupilumab 300 mg every 2 weeks**<br>***n*=77**
Gastrointestinal disorders	1 (1.3)	0	0	0
Mesenteritis	1 (1.3)	0	0	0
Skin and subcutaneous tissue disorders	1 (1.3)	0	0	0
Neurodermatitis	1 (1.3)	0	0	0
Musculoskeletal and connective tissue disorder	0	1 (1.3)	1 (1.2)	0
Musculoskeletal chest pain	0	1 (1.3)	0	0
Rotator cuff syndrome	0	0	1 (1.2)	0
Injury, poisoning, and procedural complications	1 (1.3)	0	0	0
Alcohol poisoning	1 (1.3)	0	0	0

*n (%) denotes the number and percentage of patients with ≥1 treatment-emergent serious adverse event during the 24-week treatment period.

†Event led to patient hospitalization for removal.

MedDRA, Medical Dictionary for Regulatory Affairs; PT, MedDRA Preferred Term; SOC, MedDRA System Organ Class.

Supplementary Table 7 | Adverse events leading to discontinuation by primary SOC and PT

	**PRIME**		**PRIME2**	
**Primary SOC**<br>**PT, *n* (%)***	**Placebo**<br>***n*=75**	**Dupilumab 300 mg every 2 weeks**<br>***n*=75**	**Placebo**<br>***n*=82**	**Dupilumab 300 mg every 2 weeks**<br>***n*=77**
Any event	2 (2.7)	0	1 (1.2)	0
Neoplasms benign, malignant and unspecified (including cysts and polyps)	1 (1.3)	0	0	0
Hodgkin's disease	1 (1.3)	0	0	0
Skin and subcutaneous tissue disorders	1 (1.3)	0	1 (1.2)	0
Neurodermatitis	1 (1.3)	0	0	0
Urticaria	0	0	1 (1.2)	0

Only treatment-emergent adverse events reported by the investigator as reason for permanent intervention discontinuation are included.

**n* (%) denotes number and percentage of patients with ≥1 treatment-emergent adverse event during the 24-week treatment period leading to permanent intervention discontinuation.

MedDRA, Medical Dictionary for Regulatory Affairs; PT, MedDRA Preferred Term; SOC MedDRA System Organ Class.

Supplementary Table 8 | Overview of skin infections (except herpes infections) by MedDRA SOC and PT

	PRIME		PRIME2	
**Primary SOC**<br>**PT, *n* (%)***	**Placebo**<br>***n*=75**	**Dupilumab 300 mg every 2 weeks**<br>***n*=75**	**Placebo**<br>***n*=82**	**Dupilumab 300 mg every 2 weeks**<br>***n*=77**
Number of skin infection TEAEs	10	2	6	6
Patients with skin infection TEAEs, *n* (%)	7 (9.3)	2 (2.7)	5 (6.1)	4 (5.2)
Infections and infestations	7 (9.3)	2 (2.7)	5 (6.1)	4 (5.2)
Cellulitis	0	1 (1.3)	1 (1.2)	0
Folliculitis	2 (2.7)	1 (1.3)	2 (2.4)	0
Skin bacterial infection	0	0	0	1 (1.3)
Skin infection	0	0	0	1 (1.3)
Erysipelas	0	0	1 (1.2)	0
Tinea cruris	1 (1.3)	0	0	0
Tinea versicolor	0	0	0	2 (2.6)
Body tinea	1 (1.3)	0	0	0
Dermatitis infected	2 (2.7)	0	0	0
Paronychia	0	0	0	1 (1.3)
Fungal skin infection	1 (1.3)	0	0	0
Postoperative wound infection	2 (2.7)	0	0	0
Wound infection	0	0	1 (1.2)	0
Tinea pedis	1 (1.3)	0	0	0
Eye disorders	0	0	0	1 (1.3)
Chalazion	0	0	0	1 (1.3)

Skin infection adverse event (excluding herpetic infections) selection was based on CMQ30067 (Company MedDRA Query) and the blind data review. Skin infections were manually adjudicated.

**n* (%) denotes the number and percentage of patients with ≥1 treatment-emergent skin infection during the 24-week treatment period.

MedDRA, Medical Dictionary for Regulatory Affairs; PT, MedDRA Preferred Term; SOC, MedDRA System Organ Class; TEAE, treatment-emergent adverse event.

Supplementary Data 1

1. Original protocol and statistical analysis plan, and final protocol and final statistical analysis plan (including a summary of changes) of the LIBERTY-PN PRIME study (EFC16459)

2. Original protocol and statistical analysis plan, and final protocol and final statistical analysis plan (including a summary of changes) of the LIBERTY-PN PRIME2 study (EFC16460)
changes).

CLINICAL TRIAL PROTOCOL

**Protocol title:**	**A randomized, double blind, placebo-controlled, multi-center, parallel group study to evaluate the efficacy and safety of dupilumab in patients with prurigo nodularis who are inadequately controlled on topical prescription therapies or when those therapies are not advisable**
**Protocol number:**	**EFC16459**
**Amendment number:**	**Not applicable**
**Compound number (INN/Trademark):**	**SAR231893/REGN668 dupilumab/Dupixent**
**Study phase:**	**Phase 3**
**Short title:**	**Study of dupilumab for the treatment of patients with prurigo nodularis, inadequately controlled on topical prescription therapies or when those therapies are not advisable**<br>**LIBERTY-PN PRIME**
**Sponsor name:**	
**Legal registered address:**	
**Monitoring Team's Representative Name and Contact Information**	

Regulatory agency identifier number(s):

IND:	IND107969
EudraCT:	2019-003774-41
NCT:	Not applicable
WHO:	Not applicable
Other:	Not applicable

Approval Date: 25-Oct-2019 Total number of pages: 102

Any and all information presented in this document shall be treated as confidential and shall remain the exclusive property of Sanofi (or any of its affiliated companies). The use of such confidential information must be restricted to the recipient for the agreed purpose and must not be disclosed, published or otherwise communicated to any unauthorized persons, for any reason, in any form whatsoever without the prior written consent of Sanofi (or the concerned affiliated company); 'affiliated company' means any corporation, partnership or other entity which at the date of communication or afterwards (i) controls directly or indirectly Sanofi, (ii) is directly or indirectly controlled by Sanofi, with 'control' meaning direct or indirect ownership of more than 50% of the capital stock or the voting rights in such corporation, partnership or other entity

TABLE OF CONTENTS

CLINICAL TRIAL PROTOCOL 1

TABLE OF CONTENTS 2

LIST OF TABLES 6

1 PROTOCOL SUMMARY 7

1.1 SYNOPSIS 7

1.2 SCHEMA 13

1.3 SCHEDULE OF ACTIVITIES (SOA) 14

2 INTRODUCTION 19

2.1 STUDY RATIONALE 19

2.2 BACKGROUND 19

2.3 BENEFIT/RISK ASSESSMENT 21

3 OBJECTIVES AND ENDPOINTS 24

3.1 APPROPRIATENESS OF MEASUREMENTS 25

4 STUDY DESIGN 27

4.1 OVERALL DESIGN 27

4.2 SCIENTIFIC RATIONALE FOR STUDY DESIGN 27

4.3 JUSTIFICATION FOR DOSE 28

4.4 END OF STUDY DEFINITION 29

5 STUDY POPULATION 30

5.1 INCLUSION CRITERIA 30

5.2 EXCLUSION CRITERIA 32

5.3 LIFESTYLE CONSIDERATIONS 35

5.4 SCREEN FAILURES 36

6 STUDY INTERVENTION 37

6.1 STUDY INTERVENTION(S) ADMINISTERED 37
6.1.1 Noninvestigational medicinal products 38

6.2 PREPARATION/HANDLING/STORAGE/ACCOUNTABILITY 39

6.2.1 Storage and handling 39
6.2.2 Responsibilities 39
6.3 MEASURES TO MINIMIZE BIAS: RANDOMIZATION AND BLINDING 39
6.4 STUDY INTERVENTION COMPLIANCE 41
6.5 CONCOMITANT THERAPY 41
6.5.1 Rescue medicine....42
6.6 DOSE MODIFICATION....42
6.7 INTERVENTION AFTER THE END OF THE STUDY 42
7 DISCONTINUATION OF STUDY INTERVENTION AND PARTICIPANT DISCONTINUATION/WITHDRAWAL 43
7.1 DISCONTINUATION OF STUDY INTERVENTION 43
7.1.1 Definitive discontinuation 43
7.1.2 Temporary discontinuation....44
7.1.2.1 Rechallenge 45
7.2 PARTICIPANT DISCONTINUATION/WITHDRAWAL FROM THE STUDY....45
7.3 LOST TO FOLLOW UP 46
8 STUDY ASSESSMENTS AND PROCEDURES 47
8.1 EFFICACY ASSESSMENTS 47
8.1.1 Worst-itch numeric rating scale....48
8.1.2 Investigator’s global assessment for prurigo nodularis....48
8.1.3 Prurigo activity score....48
8.1.4 Dermatology life quality index 49
8.1.5 Pain and sleep numeric rating scales 49
8.1.6 Hospital anxiety and depression scale....49
8.1.7 Patient Global Impression of Change of disease and Patient Global Impression of Severity 50
8.1.8 Euroqol 5 dimensions questionnaire....50
8.1.9 Missed school/work days 50
8.1.10 Photography....51
8.2 SAFETY ASSESSMENTS 51
8.2.1 Physical examinations 51
8.2.2 Vital signs....51
8.2.3 Electrocardiograms 51
8.2.4 Clinical safety laboratory assessments....51

8.3 ADVERSE EVENTS AND SERIOUS ADVERSE EVENTS....52
8.3.1 Time period and frequency for collecting AE and SAE information....53
8.3.2 Method of detecting AEs and SAEs....54
8.3.3 Follow-up of AEs and SAEs....54
8.3.4 Regulatory reporting requirements for SAEs....54
8.3.5 Pregnancy....55
8.3.6 Guidelines for reporting product complaints....55
8.4 TREATMENT OF OVERDOSE....55
8.5 PHARMACOKINETICS....56
8.5.1 Systemic drug concentration and antidrug antibodies....56
8.5.1.1 Sampling time....56
8.5.1.2 Handling procedures....56
8.5.1.3 Bioanalytic method....56
8.6 PHARMACODYNAMICS....57
8.7 GENETICS....57
8.8 BIOMARKERS....57
8.9 MEDICAL RESOURCE UTILIZATION AND HEALTH ECONOMICS....58
9 STATISTICAL CONSIDERATIONS....59
9.1 STATISTICAL HYPOTHESES....59
9.2 SAMPLE SIZE DETERMINATION....59
9.3 POPULATIONS FOR ANALYSES....60
9.4 STATISTICAL ANALYSES....60
9.4.1 Efficacy analyses....60
9.4.2 Safety analyses....62
9.4.3 Other analyses....63
9.5 INTERIM ANALYSES....63
9.5.1 Data Monitoring Committee (DMC)....63
9.5.2 Unblinding plan....63
10 SUPPORTING DOCUMENTATION AND OPERATIONAL CONSIDERATIONS....64
10.1 APPENDIX 1: REGULATORY, ETHICAL, AND STUDY OVERSIGHT CONSIDERATIONS....64
10.1.1 Regulatory and Ethical Considerations....64
10.1.2 Financial disclosure....64
10.1.3 Informed consent process....65

10.1.4 Data protection 65
10.1.5 Committees structure 66
10.1.6 Dissemination of clinical study data 66
10.1.7 Data quality assurance 67
10.1.8 Source documents 67
10.1.9 Study and site closure 68
10.1.10 Publication policy 68

10.2 APPENDIX 2: CLINICAL LABORATORY TESTS 69

10.3 APPENDIX 3: ADVERSE EVENTS: DEFINITIONS AND PROCEDURES FOR RECORDING, EVALUATING, FOLLOW-UP, AND REPORTING 71

10.4 APPENDIX 4: CONTRACEPTIVE GUIDANCE AND COLLECTION OF PREGNANCY INFORMATION 75

10.5 APPENDIX 5: GENETICS 78

10.6 APPENDIX 6: LIVER AND OTHER SAFETY: SUGGESTED ACTIONS AND FOLLOW-UP ASSESSMENTS 79

10.7 APPENDIX 7: CLINICIANS-REPORTED OUTCOMES AND PATIENT-REPORTED OUTCOMES 82
10.7.1 Worst itch numeric rating scale (WI-NRS) 82
10.7.2 Investigator's global assessment of prurigo nodularis (IGA PN) 83
10.7.3 Prurigo activity score (PAS) 84
10.7.4 Dermatology life quality index (DLQI) 86
10.7.5 Hospital anxiety and depression scale (HADS) 87
10.7.6 EQ-5D-5L 88
10.7.7 Pain numeric rating scale 89
10.7.8 Sleep numeric rating scale 90
10.7.9 Patient Global Impression of Change of disease (PGIC) 90
10.7.10 Patient Global Impression of Severity (PGIS) 91
10.7.11 Missed school/work days 92

10.8 APPENDIX 8: DEFINITION OF ANAPHYLAXIS 96

10.9 APPENDIX 9: LIST OF OPPORTUNISTIC INFECTIONS 96

10.10 APPENDIX 10: COUNTRY-SPECIFIC REQUIREMENTS 97

10.11 APPENDIX 11: ABBREVIATIONS 97

11 REFERENCES 100

LIST OF TABLES

Table 1 - Objectives and endpoints 24
Table 2 - Overview of study interventions administered 37
Table 3 - Summary of handling procedures 56
Table 4 - Summary of bioanalytical methods for functional dupilumab and anti-dupilumab antibodies 56
Table 5 - Populations for analyses 60
Table 6 - Efficacy analyses 61
Table 7 - Safety analyses 62
Table 8 - Other analyses 63
Table 9 - Protocol-required laboratory assessments 69

1 PROTOCOL SUMMARY

1.1 SYNOPSIS

Protocol title: A randomized, double blind, placebo-controlled, multi-center, parallel group study to evaluate the efficacy and safety of dupilumab in patients with prurigo nodularis who are inadequately controlled on topical prescription therapies or when those therapies are not advisable

Short title: Study of dupilumab for the treatment of patients with prurigo nodularis, inadequately controlled on topical prescription therapies or when those therapies are not advisable

LIBERTY-PN PRIME (PRurigo Nodularis Itch Minimization Evaluation)

Rationale:

Prurigo nodularis (PN) is a skin disease characterized by multiple, intensely itchy skin eruptions in symmetrically distributed areas of the extremities (1). The main symptom is prolonged, repetitive and uncontrollable rubbing, scratching and uncontrollable itching which leads to hyperkeratotic eroding papules and nodules on the skin. It is difficult to treat and entails a high disease burden. Approximately 50% of patients have either past or current history of atopic dermatitis (AD) or other atopic disorders (2). While case reports suggest that dupilumab may successfully treat patients with PN regardless of a preexisting atopic background (3, 4, 5, 6, 7), it remains unclear to what extent Type 2 cytokines play a role in the pathogenesis in those PN lesions not associated with atopy (8).

There are no United States (US) Food and Drug Administration (FDA) or European Medicines Agency (EMA) approved targeted therapies indicated for the treatment of PN, whether atopic or non-atopic forms. Topical corticosteroids (TCS) and topical calcineurin inhibitors (TCI) are often used initially. While there is a mechanistic rationale for their use, no rigorous clinical studies confirming their efficacy were identified.

Dupilumab is a human monoclonal IgG4 antibody that inhibits interleukin (IL)-4 and IL-13 signaling by specifically binding to the IL-4Rα subunit shared by the IL-4 and IL-13 receptor complexes. Dupilumab inhibits IL-4 signaling via the Type I receptor and both IL-4 and IL-13 signaling through the Type II receptor.

Dupilumab has shown clinical efficacy in multiple diseases with underlying Type 2 inflammation such as AD, asthma, chronic rhinosinusitis with nasal polyposis (CRSwNP), and eosinophilic esophagitis (EoE).

Recently, case series were published that suggest dupilumab may also be effective in the treatment of PN (3, 4, 5, 6, 7).

Type 2 cytokine involvement is implicated to some extent in all forms of PN. It is postulated that dupilumab may play a fundamental role in pruritus based upon its ability to block IL4R activity at the level of the sensory dorsal root ganglion (9). The present protocol will evaluate the efficacy and safety of dupilumab in PN.

Objectives and endpoints

Objectives	Endpoints
**Primary**	
• To demonstrate the efficacy of dupilumab on itch response in patients with PN, inadequately controlled on topical prescription therapies or when those therapies are not advisable.	• Proportion of participants with improvement (reduction) in worst-itch numeric rating scale (WI-NRS) by ≥4 from baseline to Week 12.
**Secondary**	
• To demonstrate the efficacy of dupilumab on additional itch endpoints in patients with PN, inadequately controlled on topical prescription therapies or when those therapies are not advisable.	• **[Key secondary endpoint]** Proportion of participants with improvement (reduction) in WI-NRS by ≥4 from baseline to Week 24.<br>• Time to onset of effect on pruritus as measured by proportion of participants with an improvement (reduction) in WI-NRS by ≥4 from baseline during the 24-week treatment period.<br>• Change from baseline in WI-NRS at Week 12 and Week 24.<br>• Percent change from baseline in WI-NRS at Week 12 and Week 24.<br>• Percent change from baseline in WI-NRS over time until Week 24.<br>• Proportion of participants with WI-NRS reduction ≥4 over time until Week 24.<br>• Onset of action in change from baseline in WI-NRS (first $p < 0.05$ difference from placebo in the daily WI-NRS that remains significant at subsequent measurements) until Week 12.
• To demonstrate efficacy of dupilumab on skin lesions of PN.	• Proportion of participants with Investigator's Global Assessment 0/1 score for PN-Stage (IGA PN-S) at Week 4, Week 8, Week 12, and Week 24.<br>• Change from baseline in IGA PN-S score at Week 4, Week 8, Week 12, and Week 24.<br>• Proportion of participants with Investigator's Global Assessment 0/1 score for PN-Assess (IGA PN-A) at Week 4, Week 8, Week 12, and Week 24.
• To demonstrate the improvement in health-related quality of life (HRQoL).	• Change from baseline in HRQoL, as measured by Dermatology Life Quality Index (DLQI) to Week 12 and Week 24.
• To evaluate safety outcome measures.	• Percentage of participants experiencing treatment-emergent adverse events (TEAEs) or serious adverse events (SAEs) from baseline through Week 24.
• To evaluate immunogenicity of dupilumab.	• Incidence of treatment-emergent antidrug antibodies (ADA) against dupilumab over time.

Overall design:

This study is a multi-center, 24-week treatment, parallel, double-blind, randomized, placebo-controlled study to evaluate the use of dupilumab in patients with PN inadequately controlled on topical prescription therapies or when those therapies are not advisable. The study will assess the effect of dupilumab on itch improvement as well as its effect on PN lesions, on patients' HRQoL, anxiety and depression, sleep quality and skin pain, and overall health status.

Disclosure Statement: This is a Parallel, Treatment study, with 2 arms, that is blinded/masked for participants and investigators.

Number of participants:

Approximately 150 participants will be randomized 1:1. This corresponds to approximately 75 participants who will be randomly assigned to each intervention arm.

Intervention groups and duration:

Participants who satisfy the inclusion and exclusion criteria will be randomized (1:1) to one of the following investigational medicinal product (IMP) treatment groups:

- Dupilumab 300 mg
- Matched placebo

Duration of study period (per participant)

- Screening period (2-4 weeks)
- Randomized IMP intervention period (24 weeks)
- Follow-up period (12 weeks)

Study interventions

Investigational medicinal product:

Dupilumab 300 mg and placebo matching dupilumab 300 mg supplied in prefilled syringes that are visually indistinguishable.

Dupilumab

- Formulation: dupilumab 300 mg: a 150 mg/mL dupilumab solution in a pre-filled syringe to deliver 300 mg in a 2 mL injection.
- Route of administration: subcutaneous (SC) injection.
- Dose regimen: 300 mg every 2 weeks (Q2W) after an initial loading dose of 600 mg (2 injections of 300 mg) on Day 1.

Placebo

- Formulation: identical formulation to the active 300 mg formulation without dupilumab, in a pre-filled syringe to deliver placebo in a 2 mL injection.
- Route of administration: SC injection.
- Dose regimen: 1 injection Q2W after an initial loading dose (2 injections) on Day 1.

Noninvestigational medicinal products

Participants will be required to apply moisturizers (emollients) once or twice daily for at least the 7 consecutive days immediately before Day 1 and continue until Week 36.

If participants are on a stable regimen of low to medium potency TCS or TCI at the screening visit, they can continue their topical steroid application once daily without tapering from Screening to Week 24. If specific lesions resolve, the participant can stop applying steroids to those sites but are permitted to continue applying to persistent lesions. If participants are on stable regimens of high potency or superpotent steroids, participants should decrease potency to medium potency TCS and continue to apply daily from screening to Week 24. Occlusion is not allowed from Screening to Week 24.

Participants can be rescued with high potency or superpotent TCS/TCI as needed throughout the study.

Post-trial access to study medication

The sponsor does not plan to provide post-trial access to the study medication.

Statistical considerations:

- **Randomization**
  - The participants will be randomized to dupilumab or placebo in 1:1 ratio with stratification factors of documented history of atopy (atopic or non-atopic), stable use of TCS/TCI (yes or no), and country/territory code. Number of participants with active mild AD upon study entry will represent up to 10% of the atopic participants. Both the atopic and the non-atopic PN population will be capped at 60% of the total enrolled population.
- **Sample size calculation**
  - The primary endpoint is the proportion of participants with a WI-NRS reduction of ≥4 from baseline to Week 12. Assuming a response rate of [redacted]% and [redacted]% in placebo and dupilumab respectively, 56 participants/arm will provide 90% power to detect the difference of [redacted]% between dupilumab and placebo with Fisher exact test at 2-sided level of 0.05. Assuming 15% drop out during the 12 weeks of treatment, the target is to randomize 75 participants/arm to allow for up to 10% of atopic patients having active mild AD upon study entry.

- **Primary analysis**
  - The analysis population for the efficacy endpoints will be the intent-to-treat (ITT) population defined as all randomized participants analyzed according to the treatment group allocated by randomization regardless if treatment kit is used or not.
  - The primary analysis on WI-NRS reduction ≥4 at Week 12 will be conducted by using Cochran-Mantel-Haenszel (CMH) test stratifying by stratification factors of documented history of atopy (atopic or non-atopic), stable use of TCS/TCI (yes or no), and region (countries combined). For participants discontinuing the study treatment before Week 12, their off-study treatment values measured up to Week 12 will be included in the analysis. Participants taking selected prohibited medications and/or rescue medications (details of selection will be specified in the statistical analysis plan [SAP]) prior to Week 12 or have missing data at Week 12 will be considered non-responders.
  - Sensitivity analyses using alternative methods will be performed to handle missing data and/or data collected after participants taking selected prohibited medications and/or rescue medications. A subgroup analysis will be performed excluding participants with a current diagnosis of AD. More details of the sensitivity and subgroup analyses will be specified in the SAP.
- **Analysis of secondary endpoints**
  - Secondary efficacy endpoints that measure binary responses will be analyzed in the same fashion as the primary endpoint.
  - Time-to-event secondary efficacy endpoint will be analyzed using the Cox proportional hazards model, including treatment, stratification factors (countries combined to region). The hazards ratio, its 95% confidence interval and p-value will be reported. Kaplan-Meier curves will be also provided.
  - Continuous secondary efficacy endpoints will be analyzed using a hybrid method of the worst observation carried forward (WOCF) and multiple imputations. Data of participants taking selected prohibited medications and/or rescue medications will be set to missing after the medication usage, and the worst postbaseline value on or before the time of the medication usage will be used to impute missing endpoint value (for participants whose postbaseline values are all missing, the baseline will be used to impute). Participants who discontinue the treatment prematurely are encouraged to follow the planned clinical visits and in those participants who did not take the selected prohibited medications and/or rescue medications, all data collected after treatment discontinuation will be used in the analysis. For these participants, missing data may still happen despite all efforts have been tried to collect the data after treatment discontinuation. For participants who discontinue due to lack of efficacy, all data collected after discontinuation will be used in the analysis, and a WOCF approach will be used to impute missing data if needed. For participants who discontinued not due to lack of efficacy, a multiple imputation approach will be used to impute missing endpoint value, and this multiple imputation will use all participants excluding participants who have taken the selected prohibited medications and/or rescue medications prior to timepoint of endpoint of interest and excluding patients who

VV-CLIN-0637729 1.0

discontinue due to lack of efficacy. Each of the imputed complete data will be analyzed by fitting an analysis of covariance (ANCOVA) model with treatment group, stratification factors (countries combined into region) and relevant baseline measurement as covariates in the model. Statistical inference obtained from all imputed data will be combined using Rubin's rule.

- The safety variables will be summarized using descriptive statistics.
- Antidrug antibodies variables including treatment-emergent ADA will be summarized using descriptive statistics by treatment group.

- **Multiplicity considerations**
  - The multiplicity procedure is proposed to control the overall Type-I error rate for testing the primary endpoint and the key secondary endpoints. Detailed hierarchical testing procedure will be defined in the study SAP. The study is considered positive when the primary endpoint achieves statistical significance.
- **Planned database lock date**
  - A primary database lock will be performed when all randomized participants have completed their 24-week treatment phase.
  - The database will be updated at the end of the study for all participants to include the post-treatment follow-up information and updates for the events previously ongoing at the time of the primary lock.
- **Unblinding plan**
  - The unblinding plan will be detailed in the SAP or in a separate unblinding plan.

Data Monitoring Committee: No

1.2 SCHEMA

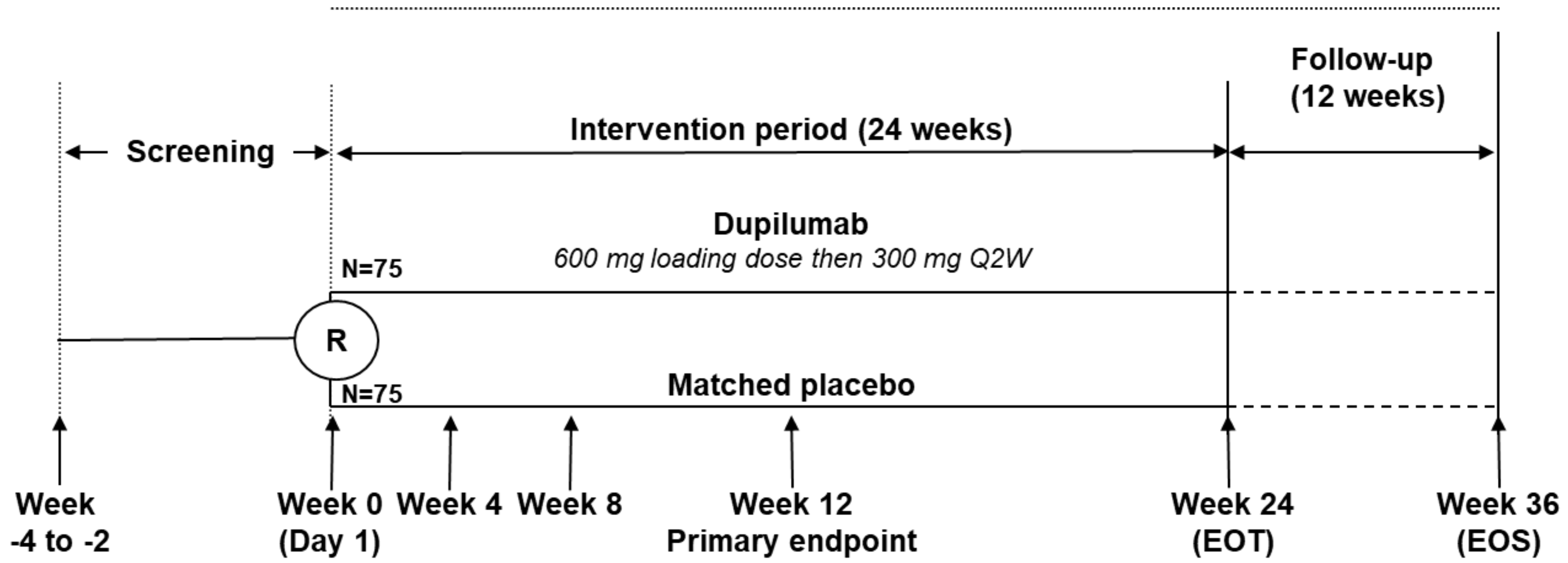

Background: low to medium potency TCS/TCI as background therapy permitted (maintain dose from screening to EOT)

Dupilumab 300 mg Q2W, administered as 1 SC injection of dupilumab 300 mg (2 mL)
A 600 mg loading dose will be administered on Day 1.
Matched placebo is prepared in the same formulation without the addition of protein (ie, the active substance)

EOS: end of study; EOT: end of treatment; PN: prurigo nodularis; Q2W: every 2 weeks; R: randomization; SC: subcutaneous; TCI: topical calcineurin inhibitors; TCS: topical corticosteroids.

1.3 SCHEDULE OF ACTIVITIES (SOA)

Procedure	Screening (2 to 4 weeks before Day 1)	Intervention period (Weeks)					Follow-up (12 weeks)	Notes
		0 (Day 1)	4	8	12	24		
**Visit**	**1**	**2**[a]	**3**	**4**	**5**	**6**[b] **(EOT)**	**7 (EOS)**	Visit window: ±3 days for Visits 3, 4, 5, 6, and 7.
**Screening and baseline**								
Informed consent	X							Separate consent to be obtained for optional procedures (skin biopsy, DNA and RNA sampling, serum/plasma sampling for archival, and photography; HIV test if specific consent locally required).
Medical history	X							
Prior and concomitant medication	X	X	X	X	X	X	X	Concomitant medication will be collected throughout the study.
Demography	X							
Inclusion and exclusion criteria	X	X						
Hepatitis[c], HIV serology, TB test	X							HBs Ag, HBs Ab, HBc Ab, HCV Ab, HIV screen (Anti-HIV-1 and HIV-2 antibodies), TB test (performed locally if required and results noted in the eCRF).
Randomization		X						
**Study intervention**[d]								
Dispense or download e-diary	X	X	X	X	X	X	X	Device will be dispensed at Screening (including instructions for use). At EOS, the e-diary will be downloaded and returned to the site.
Participant e-diary training	X	X						
Call IVRS/IWRS	X	X	X	X	X	X	X	At screening visit, IVRS/IWRS will be called after medical history check.
IMP administration		X	X	X	X			IMP administration Q2W (±3 days) from Week 0 until Week 22. Between visits, the participants will self-inject IMP at home or may choose to have injections administered at home by a caregiver or at the study site.

Procedure	Screening (2 to 4 weeks before Day 1)	Intervention period (Weeks)					Follow-up (12 weeks)	Notes
		0 (Day 1)	4	8	12	24		
**Visit**	1	2[a]	3	4	5	6[b] (EOT)	7 (EOS)	Visit window: ±3 days for Visits 3, 4, 5, 6, and 7.
**Safety**[d]								
Physical examination	X	X				X	X	Including skin (full body skin exam), nasal cavities, eyes, ears, respiratory, cardiovascular, gastrointestinal, neurological, lymphatic, and musculoskeletal systems.
Vital signs	X	X	X	X	X	X	X	Including SBP and DBP (mmHg), pulse rate (beats per minute), body temperature (°C), and respiratory rate. Body weight will be recorded at Visits 1, 6, and 7 only. Height will be recorded at Visit 1 only.
12-lead ECG	X					X		Locally collected and read.
Hematology, biochemistry[e]	X	X	X	X	X	X	X	Including hemoglobin, hematocrit, platelet count, total WBC count, differential count, total RBC count, creatinine, BUN, glucose, lactate dehydrogenase, uric acid, total cholesterol, total protein, albumin, total bilirubin, ALT, AST, alkaline phosphatase, electrolytes (sodium, potassium, chloride), bicarbonate, and creatine phosphokinase.
Urinalysis	X	X	X	X	X	X		Dipstick analysis including specific gravity, pH, glucose, ketones, blood, protein, nitrite, leukocyte esterase, urobilinogen and bilirubin. If positive for protein and/or red blood cells, microscopic analysis will be performed by the central laboratory. Creatinine and leukotriene will be tested by the central laboratory.
CD4 T cell count and HIV viral load	X					X		For participants with HIV history only.
Pregnancy test (WOCBP only)[g]	Serum	Urine	Urine	Urine	Urine	Urine	Urine	In between visit urine pregnancy tests will be performed at home (Weeks 16, 20, 28, 32).
AE reporting, including SAEs	X	X	X	X	X	X	X	
Rescue medication use	←--→							

Procedure	Screening (2 to 4 weeks before Day 1)	Intervention period (Weeks)					Follow-up (12 weeks)	Notes
		0 (Day 1)	4	8	12	24		
**Visit**	1	2[a]	3	4	5	6[b] (EOT)	7 (EOS)	Visit window: ±3 days for Visits 3, 4, 5, 6, and 7.
**PK and ADA**[d, f]								
Serum PK samples for dupilumab concentration		X	X	X	X	X	X	
Anti-dupilumab antibody		X			X	X	X	
**Biomarkers**[d]								
Total serum IgE		X	X	X	X	X	X	
Optional skin biopsy (substudy)		X				X		The sample will be taken from lesion and non-lesion skin using punch biopsy.
Optional serum/plasma for archival samples		X	X	X	X	X	X	Archive serum and plasma samples will be collected and stored for possible future analysis of potential biomarkers of drug response, disease activity, safety, and the Type 2 inflammation pathway.
Optional DNA (whole blood) sample[h]		X						
Optional RNA (whole blood) sample[h]		X				X		
**Efficacy**[d]								
WI-NRS	NRS: once a day from Screening to EOS							To be recorded once a day in e-diary.
Pain-NRS								
Sleep-NRS								

Procedure	Screening (2 to 4 weeks before Day 1)	Intervention period (Weeks)					Follow-up (12 weeks)	Notes
		0 (Day 1)	4	8	12	24		
**Visit**	**1**	**2**[a]	**3**	**4**	**5**	**6**[b] **(EOT)**	**7 (EOS)**	Visit window: ±3 days for Visits 3, 4, 5, 6, and 7.
DLQI		X	X	X	X	X	X	Entered by participant during site visit on a tablet provided to the site.
HADS		X			X	X	X	
EQ-5D-5L		X			X	X	X	
PGIS	X	X	X	X	X	X		
PGIC			X	X	X	X		
Missed school/work days (baseline version)		X						
Missed school/work days (postbaseline version)					X	X	X	
Modified PAS (screening-baseline version)	X	X						Investigator's assessment to be entered on a tablet provided to the site.
Modified PAS (follow-up version)			X	X	X	X	X	
IGA PN-A and IGA PN-S		X	X	X	X	X	X	
Optional photography (substudy)		X	X	X	X	X	X	

a All assessments at Visit 2 (Day 1) are to be conducted pre-IMP dose with the exception of the assessment of local tolerability of SC injections.

b Participants who discontinue the study treatment prematurely (prior to completing the 24-week treatment period) will perform the EOT assessments at the time of discontinuation to assure a complete clinical assessment in close temporal proximity to the premature termination of study treatment. In addition, to allow assessment of participant outcomes over the stipulated study period, participants will be asked and encouraged to complete all remaining study visits and participate in all assessments according to the visit schedule.

c In case of results showing HBs Ag (negative), HBs Ab (negative) and HBc Ab (positive), an HBV DNA testing may be performed prior to randomization to rule out a false positivity if the Investigator believes the participant is a false positive, or to clarify the serological status if the Investigator finds it unclear to interpret in absence of known HBV infection. In case of results showing HCV Ab (positive), an HCV RNA testing may be performed to rule out a false positivity, if the participant is a false positive per Investigator's judgement.

d Assessments/procedures should be conducted in the following order: PRO, investigator assessments, safety and laboratory assessments (including sample collection for ADA, PK, biomarker, and optional DNA and RNA), and administration of IMP.

e Refer to Section 10.2 and central lab manual for collection details.

f In the event of any SAE, any AE of severe injection site reaction lasting longer than 24 hours, or any AESI of anaphylactic reaction or systemic allergic reaction that is related to IMP and require treatment, PK and ADA samples will be collected at or near the onset of the event for any additional analysis if required or for archival purposes.

g Pregnancy will lead to definitive treatment discontinuation in all cases. Female participants will be supplied with urine dipsticks for use between visits.

h DNA sample should be collected at the Day 1 visit, but can be collected at any visit during the study. RNA samples must be collected before the administration of the first dose of study drug and at Week 24.

ADA: antidrug antibodies; AE: adverse event; AESI: adverse event of special interest; ALT: alanine aminotransferase; AST: aspartate aminotransferase; BUN: blood urea nitrogen; DBP: diastolic blood pressure; DLQI: Dermatology Life Quality Index; DNA: deoxyribonucleic acid; ECG: electrocardiogram; eCRF: electronic case report form; EOS: end of study; EOT: end of treatment; EQ-5D-5L: Euroqol 5 dimensions 5 levels; HADS: Hospital Anxiety and Depression Scale; HBc Ab: hepatitis B core antibody; HBs Ab: hepatitis B surface antibody; HBs Ag: hepatitis B surface antigen; HBV: hepatitis B virus; HCV: hepatitis C virus; HCV Ab: hepatitis C virus antibody; HIV: human immunodeficiency virus; Ig: immunoglobulin; IGA PN: Investigator's Global Assessment for prurigo nodularis; IGA PN-A: IGA PN-Activity; IGA PN-S: IGA PN-Stage; IMP: Investigational Medicinal Product; IVRS: interactive voice response system; IWRS: interactive web response system; NRS: numeric rating scale; PAS: prurigo activity score; PGIC: Patient Global Impression of Change; PGIS: Patient Global Impression of Severity; PK: pharmacokinetic; PRO: patient reported outcome; Q2W: every 2 weeks; RBC: red blood cells; RNA: ribonucleic acid; SAE: serious adverse event; SBP: systolic blood pressure; SC: subcutaneous; TB: tuberculosis; WBC: white blood cells; WI-NRS: worst itch numeric rating scale; WOCBP: woman of childbearing potential.

2 INTRODUCTION

Dupilumab is a human monoclonal IgG4 antibody that inhibits IL-4 and IL-13 signaling by specifically binding to the IL-4Rα subunit shared by the IL-4 and IL-13 receptor complexes. Dupilumab inhibits IL-4 signaling via the Type I receptor and both IL-4 and IL-13 signaling through the Type II receptor. Blocking IL-4Rα with dupilumab inhibits IL-4 and IL-13, key cytokines that drive Type 2 inflammatory responses, including the release of proinflammatory cytokines, chemokines, nitric oxide, and IgE.

Dupilumab is approved by major regulatory agencies for use in AD and in asthma in adults and adolescents, and in the US for CRSwNP in adults.

2.1 STUDY RATIONALE

Prurigo nodularis is a skin disease characterized by multiple, intensely itchy skin eruptions in symmetrically distributed areas of the extremities (1). It is difficult to treat and entails a high disease burden.

Dupilumab has shown efficacy in multiple diseases with underlying Type 2 inflammation such as AD, asthma, CRSwNP and EoE. In light of the published research suggesting a principal role for Type 2 cytokines in the pathogenesis of PN (see Section 2.2), the Sponsor proposes that selective and simultaneous inhibition of IL-4 and IL-13 signaling such as that achieved with dupilumab treatment may provide clinical benefit in relieving the signs and symptoms of PN, especially in the high unmet need population of patients inadequately treated with, or ineligible for, TCS.

Recently, case series were published that suggest that dupilumab may also be effective in the treatment of PN (3, 4, 5, 6). Type 2 cytokine involvement is implicated in all forms of PN. This is consistent with a recent case series of nine patients without an atopic history who benefitted from dupilumab treatment (7).

The primary hypothesis of the study is that dupilumab will be effective in reducing itch and the secondary hypothesis is that it will reduce and promote the healing of lesions of PN, in patients who are inadequate responders to topical prescription therapies or when those therapies are not advisable.

2.2 BACKGROUND

Prurigo nodularis is a skin disease characterized by multiple, intensely itchy skin eruptions in symmetrically distributed areas of the extremities (1). The main symptom is prolonged, repetitive and uncontrollable rubbing, scratching and uncontrollable itching which leads to hyperkeratotic eroding papules and nodules on the skin. A broadly accepted definition for chronic prurigo has been published by the European Academy of Dermatology and Venereology (EADV) (10). The convened experts agreed that chronic prurigo should be used as an umbrella term for the range of clinical manifestations (eg, papular, nodular, plaque or umbilicated types). Prurigo nodularis is

considered a distinct disease defined by the presence of chronic pruritus for ≥6 weeks, history and/or signs of repeated scratching and multiple localized/generalized pruriginous skin lesions (whitish, hyperpigmented, or pink papules, nodules and/or plaques).

Pathophysiological studies in PN patients reveal increased dermal nerve fiber density and changes in many types of skin cells. These skin cells cause inflammation and pruritus (severe, constant itching) through the release of cytokine IL-31, tryptase, prostaglandins, eosinophil cationic protein, histamine, and neuropeptides, such as substance P, calcitonin gene-related peptide and nerve growth factor (11). The itch caused by primary dermatoses or systemic disorders is postulated to cause an itch scratch cycle which leads to neuronal hypersensitization. This neuronal hypersensitization is the final common pathway which drives the symptomatology of PN (1). Fukushi et al. studied 22 PN skin biopsies and found that 86% of them showed activation of STAT6, a marker for Type 2 inflammatory pathway activation downstream of the Type 2 cytokines IL-4 and IL-13 (12). Consistent with this, IL-4 and IL-5 messenger RNA expression is upregulated in the skin of PN patients (13). Importantly, these two studies did not mention whether these skin biopsy specimens came from patients with or without atopy. The etiology of the profound pruritus in this disease is thought to be driven by high Type 2 inflammation driving activity at the level of the sensory neuron. Human sensory neurons in skin that transmit the sensation of itch are known to express IL- 4Rα, IL-13Ra1, and IL-31RA (14). On testing for functional stimulation, IL-4 was shown to enhance responsiveness to other pruritogens like histamine (14). These findings support that Type 2 cytokines may play a principal role in the pathogenesis of PN.

Due to the central manifestation of itch, PN carries a significant burden of disease (10, 15). The effect on quality of life due to PN has been reported to be higher than other common skin disorders like AD and psoriasis (16). Patients report chronic sleep loss due to constant itching; constant burning, stinging, and pain at affected area; and chronic depression, anxiety, anger, disgust, and shame; and hence overall experience a great impact on their quality of life. According to a 5 year cross-sectional study on 909 adult PN patients in the John Hopkins hospital system (15), PN is a key contributing factor to mood disorders such as anxiety and depression. Of all patients with chronic pruritus, 70% have some type of psychiatric comorbidity, and the severity of depression in patients with chronic pruritus correlates with intensity of itch. The concomitant psychologic component of PN likely contributes to the challenging nature of treating this disease.

Data on the epidemiology of PN are limited. Most studies show a predominance of older patients with a median age of more than 50 years (2, 10, 15). Prurigo nodularis is only occasionally observed in younger patients, in whom it is often associated with atopic conditions (17).

There are no FDA or EMA approved targeted therapies indicated for the treatment of PN, whether atopic or non-atopic forms; the lesions of atopic and non-atopic PN are clinically indistinguishable and treatment options remain the same. Topical corticosteroids and TCI are often used initially. While there is a mechanistic rationale for their use, no rigorous clinical studies confirming their efficacy were identified. Anecdotal feedback obtained from dermatological experts indicate variable, modest efficacy. For thicker lesions, TCS is also administered intralesionally. Lesional cryotherapy is another available topical treatment; case reports indicate temporary relief. Antihistamines and antileukotrienes are occasionally used; their efficacy, however, is not supported by well conducted, randomized clinical trials and is rated low

by patients (18). Phototherapy, in particular narrowband ultraviolet B (UVB), is occasionally added in patients not responding to topical pharmacotherapy. While some improvements with phototherapy have been reported, they remain usually partial and complete remissions are uncommon.

Oral immunosuppressants such as methotrexate and cyclosporine have been used off-label with some success as reported based on case reports and retrospective (18). Use of cyclosporine in PN is limited by commonly recognized toxicities including hypertension, impaired renal and hepatic function, and potential for increased susceptibility to infections and cancer, particularly skin cancer, due to decreased cancer immunosurveillance (19). Methotrexate has well established toxicities, in particular, myelosuppression and hepatotoxicity. In addition, the broad immunosuppression caused by all these drugs carries an increased risk of developing serious bacterial, fungal, viral, and mycobacterial infections.

Neuromodulatory agents such as gabapentin and anti-inflammatory agents such as thalidomide have been used in PN with varying degrees of success (18), but have also considerable adverse effects. Adverse effects of thalidomide include peripheral neuropathies, sedation, dizziness and teratogenicity, while adverse effects of gabapentin include headache, sedation and dizziness.

Overall, despite the use of multiple treatments, many patients with PN remain uncontrolled, and some of the available therapies are associated with serious potential adverse reactions. Given the lack of targeted treatments and the suboptimal efficacy associated with currently available therapies, there remains a significant unmet need in patients with PN.

Dupilumab has shown efficacy in multiple diseases with underlying Type 2 inflammation such as AD, asthma, CRSwNP, and EoE. The most compelling data suggesting that PN is driven by Type 2 inflammation are the many case series of PN patients successfully treated with dupilumab. Recently, five case series were published that suggest that dupilumab may also be effective in the treatment of PN: Mollanazar et al. (4 patients, 3 of those without history of AD) (3), Beck et al. (3 patients) (4), Rambhia et al. (2 patients) (5), Calugareanu et al. (one patient) (6), and Zhai et al. (9 patients, all without history of AD) (7). These case series reported major improvements in treatment-refractory cases of PN after treatment with dupilumab 300 mg Q2W (after an initial loading dose of 600 mg). These published case reports show rapid and substantial effects of dupilumab on itch, and somewhat less well documented effects also on lesions in patients with PN refractory to standard treatment.

2.3 BENEFIT/RISK ASSESSMENT

Dupilumab solution for injection is currently authorized:

- In over 40 countries worldwide including the US, European Union (EU) (Centralised Procedure), and Japan for the treatment of adults with inadequately controlled moderate-to-severe AD. In the US and EU, it has also been authorized for use in adolescent patients (≥12 years) with inadequately controlled moderate-to-severe AD.

- In the US for use in moderate-to-severe eosinophilic or oral steroid dependent asthma, in the EU for severe Type 2 asthma in adults and adolescents, and in Japan for use in adults and adolescents (≥12 years) with severe or refractory bronchial asthma,
- In the US for use in adults with inadequately controlled CRSwNP.

Dupilumab has shown clinically relevant benefit in several Type 2 driven immunological disorders such as AD, bronchial asthma, CRSwNP, and EoE. In asthma and AD indications, studies were also conducted in adolescents and similar benefit to adults was observed.

While the mechanism of PN is not well-known, there is some evidence of a Type 2 immunologic signature in prurigo nodule skin biopsy specimens and activation of Type 2 transcription factors STAT3 and STAT6 in the epidermis (12, 13). Therefore, it is hypothesized that dupilumab may show significant clinical benefit in the treatment of PN. This hypothesis is supported by a series of case reports that show dupilumab treatment resulting in a significant reduction in pruritus in 19 PN patients (3, 4, 5, 6, 7).

No tissue targets or specific hazards to humans were identified in nonclinical general and reproductive toxicology studies.

Dupilumab has an extensive safety database. As of the 28 March 2019 (data lock point), 8798 subjects were enrolled into the development program for dupilumab and are included in the safety population treated: 242 as healthy volunteers, 4227 from AD studies, 3377 from asthma studies, 782 from CRSwNP studies, 53 from EoE studies, 117 from grass allergy and peanut allergy studies. The number of subjects exposed to dupilumab in clinical studies was 7781 (218 in healthy volunteer studies, 3931 in AD studies, 3073 in asthma studies, 470 in CRSwNP studies, 26 in EoE studies, and 63 in grass allergy and peanut allergy studies). Based on the sales figure retrieved from Intercontinental Marketing Services Health and using the World Health Organization's defined average daily dose for dupilumab of 21.4 mg, the cumulative post marketing exposure to dupilumab is estimated to be 38 816 patient years (01 January 2017 through 31 December 2018).

Dupilumab was generally well tolerated in all populations tested in clinical development programs. The adverse drug reactions (ADRs) identified to date for dupilumab include injection site reactions, conjunctivitis, oral herpes, conjunctivitis allergic, conjunctivitis bacterial, herpes simplex, blepharitis, dry eye, eye pruritus, and eosinophilia. These ADRs occur with relatively low frequency with dupilumab treatment, and were generally mild or moderate, transient, and manageable. More significant serious allergic reactions were very rare. Importantly, no increased overall infection risk was observed in patients treated with dupilumab.

Systemic hypersensitivity has been established as an important identified risk with dupilumab. As protein therapeutics, all monoclonal antibodies are potentially immunogenic. Rare serious and systemic hypersensitivity reactions have been observed in the dupilumab program including serum sickness/serum sickness-like reaction in the adult AD program and anaphylaxis related to dupilumab in the adult asthma clinical trials.

It is hypothesized that dupilumab in patients with PN will have a favorable safety profile as observed across other Type 2-driven immunological disorders.

The safety data available to date, in conjunction with the risk monitoring and mitigation strategies in the study protocol, and the clinical benefit of dupilumab demonstrated in multiple Type 2 indications (AD, asthma, CRSwNP) and case reports and series in PN so far, support a favorable benefit-risk profile for dupilumab.

A risk-benefit statement with respect to the overall development program is provided in the investigator's brochure (IB).

3 OBJECTIVES AND ENDPOINTS

Table 1 - Objectives and endpoints

Objectives	Endpoints
**Primary**	
• To demonstrate the efficacy of dupilumab on itch response in patients with PN, inadequately controlled on topical prescription therapies or when those therapies are not advisable.	• Proportion of participants with improvement (reduction) in worst-itch numeric rating scale (WI-NRS) by ≥4 from baseline to Week 12.
**Secondary**	
• To demonstrate the efficacy of dupilumab on additional itch endpoints in patients with PN, inadequately controlled on topical prescription therapies or when those therapies are not advisable.	• **[Key secondary endpoint]** Proportion of participants with improvement (reduction) in WI-NRS by ≥4 from baseline to Week 24.<br>• Time to onset of effect on pruritus as measured by proportion of participants with an improvement (reduction) in WI-NRS by ≥4 from baseline during the 24-week treatment period.<br>• Change from baseline in WI-NRS at Week 12 and Week 24.<br>• Percent change from baseline in WI-NRS at Week 12 and Week 24.<br>• Percent change from baseline in WI-NRS over time until Week 24.<br>• Proportion of participants with WI-NRS reduction ≥4 over time until Week 24.<br>• Onset of action in change from baseline in WI-NRS (first $p < 0.05$ difference from placebo in the daily WI-NRS that remains significant at subsequent measurements) until Week 12.
• To demonstrate efficacy of dupilumab on skin lesions of PN.	• Proportion of participants with Investigator's Global Assessment 0/1 score for PN-Stage (IGA PN-S) at Week 4, Week 8, Week 12, and Week 24.<br>• Change from baseline in IGA PN-S score at Week 4, Week 8, Week 12, and Week 24.<br>• Proportion of participants with Investigator's Global Assessment 0/1 score for PN-Assess (IGA PN-A) at Week 4, Week 8, Week 12, and Week 24.
• To demonstrate the improvement in health-related quality of life (HRQoL).	• Change from baseline in HRQoL, as measured by Dermatology Life Quality Index (DLQI) to Week 12 and Week 24.
• To evaluate safety outcome measures.	• Percentage of participants experiencing treatment-emergent adverse events (TEAEs) or serious adverse events (SAEs) from baseline through Week 24.
• To evaluate immunogenicity of dupilumab.	• Incidence of treatment-emergent antidrug antibodies (ADA) against dupilumab over time.

Objectives	Endpoints
**Tertiary/exploratory**	
• To demonstrate a reduction in the use of rescue medication and systemic immunosuppressant	• Use of high potency TCS rescue medication through Week 24<br>• Use of systemic immunosuppressant through Week 24, constituting treatment failure
• To evaluate exploratory outcome measures	• Change from baseline in Hospital Anxiety and Depression Scale (HADS) total score to Week 24.<br>• Change from baseline in EQ5D-5L to Week 24.<br>• Change from baseline in Pain numeric rating scale (NRS) to Week 4, Week 8, Week 12, and Week 24.<br>• Change from baseline in Sleep NRS to Week 4, Week 8, Week 12, and Week 24.<br>• Missed school/work days through Week 24.<br>• Incidence of skin-infection TEAEs (excluding herpetic infections) through Week 24.
• To evaluate efficacy of dupilumab on skin lesions using a modified PAS 5-item questionnaire	• Percent change from baseline in number of healed lesions (as part of PAS) at Week 4, Week 8, Week 12, and Week 24.<br>• Change from baseline in exact number of lesions in representative area (as determined from PAS) at Week 4, Week 8, Week 12, and Week 24.<br>• Change from baseline in PAS total score at Week 4, Week 8, Week 12, and Week 24
• To evaluate efficacy of dupilumab on other PN endpoints	• Change from baseline in Patient Global Impression of Severity (PGIS) of PN to Week 4, Week 8, Week 12, and Week 24 and Patient Global Impression of Change (PGIC) of PN at Week 4, Week 8, Week 12, and Week 24.
**PK**	
• To evaluate pharmacokinetic (PK) and pharmacodynamic (PD) outcome measures	• Serum functional dupilumab concentrations and PK profile.<br>• Pharmacodynamic response for selected biomarkers (total IgE).

3.1 APPROPRIATENESS OF MEASUREMENTS

The study endpoints will include both assessments of itch and assessments of the PN lesions. As discussed in Section 2.2, the severe itch observed in this disease, the fact that lesions are typically observed in areas that are within reach for scratching, and the importance of itch-scratch cycles in the pathophysiology of the disease support itch as a reasonable primary endpoint. This choice is in line with the study design publicly available for other drug classes such as NK1-antagonists (20). Amongst standard measurement instruments for evaluation of chronic pruritus, NRS assessing worst itch in the past 24 hours features high reliability and concurrent validity and is a popular choice for all patients due to its simple format (21, 22, 23, 24). The proposed WI-NRS is currently

being used in Phase 3 trial assessing serlopitant efficacy in PN (20). A similar pruritus NRS (peak pruritus) has been validated for AD. Additional validation work covering PN as additional indication will be conducted in the context of the current study. The primary assessment will be a responder analysis based on an at least 4 points improvement of the WI-NRS at Week 12, similar to the assessment widely used in AD studies, and similar to the value proposed for assessment of clinical improvement in the WI-NRS as measures of response in the Phase 2 trial assessing serlopitant efficacy and safety in PN, and currently used in the Phase 3 trial (20). The itch response occurs typically faster than the lesion response but since improvement of both may occur prior to Week 12, assessments will be performed at Week 4 and Week 8. The study of the primary assessment of itch is planned for Week 12 and the lesion assessments are planned for Week 12 and Week 24.

As a secondary endpoint, PN lesions will be assessed by an IGA PN, which is widely used in several dermatological indications (as Physician Global Assessment [PGA] in psoriasis). A modified PAS will be used as an exploratory measure. Both instruments are PN-specific and have been developed by experts in the field. These are the only two disease-specific instruments currently available for the lesion assessment. As an exploratory measure, the PAS will be evaluated. The validation of both instruments for use in PN will be conducted in the context of the Phase 3 trials.

Secondary endpoints will also include HRQoL assessments using the skin-disease specific DLQI in recognition of the significant impacts on quality of life experienced by patients with PN. Even though not validated in PN specifically, the DLQI is well established and widely used to assess HRQoL in patients with skin conditions in clinical trials (25).

4 STUDY DESIGN

4.1 OVERALL DESIGN

This study is a multi-center, 24-week treatment, parallel, double-blind, randomized, placebo-controlled study to evaluate the use of dupilumab in patients with PN inadequately controlled on topical prescription therapies or when those therapies are not advisable.

The study will assess the effect of dupilumab on itch improvement as well as its effect on PN lesions, on patients' health-related quality of life, anxiety and depression, sleep quality and skin pain, and overall health status.

Participants who satisfy the inclusion and exclusion criteria will be randomized (1:1) to one of the following IMP treatment groups:

- Dupilumab 300 mg
- Matched placebo

Duration of study period (per participant)

- Screening period (2-4 weeks)
- Randomized IMP intervention period (24 weeks)
- Follow-up period (12 weeks)

4.2 SCIENTIFIC RATIONALE FOR STUDY DESIGN

This study is a multi-center, 24-week treatment, double-blind, randomized, placebo-controlled study to evaluate the use of dupilumab in patients with PN inadequately controlled on topical prescription therapies or when those therapies are not advisable. Based on mechanistic data (see Section 2), Type 2-inflammation may play a principal role in the pathogenesis of PN in all patients.

The double-blind design intends to mitigate potential bias and placebo control is necessary to assess the net effect of active dupilumab treatment.

The patient population to be studied in this trial is intended to include patients with underlying systemic medical conditions known to be associated with PN. Given that better control of severe manifestations of underlying systemic illnesses can confound the primary itch endpoint, we will enroll 40% to 60% of non-atopic PN patients that have medically well-controlled systemic conditions. Since the literature suggests that approximately 40%-60% of PN patients have a history of atopy (defined as having a medical history of AD, allergic rhinitis/rhinoconjunctivits, asthma, or food allergy), our study design will require that the remaining PN patients have a history or a current diagnosis of atopy. Because active AD can confound the primary endpoint, we will exclude moderate-to-severe AD patients and will institute a cap of up to 10% of the atopic

patient subgroup having active mild AD. It is the goal of this design to mirror the overall PN patient population consistent with real-world estimates of overall prevalence while trying to minimize potential confounders on the primary endpoint.

The primary endpoint, WI-NRS, is a validated measurement of the highest PN burden, ie, extreme itching.

Dupilumab treatment has shown clinical efficacy prior to Week 24 in all Phase 3 trials examined across all indications. In addition, available biomarker data of disease response, including TARC and eotaxin-3, plateau after Week 16 but before Week 24 in prior AD trials, including the 52-week AD study (R668-AD-1224; CHRONOS). The Sponsor considers that a 24-week treatment period is an appropriate duration to observe dupilumab's effect in PN to ensure an adequate evaluation of PN lesions. The duration of the 12-week follow-up period is based on the time expected for drug concentration to reach zero (below the lower limit of quantification) in most patients after the last dose of dupilumab. This follow-up period will allow assessment of the need for chronic treatment with dupilumab for this disease by an assessment of return of PN symptoms post-treatment.

The study will not include adolescents because PN is rare in adolescent patients.

4.3 JUSTIFICATION FOR DOSE

The selected dosing regimen is dupilumab 300 mg Q2W with a loading dose of 600 mg.

This dose regimen is expected to achieve serum concentrations that saturate the target-mediated clearance pathway rapidly with the loading dose and maintain saturation thereafter. The PK of dupilumab is consistent across populations of patients with AD, asthma, CRSwNP, and EoE, as well as healthy volunteers. Furthermore, the sources of variability of dupilumab PK identified in each population and the magnitude of the covariate effects indicate that body weight is the most influential factor, whereas other covariates identified as being statistically significant have no meaningful impact on dupilumab PK. The immunogenicity of dupilumab is also comparable in these populations (see IB).

Dosing regimens of 300 mg Q2W after a loading dose of 600 mg were used in the PN case studies, which resulted in marked improvement in itch within three months and subsequently a notable improvement of the skin lesions (see Section 2).

The 300 mg Q2W dose, with loading dose, is the approved dose for AD and is an approved dose for asthma in the US and the EU.

Therefore, based on the totality of the data, dupilumab 300 mg Q2W (with loading dose) regimen is adequate for patients with PN and a further dose ranging study is not necessary to select a Phase 3 dose regimen.

4.4 END OF STUDY DEFINITION

A participant is considered to have completed the study if he/she has completed all phases of the study including the last visit.

The end of the study is defined as the date of the last visit of the last participant in the study.

5 STUDY POPULATION

Prospective approval of protocol deviations to recruitment and enrollment criteria, also known as protocol waivers or exemptions, is not permitted.

5.1 INCLUSION CRITERIA

Participants are eligible to be included in the study only if all of the following criteria apply:

Age

I 01. Participants must be 18 to 80 years of age, at the time of signing the informed consent.

Type of participant and disease characteristics

Patients with a clinical diagnosis of PN, as defined by all of the following:

I 02. Diagnosed by a dermatologist for at least 3 months before the Screening visit.

I 03. On the WI-NRS ranging from 0 to 10, patients must have an average worst itch score of ≥7 in the 7 days prior to Day 1.

NOTE: Baseline pruritus NRS average score for maximum itch intensity will be determined based on the average of daily NRS scores for maximum intensity (the daily score ranges from 0 to 10) during the 7 days immediately preceding randomization. A minimum of 4 daily scores out of the 7 days is required to calculate the baseline average score. For patients who do not have at least 4 daily scores reported during the 7 days immediately preceding the planned randomization date, randomization should be postponed until this requirement is met, but without exceeding the 28-day maximum duration of the screening period.

I 04. Patients must have a minimum of 20 PN lesions in total on both legs, and/or both arms and/or trunk, at Screening visit and on Day 1.

NOTE: Patients need to have bilaterally symmetrical lesions on the extremities. The presence of lesions on at least 2 body surface areas is required.

I 05. History of failing a 2-week course of medium-to-high potency TCS or when TCS are not medically advisable.

NOTE: Failure is defined as patients who are unable to achieve and/or maintain remission and low disease activity (similar to IGA PN-S score of ≤2 [≤19 nodules]) despite treatment with a daily regimen of medium to higher potency TCS (±TCI as appropriate), applied for at least 14 days, or for the maximum duration recommended by the product prescribing information, whichever is shorter.

I 06. Have applied a stable dose of topical emollient (moisturizer) once or twice daily for at least the 7 consecutive days immediately before Day 1 (NOTE: See exclusion criterion E 20 for limitations regarding emollients; see background treatment with topical emollient in Section 6.1.1).

I 07. Participants must be willing and able to complete a daily symptom eDiary for the duration of the study.

Sex

I 08. Male or Female

Contraceptive use by women should be consistent with local regulations regarding the methods of contraception for those participating in clinical studies.

a) Female participants

- A female participant is eligible to participate if she is not pregnant or breastfeeding, and at least one of the following conditions applies:
  - Is not a WOCBP.

  OR

  - Is a WOCBP and agrees to use an acceptable contraceptive method as described in Appendix 4 (Section 10.4) during the study (at a minimum until 12 weeks after the last dose of study intervention).
  - A WOCBP must have a negative highly sensitive (Section 10.2) pregnancy test (urine or serum as required by local regulations) on Day 1 before the first dose of study intervention.
  - If a urine test cannot be confirmed as negative (eg, an ambiguous result), a serum pregnancy test is required. In such cases, the participant must be excluded from participation if the serum pregnancy result is positive.
- Additional details can be found in Appendix 4 (Section 10.4).
- The investigator is responsible for review of medical history, menstrual history, and recent sexual activity to decrease the risk for inclusion of a woman with an early undetected pregnancy.

Informed Consent

I 09. Capable of giving signed informed consent as described in Appendix 1 (Section 10.1) of the protocol which includes compliance with the requirements and restrictions listed in the informed consent form (ICF) and in this protocol. In countries where legal age of majority is above 18 years, a specific ICF must also be signed by the participant's legally authorized representative.

5.2 EXCLUSION CRITERIA

Participants are excluded from the study if any of the following criteria apply:

Medical conditions

E 01. Presence of skin morbidities other than PN and mild AD that may interfere with the assessment of the study outcomes. Conditions such as, but not limited to, the following: scabies, insect bite, lichen simplex chronicus, psoriasis, acne, folliculitis, habitual picking, lymphomatoid papulosis, chronic actinic dermatitis, dermatitis herpetiformis, sporotrichosis, bullous disease.

NOTE: patients with mild active AD will represent up to 10% of the atopic PN study population.

E 02. PN secondary to medications (eg, opioids, angiotensin converting enzyme [ACE] inhibitors).

E 03. PN secondary to medical conditions such as neuropathy or psychiatric disease (eg, notalgia paresthetica, brachioradial pruritus, neurotic excoriations, obsessive compulsive disorder, delusions of parasitosis, etc).

E 04. Patients with a documented AD severity of moderate to severe within 6 months before or at the screening visit (eg, IGA AD of 3 or 4, eczema area and severity index [EASI] ≥16, scoring atopic dermatitis [SCORAD] ≥25).

E 05. Severe concomitant illness(es) under poor control that, in the investigator's judgment, would adversely affect the patient's participation in the study. Examples include, but are not limited to patients with life expectancy shorter than 1 year, patients with uncontrolled diabetes (hemoglobin A1c ≥9%), patients with cardiovascular conditions (eg, Class III or IV heart failure according to the New York Heart Association classification), hepato-biliary conditions (eg, Child-Pugh Class B or C), neurological conditions (eg, demyelinating diseases), active major autoimmune diseases (eg, lupus, inflammatory bowel disease, rheumatoid arthritis, etc), other severe endocrinological, gastrointestinal, metabolic, pulmonary, or lymphatic diseases. The specific justification for patients excluded under this criterion will be noted in study documents (chart notes, eCRF, etc).

E 06. Severe renal conditions (eg, patients with uremia and/or on dialysis).

E 07. Participants with uncontrolled thyroid disease.

E 08. Active TB or non-tuberculous mycobacterial infection, or a history of incompletely treated TB will be excluded from the study unless it is well documented by a specialist that the participant has been adequately treated and can now start treatment with dupilumab in the medical judgment of the investigator and/or infectious disease specialist. Tuberculosis testing will be performed on a country-by-country basis, according to local guidelines if required by regulatory authorities or ethics boards.

E 09. Diagnosed active endoparasitic infections; suspected or high risk of endoparasitic infection, unless clinical and (if necessary) laboratory assessment have ruled out active infection before randomization.

E 10. Active chronic or acute infection (except HIV infection) requiring treatment with systemic antibiotics, antivirals, antiprotozoals, or antifungals within 2 weeks before the screening visit.

E 11. Known or suspected immunodeficiency, including history of invasive opportunistic infections (eg, TB, histoplasmosis, listeriosis, coccidioidomycosis, pneumocystosis, aspergillosis) despite infection resolution, or otherwise recurrent infections of abnormal frequency or prolonged duration suggesting an immune-compromised status, as judged by the investigator.

E 12. Active malignancy or history of malignancy within 5 years before the baseline visit, except completely treated in situ carcinoma of the cervix, completely treated and resolved non-metastatic squamous or basal cell carcinoma of the skin.

E 13. History of systemic hypersensitivity or anaphylaxis to any biologic therapy, including any excipients.

E 14. Any other medical or psychological condition including relevant laboratory abnormalities at screening that, in the opinion of the investigator, suggest a new and/or insufficiently understood disease, may present an unreasonable risk to the study patient as a result of his/her participation in this clinical trial, may make patient's participation unreliable, or may interfere with study assessments. The specific justification for patients excluded under this criterion will be noted in study documents (chart notes, eCRF, etc).

E 15. History of substance and/or alcohol abuse.

E 16. Planned major surgical procedure during the patient's participation in this study.

Prior/concomitant therapy

E 17. Exposure to another systemic or topical investigative drug (monoclonal antibodies as well as small molecules) within a certain time period prior to Visit 1 (screening), as follows: an interval of less than 6 months or <5 PK half-lives for investigative monoclonal antibodies, whichever is longer, and an interval of less than 30 days or <5 PK half-lives, whichever is longer, for investigative small molecules.

E 18. Having used any of the following treatments within 4 weeks before the screening visit
- Systemic immunosuppressive/immunomodulating drugs (eg, systemic corticosteroids, cyclosporine, mycophenolate-mofetil, interferon gamma, Janus kinase inhibitors, azathioprine, methotrexate, hydroxychloroquine, dapsone, sulfasalazine, colchine, etc).
- Intralesional corticosteroid injections and cryotherapy.
- Phototherapy, including tanning beds.

- Naltrexone or other opioid antagonist.
- Paroxetine, fluvoxamine, or other selective serotonin reuptake inhibitors (SSRIs).
- Serotonin and norepinephrine reuptake inhibitors (SNRIs)
- Amitriptyline or other tricyclic antidepressants.
- Gabapentin, pregabalin, and thalidomide.

E 19. Previous treatment with biologic medicines within the following timeframe:
- Any cell-depleting agents including but not limited to rituximab: within 6 months before the screening visit.
- Omalizumab: within 5 months before screening visit.
- Other immunomodulatory biologics: within 5 half-lives (if known) or 16 weeks before the screening visit, whichever is longer.

E 20. Initiation of treatment with prescription moisturizers or moisturizers containing additives such as ceramide, hyaluronic acid, urea, or filaggrin degradation products during the screening period (patients may continue using stable doses of such moisturizers if initiated before the Screening visit).

E 21. Initiation of treatment with TCS/TCI (any potency) during the screening period or treatment with high potency or superpotent TCS/TCI during the screening period.

E 22. For participants who were on a stable regimen of TCS/TCI at the screening visit:
- Application of TCS/TCI on fewer than 6 days during the 7 days immediately preceding randomization.
- Application of TCS/TCI of incorrect potency within 7 days before Day 1 according to the requirements of Section 6.1.1, ie, low potency if on low potency at screening visit and medium potency if on medium or higher potency at screening visit.

E 23. Treatment with a live (attenuated) vaccine within 4 weeks before the screening visit.

NOTE: For patients who have vaccination with live, attenuated vaccines planned during the course of the study (based on national vaccination schedule/local guidelines), it will be determined, after consultation with a physician, whether the administration of vaccine can be postponed until after the end of study, or preponed to before the start of the study, without compromising the health of the patient:
- Patient for whom administration of live (attenuated) vaccine can be safely postponed would be eligible to enroll into the study.
- Patients who have their vaccination preponed can enroll in the study only after a gap of 4 weeks following administration of the vaccine.

E 24. Planned or anticipated use of any prohibited medications and procedures during screening and study treatment period.

Prior/concurrent clinical study experience

E 25. Participation in prior dupilumab clinical study, prior use of biologics for PN.

Diagnostic assessments

E 26. HIV with CD4+ counts ≤300 cells/µL and/or detectable HIV viral load at the screening visit.

E 27. Participants with any of the following result at screening:

- Positive (or indeterminate) HBs Ag or,
- Positive HBV DNA or,
- Positive HCV RNA.

Other exclusions

E 28. Individuals accommodated in an institution because of regulatory or legal order; prisoners or subjects who are legally institutionalized.

E 29. Any country-related specific regulation that would prevent the subject from entering the study - see Section 10.10 (country-specific requirements).

E 30. Participant not suitable for participation, whatever the reason, as judged by the Investigator, including medical or clinical conditions, or participants potentially at risk of noncompliance to study procedures.

E 31. Participants are dependent on the Sponsor or Investigator (in conjunction with Section 1.61 of the International Council for Harmonisation (ICH) good clinical practice (GCP) Ordinance E6).

E 32. Participants are employees of the clinical study site or other individuals directly involved in the conduct of the study, or immediate family members of such individuals.

E 33. Any specific situation during study implementation/course that may raise ethics considerations.

E 34. Sensitivity to any of the study interventions, or components thereof, or drug or other allergy that, in the opinion of the Investigator, contraindicates participation in the study.

5.3 LIFESTYLE CONSIDERATIONS

Not applicable.

5.4 SCREEN FAILURES

Screen failures are defined as participants who consent to participate in the clinical study but are not subsequently randomly assigned to study intervention/entered in the study. A minimal set of screen failure information is required to ensure transparent reporting of screen failure participants to meet the Consolidated Standards of Reporting Trials (CONSORT) publishing requirements and to respond to queries from regulatory authorities. Minimal information includes demography, screen failure reasons, eligibility criteria, and any SAE.

In the case of technical malfunction of equipment, the patient may be rescreened. Patients who fail the exclusion criteria may be rescreened once during the open screening period of the study. Rescreened participants will be assigned a new participant number versus the one received for the initial screening.

There is no requirement for a waiting period between the screen-failure date and the rescreening date. The interactive response technology (IRT) report will flag rescreened patients. Patients that are rescreened must sign a new consent form and all Visit 1 procedures must be repeated.

If certain dynamic laboratory tests do not meet the eligibility criteria at screening, these laboratory assessments may be repeated, at the discretion of the Investigator, if it is judged to be likely to return to acceptable range for study inclusion within the screening visit window prior to Day 1. There is no need to screen fail such participants if the test finally meets the eligibility criteria.

6 STUDY INTERVENTION

Study intervention is defined as any investigational intervention(s), marketed product(s), placebo, or medical device(s) intended to be administered to a study participant according to the study protocol.

6.1 STUDY INTERVENTION(S) ADMINISTERED

Table 2 - Overview of study interventions administered

**ARM name**	Dupilumab	Placebo
**Intervention name**	Dupilumab 300 mg	Placebo matching dupilumab 300 mg
**Type**	Biological/Vaccine	Other
**Dose formulation**	A 150 mg/mL dupilumab solution in a pre-filled syringe to deliver 300 mg in 2 mL	Identical formulation to the active 300 mg formulation without dupilumab, in a pre-filled syringe to deliver placebo in 2 mL
**Unit dose strength(s)**	300 mg	0 mg (placebo)
**Dosage level(s)**	300 mg every 14 ±3 days after an initial loading dose of 600 mg	0 mg every 14 ±3 days with a loading dose of 0 mg
**Route of administration**	Subcutaneous[a]	Subcutaneous[a]
**IMP and NIMP**	IMP	IMP
**Packaging and labeling**	One glass pre-filled syringe packed in a patient kit box. Both the glass pre-filled syringe and the box will be labeled as required per country requirement.	One glass pre-filled syringe packed in a patient kit box. Both glass pre-filled the syringe and the box will be labeled as required per country requirement

a Subcutaneous injection sites should alternate between the upper thighs, 4 quadrants of the abdomen or the upper arms, so that the same site is not injected twice during consecutive administrations. Injection in the upper arms can only be done by a trained person (parent/legally authorized representative/caregiver trained by Investigator or Delegate) or health care professional but not the participant themselves.

IMP: investigational medicinal product; NIMP: noninvestigational medicinal product.

The Investigator or delegate will train the patient (or caregiver) how to prepare and inject IMP at Visit 2. He/she will inject the first of the two injections. The participant (or caregiver) will perform the second injection under the supervision of the Investigator or delegate. Patient should also be trained by the site staff to recognize potential signs and symptoms of hypersensitivity reaction in order to self-monitor at home for at least 30 minutes (or longer per country specific or local site-specific requirements) following injection. In case of hypersensitivity symptoms the patient should contact healthcare provider/emergency. The training must be documented in the participant's study file.

When the participant has a study visit, the IMP will be administered following clinical procedures and blood collection. Patients should be monitored for at least 30 minutes. The monitoring period may be extended as per country specific or local site-specific requirements.

Between the protocol-scheduled on-site visits, participants are allowed to self-inject IMP at home. Participants who prefer to have a healthcare professional administer the IMP may choose to have injections administered at home by a nurse or at the study site.

Between the protocol-scheduled on-site visits, interim visits may be required for IMP dispensing. As an alternative to these visits, dupilumab 300 mg or matching placebo may be supplied from the site to the participant via a Sponsor-approved courier company where allowed by local regulations and approved by the participant.

6.1.1 Noninvestigational medicinal products

Participants will be required to apply moisturizers (emollients) once or twice daily for at least the 7 consecutive days immediately before Day 1 and continue until Week 36. However, to allow adequate assessment of skin dryness, moisturizers should not be applied on the area(s) of skin designated for such assessments for at least 8 hours before each clinic visit. All types of moisturizers are permitted, but patients may not initiate new treatment with prescription moisturizers or over-the-counter moisturizers containing additives during the screening period or during the intervention period. Patients may continue using stable doses of such moisturizers if initiated before the screening visit.

If participants are on a stable regimen of low to medium potency TCS or TCI at the screening visit, they can continue their topical steroid application once daily without tapering from Screening to Week 24. If specific lesions resolve, the participant can stop applying steroids to those sites but are permitted to continue applying to persistent lesions. If participants are on stable regimens of high potency or superpotent steroids, participants should decrease potency to medium potency TCS and continue to apply daily from screening to Week 24. Occlusion is not allowed from Screening to Week 24.

It is recommended that patients use triamcinolone acetonide 0.1% cream or fluocinolone acetonide 0.025% ointment for medium potency, and hydrocortisone 1% cream for low potency. If rescue with TCS is needed, it is recommended that patients use either betamethasone dipropionate 0.05% optimized ointment for high potency TCS or clobetasol propionate 0.05% cream for super high potency TCS. If patients have tolerance issues with any of these steroids or if they are not commercially available in some countries, they may substitute with products of the same potency from the list provided by the Sponsor.

On areas treated with TCS, moisturizers must be applied once daily only at the time when TCS is not applied (ie, do not use moisturizers and TCS on the same areas at the same time during the day). For example, if TCS are applied in the evening, moisturizers will not be used in the evening on areas treated with TCS, but will be applied to those areas in the morning. On areas not treated with TCS, moisturizers will be applied twice daily - morning and evening.

Rescue therapy is described in Section 6.5.1.

6.2 PREPARATION/HANDLING/STORAGE/ACCOUNTABILITY

6.2.1 Storage and handling

1. The Investigator or designee must confirm appropriate temperature conditions have been maintained during transit for all study intervention received and any discrepancies are reported and resolved before use of the study intervention.
2. Only participants enrolled in the study may receive study intervention and only authorized site staff may supply or administer study intervention. All study intervention must be stored in a secure, environmentally controlled, and monitored (manual or automated) area in accordance with the labeled storage conditions with access limited to the Investigator and authorized site staff.
3. The Investigator, institution, or the head of the medical institution (where applicable) is responsible for study intervention accountability, reconciliation, and record maintenance (ie, receipt, reconciliation, and final disposition records).

6.2.2 Responsibilities

Any quality issue noticed with the receipt or use of an IMP (deficiency in condition, appearance, pertaining documentation, labeling, expiration date, etc) must be promptly notified to the Sponsor. Some deficiencies may be recorded through a complaint procedure (see Section 8.3.6).

A potential defect in the quality of IMP may be subject to initiation of a recall procedure by the Sponsor. In this case, the Investigator will be responsible for promptly addressing any request made by the Sponsor, in order to recall the IMP and eliminate potential hazards.

Under no circumstances will the Investigator supply IMP to a third party (except for direct-to-patient [DTP] shipment, for which a courier company has been approved by the Sponsor), allow the IMP to be used other than as directed by this clinical trial protocol, or dispose of IMP in any other manner.

6.3 MEASURES TO MINIMIZE BIAS: RANDOMIZATION AND BLINDING

All participants will be centrally assigned to randomized study intervention using an IRT. Before the study is initiated, the telephone number and call-in directions for the IVRS and/or the log in information & directions for the IWRS will be provided to each site.

At screening (Visit 1), the Investigator or designee will contact the IRT system to receive the participant number. If a patient who had previously failed screening is approached for re-screening, a new ICF must be signed. In such case, a new patient number will be assigned by IRT.

Methods of assigning patients to treatment group

The randomized intervention kit number list is generated centrally by Sanofi and IMPs are packaged in accordance with this list. The randomization and intervention allocation are performed centrally by an IRT. The IRT generates the participant randomization list and allocates the intervention number and the corresponding intervention kits to the participants according to it.

Participants will be randomized in 1:1 ratio to treatment arms described in Table 2.

Randomization will be stratified by the following factors:

- documented history of atopy (atopic or non-atopic)
  - Atopic: Patients with a physician-documented history of atopic comorbidities defined as AD, allergic rhinitis/rhinoconjunctivits, asthma, or food allergy, or a current diagnosis of at least one of these atopic comorbidities, per investigator judgement. NOTE: enrollment of patients with a current diagnosis of active mild AD will be capped at up to 10% of the participants in this subgroup
  - Non-atopic: Patients without a physician-documented history of atopic comorbidities defined as AD, allergic rhinitis/rhinoconjunctivitis, asthma or food allergy, and without a current diagnosis of at least one of such atopic comorbidities, per investigator judgement
- stable use of TCS/TCI (yes or no)
- country/territory code

A randomized participant is defined as a participant who has been allocated to a randomized intervention regardless whether the intervention kit was used or not (ie, participant registered by the IRT).

A participant cannot be randomized more than once in the study.

Study intervention will be dispensed at the study visits summarized in SoA (see Section 1.3). Returned study intervention should not be re-dispensed to the participants.

Methods of blinding

- Dupilumab 300 mg and placebo matching dupilumab 300 mg will be provided in identically matched 2 mL pre-filled syringes that are visually indistinguishable. Syringes and box will be labeled with a treatment kit number.
- The unblinding plan is described in Section 9.5.2.

Randomization code breaking during the study

The IRT will be programmed with blind-breaking instructions. In case of an emergency, the Investigator has the sole responsibility for determining if unblinding of a participant's treatment assignment is warranted (eg, in case of available antidote). Participant safety must always be the first consideration in making such a determination. If the Investigator decides that unblinding is warranted, the Investigator should make every effort to contact the Sponsor prior to unblinding a participant's treatment assignment unless this could delay emergency treatment of the participant. If a participant's treatment assignment is unblinded, the Sponsor must be notified within 24 hours after breaking the blind. The date and reason that the blind was broken must be recorded in the source documentation and case report form (CRF), as applicable.

VV-CLIN-0649724 1.0

6.4 STUDY INTERVENTION COMPLIANCE

Investigator or his/her delegate must ensure that IMP will be administered to each participant according to the labeling instructions.

IMP accountability:

- Intervention units are returned by the participant at each visit. In case of DTP process, the intervention units can be returned by the carrier (if defined in the contract).
- The Investigator counts the number of remaining kit/pre-filled syringe, and fills in the IMP accountability and inventory forms.
- The Investigator or his/her delegate records the dosing information on the appropriate page(s) of the eCRF.
- The monitor in charge of the study then checks the eCRF data by comparing them with the IMP which he/she has retrieved and source documents.

Participant compliance with study intervention will be assessed at each visit. Compliance will be assessed by counting returned kit/pre-filled syringe. Deviation(s) from the prescribed dosage regimen should be recorded in the eCRF.

6.5 CONCOMITANT THERAPY

Any medication or vaccine (including over-the-counter or prescription medicines, vitamins, and/or herbal supplements) that the participant is receiving at the time of enrollment or receives during the study must be recorded along with:

- Reason for use.
- Dates of administration including start and end dates.
- Dosage information including dose and frequency.

The concomitant use of the following therapies is prohibited during the entire study. Study treatment will need to be discontinued in participants receiving these treatments:

- Systemic immunosuppressive/immunomodulating drugs (eg, systemic corticosteroids, cyclosporine, mycophenolate-mofetil, interferon gamma, Janus kinase inhibitors, azathioprine, methotrexate, hydroxychloroquine, dapsone, sulfasalazine, colchine, etc).
- Other biologics.
- Phototherapy, including tanning beds.
- Naltrexone or other opioid antagonist.
- Gabapentin, pregabalin, and thalidomide.

The concomitant use of the following therapies is also prohibited during the entire study, but study treatment will not need to be discontinued in participants receiving the treatments listed below.

- Intralesional corticosteroid injections and cryotherapy.
- Paroxetine, fluvoxamine, or other SSRIs.
- SNRIs.
- Amitriptyline or other tricyclic antidepressants.

6.5.1 Rescue medicine

The following rescue medications may be used:

- Dermatological preparations of high potency or superpotent TCS and TCI.

Although the use of rescue medications is allowed at any time during the study, the use of rescue medications should be delayed, if possible, for at least 14 days following the initiation of the investigational treatment. The date and time of rescue medication administration as well as the name and dosage regimen of the rescue medication must be recorded in the eCRF.

6.6 DOSE MODIFICATION

No change in IMP dose is allowed.

6.7 INTERVENTION AFTER THE END OF THE STUDY

The Sponsor will not be responsible for intervention after the EOS Visit. Intervention after the EOS Visit will be at the discretion of investigator or treating physician.

7 DISCONTINUATION OF STUDY INTERVENTION AND PARTICIPANT DISCONTINUATION/WITHDRAWAL

7.1 DISCONTINUATION OF STUDY INTERVENTION

7.1.1 Definitive discontinuation

In rare instances, it may be necessary for a participant to permanently discontinue study intervention. If study intervention is permanently discontinued, the participant will remain in the study to be evaluated for safety. See the SoA (Section 1.3) for data to be collected at the time of discontinuation of study intervention.

The participants may withdraw from treatment with the IMP if he or she decides to do so, at any time and irrespective of the reason, or this may be the investigator's decision. All efforts should be made to document the reason(s) for treatment discontinuation and this should be documented in the eCRF.

Participants must be permanently withdrawn from the study treatment for the following reasons:

- At their own request or at the request of their legally authorized representative (legally authorized representative means an individual or judicial or other body authorized under applicable law to consent on behalf of a prospective participant to the patient's participation in the procedure(s) involved in the research).
- If, in the investigator's opinion, continuation in the study would be detrimental to the participant's well-being.
- At the specific request of the Sponsor.
- If they are treated with the specific prohibited medications mentioned in Section 6.5.
- If they miss 2 consecutive IMP doses.
- In the event of a protocol deviation, at the discretion of the investigator or the Sponsor.
- Any code broken at the requested of the investigator.
- Pregnancy.
- Anaphylactic reactions or systemic allergic reactions that are related to IMP and require treatment (see Section 10.8).
- Diagnosis of a malignancy during study, excluding carcinoma in situ of the cervix, or squamous or basal cell carcinoma of the skin.
- Any opportunistic infection or other infections whose nature or course may suggest an immunocompromised status (see Section 10.9).
- Serum ALT >3 ULN and Total Bilirubin >2 ULN (see Section 10.6).
- Serum ALT >5 ULN if baseline ALT ≤2 ULN or ALT >8 ULN if baseline ALT >2 ULN (see Section 10.6).

If a clinically significant finding is identified (including, but not limited to changes from baseline in QT interval corrected using Fridericia's formula [QTcF] after enrollment), the Investigator or qualified designee will determine if the participant can continue in the study and if any change in participant management is needed. This review of the ECG printed at the time of collection must be documented. Any new clinically relevant finding should be reported as an AE.

Any abnormal laboratory value or ECG parameter will be immediately rechecked for confirmation within a reasonable timeframe as assessed by the Investigator before making a decision of definitive discontinuation of the IMP for the concerned participant.

Handling of participants after definitive intervention discontinuation

Participants will be followed-up according to the study procedures specified in this protocol up to the scheduled date of study completion, or up to recovery or stabilization of any AE to be followed-up as specified in this protocol, whichever comes last.

Participants who discontinue the study intervention prematurely (prior to completing the 24-week treatment period) will perform, as soon as possible, the early termination visit with all assessments normally planned for the EOT Visit (Visit 6), to assure a complete clinical assessment in close temporal proximity to the premature termination of study treatment is available.

In addition, and to allow assessment of participant outcomes over the stipulated study period, patients will be asked and encouraged to complete all remaining study treatment visits, and participate in all assessments according to the visit schedule with a ±3 day window. Under exceptional circumstances when a participant cannot come to the site for a scheduled visit, a phone contact can be made. During the phone contact, at least information about AEs, concomitant medication and status of PN should be collected.

All cases of definitive intervention discontinuation must be recorded by the Investigator in the appropriate pages of the eCRF when considered as confirmed.

7.1.2 Temporary discontinuation

Temporary intervention discontinuation may be considered by the Investigator because of suspected AEs.

In addition, the following conditions will be causes for temporary discontinuation of study intervention:

- Infections or infestations that do not respond to medical treatment
- Any laboratory abnormality that meets temporary treatment discontinuation criteria as per Section 10.6

Temporary intervention discontinuation decided by the Investigator corresponds to more than 1 dose not administered to the participant. Following a temporary interruption or missed dose, the treatment should be reinitiated at the next scheduled administration, maintaining the planned dose. If 2 consecutive IMP doses have been missed, the study treatment will be definitively discontinued (see Section 7.1.1).

7.1.2.1 Rechallenge

Reinitiation of intervention with the IMP will be done under close and appropriate clinical/and or laboratory monitoring once the Investigator will have considered according to his/her best medical judgment that the responsibility of the IMP(s) in the occurrence of the concerned event was unlikely and if the selection criteria for the study are still met (refer to Section 5).

7.2 PARTICIPANT DISCONTINUATION/WITHDRAWAL FROM THE STUDY

- A participant may withdraw from the study at any time at his/her own request, or may be withdrawn at any time at the discretion of the Investigator for safety, behavioral, compliance, or administrative reasons. This is expected to be uncommon.
- At the time of discontinuing from the study, if possible, an early discontinuation visit should be conducted, as shown in the SoA (Section 1.3). See SoA for data to be collected at the time of study discontinuation and follow-up and for any further evaluations that need to be completed.
- The participant will be permanently discontinued both from the study intervention and from the study at that time.
- If the participant withdraws consent for disclosure of future information, the Sponsor may retain and continue to use any data collected before such a withdrawal of consent.
- If a participant withdraws from the study, he/she may request destruction of any samples taken and not tested, and the Investigator must document this in the site study records.

If participants no longer wish to take the IMP, they will be encouraged to remain in the study.

The Investigators should discuss with them key visits to attend. The value of all their study data collected during their continued involvement will be emphasized as important to the public health value of the study.

Participants who withdraw from the study intervention should be explicitly asked about the contribution of possible AEs to their decision, and any AE information elicited must be documented.

All study withdrawals should be recorded by the Investigator in the appropriate screens of the eCRF and in the participant's medical records. In the medical record, at least the date of the withdrawal and the reason should be documented.

In addition, a participant may withdraw his/her consent to stop participating in the study. Withdrawal of consent for intervention should be distinguished from withdrawal of consent for follow-up visits and from withdrawal of consent for non-participant contact follow-up, eg, medical record checks. The site should document any case of withdrawal of consent.

Participants who have withdrawn from the study cannot be rerandomized (treated) in the study. Their inclusion and intervention numbers must not be reused.

7.3 LOST TO FOLLOW UP

A participant will be considered lost to follow-up if he or she repeatedly fails to return for scheduled visits and is unable to be contacted by the study site.

The following actions must be taken if a participant fails to return to the clinic for a required study visit:

- The site must attempt to contact the participant and reschedule the missed visit as soon as possible and counsel the participant on the importance of maintaining the assigned visit schedule and ascertain whether or not the participant wishes to and/or should continue in the study.
- Before a participant is deemed lost to follow up, the Investigator or designee must make every effort to regain contact with the participant (where possible, 3 telephone calls and, if necessary, a certified letter to the participant's last known mailing address or local equivalent methods). These contact attempts should be documented in the participant's medical record.
- Should the participant continue to be unreachable, he/she will be considered to have withdrawn from the study.
- Discontinuation of specific sites or of the study as a whole are handled as part of Section 10.1.

8 STUDY ASSESSMENTS AND PROCEDURES

- Study procedures and their timing are summarized in the SoA (see Section 1.3). Protocol waivers or exemptions are not allowed.
- Adherence to the study design requirements, including those specified in the SoA (see Section 1.3), is essential and required for study conduct.
- All screening evaluations must be completed and reviewed to confirm that potential participants meet all eligibility criteria. The Investigator will maintain a screening log to record details of all participants screened and to confirm eligibility or record reasons for screening failure, as applicable.
- Procedures conducted as part of the participant's routine clinical management (eg, blood count) and obtained before signing of the ICF may be utilized for screening or baseline purposes provided the procedures met the protocol-specified criteria and were performed within the time frame defined in the SoA (see Section 1.3).
- Patient-Reported Outcome questionnaires including NRS should be completed by the participants before the consultation and/or clinical tests, in a quiet place. The questionnaires should be completed by the participants themselves, independently from their physician, the study nurse or any other medical personnel and without any help from friends or relatives.

8.1 EFFICACY ASSESSMENTS

Efficacy data will be collected via electronic devices.

The e-diary is used for daily recording of participant's answers to the WI-NRS, pain-NRS, and sleep-NRS questionnaires. This device will be dispensed at screening visit (Visit 1), including instructions for use. Participants will be instructed on the use of the device.

Recorded information will be downloaded from this device daily. At EOS Visit, the e-diary will be downloaded and returned to the site.

On regular basis, the site staff should review on vendor's website the information downloaded from participants' e-diary. They should particularly check status of the disease reviewing WI-NRS, as well as compliance to background therapy and overall e-diary compliance. The site should follow up with the participant as appropriate.

Participants will fill in the DLQI, HADS, EQ-5D-5L, PGIC, PGIS, and missed school/work days questionnaires during their site visit on a tablet that will be provided to the site. This device is kept at the site during the study.

8.1.1 Worst-itch numeric rating scale

Worst-itch numerical rating scale (WI-NRS) is a PRO comprised of a single item rated on a scale from 0 (“No itch”) to 10 (“Worst imaginable itch”). Participants are asked to rate the intensity of their worst pruritus (itch) over the past 24 hours using this scale.

Participants will complete the WI-NRS daily according to the SoA (see Section 1.3). The assessment tool is provided in Section 10.7.1.

8.1.2 Investigator’s global assessment for prurigo nodularis

Investigator’s global assessment for prurigo nodularis (IGA PN) is a clinician-reported outcome (ClinRO) that allows clinicians to assess the activity of PN (IGA PN-A) using a 5-point scale from 0 (clear) to 4 (severe); and the stage of the disease (IGA PN-S) using a 5-point scale from 0 (clear) to 4 (severe).

Clinicians will complete the IGA PN as described in the SoA (see Section 1.3). The assessment tool is provided in Section 10.7.2.

8.1.3 Prurigo activity score

The PAS is a ClinRO measurement. The original PAS questionnaire Version 0.9 consists of 7 items, developed by expert clinicians in PN (26). The items of the PAS evaluate the pruriginous lesions in terms of:

- Type (visible lesions: Item 1a; predominant lesions: Item 1b);
- Estimated number (Item 2);
- Distribution (Item 3, 4);
- Size (biggest lesion: Item 6a; representative lesion: Item 6b).

Other items evaluate the representative body area and exact number of lesions (Item 5), the activity in terms of percentage of pruriginous lesions with excoriations/crusts on top (reflecting active scratching; Item 7a) and the percentage of healed pruriginous lesions (reflecting healing of chronic prurigo; Item 7b).

A 5-item simplified version of the PAS will be used in the current study. In particular, Item 3 (lesion distribution) and Item 6 (lesion monitoring) of the original PAS were removed, and the response options slightly modified and refined.

Clinicians will complete the screening/baseline version at screening and baseline visits, and will complete the follow-up version of the modified PAS at the other visits, as reported in the SoA (see Section 1.3). The assessment tool is provided in Section 10.7.3.

8.1.4 Dermatology life quality index

The DLQI is a PRO developed to measure dermatology-specific HRQoL in adult patients (27). The instrument comprises 10 items assessing the impact of skin disease on patients' HRQoL over the previous week. The items cover symptoms, leisure activities, work/school or holiday time, personal relationships including intimate, the side effects of treatment, and emotional reactions to having a skin disease. It is a validated questionnaire used in clinical practice and clinical trials (25). Response scale is a 4-point Likert scale (0 = "not at all" and 3 = "very much") for nine items. The remaining one item about work/studying asks whether work/study has been prevented and then (if "No") to what degree the skin condition has been a problem at work/study; the item is rated on a 3-point Likert scale ("Not at all" to "A lot"). Overall scoring ranges from 0 to 30, with a high score indicative of a poor HRQoL.

Participants will complete the questionnaire at time points according to SoA (see Section 1.3). The assessment tool is provided in Section 10.7.4.

8.1.5 Pain and sleep numeric rating scales

Participants will be asked to rate their worst skin pain in the past 24 hours using a 0 to 10 NRS, with 0 = No pain to 10 = Worst pain possible.

In addition, participants will be asked to rate their sleep quality on their past night upon awakening, using a 0 to 10 NRS, with 0 = Worst possible sleep and 10 = Best possible sleep.

Participants will complete the skin pain NRS and sleep quality NRS once a day.

The assessment tools are provided in Section 10.7.7 and Section 10.7.8.

8.1.6 Hospital anxiety and depression scale

The HADS is a PRO instrument for screening anxiety and depression in non-psychiatric populations; repeated administration also provides information about changes to a patient's emotional state (28, 29). The HADS consists of 14 items, 7 each for anxiety and depression symptoms; possible scores range from 0 to 21 for each subscale. The following cut-off scores are recommended for both subscales:

- 0 to 7: normal
- 8 to 10: borderline abnormal (borderline case)
- 11 to 21: abnormal

Participants will complete the questionnaire as described in the SoA (see Section 1.3). The assessment tool is provided in Section 10.7.5.

8.1.7 Patient Global Impression of Change of disease and Patient Global Impression of Severity

The PGIC is a one-item questionnaire that asks patients to provide the overall self-assessment of change in their PN overall on a 7-point scale, compared to just before patient started taking the study injection. Response choices are: 0 = “Very much better”, 1 = “Moderately better”, 2 = “A little better”, 3 = “No change”, 4 = “A little worse”, 5 = “Moderately worse”, 6 = “Very much worse”.

The PGIS is a one-item questionnaire that asks patients to provide the overall self-assessment of their disease severity on a 4-point scale for the past week. Response choices are: 1 = “none”, 2 = “Mild”, 3 = “Moderate”, 4 = “Severe”.

Participants will complete the 2 items as described in the SoA (see Section 1.3). The items are provided in Section 10.7.9 and Section 10.7.10.

8.1.8 Euroqol 5 dimensions questionnaire

The Euroqol-5 dimensions (EQ-5D) is a standardized PRO measure of health status developed by the EuroQOL Group in order to provide a simple, generic measure of health for clinical and economic appraisal (30). The EQ-5D consists of 2 parts: the descriptive system and the EQ visual analog scale (VAS). The EQ-5D descriptive system comprises the following 5 dimensions: mobility, self-care, usual activities, pain/discomfort, and anxiety/depression. Each dimension has 5 levels of perceived problems: “no problem”, “slight problems”, “moderate problems”, “severe problems” and “inability to do the activity”. The respondent is asked to indicate his/her health state by ticking (or placing a cross) in the box against the most appropriate statement in each of the 5 dimensions; this results in a 1-digit number expressing the level for that dimension. The digits for 5 dimensions can be combined in a 5-digit number describing the respondent’s health state. The EQ VAS records the respondent’s self-rated health on a vertical VAS where the endpoints are labeled “best imaginable health state (100)” and “worst imaginable health state (0)”. This information can be used as a quantitative measure of health outcome as judged by the individual respondents.

Participants will complete the questionnaire as described in the SoA (see Section 1.3). The assessment tool is provided in Section 10.7.6.

8.1.9 Missed school/work days

Participants who are employed or enrolled in school will be asked to report the number of sick leave/missed school days since the last study assessment.

Participants will complete the questionnaire as described in the SoA (see Section 1.3). The assessment tool is provided in Section 10.7.11.

8.1.10 Photography

Participants in selected sites who decide to participate in this substudy need to provide separate consent as described in the SoA (see Section 1.3). One or several lesions will be photographed at baseline. The same lesions will be photographed at subsequent visits to evaluate their progression. The detailed information will be provided in the photography user guide.

8.2 SAFETY ASSESSMENTS

Planned time points for all safety assessments are provided in the SoA (see Section 1.3).

8.2.1 Physical examinations

- A complete physical examination will include, at a minimum, assessments of the skin (full body skin exam), nasal cavities, eyes, ears, respiratory, cardiovascular, gastrointestinal, neurological, lymphatic, and musculoskeletal systems.
- Participants must be disrobed and provided with a hospital gown before the skin examination.
- Investigators should pay special attention to clinical signs related to previous serious illnesses.
- Any new finding or worsening of previous finding should be reported as a new AE

8.2.2 Vital signs

- Vital signs will be measured in a semi-supine or sitting position after 5 minutes rest and will include body temperature, SBP and DBP, and pulse and respiratory rate. Blood pressure and pulse measurements should be assessed using the same arm with a completely automated device. Manual techniques will be used only if an automated device is not available.
- Body weight (kg) will be measured at screening (Visit 1), EOT, and EOS visits. Height will be measured at screening visit (Visit 1). Height and weight should be measured with indoor clothing but without shoes.

8.2.3 Electrocardiograms

- Single 12-lead ECG will be obtained as outlined in the SoA (see Section 1.3) using an ECG machine that automatically calculates the heart rate and measures PR, QRS, QT, and QTcF intervals. Refer to Section 7 for QTcF withdrawal criteria and any additional QTcF readings that may be necessary. The ECG should be recorded after 10 minutes of rest in the supine position.

8.2.4 Clinical safety laboratory assessments

- See Appendix 2 (Section 10.2) for the list of clinical laboratory tests to be performed and to the SoA (see Section 1.3) for the timing and frequency.

- The Investigator must review the laboratory report, document this review, and record any clinically relevant changes occurring during the study in the AE section of the CRF. The laboratory reports must be filed with the source documents. Clinically significant abnormal laboratory findings are those which are not associated with the underlying disease, unless judged by the Investigator to be more severe than expected for the participant's condition.
- All laboratory tests with values considered clinically significantly abnormal during participation in the study or within 12 weeks after the last dose of study intervention should be repeated until the values return to normal or baseline or are no longer considered clinically significant by the Investigator or medical monitor.
  - If such values do not return to normal/baseline within a period of time judged reasonable by the Investigator, the etiology should be identified and the Sponsor notified.
  - All protocol-required laboratory assessments, as defined in Appendix 2 (Section 10.2), must be conducted in accordance with the laboratory manual and the SoA (see Section 1.3).
  - If laboratory values from non-protocol specified laboratory assessments performed at the institution’s local laboratory require a change in participant management or are considered clinically significant by the Investigator (eg, SAE or AE or dose modification), then the results must be recorded in the CRF.

8.3 ADVERSE EVENTS AND SERIOUS ADVERSE EVENTS

Adverse event of special interest

An AESI is an AE (serious or nonserious) of scientific and medical concern specific to the Sponsor’s product or program, for which ongoing monitoring and immediate notification by the Investigator to the Sponsor is required. Such events may require further investigation in order to characterize and understand them. Adverse events of special interest may be added, modified or removed during a study by protocol amendment.

For these AESIs, the Sponsor will be informed immediately (ie, within 24 hours), per SAE notification described in Section 10.3, even if not fulfilling a seriousness criterion, using the corresponding pages in the CRF (to be sent) or screens in the eCRF. If an SAE or any AESI of anaphylactic reactions or systemic allergic reactions that are related to IMP and require treatment, or severe injection site reactions lasting longer than 24 hours, occurs in a participant, blood samples should be collected for determination of functional dupilumab concentration, and ADA assessment at or near the onset and completion of the occurrence of the event, if possible.

- Anaphylactic reactions
- Systemic hypersensitivity reactions
- Helminthic infections
- Any type of conjunctivitis or blepharitis (severe or serious)
- Keratitis

- Clinically symptomatic eosinophilia (or eosinophilia associated with clinical symptoms)
- Pregnancy of a female subject entered in a study as well as pregnancy occurring in a female partner of a male subject entered in a study with IMP/NIMP;
  - Pregnancy occurring in a female participant entered in the clinical trial or in a female partner of a male participant entered in the clinical trial. It will be qualified as an SAE only if it fulfills one of the seriousness criteria.
  - In the event of pregnancy in a female participant, IMP should be discontinued.
  - Follow-up of the pregnancy in a female participant or in a female partner of a male participant is mandatory until the outcome has been determined.
  - Abnormal pregnancy outcomes (eg, spontaneous abortion, fetal death, stillbirth, congenital anomalies, ectopic pregnancy) are considered SAEs.
- Significant ALT elevation
  - ALT >5 x ULN in participants with baseline ALT ≤2 x ULN;

    OR
  - ALT >8 x ULN if baseline ALT >2 x ULN.
- Symptomatic overdose (serious or nonserious) with IMP/NIMP
  - An overdose (accidental or intentional) with the IMP is an event suspected by the Investigator or spontaneously notified by the participant and defined as at least twice the intended dose during an interval of less than 11 days. The circumstances (ie, accidental or intentional) should be clearly specified in the verbatim and symptoms, if any, entered on separate AE forms.
  - An overdose (accidental or intentional) with any NIMP is an event suspected by the Investigator or spontaneously notified by the participant and defined as at least twice the maximum prescribed daily dose, within the intended therapeutic interval. The circumstances (ie, accidental or intentional) should be clearly specified in the overdose form.

The definitions of an AE or SAE can be found in Appendix 3 (Section 10.3).

Adverse events will be reported by the participant (or, when appropriate, by a caregiver, surrogate, or the participant's legally authorized representative).

The Investigator and any qualified designees are responsible for detecting, documenting, and recording events that meet the definition of an AE or SAE and remain responsible for following up AEs that are serious, considered related to the study intervention or study procedures, or that caused the participant to discontinue the study intervention (see Section 7).

8.3.1 Time period and frequency for collecting AE and SAE information

All AEs, serious or nonserious, will be collected from the signing of the ICF until the EOS visit at the time points specified in the SoA (Section 1.3).

All SAEs and AESI will be recorded and reported to the Sponsor or designee immediately and under no circumstance should this exceed 24 hours, as indicated in Appendix 3 (Section 10.3). The Investigator will submit any updated SAE data to the Sponsor within 24 hours of it being available.

Investigators are not obligated to actively seek AE or SAE after conclusion of the study participation. However, if the Investigator learns of any SAE, including a death, at any time after a participant has been discharged from the study, and he/she considers the event to be reasonably related to the study intervention or study participation, the Investigator must promptly notify the Sponsor.

8.3.2 Method of detecting AEs and SAEs

The method of recording, evaluating, and assessing causality of AE and SAE and the procedures for completing and transmitting SAE reports are provided in Appendix 3 (Section 10.3).

Care will be taken not to introduce bias when detecting AEs and/or SAEs. Open-ended and non-leading verbal questioning of the participant is the preferred method to inquire about AE occurrences.

8.3.3 Follow-up of AEs and SAEs

After the initial AE/AESI/SAE report, the Investigator is required to proactively follow each participant at subsequent visits/contacts. At the pre-specified study end-date, all SAEs and nonserious AESI (as defined in Section 8.3) will be followed until resolution, stabilization, the event is otherwise explained, or the participant is lost to follow-up (as defined in Section 7.3). Further information on follow-up procedures is given in Appendix 3 (Section 10.3).

8.3.4 Regulatory reporting requirements for SAEs

- Prompt notification by the Investigator to the Sponsor of a SAE is essential so that legal obligations and ethical responsibilities towards the safety of participants and the safety of a study intervention under clinical investigation are met.
- The Sponsor has a legal responsibility to notify both the local regulatory authority and other regulatory agencies about the safety of a study intervention under clinical investigation. The Sponsor will comply with country-specific regulatory requirements relating to safety reporting to the regulatory authority, Institutional Review Boards (IRB)/Independent Ethics Committees (IEC), and Investigators.
- Investigator safety reports must be prepared for suspected unexpected serious adverse reactions (SUSAR) according to local regulatory requirements and Sponsor policy and forwarded to Investigators as necessary.
- Adverse events that are considered expected will be specified in the reference safety information.
- An Investigator who receives an Investigator safety report describing a SAE or other specific safety information (eg, summary or listing of SAEs) from the Sponsor will review and then file it along with the IB and will notify the IRB/IEC, if appropriate according to local requirements.

8.3.5 Pregnancy

- Details of all pregnancies in female participants and female partners of male participants will be collected after the start of study intervention and until the outcome has been determined.
- If a pregnancy is reported, the Investigator should inform the Sponsor within 24 hours of learning of the pregnancy and should follow the procedures outlined in Appendix 4 (Section 10.4).
- Abnormal pregnancy outcomes (eg, spontaneous abortion, fetal death, stillbirth, congenital anomalies, ectopic pregnancy) are considered SAEs.

8.3.6 Guidelines for reporting product complaints

Any defect in the IMP/NIMP/device must be reported as soon as possible by the Investigator to the monitoring team that will complete a product complaint form within required timelines.

Appropriate information (eg, samples, labels or documents like pictures or photocopies) related to product identification and to the potential deficiencies may need to be gathered. The Investigator will assess whether or not the quality issue has to be reported together with an AE or SAE.

8.4 TREATMENT OF OVERDOSE

Overdose is an AESI (defined in Section 8.3). No antidote is available for dupilumab. Sponsor does not recommend specific treatment for an overdose.

In the event of an overdose, the Investigator/treating physician should:

1. Provide symptomatic care.
2. Contact the Medical Monitor immediately.
3. Closely monitor the participant for any AE/SAE and laboratory abnormalities until dupilumab can no longer be detected systemically.
4. Obtain a plasma sample for PK analysis as soon as possible if requested by the Medical Monitor (determined on a case-by-case basis).
5. Document appropriately in the CRF.

Decisions regarding dose interruptions will be made by the Investigator in consultation with the Medical Monitor based on the clinical evaluation of the participant.

8.5 PHARMACOKINETICS

8.5.1 Systemic drug concentration and antidrug antibodies

8.5.1.1 Sampling time

Blood samples will be collected for determination of functional dupilumab and anti-dupilumab antibodies in serum as specified in the SoA (Section 1.3). Special procedures for collection, storage, and shipping of serum are described in separate operational manuals. The date of collection should be recorded in the eCRF.

8.5.1.2 Handling procedures

Special procedures for collection, storage, and shipping of serum are described in separate operational manuals. An overview of handling procedure for samples used in the determination of systemic drug concentration and ADA is provided in Table 3.

Table 3 - Summary of handling procedures

Sample type	Functional dupilumab	Anti-dupilumab antibody
Matrix	Serum	Serum
Blood sample volume	5 mL	5 mL
Anticoagulant	None	None
Blood handling procedures	See Operational Manual	See Operational Manual
Serum aliquot split	2 aliquots	2 aliquots
Storage conditions	<6 months: below -20°C<br><24 months: below -80°C (preferred)	<6 months: below -20°C<br><24 months: below -80°C (preferred)
Serum shipment condition	In dry ice	In dry ice

8.5.1.3 Bioanalytic method

Serum PK and ADA samples will be assayed using validated methods as described in Table 4.

Table 4 - Summary of bioanalytical methods for functional dupilumab and anti-dupilumab antibodies

[redacted]

Note: In the event of any SAE, any AE of severe injection site reaction lasting longer than 24 hours, or any AESI of anaphylactic reaction or systemic allergic reaction that is related to IMP and require treatment, PK and ADA samples will be collected at or near the onset of the event for

any additional analysis if required or for archival purposes. The exact date and time of sample collection must be recorded and entered into the database by the central laboratory. An unscheduled systemic drug concentration page in the eCRF must be completed as well. If necessary for safety monitoring, additional PK and ADA samples may be collected after the EOS Visit until resolution of AE.

Specifically for PK, any changes in the timing or addition of time points for any planned study assessments must be documented and approved by the relevant study team member and then archived in the Sponsor and site study files, but will not constitute a protocol amendment. The IRB/IEC will be informed of any safety issues that require alteration of the safety monitoring scheme or amendment of the ICF.

8.6 PHARMACODYNAMICS

See Section 8.8 for IgE measurement. No other PD parameters will be evaluated in this study.

8.7 GENETICS

For those participants who consent to the optional pharmacogenetic/pharmacogenomic sample collection section of the ICF, blood samples for exploratory genetic analysis of DNA or RNA will be collected and stored for possible future use (see Section 10.5). Participation is optional. Participants who do not wish to participate in the genetic research may still participate in the study.

In the event of DNA extraction failure, a replacement genetic blood sample may be requested from the participant.

Details on processes for collection and shipment and destruction of these samples can be found in the laboratory manual.

8.8 BIOMARKERS

- Venous blood samples will be collected for measurement of total serum IgE. Total IgE will be measured using validated quantitative methods.
- Optional samples for biomarker research that should be collected from participants in the study with their consent are the following:
  - Serum and plasma: samples will be collected and stored for possible future analysis of potential biomarkers of drug response, disease activity, safety, and Type 2 inflammation.
  - Skin biopsy (sub-study): samples will be taken from lesion and non-lesion skin using punch biopsy; gene expression and immunohistological analyses may be used to investigate drug response, disease activity, and Type 2 inflammation.

Plasma, serum and skin biopsy samples may be stored for a maximum of 5 years, and DNA or RNA samples for 15 years (or according to local regulations) following the last participant's last visit for the study at a facility selected by the Sponsor.

8.9 MEDICAL RESOURCE UTILIZATION AND HEALTH ECONOMICS

Number of missed school/work days collection is described in Section 8.1.9.

9 STATISTICAL CONSIDERATIONS

9.1 STATISTICAL HYPOTHESES

The statistical hypotheses for comparing dupilumab to placebo on the primary endpoint of the proportion of participants with WI-NRS reduction of ≥4 (minimally important difference) at Week 12 are as follows:

- Null hypothesis H0: No treatment difference between dupilumab and placebo
- Alternative hypothesis H1: There is a treatment difference between dupilumab and placebo

The statistical hypotheses to be tested on secondary endpoints can be specified similarly to those on the primary endpoint.

9.2 SAMPLE SIZE DETERMINATION

The primary endpoint is the proportion of participants with WI-NRS reduction of ≥4 from baseline to Week 12. By assuming the response rate is [redacted]% and [redacted]% in placebo and dupilumab respectively, 56 participants/arm will provide 90% power to detect the difference of [redacted]% between dupilumab and placebo with Fisher exact test at 2-sided level of 0.05. Assuming 15% drop out during the 12 weeks of treatment, the target is to randomize 75 participants/arm with a cap of up to 10% of participants in the atopic population having active mild AD.

Approximately 150 participants will be randomized 1:1. This corresponds to approximately 75 participants that will be randomly assigned to each intervention arm.

The sample size calculation was performed using SAS 9.4 power procedure. The assumptions were based on the effect of dupilumab versus placebo in WI-NRS reduction ≥4 at Week 16 observed in patients with moderate to severe AD as seen in studies of AD-1334 (Solo1) and AD-1416 (Solo2).

Participants will be randomized to dupilumab or placebo in 1:1 ratio with stratification factors of documented history of atopy (atopic or non-atopic, see definition in Section 6.3), stable use of TCS/TCI (yes or no), and country/territory code. Both the atopic and the non-atopic PN population will be capped at 60% of the total enrolled population.

9.3 POPULATIONS FOR ANALYSES

For purposes of analysis, the following populations are defined (Table 5):

Table 5 - Populations for analyses

Population	Description
Screened	All participants who sign the ICF.
Randomized	The randomized population includes all participants with a treatment kit number allocated and recorded in the IRT database, and regardless of whether the treatment kit was used or not.<br>Participants treated without being randomized will not be considered randomized and will not be included in any efficacy population.
Intent-to-treat (ITT)	All randomized participants analyzed according to the treatment group allocated by randomization regardless if treatment kit is used or not
Efficacy	The ITT population
Safety	All participants randomly assigned to study intervention and who take at least 1 dose of study intervention. Participants will be analyzed according to the intervention they actually received.<br>Randomized participants for whom it is unclear whether they took the study medication will be included in the safety population as randomized.<br>For participants who accidentally receive different treatment from the planned, the actual intervention allocation for as-treated analysis will be the dupilumab group.<br>The PD analyses will be performed on the safety population.
Pharmacokinetic (PK)	The PK population includes all participants in the safety population with at least one non-missing result for functional dupilumab concentration in serum after first dose of the study treatment. Participants will be analyzed according to the intervention actually received.
Antidrug antibody (ADA)	ADA population includes all participants in the safety population who have at least one non-missing ADA result after first dose of the study treatment. Participants will be analyzed according to the intervention actually received.

ADA: antidrug antibody; ICF: informed consent form; ITT: intent to treat; PD: pharmacodynamics: PK: pharmacokinetic.

9.4 STATISTICAL ANALYSES

The SAP will be developed and finalized before primary database lock and will describe the participant populations to be included in the analyses, and procedures for accounting for missing, unused, and spurious data. This section is a summary of the planned statistical analyses of the primary and secondary endpoints.

9.4.1 Efficacy analyses

The multiplicity procedure is proposed to control the overall Type-I error rate for testing the primary endpoint and the key secondary endpoints. Detailed hierarchical testing procedure will be defined in the study SAP. The study is considered positive when the primary endpoint achieves statistical significance with two-sided significance level 0.05.

Table 6 - Efficacy analyses

Endpoint	Statistical analysis methods
**Primary**:	
Proportion of participants with improvement (reduction) in WI-NRS by ≥4 from baseline to Week 12	The primary analysis will be conducted by using CMH test stratifying by stratification factors of documented history of atopy (atopic or non-atopic), stable use of TCS/TCI (yes or no), and region (countries combined).<br>For participants discontinuing the study treatment before Week 12, their off-study treatment values measured up to Week 12 will be included in the analysis. Participants taking selected prohibited medications and/or rescue medications (details of selection will be provided in the SAP) prior to Week 12 or have missing data at Week 12 will be considered non-responders.<br>*Sensitivity analyses:*<br>The data collected after taking selected prohibited medications and/or rescue medications will not be censored and included in the sensitivity analysis to evaluate the robustness of the primary analysis results with respect to the method of handling data while taking selected prohibited medications. Details of the sensitivity analyses will be provided in the SAP.<br>*Subgroup analyses:*<br>To assess the consistency in treatment effects across different subgroup levels, subgroup analyses will be performed for the primary efficacy endpoint with respect to documented history of atopy (atopic or non-atopic), age group, gender, region, and other factors that will be specified in the SAP. A subgroup analysis will be performed excluding participants with a current diagnosis of AD.
**Secondary:**	
**[Key secondary endpoint]** Proportion of participants with improvement (reduction) in WI-NRS by ≥4 from baseline to Week 24.<br>Proportion of participants with WI-NRS reduction ≥4 over time until Week 24<br>Proportion of participants with IGA PN-S 0/1 score at Week 4, Week 8, Week 12, and Week 24.<br>Proportion of participants with IGA PN-A 0/1 score at Week 4, Week 8, Week 12, and Week 24.	Secondary efficacy endpoints that measure binary responses will be analyzed in the same fashion as the primary endpoint.
Time to onset of effect on pruritus as measured by proportion of participants with improvement (reduction) in WI-NRS by ≥4 from baseline during the 24-week treatment period.	This time-to-event endpoint will be analyzed using the Cox proportional hazards model, including treatment, stratification factors (countries combined to region). The hazards ratio, its 95% confidence interval and p-value will be reported. Kaplan-Meier curves will be also provided.

Change from baseline in WI-NRS at Week 12 and Week 24.<br>Percent change from baseline in WI-NRS at Week 12 and Week 24.<br>Percent change from baseline in WI-NRS over time until Week 24.<br>Change from baseline in IGA PN-S score at Week 4, Week 8, Week 12, and Week 24.<br>Change from baseline in HRQoL, as measured by DLQI to Week 12 and Week 24<br>Onset of action in change from baseline in WI-NRS (first p <0.05 difference from placebo in the daily WI-NRS that remains significant at subsequent measurements) until Week 12.	Continuous secondary efficacy endpoints will be analyzed using a hybrid method of the WOCF and multiple imputations.<br>Data of participants taking selected prohibited medications and/or rescue medications will be set to missing after the medication usage, and the worst postbaseline value on or before the time of the medication usage will be used to impute missing endpoint value (for participants whose postbaseline values are all missing, the baseline will be used to impute).<br>Participants who discontinue the treatment prematurely are encouraged to follow the planned clinical visits and in those participants who did not take the selected prohibited medications and/or rescue medications, all data collected after treatment discontinuation will be used in the analysis. For these participants, missing data may still happen despite all efforts have been tried to collect the data after treatment discontinuation. For participants who discontinue due to lack of efficacy, all data collected after discontinuation will be used in the analysis, and a WOCF approach will be used to impute missing data if needed. For participants who discontinued not due to lack of efficacy, a multiple imputation approach will be used to impute missing endpoint value, and this multiple imputation will use all participants excluding patients who have taken the selected prohibited medications and/or rescue medications prior to timepoint of endpoint of interest and excluding participants who discontinue due to lack of efficacy.<br>Each of the imputed complete data will be analyzed by fitting an ANCOVA model with treatment group, stratification factors of documented history of atopy (atopic or non-atopic), stable use of TCS/TCI (yes or no), and region (countries combined), and relevant baseline measurement as covariates included in the model. Statistical inference obtained from all imputed data will be combined using Rubin's rule. Descriptive statistics including number of subjects, mean, standard error, and least squares (LS) mean change and standard error will be provided. In addition, difference of the dupilumab group against placebo in LS means and the corresponding 95% confidence interval will be provided along with the p-values.<br>Detailed analyses will be described in the SAP finalized before database lock.
**Tertiary/exploratory**	Will be described in the SAP finalized before database lock.

ANCOVA: analysis of covariance; CMH: Cochran-Mantel-Haenszel; DLQI: Dermatology Life Quality Index; IGA: Investigator's global assessment; LS: Least squares; NRS: numeric rating scale; PAS: prurigo activity score; WI-NRS: worst itch numeric rating scale.

9.4.2 Safety analyses

All safety analyses will be performed on the Safety Population. The summary of safety results will be presented by treatment group. The baseline value is defined generally as the last available value before randomization.

Table 7 - Safety analyses

Endpoint	Statistical analysis methods
Percentage of participants experiencing TEAEs or SAEs from baseline through Week 24	Adverse event incidence tables will present by SOC (sorted by internationally agreed order), and PT sorted in alphabetical order for each treatment group, the number (n) and percentage (%) of participants experiencing an AE. Multiple occurrences of the same event in the same participant will be counted only once in the tables within a treatment phase. The denominator for computation of percentages is the safety population within each treatment group.<br>Proportion of participants with at least one TEAE, treatment emergent SAE, TEAE leading to death, and TEAE leading to permanent treatment discontinuation will be tabulated by treatment group. In addition TEAEs will be described according to maximum intensity and relation to the study intervention. Serious AEs and AEs leading to study discontinuation that occur outside the treatment-emergent period will be summarized separately.

AE: adverse event; PT: preferred term; SOC: system organ class; SAE: serious adverse event; TEAE: treatment-emergent adverse event

9.4.3 Other analyses

Table 8 - Other analyses

Endpoint	Statistical analysis methods
Incidence of treatment-emergent ADA against dupilumab over time	The ADA analysis will be conducted on ADA population. ADA variables including treatment-emergent ADA will be summarized using descriptive statistics by treatment group. Drug concentration data will be examined and the influence of ADAs on individual concentration-time profiles will be evaluated. Assessment of the potential impact of ADA on safety and efficacy may be provided.

ADA: antidrug antibodies

Other PK, PD, biomarker, and outcome measures exploratory analyses will be described in the SAP finalized before database lock. The population PK analysis and PD analyses will be presented separately from the main clinical study report (CSR).

9.5 INTERIM ANALYSES

No interim analysis is planned.

A primary database lock will be performed when all randomized participants in this study have completed their 24-week treatment phase. Final analyses in the CSR will be based on this database.

The database will be updated at the end of the study for all participants to include the post-treatment follow-up information and updates for the events previously ongoing at the time of the primary lock. Additional data between this database lock and last participant completing last visit will be summarized in a CSR addendum.

More details will be described in the SAP.

9.5.1 Data Monitoring Committee (DMC)

Due to extensive safety record of the post marked IMP (dupilumab), it is not planned to have a DMC for this study.

9.5.2 Unblinding plan

The unblinding plan will be detailed in the SAP or in a separate unblinding plan.

10 SUPPORTING DOCUMENTATION AND OPERATIONAL CONSIDERATIONS

10.1 APPENDIX 1: REGULATORY, ETHICAL, AND STUDY OVERSIGHT CONSIDERATIONS

10.1.1 Regulatory and Ethical Considerations

- This study will be conducted in accordance with the protocol and with the following:
  - Consensus ethical principles derived from international guidelines including the Declaration of Helsinki and the applicable amendments and Council for International Organizations of Medical Sciences (CIOMS) International Ethical Guidelines.
  - Applicable ICH GCP Guidelines.
  - Applicable laws and regulations.
- The protocol, protocol amendments, ICF, Investigator Brochure, and other relevant documents (eg, advertisements) must be submitted to an IRB/IEC by the Investigator and reviewed and approved by the IRB/IEC before the study is initiated.
- Any amendments to the protocol will require IRB/IEC approval before implementation of changes made to the study design, except for changes necessary to eliminate an immediate hazard to study participants.
- The Investigator will be responsible for the following:
  - Providing written summaries of the status of the study to the IRB/IEC annually or more frequently in accordance with the requirements, policies, and procedures established by the IRB/IEC.
  - Notifying the IRB/IEC of SAEs or other significant safety findings as required by IRB/IEC procedures.
  - Providing oversight of the conduct of the study at the site and adherence to requirements of 21 Code of Federal Regulations (CFR), ICH guidelines, the IRB/IEC, European regulation 536/2014 for clinical studies (if applicable), and all other applicable local regulations.

10.1.2 Financial disclosure

Investigators and sub-Investigators will provide the Sponsor with sufficient, accurate financial information as requested to allow the Sponsor to submit complete and accurate financial certification or disclosure statements to the appropriate regulatory authorities. Investigators are responsible for providing information on financial interests during the course of the study and for 1 year after completion of the study.

10.1.3 Informed consent process

- The Investigator or his/her representative will explain the nature of the study to the participant or his/her legally authorized representative and answer all questions regarding the study.
- Participants must be informed that their participation is voluntary. Participants will be required to sign a statement of informed consent that meets the requirements of 21 CFR 50, local regulations, ICH guidelines, Health Insurance Portability and Accountability Act requirements, where applicable, and the IRB/IEC or study center.
- The medical record must include a statement that written informed consent was obtained before the participant was enrolled in the study and the date the written consent was obtained. The authorized person obtaining the informed consent must also sign the ICF.
- Participants must be re-consented to the most current version of the ICF(s) during their participation in the study.
- A copy of the ICF(s) must be provided to the participant or the participant's legally authorized representative.

Participants who are rescreened are required to sign a new ICF.

The ICF will contain a separate section that addresses the use of remaining mandatory samples as well as additional serum/plasma samples for optional exploratory research. The Investigator or authorized designee will explain to each participant the objectives of the exploratory research. Participants will be told that they are free to refuse to participate and may withdraw their consent at any time and for any reason during the storage period. A separate signature will be required to document a participant's agreement to allow any remaining specimens to be used for exploratory research. Participants who decline to participate in this optional research will not provide this separate signature.

The ICF will also contains a separate section concerning the other optional procedures (genetic testing [DNA/RNA sampling] and, for selected sites, skin biopsy and photography). A separate signature will be required to document a participant's agreement to each of these procedures. Participants who decline to participate will not provide this separate signature. A specific consent will be provided for HIV testing if specific consent is locally required.

10.1.4 Data protection

All personal data collected related to participants, Investigators, or any person involved in the study, which may be included in the Sponsor's databases, shall be treated in compliance with all applicable laws and regulations including the Global Data Protection Regulation.

Data collected must be adequate, relevant and not excessive, in relation to the purposes for which they are collected. Each category of data must be properly justified and in line with the study objective.

VV-CLIN-0549724 3.0

Participant race and ethnicity will be collected in this study because these data are required by regulatory agencies (eg, for reporting effectiveness data by racial subgroup in marketing authorization applications for FDA, or to report on the Japanese population for the Pharmaceuticals and Medical Devices Agency in Japan). In addition, race has been identified to influence PN incidence (15).

- Participants will be assigned a unique identifier by the Sponsor. Any participant records or datasets that are transferred to the Sponsor will contain the identifier only; participant names or any information which would make the participant identifiable will not be transferred.
- The participant must be informed that his/her personal study-related data will be used by the Sponsor in accordance with local data protection law. The level of disclosure must also be explained to the participant.
- The participant must be informed that his/her medical records may be examined by Clinical Quality Assurance auditors or other authorized personnel appointed by the Sponsor, by appropriate IRB/IEC members, and by inspectors from regulatory authorities.
- When archiving or processing personal data pertaining to the Investigator and/or to the participants, the Sponsor shall take all appropriate measures to safeguard and prevent access to this data by any unauthorized third party.

10.1.5 Committees structure

There will be no study committees.

10.1.6 Dissemination of clinical study data

Sanofi shares information about clinical trials and results on publically accessible websites, based on company commitments, international and local legal and regulatory requirements, and other clinical trial disclosure commitments established by pharmaceutical industry associations. These websites include clinicaltrials.gov, EU clinicaltrialregister (eu.ctr), and sanofi.com, as well as some national registries.

In addition, results from clinical trials in patients are required to be submitted to peer-reviewed journals following internal company review for accuracy, fair balance and intellectual property. For those journals that request sharing of the analyzable data sets that are reported in the publication, interested researchers are directed to submit their request to clinicalstudydatarequest.com.

Individual participant data and supporting clinical documents are available for request at clinicalstudydatarequest.com. While making information available we continue to protect the privacy of participants in our clinical trials. Details on data sharing criteria and process for requesting access can be found at this web address: clinicalstudydatarequest.com.

10.1.7 Data quality assurance

- All participant data relating to the study will be recorded on printed or electronic CRF unless transmitted to the Sponsor or designee electronically (eg, laboratory data). The Investigator is responsible for verifying that data entries are accurate and correct by physically or electronically signing the CRF.
- The Investigator must maintain accurate documentation (source data) that supports the information entered in the CRF.
- The Investigator must permit study-related monitoring, audits, IRB/IEC review, and regulatory agency inspections and provide direct access to source data documents.
- Monitoring details describing strategy (eg, risk-based initiatives in operations and quality such as Risk Management and Mitigation Strategies and Analytical Risk-Based Monitoring), methods, responsibilities and requirements, including handling of noncompliance issues and monitoring techniques (central, remote, or on-site monitoring) are provided in separate study documents.
- The Sponsor or designee is responsible for the data management of this study including quality checking of the data.
- The Sponsor assumes accountability for actions delegated to other individuals (eg, Contract Research Organizations).
- Study monitors will perform ongoing source data verification to confirm that data entered into the CRF by authorized site personnel are accurate, complete, and verifiable from source documents; that the safety and rights of participants are being protected; and that the study is being conducted in accordance with the currently approved protocol and any other study agreements, ICH GCP, and all applicable regulatory requirements.
- Records and documents, including signed ICFs, pertaining to the conduct of this study must be retained by the Investigator for 25 years after the signature of the final study report unless local regulations or institutional policies require a longer retention period. No records may be destroyed during the retention period without the written approval of the Sponsor. No records may be transferred to another location or party without written notification to the Sponsor.

10.1.8 Source documents

- Source documents provide evidence for the existence of the participant and substantiate the integrity of the data collected. Source documents are filed at the Investigator's site.
- Data reported on the CRF or entered in the eCRF that are transcribed from source documents must be consistent with the source documents or the discrepancies must be explained. The Investigator may need to request previous medical records or transfer records, depending on the study. Also, current medical records must be available.

- Source data is all information in original records and certified copies of original records of clinical findings, observations, or other activities in a clinical trial necessary for the reconstruction and evaluation of the trial. Source data are contained in source documents. Source documents are original documents, data and records such as hospital records, clinic and office charts, laboratory notes, memoranda, pharmacy dispensing records, recorded data from automated instruments, etc.

10.1.9 Study and site closure

The Sponsor or designee reserves the right to close the study site or terminate the study at any time for any reason at the sole discretion of the Sponsor. Study sites will be closed upon study completion. A study site is considered closed when all required documents and study supplies have been collected and a study-site closure visit has been performed.

The Investigator may initiate study-site closure at any time, provided there is reasonable cause and sufficient notice is given in advance of the intended termination.

Reasons for study termination by the Sponsor, as well as reasons for the early closure of a study site by the Sponsor or Investigator may include but are not limited to:

- For study termination:
  - Information on the product leads to doubt as to the benefit/risk ratio
  - Discontinuation of further study intervention development
- For site termination:
  - Failure of the Investigator to comply with the protocol, the requirements of the IRB/IEC or local health authorities, the Sponsor’s procedures, or GCP guidelines
  - Inadequate or no recruitment (evaluated after a reasonable amount of time) of participants by the Investigator
  - Total number of participants included earlier than expected

10.1.10 Publication policy

- The results of this study may be published or presented at scientific meetings. If this is foreseen, the Investigator agrees to submit all manuscripts or abstracts to the Sponsor before submission. This allows the Sponsor to protect proprietary information and to provide comments.
- The Sponsor will comply with the requirements for publication of study results. In accordance with standard editorial and ethical practice, the Sponsor will generally support publication of multicenter studies only in their entirety and not as individual site data. In this case, a coordinating Investigator will be designated by mutual agreement.
- Authorship will be determined by mutual agreement and in line with International Committee of Medical Journal Editors authorship requirements.

10.2 APPENDIX 2: CLINICAL LABORATORY TESTS

The tests detailed in Table 9 will be performed by the central laboratory.

- Local laboratory results are only required in the event that the central laboratory results are not available in time for either study intervention administration and/or response evaluation. If a local sample is required, it is important that the sample for central analysis is obtained at the same time. Additionally, if the local laboratory results are used to make either a study intervention decision or response evaluation, the results must be entered into the CRF.
- Protocol-specific requirements for inclusion or exclusion of participants are detailed in Section 5 of the protocol.
- Additional tests may be performed at any time during the study as determined necessary by the Investigator or required by local regulations.
- Additional serum or urine pregnancy tests may be performed, as determined necessary by the investigator or required by local regulation, to establish the absence of pregnancy at any time during the subject's participation in the study.

Table 9 - Protocol-required laboratory assessments

Laboratory assessments	Parameters
Hematology	
	Platelet count
	RBC count
	Hemoglobin
	Hematocrit
	WBC count with differential:
	Neutrophils
	Lymphocytes
	Monocytes
	Eosinophils
	Basophils
	CD4 T cell count (for participants with HIV history only)
Clinical chemistry[a]	
	BUN
	Creatinine
	Glucose
	Lactate dehydrogenase
	Uric acid
	Total cholesterol
	Potassium

Laboratory assessments	Parameters
	Sodium<br>Chloride<br>Bicarbonate<br>AST/SGOT<br>ALT/SGPT<br>Alkaline phosphatase<br>Creatine phosphokinase<br>Total bilirubin<br>Total protein<br>Albumin
Routine urinalysis	• Specific gravity<br>• pH, glucose, protein, blood, ketones, bilirubin, urobilinogen, nitrite, leukocyte esterase by dipstick<br>• Creatinine and leukotriene (central laboratory)<br>• Microscopic examination (if blood or protein is abnormal)
Other screening tests	• Highly sensitive serum (at screening) or urine (at other timepoints) hCG pregnancy test (as needed for WOCBP)[b]<br>• Serology[c]: HBs Ag, HBs Ab, HBc Ab, HCV Ab, HIV screen (Anti-HIV-1 and HIV-2 antibodies); HIV viral load test (for participants with HIV history only)<br>• Tuberculosis test<br>• All study-required laboratory assessments will be performed by a central laboratory, with the exception of TB test, urine pregnancy test, and routine urinalysis.

NOTES:

a Details of liver chemistry stopping criteria and required actions and follow-up assessments after liver stopping or monitoring event are given in Section 7.1 and Section 10.6. All events which may indicate severe liver injury (meeting Hy's Law laboratory criteria; ALT or AST >3 x ULN and total bilirubin >2 x ULN) must be reported as an SAE.

b After screening, local urine testing will be standard for the protocol unless serum testing is required by local regulation or IRB/IEC.

c In case of results showing HBs Ag (negative), HBs Ab (negative) and HBc Ab (positive), an HBV DNA testing may be performed prior to randomization to rule out a false positivity if the Investigator believes the patient is a false positive, or to clarify the serological status if the Investigator finds it unclear to interpret in absence of known HBV infection. In case of results showing HCV Ab (positive), an HCV RNA testing may be performed to rule out a false positivity, if the Investigator believes the patient is a false positive.

ALT: alanine aminotransferase; AST: aspartate aminotransferase; BUN: blood urea nitrogen; DNA: deoxyribonucleic acidHBc Ab: hepatitis B core antibody; HBs Ab: hepatitis B surface antibody; HBs Ag: hepatitis B surface antigen; HBV: hepatitis B virus; hCG: human chorionic gonadotropin; HCV: hepatitis C virus; HCV Ab: hepatitis C virus antibody; HIV: human immunodeficiency virus; IEC: Independent Ethics Committee; IRB: Institutional Review Board; RBC: red blood cells; RNA: ribonucleic acid; SAE: serious adverse event; SGOT: serum glutamic-oxaloacetic transaminase; SGPT: serum glutamic-pyruvic transaminase; TB: tuberculosis; ULN: upper limit of normal; WBC: white blood cells; WOCBP: woman of childbearing potential.

Investigators must document their review of each laboratory safety report.

Laboratory/analyte results that could unblind the study will not be reported to investigative sites or other blinded personnel until the study has been unblinded.

VV-CLIN-0[illegible] [illegible].0

10.3 APPENDIX 3: ADVERSE EVENTS: DEFINITIONS AND PROCEDURES FOR RECORDING, EVALUATING, FOLLOW-UP, AND REPORTING

DEFINITION OF AE

AE definition

- An AE is any untoward medical occurrence in a patient or clinical study participant, temporally associated with the use of study intervention, whether or not considered related to the study intervention.

  NOTE: An AE can therefore be any unfavorable and unintended sign (including an abnormal laboratory finding), symptom, or disease (new or exacerbated) temporally associated with the use of study intervention.

Events meeting the AE definition

- Any abnormal laboratory test results (hematology, clinical chemistry, or urinalysis) or other safety assessments (eg, ECG, radiological scans, vital signs measurements), including those that worsen from baseline, considered clinically significant in the medical and scientific judgment of the Investigator (ie, not related to progression of underlying disease).
- Exacerbation of a chronic or intermittent pre-existing condition including either an increase in frequency and/or intensity of the condition.
- New conditions detected or diagnosed after study intervention administration even though it may have been present before the start of the study.
- Signs, symptoms, or the clinical sequelae of a suspected drug-drug interaction.
- Signs, symptoms, or the clinical sequelae of a suspected overdose of either study intervention or a concomitant medication.
- “Lack of efficacy” or “failure of expected pharmacological action” per se will not be reported as an AE or SAE. Such instances will be captured in the efficacy assessments. However, the signs, symptoms, and/or clinical sequelae resulting from lack of efficacy will be reported as AE or SAE if they fulfil the definition of an AE or SAE.

Events NOT meeting the AE definition

- Any clinically significant abnormal laboratory findings or other abnormal safety assessments which are associated with the underlying disease, unless judged by the Investigator to be more severe than expected for the participant’s condition.
- The disease/disorder being studied or expected progression, signs, or symptoms of the disease/disorder being studied, unless more severe than expected for the participant’s condition.
- Medical or surgical procedure (eg, endoscopy, appendectomy): the condition that leads to the procedure is the AE.
- Situations in which an untoward medical occurrence did not occur (social and/or convenience admission to a hospital).
- Anticipated day-to-day fluctuations of pre-existing disease(s) or condition(s) present or detected at the start of the study that do not worsen.

DEFINITION OF SAE

If an event is not an AE per definition above, then it cannot be an SAE even if serious conditions are met (eg, hospitalization for signs/symptoms of the disease under study, death due to progression of disease).

A SAE is defined as any untoward medical occurrence that, at any dose:

a) Results in death

b) Is life-threatening

The term “life-threatening” in the definition of “serious” refers to an event in which the participant was at risk of death at the time of the event. It does not refer to an event, which hypothetically might have caused death, if it were more severe.

c) Requires inpatient hospitalization or prolongation of existing hospitalization

In general, hospitalization signifies that the participant has been detained (usually involving at least an overnight stay) at the hospital or emergency ward for observation and/or treatment that would not have been appropriate in the physician’s office or outpatient setting. Complications that occur during hospitalization are AEs. If a complication prolongs hospitalization or fulfills any other serious criteria, the event is serious. When in doubt as to whether “hospitalization” occurred or was necessary, the AE should be considered serious.

Hospitalization for elective treatment of a pre-existing condition that did not worsen from baseline is not considered an AE.

d) Results in persistent disability/incapacity

- The term disability means a substantial disruption of a person’s ability to conduct normal life functions.
- This definition is not intended to include experiences of relatively minor medical significance such as uncomplicated headache, nausea, vomiting, diarrhea, influenza, and accidental trauma (eg, sprained ankle) which may interfere with or prevent everyday life functions but do not constitute a substantial disruption.

e) Is a congenital anomaly/birth defect

f) Other situations:

- Medical or scientific judgment should be exercised in deciding whether SAE reporting is appropriate in other situations such as important medical events that may not be immediately life-threatening or result in death or hospitalization but may jeopardize the participant or may require medical or surgical intervention to prevent one of the other outcomes listed in the above definition. These events should usually be considered serious.
- Examples of such events include invasive or malignant cancers, intensive treatment in an emergency room or at home for allergic bronchospasm, blood dyscrasias or convulsions that do not result in hospitalization, or development of drug dependency or drug abuse.

RECORDING AND FOLLOW-UP OF AE AND/OR SAE

AE and SAE recording

- When an AE/SAE occurs, it is the responsibility of the Investigator to review all documentation (eg, hospital progress notes, laboratory reports, and diagnostics reports) related to the event.
- The Investigator will then record all relevant AE/SAE information in the CRF.
- It is **not** acceptable for the Investigator to send photocopies of the participant's medical records to the Sponsor's representative in lieu of completion of the AE/SAE CRF page.
- There may be instances when copies of medical records for certain cases are requested by the Sponsor's representative. In this case, all participant identifiers, with the exception of the participant number, will be redacted on the copies of the medical records before submission to the Sponsor's representative.
- The Investigator will attempt to establish a diagnosis of the event based on signs, symptoms, and/or other clinical information. Whenever possible, the diagnosis (not the individual signs/symptoms) will be documented as the AE/SAE.

Assessment of intensity

The Investigator will make an assessment of intensity for each AE and SAE reported during the study and assign it to 1 of the following categories:

- Mild: An event that is easily tolerated by the participant, causing minimal discomfort and not interfering with everyday activities.
- Moderate: An event that causes sufficient discomfort and interferes with normal everyday activities.
- Severe: An event that prevents normal everyday activities. An AE that is assessed as severe should not be confused with a SAE. Severe is a category utilized for rating the intensity of an event; and both AEs and SAEs can be assessed as severe.

  An event is defined as "serious" when it meets at least 1 of the predefined outcomes as described in the definition of an SAE, NOT when it is rated as severe.

Assessment of causality

- The Investigator is obligated to assess the relationship between study intervention and each occurrence of each AE/SAE.
- A "reasonable possibility" of a relationship conveys that there are facts, evidence, and/or arguments to suggest a causal relationship, rather than that a relationship cannot be ruled out.
- The Investigator will use clinical judgment to determine whether or not the relationship is causal.

- Alternative causes, such as underlying disease(s), concomitant therapy, and other risk factors, as well as the temporal relationship of the event to study intervention administration will be considered and investigated.
- The Investigator will also consult the IB and/or Product Information, for marketed products, in his/her assessment.
- For each AE/SAE, the Investigator **must** document in the medical notes that he/she has reviewed the AE/SAE and has provided an assessment of causality.
- There may be situations in which an SAE has occurred and the Investigator has minimal information to include in the initial report to the Sponsor's representative. However, **it is very important that the Investigator always make an assessment of causality for every event before the initial transmission of the SAE data to the Sponsor's representative.**
- The Investigator may change his/her opinion of causality in light of follow-up information and send a SAE follow-up report with the updated causality assessment.
- The causality assessment is one of the criteria used when determining regulatory reporting requirements.

Follow-up of AEs and SAEs

- The Investigator is obligated to perform or arrange for the conduct of supplemental measurements and/or evaluations as medically indicated or as requested by the Sponsor's representative to elucidate the nature and/or causality of the AE or SAE as fully as possible. This may include additional laboratory tests or investigations, histopathological examinations, or consultation with other health care professionals.
- If a participant dies during participation in the study or during a recognized follow-up period, the Investigator will provide the Sponsor's representative with a copy of any post-mortem findings including histopathology.
- New or updated information will be recorded in the originally completed CRF.
- The Investigator will submit any updated SAE data to the Sponsor within 24 hours of receipt of the information.

REPORTING OF SAES

SAE reporting to the Sponsor via an electronic data collection tool

- The primary mechanism for reporting an SAE to the Sponsor's representative will be the electronic data collection tool.
- If the electronic system is unavailable, then the site will use the paper SAE data collection tool (see next section) in order to report the event within 24 hours.
- The site will enter the SAE data into the electronic system as soon as it becomes available.
- After the study is completed at a given site, the electronic data collection tool will be taken off-line to prevent the entry of new data or changes to existing data.

- If a site receives a report of a new SAE from a study participant or receives updated data on a previously reported SAE after the electronic data collection tool has been taken off-line, then the site can report this information on a paper SAE form (see next section) or to the Sponsor's representative by telephone.
- Contacts for SAE reporting can be found in the protocol.

SAE reporting to the Sponsor's representative via paper CRF

- Facsimile transmission of the SAE paper CRF is the preferred method to transmit this information to the Sponsor's representative.
- In rare circumstances and in the absence of facsimile equipment, notification by telephone is acceptable with a copy of the SAE data collection tool sent by overnight mail or courier service.
- Initial notification via telephone does not replace the need for the Investigator to complete and sign the SAE CRF pages within the designated reporting time frames.
- Contacts for SAE reporting can be found in the protocol.

10.4 APPENDIX 4: CONTRACEPTIVE GUIDANCE AND COLLECTION OF PREGNANCY INFORMATION

DEFINITIONS:

Woman of childbearing potential (WOCBP)

A woman is considered fertile following menarche and until becoming post-menopausal unless permanently sterile (see below).

If fertility is unclear (eg, amenorrhea in adolescents or athletes) and a menstrual cycle cannot be confirmed before first dose of study intervention, additional evaluation should be considered.

Women in the following categories are not considered WOCBP

1. Premenarchal
2. Premenopausal female with 1 of the following:
   - Documented hysterectomy
   - Documented bilateral salpingectomy
   - Documented bilateral oophorectomy

   For individuals with permanent infertility due to an alternate medical cause other than the above, (eg, mullerian agenesis, androgen insensitivity), Investigator discretion should be applied to determining study entry.

   Note: Documentation can come from the site personnel's: review of the participant's medical records, medical examination, or medical history interview.

3. Postmenopausal female
   - A postmenopausal state is defined as no menses for 12 consecutive months without an alternative medical cause.
   - Females on hormone replacement therapy (HRT) and whose menopausal status is in doubt will be required to use one of the non-estrogen hormonal highly effective contraception methods if they wish to continue their HRT during the study.

CONTRACEPTION GUIDANCE

Female participants of childbearing potential are eligible to participate if they agree to use a highly effective method of contraception consistently and correctly as described below.

Contraceptives[a] allowed during the study include:

Highly effective methods[b] that have low user dependency

Failure rate of <1% per year when used consistently and correctly.

- Implantable progestogen-only hormone contraception associated with inhibition of ovulation[b]
  - Intrauterine device (IUD)
  - Intrauterine hormone-releasing system (IUS)[b]
- Bilateral tubal occlusion

Vasectomized partner

(Vasectomized partner is a highly effective contraceptive method provided that the partner is the sole sexual partner of the WOCBP and the absence of sperm has been confirmed. If not, an additional highly effective method of contraception should be used. Spermatogenesis cycle is approximately 90 days.)

Highly effective methods[b] that are user dependent

Failure rate of <1% per year when used consistently and correctly.

- Combined (estrogen- and progestogen-containing) hormonal contraception associated with inhibition of ovulation[c]
  - oral
  - intravaginal
  - transdermal
  - injectable
- Progestogen-only hormone contraception associated with inhibition of ovulation[c]
  - oral
  - injectable

Sexual abstinence

Sexual abstinence is considered a highly effective method only if defined as refraining from heterosexual intercourse during the entire period of risk associated with the study intervention. The reliability of sexual abstinence needs to be evaluated in relation to the duration of the study and the preferred and usual lifestyle of the participant.)

Acceptable methods[d]

- Progestogen-only oral hormonal contraception where inhibition of ovulation is not the primary mode of action
- Male or female condom with or without spermicide[e]
- Cervical cap, diaphragm, or sponge with spermicide
- A combination of male condom with either cervical cap, diaphragm, or sponge with spermicide (double-barrier methods)[c]

a Contraceptive use by men or women should be consistent with local regulations regarding the use of contraceptive methods for those participating in clinical studies.
b Failure rate of <1% per year when used consistently and correctly. Typical use failure rates differ from those when used consistently and correctly.
c If locally required, in accordance with Clinical Trial Facilitation Group (CTFG) guidelines, acceptable contraceptive methods are limited to those which inhibit ovulation as the primary mode of action.
d Considered effective, but not highly effective - failure rate of ≥1% per year. Periodic abstinence (calendar, symptothermal, post-ovulation methods), withdrawal (coitus interruptus), spermicides only, and lactational amenorrhoea method (LAM) are not acceptable methods of contraception.
e Male condom and female condom should not be used together (due to risk of failure with friction).
WOCBP: woman of childbearing potential.

COLLECTION OF PREGNANCY INFORMATION:

Male participants with partners who become pregnant

- The Investigator will attempt to collect pregnancy information on any male participant's female partner who becomes pregnant while the male participant is in this study. This applies only to male participants who receive dupilumab.
- After obtaining the necessary signed informed consent from the pregnant female partner directly, the Investigator will record pregnancy information on the appropriate form and submit it to the Sponsor within 24 hours of learning of the partner's pregnancy. The female partner will also be followed to determine the outcome of the pregnancy. Information on the status of the mother and child will be forwarded to the Sponsor. Generally, the follow-up will be no longer than 6 to 8 weeks following the estimated delivery date. Any termination of the pregnancy will be reported regardless of fetal status (presence or absence of anomalies) or indication for the procedure.

Female participants who become pregnant

- The Investigator will collect pregnancy information on any female participant who becomes pregnant while participating in this study. Information will be recorded on the appropriate form and submitted to the Sponsor within 24 hours of learning of a participant's pregnancy. The participant will be followed to determine the outcome of the pregnancy. The Investigator will collect follow-up information on the participant and the neonate and the information will be forwarded to the Sponsor. Generally, follow-up will not be required for longer than 6 to 8 weeks beyond the estimated delivery date. Any termination of pregnancy will be reported, regardless of fetal status (presence or absence of anomalies) or indication for the procedure.
- Any pregnancy complication or elective termination of a pregnancy will be reported as an AE or SAE. A spontaneous abortion is always considered to be an SAE and will be reported as such. Any post-study pregnancy related SAE considered reasonably related to the study intervention by the Investigator will be reported to the Sponsor as described in Section 8.3.4 of the protocol. While the Investigator is not obligated to actively seek this information in former study participants, he or she may learn of an SAE through spontaneous reporting.
- Any female participant who becomes pregnant while participating in the study will discontinue study intervention.

10.5 APPENDIX 5: GENETICS

Use/Analysis of DNA and RNA

- Genetic variation may impact a participant's response to study intervention, susceptibility to, and severity and progression of disease. Variable response to study intervention may be due to genetic determinants that impact drug absorption, distribution, metabolism, and excretion; mechanism of action of the drug; safety, disease etiology; and/or molecular subtype of the disease being treated. Therefore, where local regulations and IRB/IEC allow, a blood sample will be collected for DNA analysis from consenting participants.
- DNA and RNA samples will be used for research related to dupilumab or PN and related diseases. They may also be used to develop tests/assays including diagnostic tests related to dupilumab or related drugs and atopic/allergic diseases. Genetic research may consist of the analysis of one or more candidate genes or the analysis of genetic markers throughout the genome including whole-exome sequencing, whole-genome sequencing, and DNA copy number variation. Transcriptome sequencing (or other methods for quantitating RNA expression) may also be performed.
- The samples may be analyzed as part of a multi-study assessment of genetic factors involved in the response to dupilumab or study interventions of this class to understand study disease or related conditions.
- The results of genetic analyses will not be reported in the CSR.
- The Sponsor will store the DNA samples in a secure storage space with adequate measures to protect confidentiality.
- The samples will be retained no longer than 15 years or other period as per local requirements.

10.6 APPENDIX 6: LIVER AND OTHER SAFETY: SUGGESTED ACTIONS AND FOLLOW-UP ASSESSMENTS

INCREASE IN ALT

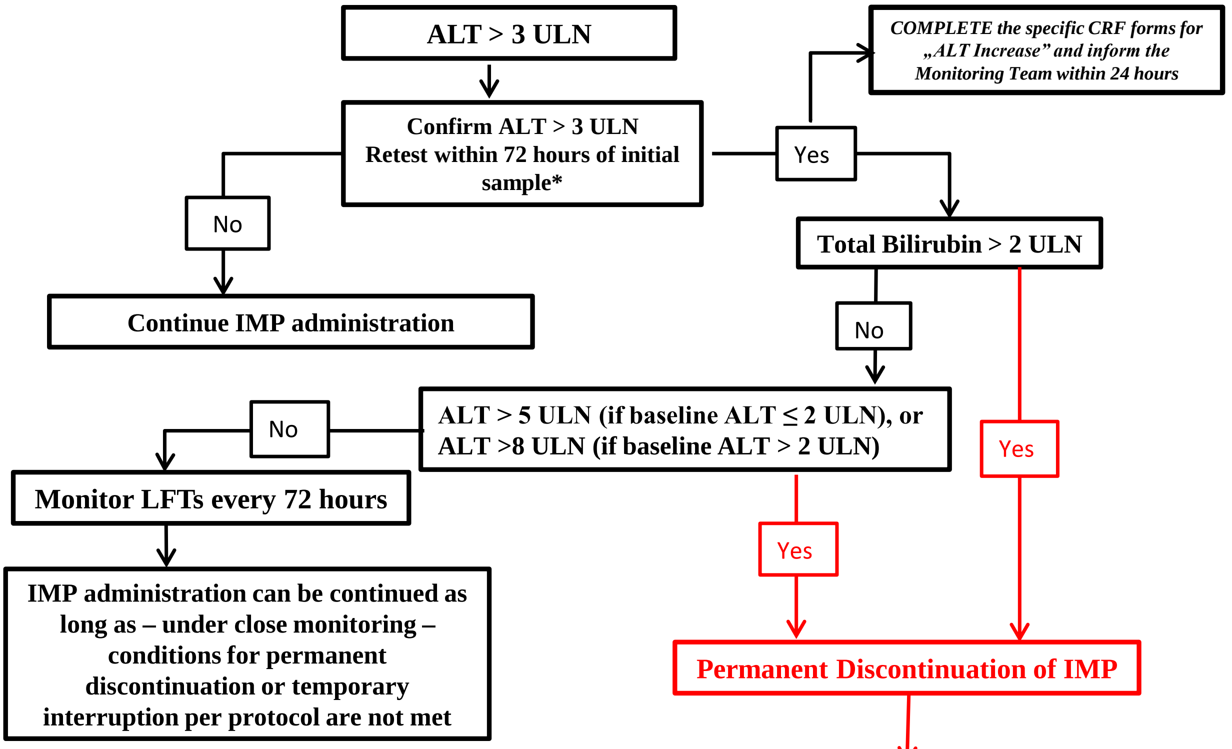

In ANY CASE, FOLLOW the instructions listed in the box below:

1. **INFORM** the Site Monitor who will forward the information to the Study Manager
2. **INVESTIGATE** specifically for malaise with or without loss of consciousness, dizziness, and/or hypotension and/or episode of arrhythmia in the previous 72 hours; rule out muscular injury
3. **PERFORM** the following tests:
   - LFTs: AST, ALT, alkaline phosphatase, total and conjugated bilirubin and prothrombin time / INR
   - CPK, serum creatinine, complete blood count
   - Anti-HAV IgM, anti-HBc IgM (HBV-DNA if clinically indicated), anti-HCV and HCV RNA, anti-CMV IgM and anti-HEV IgM antibodies
   - Depending on the clinical context, check for recent infection with EBV, herpes viruses, and toxoplasma
   - Hepatobiliary ultrasonography (or other imaging investigations if needed)
4. **CONSIDER** Auto-antibodies: antinuclear, anti-DNA, anti-smooth muscle, anti-LKM
5. **CONSIDER** consulting with hepatologist
6. **CONSIDER** patient hospitalisation if INR>2 (or PT<50%) and/or central nervous system disburbances suggesting hepatic encephalopathy
7. **MONITOR LFTs after discontinuation of IMP:**
   - As *closely as possible* (or **every 48 hours)** until stabilization, then every 2 weeks until return to normal/baseline or clinical resolution.
8. **FREEZE** serum sample (5ml x 2)
9. **In case of SUSPICION of GILBERT Syndrome,** a DNA diagnostic test should be done

*If unable to retest in 72 hours, use original lab results to decide on further reporting/monitoring/discontinuation.

Note:

"Baseline" refers to ALT sampled at baseline visit; or if baseline value unavailable, to the latest ALT sampled before the baseline visit. The algorithm does not apply to the instances of increase in ALT during screening.

See Section 10.3 for guidance on safety reporting.

Normalization is defined as ≤ULN or baseline value, if baseline value is >ULN.

INCREASE IN SERUM CREATININE in patients with normal baseline (creatininemia between 45 µmol/L and 84 µmol/L)

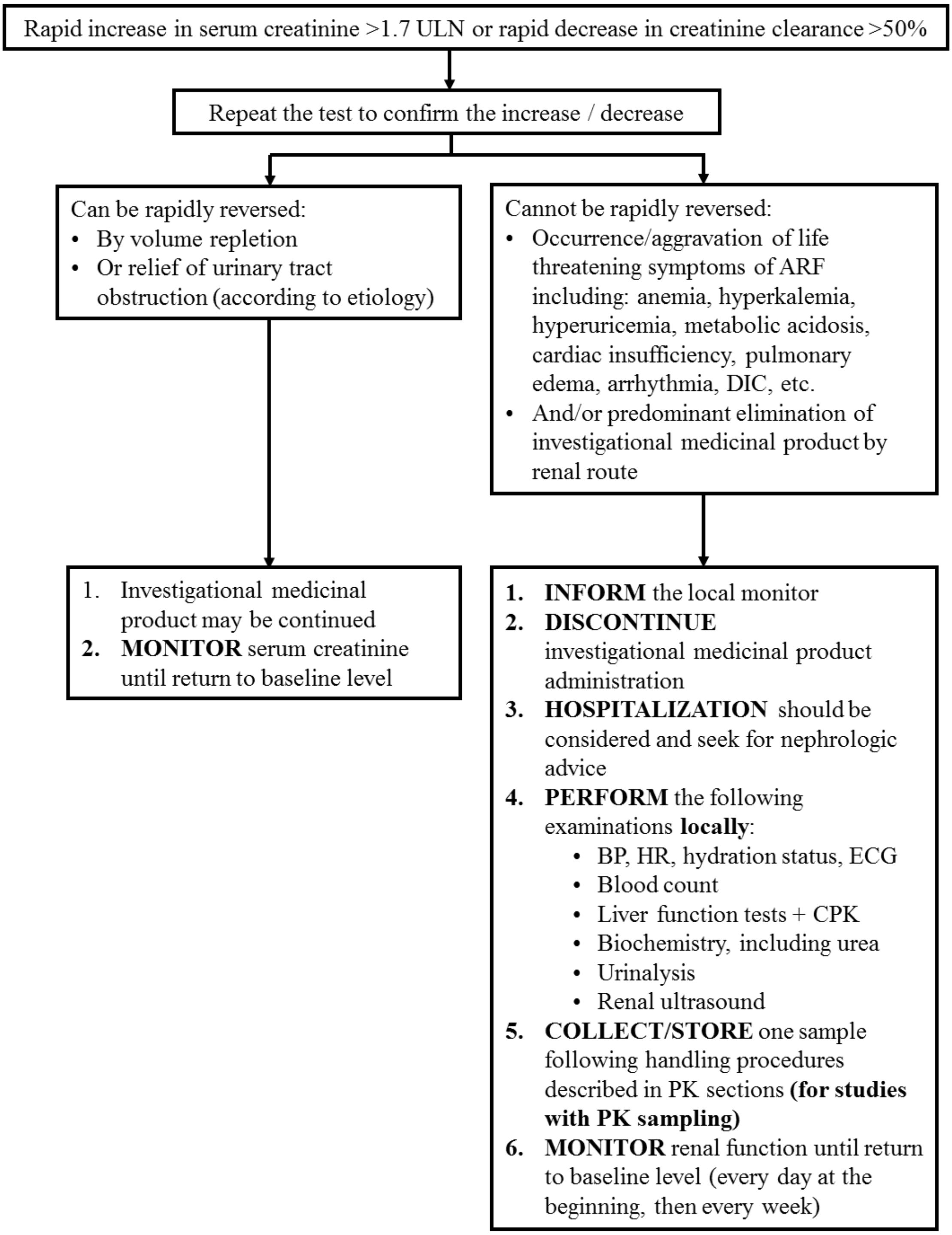

Increase in serum creatinine is to be recorded as an AE only if at least 1 of the criteria listed in the general guidelines for reporting AEs in Section 10.3 is met.

INCREASE IN CPK OF NON-CARDIAC ORIGIN AND NOT RELATED TO INTENSIVE PHYSICAL ACTIVITY

If Increase in CPK > 10 ULN

↓

Repeat immediately locally the count.
If confirmed, inform the local monitor and

↓

INVESTIGATE for the origin:
- **INTERVIEW** the patient about a recent intensive muscular effort, trauma, convulsions, electrical injury, or stress to the skeletal muscle, multiple intramuscular injections, recent surgery, concomitant medications, consumption of alcohol, morphine, cocaine…
- **PERFORM locally**:
  - ECG, CK-MB , Troponin if not previously done
  - CK-MM
  - Creatinine
  - Iono (K+)
  - Transaminases + Total and conjugated bilirubin
  - And any other parameters considered as relevant per medical judgement (e.g. $Ca^{2}+$, Myoglobin)
- **COLLECT/STORE** one sample following handling procedures described in PK sections **(for studies with PK sampling)**
- **SEARCH** for the cause

↓

1. Hospitalization should be considered depending on clinical context and profile of the patient
2. Consider to discontinue investigational medicinal product administration
3. Monitor biological parameters as appropriate within the next days/weeks/months until return to baseline

Increase in CPK is to be recorded as an AE only if at least 1 of the criteria in the general guidelines for reporting AEs in Section 10.3 is met.

10.7 APPENDIX 7: CLINICIANS-REPORTED OUTCOMES AND PATIENT-REPORTED OUTCOMES

10.7.1 Worst itch numeric rating scale (WI-NRS)

Worst-itch NRS

[redacted]

GB ZD 01/2019

UKM: AöR, Prof. Dr. Martin Schulze Schwienhorst (Aufsichtsratsvorsitzender),
Univ.-Prof. Dr. med. Dr. phil. Robert Nitsch (Vorstandsvorsitzender, Ärztlicher Direktor),
Dr. rer. pol. Christoph Hoppenheit (stellv. Vorstandsvorsitzender, Kaufmännischer Direktor),
Univ.-Prof. Dr. med. Mathias Herrmann (Dekan), Thomas van den Hooven (Pflegedirektor),
Univ.-Prof. Dr. med. Claudia Rössig (stellv. Ärztliche Direktorin)

10.7.2 Investigator's global assessment of prurigo nodularis (IGA PN)

[redacted]

10.7.3 Prurigo activity score (PAS)

[redacted]

[redacted]

10.7.4 Dermatology life quality index (DLQI)

[redacted]

10.7.5 Hospital anxiety and depression scale (HADS)

[redacted]

10.7.6 EQ-5D-5L

[redacted]

10.7.7 Pain numeric rating scale

Worst Skin Pain Numerical Rating Scale (Generic)

How would you rate your skin pain at its **worst** in the past 24 hours?

0	1	2	3	4	5	6	7	8	6	10
No pain										Worst pain possible

10.7.8 Sleep numeric rating scale

Sleep Quality Numerical Rating Scale (Generic)

Instructions:

Please complete the following questions upon awakening for the day.

Select the number that best describes the quality of your sleep last night.

0	1	2	3	4	5	6	7	8	9	10
Worst possible sleep										Best possible sleep

10.7.9 Patient Global Impression of Change of disease (PGIC)

Patient Global Impression of Change (PGIC) of PN Disease

Please choose the response below that best describes the overall change in your prurigo nodularis since you started taking the study medication.

☐ Very much better

☐ Moderately better

☐ A little better

☐ No change

☐ A little worse

☐ Moderately worse

☐ Very much worse

10.7.10 Patient Global Impression of Severity (PGIS)

Patient Global Impression of Severity (PGIS) of PN Disease

Please choose the response below that best describes the severity of your prurigo nodularis over the past week.

☐ None

☐ Mild

☐ Moderate

☐ Severe

10.7.11 Missed school/work days

Patients with Prurigo Nodularis *(PN)*

Missed School/Work days
BASELINE Visit

Site Number: __________ Patient Study Number: ___________________________

Visit Date (dd/mmm/yyyy): ______/______/______Time of Completion: ___:_____am/pm

Employment status

1. Have you been working?

□ Yes – I am working full time (≥4 days/week)
□ Yes – I am working part time (<4 days/week)
□ No – I am not working (unemployed, retired)

For those working (full time or part time):

2. How many days have you missed from work because of your Prurigo Nodularis in the last 4 weeks prior to the visit today?

Please do not include time you missed from work for taking part in this research.

Specify: __ __ __ Days

Student status

1. Have you been in school?

□ Yes – I am in school full time (≥4 days/week)
□ Yes – I am in school part time (<4 days/week)
□ No – I am not in school

For those in school (full time or part time):

2. How many days have you missed from school because of your Prurigo Nodularis in the last 4 weeks prior to the visit today?

Please do not include time you missed from school for taking part in this research.

Specify: __ __ __ Days

NOTES:

Please make sure the instructions are read to the patient BEFORE they start the questionnaire.

Assessment periods (i.e., time interval from the date of last assessment through the current date) must be continuous. Leave no gaps. Cumulatively, these periods must cover the entire study duration.

Avoid duplicate reporting. If the patient missed school/work because of his/her PN on the day of

assessment, this day should be recorded only once.

Record only days on which patient's PN prevented him/her to go to school/work. Otherwise, do not record the days on which the patient missed school/work only to participate in study visits.

Print Name (First/Last) of Investigator Completing Assessment	Signature	Date

R668-XXX-Missed School/work Days Version 1.0 BASELINE; *date*

Page 1 of 1

Patients with *Prurigo Nodularis (PN)*

Missed School/work days

POST Baseline Visits

Site Number: ______________Patient Study Number: ______________

Visit Date (dd/mmm/yyyy): ____/_____/_____Time of Completion: ___:_____am/pm

Employment status

1. Have you been working since your last assessment?

   □ Yes – I am working full time (≥4 days/week)
   □ Yes – I am working part time (<4 days/week)
   □ No – I am not working (unemployed, retired)

For those working (full time or part time):

2. How many days have you missed from work because of your Prurigo Nodularis since the day of your last assessment, including today?

   Please do not include time you missed from work for taking part in this research.

   Specify: __ __ __ Days

Student status

1. Have you been in school since your last assessment?

   □ Yes – I am in school full time (≥4 days/week)
   □ Yes – I am in school part time (<4 days/week)
   □ No – I am not in school

For those in school (full time or part time):

2. How many days have you missed from school because of your Prurigo Nodularis since the day of your last assessment, including today?

   Please do not include time you missed from school for taking part in this research.

   Specify: __ __ __ Days

NOTES:

Please make sure the instructions are read to the patient BEFORE they start the questionnaire.

Assessment periods (i.e., time interval from the date of last assessment through the current date) must be continuous. Leave no gaps. Cumulatively, these periods must cover the entire study duration.

Avoid duplicate reporting. If the patient missed school/work because of his/her PN on the day of assessment, this day should be recorded only once.

Record only days on which patient's PN prevented him/her to go to school/work. Otherwise, do not record the days on which the patient missed school/work only to participate in study visits.

**Print Name (First/Last) of Investigator Completing Assessment**	**Signature**	**Date**

R668-XXX Missed School/work Days Version 2.0; date

Page 1 of 1

10.8 APPENDIX 8: DEFINITION OF ANAPHYLAXIS

"Anaphylaxis is a serious allergic reaction that is rapid in onset and may cause death (31)."

Clinical criteria for diagnosing anaphylaxis

Anaphylaxis is highly likely when any one of the following 3 criteria are fulfilled:

1. Acute onset of an illness (minutes to several hours) with involvement of the skin, mucosal tissue, or both (eg, generalized hives, pruritus or flushing, swollen lips-tongue-uvula)
   AND AT LEAST ONE OF THE FOLLOWING
   a. Respiratory compromise (eg, dyspnea, wheeze-bronchospasm, stridor, reduced PEF, hypoxemia)
   b. Reduced BP or associated symptoms of end-organ dysfunction (eg, hypotonia [collapse], syncope, incontinence)
2. Two or more of the following that occur rapidly after exposure *to a likely allergen for that patient* (minutes to several hours):
   a. Involvement of the skin-mucosal tissue (eg, generalized hives, itch-flush, swollen lips-tongue-uvula)
   b. Respiratory compromise (eg, dyspnea, wheeze-bronchospasm, stridor, reduced PEF, hypoxemia)
   c. Reduced BP or associated symptoms (eg, hypotonia [collapse], syncope, incontinence)
   d. Persistent gastrointestinal symptoms (eg, crampy abdominal pain, vomiting)
3. Reduced BP after exposure to *known allergen for that patient* (minutes to several hours):
   a. Infants and children: low systolic BP (age specific) or greater than 30% decrease in systolic BP*
   b. Adults: systolic BP of less than 90 mm Hg or greater than 30% decrease from that person's baseline

PEF, Peak expiratory flow; *BP*, blood pressure.
*Low systolic blood pressure for children is defined as less than 70 mm Hg from 1 month to 1 year, less than (70 mm Hg + [2 × age]) from 1 to 10 years, and less than 90 mm Hg from 11 to 17 years.

10.9 APPENDIX 9: LIST OF OPPORTUNISTIC INFECTIONS

- Aspergillosis
- Blastomyces dermatitidis (endemic in the south-eastern and south-central states US, along Mississippi and Ohio Rivers)
- Candidiasis - only systemic or extensive mucosal or cutaneous candidiasis
- Coccidioides immitis (endemic south-western US and Central and South America)
- Cryptococcus
- Cytomegalovirus
- Herpes Simplex (disseminated)
- Herpes Zoster (disseminated; ophthalmic; involvement of 2 or more dermatomes)
- Histoplasmosis (pulmonary or disseminated; most common tropical areas Tennessee-Ohio-Mississippi river basins)
- Listeriosis
- Mycobacterium avium
- Nontuberculosis mycobacteria
- Pneumocystis pneumonia

This list is indicative and not exhaustive.

10.10 APPENDIX 10: COUNTRY-SPECIFIC REQUIREMENTS

Not applicable.

10.11 APPENDIX 11: ABBREVIATIONS

ACE:	angiotensin converting enzyme
AD:	atopic dermatitis
ADA:	antidrug antibody
ADR:	adverse drug reaction
AE:	adverse event
AESI:	adverse event of special interest
ALT:	alanine aminotransferase
ANCOVA:	analysis of covariance
AST:	aspartate aminotransferase
BUN:	blood urea nitrogen
CFR:	Code of Federal Regulations
CIOMS:	Council for International Organizations of Medical Sciences
ClinRO:	clinician-reported outcome
CMH:	Cochran-Mantel-Haenszel
CONSORT:	Consolidated Standards of Reporting Trials
CRF:	case report form
CRSwNP:	chronic rhinosinusitis with nasal polyposis
CSR:	clinical study report
DBP:	diastolic blood pressure
DLQI:	dermatology life quality index
DMC:	data monitoring committee
DNA:	deoxyribonucleic acid
DTP:	direct-to-patient
EADV:	European Academy of Dermatology and Venereology
EASI:	eczema area and severity index
ECG:	electrocardiogram
eCRF:	electronic case report form
ELISA:	enzyme-linked immunosorbent assay
EMA:	European Medicines Agency
EoE:	eosinophilic esophagitis
EOS:	end of study
EOT:	end of treatment
EQ-5D:	Euroqol 5 dimensions
EQ-5D-5L:	Euroqol 5 dimensions 5 levels
EU:	European Union
FDA:	Food and Drug Administration
GCP:	Good Clinical Practice
HADS:	hospital anxiety and depression scale
HBc Ab:	hepatitis B core antibody

HBs Ab:	hepatitis B surface antibody
HBs Ag:	hepatitis B surface antigen
HBV:	hepatitis B virus
hCG:	human chorionic gonadotropin
HCV:	hepatitis C virus
HCV Ab:	hepatitis C virus antibody
HIV:	human immunodeficiency virus
HRQoL:	health-related quality of life
HRT:	hormone replacement therapy
IB:	investigator's brochure
ICF:	informed consent form
ICH:	International Council for Harmonisation
IEC:	Independent Ethics Committee
Ig:	immunoglobulin
IGA:	investigator's global assessment
IGA PN:	investigator's global assessment for prurigo nodularis
IGA PN-A:	IGA PN-Activity
IGA PN-S:	IGA PN-Stage
IL:	interleukin
IMP:	investigational medicinal product
IRB:	Institutional Review Board
IRT:	interactive response technology
ITT:	intent to treat
IVRS:	interactive voice response system
IWRS:	interactive web response system
LS:	least squares
NIMP:	noninvestigational medicinal product
NRS:	numeric rating scale
PAS:	prurigo activity score
PD:	pharmacodynamic
PGA:	physician global assessment
PGIC:	Patient Global Impression of Change
PGIS:	Patient Global Impression of Severity
PK:	pharmacokinetic
PN:	prurigo nodularis
PRO:	patient reported outcome
PT:	preferred term
Q2W:	every 2 weeks
QTcF:	QT interval corrected using Fridericia's formula
RBC:	red blood cells
RNA:	ribonucleic acid
SAE:	serious adverse event
SAP:	statistical analysis plan
SBP:	systolic blood pressure
SC:	subcutaneous
SCORAD:	scoring atopic dermatitis

VV-CLIN-0649729 1.0
VV-CLIN-0539724 3.0

SGOT:	serum glutamic-oxaloacetic transaminase
SGPT:	serum glutamic-pyruvic transaminase
SNRI:	serotonin and norepinephrin reuptake inhibitor
SoA:	schedule of activities
SOC:	system organ class
SSRI:	selective serotonin reuptake inhibitor
SUSAR:	suspected unexpected serious adverse reaction
TB:	tuberculosis
TCI:	topical calcineurin inhibitors
TCS:	topical corticosteroids
TEAE:	treatment-emergent adverse event
ULN:	upper limit of normal
US:	United States
UVB:	ultraviolet B
VAS:	visual analog scale
WBC:	white blood cells
WI-NRS:	worst-itch numeric rating scale
WOCBP:	woman of childbearing potential
WOCF:	worst observation carried forward

11 REFERENCES

1. Zeidler C, Athanasios T, Pereira M, Stander H, Yosipovitch G, Stander S. Chronic prurigo of nodular type: A Review. Acta Derm Venereol 2018;98(2):173-9.

2. Iking A, Grundmann S, Chatzigeorgakidis E, Phan NQ, Klein D, Ständer S. Prurigo as a symptom of atopic and non-atopic diseases: aetiological survey in a consecutive cohort of 108 patients. J Eur Acad Dermatol Venereol. 2013;27(5):550-7.

3. Mollanazar NK, Elgash M, Weaver L, Valdes-Rodriguez R, Hsu S. Reduced itch associated with dupilumab treatment in 4 patients with prurigo nodularis. JAMA Dermatol. 2019;155(1):121-2.

4. Beck KM, Yang EJ, Sekhon S, Bhutani T, Liao W. Dupilumab treatment for generalized prurigo nodularis. JAMA Dermatol. 2019;155(1):118-20.

5. Rambhia PH, Levitt JO. Recalcitrant prurigo nodularis treated successfully with dupilumab. JAAD Case Rep. 2019;5(5):471-3.

6. Calugareanu A, Jachiet M, Lepelletier C, De Masson A, Rybojad M, Bagot M, et al. Dramatic improvement of generalized prurigo nodularis with dupilumab. J Eur Acad Dermatol Venereol. 2019; 33(8):e303-4.

7. Zhai LL, Savage KT, Qiu CC, Jin A, Valdes-Rodriguez R, Mollanazar NK. Chronic Pruritus Responding to Dupilumab-A Case Series. Medicines (Basel). 2019;6(3):E72.

8. Weigelt N, Metze D, Stander S. Prurigo nodularis: systematic analysis of 58 histological criteria in 136 patients. J Cutan Pathol. 2010;37(5):578-86

9. Yang TB, Kim BS. Pruritus in allergy and immunology. J Allergy Clin Immunol. 2019;144(2):353-60.

10. Pereira MP, Steinke S, Zeidler C, Forner C, Riepe C, Augustin M, et al. European academy of dermatology and venereology European prurigo project: expert consensus on the definition, classification and terminology of chronic prurigo. J Eur Acad Dermatol Venereol. 2018;32(7):1059-65.

11. Zeidler C, Ständer S. The pathogenesis of Prurigo nodularis--'Super-Itch' in exploration. Eur J Pain. 2016;20(1):37-40.

12. Fukushi S, Yamasaki K, Aiba S. Nuclear localization of activated STAT6 and STAT3 in epidermis of prurigo nodularis. Br J Dermatol. 2011;165(5):990-6.

13. Tokura Y, Yagi H, Hanaoka K, Ito T, Towyama K, Sachi Y, et al. Subacute and chronic prurigo effectively treated with recombination interferon-gamma: implications for

participation of Th2 cells in the pathogenesis of prurigo. Acta Derm Venereol. 1997;77(3):231-4.

14. Oetjen LK, Mack MR, Feng J, Whelan TM, Niu H, Guo CJ, et al. Sensory neurons co-opt classical immune signaling pathways to mediate chronic itch. Cell. 2017;171(1):217-28.

15. Boozalis E, Tang O, Patel S, Semenov YR, Pereira MP, Stander S, et al. Ethnic differences and comorbidities of 909 prurigo nodularis patients. J Am Acad Dermatol. 2018;79(4):714-9.

16. Steinke S, Zeidler C, Riepe C, Bruland P, Soto-Rey I, Storck M, et al. Humanistic burden of chronic pruritus in patients with inflammatory dermatoses: Results of the European Academy of Dermatology and Venereology Network on Assessment of Severity and Burden of Pruritus (PruNet) cross-sectional trial. J Am Acad Dermatol. 2018;79(3):457-63.

17. Tanaka M, Aiba S, Matsumura N, Aoyama H, Tagami H. Prurigo nodularis consists of two distinct forms: early-onset atopic and late-onset non-atopic. Dermatology. 1995;190(4):269-76.

18. Kowalski EH, Kneiber D, Valdebran M, Patel U, Amber KT. Treatment-resistant prurigo nodularis: challenges and solutions. Clin Cosmet Investig Dermatol. 2019;12:163-72.

19. Novartis Laboratories. NEORAL Soft Gelatin Capsules (cyclosporine capsules, USP) MODIFIED NEORAL Oral Solution (cyclosporine oral solution, USP) MODIFIED. Prescribing information. 2009.

20. Ständer S, Kwon P, Hirman J, Perlman AJ, Weisshaar E, Metz M, et al. Serlopitant reduced pruritus in patients with prurigo nodularis in a phase 2, randomized, placebo-controlled trial. J Am Acad Dermatol. 2019;80(5):1395-402.

21. Verweyen E, Ständer S, Kreitz K, Höben I, Osada N, Gernart M, et al. Validation of a Comprehensive Set of Pruritus Assessment Instruments: The Chronic Pruritus Tools Questionnaire PRURITOOLS. Acta Derm Venereol. 2019;99(7):657-63.

22. Reich A, Bożek A, Janiszewska K, Szepietowski JC. 12-Item Pruritus Severity Scale: Development and Validation of New Itch Severity Questionnaire. Biomed Res Int. 2017;2017:3896423.

23. Reich A, Riepe C, Anastasiadou Z, Mędrek K, Augustin M, Szepietowski JC, et al. Itch Assessment with Visual Analogue Scale and Numerical Rating Scale: Determination of Minimal Clinically Important Difference in Chronic Itch. Acta Derm Venereol. 2016;96(7):978-80.

24. Phan NQ, Blome C, Fritz F, Gerss J, Reich A, Ebata T, et al. Assessment of pruritus intensity: prospective study on validity and reliability of the visual analogue scale, numerical rating scale and verbal rating scale in 471 patients with chronic pruritus. Acta Derm Venereol. 2012;92(5):502-7.

25. Chernyshov PV. The evolution of quality of life assessment and use in dermatology. Dermatology. 2019;235(3):167-74.

26. Pölking J, Zeidler C, Schedel F, Osada N, Augustin M, Metze D, et al. Prurigo Activity Score (PAS): validity and reliability of a new instrument to monitor chronic prurigo. J Eur Acad Dermatol Venereol. 2018;32(10):1754-60.

27. Finlay AY, Khan GK. Dermatology Life Quality Index (DLQI)--a simple practical measure for routine clinical use. Clin Exp Dermatol. 1994;19(3):210-6.

28. Zigmond AS, Snaith RP. The hospital anxiety and depression scale. Acta Psychiatr Scand. 1983;67(6):361-70.

29. Herrmann C. International experiences with the Hospital Anxiety and Depression Scale--a review of validation data and clinical results. J Psychosom Res. 1997;42(1):17-41.

30. Herdman M, Gudex C, Lloyd A, Janssen M, Kind P, Parkin D, et al. Development and preliminary testing of the new five-level version of EQ-5D (EQ-5D-5L). Qual Life Res. 2011;20(10):1727-36.

31. Sampson HA, Munoz-Furlong A, Campbell RL, Adkinson NF, Bock SA, Branum A, et al. Second symposium on the definition and management of anaphylaxis: Summary report - Second National Institute of Allergy and Infectious Disease/Food Allergy and Anaphylaxis Network symposium. J Allergy Clin Immunol. 2006;117(2):391-7.

Signature Page for VV-CLIN-0549724 v3.0
efc16459-16-1-1-protocol

Approve & eSign	[redacted]

Approve & eSign	[redacted]

SANOFI

STATISTICAL ANALYSIS PLAN

**Protocol title:**	**A randomized, double blind, placebo-controlled, multi-center, parallel group study to evaluate the efficacy and safety of dupilumab in patients with prurigo nodularis who are inadequately controlled on topical prescription therapies or when those therapies are not advisable**
**Protocol number:**	**EFC16459**
**Compound number (INN/Trademark):**	**SAR231893/REGN668 dupilumab/Dupixent**
**Study phase:**	**Phase 3**
**Short title:**	**Study of dupilumab for the treatment of patients with prurigo nodularis, inadequately controlled on topical prescription therapies or when those therapies are not advisable**<br>**LIBERTY-PN PRIME**
**Statistician:**	[redacted]
**Statistical project leader:**	
**Date of issue:**	**04-Feb-2021**

Regulatory agency identifier number(s):

IND:	IND107969
EudraCT:	2019-003774-41
NCT:	NCT04183335
WHO:	U1111-1241-8153
Other:	Not applicable

Total number of pages: 62

Any and all information presented in this document shall be treated as confidential and shall remain the exclusive property of Sanofi (or any of its affiliated companies). The use of such confidential information must be restricted to the recipient for the agreed purpose and must not be disclosed, published or otherwise communicated to any unauthorized persons, for any reason, in any form whatsoever without the prior written consent of Sanofi (or the concerned affiliated company); 'affiliated company' means any corporation, partnership or other entity which at the date of communication or afterwards (i) controls directly or indirectly Sanofi, (ii) is directly or indirectly controlled by Sanofi, with 'control' meaning direct or indirect ownership of more than 50% of the capital stock or the voting rights in such corporation, partnership or other entity

TABLE OF CONTENTS

STATISTICAL ANALYSIS PLAN 1

TABLE OF CONTENTS 2

LIST OF TABLES 4

VERSION HISTORY 5

1 INTRODUCTION 6

1.1 STUDY DESIGN 6

1.2 OBJECTIVE AND ENDPOINTS 6
1.2.1 Estimands 9

2 SAMPLE SIZE DETERMINATION 13

3 ANALYSIS POPULATIONS 14

4 STATISTICAL ANALYSES 16

4.1 GENERAL CONSIDERATIONS 16

4.2 PARTICIPANT DISPOSITIONS 16

4.3 PRIMARY ENDPOINT(S) ANALYSIS 17
4.3.1 Definition of endpoint(s) 17
4.3.2 Main analytical approach 19
4.3.3 Sensitivity analysis 20
4.3.4 Supplementary analyses 20
4.3.5 Subgroup analyses 20

4.4 SECONDARY ENDPOINT(S) ANALYSIS 21
4.4.1 Key/Confirmatory secondary endpoint(s) 21
4.4.1.1 Definition of endpoint(s) 21
4.4.1.2 Main analytical approach 22
4.4.2 Supportive secondary endpoint(s) 22

4.5 TERTIARY/EXPLORATORY ENDPOINT(S) ANALYSIS 24
4.5.1 Definition of endpoint(s) 24
4.5.2 Main analytical approach 27

4.6 MULTIPLICITY ISSUES 27

4.7 SAFETY ANALYSES 28
4.7.1 Extent of exposure 28
4.7.2 Adverse events 29
4.7.3 Additional safety assessments....34
4.7.3.1 Laboratory variables, vital signs and electrocardiograms (ECGs)....34

4.8 OTHER ANALYSES....36
4.8.1 Pharmacokinetic analyses 36
4.8.2 Immunogenicity analyses....36
4.8.3 Pharmacodynamic/genomics endpoints 39

4.9 INTERIM ANALYSES 40

5 SUPPORTING DOCUMENTATION 41

5.1 APPENDIX 1 LIST OF ABBREVIATIONS 41

5.2 APPENDIX 2 CHANGES TO PROTOCOL-PLANNED ANALYSES 43

5.3 APPENDIX 3 DEMOGRAPHICS AND BASELINE CHARACTERISTICS, PRIOR OR CONCOMITANT MEDICATIONS/PROCEDURES....44

5.4 APPENDIX 4 DATA HANDLING CONVENTIONS 48

5.5 APPENDIX 5 SAMPLE SAS CODE....54

5.6 APPENDIX 6 SELECTION CRITERIA FOR AE/MEDICATION GROUPINGS 57

5.7 APPENDIX 7 MEDICATION/PROCEDURE AJUDICATION ALGORITHM 59

6 REFERENCES....62

LIST OF TABLES

Table 1 - Objectives and endpoints....................6
Table 2 - Summary of primary estimand for main endpoints....................10
Table 3 - Populations for analyses....................14
Table 4 - Selected prohibited medications/procedures and rescue medications that impact on efficacy.....18
Table 5 - Sorting of AE tables....................30
Table 6 - Analyses of adverse events....................31
Table 7 - Selections for AESIs and other AEs of interest....................32
Table 8 – Major statistical changes in protocol amendment(s)....................43
Table 9 – Time window for eDiary efficacy variables....................49
Table 10 – Time window for efficacy variables....................51
Table 11 – Time window for safety endpoints....................53
Table 12 – Time window for pharmacokinetics/pharmacodynamics variables....................54
Table 13 – List of PTs or Medications for CMQs/CDGs....................57

VERSION HISTORY

This Statistical Analysis Plan (SAP) for study EFC16459 is based on the Protocol Amendment 1 dated 20-May-2020. There are no major changes to the statistical analysis features in this SAP, but the changes to the statistical analysis features from the original protocol to Amendment 1 are described in Appendix 2.

The first participant was randomized on 2020-01-02.

1 INTRODUCTION

1.1 STUDY DESIGN

This study is a multi-center, 24-week treatment, parallel group, double-blind, randomized, placebo-controlled study to evaluate the use of dupilumab in participants with prurigo nodularis (PN) inadequately controlled on topical prescription therapies or when those therapies are not advisable. The study will assess the effect of dupilumab on itch improvement as well as its effect on PN lesions, on participants' health-related quality of life (HRQoL), anxiety and depression, sleep quality, skin pain, and overall health status.

After 2-4 weeks of screening, participants will be centrally randomized using a permuted block randomization schedule via Interactive Voice Response System/Interactive Web Response System (IVRS/IWRS) in a 1:1 randomization ratio to dupilumab 300 mg q2w or matching placebo. Randomization will be stratified by documented history of atopy (atopic or non-atopic), stable use of topical corticosteroids (TCS)/ topical calcineurin inhibitors (TCI) (yes or no), and country/territory code.

A total of approximately 150 participants will be randomized to two treatment arms (75 participants/arm). The number of participants with active mild AD upon study entry will represent up to 10% of the atopic participants. Both the atopic and the non-atopic PN populations will each be capped at 60% of the total enrolled population.

The study duration consists of the following periods:

- Screening period (2-4 weeks)
- Randomized IMP intervention period (24 weeks)
- Follow-up period (12 weeks)

1.2 OBJECTIVE AND ENDPOINTS

Table 1 - Objectives and endpoints

Objectives	Endpoints
**Primary**	
• To demonstrate the efficacy of dupilumab on itch response in participants with PN, inadequately controlled on topical prescription therapies or when those therapies are not advisable.	• Proportion of participants with improvement (reduction) in worst-itch numeric rating scale (WI-NRS) by ≥4 from baseline to Week 12.
**Secondary**	
• To demonstrate the efficacy of dupilumab on additional itch endpoints in participants with PN, inadequately controlled on topical prescription therapies or when those therapies are not advisable.	• Proportion of participants with improvement (reduction) in WI-NRS by ≥4 from baseline to Week 24 **[Key secondary endpoint]**.<br>• Time to onset of effect on pruritus as measured by proportion of participants with an improvement

Objectives	Endpoints
	(reduction) in WI-NRS by ≥4 from baseline during the 24-week treatment period.<br>• Change from baseline in WI-NRS at Week 24.<br>• Change from baseline in WI-NRS at Week 12.<br>• Percent change from baseline in WI-NRS at Week 24.<br>• Percent change from baseline in WI-NRS at Week 12.<br>• Percent change from baseline in WI-NRS at Week 4.<br>• Percent change from baseline in WI-NRS at Week 2.<br>• Percent change from baseline in WI-NRS over time until Week 24.<br>• Proportion of participants with WI-NRS reduction ≥4 at Week 4.<br>• Proportion of participants with WI-NRS reduction ≥4 over time until Week 24[a].<br>• Onset of action in change from baseline in WI-NRS (first p <0.05 difference from placebo in the daily WI-NRS that remains significant at subsequent measurements) until Week 12[b].
• To demonstrate efficacy of dupilumab on skin lesions of PN.	• Proportion of participants with Investigator's Global Assessment 0 or 1 score for PN-Stage (IGA PN-S) at Week 24 **[Key secondary endpoint]**.<br>• Proportion of participants with IGA PN-S 0 or 1 score at Week 12.<br>• Proportion of participants with IGA PN-S 0 or 1 score at Week 8[c].<br>• Proportion of participants with IGA PN-S 0 or 1 score at Week 4[c].<br>• Change from baseline in IGA PN-S score at Week 24.<br>• Change from baseline in IGA PN-S score at Week 12.<br>• Change from baseline in IGA PN-S score at Week 8.<br>• Change from baseline in IGA PN-S score at Week 4.<br>• Proportion of participants with Investigator's Global Assessment 0 or 1 score for PN-Activity (IGA PN-A) at Week 24.<br>• Proportion of participants with IGA PN-A 0 or 1 score at Week 12.<br>• Proportion of participants with IGA PN-A 0 or 1 score at Week 8.<br>• Proportion of participants with IGA PN-A 0 or 1 score at Week 4.
• To demonstrate the improvement in health-related quality of life (HRQoL).	• Change from baseline in HRQoL, as measured by Dermatology Life Quality Index (DLQI) to Week 24.<br>• Change from baseline in HRQoL, as measured by DLQI to Week 12.

Objectives	Endpoints
• To evaluate safety outcome measures.	• Percentage of participants experiencing treatment-emergent adverse events (TEAEs) or serious adverse events (SAEs) from baseline through Week 24.
• To evaluate immunogenicity of dupilumab.	• Incidence of treatment-emergent antidrug antibodies (ADA) against dupilumab over time.
**Tertiary/exploratory**	
• To demonstrate a reduction in the use of rescue medication and systemic immunosuppressant	• Use of high potency or superpotent TCS rescue medication through Week 24<br>• Use of systemic immunosuppressants through Week 24, constituting treatment failure
• To evaluate exploratory outcome measures	• Change from baseline in Hospital Anxiety and Depression Scale (HADS) total score to Week 24.<br>• Change from baseline in EQ5D-5L to Week 24.<br>• Change from baseline in Pain numeric rating scale (NRS) to Week 4, Week 8, Week 12, and Week 24, respectively.<br>• Change from baseline in Sleep NRS to Week 4, Week 8, Week 12, and Week 24, respectively.<br>• Missed school/work days through Week 24.<br>• Incidence of skin-infection TEAEs (excluding herpetic infections) through Week 24.
• To evaluate the efficacy of dupilumab on skin lesions using a modified PAS 5-item questionnaire	• Proportion of participants who achieve ≥75% healed lesions from Prurigo Activity Score (PAS) at Week 4, Week 8, Week 12, and Week 24, respectively.<br>• Change from baseline in exact number of lesions in representative area (as determined from PAS) at Week 4, Week 8, Week 12, and Week 24, respectively.
• To evaluate the efficacy of dupilumab on other PN endpoints	• Change from baseline in Participant Global Impression of Severity (PGIS) of PN to Week 4, Week 8, Week 12, and Week 24, respectively.<br>• Participant Global Impression of Change (PGIC) of PN at Week 4, Week 8, Week 12, and Week 24, respectively.
**PK**	
• To evaluate pharmacokinetic (PK) and pharmacodynamic (PD) outcome measures	• Serum functional dupilumab concentrations and PK profile.<br>• Pharmacodynamic response for selected biomarkers (total IgE).

a. The early onset for significant change in the proportion of responders with WI-NRS reduction ≥4 between dupilumab and placebo will be claimed at Week xx if the p-value for Week xx is <0.05 and the remaining timepoints are also statistically significant.

b. The early onset for significant change in WI-NRS from baseline between dupilumab and placebo will be claimed at Week xx if the p-value for Week xx is <0.05 and the remaining timepoints are also statistically significant.

c. The early onset for reaching IGA PN-S(0,1) between dupilumab and placebo will be claimed at Week xx if the p-value for Week xx is <0.05 and the remaining timepoints are also statistically significant.

1.2.1 Estimands

The primary estimands defined for main endpoints are summarized in below Table 2. More details are provided in Section 4.

Table 2 - Summary of primary estimand for main endpoints

Endpoint Category	Estimands			
	**Endpoint(s)**[a]	**Population**	**Intercurrent event(s) strategy and missing data handling**	**Population-level summary**
**Primary objective: To demonstrate the efficacy of dupilumab on itch response in participants with PN, inadequately controlled on topical prescription therapies or when those therapies are not advisable.**				
Primary endpoint	Proportion of participants with improvement (reduction) in worst-itch numeric rating scale (WI-NRS) by ≥4 from baseline to Week 12	ITT	The intercurrent events will be handled as follows:<br>• Discontinuation of study treatment before Week 12: Off-study treatment data up to Week 12 will be included in the analysis (treatment policy strategy).<br>• Taking selected prohibited medications/procedures and/or rescue medications[b] prior to Week 12: Participants will be considered as non-responders (composite strategy).<br>In addition, the missing data imputation rules are as follows:<br>• Having missing data at Week 12: Participants will be considered as non-responders.	CMH test adjusted by documented history of atopy (atopic or non-atopic), stable use of TCS/TCI (yes or no), region (countries combined), and baseline antidepressant use (yes or no).
**Secondary objective: To demonstrate the efficacy of dupilumab on additional itch endpoints in participants with PN, inadequately controlled on topical prescription therapies or when those therapies are not advisable.**				

Endpoint Category	Estimands			
	Endpoint(s)[a]	Population	Intercurrent event(s) strategy and missing data handling	Population-level summary
Secondary endpoint	Time to onset of effect on pruritus as measured by proportion of participants with an improvement (reduction) in WI-NRS by ≥4 from baseline during the 24-week treatment period	ITT	The intercurrent events will be handled as follows:<br>• Discontinuation of study treatment before Week 24: Off-study treatment data up to Week 24 will be included in the analysis (treatment policy strategy).<br>• Taking selected prohibited medications/procedures and/or rescue medications[b] prior to Week 24: Analyses will be censored at Week 24 (composite strategy).<br>In addition, the missing data imputation rules are as follows:<br>• Discontinuing the study follow-up before Week 24: Analyses will be censored at the time of last WI-NRS assessment.	This time-to-event endpoint will be analyzed using the Cox proportional hazards model, including intervention group, documented history of atopy (atopic or non-atopic), stable use of TCS/TCI (yes or no), region (countries combined), and baseline antidepressant use (yes or no). The hazards ratio, its 95% confidence interval and p-value will be reported. Kaplan-Meier curves will be also provided.

Endpoint Category	Estimands			
	**Endpoint(s)[a]**	**Population**	**Intercurrent event(s) strategy and missing data handling**	**Population-level summary**
Secondary endpoint	Change from baseline in WI-NRS at Week 24	ITT	The intercurrent events will be handled as follows:<br>• Discontinuing the study treatment: all data collected following schedule after treatment discontinuation will be used in the analysis (treatment policy strategy).<br>• Taking selected prohibited medications/procedures and/or rescue medications[b] prior to Week 24: data will be set to missing values after the medication usage, and the participant's worst postbaseline value on or before the time of the medication usage will be used to impute missing endpoint value (for participants whose postbaseline values are all missing, the participant's baseline will be used to impute the missing endpoint value) (hypothetical strategy)<br><br>In addition, the missing data imputation rules are as follows:<br>• After discontinuation of the study treatment due to lack of efficacy prior to Week 24: WOCF approach will be used to impute missing data if needed[2].<br>• After discontinuation of the study treatment due to reasons other than lack of efficacy prior to Week 24: multiple imputation (MI) approach will be used to impute missing endpoint value, and this multiple imputation will use all participants excluding participants who have taken the selected prohibited medications and/or rescue medications prior to Week 24 and excluding participants who discontinue due to lack of efficacy prior to Week 24.	ANCOVA model with intervention group, documented history of atopy (atopic or non-atopic), stable use of TCS/TCI (yes or no), region (countries combined), baseline antidepressant use (yes or no), and relevant baseline measurement as covariates is used. Statistical inference obtained from all imputed data by ANCOVA model will be combined using Rubin's rule.

a Additional secondary objectives/endpoints are not included in this table but would be handled with a similar strategy as the endpoint type (ie continuous, proportion, time-to-event) at other weeks

b Selected prohibited medications and/or rescue medications are listed in Table 4.

2 SAMPLE SIZE DETERMINATION

The primary endpoint is the proportion of participants with WI-NRS reduction of ≥4 from baseline to Week 12. By assuming the response rate is [redacted]% and [redacted]% in the placebo and dupilumab arms, respectively, 56 participants/arm will provide 90% power to detect the difference of [redacted]% between dupilumab and placebo with Fisher exact test at 2-sided level of 0.05. Assuming 15% drop out during the 12 weeks of treatment, the target is to randomize 75 participants/arm with a cap of up to 10% of participants in the atopic population having active mild AD.

The assumptions were based on the effect of dupilumab versus placebo in WI-NRS reduction ≥4 at Week 16 observed in participants with moderate to severe AD as seen in studies of AD-1334 (Solo1) and AD 1416 (Solo2).

Approximately 150 participants will be randomized to dupilumab or placebo in a 1:1 ratio with stratification factors of documented history of atopy (atopic or non-atopic), stable use of TCS/TCI (yes or no), and country/territory code. Both the atopic and the non-atopic PN population will be capped at 60% of the total enrolled population.

The sample size calculation was performed using SAS 9.4 power procedure.

3 ANALYSIS POPULATIONS

The following populations for analyses are defined:

Table 3 - Populations for analyses

Population	Description
Screened	All participants who sign the ICF.
Randomized	The randomized population includes all participants with a treatment kit number allocated and recorded in the interactive response technology (IRT) database, and regardless of whether the treatment kit was used or not.<br>Participants treated without being randomized will not be considered randomized and will not be included in any efficacy population.
Intent-to-treat (ITT)	All randomized participants analyzed according to the intervention group allocated by randomization regardless if treatment kit is used or not
Efficacy	The ITT population
Safety	All participants randomly assigned to study intervention and who take at least 1 dose of study intervention. Participants will be analyzed according to the intervention they actually received.<br>Randomized participants for whom it is unclear whether they took the study medication will be included in the safety population as randomized.<br>For participants who accidentally receive different treatment from the planned, the actual intervention allocation for as-treated analysis will be the dupilumab group.<br>The PD analyses will be performed on the safety population.
Pharmacokinetic (PK)	The PK population includes all participants in the safety population with at least one non-missing result for functional dupilumab concentration in serum after first dose of the study treatment. Participants will be analyzed according to the intervention actually received.
Antidrug antibody (ADA)	ADA population includes all participants in the safety population who have at least one non-missing ADA result after first dose of the study treatment. Participants will be analyzed according to the intervention actually received.

ADA: antidrug antibody; ICF: informed consent form; ITT: intent to treat; PD: pharmacodynamics: PK: pharmacokinetic.

Participants exposed to study intervention before or without being randomized will not be considered randomized and will not be included in any analysis population. The safety experience of these participants will be reported separately.

Randomized participants for whom it is unclear whether they took the study intervention will be considered as exposed and will be included in the safety population as randomized.

For any participant randomized more than once, only the data associated with the first randomization will be used in any analysis population. The safety experience associated with any later randomization will be reported separately.

For participants receiving more than one study intervention (placebo and dupilumab) during the study, the intervention group for as-treated analysis will be the dupilumab group.

If >10% participants are impacted by the COVID-19 pandemic, additional summaries by COVID-19 subgroups will be provided. Participants impacted by the COVID-19 pandemic are defined as randomized participants with any critical or major deviation related to COVID-19 or who permanently discontinued study intervention or study due to COVID-19.

4 STATISTICAL ANALYSES

4.1 GENERAL CONSIDERATIONS

In general, continuous data will be summarized using the number of observations available, mean, standard deviation (SD), median, minimum, and maximum. Categorical and ordinal data will be summarized using the count and percentage of participants.

The baseline value of efficacy parameters is defined as the last available value before randomization and prior to the first dose of study medication unless below eDiary data (WI-NRS, Pain-NRS and Sleep-NRS) score or otherwise specified.

The baseline for weekly average WI-NRS (Pain-NRS and Sleep-NRS) score is defined as the average of daily non-missing scores obtained during the 7 days prior to randomization.

The baseline value of the other parameters is defined as the last available value prior to the first dose of investigational medicinal product (IMP) if the participant is treated, or the last available value up to randomization if the participant is not exposed to IMP.

Observation period

The observation period will be divided into 4 segments:

- The **pre-treatment period** is defined as the period up to first IMP administration.
- The **treatment-emergent (TE) period** is defined as the period from the first IMP administration to the last IMP administration + 98 days. The treatment-emergent period includes the following 2 periods:
  - The **on-treatment period** is defined as the period from the first IMP administration to the last administration of the IMP + 14 days
  - The **residual treatment period** is defined as the period from the end of the on-treatment period to the end of the treatment-emergent period.
- The **post-treatment period** is defined as the period from the end of the treatment-emergent period.

The on-study observation period is defined as the time from start of intervention until the end of the study defined as the status date collected on e-CRF page “Completion of End of Study”.

4.2 PARTICIPANT DISPOSITIONS

The number (%) of participants included in each of the analysis populations listed in Table 3 will be summarized.

Screen failures are defined as participants who consent to participate in the study but are not subsequently randomized. The number (%) of screen failures and reasons for screen failures will be provided in the screened population.

The number (%) of participants in the following categories will be provided:

- Randomized participants
- Randomized but not exposed participants
- Randomized and exposed participants
- Participants who completed the study treatment period as per protocol
- Participants who did not complete the study treatment period as per protocol and discontinued study treatment prior to Week 12 by main reason for permanent intervention discontinuation including due to COVID-19
- Participants who did not complete the study treatment period as per protocol and discontinued study treatment prior to Week 24 by main reason for permanent intervention discontinuation including due to COVID-19
- Participants who completed the study period as per protocol
- Participants who did not complete the study period as per protocol and discontinued study by main reason for study discontinuation including due to COVID-19.
- Vital status at last study contact

The number (%) of exposed and not randomized participants will also be summarized.

In addition, the number (%) of participants screened, screened-failed, randomized, with permanent intervention discontinuation and with early study discontinuation will be provided by country and site.

Protocol deviations

Critical and major protocol deviations (automatic or manual) will be summarized in the randomized population and according to COVID-19 impact (i.e. deviations related to the COVID-19 pandemic and deviations not related to the COVID-19 pandemic). In addition, deviations potentially impacting the primary endpoint analysis will be summarized.

4.3 PRIMARY ENDPOINT(S) ANALYSIS

4.3.1 Definition of endpoint(s)

The primary endpoint for this study is the proportion of participants with improvement (reduction) in worst-itch numeric rating scale (WI-NRS) by ≥4 from baseline to Week 12.

WI-NRS is a patient-reported outcome (PRO) comprised of a single item rated on a scale from 0 (“No itch”) to 10 (“Worst imaginable itch”). Participants are asked to rate the intensity of their worst pruritus (itch) over the past 24 hours using this scale.

The weekly average WI-NRS score at each week, which is defined as the average of daily non-missing scores within the week window of each week (see Section 5.4), will be used for analyses.

For efficacy analysis, Table 4 presents the prohibited and rescue medications/procedures which will be considered as intercurrent events if the last column indicates as “Yes” and therefore be handled in the estimands for endpoints defined in Table 2.

Table 4 - Selected prohibited medications/procedures and rescue medications that impact on efficacy

Medication/procedure	Comment	Data to be set as non-responder after taking medication in the main statistical analysis (Yes/No) [a]/ Selection criteria
**Prohibited medications/procedures**		
Systemic immunosuppressive/immunomodulating drugs (eg, systemic corticosteroids, cyclosporine, mycophenolate-mofetil, interferon gamma, Janus kinase inhibitors, azathioprine, methotrexate, hydroxychloroquine, dapsone, sulfasalazine, colchine, etc)	IMP to be discontinued	Yes (CDG[b] Immunosuppressant drugs Narrow and SDG[b]Corticosteroids Narrow)
Other monoclonal antibodies (that are biological response modifiers).	IMP to be discontinued	Yes (SDG[b] SDGMonoclonal antibodies Narrow)
Phototherapy, including tanning beds.	IMP to be discontinued	Yes (CMQ[b] HLT Phototherapies, CMQ[b] Tanning_single PT)
Naltrexone or other opioid antagonist	IMP to be discontinued	Yes (SDG[b] Analgesia producing opioids NARROW)
Gabapentin, pregabalin, and thalidomide	IMP to be discontinued	Yes (CDG[b] Gabapentin mono and multi ingredients, Pregabalin mono and multi ingredients, or Thalidomide mono and multi ingredients)
Paroxetine, fluvoxamine or other SSRIs.	No IMP discontinuation, see requirement in the footnote	Yes[c,d] (CDG[b] N06AB selective serotonin reuptake inhibitors)
SNRIs.	No IMP discontinuation, see requirement in the footnote	Yes[c,d] (CDG[b] serotonin and norepinephrine reuptake inhibitors)
Amitriptyline or other tricyclic or tetracyclic antidepressants	No IMP discontinuation, see requirement in the footnote	Yes[c,d] (CDG[b] N06AA non-selective monoamine reuptake inhibitors)
Intralesional corticosteroid injections and cryotherapy.	No IMP discontinuation	Yes[d] (CMQ[b] Injection_single pt, cryotherapy or skin cryotherapy PT)
Sedating antihistamine	No IMP discontinuation	Yes[d,e] (CDG[b] sedating antihistamines)
Non-sedating antihistamine if used specifically for the treatment of itch secondary to AD or PN	No IMP discontinuation	Yes[d,e] (CDG[b] non-sedating antihistamines)

Medication/procedure	Comment	Data to be set as non-responder after taking medication in the main statistical analysis (Yes/No) [a]/ Selection criteria
**Rescue medications**		
Dermatological preparations of high potency or superpotent TCS and TCI	No IMP discontinuation	Yes[d] (CDG[b] calcineurin inhibitors and corticosteroids narrow, Ticked 'RESCUE THERAPY' in CRF page)

a. When yes, the estimand for the intercurrent event handling strategy will be as follows: hypothetical for continuous endpoints, and composite for responder and time-to-event endpoints. When no, a treatment policy strategy will be applied.
b. CDG=company drug groupings, CMQ=company MedDRA query, SDG= Standardized drug groupings.
c. Only if the antidepressant is initiated during the study or its dose is increased from baseline provided that medication was taken at least 3 months prior to screening.
d. As per medical adjudication, see Appendix 7 for details.
e. Only if the medication is initiated at Week 12 or Week 24, or the dose is increased from Week 11 to Week 12 or from Week 23 to Week 24.

Blinded review of prohibited/rescue treatment (medication or procedure) based on Table 4 and a pre-specified algorithm (Appendix 7) will be implemented before database locks by considering the type of medication or procedure, indication, timing, frequency and the potential impact of the use of the prohibited medications/procedures and rescue medications.

4.3.2 Main analytical approach

The primary estimand for the primary endpoint is the treatment policy/composite approach as defined in Table 2.

The primary analysis population for the efficacy endpoints will be the ITT population.

The following null hypothesis H0 and alternative hypothesis H1 will be tested for dupilumab against placebo:

- H0: No treatment difference between dupilumab and placebo.
- H1: There is a treatment difference between dupilumab and placebo

The primary analysis will be conducted by using Cochran–Mantel–Haenszel test (CMH) test adjusted by documented history of atopy (atopic or non-atopic), stable use of TCS/TCI (yes or no), region, and baseline anti-depressant use (yes or no). Comparisons of the response rates between dupilumab and placebo will be derived. In addition, odds ratio and response rate difference as well as the corresponding 95% confidence intervals (CI) will be provided along with the p-values.

As defined in Table 2 for participants discontinuing the study treatment before Week 12, their off-study treatment values measured up to Week 12 will be included in the analysis. Participants taking selected prohibited medications/procedures and/or rescue medications (see Table 4) prior to Week 12 or having missing data at Week 12 will be considered non-responders.

4.3.3 Sensitivity analysis

For the primary estimand for the primary endpoint, no sensitivity analysis will be performed. However, two supplementary analyses will be performed as described in the section below.

4.3.4 Supplementary analyses

The following supplementary analyses will be performed:

As-observed analysis (including all data after taking selected prohibited and/or rescue medications)

The data collected after taking all prohibited medications and/or rescue medications will be included in the supplementary analysis to evaluate the robustness of the primary analysis results with respect to the method of handling data while taking selected prohibited medications (eg, treatment policy strategy). In addition, for participants discontinuing the study treatment before Week 12, their off-study treatment values measured up to Week 12 will be included in the analysis. The patients having missing data at Week 12 regardless of reason(s) will be considered non-responders at that timepoint.

Hybrid method analysis (the worst-observation carried forward (WOCF) and multiple imputation (MI))

In the primary analysis of change from baseline in WI-NRS (continuous variable) at Week 12, the hybrid method of the WOCF and MI will be used (see Section 4.4.2). Similar to the continuous variable, the same imputation method will be used in the analysis of the proportion of participants with improvement (reduction) in WI-NRS by ≥4 from baseline to Week 12, which is consistent for the intercurrent event strategy and missing data handling in the binary variables and continuous variable. That is, after the imputation of continuous WI-NRS data at Week 12 using the hybrid method of the WOCF and MI (see Section 4.4.2), responders will be defined as patients with improvement in WI-NRS by ≥4 from baseline to Week 12 in each of the imputed datasets with about 40 imputations, and then the CMH test adjusted by documented history of atopy (atopic or non-atopic), stable use of TCS/TCI (yes or no), region, and baseline anti-depressant use (yes or no) will be used. Statistical inference obtained from all imputed data will be combined using Rubin’s rule.

4.3.5 Subgroup analyses

To assess the homogeneity of the treatment effect across various subgroups, analyses will be performed on the primary endpoint across the following subgroups (categories with fewer than 5 participants may be combined with other categories):

- Participants without a current diagnosis of AD
- Age group (<65, ≥65 years)
- Gender (Male, Female)
- Region

- Territory
- Race (Caucasian/White, Black/of African descent, Asian/Oriental, Others)
- Ethnicity (Hispanic or Latino, Not Hispanic or Latino)
- Baseline weight (<60, ≥60- < 90, ≥ 90 kg)
- Baseline BMI (<25, ≥25- <30, ≥30 kg/m^2)
- History of atopy (atopic or non-atopic)
- Stable use of TCS/TCI (yes or no)
- Antidepressant use (yes or no) at baseline
- HIV (positive vs. negative)
- Baseline IGA PN-S moderate vs severe (3 vs. 4)

To test the interaction between intervention and subgroup factor, a logistic regression model incorporating subgroup-by-treatment interaction will be built for each subgroup factor except the subgroup of participants without a current diagnosis of AD (very few AD participants will be excluded). The model will include all the covariates in the main statistical model plus the subgroup variable and the subgroup-by-treatment interaction. A p-value for the test of interaction will be provided.

In each subgroup, the treatment effects for the primary endpoint will be provided, as well as the corresponding 95% CI, using the same method as applied to the primary analysis. Forest plots will be provided.

4.4 SECONDARY ENDPOINT(S) ANALYSIS

The weekly average Pain-NRS or Sleep-NRS score at each week, which is defined as the average of non-missing daily scores within the week window of each week (see Section 5.4), will be used for analyses.

4.4.1 Key/Confirmatory secondary endpoint(s)

4.4.1.1 Definition of endpoint(s)

There are two key secondary endpoints:

- Proportion of participants with improvement (reduction) in WI-NRS by ≥4 from baseline to Week 24, and
- Proportion of participants with IGA PN-S 0 or 1 score at Week 24.

Investigator's global assessment for prurigo nodularis (IGA PN)

The IGA PN is a clinician-reported outcome (ClinRO) that allows clinicians to assess the activity of PN (IGA PN-A) using a 5-point scale from 0 (clear) to 4 (severe); and the stage of the disease (IGA PN-S) using a 5-point scale from 0 (clear) to 4 (severe).

4.4.1.2 Main analytical approach

For both key secondary efficacy endpoints, the analysis will be conducted by using CMH test adjusted by documented history of atopy (atopic or non-atopic), stable use of TCS/TCI (yes or no), region, and baseline anti-depressant use (yes or no). The same estimand, i.e., intercurrent event strategy and missing data handling method, as the primary endpoint, by replacing Week 12 with Week 24, is used.

4.4.2 Supportive secondary endpoint(s)

The other secondary endpoints are as follows:

- Proportion of participants with WI-NRS reduction ≥4 over time until Week 24.
- Proportion of participants with WI-NRS reduction ≥4 at Week 4
- Proportion of participants with IGA PN-S 0 or 1 score at Week 12
- Proportion of participants with IGA PN-S 0 or 1 score at Week 8
- Proportion of participants with IGA PN-S 0 or 1 score at Week 4
- Proportion of participants with IGA PN-A 0 or 1 score at Week 24
- Proportion of participants with IGA PN-A 0 or 1 score at Week 12
- Proportion of participants with IGA PN-A 0 or 1 score at Week 8
- Proportion of participants with IGA PN-A 0 or 1 score at Week 4
- Time to onset of effect on pruritus as measured by proportion of participants with an improvement (reduction) in WI-NRS by ≥4 from baseline during the 24-week treatment period
- Change from baseline in WI-NRS at Week 24
- Change from baseline in WI-NRS at Week 12
- Percent change from baseline in WI-NRS at Week 24
- Percent change from baseline in WI-NRS at Week 12
- Percent change from baseline in WI-NRS at Week 4
- Percent change from baseline in WI-NRS at Week 2
- Percent change from baseline in WI-NRS over time until Week 24.

- Onset of action in change from baseline in WI-NRS (first $p < 0.05$ difference from placebo in the daily WI-NRS that remains significant at subsequent measurements) until Week 12
- Change from baseline in IGA PN-S score at Week 24
- Change from baseline in IGA PN-S score at Week 12
- Change from baseline in IGA PN-S score at Week 8
- Change from baseline in IGA PN-S score at Week 4
- Change from baseline in HRQoL, as measured by Dermatology Life Quality Index (DLQI) to Week 24
- Change from baseline in HRQoL, as measured by Dermatology Life Quality Index (DLQI) to Week 12

Dermatology life quality index (DLQI)

The DLQI is a PRO developed to measure dermatology-specific HRQoL in adult participants. The instrument comprises 10 items assessing the impact of skin disease on participants' HRQoL over the previous week. The items cover symptoms, leisure activities, work/school or holiday time, personal relationships including intimate, the side effects of treatment, and emotional reactions to having a skin disease. It is a validated questionnaire used in clinical practice and clinical trials. Response scale is a 4-point Likert scale (0 = "not at all" and 3 = "very much") for nine items. The remaining one item about work/studying asks whether work/study has been prevented and then (if "No") to what degree the skin condition has been a problem at work/study; the item is rated on a 3-point Likert scale ("Not at all" to "A lot"). Overall scoring ranges from 0 to 30, with a high score indicative of a poor HRQoL.

Time-to-event secondary efficacy endpoint

Time to first onset of effect on pruritus defined as an improvement (reduction) in WI-NRS by ≥4 from baseline during the 24-week treatment period will be analyzed using the Cox proportional hazards model, including treatment, documented history of atopy (atopic or non-atopic), stable use of TCS/TCI (yes or no), region, and baseline anti-depressant use (yes or no). The hazards ratio, its 95% confidence interval and p-value will be reported. Kaplan-Meier curves will be also provided.

For time to first WI-NRS response (WI-NRS by ≥4 from baseline), participants who receive selected prohibited medications and/or rescue medications (see Table 4), data prior to start of the medications will be used, but after medication start, the participants' data will not be used and they will be censored at Week 24 (i.e. Day 172). For other participants, all available data up to Week 24 (i.e. Day 172) including those collected during the off-treatment period will be used. Participants without events will be censored at Day 172 or their last WI-NRS assessment date if discontinued from the study, whichever is earlier.

Secondary efficacy endpoints that measure binary responses

Secondary efficacy endpoints that measure binary responses will be analyzed in the same fashion as the primary endpoint using primary statistical model.

Continuous secondary efficacy endpoints

The primary estimand for continuous secondary efficacy endpoints is defined in Table 2 and will be analyzed using an analysis of covariance (ANCOVA) model with intervention group, documented history of atopy (atopic or non-atopic), stable use of TCS/TCI (yes or no), region (countries combined), baseline antidepressant use (yes or no), and relevant baseline measurement as covariate, with intercurrent event strategy and missing data handling as defined as in Table 2. Specifically, data of participants taking selected prohibited medications/procedures and/or rescue medications will be set to missing after the medication usage, and the worst postbaseline value on or before the time of the medication usage will be used to impute missing endpoint value (for participants whose postbaseline values are all missing, the baseline will be used to impute). Participants who discontinue the treatment prematurely are encouraged to follow the planned clinical visits and in those participants who did not take the selected prohibited medications/procedures and/or rescue medications, all data collected after treatment discontinuation will be used in the analysis. For these participants, missing data may still happen despite all efforts have been tried to collect the data after treatment discontinuation. For participants who discontinue due to lack of efficacy, all data collected after discontinuation will be used in the analysis, and a WOCF approach will be used to impute missing data if needed. For participants who discontinued not due to lack of efficacy, a multiple imputation (MI) approach will be used to impute missing endpoint value, and this MI method will use all participants excluding participants who have taken the selected prohibited medications/procedures and/or rescue medications prior to timepoint of endpoint of interest and excluding patients who discontinue due to lack of efficacy.

Each of the imputed complete data will be analyzed by fitting an ANCOVA model as described above. The imputation number will be about 40. Statistical inference obtained from all imputed data will be combined using Rubin's rule. Descriptive statistics including number of participants, mean, standard error, and least squares (LS) mean changes (and standard error) will be provided. In addition, difference of the dupilumab group against placebo in LS means and the corresponding 95% confidence intervals (CI) will be provided along with the p-values.

See Section 5.5 for the sample SAS code for the imputation and the analysis.

4.5 TERTIARY/EXPLORATORY ENDPOINT(S) ANALYSIS

4.5.1 Definition of endpoint(s)

The exploratory endpoints are as follows:

- Use of high potency or superpotent TCS rescue medication through Week 24
- Use of systemic immunosuppressant through Week 24, constituting treatment failure

- Change from baseline in Hospital Anxiety and Depression Scale (HADS) total score to Week 24
- Change from baseline in EQ-5D-5L Single Index score to Week 24
- Change from baseline in EQ-5D visual analog scale (VAS)to Week 24
- Change from baseline in Pain-NRS to Week 4, Week 8, Week 12, and Week 24, respectively
- Change from baseline in Sleep-NRS to Week 4, Week 8, Week 12, and Week 24, respectively
- Missed school/work days through Week 24
- Incidence of skin-infection TEAEs (excluding herpetic infections) through Week 24
- Proportion of participants who achieve ≥75% healed lesions from PAS at Week 4, Week 8, Week 12, and Week 24, respectively
- Change from baseline in exact number of lesions in representative area (as determined from PAS) at Week 4, Week 8, Week 12, and Week 24, respectively
- Change from baseline in Participant Global Impression of Severity (PGIS) of PN to Week 4, Week 8, Week 12, and Week 24 respectively
- Proportion of participants with PGIS score of "none" at Week 4, Week 8, Week 12, and Week 24 respectively
- Proportion of participants with PGIS score of "non" or "mild" at Week 4, Week 8, Week 12, and Week 24 respectively
- Participant Global Impression of Change (PGIC) of PN at Week 4, Week 8, Week 12, and Week 24, respectively
- Proportion of participants with PGIC score of "very much better" at Week 4, Week 8, Week 12, and Week 24 respectively
- Proportion of participants with PGIC score of "very much better" or "moderately better" at Week 4, Week 8, Week 12, and Week 24 respectively.

Hospital anxiety and depression scale (HADS)

The HADS is a PRO instrument for screening anxiety and depression in non-psychiatric populations; repeated administration also provides information about changes to a participant's emotional state. The HADS consists of 14 items, 7 each for anxiety and depression symptoms; possible scores range from 0 to 21 for each subscale. The following cut-off scores are recommended for both subscales:

- 0 to 7: normal
- 8 to 10: borderline abnormal (borderline case)
- 11 to 21: abnormal

Euroqol 5 dimensions questionnaire (EQ-5D)

The Euroqol-5 dimensions (EQ-5D) is a standardized PRO measure of health status developed by the EuroQOL Group in order to provide a simple, generic measure of health for clinical and economic appraisal. The EQ-5D consists of 2 parts: the descriptive system and the EQ visual analog scale (VAS). The EQ-5D descriptive system comprises the following 5 dimensions: mobility, self-care, usual activities, pain/discomfort, and anxiety/depression. Each dimension has 5 levels of perceived problems: “no problem”, “slight problems”, “moderate problems”, “severe problems” and “inability to do the activity”. The respondent is asked to indicate his/her health state by ticking (or placing a cross) in the box against the most appropriate statement in each of the 5 dimensions; this results in a 1-digit number expressing the level for that dimension. The digits for 5 dimensions can be combined in a 5-digit number describing the respondent’s health state. The EQ VAS records the respondent’s self-rated health on a vertical VAS where the endpoints are labeled “best imaginable health state (100)” and “worst imaginable health state (0)”. This information can be used as a quantitative measure of health outcome as judged by the individual respondents.

Pain and sleep numeric rating scales (Pain-NRS, Sleep-NRS)

Participants will be asked to rate their worst skin pain in the past 24 hours using a 0 to 10 NRS, with 0 = No pain to 10 = Worst pain possible.

In addition, participants will be asked to rate their sleep quality on their past night upon awakening, using a 0 to 10 NRS, with 0 = Worst possible sleep and 10 = Best possible sleep.

Participants will complete the skin pain NRS and sleep quality NRS once a day.

Prurigo activity score (PAS)

The PAS is a clinician-reported outcome (ClinRO) measurement. The original PAS questionnaire Version 0.9 consists of 7 items, developed by expert clinicians in PN.

A 5-item simplified version of the PAS will be used in the current study. Item 4 (exact number of pruriginous lesions in representative area) and Item 5b (percentage of healed prurigo lesions in all pruriginous lesions) will be used for analyses. In addition, Item 2 (number of prurigo lesions) may be used for analysis too.

Participant Global Impression of Change of disease (PGIC) and Participant Global Impression of Severity (PGIS)

The PGIC is a one-item questionnaire that asks participants to provide the overall self-assessment of change in their PN overall on a 7-point scale, compared to just before participant started taking the study injection. Response choices are: 0 = “Very much better”, 1 = “Moderately better”, 2 = “A little better”, 3 = “No change”, 4 = “A little worse”, 5 = “Moderately worse”, 6 = “Very much worse”.

The PGIS is a one-item questionnaire that asks participants to provide the overall self-assessment of their disease severity on a 4-point scale for the past week. Response choices are: 1 = “none”, 2 = “Mild”, 3 = “Moderate”, 4 = “Severe”.

Missed school/work days

Participants who are employed or enrolled in school will be asked to report the number of sick leave/missed school days since the last study assessment.

Photography substudy

Participants in selected sites who decide to participate in this substudy need to provide separate consent. One or several lesions will be photographed at baseline. The same lesions will be photographed at subsequent visits to evaluate their progression.

4.5.2 Main analytical approach

Exploratory efficacy endpoints will be analyzed using the same methodology as secondary efficacy for similar data (continuous or proportion) except the endpoints below.

- Use of high potency or superpotent TCS rescue medication through Week 24
- Use of systemic immunosuppressant through Week 24, constituting treatment failure
- Missed school/work days through Week 24
- Incidence of skin-infection TEAEs (excluding herpetic infections) through Week 24

The number of participants and percentage with the events for these endpoints will be provided in observed cases. In addition, the time (week) to first event for those participants will also be analyzed using Kaplan-Meier method and the duration of the rescue medication use may be summarized if applicable.

An additional exploratory endpoint of time to first select prohibited/rescue medication that impact efficacy will be provided. This includes medications where WOCF will be applied (see Table 4).

4.6 MULTIPLICITY ISSUES

The multiplicity procedure is proposed to control the overall type-I error rate for testing the primary and selected secondary endpoints. The overall alpha is 0.05. The comparisons with placebo will be tested based on the hierarchical order below at 2-sided $\alpha = 0.05$:

Primary efficacy endpoints:

- Proportion of participants with improvement (reduction) in WI-NRS by ≥4 from baseline to Week 12.

Secondary efficacy endpoints:

- Proportion of participants with improvement (reduction) in WI-NRS by ≥4 from baseline to Week 24
- Proportion of participants with IGA PN-S 0 or 1 score at Week 24

Other secondary efficacy endpoints

- Percent change from baseline in WI-NRS at Week 24
- Proportion of participants with IGA PN-S 0 or 1 score at Week 12
- Change from baseline in HRQoL, as measured by Dermatology Life Quality Index (DLQI) to Week 24
- Change from baseline in Pain-NRS to Week 24
- Change from baseline in Sleep-NRS to Week 24
- Change from baseline in Hospital Anxiety and Depression Scale (HADS) total score to Week 24

The study is considered positive when the primary endpoint achieves statistical significance.

4.7 SAFETY ANALYSES

All safety analyses will be performed on the safety population as defined in Section 3, unless otherwise specified, using the following common rules:

- The analysis of the safety variables will be descriptive, and no testing is planned.
- Safety data in participants who do not belong to the safety population (e.g., exposed but not randomized) will be provided separately.

4.7.1 Extent of exposure

The extent of IMP exposure will be assessed by the duration of IMP exposure and compliance and summarized within the safety population.

Duration of IMP exposure

Duration of IMP exposure is defined as last dose date – first dose date + 14 days, regardless of unplanned intermittent discontinuations. Duration of IMP exposure will be summarized descriptively as a quantitative variable (number, mean, SD, median, minimum, and maximum). In addition, duration of treatment exposure will also be summarized categorically by numbers and percentages for each of the following categories and cumulatively according to these categories:

- >0 and ≤2 weeks
- >2 and ≤4 weeks
- >4 and ≤8 weeks
- >8 and ≤12 weeks

- >12 and ≤16 weeks
- >16 and ≤20 weeks
- >20 and ≤24 weeks
- >24 weeks and ≤ 24 weeks + 3 days
- > 24 weeks + 3 days

Additionally, the cumulative duration of treatment exposure will be provided, defined as the sum of the duration of treatment exposure for all participants, and will be expressed in participant years.

Treatment compliance

A given administration will be considered noncompliant if the participant did not take the planned dose as required by the protocol. No imputation will be made for participants with missing or incomplete data.

Percentage of treatment compliance for a participant will be defined as the number of administrations that the participant was compliant divided by the total number of administrations that the participant was planned to take from the first administration of IMP up to the actual last administration of IMP.

Treatment compliance will be summarized quantitatively and categorically: <80%, ≥80%.

Cases of overdose (defined as at least twice the intended dose during an interval of less than 11 days) will constitute SAEs and will be listed as such.

4.7.2 Adverse events

General common rules for adverse events

All adverse events (AEs) will be coded to a lower-level term (LLT), preferred term (PT), high-level term (HLT), high-level group term (HLGT), and associated primary system organ class (SOC) using the Medical Dictionary for Regulatory Activities (MedDRA) version currently in effect at Sanofi at the time of database lock.

The AEs will be analyzed in the following 3 categories:

- Pre-treatment AEs: AEs that developed, worsened or became serious during the pre-treatment period.
- Treatment-emergent adverse events (TEAE)s: AEs that developed, worsened or became serious during the treatment-emergent period
- Post-treatment AEs: AEs that developed, worsened or became serious during the post-treatment period

Similarly, the deaths will be analyzed in the pre-treatment, treatment-emergent and post-treatment periods.

The primary focus of AE reporting will be on TEAEs. Pre-treatment and post-treatment AEs will be described separately.

An AE with incomplete or missing date/time of onset (occurrence, worsening, or becoming serious) will be classified as a TEAE unless there is definitive information to determine it is a pre-treatment or a post-treatment AE.

If the assessment of the relationship to IMP is missing for an AE, this AE will be assumed as related to IMP. If the severity is missing for 1 of the treatment-emergent occurrences of an AE, the severity will be imputed with the maximal severity of the other occurrences. If the severity is missing for all the occurrences, the severity will be left as missing.

Multiple occurrences of the same event in the same participant will be counted only once in the tables within a treatment phase.

The AE tables will be sorted as indicated in Table 5.

Table 5 - Sorting of AE tables

AE presentation	Sorting rules
SOC, HLGT, HLT and PT	By the internationally agreed SOC order and by alphabetic order of HLGTs, HLTs and PTs.
SOC, HLT and PT	By the internationally agreed SOC order and by alphabetic order of HLTs and PTs.
SOC and PT	By the internationally agreed SOC order and decreasing frequency of PTs[a, b]
SMQ/CMQ and PT	By decreasing frequency of SMQs/CMQs and PTs[a]
PT	By decreasing frequency of PTs[a]

a Sorting will be based on the SAR231893 dupilumab group
b The table of all TEAEs presented by SOC and PT will define the presentation order for all other tables (eg, treatment-emergent SAE) presented by SOC and PT, unless otherwise specified.

Analysis of all adverse events

The overview of TEAE with the details below will be generated:

- Any TEAE
- Any treatment emergent SAE
- TEAE leading to death
- Any TEAE leading to permanent intervention discontinuation

The AE summaries of Table 6 will be generated with number (%) of participants experiencing at least one event.

Table 6 - Analyses of adverse events

Type of AE	MedDRA levels
All TEAE	Primary SOC, HLGT, HLT and PT
	Primary SOC and PT
	PT
	Primary and secondary SOC, HLGT, HLT and PT
Common TEAE (≥2% and 5% in any group)	Primary SOC and PT
TEAE related to IMP as per Investigator's judgment	Primary SOC, HLGT, HLT and PT
	Primary SOC and PT
TEAE by maximal intensity	Primary SOC and PT
Treatment emergent SAE	Primary SOC, HLGT, HLT and PT
	Primary SOC and PT
Treatment emergent SAE related to IMP as per Investigator's judgment	Primary SOC, HLGT, HLT and PT
TEAE leading to permanent intervention discontinuation	Primary SOC, HLGT, HLT and PT
	Primary SOC and PT
TEAE leading to death (death as an outcome of the AE as reported by the Investigator in the AE page)	Primary SOC, HLGT, HLT and PT
Pretreatment AE	Overview[a]
	Primary SOC and PT

a Will include the following AE categories: any AEs, any serious AEs, any AEs leading to death, any AEs leading to permanent intervention discontinuation

Analysis of deaths

In addition to the analyses of deaths included in Table 5 the number (%) of participants in the following categories will be provided:

- Deaths during the treatment-emergent and post-treatment periods
- Deaths in non-randomized or randomized but not treated participants

Analysis of adverse events of special interest (AESIs) and other AEs of interest

Adverse events of special interest (AESIs) and other AEs of interest will be selected for analyses as indicated in Table 7. Number (%) of participants experiencing at least one event will be provided for each event of interest. Tables will be sorted as indicated in Table 5.

Table 7 - Selections for AESIs and other AEs of interest

AE Grouping	Criteria
**AESI**	
Anaphylactic reaction	Anaphylactic reaction algorithmic approach (Introductory Guide for Standardised MedDRA Queries (SMQs) Version 18.1): includes anaphylactic reaction narrow SMQ (20000021) terms and programmatic identification of cases based on occurrence of at least two preferred terms meeting the algorithm criteria occurring within 24 hours of each other. The latter cases identified using the algorithm will undergo blinded medical review taking into account the timing of events relative to each other and to IMP administration for final determination of an anaphylactic reaction or not.
Systemic hypersensitivity reactions	SMQ [20000214] hypersensitivity narrow search and [AE corrective treatment/therapy='Y' or Action taken with IMP='Drug withdrawn' or Action taken with IMP='Drug interrupted'] followed by blinded medical review (documented process) for selection of relevant systemic hypersensitivity events
Helminthic infections	CMQ10544 based on HLGT as “Helminthic disorder”
Any severe type of conjunctivitis	CMQ10498 based on PTs (See Section 5.5) [a] and “Severe” ticked in Adverse Events eCRF page
Any severe type of blepharitis	CMQ10497 based on HLT as “Lid, lash and lacrimal infections, irritations and inflammations” and “Severe” ticked in Adverse Events eCRF page
Keratitis	CMQ10642 based on the following PTs [keratitis, allergic keratitis, ulcerative keratitis, atopic keratoconjunctivitis, herpes ophthalmic, ophthalmic herpes simplex, corneal infection][a]
Clinically symptomatic eosinophilia (or eosinophilia associated with clinical symptoms)[b]	CMQ10641 based on HLT = Eosinophilic disorders or PT=Eosinophil count increased
Pregnancy of a female participants entered in a study as well as pregnancy occurring in a female partner of a male participant entered in a study with IMP/NIMP	“Pregnancy” or “Partner Pregnancy” checked on the Pregnancy eCRF page as reported by the Investigator
Significant ALT elevation	ALT >5 x ULN in participants with baseline ALT ≤2 x ULN;<br>OR ALT >8 x ULN if baseline ALT >2 x ULN
Symptomatic overdose with IMP	Symptomatic Overdose is answered Yes, with Overdose of IMP answered Yes on AE eCRF.
Symptomatic overdose with NIMP	Symptomatic Overdose is answered Yes, with Overdose of NIMP answered Yes on AE eCRF.

AE Grouping	Criteria
**Other selected AE Grouping**	
Serious injection site reactions or severe injection site reactions that last longer than 24 hours	HLT = ‘Injection site reaction’ and either with serious status, or with severe status and (AE end date/time - AE start date/time) ≥ 24 hours or ongoing
Severe or serious infection	Primary SOC = ‘Infections and infestations’ and with severe or serious status
Drug-related hepatic disorder	SMQ [20000006] Drug-related hepatic disorders- narrow
Injection site reaction	HLT = ‘Injection site reaction’
Malignancy	SMQ [20000091]- Malignant or unspecified tumors narrow
Suicidal behavior	CMQ10639 based on the following PTs [Completed suicide, Suicidal ideation, Depression suicidal, Suicidal behavior, Suicide attempt][a]
Conjunctivitis (narrow)	CMQ10644 based on the following PTs [Conjunctivitis, Conjunctivitis allergic, Conjunctivitis bacterial, Conjunctivitis viral, Atopic keratoconjunctivitis][a]
Conjunctivitis (broad)	CMQ10645 based on the following PTs [Conjunctivitis, Conjunctivitis allergic, Conjunctivitis bacterial, Conjunctivitis viral, Atopic keratoconjunctivitis, Blepharitis, Dry eye, Eye irritation, Eye pruritus, Lacrimation increased, Eye discharge, Foreign body sensation in eyes, Photophobia, Xerophthalmia, Ocular hyperaemia, Conjunctival hyperaemia][a]
Conjunctivitis (FDA)	CMQ10643 based on the following PTs [Conjunctivitis, Conjunctivitis allergic, Conjunctivitis bacterial, Conjunctivitis viral, Eye irritation, Eye inflammation, Giant papillary conjunctivitis][a]

a The list of terms may be adjusted according to MedDRA version changes

b All cases of Eosinophilia will be included in the analysis, where cases associated with clinical symptoms will be further described in the CSR

The following summaries will be provided:

- All TEAEs, by selected standardized MedDRA query (SMQ)/Customized MedDRA query (CMQ) and PT or by laboratory values (as in alanine aminotransferase (ALT) elevation), showing the number (%) of participants with at least 1 PT,
- For each AESI and other selected AE groupings,
  - Number (%) of participants with any specific TEAE
  - Number (%) of participants with any specific serious AE (regardless of treatment emergent status)
  - Number (%) of participants with any specific treatment emergent serious AE
  - Number (%) of participants with any specific AE leading to death
  - Number (%) of participants with any specific TEAE leading to permanent study drug discontinuation

  - Number (%) of participants with any specific TEAE related to IMP reported by Investigator
  - Number (%) of participants with any specific TEAE by maximum intensity, corrective treatment, and final outcome
  - Number of any specific TEAE adjusted by the exposure duration
  - Number of participants with any specific TEAE adjusted by the exposure duration at risk. For each specific TEAE, Kaplan-Meier estimates of cumulative incidence at Week 12, and 24 and K-M plot may be provided to depict the course of onset over time if the number of events is large enough.
  - Number (%) of participants with injection site reactions by IMP injection.
  - Number (%) of participants with different number of injection site reactions.
- In addition, AESIs reported by the Investigator in eCRF will be summarized separately.

4.7.3 Additional safety assessments

4.7.3.1 Laboratory variables, vital signs and electrocardiograms (ECGs)

The following laboratory variables, vital signs and electrocardiogram (ECG) variables will be analyzed. They will be converted into standard international units.

- Hematology:
  - Red blood cells and platelets and coagulation: hemoglobin, hematocrit, red blood cell count, platelet count
  - White blood cells: white blood cell count, neutrophils, lymphocytes, monocytes, basophils, eosinophils
- Clinical chemistry:
  - Metabolism: glucose, total cholesterol, total protein, creatine phosphokinase
  - Electrolytes: sodium, potassium, chloride, calcium, bicarbonate
  - Renal function: creatinine, blood urea nitrogen, uric acid
  - Liver function: alanine aminotransferase, aspartate aminotransferase, alkaline phosphatase, lactate dehydrogenase, total bilirubin, albumin
  - Pregnancy test: Serum β-human chorionic gonadotropin (all female participants) will be performed at screening (V1) in women of childbearing potential, and a urine pregnancy test will be performed at V2 and every 4 weeks thereafter.
  - Hepatitis screen: hepatitis B surface antigen (HBs Ag), hepatitis B surface antibody (HBs Ab), hepatitis B core antibody (HBc Ab), hepatitis C virus antibodies (HCV Ab) will be tested at screening (V1). In case of results showing HBs Ag (negative) and HBc Ab (positive), an hepatitis B virus (HBV) deoxyribonucleic acid (DNA) testing will be performed and should be confirmed negative prior to randomization. In case of

results showing HCV Ab (positive), an HCV ribonucleic acid (RNA) testing will be performed and should be confirmed negative prior to randomization.

- HIV screen: Anti-HIV-1 and HIV-2 antibodies will be tested at Visit 1.
- TB test (performed locally if required and results noted in the eCRF).

- Urinalysis:
  - Urinalysis will include specific gravity, pH, glucose, ketones, blood, protein, nitrate, leukocyte esterase, urobilinogen and bilirubin. In case the urine dipstick test result is abnormal, a urine sample should be sent into the central laboratory for microscopic analysis. Creatinine and leukotriene are tested by the central laboratory.
- Vital signs: pulse rate (beats per minute), systolic and diastolic blood pressure (mmHg) in a semi-supine or sitting position after 5 minutes, weight, respiratory rate (breaths per minute), temperature (degrees Celsius) and height (screening only).
- ECG variables: heart rate, PR, QRS, QT, and QTcF intervals after 10 minutes of rest in the supine position. Data are locally collected and read at screening (V1) and Week 24 (V6).

Data below the lower limit of quantitation/detection limit (LLOQ) will be replaced by half of the LLOQ, data above the upper limit of quantification will be replaced by ULOQ value.

Quantitative analyses

For all laboratory variables, vital signs and ECG variables above, descriptive statistics for results and changes from baseline will be provided for each analysis window, the last value and the worst value during the on-treatment period. These analyses will be performed using central measurements only (when available) for laboratory variables.

For all parameters, mean changes from baseline with the corresponding standard error will be plotted over time.

Analyses according to PCSA

Analysis of potentially clinically significant abnormality (PCSA) will be performed based on the PCSA list in effect at Sanofi at the time of the database lock. For parameters for which no PCSA criteria are defined, similar analyses will be done using the normal range, if applicable.

Analyses according to PCSA will be performed based on the worst value during the treatment-emergent period, using all measurements (either local or central, either scheduled, nonscheduled or repeated).

For laboratory variables, vital signs and ECG variables above, the incidence of participants with at least one PCSA during the treatment-emergent period will be summarized regardless of the baseline level and according to the following baseline status categories:

- Normal/missing
- Abnormal according to PCSA criterion or criteria;

Additional analyses for suspect drug-induced liver injury

The following additional analyses will be performed for drug-induced liver injury:

- Time to onset of the initial ALT or aspartate aminotransferase (AST) elevation (>3 x ULN) and total bilirubin elevation (>2 x ULN) during the treatment-emergent period will be analyzed using Kaplan-Meier method.
- A graph of the distribution of peak values of ALT versus peak values of total bilirubin during the treatment-emergent period will be provided.
- For each liver function test (eg, ALT), participants having a PCSA (eg, ALT >5 ULN) will be summarized using the following categories: Returned to baseline PCSA status (or returned to value ≤ ULN in case of missing baseline) before last IMP dose, Returned to baseline PCSA status after last IMP dose, Never returned to baseline PCSA status, No assessment after elevation. This summary will be performed by categories of elevation (ALT >3, >5, >10, >20 ULN).

4.8 OTHER ANALYSES

4.8.1 Pharmacokinetic analyses

Predose serum dupilumab concentrations at Visit 2 (Day 1), dupilumab trough levels at Week 4, Week 8, Week 12, Week 24/EOT Visit and posttreatment serum dupilumab at Week 36/EOS Visit will be provided.

Serum concentrations of SAR231893 (REGN668) will be summarized in the PK population using arithmetic and geometric means, SD, standard error of the mean (SEM), coefficient of variation (CV), minimum, median and maximum per sampling time. If date and/or time of the drug injection and/or sampling is missing then the concentration will not be taken into account. For drug-treated participants, where concentration values are below the lower limit of quantification (LLOQ), one-half of the LLOQ will be used. Values will be expressed in the tables with no more than three significant figures. For participants in the placebo group, concentration values that are below the LLOQ will be taken into account with a plasma concentration considered equal to 0.

4.8.2 Immunogenicity analyses

Anti-drug antibody (ADA) status to dupilumab (negative or titer value, if positive in the ADA assay) at Visit 2 (Day 1), Week 12, Week 24/EOT and follow up at Week 36 will be provided. The neutralizing antibody status for ADA positive samples will be provided.

Incidence will be provided for the following ADA response categories:

Pre-existing immunoreactivity is defined as:

An ADA positive response in the assay at baseline with all post first dose ADA results negative, OR an ADA positive response at baseline with all post first dose ADA responses less than 4-fold over baseline titer levels.

Treatment-emergent ADA response is defined as:

A positive response in the ADA assay post first dose, when baseline results are negative or missing.

Treatment-emergent ADA responses are further classified as Persistent, Indeterminate or Transient

a) Persistent Response- defined as a treatment-emergent ADA response with two or more consecutive ADA positive sampling time points, separated by greater than (>) 12-week period (84 days), with no ADA negative samples in between.

b) Indeterminate Response- defined as a treatment-emergent response with only the last collected sample positive in the ADA assay

c) Transient Response - defined as a treatment-emergent response that is not considered persistent OR indeterminate

Treatment-boosted response is defined as:

An ADA positive response in the assay post first dose that is greater-than or equal to 4-fold over baseline titer levels, when baseline results are positive.

Titer values (Titer value category)

- Low (Titer <1000)
- Moderate (1,000 ≤ Titer ≤10,000)
- High (Titer >10,000)

The following summary will be provided based on ADA population:

- Number (%) of participants with pre-existing immunoreactivity
- Number (%) of participants with treatment-emergent ADA
- The summary statistics (including number, median, Q1, Q3, minimum and maximum) of the peak post-baseline titer for participants with treatment-emergent ADA, and participants with persistent, indeterminate and transient ADA response
- Number (%) of participant with transient treatment-emergent ADA
- Number (%) of participants with persistent treatment-emergent ADA

- Number (%) of participants with indeterminate treatment-emergent ADA
- Number (%) of participants with treatment-boosted ADA
- The summary statistics (including number, median, Q1, Q3, minimum and maximum) of the peak post-baseline titer for participants with treatment-boosted ADA
- The summary statistics (including number, mean, SD, median, Q1, Q3, minimum and maximum) of the ratio of peak post-baseline titer to baseline titer for participants with treatment-boosted ADA
- Listing of ADA peak titer levels and neutralizing antibody status
- Number (%) of participants with neutralizing antibody status

Kinetics of treatment-emergent ADA response

Number (%) of participants with treatment-emergent ADA positive response at each visit will be summarized, including titer categories (lower, moderate, and high titer), by each intervention group.

A plot of percentage of participants with treatment-emergent ADA positive response at each visit will be provided by each intervention group.

Association of Immunogenicity with Exposure, Safety and Efficacy

The safety and efficacy analyses mentioned below will be conducted using the following categories:

· ADA positive patients: Patients with treatment-emergent or treatment-boosted response.

· ADA negative patients: Patients with pre-existing immunoreactivity or negative in the ADA assay at all time points.

Association of ADA with PK

Associations between ADA variables (eg, ADA peak titers, neutralizing antibody status, treatment-emergent, persistent, indeterminate and transient response, treatment-boosted) and serum concentration of dupilumab may be explored for each dupilumab dose group. A plot of serum concentration of functional dupilumab versus visit will be provided by ADA classifications for the dupilumab dose group. Individual participant plots of dupilumab concentration according to ADA status will be provided to assess the impact of ADA on PK.

Association of ADA with clinical efficacy endpoints

Associations between the ADA variables (eg, ADA peak titers, neutralizing antibody status, treatment-emergent, persistent and treatment-boosted) and the primary efficacy endpoint may be explored for the dupilumab dosed group.

Association of ADA with clinical safety endpoints

Association of safety versus ADA status may be analyzed in the ADA population. The safety assessment may focus on the following events:

- Severe injection site reactions last longer than 24 hours or serious injection site reactions
- Hypersensitivity reactions (SMQ (20000214) hypersensitivity narrow search confirmed by medical review)
- Anaphylactic reactions (SMQ (20000021) anaphylactic reaction narrow search)

Associations between ADA variables (eg, ADA peak titers, neutralizing antibody status, treatment-emergent, persistent and treatment-boosted) and safety may be explored.

4.8.3 Pharmacodynamic/genomics endpoints

Venous blood samples will be collected at Visit 2 (Week 0), Visit 3 (Week 4), Visit 4 (Week 8), Visit 5 (Week 12), Visit 6 (Week 24), and Visit 7 (Week 36), for measurement of total serum IgE. Total IgE will be measured using validated quantitative methods.

For those participants who consent to the optional pharmacogenetic/pharmacogenomic sample collection section of the ICF, serum/plasma for archival samples for possible future analysis of potential biomarkers of drug response, disease activity, safety, and the type 2 inflammation pathway, and blood samples for exploratory genetic analysis of DNA or RNA will be collected and stored for possible future use. Participation is optional. Participants who do not wish to participate in the genetic research may still participate in the study.

For the optional skin biopsy (substudy), the sample will be taken from lesion and non-lesion skin using punch biopsy.

Total IgE will be summarized in the safety population defined as participants who actually received at least 1 dose or part of a dose of the IMP. Baseline values will be the last value collected prior to the first IMP. Descriptive statistics (including number, mean, SD, median, Q1, Q3, min, max) of biomarkers at baseline will be summarized. In addition, for patients who take selected prohibited medications/procedures or rescue medications defined in Table 4, the data after the medications will be censored and then last observation carried forward (LOCF) method will be implemented for missing data imputation in the analysis.

Summary plots (mean +/- standard error of the mean) on values at each visit, absolute changes from baseline and percent changes from baseline will be provided for the total IgE by intervention group and visit.

Exploratory analysis of DNA/RNA will be addressed in a separate document.

The analyses of the skin biopsy substudy will be addressed in a separate document.

4.9 INTERIM ANALYSES

No interim analysis is planned.

A primary database lock will be performed when all randomized participants in this study have completed their 24-week treatment phase. Final analyses in the CSR will be based on this database.

The database will be updated at the end of the study for all participants to include the post-treatment follow-up information and updates for the events previously ongoing at the time of the primary lock. Additional data between this database lock and last participant completing last visit will be summarized in a CSR addendum.

5 SUPPORTING DOCUMENTATION

5.1 APPENDIX 1 LIST OF ABBREVIATIONS

ADA:	anti-drug antibody
AE:	adverse event
AESIs:	adverse events of special interest
ALT:	alanine aminotransferase
ANCOVA:	analysis of covariance
AST:	aspartate aminotransferase
ATC:	anatomic category
CDG:	company drug grouping
CI:	confidence interval
CLcr:	Creatinine clearance
CMH:	Cochran-Mantel Haenszel
CMQ:	company MedDRA query
CSR:	clinical study report
DLQI:	Dermatology Life Quality Index
DNA:	deoxyribonucleic acid
ECG:	electrocardiogram
eCRF:	electronic case report form
EMA:	European Medicines Agency
EOT:	end of treatment
EQ-5D:	Euroqol 5 dimensions
EQ-5D-5L:	5-level EuroQol 5-dimensional questionnaire
FDA:	Food and Drug Administration
HADS:	hospital anxiety and depression scale
HBc Ab:	hepatitis B core antibody
HBs Ab:	hepatitis B surface antibody
HBs Ag:	hepatitis B surface antigen
HBV:	hepatitis B virus
HCV Ab:	hepatitis C virus antibodies
HGLT:	high level group term
HLT:	high level term
HRQoL:	health-related quality-of-life
IGA PN:	Investigator's global assessment for prurigo nodularis
IGA PN-A:	IGA PN-Activity
IGA PN-S:	IGA PN-Stage
IMP:	investigational medicinal product
ITT:	intent-to-treat
LLT:	lower-level term
LS:	least squares
MedDRA:	medical dictionary for regulatory activities
NRS:	numeric rating scale

PCSA:	potentially clinically significant abnormality
PD:	pharmacodynamic
PGIC:	Participant Global Impression of Change
PGIS:	Participant Global Impression of Severity
PK:	pharmacokinetic
PN:	prurigo nodularis
PT:	preferred term
RNA:	ribonucleic acid
SAE:	serious adverse event
SAP:	statistical analysis plan
SD:	standard deviation
SDG:	standardized drug grouping
SMQ:	standardized MedDRA query
SOC:	system organ class
TEAE:	treatment-emergent adverse event
ULN:	upper limit of normal
VAS:	visual analog scale, visual analog scale
WHO-DD:	World Health Organization-Drug Dictionary
WI-NRS:	worst-itch numeric rating scale
WOCF:	worst-observation carried forward

5.2 APPENDIX 2 CHANGES TO PROTOCOL-PLANNED ANALYSES

This SAP for study EFC16459 is based on the Protocol Amendment 1 dated 20-May-2020. There are no major changes to the statistical analysis features in this SAP, this section summarizes major statistical changes in the protocol amendment.

Table 8 – Major statistical changes in protocol amendment(s)

Amendment Number	Approval Date	Changes	Rationale
1	20-May-2020	To add "proportion of participants with Investigator's Global Assessment 0 or 1 score for PN-Stage (IGA PN-S) at Week 24" as another key secondary endpoint	To include a lesion-related key secondary endpoint according to the health authority's recommendations
		To remove the endpoint "Change from baseline in PAS total score at Week 4, Week 8, Week 12, and Week 24" in the exploratory endpoint and modify the exploratory endpoint regarding the healed lesions from PAS questionnaire analysis	To clarify the analysis on efficacy evaluation of dupilumab on skin lesions using a modified prurigo activity score (PAS) 5-item questionnaire
		To break out the secondary endpoints with multiple measuring timepoints into individual endpoints.	To clearly define the timepoints of each endpoint according to health authority's recommendation
		To add the sensitivity analysis for secondary endpoints information, and to separate key secondary endpoints in a different row	To evaluate the robustness of the missing data imputation assumption by sensitivity analyses, and to clarify the endpoints analyses
		To add the covariate "baseline anti-depressant use (yes or no)" to primary and secondary endpoint analyses	To adjust for potential impact of anti-depressant use on the treatment effect in primary and secondary efficacy analyses
		The following wording was added: "Data collected regarding the impact of the COVID-19 or other pandemics, on the participants will be summarized (eg, discontinuation due to COVID-19). Any additional analyses and methods required to investigate the impact of COVID-19 or other pandemics requiring public health emergency on the efficacy (eg, missing data due to COVID-19) and safety will be detailed in the SAP".	To describe alternative temporary mechanism that can be implemented in the study conduct in case of pandemic requiring public health emergency eg, COVID-19

5.3 APPENDIX 3 DEMOGRAPHICS AND BASELINE CHARACTERISTICS, PRIOR OR CONCOMITANT MEDICATIONS/PROCEDURES

Demographics, baseline characteristics, medical surgical history

The following demographics and baseline characteristics, medical and surgical history and disease characteristics at baseline will be summarized using descriptive statistics in the randomized population.

Demographic variables are

- Age in years (quantitative and qualitative variable: 18-<40, 40–<65, 65-<75, and ≥75 years),
- Gender (Male, Female),
- Race (White, Black or African American, Asian, American Indian or Alaska Native, Native Hawaiian or other Pacific Island, unknown),
- Ethnicity (Hispanic or Latino, Not Hispanic or Latino, Unknown),
- Region (Asia: Japan, China, South Korea; East Europe: Hungary, Russia; Latin America: Argentina, Mexico; Western Countries: USA, Canada, France, Portugal, Spain),
- Territory (North America: USA, Canada; European Union: France, Hungary, Portugal, Spain; Rest of World: Russia, Japan, China, South Korea, Argentina, Mexico),
- Weight in kg (quantitative and qualitative variable: (<60, ≥60- <90, ≥90 kg),
- BMI in kg/m^2 (quantitative and qualitative variable: (<25, ≥25- <30, ≥30 kg/m^2).

Baseline safety and efficacy parameters (apart from those listed above) will be presented along with the safety and efficacy summaries.

Medical (or surgical) history includes all the relevant medical (or surgical) history during the lifetime of the participant.

This information will be coded using the version of MedDRA currently in effect at Sanofi at the time of database lock.

Comorbidity will be summarized separately. The following comorbid diseases will be summarized from electronic case report form (eCRF) pages which were filled in by Investigators based on participant reporting.

- Prurigo nodularis (Yes, Ongoing condition)
- Atopic dermatitis (Yes, Ongoing condition)
- Allergic rhinitis history (Yes, Ongoing condition)
- Allergic rhinoconjunctitvitis (Yes, Ongoing condition)
- Asthma history (Yes, Ongoing condition)

- Food allergy history (Yes, Ongoing condition)
- Eosinophilic esophagitis history (Yes, Ongoing condition).

Disease characteristics at baseline

The following baseline disease characteristics will be summarized by intervention group:

- Duration of PN and grouping (years; <3 and ≥3) to be derived as
  - (Year of randomization – Year of first diagnosis of PN) + (month of randomization – month of first diagnosis of PN)/12
- Age of onset of PN (years)
- History of atopy (atopic or non-atopic)
  - Number of mild AD participants under atopic
- Stable use of TCS/TCI (yes or no)
- Baseline WI-NRS score
- Baseline IGA PN-S score (summary and frequency by each score level)
- Baseline IGA PN-A score (summary and frequency by each score level)
- Baseline Pain-NRS score
- Baseline Sleep-NRS score
- Baseline PGIS score
- PAS (number of prurigo lesions in a total and in representative area, and healed prurigo lesions) at baseline
- Baseline Hospital Anxiety and Depression Scale (HADS) total score
  - Baseline Hospital Anxiety score
  - Baseline Depression Scale score
- Baseline Dermatology Life Quality Index (DLQI) score
- Baseline EuroQol five dimensions questionnaire (ED-5D-5L) (single index score and VAS)
- Frequency of alcohol drinking in the past 12 months (never, occasional, at least monthly, at least weekly, at least daily) and number of drinks on a typical day (1 or 2, >2)
- Antidepressant use (yes or no) at baseline
- HIV (positive versus negative)

Prior or concomitant medications and procedure

All medications will be coded using the World Health Organization-Drug Dictionary (WHO-DD) using the version currently in effect at Sanofi at the time of database lock.

All procedures will be coded to a PT and associated primary SOC using the version of MedDRA currently in effect at Sanofi at the time of database lock.

- Prior medications/procedures are those the participant used prior to first investigational medicinal product (IMP) injection. Prior medications/procedures can be discontinued before first administration or can be ongoing during treatment phase.
- Concomitant medications/procedures are any interventions received by the participant concomitantly to the IMP, from first administration of IMP to last IMP intake + 98 days.
- Post-treatment medications/procedures are those the participant took in the period running from the end of the concomitant medications period up to the end of the study.
- A given medication/procedure can be classified as a prior medication/procedure and/or as a concomitant medication/procedure and/or as post-treatment medication/procedure. If it cannot be determined whether a given medication/procedure was taken prior or concomitantly or post, it will be considered as prior, concomitant, and post-treatment medication/procedure.

The prior and concomitant medications/procedure will be summarized for the randomized population.

Medications will be summarized by intervention group according to the WHO-DD dictionary, considering the first digit of the anatomic category (ATC) class (anatomic category) and the first 3 digits of the ATC class (therapeutic category). All ATC codes corresponding to a medication will be summarized, and participants will be counted once in each ATC category (anatomic or therapeutic) linked to the medication. Therefore, participants may be counted several time for the same medication.

The table for prior medications will be sorted by decreasing frequency of ATC followed by all other therapeutic classes based on the overall incidence across intervention groups. In case of equal frequency regarding ATCs (anatomic or therapeutic categories), alphabetical order will be used.

Concomitant medication received during first IMP to last IMP +14 days and concomitant medication received during first IMP to last IMP +98 days will be summarized separately. The tables for concomitant medications will be sorted by decreasing frequency of ATC followed by all other therapeutic classes based on the incidence in the dupilumab group. In case of equal frequency regarding ATCs (anatomic or therapeutic categories), alphabetical order will be used.

Medications will also be summarized by generic name sorted by decreasing frequency based on the incidence in the dupilumab group.

Procedures will be summarized by intervention group by primary SOC (sorted by internationally

agreed order) and PT (sorted in alphabetical order), sorting is based on the overall incidence across intervention groups.

Background intervention

Participants will be required to apply moisturizers (emollients) once or twice daily for at least the 7 consecutive days immediately before baseline (Day 1) and continue until Week 36.

The compliance of moisturizers (emollients) used from 7 days before the baseline visit to Week 36 (or end of study), which is defined as the (number of days moisturizers used during the period) / (number of days within the period) x 100%, will be summarized by intervention group.

Similarly, the compliance of moisturizers (emollients) used from the baseline visit to Week 12 and Week 24 will be summarized by intervention group respectively.

In addition, the participants who take TCS/TCI during the study will be summarized by visit.

Prohibited medications/procedures

The concomitant use of the following therapies is prohibited during the entire study. Study treatment will need to be discontinued in participants receiving these treatments:

- Systemic immunosuppressive/immunomodulating drugs (eg, systemic corticosteroids, cyclosporine, mycophenolate-mofetil, interferon gamma, Janus kinase inhibitors, azathioprine, methotrexate, hydroxychloroquine, dapsone, sulfasalazine, colchine, etc)
- Other monoclonal antibodies (which are biological modifiers)
- Phototherapy, including tanning beds
- Naltrexone or other opioid antagonist
- Gabapentin, pregabalin, and thalidomide

The concomitant use of the following therapies is prohibited except if the dose has been stable for at least 3 months prior to screening, but study treatment will not need to be discontinued in participants receiving the treatments listed below. The dose should also remain stable (can be reduced or discontinued if medically indicated) and should not be increased during the study.

- Paroxetine, fluvoxamine, or other SSRIs
- SNRIs
- Amitriptyline or other tricyclic or tetracyclic antidepressants

The concomitant use of the following therapies is also prohibited during the entire study, but study treatment will not need to be discontinued in participants receiving the treatments listed below:

- Intralesional corticosteroid injections and cryotherapy
- Sedating antihistamine

- Non-sedating antihistamine if used specifically for the treatment of itch secondary to AD or PN.

The number and percentage of participants who take the prohibited medications/procedures will be provided. In addition, the time (week) of first prohibited medication/procedure taken will also be analyzed using Kaplan-Meier method.

Rescue medications

The following rescue medications may be used:

- Dermatological preparations of high potency or superpotent TCS and TCI.

If medically necessary (ie, to control intolerable PN symptoms), rescue treatment for PN may be provided to study participants at the discretion of the Investigator.

Although the use of rescue medications is allowed at any time during the study, the use of rescue medications should be delayed, if possible, for at least 14 days following the initiation of the investigational treatment. The date and time of rescue medication administration as well as the name and dosage regimen of the rescue medication must be recorded in the eCRF.

- For the purpose of the efficacy responder analysis, a pre-specified algorithm will be used to classify rescue. In addition, a blinded review of all post-baseline medications to adjudicate rescue treatment, based on medical judgment, will be performed to adjudicate rescue. Participants who receive rescue treatment as per this adjudication during the study will be considered treatment failures.

The rescue medications will be analyzed by the same method as that used for prohibited medications/procedures.

5.4 APPENDIX 4 DATA HANDLING CONVENTIONS

Demographic formulas

Age of onset of PN is calculated as:

Year of PN diagnosis – Year of birth

BMI is calculated as:

$$\text{Weight in kg} / (\text{height}^2 \text{ in meters})$$

Renal function formulas

For adults, creatinine clearance (CLcr) value will be derived using the equation of Cockroft and Gault:

$$\text{CLcr (ml/min)} = (140 - \text{age}) \times \text{weight (kg)} \times (1 - 0.15 \times \text{sex (0-M, 1-F)}) / (0.814 \times \text{creatinine } (\mu\text{mol/l}))$$

CLcr will be calculated using the last weight measurement on or before the visit of the creatinine measurement and age at the lab sampling day. Here age is calculated as following:

Age = age collected at screening + integer part of (lab sampling analysis day/365.25)

Analysis windows for time points

Efficacy assessment

For the efficacy assessment, the reference date for the derivation of relative days of events or findings will be the randomization day. If a participant receives IMP prior to the randomization by mistake, the reference date of efficacy assessment will be the date of the first IMP administration for that participant.

For daily eDiary data (WI-NRS, Pain-NRS, Sleep-NRS), all available values of daily measurements will be assigned to each week window according to Table 9, and then weekly average score will be calculated. Randomization day is used as the reference day (Day 1).

Table 9 – Time window for eDiary efficacy variables

Time	Target day	Day range for calculating weekly score
Baseline (Week 0)	1	-7- <1
Week 1	8	1-11
Week 2	15	12-18
Week 3	22	19-25
Week 4	29	26-32
Week 5	36	33-39
Week 6	43	40-46
Week 7	50	47-53
Week 8	57	54-60
Week 9	64	61-67
Week 10	71	68-74
Week 11	78	75-81
Week 12	85	82-88
Week 13	92	89-95
Week 14	99	96-102
Week 15	106	103-109
Week 16	113	110-116
Week 17	120	117-123
Week 18	127	124-130
Week 19	134	131-137

Time	Target day	Day range for calculating weekly score
Week 20	141	138-144
Week 21	148	145-151
Week 22	155	152-158
Week 23	162	159-165
Week 24	169	166-172
Week 25	176	173-179
Week 26	183	180-186
Week 27	190	187-193
Week 28	197	194-200
Week 29	204	201-207
Week 30	211	208-214
Week 31	218	215-221
Week 32	225	222-228
Week 33	232	229-235
Week 34	239	236-242
Week 35	246	243-249
Week 36	253	250-256

For other efficacy variables, all available values of scheduled measurements will be assigned to the appropriate visit window according to Table 10. In the event of multiple measurements of the same test in the same window, the one closest to the targeted visit date will be used for the by-visit summary. If they are at the same number of days away from the target day, the latest one will be used.

Table 10 – Time window for efficacy variables

Visit	Target Day	Time windows for DLQI, IGA PN-A/PN-S	HADS, EQ-5D-5L, Missed school/work days	PGIS	PGIC	PAS
Visit 1 (Week -4 to -2)	<1			<-14		<-14
Visit 2 (Week 0)	1	1^-	1^-	-14-1^-		-14-1^-
Visit 3 (Week 4)	29	1^+-42		1^+-42	1^+-42	1^+-42
Visit 4 (Week 8)	57	43-70		43-70	43-70	43-70
Visit 5 (Week 12)	85	71-126	1^+-126	71-126	71-126	71-126
Visit 6 (Week 24)	169	127-210	127-210	>126	>126	127-210
Visit 7 (Week 36)	253	>210	>210			>210

1^-: up to randomization and before 1st dose date/time; 1^+: after randomization or 1st dose date/time

Safety assessment

For the safety assessment, the reference date for the derivation of relative days of events or findings will be the date of first IMP administration. Selected safety variables will be summarized by the analysis window define in Table 11 for the by visit descriptive analysis. All available values from central lab will be assigned to the appropriate visit window. In the event of multiple measurements of the same test in the same window, the one closest to the targeted visit date will be used for the by-visit summary. If they are at the same number of days away from the target day, the latest one will be used. For procedures planned on Visit 2, if it is done on the same date as the first IMP injection but the performance time is missing, it will belong to Visit 2 time window.

Table 11 – Time window for safety endpoints

		Time windows for							
Visit	Target Day	Vital signs	Hematology, biochemistry	Urinalysis	CD4 T cell count and HIV test	Serum Pregnancy test	Urine Pregnancy test	Physical examination	ECG
Visit 1 (Week -4 to -2)	<1	<-14	<-14	<-14	1^-	<-14		<-14	1^-
Visit 2 (Week 0)	1	-14-1^-	-14-1^-	-14-1^-			-14-1^-	-14-1^-	
Visit 3 (Week 4)	29	1^+-42	1^+-42	1^+-42			1^+-42		
Visit 4 (Week 8)	57	43-70	43-70	43-70			43-70		
Visit 5 (Week 12)	85	71-126	71-126	71-126			71-126		
Visit 6 (Week 24)	169	127-210	127-210	>126	1^+		127-210	1^+-210	1^+
Visit 7(Week 36)	253	>210	>210				>210	>210	

1^-: up to 1st dose date/time; 1^+: after 1st dose date/time;

Pharmacokinetics/pharmacodynamics assessment

For the pharmacokinetics/pharmacodynamics variables summary, the reference date for the derivation of relative days of measurements will be the date of first IMP administration if the participant is treated with study treatment, or the randomization date if the participant is not treated. Pharmacokinetics /pharmacodynamics variables will be summarized by the analysis window defined in Table 12 for the by visit descriptive analyses. All available values of measurements will be assigned to the appropriate visit window. In the event of multiple measurements of the same test in the same window, the one closest to the targeted visit date will be used for the by-visit summary. If they are at the same distance to the target day, the latest one will be used. For procedures planned on Visit 2, if it is done on the same date as the first IMP injection but the performance time is missing, it will belong to Visit 2 time window.

Table 12 – Time window for pharmacokinetics/pharmacodynamics variables

		Time windows for		
**Visit**	**Target day**	**Serum dupilumab concentration**	**Anti-drug antibodies**	**Total IgE**
Visit 1 (Week -4)	<1			
Visit 2 (Week 0)	1	1^-	1^-	1^-
Visit 3 (Week 4)	29	1^+-42		1^+-42
Visit 4 (Week 8)	57	43-70		43-70
Visit 5 (Week 12)	85	71-126	1^+-126	71-126
Visit 6 (Week 24)	169	127-210	127-210	127-210
Visit 7(Week 36)	253	>210	>210	>210

1^-: up to 1st dose date/time or randomization if participant is not treated; 1^+: after 1st dose date/time or randomization date if participant is not treated;

Unscheduled visits

Unscheduled visit measurements of laboratory data, vital signs, and ECG will be used for computation of baseline, the last on-treatment value, analysis according to PCSAs, and the shift summaries for safety. They will also be included in the by-visit summaries if they are re-allocated to scheduled visits.

5.5 APPENDIX 5 SAMPLE SAS CODE

The multiple imputation and analysis model for the primary analysis of change from baseline in WI-NRS at Week 12 will be built with the following sample SAS code.

1. 40 datasets with a monotone missing pattern will be obtained, induced by Markov Chain Monte Carlo (MCMC) method on participants who have not taken selected prohibited

medications and/or rescue medications or have discontinued study treatment due to lack of efficacy prior to Week 12.

```
proc mi data=dat_etd seed=16461 nimpute=40 out=dat_mc;
    mcmc impute=monotone;
    var atopicyn stableyn region antidblyn trt01p winrsbl chg1winrs …
    chg24winrs;
run;
```

2. For each of the imputed dataset with monotone missing pattern in step 1, the remaining missing data will be imputed using the regression method for the monotone pattern with adjustment for covariates including intervention groups, documented history of atopy (atopic or non-atopic), stable use of TCS/TCI (yes or no), region, baseline anti-depressant use (yes or no), and baseline value of the response variable.

```
proc mi data=dat_mc nimpute=1 seed=16461 out=dat_mi;
    by _imputation_;
    class atopicyn stableyn region antidblyn trt01p;
    monotone method=reg;
    var atopicyn stableyn region antidblyn trt01p winrsbl chg1winrs …
    chg24winrs;
run;
```

3. Each of the 40 imputed datasets will be merged with the one dataset imputed by WOCF approach, and then be analyzed using the main statistical model. These 40 imputed datasets will be saved.

```
%macro w1;
      %do i=1%to 40;
      data wocf&i.;
      set wocf;
      _imputation_=&i.;
      run;
      %end;
      data wocf_all;
      set %do j=1 %to 40; wocf&j. %end;;
      run;
%mend w1;

%w1

data dat_imp;
      set dat_mi  wocf_all;
Run;

proc sort data=dat_imp;
      by _imputation_;
run;
```

```
proc glm data= dat_imp;
     by _imputation_;
     class atopicyn stableyn region antidblyn trt01p;
     model  chg24winrs = atopicyn stableyn region antidblyn
              trt01p winrsbl;
     lsmeans trt01p / stderr;
     estimate 'Diff Dupilumab vs Placebo' trt01p -1 1;
     ods output LSMeans=implsmeans Estimates=implsmeandiff;
run;
```

4. Applying Rubin's rule to combine analysis results (point estimates and standard errors) from 40 imputations using PROC MIANALYZE for the LS means and difference in LS means between dupilumab and placebo. Sample code:

```
proc sort data=implsMeans; by trt01pn _imputation_;run;

proc mianalyze data= implsmeans;
     by trt01pn;
     modeleffects lsmean;
     stderr stderr;
     ods output ParameterEstimates=lsmeans;
run;

proc mianalyze data=implsmeandiff;
     modeleffects estimate;
     stderr stderr;
     ods output ParameterEstimates=lsmeandiff;

run;
```

5.6 APPENDIX 6 SELECTION CRITERIA FOR AE/MEDICATION GROUPINGS

Table 13 – List of PTs or Medications for CMQs/CDGs

Grouping	Preferred Term/Medication Code	Preferred Term/Medication
Conjunctivitis	10001257	Adenoviral conjunctivitis
Conjunctivitis	10010725	Conjunctival irritation
Conjunctivitis	10010726	Conjunctival oedema
Conjunctivitis	10010736	Conjunctival ulcer
Conjunctivitis	10010741	Conjunctivitis
Conjunctivitis	10010744	Conjunctivitis allergic
Conjunctivitis	10010745	Conjunctivitis chlamydial
Conjunctivitis	10010749	Conjunctivitis gonococcal neonatal
Conjunctivitis	10010754	Conjunctivitis tuberculous
Conjunctivitis	10010755	Conjunctivitis viral
Conjunctivitis	10018258	Giant papillary conjunctivitis
Conjunctivitis	10021629	Inclusion conjunctivitis
Conjunctivitis	10030861	Ophthalmia neonatorum
Conjunctivitis	10048908	Seasonal allergy
Conjunctivitis	10049458	Herpes simplex virus conjunctivitis neonatal
Conjunctivitis	10051625	Conjunctival hyperaemia
Conjunctivitis	10053991	Inclusion conjunctivitis neonatal
Conjunctivitis	10061784	Conjunctivitis bacterial
Conjunctivitis	10062889	Pingueculitis
Conjunctivitis	10063669	Photoelectric conjunctivitis
Conjunctivitis	10067317	Oculorespiratory syndrome
Conjunctivitis	10067817	Acute haemorrhagic conjunctivitis
Conjunctivitis	10069166	Blebitis
Conjunctivitis	10071570	Ligneous conjunctivitis
Conjunctivitis	10074701	Noninfective conjunctivitis
Conjunctivitis	10075264	Oculoglandular syndrome
Conjunctivitis	10080825	Conjunctivitis fungal
Conjunctivitis	10084034	Conjunctival suffusion
Intravenous immunoglobulin therapy	CAS 8000012671	IMMUNOGLOBULIN HUMAN NORMAL
Intravenous immunoglobulin therapy	CAS 8000050682	IMMUNOGLOBULIN, PORCINE
Intravenous immunoglobulin therapy	CAS 8000056919	IMMUNOGLOBULIN G HUMAN

Grouping	Preferred Term/Medication Code	Preferred Term/Medication
Intravenous immunoglobulin therapy	CAS 8600000563	IMMUNOGLOBULINS NOS
Intravenous immunoglobulin therapy	CAS 8600001670	IMMUNOGLOBULIN HUMAN NORMAL SLRA
Intravenous immunoglobulin therapy	CAS 8600001671	IMMUNOGLOBULIN HUMAN NORMAL IFAS
Intravenous immunoglobulin therapy	RECNO 900708	OTHER IMMUNOGLOBULINS
Intravenous immunoglobulin therapy	RECNO 900722	IMMUNE SERA AND IMMUNOGLOBULINS
Intravenous immunoglobulin therapy	RECNO 900728	IMMUNOGLOBULINS
Intravenous immunoglobulin therapy	RECNO 900914	SPECIFIC IMMUNOGLOBULINS
Intravenous immunoglobulin therapy	RECNO 901112	IMMUNOGLOBULINS, NORMAL HUMAN

Abbreviations: CAS : Chemical Abstract Service Registry Number RECNO : Drug Record Number

5.7 APPENDIX 7 MEDICATION/PROCEDURE AJUDICATION ALGORITHM

Algorithm for determining whether treatment with following medications or procedures constitutes treatment failure resulting in setting data as non-responder after taking medication or undergoing procedure in the main statistical analysis

1. Not required to adjudicate post-baseline medications (WHODD-coded) or procedures (CMQ-coded) as these will be considered treatment failures if used at any time[1].

a. Always considered treatment failure

- ATC2 = CORTICOSTEROIDS FOR SYSTEMIC USE
- ATC2=IMMUNOSUPPRESSANTS (systemic, e.g. oral or parenteral route)
- Preferred Drug Name = Ciclosporin
- Preferred Drug Name = Methotrexate
- Preferred Drug Name = Mycophenolate sodium
- Preferred Drug Name = Mycophenolic acid
- Preferred Drug Name = Azathioprine
- Preferred Drug Name = Gabapentin
- Preferred Drug Name = Pregabalin
- Preferred Drug Name = Thalidomide
- Preferred Drug Name = Lenalidomide
- Preferred Drug Name = Baricitinib
- Preferred Drug Name = Ruxolitinib
- Preferred Drug Name = Tofacitinib
- Preferred Drug Name = AbrocitinibPreferred Drug Name = Delgocitinib
- Preferred Drug Name = Nalbuphine
- Preferred Drug Name = Naltrexone
- Preferred Drug Name = Naloxone
- Preferred Drug Name = Vixarelimab
- Preferred Drug Name = Nemolizumab
- Preferred Drug Name = Serlopitant
- Preferred Drug Name = Aprepitant
- CMQb HLT Phototherapies
- CMQ Tanning_single PT

b. Never considered treatment failure:

- ATC2 = EMOLLIENTS AND PROTECTIVES
- ATC2 = GENERAL NUTRIENTS
- ATC2 = VITAMINS
- ATC2 = ANTIVIRALS FOR SYSTEMIC USE
- ATC2 = ANTIFUNGALS FOR DERMATOLOGICAL USE
- ATC2 = ANTISEPTICS AND DISINFECTANTS

- ATC2 = ANTIBIOTICS AND CHEMOTHERAPEUTICS FOR DERMATOLOGICAL USE
- ATC2 = ANTI-ACNE PREPARATIONS, excluding D10AA Corticosteroids, combinations for treatment of acne
- ATC2 = OPHTHALMOLOGICALS
- ATC1 = ANTIINFECTIVES FOR SYSTEMIC USE, excluding D07C Corticosteroids, combinations with antibiotics
- ATC1 = BLOOD AND BLOOD FORMING ORGANS
- ATC1 = ALIMENTARY TRACT AND METABOLISM
- ATC1 = MUSCULO-SKELETAL SYSTEM, excluding M01BA Antiinflammatory/antirheumatic agents in combination with corticosteroids
- ATC2 = COUGH AND COLD PREPARATIONS
- ATC2= PSYCHOLEPTICS, excluding N05BB Diphenylmethane derivatives and N05C HYPNOTICS AND SEDATIVES

[1] A blinded review of all post-baseline medications or procedures to adjudicate whether treatment constitutes treatment failure, based on medical judgment, may be performed in addition. A listing of treatments or procedures classified as treatment failure in a manner inconsistent with the classification under #1 will be provided, along with supporting rationale.

2. **Require to adjudicate medications or procedures that may constitute treatment failure**
    - All other medications and procedures listed in the protocol and Table 4 of the SAP (not noted in 1. above) given for indications consistent with PN[2]
    - Considerations in determining treatment failure include the criteria already set forth in Table 4 of the SAP in addition to the type of medication or procedure, indication, dose, route of administration, timing, frequency and the potential impact of the use of the prohibited medications/procedures and rescue medications on the primary and key secondary efficacy endpoints.

[2] Below is a list of indications consistent with PN based on PT level from concomitant medication/procedure data using MedDRA dictionary

System Organ Class	High Level Term	Preferred Term	Preferred Term Code
Skin and subcutaneous tissue disorders	Dermatitis and eczema	Neurodermatitis	10029263
Infections and infestations	Bacterial infections NEC	Eczema impetiginous	10051890
Infections and infestations	Skin structures and soft tissue infections	Dermatitis infected	10012470
Infections and infestations	Skin structures and soft tissue infections	Eczema infected	10014199

System Organ Class	High Level Term	Preferred Term	Preferred Term Code
Skin and subcutaneous tissue disorders	Dermatitis and eczema	Dermatitis	10012431
Skin and subcutaneous tissue disorders	Dermatitis and eczema	Dermatitis atopic	10012438
Skin and subcutaneous tissue disorders	Dermatitis and eczema	Eczema	10014184

6 REFERENCES

1. Carpenter JR, Roger JH, Kenward MG. Analysis of longitudinal trials with protocol deviation: a framework for relevant, accessible assumptions, and inference via multiple imputation. J Biopharm Stat. 2013; 23(6):1352-1371.

Signature Page for VV-CLIN-0602722 v1.0
efc16459-16-1-9-sap

Approve & eSign	[redacted]

Approve & eSign	[redacted]

AMENDED CLINICAL TRIAL PROTOCOL 03

**Protocol title:**	**A randomized, double blind, placebo-controlled, multi-center, parallel group study to evaluate the efficacy and safety of dupilumab in patients with prurigo nodularis who are inadequately controlled on topical prescription therapies or when those therapies are not advisable**
**Protocol number:**	**EFC16459**
**Amendment number:**	**03**
**Compound number (INN/Trademark):**	**SAR231893/REGN668 dupilumab/Dupixent**
**Study phase:**	**Phase 3**
**Short title:**	**Study of dupilumab for the treatment of patients with prurigo nodularis, inadequately controlled on topical prescription therapies or when those therapies are not advisable**<br>**LIBERTY-PN PRIME**
**Sponsor name:**	**Sanofi-Aventis Recherche & Développement**
**Legal registered address:**	**1 avenue Pierre Brossolette Chilly-Mazarin France**
**Monitoring Team's Representative Name and Contact Information**	

Regulatory agency identifier number(s):

IND:	IND107969
EudraCT:	2019-003774-41
NCT:	NCT04183335
WHO:	U1111-1241-8153
Other:	Not applicable

Approval Date: 21-Oct-2021 Total number of pages: 115

Any and all information presented in this document shall be treated as confidential and shall remain the exclusive property of Sanofi (or any of its affiliated companies). The use of such confidential information must be restricted to the recipient for the agreed purpose and must not be disclosed, published or otherwise communicated to any unauthorized persons, for any reason, in any form whatsoever without the prior written consent of Sanofi (or the concerned affiliated company); 'affiliated company' means any corporation, partnership or other entity which at the date of communication or afterwards (i) controls directly or indirectly Sanofi, (ii) is directly or indirectly controlled by Sanofi, with 'control' meaning direct or indirect ownership of more than 50% of the capital stock or the voting rights in such corporation, partnership or other entity

PROTOCOL AMENDMENT SUMMARY OF CHANGES

DOCUMENT HISTORY

Document	Country/countries impacted by amendment	Date, version
Amended Clinical Trial Protocol 03	All	21 Oct 2021, version 1 (electronic 1.0)
Amended Clinical Trial Protocol 02	All	14 Apr 2021, version 1 (electronic 2.0)
Amended Clinical Trial Protocol 01	All	20 May 2020, version 1 (electronic 1.0)
Original Protocol		25 October 2019, version 1 (electronic 3.0)

Amended protocol 03 (21 October 2021)

This Amended Protocol 03 (Amendment 03) is considered to be substantial based on the criteria set forth in Article 10(a) of Directive 2001/20/EC of the European Parliament and the Council of the European Union.

OVERALL RATIONALE FOR THE AMENDMENT

EFC16460, a second Phase 3 study in prurigo nodularis (PN) with a similar design and population, provided evidence of the positive treatment effect of dupilumab in PN participants, based on the recently completed primary analysis. Results of the primary analysis evidenced that the effect of dupilumab over time showed continuous improvement after Week 12 across all endpoints, with a similar time course of improvement in both itch and lesion endpoints through at least Week 24. Based on these data, the Amendment is changing the primary endpoint to be the proportion of participants with improvement (reduction) in worst-itch numeric rating scale (WI-NRS) by ≥4 from baseline to Week 24, which represents the overall treatment effect more accurately and synchronizes the primary itch assessment with the primary lesion assessment.

Protocol amendment summary of changes table

Section # and Name	Description of Change	Brief Rationale
1.1 Synopsis: Objectives and endpoints<br>3 Objectives and Endpoints	To promote the "proportion of participants with improvement (reduction) in worst-itch numeric rating scale (WI-NRS) by ≥4 from baseline to Week 24 as primary endpoint and to move the "proportion of participants with improvement (reduction) in WI-NRS by ≥4 from baseline to Week 12" to a secondary endpoint	Based on the data from EFC16460, the treatment effect of dupilumab continued to improve over time through Week 24. Therefore, the Sponsor proposes to assess the proportion of participants with improvement (reduction) in WI-NRS by ≥4 at Week 24, which represents the effect more accurately and synchronizes the primary itch assessment with the primary lesion assessment.
1.1 Synopsis Statistical considerations – Sample size calculation	To update the sample size calculation based on the observed effect sizes from EFC16460.	To assess a timepoint that reflects the optimal dupilumab treatment effect as a primary endpoint

Section # and Name	Description of Change	Brief Rationale
1.1 Synopsis Statistical considerations – Analysis of primary endpoint	To update the primary analysis considerations to the Week 24 timepoint.	To be consistent with primary endpoint change
1.2 Schema	To update Week 24 timepoint as the primary endpoint.	To be consistent with primary endpoint change
3.1 Appropriateness of measurement	To remove the sentence “A key secondary endpoint will be the responder analyses of itch improvement of at least 4 points at Week 24.” as it is now the primary endpoint, and to provide rationale for choosing the primary endpoint timepoint at Week 24 with the following language: “The timing of the primary assessment, ie, at Week 24, was based on the results of the primary analysis of EFC16460, showing that the effect of dupilumab over time showed continuous improvement after Week 12 across all endpoints, with a similar time course of improvement in both itch and lesion endpoints through at least Week 24. Since improvement of itch and lesions may occur prior to Week 24, responder analyses assessments will be performed at earlier time points as well, starting at Week 2 for itch, and Week 4 for lesions.”	To provide rationale and maintain consistency in language with the change in the primary endpoint.
4.2 Scientific Rationale for Study Design	To specify that the rationale for a 24-week duration of the trial is an appropriate duration based on data observed from EFC16460 and to remove references to atopic dermatitis trials.	To reflect that data from EFC16460 has informed our understanding of the dupilumab treatment effect continuing to improve itch through Week 24. The Week 24 timepoint most accurately represents the overall treatment effect and synchronizes the primary itch assessment with the primary lesion assessment.
9.1 Statistical hypotheses<br>9.2 Sample size determination	To reflect that the primary endpoint timepoint is Week 24.	To maintain consistency in language with the change in the primary endpoint.
9.4.1 Efficacy Analyses	To update Table 6 to reflect that the primary endpoint is assessed at Week 24.	To maintain consistency in language with the change in the primary endpoint.
1.1 Synopsis – Primary analysis, Planned database lock, and Unblinding plan<br>9.3 Populations for analyses<br>9.4.1 Efficacy analyses	To remove the possibility of re-evaluating the timing of the primary database lock of EFC16459 based on the observed treatment effect size in EFC16460, that was added per Amendment 02.<br>And to consequently remove the 2 populations (ITT-Week 12 and ITT-Week 24) that were added per Amendment 02.	Amended protocol 02 was submitted in few countries but was withdrawn following a Health Authority feedback and was not implemented in any country.

Section # and Name	Description of Change	Brief Rationale
9.4.2 Safety analyses<br>9.6 Timing of primary database lock<br>9.8 Unblinding plan		
10.12 Appendix 12 Protocol amendment history	Subsection 10.12.2 was added and the overall rationale and table with summary of changes for Amended protocol 02 were moved from the cover page to this section.	This is aligned with Sanofi procedures.
Global	Minor editorial/formatting changes were done throughout the document.	To correct minor editorial/formatting issues.

TABLE OF CONTENTS

AMENDED CLINICAL TRIAL PROTOCOL 03 1

PROTOCOL AMENDMENT SUMMARY OF CHANGES 2

TABLE OF CONTENTS 5

LIST OF TABLES 9

1 PROTOCOL SUMMARY 10

1.1 SYNOPSIS 10

1.2 SCHEMA 17

1.3 SCHEDULE OF ACTIVITIES (SOA) 18

2 INTRODUCTION 23

2.1 STUDY RATIONALE 23

2.2 BACKGROUND 23

2.3 BENEFIT/RISK ASSESSMENT 25

3 OBJECTIVES AND ENDPOINTS 28

3.1 APPROPRIATENESS OF MEASUREMENTS 29

4 STUDY DESIGN 31

4.1 OVERALL DESIGN 31

4.2 SCIENTIFIC RATIONALE FOR STUDY DESIGN 31

4.3 JUSTIFICATION FOR DOSE 32

4.4 END OF STUDY DEFINITION 32

5 STUDY POPULATION 33

5.1 INCLUSION CRITERIA 33

5.2 EXCLUSION CRITERIA 35

5.3 LIFESTYLE CONSIDERATIONS 39

5.4 SCREEN FAILURES 39

6 STUDY INTERVENTION 40

6.1 STUDY INTERVENTION(S) ADMINISTERED 40

6.1.1 Noninvestigational medicinal products 41

6.2 PREPARATION/HANDLING/STORAGE/ACCOUNTABILITY....42
6.2.1 Storage and handling....42
6.2.2 Responsibilities....42
6.3 MEASURES TO MINIMIZE BIAS: RANDOMIZATION AND BLINDING....42
6.4 STUDY INTERVENTION COMPLIANCE....44
6.5 CONCOMITANT THERAPY....44
6.5.1 Rescue medicine....45
6.6 DOSE MODIFICATION....46
6.7 INTERVENTION AFTER THE END OF THE STUDY....46
7 DISCONTINUATION OF STUDY INTERVENTION AND PARTICIPANT DISCONTINUATION/WITHDRAWAL....47
7.1 DISCONTINUATION OF STUDY INTERVENTION....47
7.1.1 Definitive discontinuation....47
7.1.2 Temporary discontinuation....48
7.1.2.1 Rechallenge....49
7.2 PARTICIPANT DISCONTINUATION/WITHDRAWAL FROM THE STUDY....49
7.3 LOST TO FOLLOW UP....50
8 STUDY ASSESSMENTS AND PROCEDURES....51
8.1 EFFICACY ASSESSMENTS....51
8.1.1 Worst-itch numeric rating scale....52
8.1.2 Investigator’s global assessment for prurigo nodularis....52
8.1.3 Prurigo activity score....52
8.1.4 Dermatology life quality index....53
8.1.5 Pain and sleep numeric rating scales....53
8.1.6 Hospital anxiety and depression scale....53
8.1.7 Patient Global Impression of Change of disease and Patient Global Impression of Severity....54
8.1.8 Euroqol 5 dimensions questionnaire....54
8.1.9 Missed school/work days....54
8.1.10 Photography....55
8.2 SAFETY ASSESSMENTS....55
8.2.1 Physical examinations....55
8.2.2 Vital signs....55
8.2.3 Electrocardiograms....55

8.2.4 Clinical safety laboratory assessments 55

8.3 ADVERSE EVENTS AND SERIOUS ADVERSE EVENTS 56

8.3.1 Time period and frequency for collecting AE and SAE information 57

8.3.2 Method of detecting AEs and SAEs 58

8.3.3 Follow-up of AEs and SAEs 58

8.3.4 Regulatory reporting requirements for SAEs 58

8.3.5 Pregnancy 59

8.3.6 Guidelines for reporting product complaints 59

8.4 TREATMENT OF OVERDOSE 59

8.5 PHARMACOKINETICS 60

8.5.1 Systemic drug concentration and antidrug antibodies 60

8.5.1.1 Sampling time 60

8.5.1.2 Handling procedures 60

8.5.1.3 Bioanalytic method 60

8.6 PHARMACODYNAMICS 61

8.7 GENETICS 61

8.8 BIOMARKERS 61

8.9 MEDICAL RESOURCE UTILIZATION AND HEALTH ECONOMICS 62

9 STATISTICAL CONSIDERATIONS 63

9.1 STATISTICAL HYPOTHESES 63

9.2 SAMPLE SIZE DETERMINATION 63

9.3 POPULATIONS FOR ANALYSES 64

9.4 STATISTICAL ANALYSES 64

9.4.1 Efficacy analyses 64

9.4.2 Safety analyses 67

9.4.3 Other analyses 68

9.5 INTERIM ANALYSES 68

9.6 TIMING OF PRIMARY DATABASE LOCK 68

9.7 DATA MONITORING COMMITTEE (DMC) 68

9.8 UNBLINDING PLAN 68

10 SUPPORTING DOCUMENTATION AND OPERATIONAL CONSIDERATIONS 69

10.1 APPENDIX 1: REGULATORY, ETHICAL, AND STUDY OVERSIGHT CONSIDERATIONS.......69
10.1.1 Regulatory and Ethical Considerations.......69
10.1.2 Financial disclosure.......69
10.1.3 Informed consent process.......70
10.1.4 Data protection.......70
10.1.5 Committees structure.......71
10.1.6 Dissemination of clinical study data.......71
10.1.7 Data quality assurance.......72
10.1.8 Source documents.......72
10.1.9 Study and site closure.......73
10.1.10 Publication policy.......73
10.2 APPENDIX 2: CLINICAL LABORATORY TESTS.......74
10.3 APPENDIX 3: ADVERSE EVENTS: DEFINITIONS AND PROCEDURES FOR RECORDING, EVALUATING, FOLLOW-UP, AND REPORTING.......76
10.4 APPENDIX 4: CONTRACEPTIVE GUIDANCE AND COLLECTION OF PREGNANCY INFORMATION.......80
10.5 APPENDIX 5: GENETICS.......82
10.6 APPENDIX 6: LIVER AND OTHER SAFETY: SUGGESTED ACTIONS AND FOLLOW-UP ASSESSMENTS.......84
10.7 APPENDIX 7: CLINICIANS-REPORTED OUTCOMES AND PATIENT-REPORTED OUTCOMES.......87
10.7.1 Worst itch numeric rating scale (WI-NRS).......87
10.7.2 Investigator's global assessment of prurigo nodularis (IGA PN).......88
10.7.3 Prurigo activity score (PAS).......89
10.7.4 Dermatology life quality index (DLQI).......91
10.7.5 Hospital anxiety and depression scale (HADS).......92
10.7.6 EQ-5D-5L.......93
10.7.7 Pain numeric rating scale.......94
10.7.8 Sleep numeric rating scale.......95
10.7.9 Patient Global Impression of Change of disease (PGIC).......95
10.7.10 Patient Global Impression of Severity (PGIS).......96
10.7.11 Missed school/work days.......97
10.8 APPENDIX 8: DEFINITION OF ANAPHYLAXIS.......101
10.9 APPENDIX 9: LIST OF OPPORTUNISTIC INFECTIONS.......101
10.10 APPENDIX 10: COUNTRY-SPECIFIC REQUIREMENTS.......102

10.11 APPENDIX 11: ABBREVIATIONS 102

10.12 APPENDIX 12: PROTOCOL AMENDMENT HISTORY 104

10.12.1 Amended protocol 01 (20 May 2020) 104

10.12.2 Amended protocol 02 (14 April 2021) 108

11 REFERENCES 113

LIST OF TABLES

Table 1 - Objectives and endpoints 28

Table 2 - Overview of study interventions administered 40

Table 3 - Summary of handling procedures 60

Table 4 - Summary of bioanalytical methods for functional dupilumab and anti-dupilumab antibodies 60

Table 5 - Populations for analyses 64

Table 6 - Efficacy analyses 65

Table 7 - Safety analyses 67

Table 8 - Other analyses 68

Table 9 - Protocol-required laboratory assessments 74

1 PROTOCOL SUMMARY

1.1 SYNOPSIS

Protocol title:

A randomized, double blind, placebo-controlled, multi-center, parallel group study to evaluate the efficacy and safety of dupilumab in patients with prurigo nodularis who are inadequately controlled on topical prescription therapies or when those therapies are not advisable

Short title:

Study of dupilumab for the treatment of patients with prurigo nodularis, inadequately controlled on topical prescription therapies or when those therapies are not advisable

LIBERTY-PN PRIME (PRurigo Nodularis Itch Minimization Evaluation)

Rationale:

Prurigo nodularis (PN) is a skin disease characterized by multiple, intensely itchy skin eruptions in symmetrically distributed areas of the extremities (1). The main symptom is prolonged, repetitive and uncontrollable rubbing, scratching and uncontrollable itching which leads to hyperkeratotic eroding papules and nodules on the skin. It is difficult to treat and entails a high disease burden. Approximately 50% of patients have either past or current history of atopic dermatitis (AD) or other atopic disorders (2). While case reports suggest that dupilumab may successfully treat patients with PN regardless of a preexisting atopic background (3, 4, 5, 6, 7), it remains unclear to what extent type 2 cytokines play a role in the pathogenesis in those PN lesions not associated with atopy (8).

There are no United States (US) Food and Drug Administration (FDA) or European Medicines Agency (EMA) approved targeted therapies indicated for the treatment of PN, whether atopic or non-atopic forms. Topical corticosteroids (TCS) and topical calcineurin inhibitors (TCI) are often used initially. While there is a mechanistic rationale for their use, no rigorous clinical studies confirming their efficacy were identified.

Dupilumab is a human monoclonal IgG4 antibody that inhibits interleukin (IL)-4 and IL-13 signaling by specifically binding to the IL-4Rα subunit shared by the IL-4 and IL-13 receptor complexes. Dupilumab inhibits IL-4 signaling via the Type I receptor and both IL-4 and IL-13 signaling through the Type II receptor.

Dupilumab has shown clinical efficacy in multiple diseases with underlying type 2 inflammation such as AD, asthma, chronic rhinosinusitis with nasal polyposis (CRSwNP), and eosinophilic esophagitis (EoE).

Recently, case series were published that suggest dupilumab may also be effective in the treatment of PN (3, 4, 5, 6, 7).

Type 2 cytokine involvement is implicated to some extent in all forms of PN. It is postulated that dupilumab may play a fundamental role in pruritus based upon its ability to block IL4R activity at the level of the sensory dorsal root ganglion (9). The present protocol will evaluate the efficacy and safety of dupilumab in PN.

Objectives and endpoints

Objectives	Endpoints
**Primary**	
• To demonstrate the efficacy of dupilumab on itch response in patients with PN, inadequately controlled on topical prescription therapies or when those therapies are not advisable.	• Proportion of participants with improvement (reduction) in worst-itch numeric rating scale (WI-NRS) by ≥4 from baseline to Week 24.
**Secondary**	
• To demonstrate the efficacy of dupilumab on additional itch endpoints in patients with PN, inadequately controlled on topical prescription therapies or when those therapies are not advisable.	• Time to onset of effect on pruritus as measured by proportion of participants with an improvement (reduction) in WI-NRS by ≥4 from baseline during the 24-week treatment period.<br>• Change from baseline in WI-NRS at Week 24.<br>• Change from baseline in WI-NRS at Week 12.<br>• Percent change from baseline in WI-NRS at Week 24.<br>• Percent change from baseline in WI-NRS at Week 12.<br>• Percent change from baseline in WI-NRS at Week 4.<br>• Percent change from baseline in WI-NRS at Week 2.<br>• Percent change from baseline in WI-NRS over time until Week 24.<br>• Proportion of participants with improvement (reduction) in WI-NRS ≥4 from baseline to Week 12.<br>• Proportion of participants with WI-NRS reduction ≥4 at Week 4.<br>• Proportion of participants with WI-NRS reduction ≥4 over time until Week 24.<br>• Onset of action in change from baseline in WI-NRS (first p <0.05 difference from placebo in the daily WI NRS that remains significant at subsequent measurements) until Week 12.
• To demonstrate efficacy of dupilumab on skin lesions of PN.	• Proportion of participants with Investigator's Global Assessment 0 or 1 score for PN-Stage (IGA PN-S) at Week 24 [Key secondary endpoint].<br>• Proportion of participants with IGA PN-S 0 or 1 score at Week 12.<br>• Proportion of participants with IGA PN-S 0 or 1 score at Week 8.<br>• Proportion of participants with IGA PN-S 0 or 1 score at Week 4.<br>• Change from baseline in IGA PN-S score at Week 24.<br>• Change from baseline in IGA PN-S score at Week 12.<br>• Change from baseline in IGA PN-S score at Week 8.

Objectives	Endpoints
	• Change from baseline in IGA PN-S score at Week 4.<br>• Proportion of participants with Investigator's Global Assessment 0 or 1 score for PN-Activity (IGA PN-A) at Week 24.<br>• Proportion of participants with IGA PN-A 0 or 1 score at Week 12.<br>• Proportion of participants with IGA PN-A 0 or 1 score at Week 8.<br>• Proportion of participants with IGA PN-A 0 or 1 score at Week 4.
• To demonstrate the improvement in health-related quality of life (HRQoL).	• Change from baseline in HRQoL, as measured by Dermatology Life Quality Index (DLQI) to Week 24.<br>• Change from baseline in HRQoL, as measured by DLQI to Week 12.
• To evaluate safety outcome measures.	• Percentage of participants experiencing treatment-emergent adverse events (TEAEs) or serious adverse events (SAEs) from baseline through Week 24.
• To evaluate immunogenicity of dupilumab.	• Incidence of treatment-emergent antidrug antibodies (ADA) against dupilumab over time.

Overall design:

This study is a multi-center, 24-week treatment, parallel, double-blind, randomized, placebo-controlled study to evaluate the use of dupilumab in patients with PN inadequately controlled on topical prescription therapies or when those therapies are not advisable. The study will assess the effect of dupilumab on itch improvement as well as its effect on PN lesions, on patients' HRQoL, anxiety and depression, sleep quality and skin pain, and overall health status.

Disclosure Statement: This is a Parallel, Treatment study, with 2 arms, that is blinded/masked for participants and investigators.

Number of participants:

Approximately 150 participants will be randomized 1:1. This corresponds to approximately 75 participants who will be randomly assigned to each intervention arm.

Intervention groups and duration:

Participants who satisfy the inclusion and exclusion criteria will be randomized (1:1) to one of the following investigational medicinal product (IMP) treatment groups:

- Dupilumab 300 mg
- Matched placebo

Duration of study period (per participant)

- Screening period (2-4 weeks)
- Randomized IMP intervention period (24 weeks)
- Follow-up period (12 weeks)

Study interventions

Investigational medicinal product:

Dupilumab 300 mg and placebo matching dupilumab 300 mg supplied in prefilled syringes that are visually indistinguishable.

Dupilumab

- Formulation: dupilumab 300 mg: a 150 mg/mL dupilumab solution in a pre-filled syringe to deliver 300 mg in a 2 mL injection.
- Route of administration: subcutaneous (SC) injection.
- Dose regimen: 300 mg every 2 weeks (Q2W) after an initial loading dose of 600 mg (2 injections of 300 mg) on Day 1.

Placebo

- Formulation: identical formulation to the active 300 mg formulation without dupilumab, in a pre-filled syringe to deliver placebo in a 2 mL injection.
- Route of administration: SC injection.
- Dose regimen: 1 injection Q2W after an initial loading dose (2 injections) on Day 1.

Noninvestigational medicinal products

Participants will be required to apply moisturizers (emollients) once or twice daily for at least 5 out of the 7 consecutive days immediately before Day 1 and continue until Week 36.

If participants are on a stable regimen of low to medium potency TCS or TCI at the screening visit, they can continue their topical steroid application once daily without tapering from Screening to Week 24. If specific lesions resolve, the participant can stop applying steroids to those sites but are permitted to continue applying to persistent lesions. If participants are on stable regimens of high potency or superpotent steroids, participants should decrease potency to medium potency TCS and continue to apply daily from screening to Week 24. Occlusion is not allowed from Screening to Week 24.

Participants can be rescued with high potency or superpotent TCS/TCI as needed throughout the study.

Post-trial access to study medication

The sponsor does not plan to provide post-trial access to the study medication.

Statistical considerations:

- **Randomization**
  - The participants will be randomized to dupilumab or placebo in 1:1 ratio with stratification factors of documented history of atopy (atopic or non-atopic), stable use of TCS/TCI (yes or no), and country/territory code. Number of participants with active mild AD upon study entry will represent up to 10% of the atopic participants. Both the atopic and the non-atopic PN population will be capped at 60% of the total enrolled population.
- **Sample size calculation**
  - The primary endpoint is the proportion of participants with a WI-NRS reduction of ≥4 from baseline to Week 24. Assuming a response rate of [redacted] and [redacted] in placebo and dupilumab respectively, 56 participants/arm will provide 90% power to detect the difference of [redacted] between dupilumab and placebo with Fisher exact test at 2-sided level of 0.05. Assuming 15% drop out during treatment, the target is to randomize 75 participants/arm to allow for up to 10% of atopic patients having active mild AD upon study entry.
- **Primary analysis**
  - The analysis population for the efficacy endpoints will be the intent-to-treat (ITT) population defined as all randomized participants analyzed according to the treatment group allocated by randomization regardless if treatment kit is used or not.
- **Analysis of primary endpoint**
  - The primary analysis on WI-NRS reduction ≥4 at Week 24 will be conducted by using Cochran-Mantel-Haenszel (CMH) test stratifying by stratification factors (documented history of atopy [atopic or non-atopic], stable use of TCS/TCI [yes or no], and region [countries combined]) and covariate of baseline anti-depressant use (yes or no). For participants discontinuing the study treatment before Week 24, their off-study treatment values measured up to Week 24 will be included in the analysis. Participants taking selected prohibited medications and/or rescue medications (details of selection will be specified in the statistical analysis plan [SAP]) prior to Week 24 or have missing data at Week 24 will be considered non-responders.
  - Sensitivity analyses using alternative methods will be performed to handle missing data and/or data collected after participants taking selected prohibited medications and/or rescue medications. A subgroup analysis will be performed excluding participants with a current diagnosis of AD. More details of the sensitivity and subgroup analyses will be specified in the SAP.
- **Analysis of secondary endpoints**
  - Secondary efficacy endpoints that measure binary responses will be analyzed in the same fashion as the primary endpoint.
  - Time-to-event secondary efficacy endpoint will be analyzed using the Cox proportional hazards model, including treatment, stratification factors (countries combined to region), and covariate of baseline anti-depressant use (yes or no). The

hazards ratio, its 95% confidence interval and p-value will be reported. Kaplan-Meier curves will be also provided.

- Continuous secondary efficacy endpoints will be analyzed using a hybrid method of the worst observation carried forward (WOCF) and multiple imputations. Data of participants taking selected prohibited medications and/or rescue medications will be set to missing after the medication usage, and the worst postbaseline value on or before the time of the medication usage will be used to impute missing endpoint value (for participants whose postbaseline values are all missing, the baseline will be used to impute). Participants who discontinue the treatment prematurely are encouraged to follow the planned clinical visits and in those participants who did not take the selected prohibited medications and/or rescue medications, all data collected after treatment discontinuation will be used in the analysis. For these participants, missing data may still happen despite all efforts have been tried to collect the data after treatment discontinuation. For participants who discontinue due to lack of efficacy, all data collected after discontinuation will be used in the analysis, and a WOCF approach will be used to impute missing data if needed. For participants who discontinued not due to lack of efficacy, a multiple imputation approach will be used to impute missing endpoint value, and this multiple imputation will use all participants excluding participants who have taken the selected prohibited medications and/or rescue medications prior to timepoint of endpoint of interest and excluding patients who discontinue due to lack of efficacy. Each of the imputed complete data will be analyzed by fitting an analysis of covariance (ANCOVA) model with treatment group, stratification factors (countries combined into region), baseline anti-depressant use (yes or no), and relevant baseline measurement as covariates in the model. Statistical inference obtained from all imputed data will be combined using Rubin's rule.
- The safety variables will be summarized using descriptive statistics.
- Antidrug antibodies variables including treatment-emergent ADA will be summarized using descriptive statistics by treatment group.

- **Multiplicity considerations**
  - The multiplicity procedure is proposed to control the overall Type-I error rate for testing the primary endpoint and the key secondary endpoints. Detailed hierarchical testing procedure will be defined in the study SAP. The study is considered positive when the primary endpoint achieves statistical significance.
- **Planned database lock date**
  - A primary database lock is currently planned to be performed when all randomized participants have completed their 24-week treatment period.
  - The database will be updated at the end of the study for all participants to include the post-treatment follow-up information and updates for the events previously ongoing at the time of the primary lock. Additional data collected between the primary database lock and last participant completing last visit will be summarized in a separate clinical study report (CSR) addendum, as needed. Details will be included in the SAP.

- **Unblinding plan**
    - Unblinding plan is not applicable for this study.

Data Monitoring Committee: No

1.2 SCHEMA

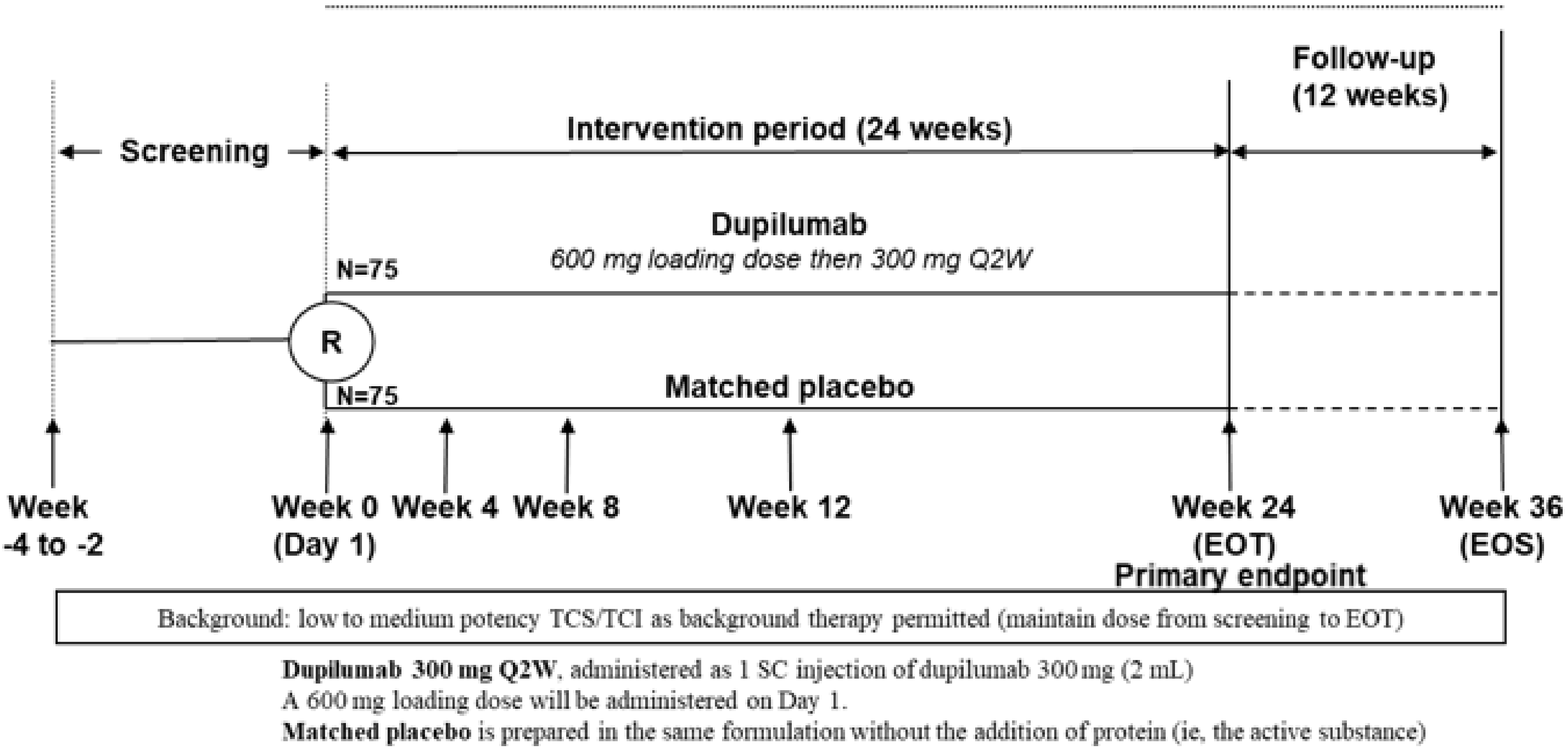

EOS: end of study; EOT: end of treatment; PN: prurigo nodularis; Q2W: every 2 weeks; R: randomization; SC: subcutaneous; TCI: topical calcineurin inhibitors; TCS: topical corticosteroids.

1.3 SCHEDULE OF ACTIVITIES (SOA)

Procedure	Screening (2 to 4 weeks before Day 1)	Intervention period (Weeks)					Follow-up (12 weeks)	Notes
		0 (Day 1)	4	8	12	24		
**Visit**	1	2[a]	3	4	5	6[b] (EOT)	7 (EOS)	Visit window: ±3 days for Visits 3, 4, 5, 6, and 7.
**Screening and baseline**								
Informed consent	X							Separate consent to be obtained for optional procedures (skin biopsy, DNA and RNA sampling, serum/plasma sampling for archival, and photography; HIV test if specific consent locally required).
Medical history	X							
Prior and concomitant medication	X	X	X	X	X	X	X	Concomitant medication will be collected throughout the study.
Demography	X							
Inclusion and exclusion criteria	X	X						
Hepatitis[c], HIV serology, TB test	X							HBs Ag, HBs Ab, HBc Ab, HCV Ab, HIV screen (Anti-HIV-1 and HIV-2 antibodies), TB test (performed locally if required and results noted in the eCRF).
Randomization		X						
**Study intervention[d]**								
Dispense or download e-diary	X	X	X	X	X	X	X	Device will be dispensed at Screening (including instructions for use). At EOS, the e-diary will be downloaded and returned to the site.
Participant e-diary training	X	X						
Call IVRS/IWRS	X	X	X	X	X	X	X	At screening visit, IVRS/IWRS will be called after medical history check.
IMP administration		X	X	X	X			IMP administration Q2W (±3 days) from Week 0 until Week 22. Between visits, the participants will self-inject IMP at home or may choose to have injections administered at home by a caregiver or at the study site.

Procedure	Screening (2 to 4 weeks before Day 1)	Intervention period (Weeks)					Follow-up (12 weeks)	Notes
		0 (Day 1)	4	8	12	24		
**Visit**	1	2[a]	3	4	5	6[b] (EOT)	7 (EOS)	Visit window: ±3 days for Visits 3, 4, 5, 6, and 7.
**Safety[d]**								
Physical examination	X	X				X	X	Including skin (full body skin exam), nasal cavities, eyes, ears, respiratory, cardiovascular, gastrointestinal, neurological, lymphatic, and musculoskeletal systems.
Vital signs	X	X	X	X	X	X	X	Including SBP and DBP (mmHg), pulse rate (beats per minute), body temperature (°C), and respiratory rate. Body weight will be recorded at Visits 1, 6, and 7 only. Height will be recorded at Visit 1 only.
12-lead ECG	X					X		Locally collected and read.
Hematology, biochemistry[e]	X	X	X	X	X	X	X	Including hemoglobin, hematocrit, platelet count, total WBC count, differential count, total RBC count, creatinine, BUN, glucose, lactate dehydrogenase, uric acid, total cholesterol, total protein, albumin, total bilirubin, ALT, AST, alkaline phosphatase, electrolytes (sodium, potassium, chloride), bicarbonate, creatine phosphokinase, and hemoglobin A1c (only for diabetic patients without hemoglobin A1c laboratory results within 3 months before screening visit).
Urinalysis	X	X	X	X	X	X		Dipstick analysis including specific gravity, pH, glucose, ketones, blood, protein, nitrite, leukocyte esterase, urobilinogen and bilirubin. If positive for protein and/or red blood cells, microscopic analysis will be performed by the central laboratory. Creatinine, leukotriene and tetranor PGDM will be tested by the central laboratory. Urine sample has to be collected at the same time (±1 hour) established at V2 in a given patient, if possible.
CD4 T cell count and HIV viral load	X					X		For participants with HIV history only.
Pregnancy test (WOCBP only)[g]	Serum	Urine	Urine	Urine	Urine	Urine	Urine	In between visit urine pregnancy tests will be performed at home (Weeks 16, 20, 28, 32).
AE reporting, including SAEs	X	X	X	X	X	X	X	

Procedure	Screening (2 to 4 weeks before Day 1)	Intervention period (Weeks)					Follow-up (12 weeks)	Notes
		0 (Day 1)	4	8	12	24		
**Visit**	1	2[a]	3	4	5	6[b] (EOT)	7 (EOS)	Visit window: ±3 days for Visits 3, 4, 5, 6, and 7.
Rescue medication use		←--→						
**PK and ADA**[d, f]								
Serum PK samples for dupilumab concentration		X	X	X	X	X	X	
Anti-dupilumab antibody		X			X	X	X	
**Biomarkers**[d]								
Total serum IgE		X	X	X	X	X	X	
Optional skin biopsy (substudy)		X				X		The sample will be taken from lesion and non-lesion skin using punch biopsy.
Optional serum/plasma for archival samples		X	X	X	X	X	X	Archive serum and plasma samples will be collected and stored for possible future analysis of potential biomarkers of drug response, disease activity, safety, and the type 2 inflammation pathway.
Optional DNA (whole blood) sample[h]		X						
Optional RNA (whole blood) sample[h]		X				X		
**Efficacy**[d]								
WI-NRS	NRS: once a day from Screening to EOS							To be recorded once a day in e-diary.
Pain-NRS								
Sleep-NRS								

Procedure	Screening (2 to 4 weeks before Day 1)	Intervention period (Weeks)					Follow-up (12 weeks)	Notes
		0 (Day 1)	4	8	12	24		
**Visit**	1	2[a]	3	4	5	6[b] (EOT)	7 (EOS)	Visit window: ±3 days for Visits 3, 4, 5, 6, and 7.
DLQI		X	X	X	X	X	X	Entered by participant during site visit on a tablet provided to the site.
HADS		X			X	X	X	
EQ-5D-5L		X			X	X	X	
PGIS	X	X	X	X	X	X		
PGIC			X	X	X	X		
Missed school/work days (baseline version)		X						
Missed school/work days (postbaseline version)					X	X	X	
Modified PAS (screening-baseline version)	X	X						Investigator's assessment to be entered on a tablet provided to the site.
Modified PAS (follow-up version)			X	X	X	X	X	
IGA PN-A and IGA PN-S		X	X	X	X	X	X	
Optional photography (substudy)		X	X	X	X	X	X	

a All assessments at Visit 2 (Day 1) are to be conducted pre-IMP dose with the exception of the assessment of local tolerability of SC injections.

b Participants who discontinue the study treatment prematurely (prior to completing the 24-week treatment period) will perform the EOT assessments at the time of discontinuation to assure a complete clinical assessment in close temporal proximity to the premature termination of study treatment. In addition, to allow assessment of participant outcomes over the stipulated study period, participants will be asked and encouraged to complete all remaining study visits and participate in all assessments according to the visit schedule.

c In case of results showing HBs Ag (negative) and HBc Ab (positive), an HBV DNA testing will be performed and should be confirmed negative prior to randomization. In case of results showing HCV Ab (positive), HCV RNA testing will be performed and should be confirmed negative prior to randomization.

d Assessments/procedures should be conducted in the following order: PRO, investigator assessments, safety and laboratory assessments (including sample collection for ADA, PK, biomarker, and optional DNA and RNA), and administration of IMP.

e Refer to Section 10.2 and central lab manual for collection details.

f PK and ADA samples to be collected prior to the administration of the drug. In the event of any SAE, any AE of severe injection site reaction lasting longer than 24 hours, or any AESI of anaphylactic reaction or systemic allergic reaction that is related to IMP and require treatment, PK and ADA samples will be collected at or near the onset of the event for any additional analysis if required or for archival purposes. Samples for ADA will only be collected for analysis in the event of any SAE for participants from China sites.

g Pregnancy will lead to definitive treatment discontinuation in all cases. Female (WOCBP) participants will be supplied with urine dipsticks for use between visits and complete the Home pregnancy diary.

h DNA sample should be collected at the Day 1 visit, but can be collected at any visit during the study. RNA samples must be collected before the administration of the first dose of study drug and at Week 24.

ADA: antidrug antibodies; AE: adverse event; AESI: adverse event of special interest; ALT: alanine aminotransferase; AST: aspartate aminotransferase; BUN: blood urea nitrogen; DBP: diastolic blood pressure; DLQI: Dermatology Life Quality Index; DNA: deoxyribonucleic acid; ECG: electrocardiogram; eCRF: electronic case report form; EOS: end of study; EOT: end of treatment; EQ-5D-5L: Euroqol 5 dimensions 5 levels; HADS: Hospital Anxiety and Depression Scale; HBc Ab: hepatitis B core antibody; HBs Ab: hepatitis B surface antibody; HBs Ag: hepatitis B surface antigen; HBV: hepatitis B virus; HCV: hepatitis C virus; HCV Ab: hepatitis C virus antibody; HIV: human immunodeficiency virus; Ig: immunoglobulin; IGA PN: Investigator's Global Assessment for prurigo nodularis; IGA PN-A: IGA PN-Activity; IGA PN-S: IGA PN-Stage; IMP: Investigational Medicinal Product; IVRS: interactive voice response system; IWRS: interactive web response system; NRS: numeric rating scale; PAS: prurigo activity score; PGIC: Patient Global Impression of Change; PGIS: Patient Global Impression of Severity; PK: pharmacokinetic; PRO: patient reported outcome; Q2W: every 2 weeks; RBC: red blood cells; RNA: ribonucleic acid; SAE: serious adverse event; SBP: systolic blood pressure; SC: subcutaneous; TB: tuberculosis; WBC: white blood cells; WI-NRS: worst itch numeric rating scale; WOCBP: woman of childbearing potential.

2 INTRODUCTION

Dupilumab is a human monoclonal IgG4 antibody that inhibits IL-4 and IL-13 signaling by specifically binding to the IL-4Rα subunit shared by the IL-4 and IL-13 receptor complexes. Dupilumab inhibits IL-4 signaling via the Type I receptor and both IL-4 and IL-13 signaling through the Type II receptor. Blocking IL-4Rα with dupilumab inhibits IL-4 and IL-13, key cytokines that drive type 2 inflammatory responses, including the release of proinflammatory cytokines, chemokines, nitric oxide, and IgE.

Dupilumab is approved by major regulatory agencies for use in AD and in asthma in adults and adolescents, and in the US for CRSwNP in adults.

2.1 STUDY RATIONALE

Prurigo nodularis is a skin disease characterized by multiple, intensely itchy skin eruptions in symmetrically distributed areas of the extremities (1). It is difficult to treat and entails a high disease burden.

Dupilumab has shown efficacy in multiple diseases with underlying type 2 inflammation such as AD, asthma, CRSwNP and EoE. In light of the published research suggesting a principal role for type 2 cytokines in the pathogenesis of PN (see Section 2.2), the Sponsor proposes that selective and simultaneous inhibition of IL-4 and IL-13 signaling such as that achieved with dupilumab treatment may provide clinical benefit in relieving the signs and symptoms of PN, especially in the high unmet need population of patients inadequately treated with, or ineligible for, TCS.

Recently, case series were published that suggest that dupilumab may also be effective in the treatment of PN (3, 4, 5, 6). Type 2 cytokine involvement is implicated in all forms of PN. This is consistent with a recent case series of nine patients without an atopic history who benefitted from dupilumab treatment (7).

The primary hypothesis of the study is that dupilumab will be effective in reducing itch and the secondary hypothesis is that it will reduce and promote the healing of lesions of PN, in patients who are inadequate responders to topical prescription therapies or when those therapies are not advisable.

2.2 BACKGROUND

Prurigo nodularis is a skin disease characterized by multiple, intensely itchy skin eruptions in symmetrically distributed areas of the extremities (1). The main symptom is prolonged, repetitive and uncontrollable rubbing, scratching and uncontrollable itching which leads to hyperkeratotic eroding papules and nodules on the skin. A broadly accepted definition for chronic prurigo has been published by the European Academy of Dermatology and Venereology (EADV) (10). The convened experts agreed that chronic prurigo should be used as an umbrella term for the range of clinical manifestations (eg, papular, nodular, plaque or umbilicated types). Prurigo nodularis is

considered a distinct disease defined by the presence of chronic pruritus for ≥6 weeks, history and/or signs of repeated scratching and multiple localized/generalized pruriginous skin lesions (whitish, hyperpigmented, or pink papules, nodules and/or plaques).

Pathophysiological studies in PN patients reveal increased dermal nerve fiber density and changes in many types of skin cells. These skin cells cause inflammation and pruritus (severe, constant itching) through the release of cytokine IL-31, tryptase, prostaglandins, eosinophil cationic protein, histamine, and neuropeptides, such as substance P, calcitonin gene-related peptide and nerve growth factor (11). The itch caused by primary dermatoses or systemic disorders is postulated to cause an itch scratch cycle which leads to neuronal hypersensitization. This neuronal hypersensitization is the final common pathway which drives the symptomatology of PN (1). Fukushi et al. studied 22 PN skin biopsies and found that 86% of them showed activation of STAT6, a marker for type 2 inflammatory pathway activation downstream of the type 2 cytokines IL-4 and IL-13 (12). Consistent with this, IL-4 and IL-5 messenger RNA expression is upregulated in the skin of PN patients (13). Importantly, these two studies did not mention whether these skin biopsy specimens came from patients with or without atopy. The etiology of the profound pruritus in this disease is thought to be driven by high type 2 inflammation driving activity at the level of the sensory neuron. Human sensory neurons in skin that transmit the sensation of itch are known to express IL- 4Rα, IL-13Ra1, and IL-31RA (14). On testing for functional stimulation, IL-4 was shown to enhance responsiveness to other pruritogens like histamine (14). These findings support that type 2 cytokines may play a principal role in the pathogenesis of PN.

Due to the central manifestation of itch, PN carries a significant burden of disease (10, 15). The effect on quality of life due to PN has been reported to be higher than other common skin disorders like AD and psoriasis (16). Patients report chronic sleep loss due to constant itching; constant burning, stinging, and pain at affected area; and chronic depression, anxiety, anger, disgust, and shame; and hence overall experience a great impact on their quality of life. According to a 5 year cross-sectional study on 909 adult PN patients in the John Hopkins hospital system (15), PN is a key contributing factor to mood disorders such as anxiety and depression. Of all patients with chronic pruritus, 70% have some type of psychiatric comorbidity, and the severity of depression in patients with chronic pruritus correlates with intensity of itch. The concomitant psychologic component of PN likely contributes to the challenging nature of treating this disease.

Data on the epidemiology of PN are limited. Most studies show a predominance of older patients with a median age of more than 50 years (2, 10, 15). Prurigo nodularis is only occasionally observed in younger patients, in whom it is often associated with atopic conditions (17).

There are no FDA or EMA approved targeted therapies indicated for the treatment of PN, whether atopic or non-atopic forms; the lesions of atopic and non-atopic PN are clinically indistinguishable and treatment options remain the same. Topical corticosteroids and TCI are often used initially. While there is a mechanistic rationale for their use, no rigorous clinical studies confirming their efficacy were identified. Anecdotal feedback obtained from dermatological experts indicate variable, modest efficacy. For thicker lesions, TCS is also administered intralesionally. Lesional cryotherapy is another available topical treatment; case reports indicate temporary relief. Antihistamines and antileukotrienes are occasionally used; their efficacy, however, is not supported by well conducted, randomized clinical trials and is rated low

by patients (18). Phototherapy, in particular narrowband ultraviolet B (UVB), is occasionally added in patients not responding to topical pharmacotherapy. While some improvements with phototherapy have been reported, they remain usually partial and complete remissions are uncommon.

Oral immunosuppressants such as methotrexate and cyclosporine have been used off-label with some success as reported based on case reports and retrospective (18). Use of cyclosporine in PN is limited by commonly recognized toxicities including hypertension, impaired renal and hepatic function, and potential for increased susceptibility to infections and cancer, particularly skin cancer, due to decreased cancer immunosurveillance (19). Methotrexate has well established toxicities, in particular, myelosuppression and hepatotoxicity. In addition, the broad immunosuppression caused by all these drugs carries an increased risk of developing serious bacterial, fungal, viral, and mycobacterial infections.

Neuromodulatory agents such as gabapentin and anti-inflammatory agents such as thalidomide have been used in PN with varying degrees of success (18), but have also considerable adverse effects. Adverse effects of thalidomide include peripheral neuropathies, sedation, dizziness and teratogenicity, while adverse effects of gabapentin include headache, sedation and dizziness.

Overall, despite the use of multiple treatments, many patients with PN remain uncontrolled, and some of the available therapies are associated with serious potential adverse reactions. Given the lack of targeted treatments and the suboptimal efficacy associated with currently available therapies, there remains a significant unmet need in patients with PN.

Dupilumab has shown efficacy in multiple diseases with underlying type 2 inflammation such as AD, asthma, CRSwNP, and EoE. The most compelling data suggesting that PN is driven by type 2 inflammation are the many case series of PN patients successfully treated with dupilumab. Recently, five case series were published that suggest that dupilumab may also be effective in the treatment of PN: Mollanazar et al. (4 patients, 3 of those without history of AD) (3), Beck et al. (3 patients) (4), Rambhia et al. (2 patients) (5), Calugareanu et al. (one patient) (6), and Zhai et al. (9 patients, all without history of AD) (7). These case series reported major improvements in treatment-refractory cases of PN after treatment with dupilumab 300 mg Q2W (after an initial loading dose of 600 mg). These published case reports show rapid and substantial effects of dupilumab on itch, and somewhat less well documented effects also on lesions in patients with PN refractory to standard treatment.

2.3 BENEFIT/RISK ASSESSMENT

Dupilumab solution for injection is currently authorized:

- In over 40 countries worldwide including the US, European Union (EU) (Centralised Procedure), and Japan for the treatment of adults with inadequately controlled moderate-to-severe AD. In the US and EU, it has also been authorized for use in adolescent patients (≥12 years) with inadequately controlled moderate-to-severe AD.

- In the US for use in moderate-to-severe eosinophilic or oral steroid dependent asthma, in the EU for severe type 2 asthma in adults and adolescents, and in Japan for use in adults and adolescents (≥12 years) with severe or refractory bronchial asthma,
- In the US for use in adults with inadequately controlled CRSwNP.

Dupilumab has shown clinically relevant benefit in several type 2 driven immunological disorders such as AD, bronchial asthma, CRSwNP, and EoE. In asthma and AD indications, studies were also conducted in adolescents and similar benefit to adults was observed.

While the mechanism of PN is not well-known, there is some evidence of a type 2 immunologic signature in prurigo nodule skin biopsy specimens and activation of type 2 transcription factors STAT3 and STAT6 in the epidermis (12, 13). Therefore, it is hypothesized that dupilumab may show significant clinical benefit in the treatment of PN. This hypothesis is supported by a series of case reports that show dupilumab treatment resulting in a significant reduction in pruritus in 19 PN patients (3, 4, 5, 6, 7).

No tissue targets or specific hazards to humans were identified in nonclinical general and reproductive toxicology studies.

Dupilumab has an extensive safety database. As of the 28 March 2019 (data lock point), 8798 subjects were enrolled into the development program for dupilumab and are included in the safety population treated: 242 as healthy volunteers, 4227 from AD studies, 3377 from asthma studies, 782 from CRSwNP studies, 53 from EoE studies, 117 from grass allergy and peanut allergy studies. The number of subjects exposed to dupilumab in clinical studies was 7781 (218 in healthy volunteer studies, 3931 in AD studies, 3073 in asthma studies, 470 in CRSwNP studies, 26 in EoE studies, and 63 in grass allergy and peanut allergy studies). Based on the sales figure retrieved from Intercontinental Marketing Services Health and using the World Health Organization’s defined average daily dose for dupilumab of 21.4 mg, the cumulative post marketing exposure to dupilumab is estimated to be 38 816 patient years (01 January 2017 through 31 December 2018).

Dupilumab was generally well tolerated in all populations tested in clinical development programs. The adverse drug reactions (ADRs) identified to date for dupilumab include injection site reactions, conjunctivitis, oral herpes, conjunctivitis allergic, conjunctivitis bacterial, herpes simplex, blepharitis, dry eye, eye pruritus, and eosinophilia. These ADRs occur with relatively low frequency with dupilumab treatment, and were generally mild or moderate, transient, and manageable. More significant serious allergic reactions were very rare. Importantly, no increased overall infection risk was observed in patients treated with dupilumab.

Systemic hypersensitivity has been established as an important identified risk with dupilumab. As protein therapeutics, all monoclonal antibodies are potentially immunogenic. Rare serious and systemic hypersensitivity reactions have been observed in the dupilumab program including serum sickness/serum sickness-like reaction in the adult AD program and anaphylaxis related to dupilumab in the adult asthma clinical trials.

It is hypothesized that dupilumab in patients with PN will have a favorable safety profile as observed across other type 2-driven immunological disorders.

The safety data available to date, in conjunction with the risk monitoring and mitigation strategies in the study protocol, and the clinical benefit of dupilumab demonstrated in multiple type 2 indications (AD, asthma, CRSwNP) and case reports and series in PN so far, support a favorable benefit-risk profile for dupilumab.

A risk-benefit statement with respect to the overall development program is provided in the investigator's brochure (IB).

3 OBJECTIVES AND ENDPOINTS

Table 1 - Objectives and endpoints

Objectives	Endpoints
**Primary**	
• To demonstrate the efficacy of dupilumab on itch response in patients with PN, inadequately controlled on topical prescription therapies or when those therapies are not advisable.	• Proportion of participants with improvement (reduction) in worst-itch numeric rating scale (WI-NRS) by ≥4 from baseline to Week 24.
**Secondary**	
• To demonstrate the efficacy of dupilumab on additional itch endpoints in patients with PN, inadequately controlled on topical prescription therapies or when those therapies are not advisable.	• Time to onset of effect on pruritus as measured by proportion of participants with an improvement (reduction) in WI-NRS by ≥4 from baseline during the 24-week treatment period.<br>• Change from baseline in WI-NRS at Week 24.<br>• Change from baseline in WI-NRS at Week 12.<br>• Percent change from baseline in WI-NRS at Week 24.<br>• Percent change from baseline in WI-NRS at Week 12.<br>• Percent change from baseline in WI-NRS at Week 4.<br>• Percent change from baseline in WI-NRS at Week 2.<br>• Percent change from baseline in WI-NRS over time until Week 24.<br>• Proportion of participants with improvement (reduction) in WI-NRS ≥4 from baseline to Week 12.<br>• Proportion of participants with WI-NRS reduction ≥4 at Week 4.<br>• Proportion of participants with WI-NRS reduction ≥4 over time until Week 24.<br>• Onset of action in change from baseline in WI-NRS (first p <0.05 difference from placebo in the daily WI NRS that remains significant at subsequent measurements) until Week 12.
• To demonstrate efficacy of dupilumab on skin lesions of PN.	• Proportion of participants with Investigator's Global Assessment 0 or 1 score for PN-Stage (IGA PN-S) at Week 24 [Key secondary endpoint].<br>• Proportion of participants with IGA PN-S 0 or 1 score at Week 12.<br>• Proportion of participants with IGA PN-S 0 or 1 score at Week 8.<br>• Proportion of participants with IGA PN-S 0 or 1 score at Week 4.<br>• Change from baseline in IGA PN-S score at Week 24.<br>• Change from baseline in IGA PN-S score at Week 12.<br>• Change from baseline in IGA PN-S score at Week 8.<br>• Change from baseline in IGA PN-S score at Week 4.<br>• Proportion of participants with Investigator's Global Assessment 0 or 1 score for PN-Activity (IGA PN-A) at Week 24.<br>• Proportion of participants with IGA PN-A 0 or 1 score at Week 12.

Objectives	Endpoints
	• Proportion of participants with IGA PN-A 0 or 1 score at Week 8.<br>• Proportion of participants with IGA PN-A 0 or 1 score at Week 4.
• To demonstrate the improvement in health-related quality of life (HRQoL).	• Change from baseline in HRQoL, as measured by Dermatology Life Quality Index (DLQI) to Week 24.<br>• Change from baseline in HRQoL, as measured by DLQI to Week 12.
• To evaluate safety outcome measures.	• Percentage of participants experiencing treatment-emergent adverse events (TEAEs) or serious adverse events (SAEs) from baseline through Week 24.
• To evaluate immunogenicity of dupilumab.	• Incidence of treatment-emergent antidrug antibodies (ADA) against dupilumab over time.
**Tertiary/exploratory**	
• To demonstrate a reduction in the use of rescue medication and systemic immunosuppressant	• Use of high potency or superpotent TCS rescue medication through Week 24<br>• Use of systemic immunosuppressant through Week 24, constituting treatment failure
• To evaluate exploratory outcome measures	• Change from baseline in Hospital Anxiety and Depression Scale (HADS) total score to Week 24.<br>• Change from baseline in EQ5D-5L to Week 24.<br>• Change from baseline in Pain numeric rating scale (NRS) to Week 4, Week 8, Week 12, and Week 24, respectively.<br>• Change from baseline in Sleep NRS to Week 4, Week 8, Week 12, and Week 24, respectively.<br>• Missed school/work days through Week 24.<br>• Incidence of skin-infection TEAEs (excluding herpetic infections) through Week 24.
• To evaluate efficacy of dupilumab on skin lesions using a modified PAS 5-item questionnaire	• Proportion of patients who achieve ≥75% healed lesions from PAS at Week 4, Week 8, Week 12, and Week 24, respectively.<br>• Change from baseline in exact number of lesions in representative area (as determined from PAS) at Week 4, Week 8, Week 12, and Week 24, respectively.
• To evaluate efficacy of dupilumab on other PN endpoints	• Change from baseline in Patient Global Impression of Severity (PGIS) of PN to Week 4, Week 8, Week 12, and Week 24, respectively.<br>• Patient Global Impression of Change (PGIC) of PN at Week 4, Week 8, Week 12, and Week 24, respectively.
PK	
• To evaluate pharmacokinetic (PK) and pharmacodynamic (PD) outcome measures	• Serum functional dupilumab concentrations and PK profile.<br>• Pharmacodynamic response for selected biomarkers (total IgE).

3.1 APPROPRIATENESS OF MEASUREMENTS

The study endpoints will include both assessments of itch and assessments of the PN lesions. As discussed in Section 2.2, the severe itch observed in this disease, the fact that lesions are typically observed in areas that are within reach for scratching, and the importance of itch-scratch cycles in the pathophysiology of the disease support itch as a reasonable primary endpoint. This choice is in line with the study design publicly available for other drug classes such as NK1-antagonists (20). Amongst standard measurement instruments for evaluation of chronic pruritus, NRS assessing worst itch in the past 24 hours features high reliability and concurrent validity and is a popular choice for all patients due to its simple format (21, 22, 23, 24). The proposed WI-NRS is currently being used in Phase 3 trial assessing serlopitant efficacy in PN (20). A similar pruritus NRS (peak pruritus) has been validated for AD. Additional validation work covering PN as additional indication will be conducted in the context of the current study. The primary assessment will be a responder analysis based on an at least 4 points improvement of the WI-NRS at Week 24. The 4-point improvement was similar to the assessment widely used in AD studies, and similar to the value proposed for assessment of clinical improvement in the WI-NRS as measures of response in the Phase 2 trial assessing serlopitant efficacy and safety in PN, and used in the Phase 3 trial (20). The timing of the primary assessment, ie, at Week 24, was based on the results of the primary analysis of EFC16460, showing that the effect of dupilumab over time showed continuous improvement after Week 12 across all endpoints, with a similar time course of improvement in both itch and lesion endpoints through at least Week 24. Since improvement of itch and lesions may occur prior to Week 24, responder analyses assessments will be performed at earlier time points as well, starting at Week 2 for itch, and Week 4 for lesions.

As a key secondary endpoint, PN lesions will be assessed by an IGA PN-Stage, which is a scale that measures the approximate number of nodules. The estimated number of lesions correlates well with increasing DLQI scores (ie, worsened quality of life), and demonstrates very good inter-rater reliability and test-retest reliability (25). The IGA scale is widely used in several dermatological indications (eg, as Physician Global Assessment [PGA] in psoriasis). A modified PAS will be used as an exploratory measure. Both instruments are PN-specific and have been developed by experts in the field. These are the only two disease-specific instruments currently available for the lesion assessment. The validation of both instruments for use in PN will be conducted in the context of the Phase 3 trials.

Secondary endpoints will also include HRQoL assessments using the skin-disease specific DLQI in recognition of the significant impacts on quality of life experienced by patients with PN. Even though not validated in PN specifically, the DLQI is well established and widely used to assess HRQoL in patients with skin conditions in clinical trials (26).

4 STUDY DESIGN

4.1 OVERALL DESIGN

This study is a multi-center, 24-week treatment, parallel, double-blind, randomized, placebo-controlled study to evaluate the use of dupilumab in patients with PN inadequately controlled on topical prescription therapies or when those therapies are not advisable.

The study will assess the effect of dupilumab on itch improvement as well as its effect on PN lesions, on patients' health-related quality of life, anxiety and depression, sleep quality and skin pain, and overall health status.

Participants who satisfy the inclusion and exclusion criteria will be randomized (1:1) to one of the following IMP treatment groups:

- Dupilumab 300 mg
- Matched placebo

Duration of study period (per participant)

- Screening period (2-4 weeks)
- Randomized IMP intervention period (24 weeks)
- Follow-up period (12 weeks)

4.2 SCIENTIFIC RATIONALE FOR STUDY DESIGN

This study is a multi-center, 24-week treatment, double-blind, randomized, placebo-controlled study to evaluate the use of dupilumab in patients with PN inadequately controlled on topical prescription therapies or when those therapies are not advisable. Based on mechanistic data (see Section 2), type 2 inflammation may play a principal role in the pathogenesis of PN in all patients.

The double-blind design intends to mitigate potential bias and placebo control is necessary to assess the net effect of active dupilumab treatment.

The patient population to be studied in this trial is intended to include patients with underlying systemic medical conditions known to be associated with PN. Given that better control of severe manifestations of underlying systemic illnesses can confound the primary itch endpoint, we will enroll 40% to 60% of non-atopic PN patients that have medically well-controlled systemic conditions. Since the literature suggests that approximately 20%-60% of PN patients have a history of atopy (defined as having a medical history of AD, allergic rhinitis/rhinoconjunctivits, asthma, or food allergy), our study design will require that the remaining PN patients have a history or a current diagnosis of atopy. Because active AD can confound the primary endpoint, we will exclude moderate-to-severe AD patients and will institute a cap of up to 10% of the atopic patient subgroup having active mild AD. It is the goal of this design to mirror the overall PN patient population consistent with real-world estimates of overall prevalence while trying to minimize potential confounders on the primary endpoint.

The primary endpoint, WI-NRS, is a validated measurement of the highest PN burden, ie, extreme itching.

The Sponsor considers that a 24-week treatment period is an appropriate duration to observe dupilumab's effect in PN to ensure an adequate evaluation of PN lesions and itch as the effect is expected to continue to increase over time and treatment effects will be most apparent by Week 24 similar to that observed in the completed primary analysis of the EFC16460 study of dupilumab in PN. The duration of the 12-week follow-up period is based on the time expected for drug concentration to reach zero (below the lower limit of quantification) in most patients after the last dose of dupilumab. This follow-up period will allow assessment of the need for chronic treatment with dupilumab for this disease by an assessment of return of PN symptoms post-treatment.

The study will not include adolescents because PN is rare in adolescent patients.

4.3 JUSTIFICATION FOR DOSE

The selected dosing regimen is dupilumab 300 mg Q2W with a loading dose of 600 mg.

This dose regimen is expected to achieve serum concentrations that saturate the target-mediated clearance pathway rapidly with the loading dose and maintain saturation thereafter. The PK of dupilumab is consistent across populations of patients with AD, asthma, CRSwNP, and EoE, as well as healthy volunteers. Furthermore, the sources of variability of dupilumab PK identified in each population and the magnitude of the covariate effects indicate that body weight is the most influential factor, whereas other covariates identified as being statistically significant have no meaningful impact on dupilumab PK. The immunogenicity of dupilumab is also comparable in these populations (see IB).

Dosing regimens of 300 mg Q2W after a loading dose of 600 mg were used in the PN case studies, which resulted in marked improvement in itch within three months and subsequently a notable improvement of the skin lesions (see Section 2).

The 300 mg Q2W dose, with loading dose, is the approved dose for AD and is an approved dose for asthma in the US and the EU.

Therefore, based on the totality of the data, dupilumab 300 mg Q2W (with loading dose) regimen is adequate for patients with PN and a further dose ranging study is not necessary to select a Phase 3 dose regimen.

4.4 END OF STUDY DEFINITION

A participant is considered to have completed the study if he/she has completed all phases of the study including the last visit.

The end of the study is defined as the date of the last visit of the last participant in the study.

5 STUDY POPULATION

Prospective approval of protocol deviations to recruitment and enrollment criteria, also known as protocol waivers or exemptions, is not permitted.

5.1 INCLUSION CRITERIA

Participants are eligible to be included in the study only if all of the following criteria apply:

Age

I 01. Participants must be 18 to 80 years of age, at the time of signing the informed consent.

Type of participant and disease characteristics

Patients with a clinical diagnosis of PN, as defined by all of the following:

I 02. Diagnosed by a dermatologist for at least 3 months before the Screening visit.

I 03. On the WI-NRS ranging from 0 to 10, patients must have an average worst itch score of ≥7 in the 7 days prior to Day 1.

NOTE: Baseline pruritus NRS average score for maximum itch intensity will be determined based on the average of daily NRS scores for maximum intensity (the daily score ranges from 0 to 10) during the 7 days immediately preceding randomization. A minimum of 4 daily scores out of the 7 days is required to calculate the baseline average score. For patients who do not have at least 4 daily scores reported during the 7 days immediately preceding the planned randomization date, randomization should be postponed until this requirement is met, but without exceeding the 28-day maximum duration of the screening period.

I 04. Patients must have a minimum of 20 PN lesions in total on both legs, and/or both arms and/or trunk, at Screening visit and on Day 1.

NOTE: Patients need to have bilaterally symmetrical lesions on the extremities. The presence of lesions on at least 2 body surface areas is required.

I 05. History of failing a 2-week course of medium-to-superpotent TCS or when TCS are not medically advisable.

NOTE: Failure is defined as patients who are unable to achieve and/or maintain remission and low disease activity (similar to IGA PN-S score of ≤2 [≤19 nodules]) despite treatment with a daily regimen of medium-to-superpotent TCS (±TCI as appropriate), applied for at least 14 days, or for the maximum duration recommended by the product prescribing information, whichever is shorter.

I 06. Have applied a stable dose of topical emollient (moisturizer) once or twice daily for at least 5 out of the 7 consecutive days immediately before Day 1 (NOTE: See exclusion criterion E 20 for limitations regarding emollients; see background treatment with topical emollient in Section 6.1.1).

I 07. Participants must be willing and able to complete a daily symptom eDiary for the duration of the study.

Sex

I 08. Male or Female

Contraceptive use by women should be consistent with local regulations regarding the methods of contraception for those participating in clinical studies.

a) Female participants

- A female participant is eligible to participate if she is not pregnant or breastfeeding, and at least one of the following conditions applies:
  - Is not a WOCBP.

  OR

  - Is a WOCBP and agrees to use a contraceptive method as described in Appendix 4 (Section 10.4) during the study (at a minimum until 12 weeks after the last dose of study intervention).
  - A WOCBP must have a negative highly sensitive (Section 10.2) pregnancy test (urine or serum as required by local regulations) on Day 1 before the first dose of study intervention.
  - If a urine test cannot be confirmed as negative (eg, an ambiguous result), a serum pregnancy test is required. In such cases, the participant must be excluded from participation if the serum pregnancy result is positive.
- Additional details can be found in Appendix 4 (Section 10.4).
- The investigator is responsible for review of medical history, menstrual history, and recent sexual activity to decrease the risk for inclusion of a woman with an early undetected pregnancy.

Informed Consent

I 09. Capable of giving signed informed consent as described in Appendix 1 (Section 10.1) of the protocol which includes compliance with the requirements and restrictions listed in the informed consent form (ICF) and in this protocol. In countries where legal age of majority is above 18 years, a specific ICF must also be signed by the participant's legally authorized representative.

5.2 EXCLUSION CRITERIA

Participants are excluded from the study if any of the following criteria apply:

Medical conditions

E 01. Presence of skin morbidities other than PN and mild AD that may interfere with the assessment of the study outcomes. Conditions such as, but not limited to, the following: scabies, insect bite, lichen simplex chronicus, psoriasis, acne, folliculitis, habitual picking, lymphomatoid papulosis, chronic actinic dermatitis, dermatitis herpetiformis, sporotrichosis, bullous disease.

NOTE: patients with mild active AD will represent up to 10% of the atopic PN study population.

E 02. PN secondary to medications (eg, opioids, angiotensin converting enzyme [ACE] inhibitors).

E 03. PN secondary to medical conditions such as neuropathy or psychiatric disease (eg, notalgia paresthetica, brachioradial pruritus, neurotic excoriations, obsessive compulsive disorder, delusions of parasitosis, etc).

E 04. Patients with a documented AD severity moderate to severe within 6 months before the screening visit, or documented diagnosis of moderate to severe AD from screening visit to randomization visit (eg, IGA AD of 3 or 4, eczema area and severity index [EASI] ≥16, scoring atopic dermatitis [SCORAD] ≥25).

E 05. Severe concomitant illness(es) under poor control that, in the investigator's judgment, would adversely affect the patient's participation in the study. Examples include, but are not limited to patients with life expectancy shorter than 1 year, patients with uncontrolled diabetes (hemoglobin A1c ≥9% according to the laboratory results within 3 months before screening visit), patients with cardiovascular conditions (eg, Class III or IV heart failure according to the New York Heart Association classification), hepato-biliary conditions (eg, Child-Pugh Class B or C), neurological conditions (eg, demyelinating diseases), active major autoimmune diseases (eg, lupus, inflammatory bowel disease, rheumatoid arthritis, etc), other severe endocrinological, gastrointestinal, metabolic, pulmonary, or lymphatic diseases. The specific justification for patients excluded under this criterion will be noted in study documents (chart notes, eCRF, etc).

E 06. Severe renal conditions (eg, patients with uremia and/or on dialysis).

E 07. Participants with uncontrolled thyroid disease.

E 08. Active TB or non-tuberculous mycobacterial infection, or a history of incompletely treated TB will be excluded from the study unless it is well documented by a specialist that the participant has been adequately treated and can now start treatment with dupilumab in the medical judgment of the investigator and/or infectious disease specialist. Tuberculosis testing will be performed on a country-by-country basis, according to local guidelines if required by regulatory authorities or ethics boards.

E 09. Diagnosed active endoparasitic infections; suspected or high risk of endoparasitic infection, unless clinical and (if necessary) laboratory assessment have ruled out active infection before randomization.

E 10. Active chronic or acute infection (except HIV infection) requiring treatment with systemic antibiotics, antivirals, antiprotozoals, or antifungals within 2 weeks before screening visit or during the screening period.

E 11. Known or suspected immunodeficiency, including history of invasive opportunistic infections (eg, TB, histoplasmosis, listeriosis, coccidioidomycosis, pneumocystosis, aspergillosis) despite infection resolution, or otherwise recurrent infections of abnormal frequency or prolonged duration suggesting an immune-compromised status, as judged by the investigator.

E 12. Active malignancy or history of malignancy within 5 years before the baseline visit, except completely treated in situ carcinoma of the cervix, completely treated and resolved non-metastatic squamous or basal cell carcinoma of the skin.

E 13. History of systemic hypersensitivity or anaphylaxis to any biologic therapy, including any excipients.

E 14. Any other medical or psychological condition including relevant laboratory abnormalities at screening that, in the opinion of the investigator, suggest a new and/or insufficiently understood disease, may present an unreasonable risk to the study patient as a result of his/her participation in this clinical trial, may make patient’s participation unreliable, or may interfere with study assessments. The specific justification for patients excluded under this criterion will be noted in study documents (chart notes, eCRF, etc).

E 15. History of substance and/or alcohol abuse.

E 16. Planned major surgical procedure during the patient’s participation in this study.

Prior/concomitant therapy

E 17. Exposure to another systemic or topical investigative drug (monoclonal antibodies as well as small molecules) within a certain time period prior to Visit 1 (screening), as follows: an interval of less than 6 months or <5 PK half-lives for investigative monoclonal antibodies, whichever is longer, and an interval of less than 30 days or <5 PK half-lives, whichever is longer, for investigative small molecules.

E 18. Having used any of the following treatments within 4 weeks before the screening visit

- Systemic immunosuppressive/immunomodulating drugs (eg, systemic corticosteroids, cyclosporine, mycophenolate-mofetil, interferon gamma, Janus kinase inhibitors, azathioprine, methotrexate, hydroxychloroquine, dapsone, sulfasalazine, colchine, etc),
- Intralesional corticosteroid injections and cryotherapy,
- Phototherapy, including tanning beds,

- Naltrexone or other opioid antagonist,
- Gabapentin, pregabalin, and thalidomide.

Or starting to use the following treatments or changed the dose of the following treatments in 3 months before the screening visit or expected the dose of the following treatments will be changed throughout the study:

- Paroxetine, fluvoxamine, or other selective serotonin reuptake inhibitors (SSRIs),
- Serotonin and norepinephrine reuptake inhibitors (SNRIs),
- Amitriptyline or other tricyclic or tetracyclic antidepressants.

E 19. Previous treatment with biologic medicines within the following timeframe:

- Any cell-depleting agents including but not limited to rituximab: within 6 months before the screening visit,
- Omalizumab: within 5 months before screening visit,
- Other immunomodulatory biologics: within 5 half-lives (if known) or 16 weeks before the screening visit, whichever is longer.

E 20. Initiation of treatment with prescription moisturizers or moisturizers containing additives such as ceramide, hyaluronic acid, urea, menthol, polidocanol, or filaggrin degradation products during the screening period (patients may continue using stable doses of such moisturizers if initiated before the Screening visit).

E 21. Initiation of treatment with TCS/TCI (any potency) during the screening period or treatment with high potency or superpotent TCS/TCI during the screening period.

E 22. For participants who were on a stable regimen of TCS/TCI (maintain same medicine, same dose from 2 weeks prior to screening visit) at the screening visit:

- Application of TCS/TCI on fewer than 6 days during the 7 days immediately preceding randomization,
- Application of TCS/TCI of incorrect potency within 7 days before Day 1 according to the requirements of Section 6.1.1, ie, low potency if on low potency at screening visit and medium potency if on medium or higher potency at screening visit.

E 23. Treatment with a live (attenuated) vaccine within 4 weeks before the screening visit.

NOTE: For patients who have vaccination with live, attenuated vaccines planned during the course of the study (based on national vaccination schedule/local guidelines), it will be determined, after consultation with a physician, whether the administration of vaccine can be postponed until after the end of study, or preponed to before the start of the study, without compromising the health of the patient:

- Patient for whom administration of live (attenuated) vaccine can be safely postponed would be eligible to enroll into the study,

- Patients who have their vaccination preponed can enroll in the study only after a gap of 4 weeks following administration of the vaccine.

E 24. Planned or anticipated use of any prohibited medications and procedures during screening and study treatment period.

Prior/concurrent clinical study experience

E 25. Participation in prior dupilumab clinical study; treated in the past with dupilumab; prior use of biologics for PN.

Diagnostic assessments

E 26. For participants without history of HIV infection before screening visit, positive HIV serology at screening.

For participants with history of HIV infection with CD4+ counts ≤300 cells/µL and/or detectable HIV viral load at screening.

E 27. Participants with any of the following result at screening:

- Positive (or indeterminate) HBs Ag or,
- Positive total HBc Ab confirmed by positive HBV DNA or,
- Positive HCV Ab confirmed by positive HCV RNA.

Other exclusions

E 28. Individuals accommodated in an institution because of regulatory or legal order; prisoners or subjects who are legally institutionalized.

E 29. Any country-related specific regulation that would prevent the subject from entering the study - see Section 10.10 (country-specific requirements).

E 30. Participant not suitable for participation, whatever the reason, as judged by the Investigator, including medical or clinical conditions, or participants potentially at risk of noncompliance to study procedures.

E 31. Participants are dependent on the Sponsor or Investigator (in conjunction with Section 1.61 of the International Council for Harmonisation (ICH) good clinical practice (GCP) Ordinance E6).

E 32. Participants are employees of the clinical study site or other individuals directly involved in the conduct of the study, or immediate family members of such individuals.

E 33. Any specific situation during study implementation/course that may raise ethics considerations.

E 34. Sensitivity to any of the study interventions, or components thereof, or drug or other allergy that, in the opinion of the Investigator, contraindicates participation in the study.

5.3 LIFESTYLE CONSIDERATIONS

Not applicable.

5.4 SCREEN FAILURES

Screen failures are defined as participants who consent to participate in the clinical study but are not subsequently randomly assigned to study intervention/entered in the study. A minimal set of screen failure information is required to ensure transparent reporting of screen failure participants to meet the Consolidated Standards of Reporting Trials (CONSORT) publishing requirements and to respond to queries from regulatory authorities. Minimal information includes demography, screen failure reasons, eligibility criteria, and any SAE.

In the case of technical malfunction of equipment, the patient may be rescreened. Patients who fail the exclusion criteria may be rescreened once during the open screening period of the study. Rescreened participants will be assigned a new participant number versus the one received for the initial screening.

There is no requirement for a waiting period between the screen-failure date and the rescreening date. The interactive response technology (IRT) report will flag rescreened patients. Patients that are rescreened must sign a new consent form and all Visit 1 procedures must be repeated.

If certain dynamic laboratory tests do not meet the eligibility criteria at screening, these laboratory assessments may be repeated, at the discretion of the Investigator, if it is judged to be likely to return to acceptable range for study inclusion within the screening visit window prior to Day 1. There is no need to screen fail such participants if the test finally meets the eligibility criteria.

6 STUDY INTERVENTION

Study intervention is defined as any investigational intervention(s), marketed product(s), placebo, or medical device(s) intended to be administered to a study participant according to the study protocol.

6.1 STUDY INTERVENTION(S) ADMINISTERED

Table 2 - Overview of study interventions administered

**ARM name**	Dupilumab	Placebo
**Intervention name**	Dupilumab 300 mg	Placebo matching dupilumab 300 mg
**Type**	Biological/Vaccine	Other
**Dose formulation**	A 150 mg/mL dupilumab solution in a pre-filled syringe to deliver 300 mg in 2 mL	Identical formulation to the active 300 mg formulation without dupilumab, in a pre-filled syringe to deliver placebo in 2 mL
**Unit dose strength(s)**	300 mg	0 mg (placebo)
**Dosage level(s)**	300 mg every 14 ±3 days after an initial loading dose of 600 mg	0 mg every 14 ±3 days with a loading dose of 0 mg
**Route of administration**	Subcutaneous[a]	Subcutaneous[a]
**IMP and NIMP**	IMP	IMP
**Packaging and labeling**	One glass pre-filled syringe packed in a patient kit box. Both the glass pre-filled syringe and the box will be labeled as required per country requirement.	One glass pre-filled syringe packed in a patient kit box. Both glass pre-filled the syringe and the box will be labeled as required per country requirement

a Subcutaneous injection sites should alternate between the upper thighs, 4 quadrants of the abdomen or the upper arms, so that the same site is not injected twice during consecutive administrations. Injection in the upper arms can only be done by a trained person (parent/legally authorized representative/caregiver trained by Investigator or Delegate) or health care professional but not the participant themselves.
IMP: investigational medicinal product; NIMP: noninvestigational medicinal product.

The Investigator or delegate will train the patient (or caregiver) how to prepare and inject IMP at Visit 2. He/she will inject the first of the two injections. The participant (or caregiver) will perform the second injection under the supervision of the Investigator or delegate. Patient should also be trained by the site staff to recognize potential signs and symptoms of hypersensitivity reaction in order to self-monitor at home for at least 30 minutes (or longer per country specific or local site-specific requirements) following injection. In case of hypersensitivity symptoms the patient should contact healthcare provider/emergency. The training must be documented in the participant's study file.

When the participant has a study visit, the IMP will be administered following clinical procedures and blood collection. Patients should be monitored for at least 30 minutes. The monitoring period may be extended as per country specific or local site-specific requirements.

Between the protocol-scheduled on-site visits, participants are allowed to self-inject IMP at home. Participants who prefer to have a healthcare professional administer the IMP may choose to have injections administered at home by a nurse or at the study site.

Between the protocol-scheduled on-site visits, interim visits may be required for IMP dispensing. As an alternative to these visits, dupilumab 300 mg or matching placebo may be supplied from the site to the participant via a Sponsor-approved courier company where allowed by local regulations and approved by the participant.

6.1.1 Noninvestigational medicinal products

Starting from the screening visit, participants will be instructed to use their daily moisturizer, if it is not containing any compound with known anti-itch effect (such as menthol, polidocanol, pramoxine, lidocaine, prilocaine, capsaicin, naltrexone, N-palmitoylethanolamine, etc). It is not authorized changing emollients or moisturizers or applying products for itching relief during the course of the study.

Participants will be required to apply moisturizers (emollients) once or twice daily for at least 5 out of the 7 consecutive days immediately before Day 1 and continue until Week 36. All types of moisturizers are permitted, but patients may not initiate new treatment with prescription moisturizers or over-the-counter moisturizers containing additives during the screening period or during the intervention period. Patients may continue using stable doses of such moisturizers if initiated before the screening visit.

If participants are on a stable regimen of low to medium potency TCS or TCI at the screening visit, they can continue their topical steroid application once daily without tapering from Screening to Week 24. If specific lesions resolve, the participant can stop applying steroids to those sites but are permitted to continue applying to persistent lesions. If participants are on stable regimens of high potency or superpotent steroids, participants should decrease potency to medium potency TCS and continue to apply daily from screening to Week 24. Stable regimen for TCS is maintaining the same medicine (low to medium potency TCS), and maintaining the same frequency of treatment (once or twice daily) used from 2 weeks prior to screening. Stable regimen for TCI is maintaining the same medicine of TCI and the treatment frequency (once or twice daily) used from 2 weeks prior to screening. If participant's prior regimen was applying once daily, the participant will maintain daily application during study and for participants who had twice daily prior to screening, participation will maintain twice daily regimen during study. If specific lesions resolve, the participant can stop applying steroids to those sites but are permitted to continue applying to persistent lesions. Occlusion is not allowed from Screening to Week 24.

It is recommended that patients use triamcinolone acetonide 0.1% cream or fluocinolone acetonide 0.025% ointment for medium potency, and hydrocortisone 1% cream for low potency. If rescue with TCS is needed, it is recommended that patients use either betamethasone dipropionate 0.05% optimized ointment for high potency TCS or clobetasol propionate 0.05% cream for super high potency TCS. If patients have tolerance issues with any of these steroids or if they are not commercially available in some countries, they may substitute with products of the same potency from the list provided by the Sponsor.

On areas treated with TCS, moisturizers must be applied once daily only at the time when TCS is not applied (ie, do not use moisturizers and TCS on the same areas at the same time during the day). For example, if TCS are applied in the evening, moisturizers will not be used in the evening on areas treated with TCS, but will be applied to those areas in the morning. On areas not treated with TCS, moisturizers will be applied twice daily - morning and evening.

Rescue therapy is described in Section 6.5.1.

6.2 PREPARATION/HANDLING/STORAGE/ACCOUNTABILITY

6.2.1 Storage and handling

1. The Investigator or designee must confirm appropriate temperature conditions have been maintained during transit for all study intervention received and any discrepancies are reported and resolved before use of the study intervention.
2. Only participants enrolled in the study may receive study intervention and only authorized site staff may supply or administer study intervention. All study intervention must be stored in a secure, environmentally controlled, and monitored (manual or automated) area in accordance with the labeled storage conditions with access limited to the Investigator and authorized site staff.
3. The Investigator, institution, or the head of the medical institution (where applicable) is responsible for study intervention accountability, reconciliation, and record maintenance (ie, receipt, reconciliation, and final disposition records).

6.2.2 Responsibilities

Any quality issue noticed with the receipt or use of an IMP (deficiency in condition, appearance, pertaining documentation, labeling, expiration date, etc) must be promptly notified to the Sponsor. Some deficiencies may be recorded through a complaint procedure (see Section 8.3.6).

A potential defect in the quality of IMP may be subject to initiation of a recall procedure by the Sponsor. In this case, the Investigator will be responsible for promptly addressing any request made by the Sponsor, in order to recall the IMP and eliminate potential hazards.

Under no circumstances will the Investigator supply IMP to a third party (except for direct-to-patient [DTP] shipment, for which a courier company has been approved by the Sponsor), allow the IMP to be used other than as directed by this clinical trial protocol, or dispose of IMP in any other manner.

6.3 MEASURES TO MINIMIZE BIAS: RANDOMIZATION AND BLINDING

All participants will be centrally assigned to randomized study intervention using an IRT. Before the study is initiated, the telephone number and call-in directions for the IVRS and/or the log in information & directions for the IWRS will be provided to each site.

At screening (Visit 1), the Investigator or designee will contact the IRT system to receive the participant number. If a patient who had previously failed screening is approached for re-screening, a new ICF must be signed. In such case, a new patient number will be assigned by IRT.

Methods of assigning patients to treatment group

The randomized intervention kit number list is generated centrally by Sanofi and IMPs are packaged in accordance with this list. The randomization and intervention allocation are performed centrally by an IRT. The IRT generates the participant randomization list and allocates the intervention number and the corresponding intervention kits to the participants according to it.

Participants will be randomized in 1:1 ratio to treatment arms described in Table 2.

Randomization will be stratified by the following factors:

- Documented history of atopy (atopic or non-atopic)
  - Atopic: Patients with a physician-documented history of atopic comorbidities defined as AD, allergic rhinitis/rhinoconjunctivits, asthma, or food allergy, or a current diagnosis of at least one of these atopic comorbidities, per investigator judgement. NOTE: enrollment of patients with a current diagnosis of active mild AD will be capped at up to 10% of the participants in this subgroup
  - Non-atopic: Patients without a physician-documented history of atopic comorbidities defined as AD, allergic rhinitis/rhinoconjunctivitis, asthma or food allergy, and without a current diagnosis of at least one of such atopic comorbidities, per investigator judgement
- Stable use of TCS/TCI (yes or no)
- Country/territory code

A randomized participant is defined as a participant who has been allocated to a randomized intervention regardless whether the intervention kit was used or not (ie, participant registered by the IRT).

A participant cannot be randomized more than once in the study.

Study intervention will be dispensed at the study visits summarized in SoA (see Section 1.3). Returned study intervention should not be re-dispensed to the participants.

Methods of blinding

- Dupilumab 300 mg and placebo matching dupilumab 300 mg will be provided in identically matched 2 mL pre-filled syringes that are visually indistinguishable. Syringes and box will be labeled with a treatment kit number.
- The unblinding plan is described in Section 9.8.

Randomization code breaking during the study

The IRT will be programmed with blind-breaking instructions. In case of an emergency, the Investigator has the sole responsibility for determining if unblinding of a participant's treatment assignment is warranted (eg, in case of available antidote). Participant safety must always be the first consideration in making such a determination. If the Investigator decides that unblinding is warranted, the Investigator should make every effort to contact the Sponsor prior to unblinding a participant's treatment assignment unless this could delay emergency treatment of the participant. If a participant's treatment assignment is unblinded, the Sponsor must be notified within 24 hours after breaking the blind. The date and reason that the blind was broken must be recorded in the source documentation and case report form (CRF), as applicable.

6.4 STUDY INTERVENTION COMPLIANCE

Investigator or his/her delegate must ensure that IMP will be administered to each participant according to the labeling instructions.

IMP accountability:

- Intervention units are returned by the participant at each visit. In case of DTP process, the intervention units can be returned by the carrier (if defined in the contract).
- The Investigator counts the number of remaining kit/pre-filled syringe, and fills in the IMP accountability and inventory forms.
- The Investigator or his/her delegate records the dosing information on the appropriate page(s) of the eCRF.
- The monitor in charge of the study then checks the eCRF data by comparing them with the IMP which he/she has retrieved and source documents.

Participant compliance with study intervention will be assessed at each visit. Compliance will be assessed by counting returned kit/pre-filled syringe. Deviation(s) from the prescribed dosage regimen should be recorded in the eCRF.

6.5 CONCOMITANT THERAPY

Any medication or vaccine (including over-the-counter or prescription medicines, vitamins, and/or herbal supplements) that the participant is receiving at the time of enrollment or receives during the study must be recorded along with:

- Reason for use.
- Dates of administration including start and end dates.
- Dosage information including dose and frequency.

The concomitant use of non-sedating antihistamine administration is allowed during the study except for treatment of AD or PN, but dose change of non-sedating antihistamine is not allowed both from Week 11 to Week 12 and from Week 23 to Week 24.

The concomitant use of the following therapies is prohibited during the entire study. Study treatment will need to be discontinued in participants receiving these treatments:

- Systemic immunosuppressive/immunomodulating drugs (eg, systemic corticosteroids, cyclosporine, mycophenolate-mofetil, interferon gamma, Janus kinase inhibitors, azathioprine, methotrexate, hydroxychloroquine, dapsone, sulfasalazine, colchine, etc).
- Other monoclonal antibodies (which are biological modifiers).
- Phototherapy, including tanning beds.
- Naltrexone or other opioid antagonist.
- Gabapentin, pregabalin, and thalidomide.

The concomitant use of the following therapies is prohibited except if the dose has been stable for at least 3 months prior to screening, but study treatment will not need to be discontinued in participants receiving the treatments listed below. The dose should also remain stable (can be reduced or discontinued if medically indicated), but should not be initiated or increased throughout the study.

- Paroxetine, fluvoxamine, or other SSRIs.
- SNRIs.
- Amitriptyline or other tricyclic or tetracyclic antidepressants.

The concomitant use of the following therapies is also prohibited during the entire study, but study treatment will not need to be discontinued in participants receiving the treatments listed below.

- Intralesional corticosteroid injections and cryotherapy.
- Sedating antihistamine.
- Non-sedating antihistamine used specifically for the treatment of itch secondary to AD or PN.

6.5.1 Rescue medicine

The following rescue medications may be used:

- Dermatological preparations of high potency or superpotent TCS and TCI.

If medically necessary (ie, to control intolerable PN symptoms), rescue treatment for PN may be provided to study patients at the discretion of the Investigator.

Although the use of rescue medications is allowed at any time during the study, the use of rescue medications should be delayed, if possible, for at least 14 days following the initiation of the investigational treatment. The date and time of rescue medication administration as well as the name and dosage regimen of the rescue medication must be recorded in the eCRF.

For the purpose of the efficacy responder analysis, a pre-specified algorithm will be used to classify rescue (details in the SAP). In addition, a blinded review of all post-baseline medications to adjudicate rescue treatment, based on medical judgment, will be performed to adjudicate rescue. Patients who receive rescue treatment as per this adjudication during the study will be considered treatment failures.

6.6 DOSE MODIFICATION

No change in IMP dose is allowed.

6.7 INTERVENTION AFTER THE END OF THE STUDY

The Sponsor will not be responsible for intervention after the EOS Visit. Intervention after the EOS Visit will be at the discretion of investigator or treating physician.

7 DISCONTINUATION OF STUDY INTERVENTION AND PARTICIPANT DISCONTINUATION/WITHDRAWAL

7.1 DISCONTINUATION OF STUDY INTERVENTION

7.1.1 Definitive discontinuation

In rare instances, it may be necessary for a participant to permanently discontinue study intervention. If study intervention is permanently discontinued, the participant will remain in the study to be evaluated for safety. See the SoA (Section 1.3) for data to be collected at the time of discontinuation of study intervention.

The participants may withdraw from treatment with the IMP if he or she decides to do so, at any time and irrespective of the reason, or this may be the investigator's decision. All efforts should be made to document the reason(s) for treatment discontinuation and this should be documented in the eCRF.

Participants must be permanently withdrawn from the study treatment for the following reasons:

- At their own request or at the request of their legally authorized representative (legally authorized representative means an individual or judicial or other body authorized under applicable law to consent on behalf of a prospective participant to the patient's participation in the procedure(s) involved in the research).
- If, in the investigator's opinion, continuation in the study would be detrimental to the participant's well-being.
- At the specific request of the Sponsor.
- If they are treated with the specific prohibited medications mentioned in Section 6.5.
- If they miss more than 2 consecutive IMP doses.
- In the event of a protocol deviation, at the discretion of the investigator or the Sponsor.
- Any code broken at the requested of the investigator.
- Pregnancy.
- Anaphylactic reactions or systemic allergic reactions that are related to IMP and require treatment (see Section 10.8).
- Diagnosis of a malignancy during study, excluding carcinoma in situ of the cervix, or squamous or basal cell carcinoma of the skin.
- Any opportunistic infection or other infections whose nature or course may suggest an immunocompromised status (see Section 10.9).
- Serum ALT >3 ULN and Total Bilirubin >2 ULN (see Section 10.6).
- Serum ALT >5 ULN if baseline ALT ≤2 ULN or ALT >8 ULN if baseline ALT >2 ULN (see Section 10.6).

If a clinically significant finding is identified (including, but not limited to changes from baseline in QT interval corrected using Fridericia's formula [QTcF] after enrollment), the Investigator or qualified designee will determine if the participant can continue in the study and if any change in participant management is needed. This review of the ECG printed at the time of collection must be documented. Any new clinically relevant finding should be reported as an AE.

Any abnormal laboratory value or ECG parameter will be immediately rechecked for confirmation within a reasonable timeframe as assessed by the Investigator before making a decision of definitive discontinuation of the IMP for the concerned participant.

Handling of participants after definitive intervention discontinuation

Participants will be followed-up according to the study procedures specified in this protocol up to the scheduled date of study completion, or up to recovery or stabilization of any AE to be followed-up as specified in this protocol, whichever comes last.

Participants who discontinue the study intervention prematurely (prior to completing the 24-week treatment period) will perform, as soon as possible, the early termination visit with all assessments normally planned for the EOT Visit (Visit 6), to assure a complete clinical assessment in close temporal proximity to the premature termination of study treatment is available.

In addition, and to allow assessment of participant outcomes over the stipulated study period, patients will be asked and encouraged to complete all remaining study treatment visits, and participate in all assessments according to the visit schedule with a ±3 day window. Under exceptional circumstances when a participant cannot come to the site for a scheduled visit, a phone contact can be made. During the phone contact, at least information about AEs, concomitant medication and status of PN should be collected.

All cases of definitive intervention discontinuation must be recorded by the Investigator in the appropriate pages of the eCRF when considered as confirmed.

7.1.2 Temporary discontinuation

Temporary intervention discontinuation may be considered by the Investigator because of suspected AEs.

In addition, the following conditions will be causes for temporary discontinuation of study intervention:

- Infections or infestations that do not respond to medical treatment.
- Any laboratory abnormality that meets temporary treatment discontinuation criteria as per Section 10.6.

Temporary intervention discontinuation decided by the Investigator corresponds to more than 1 dose not administered to the participant. Following a temporary interruption or missed dose, the treatment should be reinitiated at the next scheduled administration, maintaining the planned dose. If more than 2 consecutive IMP doses have been missed, the study treatment will be permanently discontinued (see Section 7.1.1).

7.1.2.1 Rechallenge

Reinitiation of intervention with the IMP will be done under close and appropriate clinical/and or laboratory monitoring once the Investigator will have considered according to his/her best medical judgment that the responsibility of the IMP(s) in the occurrence of the concerned event was unlikely and if the selection criteria for the study are still met (refer to Section 5).

7.2 PARTICIPANT DISCONTINUATION/WITHDRAWAL FROM THE STUDY

- A participant may withdraw from the study at any time at his/her own request, or may be withdrawn at any time at the discretion of the Investigator for safety, behavioral, compliance, or administrative reasons. This is expected to be uncommon.
- At the time of discontinuing from the study, if possible, an early discontinuation visit should be conducted, as shown in the SoA (Section 1.3). See SoA for data to be collected at the time of study discontinuation and follow-up and for any further evaluations that need to be completed.
- The participant will be permanently discontinued both from the study intervention and from the study at that time.
- If the participant withdraws consent for disclosure of future information, the Sponsor may retain and continue to use any data collected before such a withdrawal of consent.
- If a participant withdraws from the study, he/she may request destruction of any samples taken and not tested, and the Investigator must document this in the site study records.

If participants no longer wish to take the IMP, they will be encouraged to remain in the study.

The Investigators should discuss with them key visits to attend. The value of all their study data collected during their continued involvement will be emphasized as important to the public health value of the study.

Participants who withdraw from the study intervention should be explicitly asked about the contribution of possible AEs to their decision, and any AE information elicited must be documented.

All study withdrawals should be recorded by the Investigator in the appropriate screens of the eCRF and in the participant's medical records. In the medical record, at least the date of the withdrawal and the reason should be documented.

In addition, a participant may withdraw his/her consent to stop participating in the study. Withdrawal of consent for intervention should be distinguished from withdrawal of consent for follow-up visits and from withdrawal of consent for non-participant contact follow-up, eg, medical record checks. The site should document any case of withdrawal of consent.

Participants who have withdrawn from the study cannot be rerandomized (treated) in the study. Their inclusion and intervention numbers must not be reused.

7.3 LOST TO FOLLOW UP

A participant will be considered lost to follow-up if he or she repeatedly fails to return for scheduled visits and is unable to be contacted by the study site.

The following actions must be taken if a participant fails to return to the clinic for a required study visit:

- The site must attempt to contact the participant and reschedule the missed visit as soon as possible and counsel the participant on the importance of maintaining the assigned visit schedule and ascertain whether or not the participant wishes to and/or should continue in the study.
- Before a participant is deemed lost to follow up, the Investigator or designee must make every effort to regain contact with the participant (where possible, 3 telephone calls and, if necessary, a certified letter to the participant's last known mailing address or local equivalent methods). These contact attempts should be documented in the participant's medical record.
- Should the participant continue to be unreachable, he/she will be considered to have withdrawn from the study.
- Discontinuation of specific sites or of the study as a whole are handled as part of Section 10.1.

8 STUDY ASSESSMENTS AND PROCEDURES

- Study procedures and their timing are summarized in the SoA (see Section 1.3). Protocol waivers or exemptions are not allowed.
- Adherence to the study design requirements, including those specified in the SoA (see Section 1.3), is essential and required for study conduct.
- All screening evaluations must be completed and reviewed to confirm that potential participants meet all eligibility criteria. The Investigator will maintain a screening log to record details of all participants screened and to confirm eligibility or record reasons for screening failure, as applicable.
- Procedures conducted as part of the participant's routine clinical management (eg, blood count) and obtained before signing of the ICF may be utilized for screening or baseline purposes provided the procedures met the protocol-specified criteria and were performed within the time frame defined in the SoA (see Section 1.3).
- Patient-Reported Outcome questionnaires including NRS should be completed by the participants before the consultation and/or clinical tests, in a quiet place. The questionnaires should be completed by the participants themselves, independently from their physician, the study nurse or any other medical personnel and without any help from friends or relatives.
- In light of the public health emergency related to Coronavirus disease 2019 (COVID-19) (or in case of any other pandemic requiring public health emergency), the continuity of clinical study conduct and oversight may require implementation of temporary or alternative mechanisms eg, phone contact, virtual visits, online meetings, use of local clinic or laboratory locations, and home visits by skilled staff. Implementation of such mechanisms may differ country by country, depending on country regulations and local business continuity plans. Additionally, no waivers to deviate from protocol enrollment criteria due to COVID-19 (or any other pandemic) will be granted. All temporary mechanisms utilized, and deviations from planned study procedures are to be documented as being related to COVID-19 (or any other pandemic) and will remain in effect only for the duration of the public health emergency.

8.1 EFFICACY ASSESSMENTS

Efficacy data will be collected via electronic devices.

The e-diary is used for daily recording of participant's answers to the WI-NRS, pain-NRS, and sleep-NRS questionnaires. This device will be dispensed at screening visit (Visit 1), including instructions for use. Participants will be instructed on the use of the device.

Recorded information will be downloaded from this device daily. At EOS Visit, the e-diary will be downloaded and returned to the site.

On regular basis, the site staff should review on vendor's website the information downloaded from participants' e-diary. They should particularly check status of the disease reviewing

WI-NRS, as well as compliance to background therapy and overall e-diary compliance. The site should follow up with the participant as appropriate.

Participants will fill in the DLQI, HADS, EQ-5D-5L, PGIC, PGIS, and missed school/work days questionnaires during their site visit on a tablet that will be provided to the site. This device is kept at the site during the study.

8.1.1 Worst-itch numeric rating scale

Worst-itch numerical rating scale (WI-NRS) is a PRO comprised of a single item rated on a scale from 0 ("No itch") to 10 ("Worst imaginable itch"). Participants are asked to rate the intensity of their worst pruritus (itch) over the past 24 hours using this scale.

Participants will complete the WI-NRS daily according to the SoA (see Section 1.3). The assessment tool is provided in Section 10.7.1.

8.1.2 Investigator's global assessment for prurigo nodularis

Investigator's global assessment for prurigo nodularis (IGA PN) is a clinician-reported outcome (ClinRO) that allows clinicians to assess the activity of PN (IGA PN-A) using a 5-point scale from 0 (clear) to 4 (severe); and the stage of the disease (IGA PN-S) using a 5-point scale from 0 (clear) to 4 (severe).

Clinicians will complete the IGA PN as described in the SoA (see Section 1.3). The assessment tool is provided in Section 10.7.2.

8.1.3 Prurigo activity score

The PAS is a ClinRO measurement. The original PAS questionnaire Version 0.9 consists of 7 items, developed by expert clinicians in PN (25). The items of the PAS evaluate the pruriginous lesions in terms of:

- Type (visible lesions: Item 1a; predominant lesions: Item 1b);
- Estimated number (Item 2);
- Distribution (Item 3, 4);
- Size (biggest lesion: Item 6a; representative lesion: Item 6b).

Other items evaluate the representative body area and exact number of lesions (Item 5), the activity in terms of percentage of pruriginous lesions with excoriations/crusts on top (reflecting active scratching; Item 7a) and the percentage of healed pruriginous lesions (reflecting healing of chronic prurigo; Item 7b).

A 5-item simplified version of the PAS will be used in the current study. In particular, Item 3 (lesion distribution) and Item 6 (lesion monitoring) of the original PAS were removed, and the response options slightly modified and refined.

Clinicians will complete the screening/baseline version at screening and baseline visits, and will complete the follow-up version of the modified PAS at the other visits, as reported in the SoA (see Section 1.3). The assessment tool is provided in Section 10.7.3.

8.1.4 Dermatology life quality index

The DLQI is a PRO developed to measure dermatology-specific HRQoL in adult patients (27). The instrument comprises 10 items assessing the impact of skin disease on patients' HRQoL over the previous week. The items cover symptoms, leisure activities, work/school or holiday time, personal relationships including intimate, the side effects of treatment, and emotional reactions to having a skin disease. It is a validated questionnaire used in clinical practice and clinical trials (26). Response scale is a 4-point Likert scale (0 = "not at all" and 3 = "very much") for nine items. The remaining one item about work/studying asks whether work/study has been prevented and then (if "No") to what degree the skin condition has been a problem at work/study; the item is rated on a 3-point Likert scale ("Not at all" to "A lot"). Overall scoring ranges from 0 to 30, with a high score indicative of a poor HRQoL.

Participants will complete the questionnaire at time points according to SoA (see Section 1.3). The assessment tool is provided in Section 10.7.4.

8.1.5 Pain and sleep numeric rating scales

Participants will be asked to rate their worst skin pain in the past 24 hours using a 0 to 10 NRS, with 0 = No pain to 10 = Worst pain possible.

In addition, participants will be asked to rate their sleep quality on their past night upon awakening, using a 0 to 10 NRS, with 0 = Worst possible sleep and 10 = Best possible sleep.

Participants will complete the skin pain NRS and sleep quality NRS once a day.

The assessment tools are provided in Section 10.7.7 and Section 10.7.8.

8.1.6 Hospital anxiety and depression scale

The HADS is a PRO instrument for screening anxiety and depression in non-psychiatric populations; repeated administration also provides information about changes to a patient's emotional state (28, 29). The HADS consists of 14 items, 7 each for anxiety and depression symptoms; possible scores range from 0 to 21 for each subscale. The following cut-off scores are recommended for both subscales:

- 0 to 7: normal
- 8 to 10: borderline abnormal (borderline case)
- 11 to 21: abnormal

Participants will complete the questionnaire as described in the SoA (see Section 1.3). The assessment tool is provided in Section 10.7.5.

8.1.7 Patient Global Impression of Change of disease and Patient Global Impression of Severity

The PGIC is a one-item questionnaire that asks patients to provide the overall self-assessment of change in their PN overall on a 7-point scale, compared to just before patient started taking the study injection. Response choices are: 0 = "Very much better", 1 = "Moderately better", 2 = "A little better", 3 = "No change", 4 = "A little worse", 5 = "Moderately worse", 6 = "Very much worse".

The PGIS is a one-item questionnaire that asks patients to provide the overall self-assessment of their disease severity on a 4-point scale for the past week. Response choices are: 1 = "none", 2 = "Mild", 3 = "Moderate", 4 = "Severe".

Participants will complete the 2 items as described in the SoA (see Section 1.3). The items are provided in Section 10.7.9 and Section 10.7.10.

8.1.8 Euroqol 5 dimensions questionnaire

The Euroqol-5 dimensions (EQ-5D) is a standardized PRO measure of health status developed by the EuroQOL Group in order to provide a simple, generic measure of health for clinical and economic appraisal (30). The EQ-5D consists of 2 parts: the descriptive system and the EQ visual analog scale (VAS). The EQ-5D descriptive system comprises the following 5 dimensions: mobility, self-care, usual activities, pain/discomfort, and anxiety/depression. Each dimension has 5 levels of perceived problems: "no problem", "slight problems", "moderate problems", "severe problems" and "inability to do the activity". The respondent is asked to indicate his/her health state by ticking (or placing a cross) in the box against the most appropriate statement in each of the 5 dimensions; this results in a 1-digit number expressing the level for that dimension. The digits for 5 dimensions can be combined in a 5-digit number describing the respondent's health state. The EQ VAS records the respondent's self-rated health on a vertical VAS where the endpoints are labeled "best imaginable health state (100)" and "worst imaginable health state (0)". This information can be used as a quantitative measure of health outcome as judged by the individual respondents.

Participants will complete the questionnaire as described in the SoA (see Section 1.3). The assessment tool is provided in Section 10.7.6.

8.1.9 Missed school/work days

Participants who are employed or enrolled in school will be asked to report the number of sick leave/missed school days since the last study assessment.

Participants will complete the questionnaire as described in the SoA (see Section 1.3). The assessment tool is provided in Section 10.7.11.

8.1.10 Photography

Participants in selected sites who decide to participate in this substudy need to provide separate consent as described in the SoA (see Section 1.3). One or several lesions will be photographed at baseline. The same lesions will be photographed at subsequent visits to evaluate their progression. The detailed information will be provided in the photography user guide.

8.2 SAFETY ASSESSMENTS

Planned time points for all safety assessments are provided in the SoA (see Section 1.3).

8.2.1 Physical examinations

- A complete physical examination will include, at a minimum, assessments of the skin (full body skin exam), nasal cavities, eyes, ears, respiratory, cardiovascular, gastrointestinal, neurological, lymphatic, and musculoskeletal systems.
- Participants must be disrobed and provided with a hospital gown before the skin examination.
- Investigators should pay special attention to clinical signs related to previous serious illnesses.
- Any new finding or worsening of previous finding should be reported as a new AE.

8.2.2 Vital signs

- Vital signs will be measured in a semi-supine or sitting position after 5 minutes rest and will include body temperature, SBP and DBP, and pulse and respiratory rate. Blood pressure and pulse measurements should be assessed using the same arm with a completely automated device. Manual techniques will be used only if an automated device is not available.
- Body weight (kg) will be measured at screening (Visit 1), EOT, and EOS visits. Height will be measured at screening visit (Visit 1). Height and weight should be measured with indoor clothing but without shoes.

8.2.3 Electrocardiograms

- Single 12-lead ECG will be obtained as outlined in the SoA (see Section 1.3) using an ECG machine that automatically calculates the heart rate and measures PR, QRS, QT, and QTcF intervals. Refer to Section 7 for QTcF withdrawal criteria and any additional QTcF readings that may be necessary. The ECG should be recorded after 10 minutes of rest in the supine position.

8.2.4 Clinical safety laboratory assessments

- See Appendix 2 (Section 10.2) for the list of clinical laboratory tests to be performed and to the SoA (see Section 1.3) for the timing and frequency.

- The Investigator must review the laboratory report, document this review, and record any clinically relevant changes occurring during the study in the AE section of the CRF. The laboratory reports must be filed with the source documents. Clinically significant abnormal laboratory findings are those which are not associated with the underlying disease, unless judged by the Investigator to be more severe than expected for the participant's condition.
- All laboratory tests with values considered clinically significantly abnormal during participation in the study or within 12 weeks after the last dose of study intervention should be repeated until the values return to normal or baseline or are no longer considered clinically significant by the Investigator or medical monitor.
  - If such values do not return to normal/baseline within a period of time judged reasonable by the Investigator, the etiology should be identified and the Sponsor notified,
  - All protocol-required laboratory assessments, as defined in Appendix 2 (Section 10.2), must be conducted in accordance with the laboratory manual and the SoA (see Section 1.3),
  - If laboratory values from non-protocol specified laboratory assessments performed at the institution's local laboratory require a change in participant management or are considered clinically significant by the Investigator (eg, SAE or AE or dose modification), then the results must be recorded in the CRF.

8.3 ADVERSE EVENTS AND SERIOUS ADVERSE EVENTS

Adverse event of special interest

An AESI is an AE (serious or nonserious) of scientific and medical concern specific to the Sponsor's product or program, for which ongoing monitoring and immediate notification by the Investigator to the Sponsor is required. Such events may require further investigation in order to characterize and understand them. Adverse events of special interest may be added, modified or removed during a study by protocol amendment.

For these AESIs, the Sponsor will be informed immediately (ie, within 24 hours), per SAE notification described in Section 10.3, even if not fulfilling a seriousness criterion, using the corresponding pages in the CRF (to be sent) or screens in the eCRF. If an SAE or any AESI of anaphylactic reactions or systemic allergic reactions that are related to IMP and require treatment, or severe injection site reactions lasting longer than 24 hours, occurs in a participant, blood samples should be collected for determination of functional dupilumab concentration, and ADA assessment at or near the onset and completion of the occurrence of the event, if possible.

- Anaphylactic reactions.
- Systemic hypersensitivity reactions.
- Helminthic infections.
- Any severe type of conjunctivitis or blepharitis.
- Keratitis.

- Clinically symptomatic eosinophilia (or eosinophilia associated with clinical symptoms).
- Pregnancy of a female subject entered in a study as well as pregnancy occurring in a female partner of a male subject entered in a study with IMP/NIMP;
  - Pregnancy occurring in a female participant entered in the clinical trial or in a female partner of a male participant entered in the clinical trial. It will be qualified as an SAE only if it fulfills one of the seriousness criteria,
  - In the event of pregnancy in a female participant, IMP should be discontinued,
  - Follow-up of the pregnancy in a female participant or in a female partner of a male participant is mandatory until the outcome has been determined,
  - Abnormal pregnancy outcomes (eg, spontaneous abortion, fetal death, stillbirth, congenital anomalies, ectopic pregnancy) are considered SAEs.
- Significant ALT elevation
  - ALT >5 x ULN in participants with baseline ALT ≤2 x ULN;

    OR
  - ALT >8 x ULN if baseline ALT >2 x ULN.
- Symptomatic overdose (serious or nonserious) with IMP/NIMP
  - An overdose (accidental or intentional) with the IMP is an event suspected by the Investigator or spontaneously notified by the participant and defined as at least twice the intended dose during an interval of less than 11 days. The circumstances (ie, accidental or intentional) should be clearly specified in the verbatim and symptoms, if any, entered on separate AE forms.
  - An overdose (accidental or intentional) with any NIMP is an event suspected by the Investigator or spontaneously notified by the participant and defined as at least twice the maximum prescribed daily dose, within the intended therapeutic interval. The circumstances (ie, accidental or intentional) should be clearly specified in the overdose form.

The definitions of an AE or SAE can be found in Appendix 3 (Section 10.3).

Adverse events will be reported by the participant (or, when appropriate, by a caregiver, surrogate, or the participant's legally authorized representative).

The Investigator and any qualified designees are responsible for detecting, documenting, and recording events that meet the definition of an AE or SAE and remain responsible for following up AEs that are serious, considered related to the study intervention or study procedures, or that caused the participant to discontinue the study intervention (see Section 7).

8.3.1 Time period and frequency for collecting AE and SAE information

All AEs, serious or nonserious, will be collected from the signing of the ICF until the EOS visit at the time points specified in the SoA (Section 1.3).

All SAEs and AESI will be recorded and reported to the Sponsor or designee immediately and under no circumstance should this exceed 24 hours, as indicated in Appendix 3 (Section 10.3). The Investigator will submit any updated SAE data to the Sponsor within 24 hours of it being available.

Investigators are not obligated to actively seek AE or SAE after conclusion of the study participation. However, if the Investigator learns of any SAE, including a death, at any time after a participant has been discharged from the study, and he/she considers the event to be reasonably related to the study intervention or study participation, the Investigator must promptly notify the Sponsor.

8.3.2 Method of detecting AEs and SAEs

The method of recording, evaluating, and assessing causality of AE and SAE and the procedures for completing and transmitting SAE reports are provided in Appendix 3 (Section 10.3).

Care will be taken not to introduce bias when detecting AEs and/or SAEs. Open-ended and non-leading verbal questioning of the participant is the preferred method to inquire about AE occurrences.

8.3.3 Follow-up of AEs and SAEs

After the initial AE/AESI/SAE report, the Investigator is required to proactively follow each participant at subsequent visits/contacts. At the pre-specified study end-date, all SAEs and nonserious AESI (as defined in Section 8.3) will be followed until resolution, stabilization, the event is otherwise explained, or the participant is lost to follow-up (as defined in Section 7.3). Further information on follow-up procedures is given in Appendix 3 (Section 10.3).

8.3.4 Regulatory reporting requirements for SAEs

- Prompt notification by the Investigator to the Sponsor of a SAE is essential so that legal obligations and ethical responsibilities towards the safety of participants and the safety of a study intervention under clinical investigation are met.
- The Sponsor has a legal responsibility to notify both the local regulatory authority and other regulatory agencies about the safety of a study intervention under clinical investigation. The Sponsor will comply with country-specific regulatory requirements relating to safety reporting to the regulatory authority, Institutional Review Boards (IRB)/Independent Ethics Committees (IEC), and Investigators.
- Investigator safety reports must be prepared for suspected unexpected serious adverse reactions (SUSAR) according to local regulatory requirements and Sponsor policy and forwarded to Investigators as necessary.
- Adverse events that are considered expected will be specified in the reference safety information.
- An Investigator who receives an Investigator safety report describing a SAE or other specific safety information (eg, summary or listing of SAEs) from the Sponsor will review and then file it along with the IB and will notify the IRB/IEC, if appropriate according to local requirements.

8.3.5 Pregnancy

- Details of all pregnancies in female participants and female partners of male participants will be collected after the start of study intervention and until the outcome has been determined.
- If a pregnancy is reported, the Investigator should inform the Sponsor within 24 hours of learning of the pregnancy and should follow the procedures outlined in Appendix 4 (Section 10.4).
- Abnormal pregnancy outcomes (eg, spontaneous abortion, fetal death, stillbirth, congenital anomalies, ectopic pregnancy) are considered SAEs.

8.3.6 Guidelines for reporting product complaints

Any defect in the IMP/NIMP/device must be reported as soon as possible by the Investigator to the monitoring team that will complete a product complaint form within required timelines.

Appropriate information (eg, samples, labels or documents like pictures or photocopies) related to product identification and to the potential deficiencies may need to be gathered. The Investigator will assess whether or not the quality issue has to be reported together with an AE or SAE.

8.4 TREATMENT OF OVERDOSE

Overdose is an AESI (defined in Section 8.3). No antidote is available for dupilumab. Sponsor does not recommend specific treatment for an overdose.

In the event of an overdose, the Investigator/treating physician should:

1. Provide symptomatic care.
2. Contact the Medical Monitor immediately.
3. Closely monitor the participant for any AE/SAE and laboratory abnormalities until dupilumab can no longer be detected systemically.
4. Obtain a plasma sample for PK analysis as soon as possible if requested by the Medical Monitor (determined on a case-by-case basis).
5. Document appropriately in the CRF.

Decisions regarding dose interruptions will be made by the Investigator in consultation with the Medical Monitor based on the clinical evaluation of the participant.

8.5 PHARMACOKINETICS

8.5.1 Systemic drug concentration and antidrug antibodies

8.5.1.1 Sampling time

Blood samples will be collected for determination of functional dupilumab and anti-dupilumab antibodies in serum as specified in the SoA (Section 1.3). Special procedures for collection, storage, and shipping of serum are described in separate operational manuals. The date of collection should be recorded in the eCRF.

8.5.1.2 Handling procedures

Special procedures for collection, storage, and shipping of serum are described in separate operational manuals. An overview of handling procedure for samples used in the determination of systemic drug concentration and ADA is provided in Table 3.

Table 3 - Summary of handling procedures

Sample type	Functional dupilumab	Anti-dupilumab antibody
Matrix	Serum	Serum
Blood sample volume	5 mL	5 mL
Anticoagulant	None	None
Blood handling procedures	See Operational Manual	See Operational Manual
Serum aliquot split	2 aliquots	2 aliquots
Storage conditions	<6 months: below -20°C<br><24 months: below -80°C (preferred)	<6 months: below -20°C<br><24 months: below -80°C (preferred)
Serum shipment condition	In dry ice	In dry ice

8.5.1.3 Bioanalytic method

Serum PK and ADA samples will be assayed using validated methods as described in Table 4.

Table 4 - Summary of bioanalytical methods for functional dupilumab and anti-dupilumab antibodies

[redacted]

Note: In the event of any SAE, any AE of severe injection site reaction lasting longer than 24 hours, or any AESI of anaphylactic reaction or systemic allergic reaction that is related to IMP and require treatment, PK and ADA samples will be collected at or near the onset of the event for

any additional analysis if required or for archival purposes. The exact date and time of sample collection must be recorded and entered into the database by the central laboratory. An unscheduled systemic drug concentration page in the eCRF must be completed as well. If necessary for safety monitoring, additional PK and ADA samples may be collected after the EOS Visit until resolution of AE.

Specifically for PK, any changes in the timing or addition of time points for any planned study assessments must be documented and approved by the relevant study team member and then archived in the Sponsor and site study files, but will not constitute a protocol amendment. The IRB/IEC will be informed of any safety issues that require alteration of the safety monitoring scheme or amendment of the ICF.

8.6 PHARMACODYNAMICS

See Section 8.8 for IgE measurement. No other PD parameters will be evaluated in this study.

8.7 GENETICS

For those participants who consent to the optional pharmacogenetic/pharmacogenomic sample collection section of the ICF, blood samples for exploratory genetic analysis of DNA or RNA will be collected and stored for possible future use (see Section 10.5). Participation is optional. Participants who do not wish to participate in the genetic research may still participate in the study.

In the event of DNA extraction failure, a replacement genetic blood sample may be requested from the participant.

Details on processes for collection and shipment and destruction of these samples can be found in the laboratory manual.

8.8 BIOMARKERS

- Venous blood samples will be collected for measurement of total serum IgE. Total IgE will be measured using validated quantitative methods.
- Optional samples for biomarker research that should be collected from participants in the study with their consent are the following:
  - Serum and plasma: samples will be collected and stored for possible future analysis of potential biomarkers of drug response, disease activity, safety, and type 2 inflammation,
  - Skin biopsy (sub-study): samples will be taken from lesion and non-lesion skin using punch biopsy; gene expression and immunohistological analyses may be used to investigate drug response, disease activity, and type 2 inflammation.

Plasma, serum and skin biopsy samples may be stored for a maximum of 5 years, and DNA or RNA samples for 15 years (or according to local regulations) following the last participant's last visit for the study at a facility selected by the Sponsor.

8.9 MEDICAL RESOURCE UTILIZATION AND HEALTH ECONOMICS

Number of missed school/work days collection is described in Section 8.1.9.

9 STATISTICAL CONSIDERATIONS

9.1 STATISTICAL HYPOTHESES

The statistical hypotheses for comparing dupilumab to placebo on the primary endpoint of the proportion of participants with WI-NRS reduction of ≥4 (minimally important difference) at Week 24 are as follows:

- Null hypothesis H0: No treatment difference between dupilumab and placebo.
- Alternative hypothesis H1: There is a treatment difference between dupilumab and placebo.

The statistical hypotheses to be tested on secondary endpoints can be specified similarly to those on the primary endpoint.

9.2 SAMPLE SIZE DETERMINATION

The primary endpoint is the proportion of participants with WI-NRS reduction of ≥4 from baseline to Week 24. By assuming the response rate is [redacted] and [redacted] in placebo and dupilumab respectively, 56 participants/arm will provide 90% power to detect the difference of [redacted] between dupilumab and placebo with Fisher exact test at 2-sided level of 0.05. Assuming 15% drop out during treatment, the target is to randomize 75 participants/arm with a cap of up to 10% of participants in the atopic population having mild active AD.

Approximately 150 participants will be randomized 1:1. This corresponds to approximately 75 participants that will be randomly assigned to each intervention arm.

The sample size calculation was performed using SAS 9.4 power procedure. The assumptions were based on the effect of dupilumab versus placebo in WI-NRS reduction ≥4 at Week 16 observed in patients with moderate to severe AD as seen in studies of R668-AD-1334 (Solo1) and R668-AD-1416 (Solo2).

Participants will be randomized to dupilumab or placebo in 1:1 ratio with stratification factors of documented history of atopy (atopic or non-atopic, see definition in Section 6.3), stable use of TCS/TCI (yes or no), and country/territory code. Both the atopic and the non-atopic PN population will be capped at 60% of the total enrolled population.

9.3 POPULATIONS FOR ANALYSES

For purposes of analysis, the following populations are defined (Table 5):

Table 5 - Populations for analyses

Population	Description
Screened	All participants who sign the ICF.
Randomized	The randomized population includes all participants with a treatment kit number allocated and recorded in the IRT database, and regardless of whether the treatment kit was used or not.<br>Participants treated without being randomized will not be considered randomized and will not be included in any efficacy population.
Intent-to-treat (ITT)	All randomized participants analyzed according to the treatment group allocated by randomization regardless if treatment kit is used or not
Efficacy	The analysis population for the efficacy endpoints will be the ITT population.
Safety	All participants randomly assigned to study intervention and who take at least 1 dose of study intervention. Participants will be analyzed according to the intervention they actually received.<br>Randomized participants for whom it is unclear whether they took the study medication will be included in the safety population as randomized.<br>For participants who accidentally receive different treatment from the planned, the actual intervention allocation for as-treated analysis will be the dupilumab group.<br>The PD analyses will be performed on the safety population.
Pharmacokinetic (PK)	The PK population includes all participants in the safety population with at least one non-missing result for functional dupilumab concentration in serum after first dose of the study treatment. Participants will be analyzed according to the intervention actually received.
Antidrug antibody (ADA)	ADA population includes all participants in the safety population who have at least one non-missing ADA result after first dose of the study treatment. Participants will be analyzed according to the intervention actually received.

ADA: antidrug antibody; ICF: informed consent form; IRT: interactive response technology; ITT: intent to treat; PD: pharmacodynamics: PK: pharmacokinetic.

9.4 STATISTICAL ANALYSES

The SAP will be developed and finalized before primary database lock and will describe the participant populations to be included in the analyses, and procedures for accounting for missing, unused, and spurious data. This section is a summary of the planned statistical analyses of the primary and secondary endpoints.

9.4.1 Efficacy analyses

The multiplicity procedure is proposed to control the overall Type-I error rate for testing the primary endpoint and the key secondary endpoints. Detailed hierarchical testing procedure will be defined in the study SAP. The study is considered positive when the primary endpoint achieves statistical significance with two-sided significance level of 0.05.

The analysis population for the efficacy endpoints will be the ITT population.

Modification in accordance with the above proposal will be documented in the SAP prior to the database lock.

Table 6 - Efficacy analyses

Endpoint	Statistical analysis methods
<u>Primary</u>:	
Proportion of participants with improvement (reduction) in WI-NRS by ≥4 from baseline to Week 24	The primary analysis will be conducted by using CMH test stratifying by stratification factors (documented history of atopy [atopic or non-atopic], stable use of TCS/TCI [yes or no], and region [countries combined]) and covariate of baseline anti-depressant use (yes or no).<br>For participants discontinuing the study treatment before Week 24, their off-study treatment values measured up to Week 24 will be included in the analysis. Participants taking selected prohibited medications and/or rescue medications (details of selection will be provided in the SAP) prior to Week 24 or have missing data at Week 24 will be considered non-responders.<br>*Sensitivity analyses:*<br>The data collected after taking selected prohibited medications and/or rescue medications will not be censored and included in the sensitivity analysis to evaluate the robustness of the primary analysis results with respect to the method of handling data while taking selected prohibited medications. Details of the sensitivity analyses will be provided in the SAP.<br>*Subgroup analyses:*<br>To assess the consistency in treatment effects across different subgroup levels, subgroup analyses will be performed for the primary efficacy endpoint with respect to documented history of atopy (atopic or non-atopic), age group, gender, region, and other factors that will be specified in the SAP. A subgroup analysis will be performed excluding participants with a current diagnosis of AD.
Key secondary:<br>Proportion of participants with IGA PN-S 0 or 1 score at Week 24.	The key secondary efficacy endpoint that measures binary responses will be analyzed in the same fashion as the primary endpoint.

Endpoint	Statistical analysis methods
Secondary:	
Proportion of participants with improvement (reduction) in WI NRS by ≥4 from baseline to Week 12<br>Proportion of participants with WI-NRS reduction ≥4 over time until Week 24.<br>Proportion of participants with IGA PN-S 0 or 1 score at Week 12.<br>Proportion of participants with IGA PN-S 0 or 1 score at Week 8.<br>Proportion of participants with IGA PN-S 0 or 1 score at Week 4.<br>Proportion of participants with IGA PN-A 0 or 1 score at Week 24.<br>Proportion of participants with IGA PN-A 0 or 1 score at Week 12.<br>Proportion of participants with IGA PN-A 0 or 1 score at Week 8.<br>Proportion of participants with IGA PN-A 0 or 1 score at Week 4.<br>Proportion of participants with WI-NRS reduction ≥4 at Week 4.	Secondary efficacy endpoints that measure binary responses will be analyzed in the same fashion as the primary endpoint.
Time to onset of effect on pruritus as measured by proportion of participants with improvement (reduction) in WI-NRS by ≥4 from baseline during the 24-week treatment period.	This time-to-event endpoint will be analyzed using the Cox proportional hazards model, including treatment, stratification factors (countries combined to region), and covariate of baseline anti-depressant use (yes or no). The hazards ratio, its 95% confidence interval and p-value will be reported. Kaplan-Meier curves will be also provided.
Change from baseline in WI-NRS at Week 24.<br>Change from baseline in WI-NRS at Week 12.<br>Percent change from baseline in WI-NRS at Week 24.<br>Percent change from baseline in WI-NRS at Week 12.<br>Percent change from baseline in WI-NRS at Week 4.<br>Percent change from baseline in WI-NRS at Week 2.<br>Percent change from baseline in WI-NRS over time until Week 24.<br>Change from baseline in IGA PN-S score at Week 24.<br>Change from baseline in IGA PN-S score at Week 12.<br>Change from baseline in IGA PN-S score at Week 8.	Continuous secondary efficacy endpoints will be analyzed using a hybrid method of the WOCF and multiple imputations.<br>Data of participants taking selected prohibited medications and/or rescue medications will be set to missing after the medication usage, and the worst postbaseline value on or before the time of the medication usage will be used to impute missing endpoint value (for participants whose postbaseline values are all missing, the baseline will be used to impute).<br>Participants who discontinue the treatment prematurely are encouraged to follow the planned clinical visits and in those participants who did not take the selected prohibited medications and/or rescue medications, all data collected after treatment discontinuation will be used in the analysis. For these participants, missing data may still happen despite all efforts have been tried to collect the data after treatment discontinuation. For participants who discontinue due to lack of efficacy, all data collected after discontinuation will be used in the analysis, and a WOCF approach will be used to impute missing data if needed. For participants who discontinued not due to lack of efficacy, a multiple imputation approach will be used to impute missing endpoint value, and this multiple imputation will use all participants excluding patients who have taken the selected prohibited medications and/or rescue medications prior to timepoint of endpoint of interest and excluding participants who discontinue due to lack of efficacy.<br>Each of the imputed complete data will be analyzed by fitting an ANCOVA model with treatment group, stratification factors (documented history of atopy [atopic or non-

Endpoint	Statistical analysis methods
Change from baseline in IGA PN-S score at Week 4.<br>Change from baseline in HRQoL, as measured by DLQI to Week 24.<br>Change from baseline in HRQoL, as measured by DLQI to Week 12.<br>Onset of action in change from baseline in WI-NRS (first p <0.05 difference from placebo in the daily WI-NRS that remains significant at subsequent measurements) until Week 12.	atopic], stable use of TCS/TCI [yes or no], and region [countries combined]), baseline antidepressant use (yes or no), and relevant baseline measurement as covariates included in the model. Statistical inference obtained from all imputed data will be combined using Rubin's rule. Descriptive statistics including number of subjects, mean, standard error, and least squares (LS) mean change and standard error will be provided. In addition, difference of the dupilumab group against placebo in LS means and the corresponding 95% confidence interval will be provided along with the p-values.<br>Detailed analyses will be described in the SAP finalized before database lock. The sensitivity analyses will be also detailed in the SAP to evaluate the robustness of the hybrid method of the WOCF and multiple imputations handling intercurrent events such as taking prohibited medication, rescue medication, or missing data due to study treatment withdrawal.
<u>Tertiary/exploratory</u>	Will be described in the SAP finalized before database lock.

ANCOVA: analysis of covariance; CMH: Cochran-Mantel-Haenszel; DLQI: Dermatology Life Quality Index; IGA: Investigator's global assessment; LS: Least squares; NRS: numeric rating scale; PAS: prurigo activity score; WI-NRS: worst itch numeric rating scale.

9.4.2 Safety analyses

All safety analyses will be performed on the safety population. The summary of safety results will be presented by treatment group. The baseline value is defined generally as the last available value before randomization.

Table 7 - Safety analyses

Endpoint	Statistical analysis methods
Percentage of participants experiencing TEAEs or SAEs from baseline through Week 24	Adverse event incidence tables will present by SOC (sorted by internationally agreed order), and PT sorted in alphabetical order for each treatment group, the number (n) and percentage (%) of participants experiencing an AE. In addition, exposure-adjusted AE incidence rate tables will provide the number of patients with at least 1 event per 100 patient-years, presented by SOC and PT. Multiple occurrences of the same event in the same participant will be counted only once in the tables within a treatment phase. The denominator for computation of percentages is the safety population within each treatment group.<br>Proportion of participants with at least one TEAE, treatment emergent SAE, TEAE leading to death, and TEAE leading to permanent treatment discontinuation will be tabulated by treatment group. In addition, TEAEs will be described according to maximum intensity and relation to the study intervention. Serious AEs and AEs leading to study discontinuation that occur outside the treatment-emergent period will be summarized separately.

AE: adverse event; PT: preferred term; SOC: system organ class; SAE: serious adverse event; TEAE: treatment-emergent adverse event.

9.4.3 Other analyses

Table 8 - Other analyses

Endpoint	Statistical analysis methods
Incidence of treatment-emergent ADA against dupilumab over time	The ADA analysis will be conducted on ADA population. ADA variables including treatment-emergent ADA will be summarized using descriptive statistics by treatment group. Drug concentration data will be examined and the influence of ADAs on individual concentration-time profiles will be evaluated. Assessment of the potential impact of ADA on safety and efficacy may be provided.

ADA: antidrug antibodies.

Other PK, PD, biomarker, and outcome measures exploratory analyses will be described in the SAP finalized before database lock. The population PK analysis and PD analyses will be presented separately from the main CSR.

Data collected regarding the impact of the COVID-19 or other pandemics, on the patients will be summarized (eg, discontinuation due to COVID-19). Any additional analyses and methods required to investigate the impact of COVID-19 or other pandemics requiring public health emergency on the efficacy (eg, missing data due to COVID-19) and safety will be detailed in the SAP.

9.5 INTERIM ANALYSES

No interim analysis is planned.

9.6 TIMING OF PRIMARY DATABASE LOCK

A primary database lock is currently planned to be performed when all randomized participants have completed their 24-week treatment phase.

The database will be updated at the end of the study for all participants to include the data collected after the primary data cut-off and updates for the events previously ongoing at the time of the primary database lock. Additional data collected between the primary database lock and last participant completing last visit will be summarized in a separate CSR addendum, as needed. Details will be included in the SAP.

9.7 DATA MONITORING COMMITTEE (DMC)

Due to extensive safety record of the post marketed IMP (dupilumab), it is not planned to have a DMC for this study.

9.8 UNBLINDING PLAN

Unblinding plan is not applicable for this study.

10 SUPPORTING DOCUMENTATION AND OPERATIONAL CONSIDERATIONS

10.1 APPENDIX 1: REGULATORY, ETHICAL, AND STUDY OVERSIGHT CONSIDERATIONS

10.1.1 Regulatory and Ethical Considerations

- This study will be conducted in accordance with the protocol and with the following:
  - Consensus ethical principles derived from international guidelines including the Declaration of Helsinki and the applicable amendments and Council for International Organizations of Medical Sciences (CIOMS) International Ethical Guidelines.
  - Applicable ICH GCP Guidelines.
  - Applicable laws and regulations.
- The protocol, protocol amendments, ICF, Investigator Brochure, and other relevant documents (eg, advertisements) must be submitted to an IRB/IEC by the Investigator and reviewed and approved by the IRB/IEC before the study is initiated.
- Any amendments to the protocol will require IRB/IEC approval before implementation of changes made to the study design, except for changes necessary to eliminate an immediate hazard to study participants.
- The Investigator will be responsible for the following:
  - Providing written summaries of the status of the study to the IRB/IEC annually or more frequently in accordance with the requirements, policies, and procedures established by the IRB/IEC.
  - Notifying the IRB/IEC of SAEs or other significant safety findings as required by IRB/IEC procedures.
  - Providing oversight of the conduct of the study at the site and adherence to requirements of 21 Code of Federal Regulations (CFR), ICH guidelines, the IRB/IEC, European regulation 536/2014 for clinical studies (if applicable), and all other applicable local regulations.

10.1.2 Financial disclosure

Investigators and sub-Investigators will provide the Sponsor with sufficient, accurate financial information as requested to allow the Sponsor to submit complete and accurate financial certification or disclosure statements to the appropriate regulatory authorities. Investigators are responsible for providing information on financial interests during the course of the study and for 1 year after completion of the study.

10.1.3 Informed consent process

- The Investigator or his/her representative will explain the nature of the study to the participant or his/her legally authorized representative and answer all questions regarding the study.
- Participants must be informed that their participation is voluntary. Participants will be required to sign a statement of informed consent that meets the requirements of 21 CFR 50, local regulations, ICH guidelines, Health Insurance Portability and Accountability Act requirements, where applicable, and the IRB/IEC or study center.
- The medical record must include a statement that written informed consent was obtained before the participant was enrolled in the study and the date the written consent was obtained. The authorized person obtaining the informed consent must also sign the ICF.
- Participants must be re-consented to the most current version of the ICF(s) during their participation in the study.
- A copy of the ICF(s) must be provided to the participant or the participant’s legally authorized representative.

Participants who are rescreened are required to sign a new ICF.

The ICF will contain a separate section that addresses the use of remaining mandatory samples as well as additional serum/plasma samples for optional exploratory research. The Investigator or authorized designee will explain to each participant the objectives of the exploratory research. Participants will be told that they are free to refuse to participate and may withdraw their consent at any time and for any reason during the storage period. An optional consent will be required to document a participant's agreement to allow any remaining specimens to be used for exploratory research. Participants who decline to participate in this optional research will not provide this optional consent.

The ICF will also contains a separate section concerning the other optional procedures (genetic testing [DNA/RNA sampling] and, for selected sites, skin biopsy and photography). An optional consent will be required to document a participant’s agreement to each of these procedures. Participants who decline to participate will not provide this optional consent. A specific consent will be provided for HIV testing if specific consent is locally required.

10.1.4 Data protection

All personal data collected related to participants, Investigators, or any person involved in the study, which may be included in the Sponsor’s databases, shall be treated in compliance with all applicable laws and regulations including the Global Data Protection Regulation.

Data collected must be adequate, relevant and not excessive, in relation to the purposes for which they are collected. Each category of data must be properly justified and in line with the study objective.

Participant race and ethnicity will be collected in this study because these data are required by regulatory agencies (eg, for reporting effectiveness data by racial subgroup in marketing authorization applications for FDA, or to report on the Japanese population for the Pharmaceuticals and Medical Devices Agency in Japan). In addition, race has been identified to influence PN incidence (15).

- Participants will be assigned a unique identifier by the Sponsor. Any participant records or datasets that are transferred to the Sponsor will contain the identifier only; participant names or any information which would make the participant identifiable will not be transferred.
- The participant must be informed that his/her personal study-related data will be used by the Sponsor in accordance with local data protection law. The level of disclosure must also be explained to the participant.
- The participant must be informed that his/her medical records may be examined by Clinical Quality Assurance auditors or other authorized personnel appointed by the Sponsor, by appropriate IRB/IEC members, and by inspectors from regulatory authorities.
- When archiving or processing personal data pertaining to the Investigator and/or to the participants, the Sponsor shall take all appropriate measures to safeguard and prevent access to this data by any unauthorized third party.

10.1.5 Committees structure

There will be no study committees.

10.1.6 Dissemination of clinical study data

Sanofi shares information about clinical trials and results on publically accessible websites, based on company commitments, international and local legal and regulatory requirements, and other clinical trial disclosure commitments established by pharmaceutical industry associations. These websites include clinicaltrials.gov, EU clinicaltrialregister (eu.ctr), and sanofi.com, as well as some national registries.

In addition, results from clinical trials in patients are required to be submitted to peer-reviewed journals following internal company review for accuracy, fair balance and intellectual property. For those journals that request sharing of the analyzable data sets that are reported in the publication, interested researchers are directed to submit their request to clinicalstudydatarequest.com.

Individual participant data and supporting clinical documents are available for request at clinicalstudydatarequest.com. While making information available we continue to protect the privacy of participants in our clinical trials. Details on data sharing criteria and process for requesting access can be found at this web address: clinicalstudydatarequest.com.

10.1.7 Data quality assurance

- All participant data relating to the study will be recorded on printed or electronic CRF unless transmitted to the Sponsor or designee electronically (eg, laboratory data). The Investigator is responsible for verifying that data entries are accurate and correct by physically or electronically signing the CRF.
- The Investigator must maintain accurate documentation (source data) that supports the information entered in the CRF.
- The Investigator must permit study-related monitoring, audits, IRB/IEC review, and regulatory agency inspections and provide direct access to source data documents.
- Monitoring details describing strategy (eg, risk-based initiatives in operations and quality such as Risk Management and Mitigation Strategies and Analytical Risk-Based Monitoring), methods, responsibilities and requirements, including handling of noncompliance issues and monitoring techniques (central, remote, or on-site monitoring) are provided in separate study documents.
- The Sponsor or designee is responsible for the data management of this study including quality checking of the data.
- The Sponsor assumes accountability for actions delegated to other individuals (eg, Contract Research Organizations).
- Study monitors will perform ongoing source data verification to confirm that data entered into the CRF by authorized site personnel are accurate, complete, and verifiable from source documents; that the safety and rights of participants are being protected; and that the study is being conducted in accordance with the currently approved protocol and any other study agreements, ICH GCP, and all applicable regulatory requirements.
- Records and documents, including signed ICFs, pertaining to the conduct of this study must be retained by the Investigator for 25 years after the signature of the final study report unless local regulations or institutional policies require a longer retention period. No records may be destroyed during the retention period without the written approval of the Sponsor. No records may be transferred to another location or party without written notification to the Sponsor.

10.1.8 Source documents

- Source documents provide evidence for the existence of the participant and substantiate the integrity of the data collected. Source documents are filed at the Investigator's site.
- Data reported on the CRF or entered in the eCRF that are transcribed from source documents must be consistent with the source documents or the discrepancies must be explained. The Investigator may need to request previous medical records or transfer records, depending on the study. Also, current medical records must be available.

- Source data is all information in original records and certified copies of original records of clinical findings, observations, or other activities in a clinical trial necessary for the reconstruction and evaluation of the trial. Source data are contained in source documents. Source documents are original documents, data and records such as hospital records, clinic and office charts, laboratory notes, memoranda, pharmacy dispensing records, recorded data from automated instruments, etc.

10.1.9 Study and site closure

The Sponsor or designee reserves the right to close the study site or terminate the study at any time for any reason at the sole discretion of the Sponsor. Study sites will be closed upon study completion. A study site is considered closed when all required documents and study supplies have been collected and a study-site closure visit has been performed.

The Investigator may initiate study-site closure at any time, provided there is reasonable cause and sufficient notice is given in advance of the intended termination.

Reasons for study termination by the Sponsor, as well as reasons for the early closure of a study site by the Sponsor or Investigator may include but are not limited to:

- For study termination:
  - Information on the product leads to doubt as to the benefit/risk ratio,
  - Discontinuation of further study intervention development.
- For site termination:
  - Failure of the Investigator to comply with the protocol, the requirements of the IRB/IEC or local health authorities, the Sponsor's procedures, or GCP guidelines,
  - Inadequate or no recruitment (evaluated after a reasonable amount of time) of participants by the Investigator,
  - Total number of participants included earlier than expected.

10.1.10 Publication policy

- The results of this study may be published or presented at scientific meetings. If this is foreseen, the Investigator agrees to submit all manuscripts or abstracts to the Sponsor before submission. This allows the Sponsor to protect proprietary information and to provide comments.
- The Sponsor will comply with the requirements for publication of study results. In accordance with standard editorial and ethical practice, the Sponsor will generally support publication of multicenter studies only in their entirety and not as individual site data. In this case, a coordinating Investigator will be designated by mutual agreement.
- Authorship will be determined by mutual agreement and in line with International Committee of Medical Journal Editors authorship requirements.

10.2 APPENDIX 2: CLINICAL LABORATORY TESTS

The tests detailed in Table 9 will be performed by the central laboratory.

- Local laboratory results are only required in the event that the central laboratory results are not available in time for either study intervention administration and/or response evaluation. If a local sample is required, it is important that the sample for central analysis is obtained at the same time. Additionally, if the local laboratory results are used to make either a study intervention decision or response evaluation, the results must be entered into the CRF.
- Protocol-specific requirements for inclusion or exclusion of participants are detailed in Section 5 of the protocol.
- Additional tests may be performed at any time during the study as determined necessary by the Investigator or required by local regulations.
- Additional serum or urine pregnancy tests may be performed, as determined necessary by the investigator or required by local regulation, to establish the absence of pregnancy at any time during the subject's participation in the study.

Table 9 - Protocol-required laboratory assessments

Laboratory assessments	Parameters
Hematology	
	Platelet count
	RBC count
	Hemoglobin
	Hematocrit
	WBC count with differential:
	Neutrophils
	Lymphocytes
	Monocytes
	Eosinophils
	Basophils
	CD4 T cell count (for participants with HIV history only)
Clinical chemistry[a]	
	BUN
	Creatinine
	Glucose
	Lactate dehydrogenase
	Uric acid
	Total cholesterol
	Potassium

Laboratory assessments	Parameters
	Sodium<br>Chloride<br>Bicarbonate<br>AST/SGOT<br>ALT/SGPT<br>Alkaline phosphatase<br>Creatine phosphokinase<br>Total bilirubin<br>Total protein<br>Albumin<br>Hemoglobin A1c (only for diabetic patients without hemoglobin A1c laboratory results within 3 months before screening visit)
Urinalysis	• Specific gravity<br>• pH, glucose, protein, blood, ketones, bilirubin, urobilinogen, nitrite, leukocyte esterase by dipstick<br>• Creatinine and leukotriene (central laboratory)<br>• Microscopic examination (if blood or protein is abnormal)<br>• Tetranor PGDM (central laboratory)
Other screening tests	• Highly sensitive serum (at screening) or urine (at other timepoints) hCG pregnancy test (as needed for WOCBP)[b]<br>• Serology[c]: HBs Ag, HBs Ab, HBc Ab, HCV Ab, HIV screen (Anti-HIV-1 and HIV-2 antibodies); HIV viral load test (for participants with HIV history only)<br>• Tuberculosis test<br>• All study-required laboratory assessments will be performed by a central laboratory, with the exception of TB test, urine pregnancy test, and routine urinalysis.

NOTES:

a Details of liver chemistry stopping criteria and required actions and follow-up assessments after liver stopping or monitoring event are given in Section 7.1 and Section 10.6. All events which may indicate severe liver injury (meeting Hy's Law laboratory criteria; ALT or AST >3 x ULN and total bilirubin >2 x ULN) must be reported as an SAE.

b After screening, local urine testing will be standard for the protocol unless serum testing is required by local regulation or IRB/IEC.

c In case of results showing HBs Ag (negative) and HBc Ab (positive), an HBV DNA testing will be performed and should be confirmed negative prior to randomization. In case of results showing HCV Ab (positive), HCV RNA testing will be performed and should be confirmed negative prior to randomization.

ALT: alanine aminotransferase; AST: aspartate aminotransferase; BUN: blood urea nitrogen; DNA: deoxyribonucleic acidHBc Ab: hepatitis B core antibody; HBs Ab: hepatitis B surface antibody; HBs Ag: hepatitis B surface antigen; HBV: hepatitis B virus; hCG: human chorionic gonadotropin; HCV: hepatitis C virus; HCV Ab: hepatitis C virus antibody; HIV: human immunodeficiency virus; IEC: Independent Ethics Committee; IRB: Institutional Review Board; RBC: red blood cells; RNA: ribonucleic acid; SAE: serious adverse event; SGOT: serum glutamic-oxaloacetic transaminase; SGPT: serum glutamic-pyruvic transaminase; TB: tuberculosis; ULN: upper limit of normal; WBC: white blood cells; WOCBP: woman of childbearing potential.

Investigators must document their review of each laboratory safety report.

Laboratory/analyte results that could unblind the study will not be reported to investigative sites or other blinded personnel until the study has been unblinded.

10.3 APPENDIX 3: ADVERSE EVENTS: DEFINITIONS AND PROCEDURES FOR RECORDING, EVALUATING, FOLLOW-UP, AND REPORTING

DEFINITION OF AE

AE definition

- An AE is any untoward medical occurrence in a patient or clinical study participant, temporally associated with the use of study intervention, whether or not considered related to the study intervention.

  NOTE: An AE can therefore be any unfavorable and unintended sign (including an abnormal laboratory finding), symptom, or disease (new or exacerbated) temporally associated with the use of study intervention.

Events meeting the AE definition

- Any abnormal laboratory test results (hematology, clinical chemistry, or urinalysis) or other safety assessments (eg, ECG, radiological scans, vital signs measurements), including those that worsen from baseline, considered clinically significant in the medical and scientific judgment of the Investigator (ie, not related to progression of underlying disease).
- Exacerbation of a chronic or intermittent pre-existing condition including either an increase in frequency and/or intensity of the condition.
- New conditions detected or diagnosed after study intervention administration even though it may have been present before the start of the study.
- Signs, symptoms, or the clinical sequelae of a suspected drug-drug interaction.
- Signs, symptoms, or the clinical sequelae of a suspected overdose of either study intervention or a concomitant medication.
- “Lack of efficacy” or “failure of expected pharmacological action” per se will not be reported as an AE or SAE. Such instances will be captured in the efficacy assessments. However, the signs, symptoms, and/or clinical sequelae resulting from lack of efficacy will be reported as AE or SAE if they fulfil the definition of an AE or SAE.

Events NOT meeting the AE definition

- Any clinically significant abnormal laboratory findings or other abnormal safety assessments which are associated with the underlying disease, unless judged by the Investigator to be more severe than expected for the participant’s condition.
- The disease/disorder being studied or expected progression, signs, or symptoms of the disease/disorder being studied, unless more severe than expected for the participant’s condition.
- Medical or surgical procedure (eg, endoscopy, appendectomy): the condition that leads to the procedure is the AE.
- Situations in which an untoward medical occurrence did not occur (social and/or convenience admission to a hospital).
- Anticipated day-to-day fluctuations of pre-existing disease(s) or condition(s) present or detected at the start of the study that do not worsen.

DEFINITION OF SAE

If an event is not an AE per definition above, then it cannot be an SAE even if serious conditions are met (eg, hospitalization for signs/symptoms of the disease under study, death due to progression of disease).

A SAE is defined as any untoward medical occurrence that, at any dose:

a) **Results in death**

b) **Is life-threatening**

The term "life-threatening" in the definition of "serious" refers to an event in which the participant was at risk of death at the time of the event. It does not refer to an event, which hypothetically might have caused death, if it were more severe.

c) **Requires inpatient hospitalization or prolongation of existing hospitalization**

In general, hospitalization signifies that the participant has been detained (usually involving at least an overnight stay) at the hospital or emergency ward for observation and/or treatment that would not have been appropriate in the physician's office or outpatient setting. Complications that occur during hospitalization are AEs. If a complication prolongs hospitalization or fulfills any other serious criteria, the event is serious. When in doubt as to whether "hospitalization" occurred or was necessary, the AE should be considered serious.

Hospitalization for elective treatment of a pre-existing condition that did not worsen from baseline is not considered an AE.

d) **Results in persistent disability/incapacity**

- The term disability means a substantial disruption of a person's ability to conduct normal life functions.
- This definition is not intended to include experiences of relatively minor medical significance such as uncomplicated headache, nausea, vomiting, diarrhea, influenza, and accidental trauma (eg, sprained ankle) which may interfere with or prevent everyday life functions but do not constitute a substantial disruption.

e) **Is a congenital anomaly/birth defect**

f) **Other situations:**

- Medical or scientific judgment should be exercised in deciding whether SAE reporting is appropriate in other situations such as important medical events that may not be immediately life-threatening or result in death or hospitalization but may jeopardize the participant or may require medical or surgical intervention to prevent one of the other outcomes listed in the above definition. These events should usually be considered serious.
- Examples of such events include invasive or malignant cancers, intensive treatment in an emergency room or at home for allergic bronchospasm, blood dyscrasias or convulsions that do not result in hospitalization, or development of drug dependency or drug abuse.

RECORDING AND FOLLOW-UP OF AE AND/OR SAE

AE and SAE recording

- When an AE/SAE occurs, it is the responsibility of the Investigator to review all documentation (eg, hospital progress notes, laboratory reports, and diagnostics reports) related to the event.
- The Investigator will then record all relevant AE/SAE information in the CRF.
- It is **not** acceptable for the Investigator to send photocopies of the participant's medical records to the Sponsor's representative in lieu of completion of the AE/SAE CRF page.
- There may be instances when copies of medical records for certain cases are requested by the Sponsor's representative. In such case, care should be taken to ensure that the patient's identity is protected and the patient's identifiers in the study are properly mentioned on any copy of a source document provided to the Sponsor.
- The Investigator will attempt to establish a diagnosis of the event based on signs, symptoms, and/or other clinical information. Whenever possible, the diagnosis (not the individual signs/symptoms) will be documented as the AE/SAE.

Assessment of intensity

The Investigator will make an assessment of intensity for each AE and SAE reported during the study and assign it to 1 of the following categories:

- Mild: An event that is easily tolerated by the participant, causing minimal discomfort and not interfering with everyday activities.
- Moderate: An event that causes sufficient discomfort and interferes with normal everyday activities.
- Severe: An event that prevents normal everyday activities. An AE that is assessed as severe should not be confused with a SAE. Severe is a category utilized for rating the intensity of an event; and both AEs and SAEs can be assessed as severe.

  An event is defined as "serious" when it meets at least 1 of the predefined outcomes as described in the definition of an SAE, NOT when it is rated as severe.

Assessment of causality

- The Investigator is obligated to assess the relationship between study intervention and each occurrence of each AE/SAE.
- A "reasonable possibility" of a relationship conveys that there are facts, evidence, and/or arguments to suggest a causal relationship, rather than that a relationship cannot be ruled out.
- The Investigator will use clinical judgment to determine whether or not the relationship is causal.

- Alternative causes, such as underlying disease(s), concomitant therapy, and other risk factors, as well as the temporal relationship of the event to study intervention administration will be considered and investigated.
- The Investigator will also consult the IB and/or Product Information, for marketed products, in his/her assessment.
- For each AE/SAE, the Investigator **must** document in the medical notes that he/she has reviewed the AE/SAE and has provided an assessment of causality.
- There may be situations in which an SAE has occurred and the Investigator has minimal information to include in the initial report to the Sponsor's representative. However, **it is very important that the Investigator always make an assessment of causality for every event before the initial transmission of the SAE data to the Sponsor's representative.**
- The Investigator may change his/her opinion of causality in light of follow-up information and send a SAE follow-up report with the updated causality assessment.
- The causality assessment is one of the criteria used when determining regulatory reporting requirements.

Follow-up of AEs and SAEs

- The Investigator is obligated to perform or arrange for the conduct of supplemental measurements and/or evaluations as medically indicated or as requested by the Sponsor's representative to elucidate the nature and/or causality of the AE or SAE as fully as possible. This may include additional laboratory tests or investigations, histopathological examinations, or consultation with other health care professionals.
- If a participant dies during participation in the study or during a recognized follow-up period, the Investigator will provide the Sponsor's representative with a copy of any post-mortem findings including histopathology.
- New or updated information will be recorded in the originally completed CRF.
- The Investigator will submit any updated SAE data to the Sponsor within 24 hours of receipt of the information.

REPORTING OF SAES

SAE reporting to the Sponsor via an electronic data collection tool

- The primary mechanism for reporting an SAE to the Sponsor's representative will be the electronic data collection tool.
- If the electronic system is unavailable, then the site will use the paper SAE data collection tool (see next section) in order to report the event within 24 hours.
- The site will enter the SAE data into the electronic system as soon as it becomes available.
- After the study is completed at a given site, the electronic data collection tool will be taken off-line to prevent the entry of new data or changes to existing data.

- If a site receives a report of a new SAE from a study participant or receives updated data on a previously reported SAE after the electronic data collection tool has been taken off-line, then the site can report this information on a paper SAE form (see next section) or to the Sponsor's representative by telephone.
- Contacts for SAE reporting can be found in the protocol.

SAE reporting to the Sponsor's representative via paper CRF

- Facsimile transmission of the SAE paper CRF is the preferred method to transmit this information to the Sponsor's representative.
- In rare circumstances and in the absence of facsimile equipment, notification by telephone is acceptable with a copy of the SAE data collection tool sent by overnight mail or courier service.
- Initial notification via telephone does not replace the need for the Investigator to complete and sign the SAE CRF pages within the designated reporting time frames.
- Contacts for SAE reporting can be found in the protocol.

10.4 APPENDIX 4: CONTRACEPTIVE GUIDANCE AND COLLECTION OF PREGNANCY INFORMATION

DEFINITIONS:

Woman of childbearing potential (WOCBP)

A woman is considered fertile following menarche and until becoming post-menopausal unless permanently sterile (see below).

If fertility is unclear (eg, amenorrhea in adolescents or athletes) and a menstrual cycle cannot be confirmed before first dose of study intervention, additional evaluation should be considered.

Women in the following categories are not considered WOCBP

1. Premenarchal
2. Premenopausal female with 1 of the following:
   - Documented hysterectomy
   - Documented bilateral salpingectomy
   - Documented bilateral oophorectomy

     For individuals with permanent infertility due to an alternate medical cause other than the above, (eg, mullerian agenesis, androgen insensitivity), Investigator discretion should be applied to determining study entry.

   Note: Documentation can come from the site personnel's: review of the participant's medical records, medical examination, or medical history interview.

3. Postmenopausal female
    - A postmenopausal state is defined as no menses for 12 consecutive months without an alternative medical cause.
    - Females on hormone replacement therapy (HRT) and whose menopausal status is in doubt will be required to use one of the non-estrogen hormonal highly effective contraception methods if they wish to continue their HRT during the study.

CONTRACEPTION GUIDANCE

Female participants of childbearing potential are eligible to participate if they agree to use a highly effective method of contraception consistently and correctly as described below.

**Contraceptives[a] allowed during the study include:**
**Highly effective methods[b] that have low user dependency**<br>*Failure rate of <1% per year when used consistently and correctly.*
• Implantable progestogen-only hormone contraception associated with inhibition of ovulation[b]<br>- Intrauterine device (IUD)<br>- Intrauterine hormone-releasing system (IUS)[b]<br>• Bilateral tubal occlusion
Vasectomized partner<br>*(Vasectomized partner is a highly effective contraceptive method provided that the partner is the sole sexual partner of the WOCBP and the absence of sperm has been confirmed. If not, an additional highly effective method of contraception should be used. Spermatogenesis cycle is approximately 90 days.)*
**Highly effective methods[b] that are user dependent**<br>*Failure rate of <1% per year when used consistently and correctly.*
• Combined (estrogen- and progestogen-containing) hormonal contraception associated with inhibition of ovulation[c]<br>- oral<br>- intravaginal<br>- transdermal<br>- injectable<br>• Progestogen-only hormone contraception associated with inhibition of ovulation[c]<br>- oral<br>- injectable
Sexual abstinence<br>*Sexual abstinence is considered a highly effective method only if defined as refraining from heterosexual intercourse during the entire period of risk associated with the study intervention. The reliability of sexual abstinence needs to be evaluated in relation to the duration of the study and the preferred and usual lifestyle of the participant.)*

a Contraceptive use by men or women should be consistent with local regulations regarding the use of contraceptive methods for those participating in clinical studies.

b Failure rate of <1% per year when used consistently and correctly. Typical use failure rates differ from those when used consistently and correctly.

c If locally required, in accordance with Clinical Trial Facilitation Group (CTFG) guidelines, acceptable contraceptive methods are limited to those which inhibit ovulation as the primary mode of action.

WOCBP: woman of childbearing potential.

COLLECTION OF PREGNANCY INFORMATION:

Male participants with partners who become pregnant

- The Investigator will attempt to collect pregnancy information on any male participant's female partner who becomes pregnant while the male participant is in this study. This applies only to male participants who receive dupilumab.
- After obtaining the necessary signed informed consent from the pregnant female partner directly, the Investigator will record pregnancy information on the appropriate form and submit it to the Sponsor within 24 hours of learning of the partner's pregnancy. The female partner will also be followed to determine the outcome of the pregnancy. Information on the status of the mother and child will be forwarded to the Sponsor. Generally, the follow-up will be no longer than 6 to 8 weeks following the estimated delivery date. Any termination of the pregnancy will be reported regardless of fetal status (presence or absence of anomalies) or indication for the procedure.

Female participants who become pregnant

- The Investigator will collect pregnancy information on any female participant who becomes pregnant while participating in this study. Information will be recorded on the appropriate form and submitted to the Sponsor within 24 hours of learning of a participant's pregnancy. The participant will be followed to determine the outcome of the pregnancy. The Investigator will collect follow-up information on the participant and the neonate and the information will be forwarded to the Sponsor. Generally, follow-up will not be required for longer than 6 to 8 weeks beyond the estimated delivery date. Any termination of pregnancy will be reported, regardless of fetal status (presence or absence of anomalies) or indication for the procedure.
- Any pregnancy complication or elective termination of a pregnancy will be reported as an AE or SAE. A spontaneous abortion is always considered to be an SAE and will be reported as such. Any post-study pregnancy related SAE considered reasonably related to the study intervention by the Investigator will be reported to the Sponsor as described in Section 8.3.4 of the protocol. While the Investigator is not obligated to actively seek this information in former study participants, he or she may learn of an SAE through spontaneous reporting.
- Any female participant who becomes pregnant while participating in the study will discontinue study intervention.

10.5 APPENDIX 5: GENETICS

Use/Analysis of DNA and RNA

- Genetic variation may impact a participant's response to study intervention, susceptibility to, and severity and progression of disease. Variable response to study intervention may be due to genetic determinants that impact drug absorption, distribution, metabolism, and excretion; mechanism of action of the drug; safety, disease etiology; and/or molecular subtype of the disease being treated. Therefore, where local regulations and IRB/IEC allow, a blood sample will be collected for DNA analysis from consenting participants.

- DNA and RNA samples will be used for research related to dupilumab or PN and related diseases. They may also be used to develop tests/assays including diagnostic tests related to dupilumab or related drugs and atopic/allergic diseases. Genetic research may consist of the analysis of one or more candidate genes or the analysis of genetic markers throughout the genome including whole-exome sequencing, whole-genome sequencing, and DNA copy number variation. Transcriptome sequencing (or other methods for quantitating RNA expression) may also be performed.
- The samples may be analyzed as part of a multi-study assessment of genetic factors involved in the response to dupilumab or study interventions of this class to understand study disease or related conditions.
- The results of genetic analyses will not be reported in the CSR.
- The Sponsor will store the DNA samples in a secure storage space with adequate measures to protect confidentiality.
- The samples will be retained no longer than 15 years or other period as per local requirements.

10.6 APPENDIX 6: LIVER AND OTHER SAFETY: SUGGESTED ACTIONS AND FOLLOW-UP ASSESSMENTS

INCREASE IN ALT

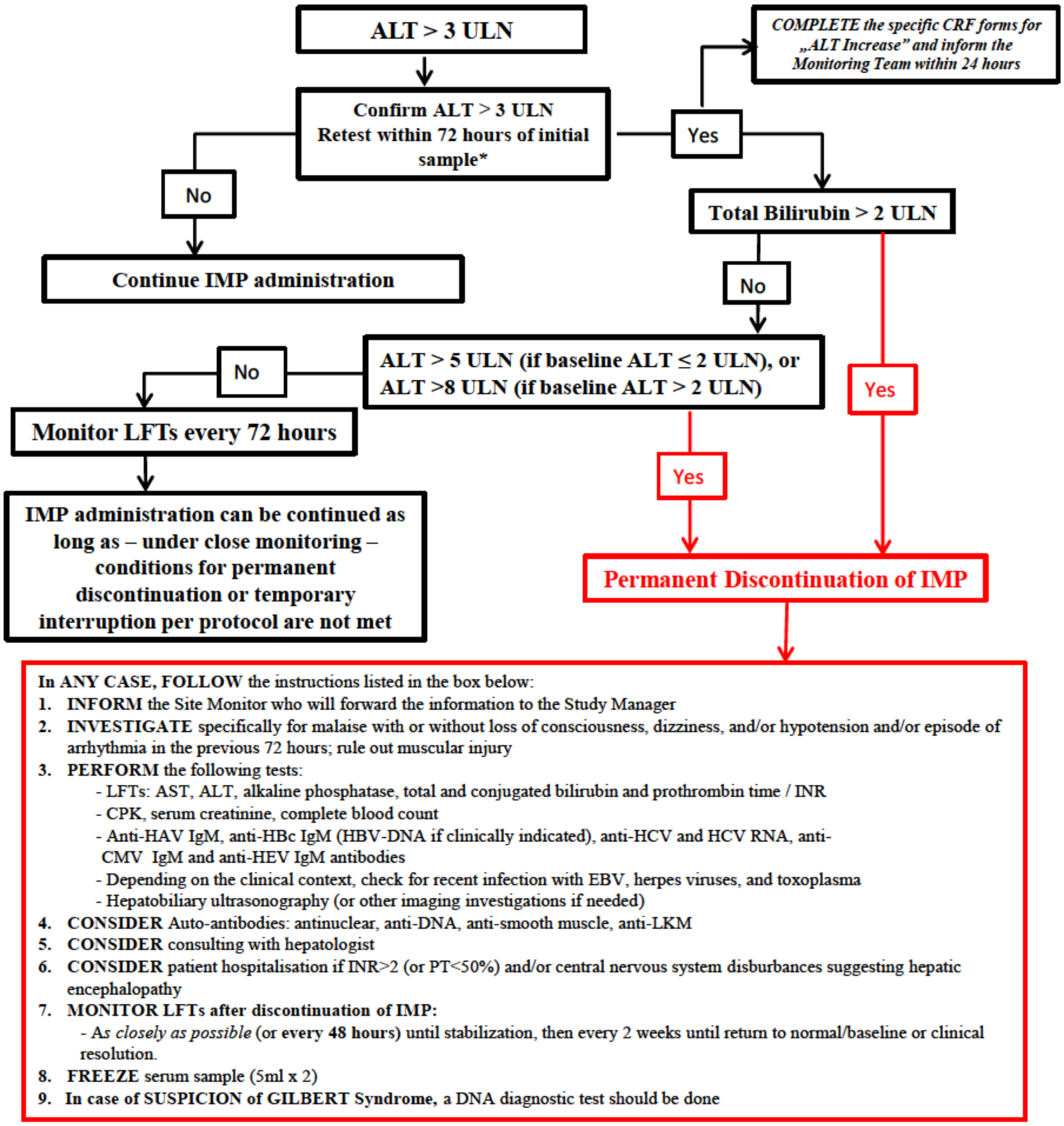

In **ANY CASE, FOLLOW** the instructions listed in the box below:

1. **INFORM** the Site Monitor who will forward the information to the Study Manager
2. **INVESTIGATE** specifically for malaise with or without loss of consciousness, dizziness, and/or hypotension and/or episode of arrhythmia in the previous 72 hours; rule out muscular injury
3. **PERFORM** the following tests:
   - LFTs: AST, ALT, alkaline phosphatase, total and conjugated bilirubin and prothrombin time / INR
   - CPK, serum creatinine, complete blood count
   - Anti-HAV IgM, anti-HBc IgM (HBV-DNA if clinically indicated), anti-HCV and HCV RNA, anti-CMV IgM and anti-HEV IgM antibodies
   - Depending on the clinical context, check for recent infection with EBV, herpes viruses, and toxoplasma
   - Hepatobiliary ultrasonography (or other imaging investigations if needed)
4. **CONSIDER** Auto-antibodies: antinuclear, anti-DNA, anti-smooth muscle, anti-LKM
5. **CONSIDER** consulting with hepatologist
6. **CONSIDER** patient hospitalisation if INR>2 (or PT<50%) and/or central nervous system disburbances suggesting hepatic encephalopathy
7. **MONITOR LFTs after discontinuation of IMP:**
   - *As closely as possible* (or **every 48 hours**) until stabilization, then every 2 weeks until return to normal/baseline or clinical resolution.
8. **FREEZE** serum sample (5ml x 2)
9. **In case of SUSPICION of GILBERT Syndrome,** a DNA diagnostic test should be done

*If unable to retest in 72 hours, use original lab results to decide on further reporting/monitoring/discontinuation.

Note:

"Baseline" refers to ALT sampled at baseline visit; or if baseline value unavailable, to the latest ALT sampled before the baseline visit. The algorithm does not apply to the instances of increase in ALT during screening.

See Section 10.3 for guidance on safety reporting.

Normalization is defined as ≤ULN or baseline value, if baseline value is >ULN.

INCREASE IN SERUM CREATININE in patients with normal baseline (creatininemia between 45 µmol/L and 84 µmol/L)

Rapid increase in serum creatinine >1.7 ULN or rapid decrease in creatinine clearance >50%

↓

Repeat the test to confirm the increase / decrease

↓

Can be rapidly reversed:
- By volume repletion
- Or relief of urinary tract obstruction (according to etiology)

↓

1. Investigational medicinal product may be continued
2. **MONITOR** serum creatinine until return to baseline level

Cannot be rapidly reversed:
- Occurrence/aggravation of life threatening symptoms of ARF including: anemia, hyperkalemia, hyperuricemia, metabolic acidosis, cardiac insufficiency, pulmonary edema, arrhythmia, DIC, etc.
- And/or predominant elimination of investigational medicinal product by renal route

↓

1. **INFORM** the local monitor
2. **DISCONTINUE** investigational medicinal product administration
3. **HOSPITALIZATION** should be considered and seek for nephrologic advice
4. **PERFORM** the following examinations **locally**:
   - BP, HR, hydration status, ECG
   - Blood count
   - Liver function tests + CPK
   - Biochemistry, including urea
   - Urinalysis
   - Renal ultrasound
5. **COLLECT/STORE** one sample following handling procedures described in PK sections **(for studies with PK sampling)**
6. **MONITOR** renal function until return to baseline level (every day at the beginning, then every week)

Increase in serum creatinine is to be recorded as an AE only if at least 1 of the criteria listed in the general guidelines for reporting AEs in Section 10.3 is met.

INCREASE IN CPK OF NON-CARDIAC ORIGIN AND NOT RELATED TO INTENSIVE PHYSICAL ACTIVITY

If Increase in CPK > 10 ULN

↓

Repeat immediately locally the count.
If confirmed, inform the local monitor and

↓

INVESTIGATE for the origin:
- **INTERVIEW** the patient about a recent intensive muscular effort, trauma, convulsions, electrical injury, or stress to the skeletal muscle, multiple intramuscular injections, recent surgery, concomitant medications, consumption of alcohol, morphine, cocaine…
- **PERFORM locally**:
    - ECG, CK-MB , Troponin if not previously done
    - CK-MM
    - Creatinine
    - Iono (K+)
    - Transaminases + Total and conjugated bilirubin
    - And any other parameters considered as relevant per medical judgement (e.g. Ca^{2+}, Myoglobin)
- **COLLECT/STORE** one sample following handling procedures described in PK sections **(for studies with PK sampling)**
- **SEARCH** for the cause

↓

1. Hospitalization should be considered depending on clinical context and profile of the patient
2. Consider to discontinue investigational medicinal product administration
3. Monitor biological parameters as appropriate within the next days/weeks/months until return to baseline

Increase in CPK is to be recorded as an AE only if at least 1 of the criteria in the general guidelines for reporting AEs in Section 10.3 is met.

10.7 APPENDIX 7: CLINICIANS-REPORTED OUTCOMES AND PATIENT-REPORTED OUTCOMES

10.7.1 Worst itch numeric rating scale (WI-NRS)

WWU MÜNSTER

UKM Universitätsklinikum Münster

Worst-itch NRS

[redacted]

UKM: AöR, Prof. Dr. Martin Schulze Schwienhorst (Aufsichtsratsvorsitzender),
Univ.-Prof. Dr. med. Dr. phil. Robert Nitsch (Vorstandsvorsitzender, Ärztlicher Direktor),
Dr. rer. pol. Christoph Hoppenheit (stellv. Vorstandsvorsitzender, Kaufmännischer Direktor),
Univ.-Prof. Dr. med. Mathias Herrmann (Dekan), Thomas van den Hooven (Pflegedirektor),
Univ.-Prof. Dr. med. Claudia Rössig (stellv. Ärztliche Direktorin)

KTQ-Zertifikat

10.7.2 Investigator's global assessment of prurigo nodularis (IGA PN)

[redacted]

Copyright @ Center for Chronic Pruritus, University Hospital Münster Under license. For permission to use refer to: KCPadministration@ukmuenster.de

10.7.3 Prurigo activity score (PAS)

[redacted]

[redacted]

Copyright @ Center for Chronic Pruritus, University Hospital Münster Under license. For permission to use refer to: KCPadministration@ukmuenster.de

10.7.4 Dermatology life quality index (DLQI)

[redacted]

10.7.5 Hospital anxiety and depression scale (HADS)

[redacted]

10.7.6 EQ-5D-5L

[redacted]

10.7.7 Pain numeric rating scale

Worst Skin Pain Numerical Rating Scale (Generic)

How would you rate your skin pain at its **worst** in the past 24 hours?

0	1	2	3	4	5	6	7	8	6	10
No pain										Worst pain possible

10.7.8 Sleep numeric rating scale

Sleep Quality Numerical Rating Scale (Generic)

Instructions:

Please complete the following questions upon awakening for the day.

Select the number that best describes the quality of your sleep last night.

0	1	2	3	4	5	6	7	8	9	10
Worst possible sleep										Best possible sleep

10.7.9 Patient Global Impression of Change of disease (PGIC)

Patient Global Impression of Change (PGIC) of PN Disease

Please choose the response below that best describes the overall change in your prurigo nodularis since you started taking the study medication.

☐ Very much better

☐ Moderately better

☐ A little better

☐ No change

☐ A little worse

☐ Moderately worse

☐ Very much worse

10.7.10 Patient Global Impression of Severity (PGIS)

Patient Global Impression of Severity (PGIS) of PN Disease

Please choose the response below that best describes the severity of your prurigo nodularis over the past week.

☐ None

☐ Mild

☐ Moderate

☐ Severe

10.7.11 Missed school/work days

Patients with Prurigo Nodularis *(PN)*

Missed School/Work days
BASELINE Visit

Site Number: __________ Patient Study Number: ___________________________

Visit Date (dd/mmm/yyyy): ______/______/______Time of Completion: ___:_____am/pm

Employment status

1. Have you been working?

   □ Yes – I am working full time (≥4 days/week)
   □ Yes – I am working part time (<4 days/week)
   □ No – I am not working (unemployed, retired)

For those working (full time or part time):

2. How many days have you missed from work because of your Prurigo Nodularis in the last 4 weeks prior to the visit today?

   Please do not include time you missed from work for taking part in this research.

   Specify: __ __ __ Days

Student status

1. Have you been in school?

   □ Yes – I am in school full time (≥4 days/week)
   □ Yes – I am in school part time (<4 days/week)
   □ No – I am not in school

For those in school (full time or part time):

2. How many days have you missed from school because of your Prurigo Nodularis in the last 4 weeks prior to the visit today?

   Please do not include time you missed from school for taking part in this research.

   Specify: __ __ __ Days

NOTES:

Please make sure the instructions are read to the patient BEFORE they start the questionnaire.

Assessment periods (i.e., time interval from the date of last assessment through the current date) must be continuous. Leave no gaps. Cumulatively, these periods must cover the entire study duration.

Avoid duplicate reporting. If the patient missed school/work because of his/her PN on the day of

assessment, this day should be recorded only once.

Record only days on which patient's PN prevented him/her to go to school/work. Otherwise, do not record the days on which the patient missed school/work only to participate in study visits.

Print Name (First/Last) of Investigator Completing Assessment	Signature	Date

R668-XXX-Missed School/work Days Version 1.0 BASELINE; *date*

Page 1 of 1

Patients with *Prurigo Nodularis (PN)*

Missed School/work days
POST Baseline Visits

Site Number: _____________Patient Study Number: _____________

Visit Date (dd/mmm/yyyy): ____/_____/_____Time of Completion: ___:_____am/pm

Employment status

1. Have you been working since your last assessment?

 ◻ Yes – I am working full time (≥4 days/week)
 ◻ Yes – I am working part time (<4 days/week)
 ◻ No – I am not working (unemployed, retired)

For those working (full time or part time):

2. How many days have you missed from work because of your Prurigo Nodularis since the day of your last assessment, including today?

 Please do not include time you missed from work for taking part in this research.

 Specify: __ __ __ Days

Student status

1. Have you been in school since your last assessment?

 ◻ Yes – I am in school full time (≥4 days/week)
 ◻ Yes – I am in school part time (<4 days/week)
 ◻ No – I am not in school

For those in school (full time or part time):

2. How many days have you missed from school because of your Prurigo Nodularis since the day of your last assessment, including today?

 Please do not include time you missed from school for taking part in this research.

 Specify: __ __ __ Days

NOTES:

Please make sure the instructions are read to the patient BEFORE they start the questionnaire.

Assessment periods (i.e., time interval from the date of last assessment through the current date) must be continuous. Leave no gaps. Cumulatively, these periods must cover the entire study duration.

Avoid duplicate reporting. If the patient missed school/work because of his/her PN on the day of assessment, this day should be recorded only once.

Record only days on which patient's PN prevented him/her to go to school/work. Otherwise, do not record the days on which the patient missed school/work only to participate in study visits.

**Print Name (First/Last) of Investigator Completing Assessment**	**Signature**	**Date**

R668-XXX Missed School/work Days Version 2.0; date

Page 1 of 1

10.8 APPENDIX 8: DEFINITION OF ANAPHYLAXIS

"Anaphylaxis is a serious allergic reaction that is rapid in onset and may cause death (31)."

Clinical criteria for diagnosing anaphylaxis

Anaphylaxis is highly likely when any one of the following 3 criteria are fulfilled:

1. Acute onset of an illness (minutes to several hours) with involvement of the skin, mucosal tissue, or both (eg, generalized hives, pruritus or flushing, swollen lips-tongue-uvula)
   AND AT LEAST ONE OF THE FOLLOWING
   a. Respiratory compromise (eg, dyspnea, wheeze-bronchospasm, stridor, reduced PEF, hypoxemia)
   b. Reduced BP or associated symptoms of end-organ dysfunction (eg, hypotonia [collapse], syncope, incontinence)
2. Two or more of the following that occur rapidly after exposure *to a likely allergen for that patient* (minutes to several hours):
   a. Involvement of the skin-mucosal tissue (eg, generalized hives, itch-flush, swollen lips-tongue-uvula)
   b. Respiratory compromise (eg, dyspnea, wheeze-bronchospasm, stridor, reduced PEF, hypoxemia)
   c. Reduced BP or associated symptoms (eg, hypotonia [collapse], syncope, incontinence)
   d. Persistent gastrointestinal symptoms (eg, crampy abdominal pain, vomiting)
3. Reduced BP after exposure to *known allergen for that patient* (minutes to several hours):
   a. Infants and children: low systolic BP (age specific) or greater than 30% decrease in systolic BP*
   b. Adults: systolic BP of less than 90 mm Hg or greater than 30% decrease from that person's baseline

PEF, Peak expiratory flow; *BP*, blood pressure.
*Low systolic blood pressure for children is defined as less than 70 mm Hg from 1 month to 1 year, less than (70 mm Hg + [2 × age]) from 1 to 10 years, and less than 90 mm Hg from 11 to 17 years.

10.9 APPENDIX 9: LIST OF OPPORTUNISTIC INFECTIONS

- Aspergillosis
- Blastomyces dermatitidis (endemic in the south-eastern and south-central states US, along Mississippi and Ohio Rivers)
- Candidiasis - only systemic or extensive mucosal or cutaneous candidiasis
- Coccidioides immitis (endemic south-western US and Central and South America)
- Cryptococcus
- Cytomegalovirus
- Herpes Simplex (disseminated)
- Herpes Zoster (disseminated; ophthalmic; involvement of 2 or more dermatomes)
- Histoplasmosis (pulmonary or disseminated; most common tropical areas Tennessee-Ohio-Mississippi river basins)
- Listeriosis
- Mycobacterium avium
- Nontuberculosis mycobacteria
- Pneumocystis pneumonia

This list is indicative and not exhaustive.

10.10 APPENDIX 10: COUNTRY-SPECIFIC REQUIREMENTS

China:

1. Urine pregnancy test can be done at local lab according to the local requirement at Week 16, Week 20, Week 28, and Week 32.
2. Scheduled PK and ADA samples will not be collected.

10.11 APPENDIX 11: ABBREVIATIONS

ACE:	angiotensin converting enzyme
AD:	atopic dermatitis
ADA:	antidrug antibody
ADR:	adverse drug reaction
AE:	adverse event
AESI:	adverse event of special interest
ALT:	alanine aminotransferase
ANCOVA:	analysis of covariance
AST:	aspartate aminotransferase
BUN:	blood urea nitrogen
CFR:	Code of Federal Regulations
CIOMS:	Council for International Organizations of Medical Sciences
ClinRO:	clinician-reported outcome
CMH:	Cochran-Mantel-Haenszel
CONSORT:	Consolidated Standards of Reporting Trials
COVID-19:	coronavirus disease 2019
CRF:	case report form
CRSwNP:	chronic rhinosinusitis with nasal polyposis
CSR:	clinical study report
DBP:	diastolic blood pressure
DLQI:	dermatology life quality index
DMC:	data monitoring committee
DNA:	deoxyribonucleic acid
DTP:	direct-to-patient
EADV:	European Academy of Dermatology and Venereology
EASI:	eczema area and severity index
ECG:	electrocardiogram
eCRF:	electronic case report form
ELISA:	enzyme-linked immunosorbent assay
EMA:	European Medicines Agency
EoE:	eosinophilic esophagitis
EOS:	end of study
EOT:	end of treatment
EQ-5D:	Euroqol 5 dimensions
EQ-5D-5L:	Euroqol 5 dimensions 5 levels

EU:	European Union
FDA:	Food and Drug Administration
GCP:	Good Clinical Practice
HADS:	hospital anxiety and depression scale
HBc Ab:	hepatitis B core antibody
HBs Ab:	hepatitis B surface antibody
HBs Ag:	hepatitis B surface antigen
HBV:	hepatitis B virus
hCG:	human chorionic gonadotropin
HCV:	hepatitis C virus
HCV Ab:	hepatitis C virus antibody
HIV:	human immunodeficiency virus
HRQoL:	health-related quality of life
HRT:	hormone replacement therapy
IB:	investigator's brochure
ICF:	informed consent form
ICH:	International Council for Harmonisation
IEC:	Independent Ethics Committee
Ig:	immunoglobulin
IGA:	investigator's global assessment
IGA PN:	investigator's global assessment for prurigo nodularis
IGA PN-A:	IGA PN-Activity
IGA PN-S:	IGA PN-Stage
IL:	interleukin
IMP:	investigational medicinal product
IRB:	Institutional Review Board
IRT:	interactive response technology
ITT:	intent to treat
IVRS:	interactive voice response system
IWRS:	interactive web response system
LS:	least squares
NIMP:	noninvestigational medicinal product
NRS:	numeric rating scale
PAS:	prurigo activity score
PD:	pharmacodynamic
PGA:	physician global assessment
PGIC:	Patient Global Impression of Change
PGIS:	Patient Global Impression of Severity
PK:	pharmacokinetic
PN:	prurigo nodularis
PRO:	patient reported outcome
PT:	preferred term
Q2W:	every 2 weeks
QTcF:	QT interval corrected using Fridericia's formula
RBC:	red blood cells
RNA:	ribonucleic acid

SAE:	serious adverse event
SAP:	statistical analysis plan
SBP:	systolic blood pressure
SC:	subcutaneous
SCORAD:	scoring atopic dermatitis
SGOT:	serum glutamic-oxaloacetic transaminase
SGPT:	serum glutamic-pyruvic transaminase
SNRI:	serotonin and norepinephrin reuptake inhibitor
SoA:	schedule of activities
SOC:	system organ class
SSRI:	selective serotonin reuptake inhibitor
SUSAR:	suspected unexpected serious adverse reaction
TB:	tuberculosis
TCI:	topical calcineurin inhibitors
TCS:	topical corticosteroids
TEAE:	treatment-emergent adverse event
ULN:	upper limit of normal
US:	United States
UVB:	ultraviolet B
VAS:	visual analog scale
WBC:	white blood cells
WI-NRS:	worst-itch numeric rating scale
WOCBP:	woman of childbearing potential
WOCF:	worst observation carried forward

10.12 APPENDIX 12: PROTOCOL AMENDMENT HISTORY

The Protocol Amendment Summary of Changes Table for the current amendment is located directly before the Table of Contents (TOC).

10.12.1 Amended protocol 01 (20 May 2020)

This amended protocol (amendment 01) is considered to be substantial based on the criteria set forth in Article 10(a) of Directive 2001/20/EC of the European Parliament and the Council of the European Union.

Overall rationale for the Amendment

The proportion of participants with Investigator's Global Assessment 0 or 1 score for PN-Stage (IGA PN-S) at Week 24 has been indicated as an additional key secondary endpoint. This is to include a lesion-related key secondary endpoint based on the recommendations from health authorities.

Protocol amendment summary of changes table

Section # and Name	Description of Change	Brief Rationale
1.1 Synopsis: Objectives and endpoints<br>3 Objectives and Endpoints<br>3.1 Appropriateness of measurements<br>9.4.1 Efficacy analyses	To add "proportion of participants with Investigator's Global Assessment 0 or 1 score for PN-Stage (IGA PN-S) at Week 24" as another key secondary endpoint	To include a lesion-related key secondary endpoint according to the health authority's recommendations
1.3 Schedule of activities	To correct rescue medication use start date to Day 1 (Visit 2)	To correct an error on the rescue medication use
3 Objectives and Endpoints	To remove the endpoint "Change from baseline in PAS total score at Week 4, Week 8, Week 12, and Week 24" in the exploratory endpoint and modify the exploratory endpoint regarding the healed lesions from PAS questionnaire analysis	To clarify the analysis on efficacy evaluation of dupilumab on skin lesions using a modified prurigo activity score (PAS) 5-item questionnaire
1.1 Synopsis (Objectives and endpoints)<br>3 Objectives and Endpoints<br>9.4.1 Efficacy analyses	To break out the secondary endpoints with multiple measuring timepoints into individual endpoints.	To clearly define the timepoints of each endpoint according to health authority's recommendation
5.1 Inclusion Criteria	Change of I05 "medium-to-high potency TCS" to "medium-to-superpotent TCS"	To enroll patients on medium to superpotent topical corticosteroids (TCS) according to health authority's recommendations
1.1 Synopsis: Noninvestigational medicinal products<br>5.1 Inclusion Criteria<br>6.1.1 Noninvestigational medicinal products	Change of topical emollient (moisturizer) application in Synopsis and I06 from "once or twice daily for at least the 7 consecutive days immediately before Day 1" to "once or twice daily for at least 5 out of the 7 consecutive days immediately before Day 1"	To allow some flexibility in adherence to the moisturizer usage by the patient during screening
5.2 Exclusion Criteria	To update E04 to "within 6 months before the screening visit, or documented diagnosis of moderate to severe AD from screening visit to randomization visit".	To specify the exclusion criteria of patients with documented atopic dermatitis severity moderate to severe
5.2 Exclusion Criteria<br>1.3 Schedule of Activities<br>10.2 Appendix 2: Clinical laboratory tests	To update E05 "hemoglobin A1c ≥9%" to "hemoglobin A1c ≥9% according to the laboratory results within 3 months before screening visit"	To clarify the exclusion criteria for patients with diabetes history is according to the hemoglobin A1c results within 3 months before screening visit

Section # and Name	Description of Change	Brief Rationale
5.2 Exclusion Criteria	To update E10 from "within 2 weeks before the screening visit" to "within 2 weeks before screening visit and during the screening period"	To exclude the patients with active chronic or acute infection (except HIV infection) requiring treatment with systemic antibiotics, antivirals, antiprotozoals, or antifungals during screening period
5.2 Exclusion Criteria	To modify E18 to allow prurigo nodularis (PN) patients to use stable dose of some anti-depressant medicines for at least 3 months prior to screening	To enroll PN patients with stable dose of anti-depressant
5.2 Exclusion Criteria	To add menthol and polidocanol as one of the moisturizer additives in E20	To clarify that the patients who initiate treatment with moisturizers containing menthol and polidocanol will be excluded
5.2 Exclusion Criteria<br>6.1.1 Noninvestigational medicinal products	To add to E22 a stable regimen of TCS/TCI "maintain same medicine, same dose from 2 weeks prior to screening visit"	To clarify the definition of stable regimen of TCS/TCI
5.2 Exclusion Criteria	To add "treated in the past with dupilumab" in E25	To exclude the patients previously treated with dupilumab
5.2 Exclusion Criteria	To describe the participants with or without history of HIV infection in E26	To clarify the exclusion criteria about HIV infection and HIV viral load, and that the CD4+ test will only be done in the patient with existing HIV history
5.2 Exclusion Criteria<br>1.3 Schedule of Activities: footnote c<br>10.2 Appendix 2: Clinical laboratory tests: Table 9 footnote c	To clarify to E27 the positive HBc Ab and HCV Ab confirmation method	To clarify that the positive HBc Ab and HCV Ab will be confirmed by HBV DNA and HCV RNA, respectively
6.1.1 Noninvestigational medicinal products	To clarify the use of noninvestigational medicinal products	To prohibit topical medications that have anti-itch properties
6.5 Concomitant Therapy	To clarify the permitted and prohibited concomitant medications	To clarify the requirement of antihistamine used concomitantly, and antidepressant dose should also remain stable (can be reduced or discontinued if medically indicated), but should not be initiated or increased throughout the study
6.5.1 Rescue Medicine	To clarify the use of rescue medicine	To add criteria for rescue therapy
6.5.1 Rescue Medicine	To add that a pre-specified algorithm will be used to classify rescue and a blinded review of all post-baseline medications, based on medical judgment, will be performed to adjudicate rescue	To clarify the review of adjudicate rescue treatment

Section # and Name	Description of Change	Brief Rationale
7.1.1 Definitive discontinuation<br>7.1.2 Temporary discontinuation	To clarify the treatment discontinuation criteria regarding the missing doses	To clarify that patients with missing more than 2 consecutive IMP doses should be permanently withdrawn from the study treatment
8 Study assessments and procedures	The following wording was added: "In light of the public health emergency related to Coronavirus disease 2019 (COVID-19) (or in case of any other pandemic requiring public health emergency), the continuity of clinical study conduct and oversight may require implementation of temporary or alternative mechanisms eg, phone contact, virtual visits, online meetings, use of local clinic or laboratory locations, and home visits by skilled staff. Implementation of such mechanisms may differ country by country, depending on country regulations and local business continuity plans. Additionally, no waivers to deviate from protocol enrollment criteria due to COVID-19 (or any other pandemic) will be granted. All temporary mechanisms utilized, and deviations from planned study procedures are to be documented as being related to COVID-19 (or any other pandemic) and will remain in effect only for the duration of the public health emergency."	To describe alternative temporary mechanism that can be implemented in the study conduct in case of pandemic requiring public health emergency eg. COVID-19
8.3 Adverse events and serious adverse events	To update the adverse event of special interest (AESI) "any type of conjunctivitis or blepharitis (severe or serious)" to "any severe type of conjunctivitis or blepharitis"	To update the AESI list to harmonize with the most recent list
9.4.1 Efficacy analyses	To add the sensitivity analysis for secondary endpoints information, and to separate key secondary endpoints in a different row	To evaluate the robustness of the missing data imputation assumption by sensitivity analyses, and to clarify the endpoints analyses
9.4.1 Efficacy analyses<br>1.1 Synopsis (Statistical considerations)	To add the covariate "baseline anti-depressant use (yes or no)" to primary and secondary endpoint analyses	To adjust for potential impact of anti-depressant use on the treatment effect in primary and secondary efficacy analyses
9.4.3 Other analyses	The following wording was added: "Data collected regarding the impact of the COVID-19 or other pandemics, on the patients will be summarized (eg, discontinuation due to COVID-19). Any additional analyses and methods required to investigate the impact of COVID-19 or other pandemics requiring public health emergency on the efficacy (eg, missing data due to COVID-19) and safety will be detailed in the SAP".	To describe alternative temporary mechanism that can be implemented in the study conduct in case of pandemic requiring public health emergency eg, COVID-19
9.5.2 Unblinding plan	To update the unblinding plan to "Unblinding plan is not applicable for this study"	Unblinding plan is not applicable for this study.

Section # and Name	Description of Change	Brief Rationale
1.3 Schedule of activities<br>10.2 Appendix 2: Clinical laboratory tests	To remove the description about false positivity	To clarify that the testing will be performed if considered that the patient is immune after a natural infection
1.3 Schedule of activities<br>10.2 Appendix 2: Clinical laboratory tests	To add “tetranor PGDM” into urinalysis	Tetranor PGDM will be tested in urinalysis since it is a Type 2 inflammation biomarker.
10.3 Appendix 3: Adverse events: Definitions and procedures for recording, evaluating, follow-up, and reporting	To update the AE and SAE recording	To minimize the transfer of personal data to be compliant with the General Data Protection Regulation (GDPR) and harmonize processes across countries.
10.4 Appendix 4: Contraceptive guidance and collection of pregnancy information	To remove the “Acceptable methods” in contraception guidance	To clarify the contraceptive methods can be used during the study.
10.7.2 Investigator’s global assessment of prurigo nodularis (IGA PN)<br>10.7.3 Prurigo activity score (PAS)	To add the copyright information	To add copyright provided by the author for the IGA PN, PAS baseline and PAS Follow-up versions
10.10 Appendix 10: Country-specific Requirements<br>1.3 Schedule of activities (Footnote f)	To add country-specific requirements for China, urine pregnancy test can be done at local lab, and scheduled PK and ADA samples will not be collected (samples for ADA will be collected for analysis in the event of any SAE for patients from China site).	Based on China health authority, local Ethics Committee and site requirement
	Minor editorial and formatting changes were done throughout the document.	To correct minor errors or formatting issues.

10.12.2 Amended protocol 02 (14 April 2021)

Amended protocol 02 was submitted in few countries but was withdrawn following FDA’s feedback and was not implemented in any country.

This Amended Protocol 02 (Amendment 02) is considered to be substantial based on the criteria set forth in Article 10(a) of Directive 2001/20/EC of the European Parliament and the Council of the European Union.

Overall rationale for the Amendment

EFC16459 and EFC16460 are 2 pivotal phase 3 studies of identical design. The purpose of this protocol amendment is to allow for the timing for the primary database lock of EFC16459 to be re-evaluated based on the observed treatment effect size in EFC16460.

Protocol amendment summary of changes table

Section # and Name	Description of Change	Brief Rationale
1.1 Synopsis: Statistical considerations - Primary analysis	Added text:<br>In the case of an early primary database lock, the analysis population will be the ITT-Week-12 population for endpoints which occur earlier than or equal to Week 12. The ITT-Week-12 population consists of all participants who had been randomized by 14 June 2021. The analysis population for Week 24 endpoints will be the ITT-Week-24 population. The ITT-Week-24 population consists of all participants who had been randomized by 22 March 2021.<br>Modification in accordance with the above proposal will be documented in the statistical analysis plan (SAP) prior to the database lock.	EFC16459 and EFC16460 are 2 pivotal phase 3 studies of identical design with EFC16460 data that will be available prior to EFC16459 data. Using data from EFC16460, the sample size calculation will be revisited aiming for a statistical power of at least 90% at an alpha level of 0.05 for the primary and key secondary endpoints in the respective analysis populations and the database lock will be performed when the number of participants based on this revised sample size are expected to have reached their Week 24 visit. This revised estimation may result in database lock occurring earlier than last participant reaching the end of treatment (EOT). At a minimum, this will include at least 135 participants having Week 24 pertinent data included at the time of database lock. Based on the timing of the availability of the EFC16460 results, and to ensure a minimum of 135 participants have the opportunity to reach the Week 24 endpoint, the randomization cut-off dates have been assessed as 22 March 2021 for Week 24 and 14 June 2021 for Week 12.
1.1 Synopsis: Statistical considerations - Planned database lock date	Adapted text:<br>A primary database lock is currently planned to be performed when all randomized participants have completed their 24-week treatment period. However, the timing for the primary analysis will be re-evaluated based on the observed treatment effect size in EFC16460. If the revised sample size calculation using EFC16460 treatment effect size maintains a statistical power of at least 90% at an alpha level of 0.05 for the primary and key secondary endpoints in the respective analysis populations, a database lock will be performed when the number of participants based on this revised sample size are expected to have reached their Week 24 visit. This database lock will be based on this revised estimation and may occur earlier than last participant reaching EOT. At a minimum, this will include 135 participants having Week 24 pertinent data included at the time of database lock. In the case of an earlier database lock prior to the last participant reaching the EOT visit, the primary endpoint at Week 12 and all secondary endpoints at Week 12,	

Section # and Name	Description of Change	Brief Rationale
1.1 Synopsis: Statistical considerations - Unblinding plan	Week 24, and other study weeks would be analyzed as the final analysis for these endpoints, and enrollment of any additional participants will stop.<br>The database will be updated at the end of the study for all participants to include the post-treatment follow-up information and updates for the events previously ongoing at the time of the primary lock. Additional data collected between the primary database lock and last participant completing last visit will be summarized in a separate clinical study report (CSR) addendum, as needed. Details will be included in the SAP.<br>Added text:<br>If database lock occurs prior to when the last enrolled participant has reached the EOT visit, specific steps will be taken to maintain the blind of the study to all individuals involved in the conduct of the study and/or analysis, and to protect the overall blinding and integrity of the study data, after the primary database lock has been performed.	
9.3 Populations for Analyses – Table 5	Added 2 more populations for analyses in Table 5 in the case an earlier primary database lock occurs.<br>The ITT-Week-12 population consists of all participants who had been randomized by 14 June 2021.<br>The ITT-Week-24 population consists of all participants who had been randomized by 22 March 2021.<br>Efficacy: the analysis population for the efficacy endpoints was adapted as described below.	To detail how the endpoints will be analyzed in the case of an early primary database lock. Based on the timing of the availability of the EFC16460 results, and to ensure a minimum of 135 participants have the opportunity to reach the Week 24 endpoint, the randomization cut-off dates have been assessed as 22 March 2021 for Week 24 and 14 June 2021 for Week 12.
9.4.1 Efficacy analyses	Added text:<br>The analysis population for the efficacy endpoints will be the ITT population. In the case of an early primary database lock prior to the last enrolled participant reaching EOT, the analysis population will be the ITT-Week-12 population for endpoints which occur earlier than or equal to Week 12. The ITT-Week-12 population consists of all participants who had been randomized by 14 June 2021. The analysis population for Week 24 endpoints will be the ITT-Week-24 population. The ITT-Week-24 population consists of all participants who had been randomized by 22 March 2021.<br>Modification in accordance with the above proposal will be documented in the SAP prior to the database lock.	To provide detail of how data from timepoint evaluations will be handled in the case of an early primary database lock.
9.4.2 Safety analyses	Added text:<br>In the case of an early primary database lock prior to the last enrolled participant reaching EOT, the analysis of safety will be performed on all available and safety data collected up to the time of the primary database lock.	With an early primary analysis, participants may have different treatment exposure because of study design.

Section # and Name	Description of Change	Brief Rationale
	Added text in Table 7:<br>In addition, exposure-adjusted AE incidence rate tables will provide the number of patients with at least 1 event per 100 patient-years, presented by SOC and PT.	
9.5 Interim Analyses	Removed text:<br>A primary database lock will be performed when all randomized participants in this study have completed their 24-week treatment phase. Final analyses in the CSR will be based on this database. The database will be updated at the end of the study for all participants to include the post-treatment follow-up information and updates for the events previously ongoing at the time of the primary lock. Additional data between this database lock and last participant completing last visit will be summarized in a CSR addendum. More details will be described in the SAP.	This text was adapted and moved to Section 9.6 (new section dedicated to timing of primary database lock) and added in Section 1.1 Planned Database lock date.
9.5.1 Data Monitoring Committee	This section has been moved to Section 9.7.	This text does not belong to the interim analysis section.
9.5.2 Unblinding plan	This section has been moved to Section 9.8.	This text does not belong to the interim analysis section.
9.6 Timing of primary database lock	New section created<br>A primary database lock is currently planned to be performed when all randomized participants have completed their 24-week treatment phase. However, the timing for the primary analysis will be re-evaluated based on the observed treatment effect size in EFC16460. If the revised sample size calculation using EFC16460 treatment effect size maintains a statistical power of at least 90% at an alpha level of 0.05 for the primary and key secondary endpoints in the respective analysis populations, a database lock will be performed when the number of participants based on this revised sample size are expected to have reached their Week 24 visit. This database lock will be based on this revised estimation and may occur earlier than last participant reaching EOT. At a minimum, this will include 135 participants having Week 24 pertinent data included at the time of database lock. In the case of an earlier database lock prior to the last participant reaching the EOT visit, the primary endpoint at Week 12 and all secondary endpoints at Week 12, Week 24, and other study weeks would be analyzed as the final analysis for these endpoints, and enrollment of any additional participants will stop.<br>The database will be updated at the end of the study for all participants to include the data collected after the primary data cut-off and updates for the events previously ongoing at the time of the primary database lock. Additional data collected between the primary	The timing for the primary analysis of EFC16459 will be re-evaluated based on the observed treatment effect size in the identical study EFC16460. If the revised sample size calculation using EFC16460 treatment effect size maintains a statistical power of at least 90% at an alpha level of 0.05 for the primary and key secondary endpoints in the respective analysis populations, a database lock will be performed when the number of participants based on this revised sample size are expected to have reached their Week 24 visit. This database lock will be based on this revised estimation and may occur earlier than last participant reaching EOT.

Section # and Name	Description of Change	Brief Rationale
	database lock and last participant completing last visit will be summarized in a separate CSR addendum, as needed. Details will be included in the SAP.	
9.7 Data Monitoring Committee (DMC)	New section created, replacing former Section 9.5.1.	
9.8 Unblinding plan	New section created, replacing former Section 9.5.2.<br>Added text:<br>If database lock occurs prior to when the last enrolled participant has reached the EOT visit, specific steps will be taken to maintain the blind of the study to all individuals involved in the conduct of the study and/or analysis, and to protect the overall blinding and integrity of the study data, after the primary database lock has been performed.	To ensure the study data integrity for the data after the potential early primary database lock.
10.12 Appendix 12: Protocol amendment history	The overall rationale and table with summary of changes for Amended protocol 01 were moved from the cover page to Section 10.12.1.	This is aligned with Sanofi procedures.
Global	Minor editorial and formatting changes were done throughout the document.	To correct minor errors or formatting issues.

11 REFERENCES

1. Zeidler C, Athanasios T, Pereira M, Stander H, Yosipovitch G, Stander S. Chronic prurigo of nodular type: A Review. Acta Derm Venereol 2018;98(2):173-9.

2. Iking A, Grundmann S, Chatzigeorgakidis E, Phan NQ, Klein D, Ständer S. Prurigo as a symptom of atopic and non-atopic diseases: aetiological survey in a consecutive cohort of 108 patients. J Eur Acad Dermatol Venereol. 2013;27(5):550-7.

3. Mollanazar NK, Elgash M, Weaver L, Valdes-Rodriguez R, Hsu S. Reduced itch associated with dupilumab treatment in 4 patients with prurigo nodularis. JAMA Dermatol. 2019;155(1):121-2.

4. Beck KM, Yang EJ, Sekhon S, Bhutani T, Liao W. Dupilumab treatment for generalized prurigo nodularis. JAMA Dermatol. 2019;155(1):118-20.

5. Rambhia PH, Levitt JO. Recalcitrant prurigo nodularis treated successfully with dupilumab. JAAD Case Rep. 2019;5(5):471-3.

6. Calugareanu A, Jachiet M, Lepelletier C, De Masson A, Rybojad M, Bagot M, et al. Dramatic improvement of generalized prurigo nodularis with dupilumab. J Eur Acad Dermatol Venereol. 2019; 33(8):e303-4.

7. Zhai LL, Savage KT, Qiu CC, Jin A, Valdes-Rodriguez R, Mollanazar NK. Chronic Pruritus Responding to Dupilumab-A Case Series. Medicines (Basel). 2019;6(3):E72.

8. Weigelt N, Metze D, Stander S. Prurigo nodularis: systematic analysis of 58 histological criteria in 136 patients. J Cutan Pathol. 2010;37(5):578-86

9. Yang TB, Kim BS. Pruritus in allergy and immunology. J Allergy Clin Immunol. 2019;144(2):353-60.

10. Pereira MP, Steinke S, Zeidler C, Forner C, Riepe C, Augustin M, et al. European academy of dermatology and venereology European prurigo project: expert consensus on the definition, classification and terminology of chronic prurigo. J Eur Acad Dermatol Venereol. 2018;32(7):1059-65.

11. Zeidler C, Ständer S. The pathogenesis of Prurigo nodularis--'Super-Itch' in exploration. Eur J Pain. 2016;20(1):37-40.

12. Fukushi S, Yamasaki K, Aiba S. Nuclear localization of activated STAT6 and STAT3 in epidermis of prurigo nodularis. Br J Dermatol. 2011;165(5):990-6.

13. Tokura Y, Yagi H, Hanaoka K, Ito T, Towyama K, Sachi Y, et al. Subacute and chronic prurigo effectively treated with recombination interferon-gamma: implications for participation of Th2 cells in the pathogenesis of prurigo. Acta Derm Venereol. 1997;77(3):231-4.

14. Oetjen LK, Mack MR, Feng J, Whelan TM, Niu H, Guo CJ, et al. Sensory neurons co-opt classical immune signaling pathways to mediate chronic itch. Cell. 2017;171(1):217-28.

15. Boozalis E, Tang O, Patel S, Semenov YR, Pereira MP, Stander S, et al. Ethnic differences and comorbidities of 909 prurigo nodularis patients. J Am Acad Dermatol. 2018;79(4):714-9.

16. Steinke S, Zeidler C, Riepe C, Bruland P, Soto-Rey I, Storck M, et al. Humanistic burden of chronic pruritus in patients with inflammatory dermatoses: Results of the European Academy of Dermatology and Venereology Network on Assessment of Severity and Burden of Pruritus (PruNet) cross-sectional trial. J Am Acad Dermatol. 2018;79(3):457-63.

17. Tanaka M, Aiba S, Matsumura N, Aoyama H, Tagami H. Prurigo nodularis consists of two distinct forms: early-onset atopic and late-onset non-atopic. Dermatology. 1995;190(4):269-76.

18. Kowalski EH, Kneiber D, Valdebran M, Patel U, Amber KT. Treatment-resistant prurigo nodularis: challenges and solutions. Clin Cosmet Investig Dermatol. 2019;12:163-72.

19. Novartis Laboratories. NEORAL Soft Gelatin Capsules (cyclosporine capsules, USP) MODIFIED NEORAL Oral Solution (cyclosporine oral solution, USP) MODIFIED. Prescribing information. 2009.

20. Ständer S, Kwon P, Hirman J, Perlman AJ, Weisshaar E, Metz M, et al. Serlopitant reduced pruritus in patients with prurigo nodularis in a phase 2, randomized, placebo-controlled trial. J Am Acad Dermatol. 2019;80(5):1395-402.

21. Verweyen E, Ständer S, Kreitz K, Höben I, Osada N, Gernart M, et al. Validation of a Comprehensive Set of Pruritus Assessment Instruments: The Chronic Pruritus Tools Questionnaire PRURITOOLS. Acta Derm Venereol. 2019;99(7):657-63.

22. Reich A, Bożek A, Janiszewska K, Szepietowski JC. 12-Item Pruritus Severity Scale: Development and Validation of New Itch Severity Questionnaire. Biomed Res Int. 2017;2017:3896423.

23. Reich A, Riepe C, Anastasiadou Z, Mędrek K, Augustin M, Szepietowski JC, et al. Itch Assessment with Visual Analogue Scale and Numerical Rating Scale: Determination of Minimal Clinically Important Difference in Chronic Itch. Acta Derm Venereol. 2016;96(7):978-80.

24. Phan NQ, Blome C, Fritz F, Gerss J, Reich A, Ebata T, et al. Assessment of pruritus intensity: prospective study on validity and reliability of the visual analogue scale, numerical rating scale and verbal rating scale in 471 patients with chronic pruritus. Acta Derm Venereol. 2012;92(5):502-7.

25. Pölking J, Zeidler C, Schedel F, Osada N, Augustin M, Metze D, et al. Prurigo Activity Score (PAS): validity and reliability of a new instrument to monitor chronic prurigo. J Eur Acad Dermatol Venereol. 2018;32(10):1754-60.

26. Chernyshov PV. The evolution of quality of life assessment and use in dermatology. Dermatology. 2019;235(3):167-74.

27. Finlay AY, Khan GK. Dermatology Life Quality Index (DLQI)--a simple practical measure for routine clinical use. Clin Exp Dermatol. 1994;19(3):210-6.

28. Zigmond AS, Snaith RP. The hospital anxiety and depression scale. Acta Psychiatr Scand. 1983;67(6):361-70.

29. Herrmann C. International experiences with the Hospital Anxiety and Depression Scale--a review of validation data and clinical results. J Psychosom Res. 1997;42(1):17-41.

30. Herdman M, Gudex C, Lloyd A, Janssen M, Kind P, Parkin D, et al. Development and preliminary testing of the new five-level version of EQ-5D (EQ-5D-5L). Qual Life Res. 2011;20(10):1727-36.

31. Sampson HA, Munoz-Furlong A, Campbell RL, Adkinson NF, Bock SA, Branum A, et al. Second symposium on the definition and management of anaphylaxis: Summary report - Second National Institute of Allergy and Infectious Disease/Food Allergy and Anaphylaxis Network symposium. J Allergy Clin Immunol. 2006;117(2):391-7.

Signature Page for VV-CLIN-0579815 v3.0
efc16459-16-1-1-amended-protocol03

Approve & eSign	[redacted]

Approve & eSign	[redacted]

STATISTICAL ANALYSIS PLAN

**Protocol title:**	**A randomized, double blind, placebo-controlled, multi-center, parallel group study to evaluate the efficacy and safety of dupilumab in patients with prurigo nodularis who are inadequately controlled on topical prescription therapies or when those therapies are not advisable**
**Protocol number:**	**EFC16459**
**Compound number (INN/Trademark):**	**SAR231893/REGN668 dupilumab/Dupixent**
**Study phase:**	**Phase 3**
**Short title:**	**Study of dupilumab for the treatment of patients with prurigo nodularis, inadequately controlled on topical prescription therapies or when those therapies are not advisable**<br>**LIBERTY-PN PRIME**
**Statistician:**	[redacted]
**Statistical project leader:**	[redacted]
**Date of issue:**	**11-Nov-2021**
**Regulatory agency identifier number(s):**	
IND:	IND107969
EudraCT:	2019-003774-41
NCT:	NCT04183335
WHO:	U1111-1241-8153
Other:	Not applicable

Total number of pages: 63

Any and all information presented in this document shall be treated as confidential and shall remain the exclusive property of Sanofi (or any of its affiliated companies). The use of such confidential information must be restricted to the recipient for the agreed purpose and must not be disclosed, published or otherwise communicated to any unauthorized persons, for any reason, in any form whatsoever without the prior written consent of Sanofi (or the concerned affiliated company); 'affiliated company' means any corporation, partnership or other entity which at the date of communication or afterwards (i) controls directly or indirectly Sanofi, (ii) is directly or indirectly controlled by Sanofi, with 'control' meaning direct or indirect ownership of more than 50% of the capital stock or the voting rights in such corporation, partnership or other entity

TABLE OF CONTENTS

STATISTICAL ANALYSIS PLAN 1

TABLE OF CONTENTS 2

LIST OF TABLES 4

VERSION HISTORY 5

1 INTRODUCTION 8

1.1 STUDY DESIGN 8

1.2 OBJECTIVE AND ENDPOINTS 8
1.2.1 Estimands 10

2 SAMPLE SIZE DETERMINATION 13

3 ANALYSIS POPULATIONS 14

4 STATISTICAL ANALYSES 16

4.1 GENERAL CONSIDERATIONS 16

4.2 PARTICIPANT DISPOSITIONS 16

4.3 PRIMARY ENDPOINT(S) ANALYSIS 17
4.3.1 Definition of endpoint(s) 17
4.3.2 Main analytical approach 19
4.3.3 Sensitivity analysis 20
4.3.4 Supplementary analyses 20
4.3.5 Subgroup analyses 21

4.4 SECONDARY ENDPOINT(S) ANALYSIS 22
4.4.1 Key/Confirmatory secondary endpoint(s) 22
4.4.1.1 Definition of endpoint(s) 22
4.4.1.2 Main analytical approach 22
4.4.2 Supportive secondary endpoint(s) 22

4.5 TERTIARY/EXPLORATORY ENDPOINT(S) ANALYSIS 25
4.5.1 Definition of endpoint(s) 25
4.5.2 Main analytical approach 28

4.6 MULTIPLICITY ISSUES 29

4.7 SAFETY ANALYSES 29
4.7.1 Extent of exposure 30
4.7.2 Adverse events 31
4.7.3 Additional safety assessments....35
4.7.3.1 Laboratory variables, vital signs and electrocardiograms (ECGs)....35
4.8 OTHER ANALYSES....37
4.8.1 Pharmacokinetic analyses 37
4.8.2 Immunogenicity analyses....38
4.8.3 Pharmacodynamic/genomics endpoints 40
4.9 INTERIM ANALYSES 41
5 SUPPORTING DOCUMENTATION 42
5.1 APPENDIX 1 LIST OF ABBREVIATIONS 42
5.2 APPENDIX 2 CHANGES TO PROTOCOL-PLANNED ANALYSES 43
5.3 APPENDIX 3 DEMOGRAPHICS AND BASELINE CHARACTERISTICS, PRIOR OR CONCOMITANT MEDICATIONS/PROCEDURES....46
5.4 APPENDIX 4 DATA HANDLING CONVENTIONS 51
5.5 APPENDIX 5 SAMPLE SAS CODE....57
5.6 APPENDIX 6 SELECTION CRITERIA FOR AE/MEDICATION GROUPINGS 59
5.7 APPENDIX 7 MEDICATION/PROCEDURE ADJUDICATION ALGORITHM....61

LIST OF TABLES

Table 1 - Major changes in statistical analysis plan 5
Table 2 - Objectives and endpoints 8
Table 3 - Summary of primary estimand for main endpoints 11
Table 4 - Populations for analyses 14
Table 5 - Prohibited medications/procedures and rescue medications that impact efficacy 18
Table 6 - Sorting of AE tables 31
Table 7 - Analyses of adverse events 32
Table 8 - Selections for AESIs and other AEs of interest 33
Table 9 - Major statistical changes in protocol amendment(s) 43
Table 10 - Time window for eDiary efficacy variables 52
Table 11 - Time window for efficacy variables 54
Table 12 - Time window for safety endpoints 56
Table 13 - Time window for pharmacokinetics/pharmacodynamics variables 57
Table 14 - List of PTs or Medications for CMQs/CDGs 59

VERSION HISTORY

This Statistical Analysis Plan (SAP) for study EFC16459 is based on Protocol Amendment 1 dated 20-May-2020 and a health authority's feedback on Protocol Amendment 2 dated 14-Apr-2021, after which, the Sponsor made the decision to complete the study as originally planned instead of resizing the study based on the results of EFC16460, and on Protocol Amendment 3 dated on 21-Oct-2021. This section summarizes major changes to the statistical analysis features in the SAP. All changes to the statistical analysis features from the original protocol to Amendment 3 are described in Appendix 2.

Table 1 - Major changes in statistical analysis plan

SAP Version	Approval Date	Changes	Rationale
1.0	04-Feb-2021	Not Applicable	Original version
2.0	20-Apr-2021	In the case of an early primary database lock prior to the last enrolled participant reaching the end of treatment (EOT), analyses for the endpoints up to Week 12 use ITT-Week-12 population and beyond Week 12 use the ITT-Week-24 population.	EFC16459 and EFC16460 are 2 pivotal Phase 3 studies of identical design with EFC16460 data that will be available prior to EFC16459 data. Using data from EFC16460, the sample size calculation will be revisited aiming for a statistical power of at least 90% at an alpha level of 0.05 for the primary and key secondary endpoints in the respective analysis populations and the database lock will be performed when the number of participants based on this revised sample size are expected to have reached their Week 24 visit. This revised estimation may result in database lock occurring earlier than last participant reaching the end of treatment. At a minimum, this will include at least 135 participants having Week 24 pertinent data included at the time of database lock. Based on the timing of the availability of the EFC16460 results, and to ensure a minimum of 135 participants have the opportunity to reach the Week 24 endpoint, the randomization cut-off dates have been assessed as 22 March 2021 for Week 24 and 14 June 2021 for Week 12.
		Add ITT-Week-12 and ITT-Week-24 populations	Same as above.
		In the case of an early primary database lock prior to the last enrolled participant reaching EOT, the additional exposure-adjusted adverse event incidence rate tables will provide the number of patients with at least 1 event per 100 patient-years.	Same as above
		Remove Appendix 7 in Section 5.7	A separate medication/procedure adjudication document for details

SAP Version	Approval Date	Changes	Rationale
3.0	08-Aug-2021	Remove all modifications specific to early database lock previously made in SAP version 2 to align with Protocol Amendment 2 including discussion on the criteria used to perform early database lock and reference to the ITT-Week 12 and ITT-Week 24 populations.	Based on a health authority's feedback on Protocol Amendment 2, the Sponsor made the decision to complete the study as originally planned instead of resizing the study based on the results of EFC16460
		Retain additional exposure-adjusted adverse event incidence rate tables that will provide the number of patients with at least 1 event per 100 patient-years previously made in SAP version 2.	As requested by a health authority to include exposure-adjusted incidence rate and patient years in the summaries for TEAEs and AESIs.
		Retain "Keratitis FDA" to other AE groupings previously made in SAP version 2	For consistency with other dupilumab studies
		Add objective "To demonstrate efficacy of dupilumab on both itch as well as skin lesions within the same participant" and multicomponent endpoint (Proportion of participants with both an improvement (reduction) in WI-NRS by ≥4 from baseline to Week 24 and an IGA PN-S 0 or 1 score at Week 24) as a key secondary endpoint for the US and US reference countries hierarchy.	To add this multicomponent endpoint as another measure of treatment success based on feedback from a health authority.
		Add supplementary analysis (tipping point analyses) for primary and key secondary endpoints	This additional supplementary analysis was recommended by a health authority to assess whether the estimate, and the inference thereof, was robust to departure from the strategies used in primary analysis for handling intercurrent events and missing data.
		Add subgroup analyses for key secondary endpoints.	To evaluate whether the treatment effect is consistent across pre-specified sub-groups on the key secondary endpoints in addition to the primary endpoint.
		Add subgroup analyses for participants who have been impacted (or not) by COVID-19	To address a health authority's request to assess the impact of the treatment effect by COVID-19.
		Modify hierarchical order for multiplicity procedure for US and US reference countries	To include the multicomponent endpoint in the hierarchy to address the health authority's feedback requesting assessment of this endpoint as another measure of treatment success.
		Add as-observed plus multiple imputation supplementary analysis for the secondary endpoint (percent change from baseline in WI-NRS at Week 24)	To explore the robustness of the estimate, and the inference thereof, with a different strategy for handling intercurrent events and missing data

SAP Version	Approval Date	Changes	Rationale
		Remove “selected” in the text including “selected prohibited and/or rescue medication”	Since a participant who takes any protocol specified prohibited medications/procedures and/or rescue medications is considered as a non-responder after medical adjudication/confirmation conducted in a blinded fashion, use of the word "selected" may be confusing.
		Add Appendix 7 back in Section 5.7	Add this medication/procedure adjudication algorithm back as it was inadvertently removed in SAP Version 2.
4.0	11-Nov-2021	To promote the “proportion of participants with improvement (reduction) in worst-itch numeric rating scale (WI-NRS) by ≥4 from baseline to Week 24 as primary endpoint and to move the “proportion of participants with improvement (reduction) in WI-NRS by ≥4 from baseline to Week 12” to a secondary endpoint	Based on the data from EFC16460, the treatment effect of dupilumab continued to improve over time through Week 24. Therefore, the Sponsor proposes to assess the proportion of participants with improvement (reduction) in WI-NRS by ≥4 at Week 24, which represents the effect more accurately and synchronizes the primary itch assessment with the primary lesion assessment. This is consistent with primary endpoint change in Protocol Amendment 3.
		To update hierarchical order in Section 4.6 by removing “Proportion of participants with improvement (reduction) in WI-NRS by ≥4 from baseline to Week 12”, “Proportion of participants with IGA PN-S 0 or 1 score at Week 12” and “Change from baseline in Sleep-NRS to Week 24”	Updated power calculations performed using data from study EFC16460 showed low power for demonstrating statistical significance for each of these three endpoints. Accordingly, the hierarchical order was revised to remove these endpoints.

The first participant was randomized on 2020-01-02.

1 INTRODUCTION

1.1 STUDY DESIGN

This study is a multi-center, 24-week treatment, parallel group, double-blind, randomized, placebo-controlled study to evaluate the use of dupilumab in participants with prurigo nodularis (PN) inadequately controlled on topical prescription therapies or when those therapies are not advisable. The study will assess the effect of dupilumab on itch improvement as well as its effect on PN lesions, on participants' health-related quality of life (HRQoL), anxiety and depression, sleep quality, skin pain, and overall health status.

After 2-4 weeks of screening, participants will be centrally randomized using a permuted block randomization schedule via Interactive Voice Response System/Interactive Web Response System (IVRS/IWRS) in a 1:1 randomization ratio to dupilumab 300 mg q2w or matching placebo. Randomization will be stratified by documented history of atopy (atopic or non-atopic), stable use of topical corticosteroids (TCS)/topical calcineurin inhibitors (TCI) (yes or no), and country/territory code.

A total of approximately 150 participants will be randomized to two treatment arms (75 participants/arm). The number of participants with active mild AD upon study entry will represent up to 10% of the atopic participants. Both the atopic and the non-atopic PN populations will each be capped at 60% of the total enrolled population.

The study duration consists of the following periods:

- Screening period (2-4 weeks)
- Randomized IMP intervention period (24 weeks)
- Follow-up period (12 weeks).

1.2 OBJECTIVE AND ENDPOINTS

Table 2 - Objectives and endpoints

Objectives	Endpoints
**Primary**	
• To demonstrate the efficacy of dupilumab on itch response in participants with PN, inadequately controlled on topical prescription therapies or when those therapies are not advisable.	• Proportion of participants with improvement (reduction) in worst-itch numeric rating scale (WI-NRS) by ≥4 from baseline to Week 24.
**Secondary**	
• To demonstrate the efficacy of dupilumab on additional itch endpoints in participants with PN, inadequately controlled on topical prescription therapies or when those therapies are not advisable.	• Time to onset of effect on pruritus as measured by proportion of participants with an improvement (reduction) in WI-NRS by ≥4 from baseline during the 24-week treatment period.<br>• Change from baseline in WI-NRS at Week 24.<br>• Change from baseline in WI-NRS at Week 12.

Objectives	Endpoints
	• Percent change from baseline in WI-NRS at Week 24.<br>• Percent change from baseline in WI-NRS at Week 12.<br>• Proportion of participants with improvement (reduction) in WI-NRS by ≥4 from baseline to Week 12<br>• Percent change from baseline in WI-NRS at Week 4.<br>• Percent change from baseline in WI-NRS at Week 2.<br>• Percent change from baseline in WI-NRS over time until Week 24.<br>• Proportion of participants with WI-NRS reduction ≥4 at Week 4.<br>• Proportion of participants with WI-NRS reduction ≥4 over time until Week 24[a].<br>• Onset of action in change from baseline in WI-NRS (first p <0.05 difference from placebo in the daily WI-NRS that remains significant at subsequent measurements) until Week 12[b].
• To demonstrate efficacy of dupilumab on skin lesions of PN.	• Proportion of participants with Investigator's Global Assessment 0 or 1 score for PN-Stage (IGA PN-S) at Week 24 [Key secondary endpoint].<br>• Proportion of participants with IGA PN-S 0 or 1 score at Week 12.<br>• Proportion of participants with IGA PN-S 0 or 1 score at Week 8[c].<br>• Proportion of participants with IGA PN-S 0 or 1 score at Week 4[c].<br>• Change from baseline in IGA PN-S score at Week 24.<br>• Change from baseline in IGA PN-S score at Week 12.<br>• Change from baseline in IGA PN-S score at Week 8.<br>• Change from baseline in IGA PN-S score at Week 4.<br>• Proportion of participants with Investigator's Global Assessment 0 or 1 score for PN-Activity (IGA PN-A) at Week 24.<br>• Proportion of participants with IGA PN-A 0 or 1 score at Week 12.<br>• Proportion of participants with IGA PN-A 0 or 1 score at Week 8.<br>• Proportion of participants with IGA PN-A 0 or 1 score at Week 4.
• To demonstrate efficacy of dupilumab on both itch as well as skin lesions within the same participant	• Proportion of participants with both an improvement (reduction) in WI-NRS by ≥4 from baseline to Week 24 and an IGA PN-S 0 or 1 score at Week 24 (key secondary endpoint for US and US reference countries only)
• To demonstrate the improvement in health-related quality of life (HRQoL).	• Change from baseline in HRQoL, as measured by Dermatology Life Quality Index (DLQI) to Week 24.<br>• Change from baseline in HRQoL, as measured by DLQI to Week 12.
• To evaluate safety outcome measures.	• Percentage of participants experiencing treatment-emergent adverse events (TEAEs) or serious adverse events (SAEs) from baseline through Week 24.

Objectives	Endpoints
• To evaluate immunogenicity of dupilumab.	• Incidence of treatment-emergent antidrug antibodies (ADA) against dupilumab over time.
**Tertiary/exploratory**	
• To demonstrate a reduction in the use of rescue medication and systemic immunosuppressant	• Use of high potency or superpotent TCS rescue medication through Week 24<br>• Use of systemic immunosuppressants through Week 24, constituting treatment failure
• To evaluate exploratory outcome measures	• Change from baseline in Hospital Anxiety and Depression Scale (HADS) total score to Week 24.<br>• Change from baseline in EQ5D-5L to Week 24.<br>• Change from baseline in skin Pain numeric rating scale (NRS) to Week 4, Week 8, Week 12, and Week 24, respectively.<br>• Change from baseline in Sleep NRS to Week 4, Week 8, Week 12, and Week 24, respectively.<br>• Missed school/work days through Week 24.<br>• Incidence of skin-infection TEAEs (excluding herpetic infections) through Week 24.
• To evaluate the efficacy of dupilumab on skin lesions using a modified PAS 5-item questionnaire	• Proportion of participants who achieve ≥75% healed lesions from Prurigo Activity Score (PAS) at Week 4, Week 8, Week 12, and Week 24, respectively.<br>• Change from baseline in exact number of lesions in representative area (as determined from PAS) at Week 4, Week 8, Week 12, and Week 24, respectively.
• To evaluate the efficacy of dupilumab on other PN endpoints	• Change from baseline in Participant Global Impression of Severity (PGIS) of PN to Week 4, Week 8, Week 12, and Week 24, respectively.<br>• Participant Global Impression of Change (PGIC) of PN at Week 4, Week 8, Week 12, and Week 24, respectively.
PK<br>• To evaluate pharmacokinetic (PK) and pharmacodynamic (PD) outcome measures	• Serum functional dupilumab concentrations and PK profile.<br>• Pharmacodynamic response for selected biomarkers (total IgE).

a The early onset for significant change in the proportion of responders with WI-NRS reduction ≥4 between dupilumab and placebo will be claimed at Week xx if the p-value for Week xx is <0.05 and the remaining timepoints also have nominal p-value ≤0.05 until Week 12.

b The early onset for significant change in WI-NRS from baseline between dupilumab and placebo will be claimed at Week xx if the p-value for Week xx is <0.05 and the remaining timepoints also have nominal p-value ≤0.05 until Week 12.

c The early onset for reaching IGA PN-S (0,1) between dupilumab and placebo will be claimed at Week xx if the p-value for Week xx is ≤0.05 and the remaining timepoints also have nominal p-value ≤0.05 until Week 12.

1.2.1 Estimands

The primary estimands defined for main endpoints are summarized in below Table 3. More details are provided in Section 4.

Table 3 - Summary of primary estimand for main endpoints

**Endpoint Category**	Estimands			
	**Endpoint(s)**[a]	**Population**	Intercurrent event(s) strategy and missing data handling	**Population-level summary**
Primary objective: To demonstrate the efficacy of dupilumab on itch response in participants with PN, inadequately controlled on topical prescription therapies or when those therapies are not advisable.				
Primary endpoint	Proportion of participants with improvement (reduction) in worst-itch numeric rating scale (WI NRS) by ≥4 from baseline to Week 24	ITT	The intercurrent events will be handled as follows:<br>• Discontinuation of study treatment before Week 24: Off-study treatment data up to Week 24 will be included in the analysis (treatment policy strategy).<br>• Taking the prohibited medications/procedures and/or rescue medications[b] prior to Week 24: Participants will be considered as non-responders (composite strategy).<br>In addition, the missing data imputation rules are as follows:<br>• Having missing data at Week 24: Participants will be considered as non-responders	CMH test adjusted by documented history of atopy (atopic or non-atopic), stable use of TCS/TCI (yes or no), region (countries combined), and baseline antidepressant use (yes or no).
Secondary objective: To demonstrate the efficacy of dupilumab on additional itch endpoints in participants with PN, inadequately controlled on topical prescription therapies or when those therapies are not advisable.				
Secondary endpoint	Time to onset of effect on pruritus as measured by proportion of participants with an improvement (reduction) in WI-NRS by ≥4 from baseline during the 24-week treatment period	ITT	The intercurrent events will be handled as follows:<br>• Discontinuation of study treatment before Week 24: Off-study treatment data up to Week 24 will be included in the analysis (treatment policy strategy).<br>• Taking the prohibited medications/procedures and/or rescue medications[b] prior to Week 24: Analyses will be censored at Week 24 (composite strategy).<br>In addition, the missing data imputation rules are as follows:<br>• Discontinuing the study follow-up before Week 24: Analyses will be censored at the time of last WI-NRS assessment.	This time-to-event endpoint will be analyzed using the Cox proportional hazards model, including intervention group, documented history of atopy (atopic or non-atopic), stable use of TCS/TCI (yes or no), region (countries combined), and baseline antidepressant use (yes or no). The hazards ratio, its 95% confidence interval and p-value will be reported. Kaplan-Meier curves will be also provided[b]

Endpoint Category	Estimands			
	**Endpoint(s)**[a]	**Population**	Intercurrent event(s) strategy and missing data handling	**Population-level summary**
Secondary endpoint	Change from baseline in WI-NRS at Week 24	• ITT	The intercurrent events will be handled as follows:<br>• Discontinuing the study treatment: all data collected following schedule after treatment discontinuation will be used in the analysis (treatment policy strategy).<br>• Taking the prohibited medications/procedures and/or rescue medications[b] prior to Week 24: data will be set to missing values after the medication usage, and the participant's worst postbaseline value on or before the time of the medication usage will be used to impute missing endpoint value (for participants whose postbaseline values are all missing, the participant's baseline will be used to impute the missing endpoint value) (hypothetical strategy)<br>In addition, the missing data imputation rules are as follows:<br>• After discontinuation of the study treatment due to lack of efficacy prior to Week 24: WOCF approach will be used to impute missing data if needed[2].<br>• After discontinuation of the study treatment due to reasons other than lack of efficacy prior to Week 24: multiple imputation (MI) approach will be used to impute missing endpoint value, and this multiple imputation will use all participants excluding participants who have taken the prohibited medications and/or rescue medications prior to Week 24 and excluding participants who discontinue due to lack of efficacy prior to Week 24.	ANCOVA model with intervention group, documented history of atopy (atopic or non-atopic), stable use of TCS/TCI (yes or no), region (countries combined), baseline antidepressant use (yes or no), and relevant baseline measurement as covariates is used. Statistical inference obtained from all imputed data by ANCOVA model will be combined using Rubin's rule.

a Additional secondary objectives/endpoints are not included in this table but would be handled with a similar strategy as the endpoint type (ie continuous, proportion, time-to-event) at other weeks

b Prohibited medications and/or rescue medications are listed in Table 5.

2 SAMPLE SIZE DETERMINATION

The primary endpoint is the proportion of participants with WI-NRS reduction of ≥4 from baseline to Week 24. By assuming the response rate is [redacted] and [redacted] in the placebo and dupilumab arms, respectively, 56 participants/arm will provide 90% power to detect the difference of [redacted] between dupilumab and placebo with Fisher exact test at 2-sided level of 0.05. Assuming 15% drop out during treatment, the target is to randomize 75 participants/arm with a cap of up to 10% of participants in the atopic population having active mild AD.

The assumptions were based on the effect of dupilumab versus placebo in WI-NRS reduction ≥4 at Week 16 observed in participants with moderate to severe AD as seen in studies of AD-1334 (Solo1) and AD 1416 (Solo2).

Approximately 150 participants will be randomized to dupilumab or placebo in a 1:1 ratio with stratification factors of documented history of atopy (atopic or non-atopic), stable use of TCS/TCI (yes or no), and country/territory code. Both the atopic and the non-atopic PN population will be capped at 60% of the total enrolled population.

The sample size calculation was performed using SAS 9.4 power procedure.

3 ANALYSIS POPULATIONS

The following populations for analyses are defined:

Table 4 - Populations for analyses

Population	Description
Screened	All participants who sign the ICF.
Randomized	The randomized population includes all participants with a treatment kit number allocated and recorded in the interactive response technology (IRT) database, and regardless of whether the treatment kit was used or not.<br>Participants treated without being randomized will not be considered randomized and will not be included in any efficacy population.
Intent-to-treat (ITT)	All randomized participants analyzed according to the intervention group allocated by randomization regardless if treatment kit is used or not
Efficacy	The ITT population
Safety	All participants randomly assigned to study intervention and who take at least 1 dose of study intervention. Participants will be analyzed according to the intervention they actually received.<br>Randomized participants for whom it is unclear whether they took the study medication will be included in the safety population as randomized.<br>For participants who accidentally receive different treatment from the planned, the actual intervention allocation for as-treated analysis will be the dupilumab group.<br>The PD analyses will be performed on the safety population.
Pharmacokinetic (PK)	The PK population includes all participants in the safety population with at least one non-missing result for functional dupilumab concentration in serum after first dose of the study treatment. Participants will be analyzed according to the intervention actually received.
Antidrug antibody (ADA)	ADA population includes all participants in the safety population who have at least one non-missing ADA result after first dose of the study treatment. Participants will be analyzed according to the intervention actually received.

ADA: antidrug antibody; ICF: informed consent form; ITT: intent to treat; PD: pharmacodynamics: PK: pharmacokinetic.

Participants exposed to study intervention before or without being randomized will not be considered randomized and will not be included in any analysis population. The safety experience of these participants will be reported separately.

Randomized participants for whom it is unclear whether they took the study intervention will be considered as exposed and will be included in the safety population as randomized.

For any participant randomized more than once, only the data associated with the first randomization will be used in any analysis population. The safety experience associated with any later randomization will be reported separately.

For participants receiving more than one study intervention (placebo and dupilumab) during the study, the intervention group for as-treated analysis will be the dupilumab group.

Regarding the COVID-19 pandemic, additional summaries by COVID-19 subgroups (ie, impacted by the COVID-19 pandemic and NOT impacted by the COVID-19 pandemic) will be provided to assess the impact of COVID-19 on treatment effect. Participants impacted by the COVID-19 pandemic are defined as randomized participants with any critical or major deviation related to COVID-19 or who permanently discontinued study intervention or study due to COVID-19.

4 STATISTICAL ANALYSES

4.1 GENERAL CONSIDERATIONS

In general, continuous data will be summarized using the number of observations available, mean, standard deviation (SD), median, minimum, and maximum. Categorical and ordinal data will be summarized using the count and percentage of participants.

The baseline value of efficacy parameters is defined as the last available value before randomization and prior to the first dose of study medication unless below eDiary data (WI-NRS, skin Pain-NRS and Sleep-NRS) score or otherwise specified.

The baseline for weekly average WI-NRS (skin Pain-NRS and Sleep-NRS) score is defined as the average of daily non-missing scores obtained during the 7 days prior to randomization.

The baseline value of the other parameters is defined as the last available value prior to the first dose of investigational medicinal product (IMP) if the participant is treated, or the last available value up to randomization if the participant is not exposed to IMP.

Observation period

The observation period will be divided into 4 segments:

- The **pre-treatment period** is defined as the period up to first IMP administration.
- The **treatment-emergent (TE) period** is defined as the period from the first IMP administration to the last IMP administration + 98 days. The treatment-emergent period includes the following 2 periods:
  - The **on-treatment period** is defined as the period from the first IMP administration to the last administration of the IMP + 14 days
  - The **residual treatment period** is defined as the period from the end of the on-treatment period to the end of the treatment-emergent period.
- The **post-treatment period** is defined as the period from the end of the treatment-emergent period.

The on-study observation period is defined as the time from start of intervention until the end of the study defined as the status date collected on e-CRF page "Completion of End of Study".

4.2 PARTICIPANT DISPOSITIONS

The number (%) of participants included in each of the analysis populations listed in Table 4 will be summarized.

Screen failures are defined as participants who consent to participate in the study but are not subsequently randomized. The number (%) of screen failures and reasons for screen failures will be provided in the screened population.

The number (%) of participants in the following categories will be provided:

- Randomized participants
- Randomized but not exposed participants
- Randomized and exposed participants
- Participants who completed the study treatment period as per protocol
- Participants who did not complete the study treatment period as per protocol and discontinued study treatment prior to Week 12 by main reason for permanent intervention discontinuation including due to COVID-19
- Participants who did not complete the study treatment period as per protocol and discontinued study treatment prior to Week 24 by main reason for permanent intervention discontinuation including due to COVID-19
- Participants who completed the study period as per protocol
- Participants who did not complete the study period as per protocol and discontinued study by main reason for study discontinuation including due to COVID-19.
- Vital status at last study contact

The number (%) of exposed and not randomized participants will also be summarized.

In addition, the number (%) of participants screened, screened-failed, randomized, with permanent intervention discontinuation and with early study discontinuation will be provided by country and site.

<u>Protocol deviations</u>

Critical and major protocol deviations (automatic or manual) will be summarized in the randomized population and according to COVID-19 impact (ie, deviations related to the COVID-19 pandemic and deviations not related to the COVID-19 pandemic). In addition, deviations potentially impacting the primary endpoint analysis will be summarized.

4.3 PRIMARY ENDPOINT(S) ANALYSIS

4.3.1 Definition of endpoint(s)

The primary endpoint for this study is the proportion of participants with improvement (reduction) in worst-itch numeric rating scale (WI-NRS) by ≥4 from baseline to Week 24.

WI-NRS is a patient-reported outcome (PRO) comprised of a single item rated on a scale from 0 (“No itch”) to 10 (“Worst imaginable itch”). Participants are asked to rate the intensity of their worst pruritus (itch) over the past 24 hours using this scale.

The weekly average WI-NRS score at each week, which is defined as the average of daily non-missing scores within the week window of each week (see Section 5.4), will be used for analyses.

For efficacy analysis, Table 5 presents the prohibited and rescue medications/procedures which will be considered as intercurrent events if the last column indicates as "Yes" and therefore be handled in the estimands for endpoints defined in Table 3.

Table 5 - Prohibited medications/procedures and rescue medications that impact efficacy

Medication/procedure	Comment	Data to be set as non-responder after taking medication in the main statistical analysis (Yes/No)[a]/Selection criteria
Prohibited medications/procedures		
Systemic immunosuppressive/immunomodulating drugs (eg, systemic corticosteroids, cyclosporine, mycophenolate-mofetil, interferon gamma, Janus kinase inhibitors, azathioprine, methotrexate, hydroxychloroquine, dapsone, sulfasalazine, colchine, etc)	IMP to be discontinued	Yes (CDG[b] Immunosuppressant drugs Narrow and SDG[b]Corticosteroids Narrow)
Other monoclonal antibodies (that are biological response modifiers).	IMP to be discontinued	Yes (SDG[b] SDGMonoclonal antibodies Narrow)
Phototherapy, including tanning beds.	IMP to be discontinued	Yes (CMQ[b] HLT Phototherapies, CMQ[b] Tanning_single PT)
Naltrexone or other opioid antagonist	IMP to be discontinued	Yes (SDG[b] Analgesia producing opioids NARROW)
Gabapentin, pregabalin, and thalidomide	IMP to be discontinued	Yes (CDG[b] Gabapentin mono and multi ingredients, Pregabalin mono and multi ingredients, or Thalidomide mono and multi ingredients)
Paroxetine, fluvoxamine or other SSRIs.	No IMP discontinuation, see requirement in the footnote	Yes[c,d] (CDG[b] N06AB selective serotonin reuptake inhibitors)
SNRIs.	No IMP discontinuation, see requirement in the footnote	Yes[c, d] (CDG[b] serotonin and norepinephrine reuptake inhibitors)
Amitriptyline or other tricyclic or tetracyclic antidepressants	No IMP discontinuation, see requirement in the footnote	Yes[c, d] (CDG[b] N06AA non-selective monoamine reuptake inhibitors)
Intralesional corticosteroid injections and cryotherapy.	No IMP discontinuation	Yes[d] (CMQ[b] Injection_single pt, cryotherapy or skin cryotherapy PT)
Sedating antihistamine	No IMP discontinuation	Yes[d, e] (CDG[b] sedating antihistamines)
Non-sedating antihistamine if used specifically for the treatment of itch secondary to AD or PN	No IMP discontinuation	Yes[d, e] (CDG[b] non-sedating antihistamines)

Medication/procedure	Comment	Data to be set as non-responder after taking medication in the main statistical analysis (Yes/No)[a]/Selection criteria
Rescue medications		
Dermatological preparations of high potency or superpotent TCS and TCI	No IMP discontinuation	Yes[d] (CDG[b] calcineurin inhibitors and corticosteroids narrow, Ticked 'RESCUE THERAPY' in CRF page)

a When yes, the estimand for the intercurrent event handling strategy will be as follows: hypothetical for continuous endpoints, and composite for responder and time-to-event endpoints. When no, a treatment policy strategy will be applied.
b CDG=company drug groupings, CMQ=company MedDRA query, SDG= Standardized drug groupings.
c Only if the antidepressant is initiated during the study or its dose is increased from baseline provided that medication was taken at least 3 months prior to screening.
d As per medical adjudication, see Section 5.7 for details.
e Only if the medication is initiated at Week 12 or Week 24, or the dose is increased from Week 11 to Week 12 or from Week 23 to Week 24.

Blinded review of prohibited/rescue treatment (medication or procedure) based on Table 5 and a pre-specified algorithm (Section 5.7) will be implemented before database locks by considering the type of medication or procedure, indication, timing, frequency and the potential impact of the use of the prohibited medications/procedures and rescue medications.

4.3.2 Main analytical approach

The primary estimand for the primary endpoint is the treatment policy/composite approach as defined in Table 3.

The primary analysis population for the efficacy endpoints will be the ITT population.

The following null hypothesis H0 and alternative hypothesis H1 will be tested for dupilumab against placebo:

- H0: No treatment difference between dupilumab and placebo.
- H1: There is a treatment difference between dupilumab and placebo

The primary analysis will be conducted by using Cochran–Mantel–Haenszel test (CMH) test adjusted by documented history of atopy (atopic or non-atopic), stable use of TCS/TCI (yes or no), region, and baseline anti-depressant use (yes or no). Comparisons of the response rates between dupilumab and placebo will be derived. In addition, odds ratio and response rate difference as well as the corresponding 95% confidence intervals (CI) will be provided along with the p-values.

As defined in Table 3 for participants discontinuing the study treatment before Week 24, their off-study treatment values measured up to Week 24 will be included in the analysis. Participants taking the prohibited medications/procedures and/or rescue medications (see Table 5) prior to Week 24 or having missing data at Week 24 will be considered non-responders.

4.3.3 Sensitivity analysis

For the primary estimand for the primary endpoint, no sensitivity analysis will be performed. However, three supplementary analyses will be performed as described in the section below.

4.3.4 Supplementary analyses

The following supplementary analyses will be performed:

As-observed analysis (including all data after taking the prohibited and/or rescue medications)

The data collected after taking all prohibited medications and/or rescue medications will be included in the supplementary analysis to evaluate the robustness of the primary analysis results with respect to the method of handling data while taking the prohibited medications (eg, treatment policy strategy). In addition, for participants discontinuing the study treatment before Week 24, their off-study treatment values measured up to Week 24 will be included in the analysis. The participants having missing data at Week 24 regardless of reason(s) will be considered non-responders at that timepoint.

Hybrid method analysis (the worst-observation carried forward (WOCF) and multiple imputation (MI))

In the primary analysis of change from baseline in WI-NRS (continuous variable) at Week 24, the hybrid method of the WOCF and MI will be used (see Section 4.4.2). Similar to the continuous variable, the same imputation method will be used in the analysis of the proportion of participants with improvement (reduction) in WI-NRS by ≥4 from baseline to Week 24, which is consistent for the intercurrent event strategy and missing data handling in the binary variables and continuous variable. That is, after the imputation of continuous WI-NRS data at Week 24 using the hybrid method of the WOCF and MI (see Section 4.4.2), responders will be defined as patients with improvement in WI-NRS by ≥4 from baseline to Week 24 in each of the imputed datasets with about 40 imputations, and then the CMH test adjusted by documented history of atopy (atopic or non-atopic), stable use of TCS/TCI (yes or no), region, and baseline anti-depressant use (yes or no) will be used. Statistical inference obtained from all imputed data will be combined using Rubin's rule.

Tipping point analysis

The intercurrent events will be handled as follows:

- Discontinuation of study treatment before Week 24: Off-study treatment data up to Week 24 will be included in the analysis (treatment policy strategy).
- Taking the prohibited medications/procedures and/or rescue medications prior to Week 24: Data after the intercurrent events will be censored and then imputed with the below tipping point method

In addition, the missing data imputation rules are as follows:

- Having missing data at Week 24: Data will be imputed with the following tipping point method.

Tipping point method:

- A sequence of analyses will be performed with the adjustment to artificially decrease the response rate in the dupilumab group and increase the response rate in the placebo group with a fixed and definite set of values for data imputation.
- For each combination of increasing response rate in placebo and decreasing response rate in dupilumab, multiple imputed datasets will be generated and analyzed using CMH test. The results obtained from multiple imputed datasets will be combined to generate statistical inference, ie, p-value and treatment difference between 2 treatment groups.
- A “tipping point” will be identified while the result is no longer statistically significant (ie, p-value >0.05).

4.3.5 Subgroup analyses

To assess the homogeneity of the treatment effect across various subgroups, analyses will be performed on the primary endpoint across the following subgroups (categories with fewer than 5 participants may be combined with other categories):

- Age group (<65, ≥65 years)
- Gender (Male, Female)
- Region
- Territory
- Race (Caucasian/White, Black/of African descent, Asian/Oriental, Others)
- Ethnicity (Hispanic or Latino, Not Hispanic or Latino)
- Baseline weight (<60, ≥60- <90, ≥90 kg)
- Baseline BMI (<25, ≥25- <30, ≥30 kg/m^2)
- Participants without a current diagnosis of AD
- History of atopy (atopic or non-atopic)
- Stable use of TCS/TCI (yes or no)
- Antidepressant use (yes or no) at baseline
- Baseline IGA PN-S moderate versus severe (3 versus. 4)
- Participants who have not been impacted by COVID-19 vs impacted by COVID-19 (for participants who have been impacted by the COVID-19, the efficacy data will be descriptive only if the number of participants is not enough to perform statistical tests. Participants impacted by the COVID-19 pandemic are defined as randomized participants with any critical or major deviation related to COVID-19 or who permanently discontinued study intervention or study due to COVID-19.)

To test the interaction between intervention and subgroup factor, a logistic regression model incorporating subgroup-by-treatment interaction will be built for each subgroup factor except the

subgroup of participants without a current diagnosis of AD (very few AD participants will be excluded). The model will include all the covariates in the main statistical model plus the subgroup variable and the subgroup-by-treatment interaction. A p-value for the test of interaction will be provided.

In each subgroup, the treatment effects for the primary endpoint will be provided, as well as the corresponding 95% CI, using the same method as applied to the primary analysis. Forest plots will be provided.

4.4 SECONDARY ENDPOINT(S) ANALYSIS

The weekly average skin Pain-NRS or Sleep-NRS score at each week, which is defined as the average of non-missing daily scores within the week window of each week (see Section 5.4), will be used for analyses.

4.4.1 Key/Confirmatory secondary endpoint(s)

4.4.1.1 Definition of endpoint(s)

The key secondary endpoint is:

- Proportion of participants with IGA PN-S 0 or 1 score at Week 24.

In addition, for US and US reference countries only, there is another key secondary endpoint:

- Proportion of participants with both an improvement (reduction) in WI-NRS by ≥4 from baseline to Week 24 and an IGA PN-S 0 or 1 score at Week 24.

<u>Investigator's global assessment for prurigo nodularis (IGA PN)</u>

The IGA PN is a clinician-reported outcome (ClinRO) that allows clinicians to assess the activity of PN (IGA PN-A) using a 5-point scale from 0 (clear) to 4 (severe); and the stage of the disease (IGA PN-S) using a 5-point scale from 0 (clear) to 4 (severe).

4.4.1.2 Main analytical approach

For the key secondary efficacy endpoints, the analysis will be conducted by using CMH test adjusted by documented history of atopy (atopic or non-atopic), stable use of TCS/TCI (yes or no), region, and baseline anti-depressant use (yes or no). The same estimand, ie, intercurrent event strategy and missing data handling method, as the primary endpoint is used.

The same supplementary and subgroup analyses used for the primary endpoint will also be performed for the key secondary efficacy endpoints.

4.4.2 Supportive secondary endpoint(s)

The other secondary endpoints are as follows:

- Proportion of participants with WI-NRS reduction ≥4 over time until Week 24.
- Proportion of participants with WI-NRS reduction ≥4 at Week 12
- Proportion of participants with WI-NRS reduction ≥4 at Week 4
- Proportion of participants with IGA PN-S 0 or 1 score at Week 12
- Proportion of participants with IGA PN-S 0 or 1 score at Week 8
- Proportion of participants with IGA PN-S 0 or 1 score at Week 4
- Proportion of participants with IGA PN-A 0 or 1 score at Week 24
- Proportion of participants with IGA PN-A 0 or 1 score at Week 12
- Proportion of participants with IGA PN-A 0 or 1 score at Week 8
- Proportion of participants with IGA PN-A 0 or 1 score at Week 4
- Time to onset of effect on pruritus as measured by proportion of participants with an improvement (reduction) in WI-NRS by ≥4 from baseline during the 24-week treatment period
- Change from baseline in WI-NRS at Week 24
- Change from baseline in WI-NRS at Week 12
- Percent change from baseline in WI-NRS at Week 24
- Percent change from baseline in WI-NRS at Week 12
- Percent change from baseline in WI-NRS at Week 4
- Percent change from baseline in WI-NRS at Week 2
- Percent change from baseline in WI-NRS over time until Week 24.
- Onset of action in change from baseline in WI-NRS (first $p < 0.05$ difference from placebo in the daily WI-NRS that remains significant at subsequent measurements) until Week 12
- Change from baseline in IGA PN-S score at Week 24
- Change from baseline in IGA PN-S score at Week 12
- Change from baseline in IGA PN-S score at Week 8
- Change from baseline in IGA PN-S score at Week 4
- Change from baseline in HRQoL, as measured by Dermatology Life Quality Index (DLQI) to Week 24
- Change from baseline in HRQoL, as measured by Dermatology Life Quality Index (DLQI) to Week 12.

Dermatology life quality index (DLQI)

The DLQI is a PRO developed to measure dermatology-specific HRQoL in adult participants. The instrument comprises 10 items assessing the impact of skin disease on participants' HRQoL over the previous week. The items cover symptoms, leisure activities, work/school or holiday time, personal relationships including intimate, the side effects of treatment, and emotional reactions to having a skin disease. It is a validated questionnaire used in clinical practice and clinical trials. Response scale is a 4-point Likert scale (0 = "not at all" and 3 = "very much") for nine items. The remaining one item about work/studying asks whether work/study has been prevented and then (if "No") to what degree the skin condition has been a problem at work/study; the item is rated on a 3-point Likert scale ("Not at all" to "A lot"). Overall scoring ranges from 0 to 30, with a high score indicative of a poor HRQoL.

Time-to-event secondary efficacy endpoint

Time to first onset of effect on pruritus defined as an improvement (reduction) in WI-NRS by ≥4 from baseline during the 24-week treatment period will be analyzed using the Cox proportional hazards model, including treatment, documented history of atopy (atopic or non-atopic), stable use of TCS/TCI (yes or no), region, and baseline anti-depressant use (yes or no). The hazards ratio, its 95% confidence interval and p-value will be reported. Kaplan-Meier curves will be also provided.

For time to first WI-NRS response (WI-NRS by ≥4 from baseline), participants who receive the prohibited medications and/or rescue medications (see Table 5), data prior to start of the medications will be used, but after medication start, the participants' data will not be used and they will be censored at Week 24 (ie, Day 172). For other participants, all available data up to Week 24 (ie, Day 172) including those collected during the off-treatment period will be used. Participants without events will be censored at Day 172 or their last WI-NRS assessment date if discontinued from the study, whichever is earlier.

Secondary efficacy endpoints that measure binary responses

Secondary efficacy endpoints that measure binary responses will be analyzed in the same fashion as the primary endpoint using primary statistical model.

Continuous secondary efficacy endpoints

The primary estimand for continuous secondary efficacy endpoints is defined in Table 3 and will be analyzed using an analysis of covariance (ANCOVA) model with intervention group, documented history of atopy (atopic or non-atopic), stable use of TCS/TCI (yes or no), region (countries combined), baseline antidepressant use (yes or no), and relevant baseline measurement as covariate, with intercurrent event strategy and missing data handling as defined as in Table 3. Specifically, data of participants taking the prohibited medications/procedures and/or rescue medications will be set to missing after the medication usage, and the worst postbaseline value on or before the time of the medication usage will be used to impute missing endpoint value (for participants whose postbaseline values are all missing, the baseline will be used to impute). Participants who discontinue the treatment prematurely are encouraged to follow the planned clinical visits and in those participants who did not take the prohibited medications/procedures

and/or rescue medications, all data collected after treatment discontinuation will be used in the analysis. For these participants, missing data may still happen despite all efforts have been tried to collect the data after treatment discontinuation. For participants who discontinue due to lack of efficacy, all data collected after discontinuation will be used in the analysis, and a WOCF approach will be used to impute missing data if needed. For participants who discontinued not due to lack of efficacy, a multiple imputation (MI) approach will be used to impute missing endpoint value, and this MI method will use all participants excluding participants who have taken the prohibited medications/procedures and/or rescue medications prior to timepoint of endpoint of interest and excluding participants who discontinue due to lack of efficacy.

Each of the imputed complete data will be analyzed by fitting an ANCOVA model as described above. The imputation number will be about 40. Statistical inference obtained from all imputed data will be combined using Rubin's rule. Descriptive statistics including number of participants, mean, standard error, and least squares (LS) mean changes (and standard error) will be provided. In addition, difference of the dupilumab group against placebo in LS means and the corresponding 95% confidence intervals (CI) will be provided along with the p-values.

See Section 5.5 for the sample SAS code for the imputation and the analysis.

For the endpoint of percent change from baseline in WI-NRS at week 24, we will also perform an as-observed plus multiple imputation supplementary analysis as described below.

The data collected after taking all prohibited medications and/or rescue medications will be included in the supplementary analysis to evaluate the robustness of the primary analysis results with respect to the method of handling data while taking the prohibited medications (eg, treatment policy strategy). In addition, for participants discontinuing the study treatment before Week 24, their off-study treatment values measured up to Week 24 will be included in the analysis. For participants having missing data at Week 24 regardless of reason(s), a multiple imputation (MI) approach will be used to impute the missing endpoint value.

4.5 TERTIARY/EXPLORATORY ENDPOINT(S) ANALYSIS

4.5.1 Definition of endpoint(s)

The exploratory endpoints are as follows:

- Use of high potency or superpotent TCS rescue medication through Week 24
- Use of systemic immunosuppressant through Week 24, constituting treatment failure
- Change from baseline in Hospital Anxiety and Depression Scale (HADS) total score to Week 24
- Change from baseline in EQ-5D-5L Single Index score to Week 24
- Change from baseline in EQ-5D visual analog scale (VAS)to Week 24
- Change from baseline in skin Pain-NRS to Week 4, Week 8, Week 12, and Week 24, respectively

- Change from baseline in Sleep-NRS to Week 4, Week 8, Week 12, and Week 24, respectively
- Missed school/work days through Week 24
- Incidence of skin-infection TEAEs (excluding herpetic infections) through Week 24
- Proportion of participants who achieve ≥75% healed lesions from PAS at Week 4, Week 8, Week 12, and Week 24, respectively
- Change from baseline in exact number of lesions in representative area (as determined from PAS) at Week 4, Week 8, Week 12, and Week 24, respectively
- Change from baseline in Participant Global Impression of Severity (PGIS) of PN to Week 4, Week 8, Week 12, and Week 24 respectively
- Proportion of participants with PGIS score of "none" at Week 4, Week 8, Week 12, and Week 24 respectively
- Proportion of participants with PGIS score of "none" or "mild" at Week 4, Week 8, Week 12, and Week 24 respectively
- Participant Global Impression of Change (PGIC) of PN at Week 4, Week 8, Week 12, and Week 24, respectively
- Proportion of participants with PGIC score of "very much better" at Week 4, Week 8, Week 12, and Week 24 respectively
- Proportion of participants with PGIC score of "very much better" or "moderately better" at Week 4, Week 8, Week 12, and Week 24 respectively.

<u>Hospital anxiety and depression scale (HADS)</u>

The HADS is a PRO instrument for screening anxiety and depression in non-psychiatric populations; repeated administration also provides information about changes to a participant's emotional state. The HADS consists of 14 items, 7 each for anxiety and depression symptoms; possible scores range from 0 to 21 for each subscale. The following cut-off scores are recommended for both subscales:

- 0 to 7: normal
- 8 to 10: borderline abnormal (borderline case)
- 11 to 21: abnormal.

Euroqol 5 dimensions questionnaire (EQ-5D)

The Euroqol-5 dimensions (EQ-5D) is a standardized PRO measure of health status developed by the EuroQOL Group in order to provide a simple, generic measure of health for clinical and economic appraisal. The EQ-5D consists of 2 parts: the descriptive system and the EQ visual analog scale (VAS). The EQ-5D descriptive system comprises the following 5 dimensions: mobility, self-care, usual activities, pain/discomfort, and anxiety/depression. Each dimension has 5 levels of perceived problems: “no problem”, “slight problems”, “moderate problems”, “severe problems” and “inability to do the activity”. The respondent is asked to indicate his/her health state by ticking (or placing a cross) in the box against the most appropriate statement in each of the 5 dimensions; this results in a 1-digit number expressing the level for that dimension. The digits for 5 dimensions can be combined in a 5-digit number describing the respondent’s health state. The EQ VAS records the respondent’s self-rated health on a vertical VAS where the endpoints are labeled “best imaginable health state (100)” and “worst imaginable health state (0)”. This information can be used as a quantitative measure of health outcome as judged by the individual respondents.

Skin Pain and sleep numeric rating scales (skin Pain-NRS, Sleep-NRS)

Participants will be asked to rate their worst skin pain in the past 24 hours using a 0 to 10 NRS, with 0 = No pain to 10 = Worst pain possible.

In addition, participants will be asked to rate their sleep quality on their past night upon awakening, using a 0 to 10 NRS, with 0 = Worst possible sleep and 10 = Best possible sleep.

Participants will complete the skin pain NRS and sleep quality NRS once a day.

Prurigo activity score (PAS)

The PAS is a clinician-reported outcome (ClinRO) measurement. The original PAS questionnaire Version 0.9 consists of 7 items, developed by expert clinicians in PN.

A 5-item simplified version of the PAS will be used in the current study. Item 4 (exact number of pruriginous lesions in representative area) and Item 5b (percentage of healed prurigo lesions in all pruriginous lesions) will be used for analyses. In addition, Item 2 (number of prurigo lesions) may be used for analysis too.

Participant Global Impression of Change of disease (PGIC) and Participant Global Impression of Severity (PGIS)

The PGIC is a one-item questionnaire that asks participants to provide the overall self-assessment of change in their PN overall on a 7-point scale, compared to just before participant started taking the study injection. Response choices are: 0 = “Very much better”, 1 = “Moderately better”, 2 = “A little better”, 3 = “No change”, 4 = “A little worse”, 5 = “Moderately worse”, 6 = “Very much worse”.

The PGIS is a one-item questionnaire that asks participants to provide the overall self-assessment of their disease severity on a 4-point scale for the past week. Response choices are: 1 = “none”, 2 = “Mild”, 3 = “Moderate”, 4 = “Severe”.

Missed school/work days

Participants who are employed or enrolled in school will be asked to report the number of sick leave/missed school days since the last study assessment.

Photography substudy

Participants in selected sites who decide to participate in this substudy need to provide separate consent. One or several lesions will be photographed at baseline. The same lesions will be photographed at subsequent visits to evaluate their progression.

4.5.2 Main analytical approach

Exploratory efficacy endpoints will be analyzed using the same methodology as secondary efficacy for similar data (continuous or proportion) except the endpoints below.

- Use of high potency or superpotent TCS rescue medication through Week 24
- Use of systemic immunosuppressant through Week 24, constituting treatment failure
- Missed school/work days through Week 24
- Incidence of skin-infection TEAEs (excluding herpetic infections) through Week 24.

The number of participants and percentage with the events for these endpoints will be provided in observed cases. In addition, the time (week) to first event for those participants will also be analyzed using Kaplan-Meier method and the duration of the rescue medication use may be summarized if applicable.

An additional exploratory endpoint of time to first select prohibited/rescue medication that impact efficacy will be provided. This includes medications where WOCF will be applied (see Table 5).

4.6 MULTIPLICITY ISSUES

The multiplicity procedure is proposed to control the overall type-I error rate for testing the primary and selected secondary endpoints. The overall alpha is 0.05. The comparisons with placebo will be tested based on the hierarchical order below at 2-sided $\alpha = 0.05$:

In US and US reference countries:

- Proportion of participants with improvement (reduction) in WI-NRS by ≥4 from baseline to Week 24
- Proportion of participants with IGA PN-S 0 or 1 score at Week 24
- Proportion of participants with both an improvement (reduction) in WI-NRS by ≥4 from baseline to Week 24 and an IGA PN-S 0 or 1 score at Week 24
- Percent change from baseline in WI-NRS at Week 24
- Change from baseline in HRQoL, as measured by Dermatology Life Quality Index (DLQI) to Week 24
- Change from baseline in skin Pain-NRS to Week 24
- Change from baseline in Hospital Anxiety and Depression Scale (HADS) total score to Week 24

In all other countries (including EU and EU reference countries, as well as Japan):

- Proportion of participants with improvement (reduction) in WI-NRS by ≥4 from baseline to Week 24
- Proportion of participants with IGA PN-S 0 or 1 score at Week 24
- Percent change from baseline in WI-NRS at Week 24
- Change from baseline in HRQoL, as measured by Dermatology Life Quality Index (DLQI) to Week 24
- Change from baseline in skin Pain-NRS to Week 24
- Change from baseline in Hospital Anxiety and Depression Scale (HADS) total score to Week 24.

The study is considered positive when the primary endpoint achieves statistical significance.

4.7 SAFETY ANALYSES

All safety analyses will be performed on the safety population as defined in Section 3, unless otherwise specified, using the following common rules:

- The analysis of the safety variables will be descriptive, and no testing is planned.
- Safety data in participants who do not belong to the safety population (eg, exposed but not randomized) will be provided separately.

4.7.1 Extent of exposure

The extent of IMP exposure will be assessed by the duration of IMP exposure and compliance and summarized within the safety population.

Duration of IMP exposure

Duration of IMP exposure is defined as last dose date – first dose date + 14 days, regardless of unplanned intermittent discontinuations. Duration of IMP exposure will be summarized descriptively as a quantitative variable (number, mean, SD, median, minimum, and maximum). In addition, duration of treatment exposure will also be summarized categorically by numbers and percentages for each of the following categories and cumulatively according to these categories:

- >0 and ≤2 weeks
- >2 and ≤4 weeks
- >4 and ≤8 weeks
- >8 and ≤12 weeks
- >12 and ≤16 weeks
- >16 and ≤20 weeks
- >20 and ≤24 weeks
- >24 weeks and ≤ 24 weeks + 3 days
- > 24 weeks + 3 days

Additionally, the cumulative duration of treatment exposure will be provided, defined as the sum of the duration of treatment exposure for all participants, and will be expressed in participant years.

Treatment compliance

A given administration will be considered noncompliant if the participant did not take the planned dose as required by the protocol. No imputation will be made for participants with missing or incomplete data.

Percentage of treatment compliance for a participant will be defined as the number of administrations that the participant was compliant divided by the total number of administrations that the participant was planned to take from the first administration of IMP up to the actual last administration of IMP.

Treatment compliance will be summarized quantitatively and categorically: <80%, ≥80%.

Cases of overdose (defined as at least twice the intended dose during an interval of less than 11 days) will be considered an AESI per dupilumab clinical programs and will be listed as such.

4.7.2 Adverse events

General common rules for adverse events

All adverse events (AEs) will be coded to a lower-level term (LLT), preferred term (PT), high-level term (HLT), high-level group term (HLGT), and associated primary system organ class (SOC) using the Medical Dictionary for Regulatory Activities (MedDRA) version currently in effect at Sanofi at the time of database lock.

The AEs will be analyzed in the following 3 categories:

- Pre-treatment AEs: AEs that developed, worsened or became serious during the pre-treatment period.
- Treatment-emergent adverse events (TEAE)s: AEs that developed, worsened or became serious during the treatment-emergent period
- Post-treatment AEs: AEs that developed, worsened or became serious during the post-treatment period

Similarly, the deaths will be analyzed in the pre-treatment, treatment-emergent and post-treatment periods.

The primary focus of AE reporting will be on TEAEs. Pre-treatment and post-treatment AEs will be described separately.

An AE with incomplete or missing date/time of onset (occurrence, worsening, or becoming serious) will be classified as a TEAE unless there is definitive information to determine it is a pre-treatment or a post-treatment AE.

If the assessment of the relationship to IMP is missing for an AE, this AE will be assumed as related to IMP. If the severity is missing for 1 of the treatment-emergent occurrences of an AE, the severity will be imputed with the maximal severity of the other occurrences. If the severity is missing for all the occurrences, the severity will be left as missing.

Multiple occurrences of the same event in the same participant will be counted only once in the tables within a treatment phase.

The AE tables will be sorted as indicated in Table 6.

Table 6 - Sorting of AE tables

AE presentation	Sorting rules
SOC, HLGT, HLT and PT	By the internationally agreed SOC order and by alphabetic order of HLGTs, HLTs and PTs.
SOC, HLT and PT	By the internationally agreed SOC order and by alphabetic order of HLTs and PTs.
SOC and PT	By the internationally agreed SOC order and decreasing frequency of PTs[a, b]
SMQ/CMQ and PT	By decreasing frequency of SMQs/CMQs and PTs[a]

AE presentation	Sorting rules
PT	By decreasing frequency of PTs[a]

a Sorting will be based on the SAR231893 dupilumab group

b The table of all TEAEs presented by SOC and PT will define the presentation order for all other tables (eg, treatment-emergent SAE) presented by SOC and PT, unless otherwise specified.

Analysis of all adverse events

The overview of TEAE with the details below will be generated:

- Any TEAE
- Any severe TEAE
- Any treatment emergent SAE
- TEAE leading to death
- Any TEAE leading to permanent intervention discontinuation
- Any treatment emergent AESI
- Any treatment emergent other AE of interest grouping
- Any TEAE related to IMP

The AE summaries of Table 7 will be generated with number (%) of participants experiencing at least one event.

Table 7 - Analyses of adverse events

Type of AE	MedDRA levels
All TEAE	Primary SOC, HLGT, HLT and PT
	Primary SOC and PT
	PT
	Primary and secondary SOC, HLGT, HLT and PT
Common TEAE (≥2% and 5% in any group)	Primary SOC and PT
TEAE related to IMP as per Investigator's judgment	Primary SOC, HLGT, HLT and PT
	Primary SOC and PT
TEAE by maximal intensity	Primary SOC and PT
Treatment emergent SAE	Primary SOC, HLGT, HLT and PT
	Primary SOC and PT
Treatment emergent SAE related to IMP as per Investigator's judgment	Primary SOC, HLGT, HLT and PT
TEAE leading to permanent intervention discontinuation	Primary SOC, HLGT, HLT and PT
	Primary SOC and PT
TEAE leading to death (death as an outcome of the AE as reported by the Investigator in the AE page)	Primary SOC, HLGT, HLT and PT

Type of AE	MedDRA levels
Pretreatment AE	Overview[a]<br>Primary SOC and PT

a Will include the following AE categories: any AEs, any serious AEs, any AEs leading to death, any AEs leading to permanent intervention discontinuation

Analysis of deaths

In addition to the analyses of deaths included in Table 6 the number (%) of participants in the following categories will be provided:

- Deaths during the treatment-emergent and post-treatment periods
- Deaths in non-randomized or randomized but not treated participants.

Analysis of adverse events of special interest (AESIs) and other AEs of interest

Adverse events of special interest (AESIs) and other AEs of interest will be selected for analyses as indicated in Table 8. Number (%) of participants experiencing at least one event will be provided for each event of interest. Tables will be sorted as indicated in Table 6.

Table 8 - Selections for AESIs and other AEs of interest

AE Grouping	Criteria
AESI	
Anaphylactic reaction	Anaphylactic reaction algorithmic approach (Introductory Guide for Standardised MedDRA Queries (SMQs) Version 18.1): includes anaphylactic reaction narrow SMQ (20000021) terms and programmatic identification of cases based on occurrence of at least two preferred terms meeting the algorithm criteria occurring within 24 hours of each other. The latter cases identified using the algorithm will undergo blinded medical review taking into account the timing of events relative to each other and to IMP administration for final determination of an anaphylactic reaction or not.
Systemic hypersensitivity reactions	SMQ [20000214] hypersensitivity narrow search and [AE corrective treatment/therapy='Y' or Action taken with IMP='Drug withdrawn' or Action taken with IMP='Drug interrupted'] followed by blinded medical review (documented process) for selection of relevant systemic hypersensitivity events
Helminthic infections	CMQ10544 based on HLGT as "Helminthic disorder"
Any severe type of conjunctivitis	CMQ10498 based on PTs (See Section 5.6)[a] and "Severe" ticked in Adverse Events eCRF page
Any severe type of blepharitis	CMQ10497 based on HLT as "Lid, lash and lacrimal infections, irritations and inflammations" and "Severe" ticked in Adverse Events eCRF page
Keratitis	CMQ10642 based on the following PTs [keratitis, allergic keratitis, ulcerative keratitis, atopic keratoconjunctivitis, herpes ophthalmic, ophthalmic herpes simplex, corneal infection][a]
Clinically symptomatic eosinophilia (or eosinophilia associated with clinical symptoms)[b]	CMQ10641 based on HLT = Eosinophilic disorders or PT=Eosinophil count increased

AE Grouping	Criteria
Pregnancy of a female participants entered in a study as well as pregnancy occurring in a female partner of a male participant entered in a study with IMP/NIMP	"Pregnancy" or "Partner Pregnancy" checked on the Pregnancy eCRF page as reported by the investigator
Significant ALT elevation	"ALT increase" and AESI answer "Yes" checked on AE eCRF as reported by the investigator (ALT >5 x ULN in participants with baseline ALT ≤2 x ULN; OR ALT >8 x ULN if baseline ALT >2 x ULN)
Symptomatic overdose with IMP	Symptomatic Overdose is answered Yes, with Overdose of IMP answered Yes on AE eCRF.
Symptomatic overdose with NIMP	Symptomatic Overdose is answered Yes, with Overdose of NIMP answered Yes on AE eCRF.
Other selected AE Grouping	
Serious injection site reactions or severe injection site reactions that last longer than 24 hours	HLT = 'Injection site reaction' and either with serious status, or with severe status and (AE end date/time - AE start date/time) ≥24 hours or ongoing
Severe or serious infection	Primary SOC = 'Infections and infestations' and with severe or serious status
Drug-related hepatic disorder	SMQ [20000006] Drug-related hepatic disorders- narrow
Injection site reaction	HLT = 'Injection site reaction'
Malignancy	SMQ [20000091]- Malignant or unspecified tumors narrow
Suicidal behavior	CMQ10639 based on the following PTs [Completed suicide, Suicidal ideation, Depression suicidal, Suicidal behavior, Suicide attempt][a]
Conjunctivitis (narrow)	CMQ10644 based on the following PTs [Conjunctivitis, Conjunctivitis allergic, Conjunctivitis bacterial, Conjunctivitis viral, Atopic keratoconjunctivitis][a]
Conjunctivitis (broad)	CMQ10645 based on the following PTs [Conjunctivitis, Conjunctivitis allergic, Conjunctivitis bacterial, Conjunctivitis viral, Atopic keratoconjunctivitis, Blepharitis, Dry eye, Eye irritation, Eye pruritus, Lacrimation increased, Eye discharge, Foreign body sensation in eyes, Photophobia, Xerophthalmia, Ocular hyperaemia, Conjunctival hyperaemia][a]
Conjunctivitis (FDA)[c]	CMQ10643 based on the following PTs [Conjunctivitis, Conjunctivitis allergic, Conjunctivitis bacterial, Conjunctivitis viral, Eye irritation, Eye inflammation, Giant papillary conjunctivitis][a]
Keratitis (FDA) [c]	CMQ30102 based on the following PTs in (keratitis, ulcerative keratitis, allergic keratitis, atopic keratoconjunctivitis, and ophthalmic herpes simplex)

a The list of terms may be adjusted according to MedDRA version changes
b All cases of Eosinophilia will be included in the analysis, where cases associated with clinical symptoms will be further described in the CSR
c FDA requested for US labeling

The following summaries will be provided:

- All TEAEs, by selected standardized MedDRA query (SMQ)/Customized MedDRA query (CMQ) and PT or by laboratory values (as in alanine aminotransferase (ALT) elevation), showing the number (%) of participants with at least 1 PT,
- For each AESI and other selected AE groupings,
  - Number (%) of participants with any specific TEAE
  - Number (%) of participants with any specific serious AE (regardless of treatment emergent status)
  - Number (%) of participants with any specific treatment emergent serious AE
  - Number (%) of participants with any specific AE leading to death
  - Number (%) of participants with any specific TEAE leading to permanent study drug discontinuation
  - Number (%) of participants with any specific TEAE related to IMP reported by investigator
  - Number (%) of participants with any specific TEAE by maximum intensity, corrective treatment, and final outcome
  - Number of any specific TEAE adjusted by the exposure duration
  - Number of participants with any specific TEAE adjusted by the exposure duration at risk. For each specific TEAE, Kaplan-Meier estimates of cumulative incidence at Week 12, and 24 and K-M plot may be provided to depict the course of onset over time if the number of events is large enough.
  - Number (%) of participants with injection site reactions by IMP injection.
  - Number (%) of participants with different number of injection site reactions.
- In addition, AESIs reported by the investigator in eCRF will be summarized separately.

In addition, the exposure-adjusted adverse event incidence rate tables will provide the number of patients with at least 1 event per 100 patient-years for the summaries of TEAEs, treatment emergent SAE, TEAE leading to permanent intervention discontinuation, and AESIs and other selected AE groups in Table 8.

4.7.3 Additional safety assessments

4.7.3.1 Laboratory variables, vital signs and electrocardiograms (ECGs)

The following laboratory variables, vital signs and electrocardiogram (ECG) variables will be analyzed. They will be converted into standard international units.

- Hematology:
  - Red blood cells and platelets and coagulation: hemoglobin, hematocrit, red blood cell count, platelet count

  - White blood cells: white blood cell count, neutrophils, lymphocytes, monocytes, basophils, eosinophils
- Clinical chemistry:
  - Metabolism: glucose, total cholesterol, total protein, creatine phosphokinase
  - Electrolytes: sodium, potassium, chloride, bicarbonate
  - Renal function: creatinine, blood urea nitrogen, uric acid
  - Liver function: alanine aminotransferase, aspartate aminotransferase, alkaline phosphatase, lactate dehydrogenase, total bilirubin, albumin
  - Pregnancy test: Serum β-human chorionic gonadotropin (all female participants) will be performed at screening (V1) in women of childbearing potential, and a urine pregnancy test will be performed at V2 and every 4 weeks thereafter.
  - Hepatitis screen: hepatitis B surface antigen (HBs Ag), hepatitis B surface antibody (HBs Ab), hepatitis B core antibody (HBc Ab), hepatitis C virus antibodies (HCV Ab) will be tested at screening (V1). In case of results showing HBs Ag (negative) and HBc Ab (positive), an hepatitis B virus (HBV) deoxyribonucleic acid (DNA) testing will be performed and should be confirmed negative prior to randomization. In case of results showing HCV Ab (positive), an HCV ribonucleic acid (RNA) testing will be performed and should be confirmed negative prior to randomization.
  - HIV screen: Anti-HIV-1 and HIV-2 antibodies will be tested at Visit 1.
  - TB test (performed locally if required and results noted in the eCRF).
- Urinalysis:
  - Urinalysis will include specific gravity, pH, glucose, ketones, blood, protein, nitrate, leukocyte esterase, urobilinogen and bilirubin. If positive for protein and/or red blood cells, microscopic analysis will be performed by the central laboratory. Creatinine, leukotriene and tetranor PGDM will be tested by the central laboratory.
- Vital signs: pulse rate (beats per minute), systolic and diastolic blood pressure (mmHg) in a semi-supine or sitting position after 5 minutes, weight, respiratory rate (breaths per minute), temperature (degrees Celsius) and height (screening only).
- ECG variables: heart rate, PR, QRS, QT, and QTcF intervals after 10 minutes of rest in the supine position. Data are locally collected and read at screening (V1) and Week 24 (V6).

Data below the lower limit of quantitation/detection limit (LLOQ) will be replaced by half of the LLOQ, data above the upper limit of quantification will be replaced by ULOQ value.

<u>Quantitative analyses</u>

For all laboratory variables, vital signs and ECG variables above, descriptive statistics for results and changes from baseline will be provided for each analysis window, the last value and the worst value during the on-treatment period. These analyses will be performed using central measurements only (when available) for laboratory variables.

For all parameters, mean changes from baseline with the corresponding standard error will be plotted over time.

Analyses according to PCSA

Analysis of potentially clinically significant abnormality (PCSA) will be performed based on the PCSA list in effect at Sanofi at the time of the database lock. For parameters for which no PCSA criteria are defined, similar analyses will be done using the normal range, if applicable.

Analyses according to PCSA will be performed based on the worst value during the treatment-emergent period, using all measurements (either local or central, either scheduled, nonscheduled or repeated).

For laboratory variables, vital signs and ECG variables above, the incidence of participants with at least one PCSA during the treatment-emergent period will be summarized regardless of the baseline level and according to the following baseline status categories:

- Normal/missing
- Abnormal according to PCSA criterion or criteria;

Additional analyses for suspect drug-induced liver injury

The following additional analyses will be performed for drug-induced liver injury:

- Time to onset of the initial ALT or aspartate aminotransferase (AST) elevation (>3 x ULN) and total bilirubin elevation (>2 x ULN) during the treatment-emergent period will be analyzed using Kaplan-Meier method.
- A graph of the distribution of peak values of ALT versus peak values of total bilirubin during the treatment-emergent period will be provided.
- For each liver function test (eg, ALT), participants having a PCSA (eg, ALT >5 ULN) will be summarized using the following categories: Returned to baseline PCSA status (or returned to value ≤ ULN in case of missing baseline) before last IMP dose, Returned to baseline PCSA status after last IMP dose, Never returned to baseline PCSA status, No assessment after elevation. This summary will be performed by categories of elevation (ALT >3, >5, >10, >20 ULN).

4.8 OTHER ANALYSES

4.8.1 Pharmacokinetic analyses

Predose dupilumab concentrations in serum at Visit 2 (Day 1), dupilumab trough concentrations at Week 4, Week 8, Week 12, Week 24/EOT Visit and post-treatment dupilumab concentrations at Week 36/EOS Visit will be provided.

Concentrations of dupilumab in serum will be summarized in the PK population using arithmetic and geometric means, SD, standard error of the mean (SEM), coefficient of variation (CV),

minimum, median and maximum per sampling time. If date and/or time of the drug injection and/or sampling is missing then the concentration will not be taken into account. For drug-treated participants, where concentration values are below the lower limit of quantification (LLOQ), one-half of the LLOQ will be used. Values will be expressed in the tables with no more than three significant figures. For participants in the placebo group, concentration values that are below the LLOQ will be taken into account with a plasma concentration considered equal to 0.

4.8.2 Immunogenicity analyses

Anti-drug antibody (ADA) status to dupilumab (negative or titer value, if positive in the ADA assay) at Visit 2 (Day 1), Week 12, Week 24/EOT and follow up at Week 36 will be provided. The neutralizing antibody status for ADA positive samples will be provided.

Incidence will be provided for the following ADA response categories:

<u>Pre-existing immunoreactivity is defined as:</u>

An ADA positive response in the assay at baseline with all post first dose ADA results negative, OR an ADA positive response at baseline with all post first dose ADA responses less than 4-fold over baseline titer levels.

<u>Treatment-emergent ADA response is defined as:</u>

A positive response in the ADA assay post first dose, when baseline results are negative or missing.

<u>Treatment-emergent ADA responses are further classified as Persistent, Indeterminate or Transient</u>

A) Persistent Response- defined as a treatment-emergent ADA response with two or more consecutive ADA positive sampling time points, separated by greater than (>) 12-week period (84 days), with no ADA negative samples in between.

B) Indeterminate Response- defined as a treatment-emergent response with only the last collected sample positive in the ADA assay

C) Transient Response - defined as a treatment-emergent response that is not considered persistent OR indeterminate

<u>Treatment-boosted response is defined as:</u>

An ADA positive response in the assay post first dose that is greater-than or equal to 4-fold over baseline titer levels, when baseline results are positive.

Titer values (Titer value category)

- Low (Titer <1000)
- Moderate (1000 ≤ Titer ≤10 000)
- High (Titer >10 000)

The following summary will be provided based on ADA population:

- Number (%) of participants with pre-existing immunoreactivity
- Number (%) of participants with treatment-emergent ADA
- The summary statistics (including number, median, Q1, Q3, minimum and maximum) of the peak post-baseline titer for participants with treatment-emergent ADA, and participants with persistent, indeterminate and transient ADA response
- Number (%) of participant with transient treatment-emergent ADA
- Number (%) of participants with persistent treatment-emergent ADA
- Number (%) of participants with indeterminate treatment-emergent ADA
- Number (%) of participants with treatment-boosted ADA
- The summary statistics (including number, median, Q1, Q3, minimum and maximum) of the peak post-baseline titer for participants with treatment-boosted ADA
- The summary statistics (including number, mean, SD, median, Q1, Q3, minimum and maximum) of the ratio of peak post-baseline titer to baseline titer for participants with treatment-boosted ADA
- Listing of ADA peak titer levels and neutralizing antibody status
- Number (%) of participants with neutralizing antibody status.

Kinetics of treatment-emergent ADA response

Number (%) of participants with treatment-emergent ADA positive response at each visit will be summarized, including titer categories (lower, moderate, and high titer), by each intervention group.

A plot of percentage of participants with treatment-emergent ADA positive response at each visit will be provided by each intervention group.

Association of Immunogenicity with Exposure, Safety and Efficacy

The safety and efficacy analyses mentioned below will be conducted using the following categories:

- ADA positive participants: Participants with treatment-emergent or treatment-boosted response.
- ADA negative participants: Participants with pre-existing immunoreactivity or negative in the ADA assay at all time points.

Association of ADA with PK

Associations between ADA variables (eg, ADA peak titers, neutralizing antibody status, treatment-emergent, persistent, indeterminate and transient response, treatment-boosted) and concentration of dupilumab in serum may be explored for each dupilumab dose group. A plot of

concentration of functional dupilumab versus visit will be provided by ADA classifications for the dupilumab dose group. Individual participant plots of dupilumab concentration according to ADA status will be provided to assess the impact of ADA on PK.

Association of ADA with clinical efficacy endpoints

Associations between the ADA variables (eg, ADA peak titers, neutralizing antibody status, treatment-emergent, persistent and treatment-boosted) and the primary efficacy endpoint may be explored for the dupilumab dosed group.

Association of ADA with clinical safety endpoints

Association of safety versus ADA status may be analyzed in the ADA population. The safety assessment may focus on the following events:

- Severe injection site reactions last longer than 24 hours or serious injection site reactions
- Hypersensitivity reactions (SMQ (20000214) hypersensitivity narrow search confirmed by medical review)
- Anaphylactic reactions (SMQ (20000021) anaphylactic reaction narrow search)

Associations between ADA variables (eg, ADA peak titers, neutralizing antibody status, treatment-emergent, persistent and treatment-boosted) and safety may be explored.

4.8.3 Pharmacodynamic/genomics endpoints

Venous blood samples will be collected at Visit 2 (Week 0), Visit 3 (Week 4), Visit 4 (Week 8), Visit 5 (Week 12), Visit 6 (Week 24), and Visit 7 (Week 36), for measurement of total serum IgE. Total IgE will be measured using validated quantitative methods.

For those participants who consent to the optional pharmacogenetic/pharmacogenomic sample collection section of the ICF, blood (serum/plasma) for possible future analysis of potential biomarkers of drug response, disease activity, safety, and the Type 2 inflammation pathway, and blood samples for exploratory genetic analysis of DNA or RNA will be collected and stored for possible future use. Participation is optional. Participants who do not wish to participate in the genetic research may still participate in the study.

For the optional skin biopsy (substudy), the sample will be taken from lesion and non-lesion skin using punch biopsy.

Total IgE will be summarized in the safety population defined as participants who actually received at least 1 dose or part of a dose of the IMP. Baseline values will be the last value collected prior to the first IMP. Descriptive statistics (including number, mean, SD, median, Q1, Q3, min, max) of biomarkers at baseline will be summarized. In addition, for participants who take the prohibited medications/procedures or rescue medications defined in Table 5, the data after the medications will be censored and then last observation carried forward (LOCF) method will be implemented for missing data imputation in the analysis.

Summary plots (median with interquartile range) on values at each visit, absolute changes from baseline and percent changes from baseline will be provided for the total IgE by intervention group and visit.

Exploratory analysis of DNA, RNA and urine biomarkers will be addressed in a separate document.

The analyses of the skin biopsy substudy will be addressed in a separate document.

4.9 INTERIM ANALYSES

No interim analysis is planned.

A primary database lock will be performed when all randomized participants in this study have completed their 24-week treatment phase. Final analyses in the CSR will be based on this database.

The database will be updated at the end of the study for all participants to include the post-treatment follow-up information and updates for the events previously ongoing at the time of the primary lock. Additional data between this database lock and last participant completing last visit will be summarized in a CSR addendum.

5 SUPPORTING DOCUMENTATION

5.1 APPENDIX 1 LIST OF ABBREVIATIONS

ADA:	anti-drug antibody
AE:	adverse event
AESIs:	adverse events of special interest
ALT:	alanine aminotransferase
ANCOVA:	analysis of covariance
AST:	aspartate aminotransferase
ATC:	anatomic category
CDG:	company drug grouping
CI:	confidence interval
CLcr:	Creatinine clearance
CMH:	Cochran-Mantel Haenszel
CSR:	clinical study report
DLQI:	Dermatology Life Quality Index
DNA:	deoxyribonucleic acid
ECG:	electrocardiogram
eCRF:	electronic case report form
EMA:	European Medicines Agency
EOT:	end of treatment
EQ-5D:	Euroqol 5 dimensions
EQ-5D-5L:	5-level EuroQol 5-dimensional questionnaire
FDA:	Food and Drug Administration
HADS:	hospital anxiety and depression scale
HBc Ab:	hepatitis B core antibody
HBs Ab:	hepatitis B surface antibody
HBs Ag:	hepatitis B surface antigen
HBV:	hepatitis B virus
HCV Ab:	hepatitis C virus antibodies
HGLT:	high level group term
HLT:	high level term
HRQoL:	health-related quality-of-life
IGA PN:	Investigator’s global assessment for prurigo nodularis
IGA PN-A:	IGA PN-Activity
IGA PN-S:	IGA PN-Stage
IMP:	investigational medicinal product
ITT:	intent-to-treat
LLT:	lower-level term
LS:	least squares
MedDRA:	medical dictionary for regulatory activities
NRS:	numeric rating scale
PCSA:	potentially clinically significant abnormality

PD: pharmacodynamic
PGIC: Participant Global Impression of Change
PGIS: Participant Global Impression of Severity
PK: pharmacokinetic
PN: prurigo nodularis
PT: preferred term
RNA: ribonucleic acid
SAE: serious adverse event
SAP: statistical analysis plan
SD: standard deviation
SDG: standardized drug grouping
SMQ: standardized MedDRA query
SOC: system organ class
TEAE: treatment-emergent adverse event
ULN: upper limit of normal
VAS: visual analog scale, visual analog scale
WHO-DD: World Health Organization-Drug Dictionary
WI-NRS: worst-itch numeric rating scale
WOCF: worst-observation carried forward

5.2 APPENDIX 2 CHANGES TO PROTOCOL-PLANNED ANALYSES

This section summarizes major statistical changes in the protocol amendments. As mentioned previously, the Sponsor made the decision to complete the study as originally planned instead of resizing the study based on the results of EFC16460 after a health authority’s feedback on Protocol Amendment 2 dated 14-Apr-2021.

Table 9 - Major statistical changes in protocol amendment(s)

Amendment Number	Approval Date	Changes	Rationale
1	20-May-2020	To add “proportion of participants with Investigator’s Global Assessment 0 or 1 score for PN-Stage (IGA PN-S) at Week 24” as another key secondary endpoint	To include a lesion-related key secondary endpoint according to the health authority’s recommendations
		To remove the endpoint “Change from baseline in PAS total score at Week 4, Week 8, Week 12, and Week 24” in the exploratory endpoint and modify the exploratory endpoint regarding the healed lesions from PAS questionnaire analysis	To clarify the analysis on efficacy evaluation of dupilumab on skin lesions using a modified prurigo activity score (PAS) 5-item questionnaire
		To break out the secondary endpoints with multiple measuring timepoints into individual endpoints.	To clearly define the timepoints of each endpoint according to health authority’s recommendation

Amendment Number	Approval Date	Changes	Rationale
		To add the sensitivity analysis for secondary endpoints information, and to separate key secondary endpoints in a different row	To evaluate the robustness of the missing data imputation assumption by sensitivity analyses, and to clarify the endpoints analyses
		To add the covariate “baseline anti-depressant use (yes or no)” to primary and secondary endpoint analyses	To adjust for potential impact of anti-depressant use on the treatment effect in primary and secondary efficacy analyses
		The following wording was added: “Data collected regarding the impact of the COVID-19 or other pandemics, on the participants will be summarized (eg, discontinuation due to COVID-19). Any additional analyses and methods required to investigate the impact of COVID-19 or other pandemics requiring public health emergency on the efficacy (eg, missing data due to COVID-19) and safety will be detailed in the SAP”.	To describe alternative temporary mechanism that can be implemented in the study conduct in case of pandemic requiring public health emergency eg, COVID-19
2	14-Apr-2021	Added 2 more populations for analyses in in the case an earlier primary database lock occurs.<br>The ITT-Week-12 population consists of all participants who had been randomized by 14 June 2021.<br>The ITT-Week-24 population consists of all participants who had been randomized by 22 March 2021.	To detail how the endpoints will be analyzed in the case of an early primary database lock. Based on the timing of the availability of the EFC16460 results, and to ensure a minimum of 135 participants have the opportunity to reach the Week 24 endpoint, the randomization cut-off dates have been assessed as 22 March 2021 for Week 24 and 14 June 2021 for Week 12.
		Added text:<br>The analysis population for the efficacy endpoints will be the ITT population. In the case of an early primary database lock prior to the last enrolled participant reaching EOT, the analysis population will be the ITT-Week-12 population for endpoints which occur earlier than or equal to Week 12. The ITT-Week-12 population consists of all participants who had been randomized by 14 June 2021. The analysis population for Week 24 endpoints will be the ITT-Week-24 population. The ITT-Week-24 population consists of all participants who had been randomized by 22 March 2021.<br>Modification in accordance with the above proposal will be documented in the SAP prior to the database lock.	To provide detail of how data from timepoint evaluations will be handled in the case of an early primary database lock.

Amendment Number	Approval Date	Changes	Rationale
		Added text:<br>In the case of an early primary database lock prior to the last enrolled participant reaching EOT, the analysis of safety will be performed on all available and safety<br>data collected up to the time of the primary database lock.<br>Added text in Table 7:<br>In addition, exposure-adjusted AE incidence rate tables will present by SOC and PT the number of patients with at least 1 event per 100 patient-years.	With an early primary analysis, participants may have different treatment exposure because of study design.
3	21-Oct-2021	To promote the "proportion of participants with improvement (reduction) in worst-itch numeric rating scale (WI-NRS) by ≥4 from baseline to Week 24 as primary endpoint and to move the "proportion of participants with improvement (reduction) in WI-NRS by ≥4 from baseline to Week 12" to a secondary endpoint	Based on the data from EFC16460, the treatment effect of dupilumab continued to improve over time through Week 24. Therefore, the Sponsor proposes to assess the proportion of participants with improvement (reduction) in WI-NRS by ≥4 at Week 24, which represents the effect more accurately and synchronizes the primary itch assessment with the primary lesion assessment
		To update the sample size calculation based on the observed effect sizes from EFC16460.	To assess a timepoint that reflects the optimal dupilumab treatment effect as a primary endpoint
		To update the primary analysis considerations to the Week 24 timepoint.	To be consistent with primary endpoint change
		To remove the sentence "A key secondary endpoint will be the responder analyses of itch improvement of at least 4 points at Week 24." as it is now the primary endpoint, and to provide rationale for choosing the primary endpoint timepoint at Week 24 with the following language: "The timing of the primary assessment, ie, at Week 24, was based on the results of the primary analysis of EFC16460, showing that the effect of dupilumab over time showed continuous improvement after Week 12 across all endpoints, with a similar time course of improvement in both itch and lesion endpoints through at least Week 24. Since improvement of itch and lesions may occur prior to Week 24, responder analyses assessments will be performed at earlier time points as well, starting at Week 2 for itch, and Week 4 for lesions."	To provide rationale and maintain consistency in language with the change in the primary endpoint.

Amendment Number	Approval Date	Changes	Rationale
		To specify that the rationale for a 24-week duration of the trial is an appropriate duration based on data observed from EFC16460 and to remove references to atopic dermatitis trials.	To reflect that data from EFC16460 has informed our understanding of the dupilumab treatment effect continuing to improve itch through Week 24. The Week 24 timepoint most accurately represents the overall treatment effect and synchronizes the primary itch assessment with the primary lesion assessment.
		To reflect that the primary endpoint timepoint is Week 24.	To maintain consistency in language with the change in the primary endpoint.
		To update Table 6 to reflect that the primary endpoint is assessed at Week 24.	To maintain consistency in language with the change in the primary endpoint.
		To remove the possibility of re-evaluating the timing of the primary database lock of EFC16459 based on the observed treatment effect size in EFC16460, that was added per Amendment 02. And to consequently remove the 2 populations (ITT-Week 12 and ITT-Week 24) that were added per Amendment 02.	Amended protocol 02 was submitted in few countries but was withdrawn following a Health Authority feedback and was not implemented in any country.
		Subsection 10.12.2 was added and the overall rationale and table with summary of changes for Amended protocol 02 were moved from the cover page to this section.	This is aligned with Sanofi procedures.

5.3 APPENDIX 3 DEMOGRAPHICS AND BASELINE CHARACTERISTICS, PRIOR OR CONCOMITANT MEDICATIONS/PROCEDURES

Demographics, baseline characteristics, medical surgical history

The following demographics and baseline characteristics, medical and surgical history and disease characteristics at baseline will be summarized using descriptive statistics in the randomized population.

Demographic variables are

- Age in years (quantitative and qualitative variable: 18-<40, 40–<65, 65-<75, and ≥75 years),
- Gender (Male, Female),
- Race (White, Black or African American, Asian, American Indian or Alaska Native, Native Hawaiian or other Pacific Island, unknown),
- Ethnicity (Hispanic or Latino, Not Hispanic or Latino, Unknown),
- Region (Asia: Japan, China, South Korea; East Europe: Russia; Latin America: Argentina, Mexico; Western Countries: USA, France),

- Territory (North America: USA; European Union: France; Rest of World: Russia, Japan, China, South Korea, Argentina, Mexico),
- Weight in kg (quantitative and qualitative variable: (<60, ≥60- <90, ≥90 kg),
- BMI in kg/m^2 (quantitative and qualitative variable: (<25, ≥25- <30, ≥30 kg/m^2).

Baseline safety and efficacy parameters (apart from those listed above) will be presented along with the safety and efficacy summaries.

Medical (or surgical) history includes all the relevant medical (or surgical) history during the lifetime of the participant.

This information will be coded using the version of MedDRA currently in effect at Sanofi at the time of database lock.

Comorbidity will be summarized separately. The following comorbid diseases will be summarized from electronic case report form (eCRF) pages which were filled in by investigators based on participant reporting.

- Prurigo nodularis (Yes, Ongoing condition)
- Atopic dermatitis (Yes, Ongoing condition)
- Allergic rhinitis history (Yes, Ongoing condition)
- Allergic rhinoconjunctitvitis (Yes, Ongoing condition)
- Asthma history (Yes, Ongoing condition)
- Food allergy history (Yes, Ongoing condition)
- Eosinophilic esophagitis history (Yes, Ongoing condition).

Disease characteristics at baseline

The following baseline disease characteristics will be summarized by intervention group:

- Duration of PN and grouping (years; <3 and ≥3) to be derived as
  - (Year of randomization – Year of first diagnosis of PN) + (month of randomization - month of first diagnosis of PN)/12
- Age of onset of PN (years)
- History of atopy (atopic or non-atopic)
  - Number of mild AD participants under atopic
- Stable use of TCS/TCI (yes or no)
- Baseline WI-NRS score
- Baseline IGA PN-S score (summary and frequency by each score level)
- Baseline IGA PN-A score (summary and frequency by each score level)

- Baseline skin Pain-NRS score
- Baseline Sleep-NRS score
- Baseline PGIS score
- PAS (number of prurigo lesions in a total and in representative area, and healed prurigo lesions) at baseline
- Baseline Hospital Anxiety and Depression Scale (HADS) total score
  - Baseline Hospital Anxiety score
  - Baseline Depression Scale score
- Baseline Dermatology Life Quality Index (DLQI) score
- Baseline EuroQol five dimensions questionnaire (ED-5D-5L) (single index score and VAS)
- Frequency of alcohol drinking in the past 12 months (never, occasional, at least monthly, at least weekly, at least daily) and number of drinks on a typical day (1 or 2, >2)
- Antidepressant use (yes or no) at baseline
- HIV (positive versus negative).

Prior or concomitant medications and procedure

All medications will be coded using the World Health Organization-Drug Dictionary (WHO-DD) using the version currently in effect at Sanofi at the time of database lock.

All procedures will be coded to a PT and associated primary SOC using the version of MedDRA currently in effect at Sanofi at the time of database lock.

- Prior medications/procedures are those the participant used prior to first investigational medicinal product (IMP) injection. Prior medications/procedures can be discontinued before first administration or can be ongoing during treatment phase.
- Concomitant medications/procedures are any interventions received by the participant concomitantly to the IMP, from first administration of IMP to last IMP intake + 98 days.
- Post-treatment medications/procedures are those the participant took in the period running from the end of the concomitant medications period up to the end of the study.
- A given medication/procedure can be classified as a prior medication/procedure and/or as a concomitant medication/procedure and/or as post-treatment medication/procedure. If it cannot be determined whether a given medication/procedure was taken prior or concomitantly or post, it will be considered as prior, concomitant, and post-treatment medication/procedure.

The prior and concomitant medications/procedure will be summarized for the randomized population.

Medications will be summarized by intervention group according to the WHO-DD dictionary, considering the first digit of the anatomic category (ATC) class (anatomic category) and the first 3 digits of the ATC class (therapeutic category). All ATC codes corresponding to a medication will be summarized, and participants will be counted once in each ATC category (anatomic or therapeutic) linked to the medication. Therefore, participants may be counted several time for the same medication.

The table for prior medications will be sorted by decreasing frequency of ATC followed by all other therapeutic classes based on the overall incidence across intervention groups. In case of equal frequency regarding ATCs (anatomic or therapeutic categories), alphabetical order will be used.

Concomitant medication received during first IMP to last IMP +14 days and concomitant medication received during first IMP to last IMP +98 days will be summarized separately. The tables for concomitant medications will be sorted by decreasing frequency of ATC followed by all other therapeutic classes based on the incidence in the dupilumab group. In case of equal frequency regarding ATCs (anatomic or therapeutic categories), alphabetical order will be used.

Medications will also be summarized by generic name sorted by decreasing frequency based on the incidence in the dupilumab group.

Procedures will be summarized by intervention group by primary SOC (sorted by internationally agreed order) and PT (sorted in alphabetical order), sorting is based on the overall incidence across intervention groups.

Background intervention

Participants will be required to apply moisturizers (emollients) once or twice daily for at least the 7 consecutive days immediately before baseline (Day 1) and continue until Week 36.

The compliance of moisturizers (emollients) used from 7 days before the baseline visit to Week 36 (or end of study), which is defined as the (number of days moisturizers used during the period)/(number of days within the period) x 100%, will be summarized by intervention group.

Similarly, the compliance of moisturizers (emollients) used from the baseline visit to Week 12 and Week 24 will be summarized by intervention group respectively.

In addition, the compliance of use of TCS/TCI from the baseline visit to Week 12 and Week 24 for participants with the stratification of stable use of TCS/TCI (yes) will be summarized by intervention group respectively.

Prohibited medications/procedures

The concomitant use of the following therapies is prohibited during the entire study. Study treatment will need to be discontinued in participants receiving these treatments:

- Systemic immunosuppressive/immunomodulating drugs (eg, systemic corticosteroids, cyclosporine, mycophenolate-mofetil, interferon gamma, Janus kinase inhibitors, azathioprine, methotrexate, hydroxychloroquine, dapsone, sulfasalazine, colchine, etc)
- Other monoclonal antibodies (which are biological modifiers)
- Phototherapy, including tanning beds
- Naltrexone or other opioid antagonist
- Gabapentin, pregabalin, and thalidomide

The concomitant use of the following therapies is prohibited except if the dose has been stable for at least 3 months prior to screening, but study treatment will not need to be discontinued in participants receiving the treatments listed below. The dose should also remain stable (can be reduced or discontinued if medically indicated) and should not be increased during the study.

- Paroxetine, fluvoxamine, or other SSRIs
- SNRIs
- Amitriptyline or other tricyclic or tetracyclic antidepressants

The concomitant use of the following therapies is also prohibited during the entire study, but study treatment will not need to be discontinued in participants receiving the treatments listed below:

- Intralesional corticosteroid injections and cryotherapy
- Sedating antihistamine
- Non-sedating antihistamine if used specifically for the treatment of itch secondary to AD or PN.

The number and percentage of participants who take the prohibited medications/procedures will be provided. In addition, the time (week) of first prohibited medication/procedure taken will also be analyzed using Kaplan-Meier method.

Rescue medications

The following rescue medications may be used:

- Dermatological preparations of high potency or superpotent TCS and TCI.

If medically necessary (ie, to control intolerable PN symptoms), rescue treatment for PN may be provided to study participants at the discretion of the Investigator.

Although the use of rescue medications is allowed at any time during the study, the use of rescue medications should be delayed, if possible, for at least 14 days following the initiation of the investigational treatment. The date and time of rescue medication administration as well as the name and dosage regimen of the rescue medication must be recorded in the eCRF.

- For the purpose of the efficacy responder analysis, a pre-specified algorithm will be used to classify rescue. In addition, a blinded review of all post-baseline medications to adjudicate rescue treatment, based on medical judgment, will be performed to adjudicate rescue. Participants who receive rescue treatment as per this adjudication during the study will be considered treatment failures.

The rescue medications will be analyzed by the same method as that used for prohibited medications/procedures.

5.4 APPENDIX 4 DATA HANDLING CONVENTIONS

Demographic formulas

Age of onset of PN is calculated as:

Year of PN diagnosis – Year of birth

BMI is calculated as:

$$\text{Weight in kg} / (\text{height}^2 \text{ in meters})$$

Renal function formulas

For adults, creatinine clearance (CLcr) value will be derived using the equation of Cockroft and Gault:

$$\text{CLcr (ml/min)} = (140 - \text{age}) \times \text{weight (kg)} \times (1 - 0.15 \times \text{sex (0-M, 1-F)}) / (0.814 \times \text{creatinine } (\mu\text{mol/l}))$$

CLcr will be calculated using the last weight measurement on or before the visit of the creatinine measurement and age at the lab sampling day. Here age is calculated as following:

Age = age collected at screening + integer part of (lab sampling analysis day/365.25)

Analysis windows for time points

Efficacy assessment

For the efficacy assessment, the reference date for the derivation of relative days of events or findings will be the randomization day. If a participant receives IMP prior to the randomization by mistake, the reference date of efficacy assessment will be the date of the first IMP administration for that participant.

For daily eDiary data (WI-NRS, skin Pain-NRS, Sleep-NRS), all available values of daily measurements will be assigned to each week window according to Table 10, and then weekly average score will be calculated. Randomization day is used as the reference day (Day 1).

Table 10 - Time window for eDiary efficacy variables

Time	Target day	Day range for calculating weekly score
Baseline (Week 0)	1	-7- <1
Week 1	8	1-11
Week 2	15	12-18
Week 3	22	19-25
Week 4	29	26-32
Week 5	36	33-39
Week 6	43	40-46
Week 7	50	47-53
Week 8	57	54-60
Week 9	64	61-67
Week 10	71	68-74
Week 11	78	75-81
Week 12	85	82-88
Week 13	92	89-95
Week 14	99	96-102
Week 15	106	103-109
Week 16	113	110-116
Week 17	120	117-123
Week 18	127	124-130
Week 19	134	131-137
Week 20	141	138-144
Week 21	148	145-151
Week 22	155	152-158
Week 23	162	159-165
Week 24	169	166-172
Week 25	176	173-179
Week 26	183	180-186
Week 27	190	187-193
Week 28	197	194-200
Week 29	204	201-207
Week 30	211	208-214
Week 31	218	215-221
Week 32	225	222-228
Week 33	232	229-235

VV-CLIN-0604231 5.0

Time	Target day	Day range for calculating weekly score
Week 34	239	236-242
Week 35	246	243-249
Week 36	253	250-256

For other efficacy variables, all available values of scheduled measurements will be assigned to the appropriate visit window according to Table 11. In the event of multiple measurements of the same test in the same window, the one closest to the targeted visit date will be used for the by-visit summary. If they are at the same number of days away from the target day, the latest one will be used.

Table 11 - Time window for efficacy variables

		Time windows for				
Visit	Target Day	DLQI, IGA PN-A/PN-S	HADS, EQ-5D-5L, Missed school/work days	PGIS	PGIC	PAS
Visit 1 (Week -4 to -2)	<1			<-14		<-14
Visit 2 (Week 0)	1	1^-	1^-	$-14-1^-$		$-14-1^-$
Visit 3 (Week 4)	29	1^+-42		1^+-42	1^+-42	1^+-42
Visit 4 (Week 8)	57	43-70		43-70	43-70	43-70
Visit 5 (Week 12)	85	71-126	1^+-126	71-126	71-126	71-126
Visit 6 (Week 24)	169	127-210	127-210	>126	>126	127-210
Visit 7 (Week 36)	253	>210	>210			>210

1^-: up to randomization and before 1st dose date/time; 1^+: after randomization or 1st dose date/time

Safety assessment

For the safety assessment, the reference date for the derivation of relative days of events or findings will be the date of first IMP administration. Selected safety variables will be summarized by the analysis window define in Table 12 for the by visit descriptive analysis. All available values from central lab will be assigned to the appropriate visit window. In the event of multiple measurements of the same test in the same window, the one closest to the targeted visit date will be used for the by-visit summary. If they are at the same number of days away from the target day, the latest one will be used. For procedures planned on Visit 2, if it is done on the same date as the first IMP injection but the performance time is missing, it will belong to Visit 2 time window.

Table 12 - Time window for safety endpoints

		Time windows for							
Visit	Target Day	Vital signs	Hematology, biochemistry	Urinalysis	CD4 T cell count and HIV test	Serum Pregnancy test	Urine Pregnancy test	Physical examination	ECG
Visit 1 (Week -4 to -2)	<1	<-14	<-14	<-14	1^-	<-14		<-14	1^-
Visit 2 (Week 0)	1	-14-1^-	-14-1^-	-14-1^-			-14-1^-	-14-1^-	
Visit 3 (Week 4)	29	1^+-42	1^+-42	1^+-42			1^+-42		
Visit 4 (Week 8)	57	43-70	43-70	43-70			43-70		
Visit 5 (Week 12)	85	71-126	71-126	71-126			71-126		
Visit 6 (Week 24)	169	127-210	127-210	>126	1^+		127-210	1^+-210	1^+
Visit 7(Week 36)	253	>210	>210				>210	>210	

1^-: up to 1st dose date/time; 1^+: after 1st dose date/time;

Pharmacokinetics/pharmacodynamics assessment

For the pharmacokinetics/pharmacodynamics variables summary, the reference date for the derivation of relative days of measurements will be the date of first IMP administration if the participant is treated with study treatment, or the randomization date if the participant is not treated. Pharmacokinetics/pharmacodynamics variables will be summarized by the analysis window defined in Table 13 for the by visit descriptive analyses. All available values of measurements will be assigned to the appropriate visit window. In the event of multiple measurements of the same test in the same window, the one closest to the targeted visit date will be used for the by-visit summary. If they are at the same distance to the target day, the latest one will be used. For procedures planned on Visit 2, if it is done on the same date as the first IMP injection but the performance time is missing, it will belong to Visit 2 time window.

Table 13 - Time window for pharmacokinetics/pharmacodynamics variables

		Time windows for		
**Visit**	**Target day**	**Serum dupilumab concentration**	**Anti-drug antibodies**	**Total IgE**
Visit 1 (Week -4)	<1			
Visit 2 (Week 0)	1	1^-	1^-	1^-
Visit 3 (Week 4)	29	1^+-42		1^+-42
Visit 4 (Week 8)	57	43-70		43-70
Visit 5 (Week 12)	85	71-126	1^+-126	71-126
Visit 6 (Week 24)	169	127-210	127-210	127-210
Visit 7(Week 36)	253	>210	>210	>210

1^-: up to 1st dose date/time or randomization if participant is not treated; 1^+: after 1st dose date/time or randomization date if participant is not treated;

Unscheduled visits

Unscheduled visit measurements of laboratory data, vital signs, and ECG will be used for computation of baseline, the last on-treatment value, analysis according to PCSAs, and the shift summaries for safety. They will also be included in the by-visit summaries if they are re-allocated to scheduled visits.

5.5 APPENDIX 5 SAMPLE SAS CODE

The multiple imputation and analysis model for the primary analysis of change from baseline in WI-NRS at Week 24 will be built with the following sample SAS code.

1. 40 datasets with a monotone missing pattern will be obtained, induced by Markov Chain Monte Carlo (MCMC) method on participants who have not taken the prohibited medications and/or rescue medications or have discontinued study treatment due to lack of efficacy prior to Week 24.

```
proc mi data=dat_etd seed=16460 nimpute=40 out=dat_mc;
    mcmc impute=monotone;
    var atopicyn stableyn region antidblyn trt01p winrsbl chg1winrs …
    chg24winrs;
run;
```

2. For each of the imputed dataset with monotone missing pattern in step 1, the remaining missing data will be imputed using the regression method for the monotone pattern with adjustment for covariates including intervention groups, documented history of atopy (atopic or non-atopic), stable use of TCS/TCI (yes or no), region, baseline anti-depressant use (yes or no), and baseline value of the response variable.

```
proc mi data=dat_mc nimpute=1 seed=16461 out=dat_mi;
    by _imputation_;
    class atopicyn stableyn region antidblyn trt01p;
    monotone method=reg;
    var atopicyn stableyn region antidblyn trt01p winrsbl chg1winrs …
    chg24winrs;
run;
```

3. Each of the 40 imputed datasets will be merged with the one dataset imputed by WOCF approach, and then be analyzed using the main statistical model. These 40 imputed datasets will be saved.

```
%macro w1;
     %do i=1%to 40;
     data wocf&i.;
     set wocf;
     _imputation_=&i.;
     run;
     %end;
     data wocf_all;
     set %do j=1 %to 40; wocf&j. %end;;
     run;
%mend w1;

%w1

data dat_imp;
     set dat_mi  wocf_all;
Run;

proc sort data=dat_imp;
     by _imputation_;
run;

proc glm data= dat_imp;
     by  imputation ;
     class atopicyn stableyn region antidblyn trt01p;
```

```
          model  chg24winrs = atopicyn stableyn region antidblyn
                   trt01p winrsbl;
          lsmeans trt01p / stderr;
          estimate 'Diff Dupilumab vs Placebo' trt01p -1 1;
          ods output LSMeans=implsmeans Estimates=implsmeandiff;
     run;
```

4. Applying Rubin's rule to combine analysis results (point estimates and standard errors) from 40 imputations using PROC MIANALYZE for the LS means and difference in LS means between dupilumab and placebo. Sample code:

```
proc sort data=implsMeans; by trt01pn _imputation_;run;

proc mianalyze data= implsmeans;
     by trt01pn;
     modeleffects lsmean;
     stderr stderr;
     ods output ParameterEstimates=lsmeans;
run;

proc mianalyze data=implsmeandiff;
     modeleffects estimate;
     stderr stderr;
     ods output ParameterEstimates=lsmeandiff;

run;
```

5.6 APPENDIX 6 SELECTION CRITERIA FOR AE/MEDICATION GROUPINGS

Table 14 - List of PTs or Medications for CMQs/CDGs

Grouping	Preferred Term/Medication Code	Preferred Term/Medication
Conjunctivitis	10001257	Adenoviral conjunctivitis
Conjunctivitis	10010725	Conjunctival irritation
Conjunctivitis	10010726	Conjunctival oedema
Conjunctivitis	10010736	Conjunctival ulcer
Conjunctivitis	10010741	Conjunctivitis
Conjunctivitis	10010744	Conjunctivitis allergic
Conjunctivitis	10010745	Conjunctivitis chlamydial
Conjunctivitis	10010749	Conjunctivitis gonococcal neonatal
Conjunctivitis	10010754	Conjunctivitis tuberculous
Conjunctivitis	10010755	Conjunctivitis viral
Conjunctivitis	10018258	Giant papillary conjunctivitis

Grouping	Preferred Term/Medication Code	Preferred Term/Medication
Conjunctivitis	10021629	Inclusion conjunctivitis
Conjunctivitis	10030861	Ophthalmia neonatorum
Conjunctivitis	10048908	Seasonal allergy
Conjunctivitis	10049458	Herpes simplex virus conjunctivitis neonatal
Conjunctivitis	10051625	Conjunctival hyperaemia
Conjunctivitis	10053991	Inclusion conjunctivitis neonatal
Conjunctivitis	10061784	Conjunctivitis bacterial
Conjunctivitis	10062889	Pingueculitis
Conjunctivitis	10063669	Photoelectric conjunctivitis
Conjunctivitis	10067317	Oculorespiratory syndrome
Conjunctivitis	10067817	Acute haemorrhagic conjunctivitis
Conjunctivitis	10069166	Blebitis
Conjunctivitis	10071570	Ligneous conjunctivitis
Conjunctivitis	10074701	Noninfective conjunctivitis
Conjunctivitis	10075264	Oculoglandular syndrome
Conjunctivitis	10080825	Conjunctivitis fungal
Conjunctivitis	10084034	Conjunctival suffusion
Intravenous immunoglobulin therapy	CAS 8000012671	IMMUNOGLOBULIN HUMAN NORMAL
Intravenous immunoglobulin therapy	CAS 8000050682	IMMUNOGLOBULIN, PORCINE
Intravenous immunoglobulin therapy	CAS 8000056919	IMMUNOGLOBULIN G HUMAN
Intravenous immunoglobulin therapy	CAS 8600000563	IMMUNOGLOBULINS NOS
Intravenous immunoglobulin therapy	CAS 8600001670	IMMUNOGLOBULIN HUMAN NORMAL SLRA
Intravenous immunoglobulin therapy	CAS 8600001671	IMMUNOGLOBULIN HUMAN NORMAL IFAS
Intravenous immunoglobulin therapy	RECNO 900708	OTHER IMMUNOGLOBULINS
Intravenous immunoglobulin therapy	RECNO 900722	IMMUNE SERA AND IMMUNOGLOBULINS
Intravenous immunoglobulin therapy	RECNO 900728	IMMUNOGLOBULINS
Intravenous immunoglobulin therapy	RECNO 900914	SPECIFIC IMMUNOGLOBULINS
Intravenous immunoglobulin therapy	RECNO 901112	IMMUNOGLOBULINS, NORMAL HUMAN

Abbreviations: CAS : Chemical Abstract Service Registry Number RECNO : Drug Record Number

VV-CLIN-0604231 5.0

5.7 APPENDIX 7 MEDICATION/PROCEDURE ADJUDICATION ALGORITHM

Algorithm for determining whether treatment with following medications or procedures constitutes treatment failure resulting in setting data as non-responder after taking medication or undergoing procedure in the main statistical analysis

1. Not required to adjudicate post-baseline medications (WHODD-coded) or procedures (CMQ-coded) as these will be considered treatment failures if used at any time[1].
   a) Always considered treatment failure
      - ATC2 = CORTICOSTEROIDS FOR SYSTEMIC USE
      - ATC2=IMMUNOSUPPRESSANTS (systemic, eg, oral or parenteral route)
      - Preferred Drug Name = Ciclosporin
      - Preferred Drug Name = Methotrexate
      - Preferred Drug Name = Mycophenolate sodium
      - Preferred Drug Name = Mycophenolic acid
      - Preferred Drug Name = Azathioprine
      - Preferred Drug Name = Gabapentin
      - Preferred Drug Name = Pregabalin
      - Preferred Drug Name = Thalidomide
      - Preferred Drug Name = Lenalidomide
      - Preferred Drug Name = Baricitinib
      - Preferred Drug Name = Ruxolitinib
      - Preferred Drug Name = Tofacitinib
      - Preferred Drug Name = AbrocitinibPreferred Drug Name = Delgocitinib
      - Preferred Drug Name = Nalbuphine
      - Preferred Drug Name = Naltrexone
      - Preferred Drug Name = Naloxone
      - Preferred Drug Name = Vixarelimab
      - Preferred Drug Name = Nemolizumab
      - Preferred Drug Name = Serlopitant
      - Preferred Drug Name = Aprepitant
      - CMQb HLT Phototherapies
      - CMQ Tanning_single PT

   b) Never considered treatment failure:
      - ATC2 = EMOLLIENTS AND PROTECTIVES

- ATC2 = GENERAL NUTRIENTS
- ATC2 = VITAMINS
- ATC2 = ANTIVIRALS FOR SYSTEMIC USE
- ATC2 = ANTIFUNGALS FOR DERMATOLOGICAL USE
- ATC2 = ANTISEPTICS AND DISINFECTANTS
- ATC2 = ANTIBIOTICS AND CHEMOTHERAPEUTICS FOR DERMATOLOGICAL USE
- ATC2 = ANTI-ACNE PREPARATIONS, excluding D10AA Corticosteroids, combinations for treatment of acne
- ATC2 = OPHTHALMOLOGICALS
- ATC1 = ANTIINFECTIVES FOR SYSTEMIC USE, excluding D07C Corticosteroids, combinations with antibiotics
- ATC1 = BLOOD AND BLOOD FORMING ORGANS
- ATC1 = ALIMENTARY TRACT AND METABOLISM
- ATC1 = MUSCULO-SKELETAL SYSTEM, excluding M01BA Antiinflammatory/antirheumatic agents in combination with corticosteroids
- ATC2 = COUGH AND COLD PREPARATIONS
- ATC2= PSYCHOLEPTICS, excluding N05BB Diphenylmethane derivatives and N05C HYPNOTICS AND SEDATIVES

1 A blinded review of all post-baseline medications or procedures to adjudicate whether treatment constitutes treatment failure, based on medical judgment, may be performed in addition. A listing of treatments or procedures classified as treatment failure in a manner inconsistent with the classification under #1 will be provided, along with supporting rationale.

2. Require to adjudicate medications or procedures that may constitute treatment failure

- All other medications and procedures listed in the protocol and Table 5 of the SAP (not noted in 1. above) given for indications consistent with PN[2]
- Considerations in determining treatment failure include the criteria already set forth in Table 5 of the SAP in addition to the type of medication or procedure, indication, dose, route of administration, timing, frequency and the potential impact of the use of the prohibited medications/procedures and rescue medications on the primary and key secondary efficacy endpoints.

2 Below is a list of indications consistent with PN based on PT level from concomitant medication/procedure data using MedDRA dictionary

System Organ Class	High Level Term	Preferred Term	Preferred Term Code
Skin and subcutaneous tissue disorders	Dermatitis and eczema	Neurodermatitis	10029263
Infections and infestations	Bacterial infections NEC	Eczema impetiginous	10051890
Infections and infestations	Skin structures and soft tissue infections	Dermatitis infected	10012470
Infections and infestations	Skin structures and soft tissue infections	Eczema infected	10014199
Skin and subcutaneous tissue disorders	Dermatitis and eczema	Dermatitis	10012431
Skin and subcutaneous tissue disorders	Dermatitis and eczema	Dermatitis atopic	10012438
Skin and subcutaneous tissue disorders	Dermatitis and eczema	Eczema	10014184

Signature Page for VV-CLIN-0602722 v5.0
efc16459-16-1-9-sap

Approve & eSign	[redacted]

Approve & eSign	[redacted]

CLINICAL TRIAL PROTOCOL

**Protocol title:**	**A randomized, double blind, placebo-controlled, multi-center, parallel group study to evaluate the efficacy and safety of dupilumab in patients with prurigo nodularis who are inadequately controlled on topical prescription therapies or when those therapies are not advisable**
**Protocol number:**	**EFC16460**
**Amendment number:**	**Not applicable**
**Compound number (INN/Trademark):**	**SAR231893/REGN668 dupilumab/Dupixent**
**Study phase:**	**Phase 3**
**Short title:**	**Study of dupilumab for the treatment of patients with prurigo nodularis, inadequately controlled on topical prescription therapies or when those therapies are not advisable**<br>**LIBERTY-PN PRIME2**
**Sponsor name:**	
**Legal registered address:**	
**Monitoring Team's Representative Name and Contact Information**	

Regulatory agency identifier number(s):

IND:	IND107969
EudraCT:	2019-003801-90
NCT:	Not applicable
WHO:	Not applicable
Other:	Not applicable

Approval Date: 30-Oct-2019 Total number of pages: 101

Any and all information presented in this document shall be treated as confidential and shall remain the exclusive property of Sanofi (or any of its affiliated companies). The use of such confidential information must be restricted to the recipient for the agreed purpose and must not be disclosed, published or otherwise communicated to any unauthorized persons, for any reason, in any form whatsoever without the prior written consent of Sanofi (or the concerned affiliated company); 'affiliated company' means any corporation, partnership or other entity which at the date of communication or afterwards (i) controls directly or indirectly Sanofi, (ii) is directly or indirectly controlled by Sanofi, with 'control' meaning direct or indirect ownership of more than 50% of the capital stock or the voting rights in such corporation, partnership or other entity

TABLE OF CONTENTS

CLINICAL TRIAL PROTOCOL **1**

TABLE OF CONTENTS **2**

LIST OF TABLES **6**

1 PROTOCOL SUMMARY **7**

1.1 SYNOPSIS 7

1.2 SCHEMA 13

1.3 SCHEDULE OF ACTIVITIES (SOA) 14

2 INTRODUCTION **19**

2.1 STUDY RATIONALE 19

2.2 BACKGROUND 19

2.3 BENEFIT/RISK ASSESSMENT 21

3 OBJECTIVES AND ENDPOINTS **24**

3.1 APPROPRIATENESS OF MEASUREMENTS 25

4 STUDY DESIGN **27**

4.1 OVERALL DESIGN 27

4.2 SCIENTIFIC RATIONALE FOR STUDY DESIGN 27

4.3 JUSTIFICATION FOR DOSE 28

4.4 END OF STUDY DEFINITION 29

5 STUDY POPULATION **30**

5.1 INCLUSION CRITERIA 30

5.2 EXCLUSION CRITERIA 32

5.3 LIFESTYLE CONSIDERATIONS 35

5.4 SCREEN FAILURES 36

6 STUDY INTERVENTION **37**

6.1 STUDY INTERVENTION(S) ADMINISTERED 37
6.1.1 Noninvestigational medicinal products 38

6.2 PREPARATION/HANDLING/STORAGE/ACCOUNTABILITY 39

6.2.1 Storage and handling 39
6.2.2 Responsibilities 39

6.3 MEASURES TO MINIMIZE BIAS: RANDOMIZATION AND BLINDING 39

6.4 STUDY INTERVENTION COMPLIANCE 41

6.5 CONCOMITANT THERAPY 41
6.5.1 Rescue medicine....42

6.6 DOSE MODIFICATION....42

6.7 INTERVENTION AFTER THE END OF THE STUDY 42

7 DISCONTINUATION OF STUDY INTERVENTION AND PARTICIPANT DISCONTINUATION/WITHDRAWAL 43

7.1 DISCONTINUATION OF STUDY INTERVENTION 43
7.1.1 Definitive discontinuation 43
7.1.2 Temporary discontinuation....44
7.1.2.1 Rechallenge 45

7.2 PARTICIPANT DISCONTINUATION/WITHDRAWAL FROM THE STUDY....45

7.3 LOST TO FOLLOW UP 46

8 STUDY ASSESSMENTS AND PROCEDURES 47

8.1 EFFICACY ASSESSMENTS 47
8.1.1 Worst-itch numeric rating scale....48
8.1.2 Investigator's global assessment for prurigo nodularis....48
8.1.3 Prurigo activity score....48
8.1.4 Dermatology life quality index 49
8.1.5 Pain and sleep numeric rating scales 49
8.1.6 Hospital anxiety and depression scale....49
8.1.7 Patient Global Impression of Change of disease and Patient Global Impression of Severity 50
8.1.8 Euroqol 5 dimensions questionnaire....50
8.1.9 Missed school/work days 50

8.2 SAFETY ASSESSMENTS 51
8.2.1 Physical examinations 51
8.2.2 Vital signs....51
8.2.3 Electrocardiograms 51
8.2.4 Clinical safety laboratory assessments....51

8.3 ADVERSE EVENTS AND SERIOUS ADVERSE EVENTS....52

8.3.1 Time period and frequency for collecting AE and SAE information 53
8.3.2 Method of detecting AEs and SAEs 54
8.3.3 Follow-up of AEs and SAEs 54
8.3.4 Regulatory reporting requirements for SAEs 54
8.3.5 Pregnancy 54
8.3.6 Guidelines for reporting product complaints 55

8.4 TREATMENT OF OVERDOSE 55

8.5 PHARMACOKINETICS 55
8.5.1 Systemic drug concentration and antidrug antibodies 55
8.5.1.1 Sampling time 55
8.5.1.2 Handling procedures 56
8.5.1.3 Bioanalytic method 56

8.6 PHARMACODYNAMICS 57

8.7 GENETICS 57

8.8 BIOMARKERS 57

8.9 MEDICAL RESOURCE UTILIZATION AND HEALTH ECONOMICS 57

9 STATISTICAL CONSIDERATIONS 58

9.1 STATISTICAL HYPOTHESES 58

9.2 SAMPLE SIZE DETERMINATION 58

9.3 POPULATIONS FOR ANALYSES 59

9.4 STATISTICAL ANALYSES 59
9.4.1 Efficacy analyses 59
9.4.2 Safety analyses 61
9.4.3 Other analyses 62

9.5 INTERIM ANALYSES 62
9.5.1 Data Monitoring Committee (DMC) 62
9.5.2 Unblinding plan 62

10 SUPPORTING DOCUMENTATION AND OPERATIONAL CONSIDERATIONS 63

10.1 APPENDIX 1: REGULATORY, ETHICAL, AND STUDY OVERSIGHT CONSIDERATIONS 63
10.1.1 Regulatory and Ethical Considerations 63
10.1.2 Financial disclosure 63
10.1.3 Informed consent process 64
10.1.4 Data protection 64

10.1.5 Committees structure 65
10.1.6 Dissemination of clinical study data 65
10.1.7 Data quality assurance 66
10.1.8 Source documents 66
10.1.9 Study and site closure 67
10.1.10 Publication policy 67

10.2 APPENDIX 2: CLINICAL LABORATORY TESTS 68

10.3 APPENDIX 3: ADVERSE EVENTS: DEFINITIONS AND PROCEDURES FOR RECORDING, EVALUATING, FOLLOW-UP, AND REPORTING 70

10.4 APPENDIX 4: CONTRACEPTIVE GUIDANCE AND COLLECTION OF PREGNANCY INFORMATION 74

10.5 APPENDIX 5: GENETICS 77

10.6 APPENDIX 6: LIVER AND OTHER SAFETY: SUGGESTED ACTIONS AND FOLLOW-UP ASSESSMENTS 78

10.7 APPENDIX 7: CLINICIANS-REPORTED OUTCOMES AND PATIENT-REPORTED OUTCOMES 81
10.7.1 Worst itch numeric rating scale (WI-NRS) 81
10.7.2 Investigator's global assessment of prurigo nodularis (IGA PN) 82
10.7.3 Prurigo activity score (PAS) 83
10.7.4 Dermatology life quality index (DLQI) 85
10.7.5 Hospital anxiety and depression scale (HADS) 86
10.7.6 EQ-5D-5L 87
10.7.7 Pain numeric rating scale 88
10.7.8 Sleep numeric rating scale 89
10.7.9 Patient Global Impression of Change of disease (PGIC) 89
10.7.10 Patient Global Impression of Severity (PGIS) 90
10.7.11 Missed school/work days 91

10.8 APPENDIX 8: DEFINITION OF ANAPHYLAXIS 95

10.9 APPENDIX 9: LIST OF OPPORTUNISTIC INFECTIONS 95

10.10 APPENDIX 10: COUNTRY-SPECIFIC REQUIREMENTS 96

10.11 APPENDIX 11: ABBREVIATIONS 96

11 REFERENCES 99

LIST OF TABLES

Table 1 - Objectives and endpoints....24
Table 2 - Overview of study interventions administered 37
Table 3 - Summary of handling procedures 56
Table 4 - Summary of bioanalytical methods for functional dupilumab and anti-dupilumab antibodies 56
Table 5 - Populations for analyses 59
Table 6 - Efficacy analyses 60
Table 7 - Safety analyses....61
Table 8 - Other analyses 62
Table 9 - Protocol-required laboratory assessments 68

1 PROTOCOL SUMMARY

1.1 SYNOPSIS

Protocol title: A randomized, double blind, placebo-controlled, multi-center, parallel group study to evaluate the efficacy and safety of dupilumab in patients with prurigo nodularis who are inadequately controlled on topical prescription therapies or when those therapies are not advisable

Short title: Study of dupilumab for the treatment of patients with prurigo nodularis, inadequately controlled on topical prescription therapies or when those therapies are not advisable

LIBERTY-PN PRIME2 (PRurigo Nodularis Itch Minimization Evaluation 2)

Rationale:

Prurigo nodularis (PN) is a skin disease characterized by multiple, intensely itchy skin eruptions in symmetrically distributed areas of the extremities (1). The main symptom is prolonged, repetitive and uncontrollable rubbing, scratching and uncontrollable itching which leads to hyperkeratotic eroding papules and nodules on the skin. It is difficult to treat and entails a high disease burden. Approximately 50% of patients have either past or current history of atopic dermatitis (AD) or other atopic disorders (2). While case reports suggest that dupilumab may successfully treat patients with PN regardless of a preexisting atopic background (3, 4, 5, 6, 7), it remains unclear to what extent Type 2 cytokines play a role in the pathogenesis in those PN lesions not associated with atopy (8).

There are no United States (US) Food and Drug Administration (FDA) or European Medicines Agency (EMA) approved targeted therapies indicated for the treatment of PN, whether atopic or non-atopic forms. Topical corticosteroids (TCS) and topical calcineurin inhibitors (TCI) are often used initially. While there is a mechanistic rationale for their use, no rigorous clinical studies confirming their efficacy were identified.

Dupilumab is a human monoclonal IgG4 antibody that inhibits interleukin (IL)-4 and IL-13 signaling by specifically binding to the IL-4Rα subunit shared by the IL-4 and IL-13 receptor complexes. Dupilumab inhibits IL-4 signaling via the Type I receptor and both IL-4 and IL-13 signaling through the Type II receptor.

Dupilumab has shown clinical efficacy in multiple diseases with underlying Type 2 inflammation such as AD, asthma, chronic rhinosinusitis with nasal polyposis (CRSwNP), and eosinophilic esophagitis (EoE).

Recently, case series were published that suggest dupilumab may also be effective in the treatment of PN (3, 4, 5, 6, 7).

Type 2 cytokine involvement is implicated to some extent in all forms of PN. It is postulated that dupilumab may play a fundamental role in pruritus based upon its ability to block IL4R activity at the level of the sensory dorsal root ganglion (9). The present protocol will evaluate the efficacy and safety of dupilumab in PN.

Objectives and endpoints

Objectives	Endpoints
**Primary**	
• To demonstrate the efficacy of dupilumab on itch response in patients with PN, inadequately controlled on topical prescription therapies or when those therapies are not advisable.	• Proportion of participants with improvement (reduction) in worst-itch numeric rating scale (WI-NRS) by ≥4 from baseline to Week 12.
**Secondary**	
• To demonstrate the efficacy of dupilumab on additional itch endpoints in patients with PN, inadequately controlled on topical prescription therapies or when those therapies are not advisable.	• **[Key secondary endpoint]** Proportion of participants with improvement (reduction) in WI-NRS by ≥4 from baseline to Week 24.<br>• Time to onset of effect on pruritus as measured by proportion of participants with an improvement (reduction) in WI-NRS by ≥4 from baseline during the 24-week treatment period.<br>• Change from baseline in WI-NRS at Week 12 and Week 24.<br>• Percent change from baseline in WI-NRS at Week 12 and Week 24.<br>• Percent change from baseline in WI-NRS over time until Week 24.<br>• Proportion of participants with WI-NRS reduction ≥4 over time until Week 24.<br>• Onset of action in change from baseline in WI-NRS (first $p < 0.05$ difference from placebo in the daily WI-NRS that remains significant at subsequent measurements) until Week 12.
• To demonstrate efficacy of dupilumab on skin lesions of PN.	• Proportion of participants with Investigator's Global Assessment 0/1 score for PN-Stage (IGA PN-S) at Week 4, Week 8, Week 12, and Week 24.<br>• Change from baseline in IGA PN-S score at Week 4, Week 8, Week 12, and Week 24.<br>• Proportion of participants with Investigator's Global Assessment 0/1 score for PN-Assess (IGA PN-A) at Week 4, Week 8, Week 12, and Week 24.
• To demonstrate the improvement in health-related quality of life (HRQoL).	• Change from baseline in HRQoL, as measured by Dermatology Life Quality Index (DLQI) to Week 12 and Week 24.
• To evaluate safety outcome measures.	• Percentage of participants experiencing treatment-emergent adverse events (TEAEs) or serious adverse events (SAEs) from baseline through Week 24.
• To evaluate immunogenicity of dupilumab.	• Incidence of treatment-emergent antidrug antibodies (ADA) against dupilumab over time.

Overall design:

This study is a multi-center, 24-week treatment, parallel, double-blind, randomized, placebo-controlled study to evaluate the use of dupilumab in patients with PN inadequately controlled on topical prescription therapies or when those therapies are not advisable. The study will assess the effect of dupilumab on itch improvement as well as its effect on PN lesions, on patients' HRQoL, anxiety and depression, sleep quality and skin pain, and overall health status.

Disclosure Statement: This is a Parallel, Treatment study, with 2 arms, that is blinded/masked for participants and investigators.

Number of participants:

Approximately 150 participants will be randomized 1:1. This corresponds to approximately 75 participants who will be randomly assigned to each intervention arm.

Intervention groups and duration:

Participants who satisfy the inclusion and exclusion criteria will be randomized (1:1) to one of the following investigational medicinal product (IMP) treatment groups:

- Dupilumab 300 mg
- Matched placebo

Duration of study period (per participant)

- Screening period (2-4 weeks)
- Randomized IMP intervention period (24 weeks)
- Follow-up period (12 weeks)

Study interventions

Investigational medicinal product:

Dupilumab 300 mg and placebo matching dupilumab 300 mg supplied in prefilled syringes that are visually indistinguishable.

Dupilumab

- Formulation: dupilumab 300 mg: a 150 mg/mL dupilumab solution in a pre-filled syringe to deliver 300 mg in a 2 mL injection.
- Route of administration: subcutaneous (SC) injection.
- Dose regimen: 300 mg every 2 weeks (Q2W) after an initial loading dose of 600 mg (2 injections of 300 mg) on Day 1.

Placebo

- Formulation: identical formulation to the active 300 mg formulation without dupilumab, in a pre-filled syringe to deliver placebo in a 2 mL injection.
- Route of administration: SC injection.
- Dose regimen: 1 injection Q2W after an initial loading dose (2 injections) on Day 1.

Noninvestigational medicinal products

Participants will be required to apply moisturizers (emollients) once or twice daily for at least the 7 consecutive days immediately before Day 1 and continue until Week 36.

If participants are on a stable regimen of low to medium potency TCS or TCI at the screening visit, they can continue their topical steroid application once daily without tapering from Screening to Week 24. If specific lesions resolve, the participant can stop applying steroids to those sites but are permitted to continue applying to persistent lesions. If participants are on stable regimens of high potency or superpotent steroids, participants should decrease potency to medium potency TCS and continue to apply daily from screening to Week 24. Occlusion is not allowed from Screening to Week 24.

Participants can be rescued with high potency or superpotent TCS/TCI as needed throughout the study.

Post-trial access to study medication

The sponsor does not plan to provide post-trial access to the study medication.

Statistical considerations:

- **Randomization**
  - The participants will be randomized to dupilumab or placebo in 1:1 ratio with stratification factors of documented history of atopy (atopic or non-atopic), stable use of TCS/TCI (yes or no), and country/territory code. Number of participants with active mild AD upon study entry will represent up to 10% of the atopic participants. Both the atopic and the non-atopic PN population will be capped at 60% of the total enrolled population.
- **Sample size calculation**
  - The primary endpoint is the proportion of participants with a WI-NRS reduction of ≥4 from baseline to Week 12. Assuming a response rate of [redacted]% and [redacted]% in placebo and dupilumab respectively, 56 participants/arm will provide 90% power to detect the difference of [redacted]% between dupilumab and placebo with Fisher exact test at 2-sided level of 0.05. Assuming 15% drop out during the 12 weeks of treatment, the target is to randomize 75 participants/arm to allow for up to 10% of atopic patients having active mild AD upon study entry.

- **Primary analysis**
  - The analysis population for the efficacy endpoints will be the intent-to-treat (ITT) population defined as all randomized participants analyzed according to the treatment group allocated by randomization regardless if treatment kit is used or not.
  - The primary analysis on WI-NRS reduction ≥4 at Week 12 will be conducted by using Cochran-Mantel-Haenszel (CMH) test stratifying by stratification factors of documented history of atopy (atopic or non-atopic), stable use of TCS/TCI (yes or no), and region (countries combined). For participants discontinuing the study treatment before Week 12, their off-study treatment values measured up to Week 12 will be included in the analysis. Participants taking selected prohibited medications and/or rescue medications (details of selection will be specified in the statistical analysis plan [SAP]) prior to Week 12 or have missing data at Week 12 will be considered non-responders.
  - Sensitivity analyses using alternative methods will be performed to handle missing data and/or data collected after participants taking selected prohibited medications and/or rescue medications. A subgroup analysis will be performed excluding participants with a current diagnosis of AD. More details of the sensitivity and subgroup analyses will be specified in the SAP.
- **Analysis of secondary endpoints**
  - Secondary efficacy endpoints that measure binary responses will be analyzed in the same fashion as the primary endpoint.
  - Time-to-event secondary efficacy endpoint will be analyzed using the Cox proportional hazards model, including treatment, stratification factors (countries combined to region). The hazards ratio, its 95% confidence interval and p-value will be reported. Kaplan-Meier curves will be also provided.
  - Continuous secondary efficacy endpoints will be analyzed using a hybrid method of the worst observation carried forward (WOCF) and multiple imputations. Data of participants taking selected prohibited medications and/or rescue medications will be set to missing after the medication usage, and the worst postbaseline value on or before the time of the medication usage will be used to impute missing endpoint value (for participants whose postbaseline values are all missing, the baseline will be used to impute). Participants who discontinue the treatment prematurely are encouraged to follow the planned clinical visits and in those participants who did not take the selected prohibited medications and/or rescue medications, all data collected after treatment discontinuation will be used in the analysis. For these participants, missing data may still happen despite all efforts have been tried to collect the data after treatment discontinuation. For participants who discontinue due to lack of efficacy, all data collected after discontinuation will be used in the analysis, and a WOCF approach will be used to impute missing data if needed. For participants who discontinued not due to lack of efficacy, a multiple imputation approach will be used to impute missing endpoint value, and this multiple imputation will use all participants excluding participants who have taken the selected prohibited medications and/or rescue medications prior to timepoint of endpoint of interest and excluding patients who

discontinue due to lack of efficacy. Each of the imputed complete data will be analyzed by fitting an analysis of covariance (ANCOVA) model with treatment group, stratification factors (countries combined into region) and relevant baseline measurement as covariates in the model. Statistical inference obtained from all imputed data will be combined using Rubin's rule.

- The safety variables will be summarized using descriptive statistics.
- Antidrug antibodies variables including treatment-emergent ADA will be summarized using descriptive statistics by treatment group.

- **Multiplicity considerations**
  - The multiplicity procedure is proposed to control the overall Type-I error rate for testing the primary endpoint and the key secondary endpoints. Detailed hierarchical testing procedure will be defined in the study SAP. The study is considered positive when the primary endpoint achieves statistical significance.
- **Planned database lock date**
  - A primary database lock will be performed when all randomized participants have completed their 24-week treatment phase.
  - The database will be updated at the end of the study for all participants to include the post-treatment follow-up information and updates for the events previously ongoing at the time of the primary lock.
- **Unblinding plan**
  - The unblinding plan will be detailed in the SAP or in a separate unblinding plan.

Data Monitoring Committee: No

1.2 SCHEMA

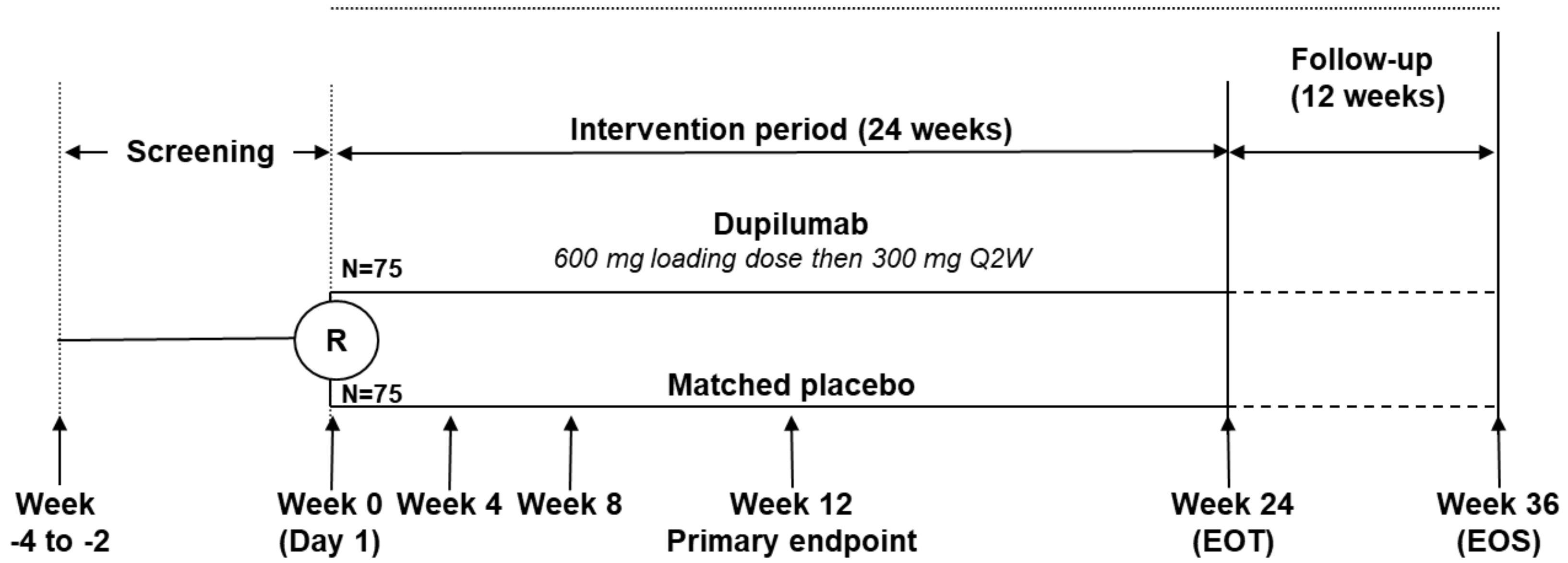

Background: low to medium potency TCS/TCI as background therapy permitted (maintain dose from screening to EOT)

Dupilumab 300 mg Q2W, administered as 1 SC injection of dupilumab 300 mg (2 mL)
A 600 mg loading dose will be administered on Day 1.
Matched placebo is prepared in the same formulation without the addition of protein (ie, the active substance)

EOS: end of study; EOT: end of treatment; PN: prurigo nodularis; Q2W: every 2 weeks; R: randomization; SC: subcutaneous; TCI: topical calcineurin inhibitors; TCS: topical corticosteroids.

1.3 SCHEDULE OF ACTIVITIES (SOA)

Procedure	Screening (2 to 4 weeks before Day 1)	Intervention period (Weeks)					Follow-up (12 weeks)	Notes
		0 (Day 1)	4	8	12	24		
**Visit**	**1**	**2**[a]	**3**	**4**	**5**	**6**[b] **(EOT)**	**7 (EOS)**	Visit window: ±3 days for Visits 3, 4, 5, 6, and 7.
**Screening and baseline**								
Informed consent	X							Separate consent to be obtained for optional procedures (DNA and RNA sampling, and serum/plasma sampling for archival; HIV test if specific consent locally required).
Medical history	X							
Prior and concomitant medication	X	X	X	X	X	X	X	Concomitant medication will be collected throughout the study.
Demography	X							
Inclusion and exclusion criteria	X	X						
Hepatitis[c], HIV serology, TB test	X							HBs Ag, HBs Ab, HBc Ab, HCV Ab, HIV screen (Anti-HIV-1 and HIV-2 antibodies), TB test (performed locally if required and results noted in the eCRF).
Randomization		X						
**Study intervention**[d]								
Dispense or download e-diary	X	X	X	X	X	X	X	Device will be dispensed at Screening (including instructions for use). At EOS, the e-diary will be downloaded and returned to the site.
Participant e-diary training	X	X						
Call IVRS/IWRS	X	X	X	X	X	X	X	At screening visit, IVRS/IWRS will be called after medical history check.

Procedure	Screening (2 to 4 weeks before Day 1)	Intervention period (Weeks)					Follow-up (12 weeks)	Notes
		0 (Day 1)	4	8	12	24		
**Visit**	1	2[a]	3	4	5	6[b] (EOT)	7 (EOS)	Visit window: ±3 days for Visits 3, 4, 5, 6, and 7.
IMP administration		X	X	X	X			IMP administration Q2W (±3 days) from Week 0 until Week 22. Between visits, the participants will self-inject IMP at home or may choose to have injections administered at home by a caregiver or at the study site.
**Safety**[d]								
Physical examination	X	X				X	X	Including skin (full body skin exam), nasal cavities, eyes, ears, respiratory, cardiovascular, gastrointestinal, neurological, lymphatic, and musculoskeletal systems.
Vital signs	X	X	X	X	X	X	X	Including SBP and DBP (mmHg), pulse rate (beats per minute), body temperature (°C), and respiratory rate. Body weight will be recorded at Visits 1, 6, and 7 only. Height will be recorded at Visit 1 only.
12-lead ECG	X					X		Locally collected and read.
Hematology, biochemistry[e]	X	X	X	X	X	X	X	Including hemoglobin, hematocrit, platelet count, total WBC count, differential count, total RBC count, creatinine, BUN, glucose, lactate dehydrogenase, uric acid, total cholesterol, total protein, albumin, total bilirubin, ALT, AST, alkaline phosphatase, electrolytes (sodium, potassium, chloride), bicarbonate, and creatine phosphokinase.
Urinalysis	X	X	X	X	X	X		Dipstick analysis including specific gravity, pH, glucose, ketones, blood, protein, nitrite, leukocyte esterase, urobilinogen and bilirubin. If positive for protein and/or red blood cells, microscopic analysis will be performed by the central laboratory. Creatinine and leukotriene will be tested by the central laboratory.
CD4 T cell count and HIV viral load	X					X		For participants with HIV history only.

Procedure	Screening (2 to 4 weeks before Day 1)	Intervention period (Weeks)					Follow-up (12 weeks)	Notes
		0 (Day 1)	4	8	12	24		
**Visit**	**1**	**2**[a]	**3**	**4**	**5**	**6**[b] **(EOT)**	**7 (EOS)**	Visit window: ±3 days for Visits 3, 4, 5, 6, and 7.
Pregnancy test (WOCBP only)[g]	Serum	Urine	Urine	Urine	Urine	Urine	Urine	In between visit urine pregnancy tests will be performed at home (Weeks 16, 20, 28, 32).
AE reporting, including SAEs	X	X	X	X	X	X	X	
Rescue medication use	←--→							
**PK and ADA**[d, f]								
Serum PK samples for dupilumab concentration		X	X	X	X	X	X	
Anti-dupilumab antibody		X			X	X	X	
**Biomarkers**[d]								
Total serum IgE		X	X	X	X	X	X	
Optional serum/plasma for archival samples		X	X	X	X	X	X	Archive serum and plasma samples will be collected and stored for possible future analysis of potential biomarkers of drug response, disease activity, safety, and the Type 2 inflammation pathway.
Optional DNA (whole blood) sample[h]		X						
Optional RNA (whole blood) sample[h]		X				X		
**Efficacy**[d]								
WI-NRS	NRS: once a day from Screening to EOS							To be recorded once a day in e-diary.
Pain-NRS								
Sleep-NRS								

Procedure	Screening (2 to 4 weeks before Day 1)	Intervention period (Weeks)					Follow-up (12 weeks)	Notes
		0 (Day 1)	4	8	12	24		
**Visit**	1	2[a]	3	4	5	6[b] (EOT)	7 (EOS)	Visit window: ±3 days for Visits 3, 4, 5, 6, and 7.
DLQI		X	X	X	X	X	X	Entered by participant during site visit on a tablet provided to the site.
HADS		X			X	X	X	
EQ-5D-5L		X			X	X	X	
PGIS	X	X	X	X	X	X		
PGIC			X	X	X	X		
Missed school/work days (baseline version)		X						
Missed school/work days (postbaseline version)					X	X	X	
Modified PAS (screening-baseline version)	X	X						Investigator's assessment to be entered on a tablet provided to the site.
Modified PAS (follow-up version)			X	X	X	X	X	
IGA PN-A and IGA PN-S		X	X	X	X	X	X	

a All assessments at Visit 2 (Day 1) are to be conducted pre-IMP dose with the exception of the assessment of local tolerability of SC injections.

b Participants who discontinue the study treatment prematurely (prior to completing the 24-week treatment period) will perform the EOT assessments at the time of discontinuation to assure a complete clinical assessment in close temporal proximity to the premature termination of study treatment. In addition, to allow assessment of participant outcomes over the stipulated study period, participants will be asked and encouraged to complete all remaining study visits and participate in all assessments according to the visit schedule.

c In case of results showing HBs Ag (negative), HBs Ab (negative) and HBc Ab (positive), an HBV DNA testing may be performed prior to randomization to rule out a false positivity if the Investigator believes the participant is a false positive, or to clarify the serological status if the Investigator finds it unclear to interpret in absence of known HBV infection. In case of results showing HCV Ab (positive), an HCV RNA testing may be performed to rule out a false positivity, if the participant is a false positive per Investigator's judgement.

d Assessments/procedures should be conducted in the following order: PRO, investigator assessments, safety and laboratory assessments (including sample collection for ADA, PK, biomarker, and optional DNA and RNA), and administration of IMP.

e Refer to Section 10.2 and central lab manual for collection details.

f In the event of any SAE, any AE of severe injection site reaction lasting longer than 24 hours, or any AESI of anaphylactic reaction or systemic allergic reaction that is related to IMP and require treatment, PK and ADA samples will be collected at or near the onset of the event for any additional analysis if required or for archival purposes.

g Pregnancy will lead to definitive treatment discontinuation in all cases. Female participants will be supplied with urine dipsticks for use between visits.

h DNA sample should be collected at the Day 1 visit, but can be collected at any visit during the study. RNA samples must be collected before the administration of the first dose of study drug and at Week 24.

ADA: antidrug antibodies; AE: adverse event; AESI: adverse event of special interest; ALT: alanine aminotransferase; AST: aspartate aminotransferase; BUN: blood urea nitrogen; DBP: diastolic blood pressure; DLQI: Dermatology Life Quality Index; DNA: deoxyribonucleic acid; ECG: electrocardiogram; eCRF: electronic case report form; EOS: end of study; EOT: end of treatment; EQ-5D-5L: Euroqol 5 dimensions 5 levels; HADS: Hospital Anxiety and Depression Scale; HBc Ab: hepatitis B core antibody; HBs Ab: hepatitis B surface antibody; HBs Ag: hepatitis B surface antigen; HBV: hepatitis B virus; HCV: hepatitis C virus; HCV Ab: hepatitis C virus antibody; HIV: human immunodeficiency virus; Ig: immunoglobulin; IGA PN: Investigator's Global Assessment for prurigo nodularis; IGA PN-A: IGA PN-Activity; IGA PN-S: IGA PN-Stage; IMP: Investigational Medicinal Product; IVRS: interactive voice response system; IWRS: interactive web response system; NRS: numeric rating scale; PAS: prurigo activity score; PGIC: Patient Global Impression of Change; PGIS: Patient Global Impression of Severity; PK: pharmacokinetic; PRO: patient reported outcome; Q2W: every 2 weeks; RBC: red blood cells; RNA: ribonucleic acid; SAE: serious adverse event; SBP: systolic blood pressure; SC: subcutaneous; TB: tuberculosis; WBC: white blood cells; WI-NRS: worst itch numeric rating scale; WOCBP: woman of childbearing potential.

2 INTRODUCTION

Dupilumab is a human monoclonal IgG4 antibody that inhibits IL-4 and IL-13 signaling by specifically binding to the IL-4Rα subunit shared by the IL-4 and IL-13 receptor complexes. Dupilumab inhibits IL-4 signaling via the Type I receptor and both IL-4 and IL-13 signaling through the Type II receptor. Blocking IL-4Rα with dupilumab inhibits IL-4 and IL-13, key cytokines that drive Type 2 inflammatory responses, including the release of proinflammatory cytokines, chemokines, nitric oxide, and IgE.

Dupilumab is approved by major regulatory agencies for use in AD and in asthma in adults and adolescents, and in the US for CRSwNP in adults.

2.1 STUDY RATIONALE

Prurigo nodularis is a skin disease characterized by multiple, intensely itchy skin eruptions in symmetrically distributed areas of the extremities (1). It is difficult to treat and entails a high disease burden.

Dupilumab has shown efficacy in multiple diseases with underlying Type 2 inflammation such as AD, asthma, CRSwNP and EoE. In light of the published research suggesting a principal role for Type 2 cytokines in the pathogenesis of PN (see Section 2.2), the Sponsor proposes that selective and simultaneous inhibition of IL-4 and IL-13 signaling such as that achieved with dupilumab treatment may provide clinical benefit in relieving the signs and symptoms of PN, especially in the high unmet need population of patients inadequately treated with, or ineligible for, TCS.

Recently, case series were published that suggest that dupilumab may also be effective in the treatment of PN (3, 4, 5, 6). Type 2 cytokine involvement is implicated in all forms of PN. This is consistent with a recent case series of nine patients without an atopic history who benefitted from dupilumab treatment (7).

The primary hypothesis of the study is that dupilumab will be effective in reducing itch and the secondary hypothesis is that it will reduce and promote the healing of lesions of PN, in patients who are inadequate responders to topical prescription therapies or when those therapies are not advisable.

2.2 BACKGROUND

Prurigo nodularis is a skin disease characterized by multiple, intensely itchy skin eruptions in symmetrically distributed areas of the extremities (1). The main symptom is prolonged, repetitive and uncontrollable rubbing, scratching and uncontrollable itching which leads to hyperkeratotic eroding papules and nodules on the skin. A broadly accepted definition for chronic prurigo has been published by the European Academy of Dermatology and Venereology (EADV) (10). The convened experts agreed that chronic prurigo should be used as an umbrella term for the range of clinical manifestations (eg, papular, nodular, plaque or umbilicated types). Prurigo nodularis is

considered a distinct disease defined by the presence of chronic pruritus for ≥6 weeks, history and/or signs of repeated scratching and multiple localized/generalized pruriginous skin lesions (whitish, hyperpigmented, or pink papules, nodules and/or plaques).

Pathophysiological studies in PN patients reveal increased dermal nerve fiber density and changes in many types of skin cells. These skin cells cause inflammation and pruritus (severe, constant itching) through the release of cytokine IL-31, tryptase, prostaglandins, eosinophil cationic protein, histamine, and neuropeptides, such as substance P, calcitonin gene-related peptide and nerve growth factor (11). The itch caused by primary dermatoses or systemic disorders is postulated to cause an itch scratch cycle which leads to neuronal hypersensitization. This neuronal hypersensitization is the final common pathway which drives the symptomatology of PN (1). Fukushi et al. studied 22 PN skin biopsies and found that 86% of them showed activation of STAT6, a marker for Type 2 inflammatory pathway activation downstream of the Type 2 cytokines IL-4 and IL-13 (12). Consistent with this, IL-4 and IL-5 messenger RNA expression is upregulated in the skin of PN patients (13). Importantly, these two studies did not mention whether these skin biopsy specimens came from patients with or without atopy. The etiology of the profound pruritus in this disease is thought to be driven by high Type 2 inflammation driving activity at the level of the sensory neuron. Human sensory neurons in skin that transmit the sensation of itch are known to express IL- 4Rα, IL-13Ra1, and IL-31RA (14). On testing for functional stimulation, IL-4 was shown to enhance responsiveness to other pruritogens like histamine (14). These findings support that Type 2 cytokines may play a principal role in the pathogenesis of PN.

Due to the central manifestation of itch, PN carries a significant burden of disease (10, 15). The effect on quality of life due to PN has been reported to be higher than other common skin disorders like AD and psoriasis (16). Patients report chronic sleep loss due to constant itching; constant burning, stinging, and pain at affected area; and chronic depression, anxiety, anger, disgust, and shame; and hence overall experience a great impact on their quality of life. According to a 5 year cross-sectional study on 909 adult PN patients in the John Hopkins hospital system (15), PN is a key contributing factor to mood disorders such as anxiety and depression. Of all patients with chronic pruritus, 70% have some type of psychiatric comorbidity, and the severity of depression in patients with chronic pruritus correlates with intensity of itch. The concomitant psychologic component of PN likely contributes to the challenging nature of treating this disease.

Data on the epidemiology of PN are limited. Most studies show a predominance of older patients with a median age of more than 50 years (2, 10, 15). Prurigo nodularis is only occasionally observed in younger patients, in whom it is often associated with atopic conditions (17).

There are no FDA or EMA approved targeted therapies indicated for the treatment of PN, whether atopic or non-atopic forms; the lesions of atopic and non-atopic PN are clinically indistinguishable and treatment options remain the same. Topical corticosteroids and TCI are often used initially. While there is a mechanistic rationale for their use, no rigorous clinical studies confirming their efficacy were identified. Anecdotal feedback obtained from dermatological experts indicate variable, modest efficacy. For thicker lesions, TCS is also administered intralesionally. Lesional cryotherapy is another available topical treatment; case reports indicate temporary relief. Antihistamines and antileukotrienes are occasionally used; their efficacy, however, is not supported by well conducted, randomized clinical trials and is rated low

by patients (18). Phototherapy, in particular narrowband ultraviolet B (UVB), is occasionally added in patients not responding to topical pharmacotherapy. While some improvements with phototherapy have been reported, they remain usually partial and complete remissions are uncommon.

Oral immunosuppressants such as methotrexate and cyclosporine have been used off-label with some success as reported based on case reports and retrospective (18). Use of cyclosporine in PN is limited by commonly recognized toxicities including hypertension, impaired renal and hepatic function, and potential for increased susceptibility to infections and cancer, particularly skin cancer, due to decreased cancer immunosurveillance (19). Methotrexate has well established toxicities, in particular, myelosuppression and hepatotoxicity. In addition, the broad immunosuppression caused by all these drugs carries an increased risk of developing serious bacterial, fungal, viral, and mycobacterial infections.

Neuromodulatory agents such as gabapentin and anti-inflammatory agents such as thalidomide have been used in PN with varying degrees of success (18), but have also considerable adverse effects. Adverse effects of thalidomide include peripheral neuropathies, sedation, dizziness and teratogenicity, while adverse effects of gabapentin include headache, sedation and dizziness.

Overall, despite the use of multiple treatments, many patients with PN remain uncontrolled, and some of the available therapies are associated with serious potential adverse reactions. Given the lack of targeted treatments and the suboptimal efficacy associated with currently available therapies, there remains a significant unmet need in patients with PN.

Dupilumab has shown efficacy in multiple diseases with underlying Type 2 inflammation such as AD, asthma, CRSwNP, and EoE. The most compelling data suggesting that PN is driven by Type 2 inflammation are the many case series of PN patients successfully treated with dupilumab. Recently, five case series were published that suggest that dupilumab may also be effective in the treatment of PN: Mollanazar et al. (4 patients, 3 of those without history of AD) (3), Beck et al. (3 patients) (4), Rambhia et al. (2 patients) (5), Calugareanu et al. (one patient) (6), and Zhai et al. (9 patients, all without history of AD) (7). These case series reported major improvements in treatment-refractory cases of PN after treatment with dupilumab 300 mg Q2W (after an initial loading dose of 600 mg). These published case reports show rapid and substantial effects of dupilumab on itch, and somewhat less well documented effects also on lesions in patients with PN refractory to standard treatment.

2.3 BENEFIT/RISK ASSESSMENT

Dupilumab solution for injection is currently authorized:

- In over 40 countries worldwide including the US, European Union (EU) (Centralised Procedure), and Japan for the treatment of adults with inadequately controlled moderate-to-severe AD. In the US and EU, it has also been authorized for use in adolescent patients (≥12 years) with inadequately controlled moderate-to-severe AD.

- In the US for use in moderate-to-severe eosinophilic or oral steroid dependent asthma, in the EU for severe Type 2 asthma in adults and adolescents, and in Japan for use in adults and adolescents (≥12 years) with severe or refractory bronchial asthma,
- In the US for use in adults with inadequately controlled CRSwNP.

Dupilumab has shown clinically relevant benefit in several Type 2 driven immunological disorders such as AD, bronchial asthma, CRSwNP, and EoE. In asthma and AD indications, studies were also conducted in adolescents and similar benefit to adults was observed.

While the mechanism of PN is not well-known, there is some evidence of a Type 2 immunologic signature in prurigo nodule skin biopsy specimens and activation of Type 2 transcription factors STAT3 and STAT6 in the epidermis (12, 13). Therefore, it is hypothesized that dupilumab may show significant clinical benefit in the treatment of PN. This hypothesis is supported by a series of case reports that show dupilumab treatment resulting in a significant reduction in pruritus in 19 PN patients (3, 4, 5, 6, 7).

No tissue targets or specific hazards to humans were identified in nonclinical general and reproductive toxicology studies.

Dupilumab has an extensive safety database. As of the 28 March 2019 (data lock point), 8798 subjects were enrolled into the development program for dupilumab and are included in the safety population treated: 242 as healthy volunteers, 4227 from AD studies, 3377 from asthma studies, 782 from CRSwNP studies, 53 from EoE studies, 117 from grass allergy and peanut allergy studies. The number of subjects exposed to dupilumab in clinical studies was 7781 (218 in healthy volunteer studies, 3931 in AD studies, 3073 in asthma studies, 470 in CRSwNP studies, 26 in EoE studies, and 63 in grass allergy and peanut allergy studies). Based on the sales figure retrieved from Intercontinental Marketing Services Health and using the World Health Organization’s defined average daily dose for dupilumab of 21.4 mg, the cumulative post marketing exposure to dupilumab is estimated to be 38 816 patient years (01 January 2017 through 31 December 2018).

Dupilumab was generally well tolerated in all populations tested in clinical development programs. The adverse drug reactions (ADRs) identified to date for dupilumab include injection site reactions, conjunctivitis, oral herpes, conjunctivitis allergic, conjunctivitis bacterial, herpes simplex, blepharitis, dry eye, eye pruritus, and eosinophilia. These ADRs occur with relatively low frequency with dupilumab treatment, and were generally mild or moderate, transient, and manageable. More significant serious allergic reactions were very rare. Importantly, no increased overall infection risk was observed in patients treated with dupilumab.

Systemic hypersensitivity has been established as an important identified risk with dupilumab. As protein therapeutics, all monoclonal antibodies are potentially immunogenic. Rare serious and systemic hypersensitivity reactions have been observed in the dupilumab program including serum sickness/serum sickness-like reaction in the adult AD program and anaphylaxis related to dupilumab in the adult asthma clinical trials.

It is hypothesized that dupilumab in patients with PN will have a favorable safety profile as observed across other Type 2-driven immunological disorders.

The safety data available to date, in conjunction with the risk monitoring and mitigation strategies in the study protocol, and the clinical benefit of dupilumab demonstrated in multiple Type 2 indications (AD, asthma, CRSwNP) and case reports and series in PN so far, support a favorable benefit-risk profile for dupilumab.

A risk-benefit statement with respect to the overall development program is provided in the investigator's brochure (IB).

3 OBJECTIVES AND ENDPOINTS

Table 1 - Objectives and endpoints

Objectives	Endpoints
**Primary**	
• To demonstrate the efficacy of dupilumab on itch response in patients with PN, inadequately controlled on topical prescription therapies or when those therapies are not advisable.	• Proportion of participants with improvement (reduction) in worst-itch numeric rating scale (WI-NRS) by ≥4 from baseline to Week 12.
**Secondary**	
• To demonstrate the efficacy of dupilumab on additional itch endpoints in patients with PN, inadequately controlled on topical prescription therapies or when those therapies are not advisable.	• **[Key secondary endpoint]** Proportion of participants with improvement (reduction) in WI-NRS by ≥4 from baseline to Week 24.<br>• Time to onset of effect on pruritus as measured by proportion of participants with an improvement (reduction) in WI-NRS by ≥4 from baseline during the 24-week treatment period.<br>• Change from baseline in WI-NRS at Week 12 and Week 24.<br>• Percent change from baseline in WI-NRS at Week 12 and Week 24.<br>• Percent change from baseline in WI-NRS over time until Week 24.<br>• Proportion of participants with WI-NRS reduction ≥4 over time until Week 24.<br>• Onset of action in change from baseline in WI-NRS (first $p < 0.05$ difference from placebo in the daily WI-NRS that remains significant at subsequent measurements) until Week 12.
• To demonstrate efficacy of dupilumab on skin lesions of PN.	• Proportion of participants with Investigator's Global Assessment 0/1 score for PN-Stage (IGA PN-S) at Week 4, Week 8, Week 12, and Week 24.<br>• Change from baseline in IGA PN-S score at Week 4, Week 8, Week 12, and Week 24.<br>• Proportion of participants with Investigator's Global Assessment 0/1 score for PN-Assess (IGA PN-A) at Week 4, Week 8, Week 12, and Week 24.
• To demonstrate the improvement in health-related quality of life (HRQoL).	• Change from baseline in HRQoL, as measured by Dermatology Life Quality Index (DLQI) to Week 12 and Week 24.
• To evaluate safety outcome measures.	• Percentage of participants experiencing treatment-emergent adverse events (TEAEs) or serious adverse events (SAEs) from baseline through Week 24.
• To evaluate immunogenicity of dupilumab.	• Incidence of treatment-emergent antidrug antibodies (ADA) against dupilumab over time.

Objectives	Endpoints
**Tertiary/exploratory**	
• To demonstrate a reduction in the use of rescue medication and systemic immunosuppressant	• Use of high potency TCS rescue medication through Week 24<br>• Use of systemic immunosuppressant through Week 24, constituting treatment failure
• To evaluate exploratory outcome measures	• Change from baseline in Hospital Anxiety and Depression Scale (HADS) total score to Week 24.<br>• Change from baseline in EQ5D-5L to Week 24.<br>• Change from baseline in Pain numeric rating scale (NRS) to Week 4, Week 8, Week 12, and Week 24.<br>• Change from baseline in Sleep NRS to Week 4, Week 8, Week 12, and Week 24.<br>• Missed school/work days through Week 24.<br>• Incidence of skin-infection TEAEs (excluding herpetic infections) through Week 24.
• To evaluate efficacy of dupilumab on skin lesions using a modified PAS 5-item questionnaire	• Percent change from baseline in number of healed lesions (as part of PAS) at Week 4, Week 8, Week 12, and Week 24.<br>• Change from baseline in exact number of lesions in representative area (as determined from PAS) at Week 4, Week 8, Week 12, and Week 24.<br>• Change from baseline in PAS total score at Week 4, Week 8, Week 12, and Week 24
• To evaluate efficacy of dupilumab on other PN endpoints	• Change from baseline in Patient Global Impression of Severity (PGIS) of PN to Week 4, Week 8, Week 12, and Week 24 and Patient Global Impression of Change (PGIC) of PN at Week 4, Week 8, Week 12, and Week 24.
**PK**	
• To evaluate pharmacokinetic (PK) and pharmacodynamic (PD) outcome measures	• Serum functional dupilumab concentrations and PK profile.<br>• Pharmacodynamic response for selected biomarkers (total IgE).

3.1 APPROPRIATENESS OF MEASUREMENTS

The study endpoints will include both assessments of itch and assessments of the PN lesions. As discussed in Section 2.2, the severe itch observed in this disease, the fact that lesions are typically observed in areas that are within reach for scratching, and the importance of itch-scratch cycles in the pathophysiology of the disease support itch as a reasonable primary endpoint. This choice is in line with the study design publicly available for other drug classes such as NK1-antagonists (20). Amongst standard measurement instruments for evaluation of chronic pruritus, NRS assessing worst itch in the past 24 hours features high reliability and concurrent validity and is a popular choice for all patients due to its simple format (21, 22, 23, 24). The proposed WI-NRS is currently being used in Phase 3 trial assessing serlopitant efficacy in PN (20). A similar pruritus NRS (peak pruritus) has been validated for AD. Additional validation work covering PN as additional

indication will be conducted in the context of the current study. The primary assessment will be a responder analysis based on an at least 4 points improvement of the WI-NRS at Week 12, similar to the assessment widely used in AD studies, and similar to the value proposed for assessment of clinical improvement in the WI-NRS as measures of response in the Phase 2 trial assessing serlopitant efficacy and safety in PN, and currently used in the Phase 3 trial (20). The itch response occurs typically faster than the lesion response but since improvement of both may occur prior to Week 12, assessments will be performed at Week 4 and Week 8. The study of the primary assessment of itch is planned for Week 12 and the lesion assessments are planned for Week 12 and Week 24.

As a secondary endpoint, PN lesions will be assessed by an IGA PN, which is widely used in several dermatological indications (as Physician Global Assessment [PGA] in psoriasis). A modified PAS will be used as an exploratory measure. Both instruments are PN-specific and have been developed by experts in the field. These are the only two disease-specific instruments currently available for the lesion assessment. As an exploratory measure, the PAS will be evaluated. The validation of both instruments for use in PN will be conducted in the context of the Phase 3 trials.

Secondary endpoints will also include HRQoL assessments using the skin-disease specific DLQI in recognition of the significant impacts on quality of life experienced by patients with PN. Even though not validated in PN specifically, the DLQI is well established and widely used to assess HRQoL in patients with skin conditions in clinical trials (25).

4 STUDY DESIGN

4.1 OVERALL DESIGN

This study is a multi-center, 24-week treatment, parallel, double-blind, randomized, placebo-controlled study to evaluate the use of dupilumab in patients with PN inadequately controlled on topical prescription therapies or when those therapies are not advisable.

The study will assess the effect of dupilumab on itch improvement as well as its effect on PN lesions, on patients' health-related quality of life, anxiety and depression, sleep quality and skin pain, and overall health status.

Participants who satisfy the inclusion and exclusion criteria will be randomized (1:1) to one of the following IMP treatment groups:

- Dupilumab 300 mg
- Matched placebo

Duration of study period (per participant)

- Screening period (2-4 weeks)
- Randomized IMP intervention period (24 weeks)
- Follow-up period (12 weeks)

4.2 SCIENTIFIC RATIONALE FOR STUDY DESIGN

This study is a multi-center, 24-week treatment, double-blind, randomized, placebo-controlled study to evaluate the use of dupilumab in patients with PN inadequately controlled on topical prescription therapies or when those therapies are not advisable. Based on mechanistic data (see Section 2), Type 2-inflammation may play a principal role in the pathogenesis of PN in all patients.

The double-blind design intends to mitigate potential bias and placebo control is necessary to assess the net effect of active dupilumab treatment.

The patient population to be studied in this trial is intended to include patients with underlying systemic medical conditions known to be associated with PN. Given that better control of severe manifestations of underlying systemic illnesses can confound the primary itch endpoint, we will enroll 40% to 60% of non-atopic PN patients that have medically well-controlled systemic conditions. Since the literature suggests that approximately 40-60% of PN patients have a history of atopy (defined as having a medical history of AD, allergic rhinitis/rhinoconjunctivits, asthma, or food allergy), our study design will require that the remaining PN patients have a history or a current diagnosis of atopy. Because active AD can confound the primary endpoint, we will

exclude moderate-to-severe AD patients and will institute a cap of up to 10% of the atopic patient subgroup having active mild AD. It is the goal of this design to mirror the overall PN patient population consistent with real-world estimates of overall prevalence while trying to minimize potential confounders on the primary endpoint.

The primary endpoint, WI-NRS, is a validated measurement of the highest PN burden, ie, extreme itching.

Dupilumab treatment has shown clinical efficacy prior to Week 24 in all Phase 3 trials examined across all indications. In addition, available biomarker data of disease response, including TARC and eotaxin-3, plateau after Week 16 but before Week 24 in prior AD trials, including the 52-week AD study (R668-AD-1224; CHRONOS). The Sponsor considers that a 24-week treatment period is an appropriate duration to observe dupilumab’s effect in PN to ensure an adequate evaluation of PN lesions. The duration of the 12-week follow-up period is based on the time expected for drug concentration to reach zero (below the lower limit of quantification) in most patients after the last dose of dupilumab. This follow-up period will allow assessment of the need for chronic treatment with dupilumab for this disease by an assessment of return of PN symptoms post-treatment.

The study will not include adolescents because PN is rare in adolescent patients.

4.3 JUSTIFICATION FOR DOSE

The selected dosing regimen is dupilumab 300 mg Q2W with a loading dose of 600 mg.

This dose regimen is expected to achieve serum concentrations that saturate the target-mediated clearance pathway rapidly with the loading dose and maintain saturation thereafter. The PK of dupilumab is consistent across populations of patients with AD, asthma, CRSwNP, and EoE, as well as healthy volunteers. Furthermore, the sources of variability of dupilumab PK identified in each population and the magnitude of the covariate effects indicate that body weight is the most influential factor, whereas other covariates identified as being statistically significant have no meaningful impact on dupilumab PK. The immunogenicity of dupilumab is also comparable in these populations (see IB).

Dosing regimens of 300 mg Q2W after a loading dose of 600 mg were used in the PN case studies, which resulted in marked improvement in itch within three months and subsequently a notable improvement of the skin lesions (see Section 2).

The 300 mg Q2W dose, with loading dose, is the approved dose for AD and is an approved dose for asthma in the US and the EU.

Therefore, based on the totality of the data, dupilumab 300 mg Q2W (with loading dose) regimen is adequate for patients with PN and a further dose ranging study is not necessary to select a Phase 3 dose regimen.

4.4 END OF STUDY DEFINITION

A participant is considered to have completed the study if he/she has completed all phases of the study including the last visit.

The end of the study is defined as the date of the last visit of the last participant in the study.

5 STUDY POPULATION

Prospective approval of protocol deviations to recruitment and enrollment criteria, also known as protocol waivers or exemptions, is not permitted.

5.1 INCLUSION CRITERIA

Participants are eligible to be included in the study only if all of the following criteria apply:

Age

I 01. Participants must be 18 to 80 years of age, at the time of signing the informed consent.

Type of participant and disease characteristics

Patients with a clinical diagnosis of PN, as defined by all of the following:

I 02. Diagnosed by a dermatologist for at least 3 months before the Screening visit.

I 03. On the WI-NRS ranging from 0 to 10, patients must have an average worst itch score of ≥7 in the 7 days prior to Day 1.

NOTE: Baseline pruritus NRS average score for maximum itch intensity will be determined based on the average of daily NRS scores for maximum intensity (the daily score ranges from 0 to 10) during the 7 days immediately preceding randomization. A minimum of 4 daily scores out of the 7 days is required to calculate the baseline average score. For patients who do not have at least 4 daily scores reported during the 7 days immediately preceding the planned randomization date, randomization should be postponed until this requirement is met, but without exceeding the 28-day maximum duration of the screening period.

I 04. Patients must have a minimum of 20 PN lesions in total on both legs, and/or both arms and/or trunk, at Screening visit and on Day 1.

NOTE: Patients need to have bilaterally symmetrical lesions on the extremities. The presence of lesions on at least 2 body surface areas is required.

I 05. History of failing a 2-week course of medium-to-high potency TCS or when TCS are not medically advisable.

NOTE: Failure is defined as patients who are unable to achieve and/or maintain remission and low disease activity (similar to IGA PN-S score of ≤2 [≤19 nodules]) despite treatment with a daily regimen of medium to higher potency TCS (±TCI as appropriate), applied for at least 14 days, or for the maximum duration recommended by the product prescribing information, whichever is shorter.

I 06. Have applied a stable dose of topical emollient (moisturizer) once or twice daily for at least the 7 consecutive days immediately before Day 1 (NOTE: See exclusion criterion E 20 for limitations regarding emollients; see background treatment with topical emollient in Section 6.1.1).

I 07. Participants must be willing and able to complete a daily symptom eDiary for the duration of the study.

Sex

I 08. Male or Female

Contraceptive use by women should be consistent with local regulations regarding the methods of contraception for those participating in clinical studies.

a) Female participants

- A female participant is eligible to participate if she is not pregnant or breastfeeding, and at least one of the following conditions applies:
  - Is not a WOCBP.

  OR

  - Is a WOCBP and agrees to use an acceptable contraceptive method as described in Appendix 4 (Section 10.4) during the study (at a minimum until 12 weeks after the last dose of study intervention).
  - A WOCBP must have a negative highly sensitive (Section 10.2) pregnancy test (urine or serum as required by local regulations) on Day 1 before the first dose of study intervention.
  - If a urine test cannot be confirmed as negative (eg, an ambiguous result), a serum pregnancy test is required. In such cases, the participant must be excluded from participation if the serum pregnancy result is positive.
- Additional details can be found in Appendix 4 (Section 10.4).
- The investigator is responsible for review of medical history, menstrual history, and recent sexual activity to decrease the risk for inclusion of a woman with an early undetected pregnancy.

Informed Consent

I 09. Capable of giving signed informed consent as described in Appendix 1 (Section 10.1) of the protocol which includes compliance with the requirements and restrictions listed in the informed consent form (ICF) and in this protocol. In countries where legal age of majority is above 18 years, a specific ICF must also be signed by the participant's legally authorized representative.

5.2 EXCLUSION CRITERIA

Participants are excluded from the study if any of the following criteria apply:

Medical conditions

E 01. Presence of skin morbidities other than PN and mild AD that may interfere with the assessment of the study outcomes. Conditions such as, but not limited to, the following: scabies, insect bite, lichen simplex chronicus, psoriasis, acne, folliculitis, habitual picking, lymphomatoid papulosis, chronic actinic dermatitis, dermatitis herpetiformis, sporotrichosis, bullous disease.

NOTE: patients with mild active AD will represent up to 10% of the atopic PN study population.

E 02. PN secondary to medications (eg, opioids, angiotensin converting enzyme [ACE] inhibitors).

E 03. PN secondary to medical conditions such as neuropathy or psychiatric disease (eg, notalgia paresthetica, brachioradial pruritus, neurotic excoriations, obsessive compulsive disorder, delusions of parasitosis, etc).

E 04. Patients with a documented AD severity of moderate to severe within 6 months before or at the screening visit (eg, IGA AD of 3 or 4, eczema area and severity index [EASI] ≥16, scoring atopic dermatitis [SCORAD] ≥25).

E 05. Severe concomitant illness(es) under poor control that, in the investigator's judgment, would adversely affect the patient's participation in the study. Examples include, but are not limited to patients with life expectancy shorter than 1 year, patients with uncontrolled diabetes (hemoglobin A1c ≥9%), patients with cardiovascular conditions (eg, Class III or IV heart failure according to the New York Heart Association classification), hepato-biliary conditions (eg, Child-Pugh Class B or C), neurological conditions (eg, demyelinating diseases), active major autoimmune diseases (eg, lupus, inflammatory bowel disease, rheumatoid arthritis, etc), other severe endocrinological, gastrointestinal, metabolic, pulmonary, or lymphatic diseases. The specific justification for patients excluded under this criterion will be noted in study documents (chart notes, eCRF, etc).

E 06. Severe renal conditions (eg, patients with uremia and/or on dialysis).

E 07. Participants with uncontrolled thyroid disease.

E 08. Active TB or non-tuberculous mycobacterial infection, or a history of incompletely treated TB will be excluded from the study unless it is well documented by a specialist that the participant has been adequately treated and can now start treatment with dupilumab in the medical judgment of the investigator and/or infectious disease specialist. Tuberculosis testing will be performed on a country-by-country basis, according to local guidelines if required by regulatory authorities or ethics boards.

E 09. Diagnosed active endoparasitic infections; suspected or high risk of endoparasitic infection, unless clinical and (if necessary) laboratory assessment have ruled out active infection before randomization.

E 10. Active chronic or acute infection (except HIV infection) requiring treatment with systemic antibiotics, antivirals, antiprotozoals, or antifungals within 2 weeks before the screening visit.

E 11. Known or suspected immunodeficiency, including history of invasive opportunistic infections (eg, TB, histoplasmosis, listeriosis, coccidioidomycosis, pneumocystosis, aspergillosis) despite infection resolution, or otherwise recurrent infections of abnormal frequency or prolonged duration suggesting an immune-compromised status, as judged by the investigator.

E 12. Active malignancy or history of malignancy within 5 years before the baseline visit, except completely treated in situ carcinoma of the cervix, completely treated and resolved non-metastatic squamous or basal cell carcinoma of the skin.

E 13. History of systemic hypersensitivity or anaphylaxis to any biologic therapy, including any excipients.

E 14. Any other medical or psychological condition including relevant laboratory abnormalities at screening that, in the opinion of the investigator, suggest a new and/or insufficiently understood disease, may present an unreasonable risk to the study patient as a result of his/her participation in this clinical trial, may make patient's participation unreliable, or may interfere with study assessments. The specific justification for patients excluded under this criterion will be noted in study documents (chart notes, eCRF, etc).

E 15. History of substance and/or alcohol abuse.

E 16. Planned major surgical procedure during the patient's participation in this study.

Prior/concomitant therapy

E 17. Exposure to another systemic or topical investigative drug (monoclonal antibodies as well as small molecules) within a certain time period prior to Visit 1 (screening), as follows: an interval of less than 6 months or <5 PK half-lives for investigative monoclonal antibodies, whichever is longer, and an interval of less than 30 days or <5 PK half-lives, whichever is longer, for investigative small molecules.

E 18. Having used any of the following treatments within 4 weeks before the screening visit

- Systemic immunosuppressive/immunomodulating drugs (eg, systemic corticosteroids, cyclosporine, mycophenolate-mofetil, interferon gamma, Janus kinase inhibitors, azathioprine, methotrexate, hydroxychloroquine, dapsone, sulfasalazine, colchine, etc).
- Intralesional corticosteroid injections and cryotherapy.
- Phototherapy, including tanning beds.

- Naltrexone or other opioid antagonist.
- Paroxetine, fluvoxamine, or other selective serotonin reuptake inhibitors (SSRIs).
- Serotonin and norepinephrine reuptake inhibitors (SNRIs)
- Amitriptyline or other tricyclic antidepressants.
- Gabapentin, pregabalin, and thalidomide.

E 19. Previous treatment with biologic medicines within the following timeframe:

- Any cell-depleting agents including but not limited to rituximab: within 6 months before the screening visit.
- Omalizumab: within 5 months before screening visit.
- Other immunomodulatory biologics: within 5 half-lives (if known) or 16 weeks before the screening visit, whichever is longer.

E 20. Initiation of treatment with prescription moisturizers or moisturizers containing additives such as ceramide, hyaluronic acid, urea, or filaggrin degradation products during the screening period (patients may continue using stable doses of such moisturizers if initiated before the Screening visit).

E 21. Initiation of treatment with TCS/TCI (any potency) during the screening period or treatment with high potency or superpotent TCS/TCI during the screening period.

E 22. For participants who were on a stable regimen of TCS/TCI at the screening visit:

- Application of TCS/TCI on fewer than 6 days during the 7 days immediately preceding randomization.
- Application of TCS/TCI of incorrect potency within 7 days before Day 1 according to the requirements of Section 6.1.1, ie, low potency if on low potency at screening visit and medium potency if on medium or higher potency at screening visit.

E 23. Treatment with a live (attenuated) vaccine within 4 weeks before the screening visit.

NOTE: For patients who have vaccination with live, attenuated vaccines planned during the course of the study (based on national vaccination schedule/local guidelines), it will be determined, after consultation with a physician, whether the administration of vaccine can be postponed until after the end of study, or preponed to before the start of the study, without compromising the health of the patient:

- Patient for whom administration of live (attenuated) vaccine can be safely postponed would be eligible to enroll into the study.
- Patients who have their vaccination preponed can enroll in the study only after a gap of 4 weeks following administration of the vaccine.

E 24. Planned or anticipated use of any prohibited medications and procedures during screening and study treatment period.

Prior/concurrent clinical study experience

E 25. Participation in prior dupilumab clinical study, prior use of biologics for PN.

Diagnostic assessments

E 26. HIV with CD4+ counts ≤300 cells/µL and/or detectable HIV viral load at the screening visit.

E 27. Participants with any of the following result at screening:

- Positive (or indeterminate) HBs Ag or,
- Positive HBV DNA or,
- Positive HCV RNA.

Other exclusions

E 28. Individuals accommodated in an institution because of regulatory or legal order; prisoners or subjects who are legally institutionalized.

E 29. Any country-related specific regulation that would prevent the subject from entering the study - see Section 10.10 (country-specific requirements).

E 30. Participant not suitable for participation, whatever the reason, as judged by the Investigator, including medical or clinical conditions, or participants potentially at risk of noncompliance to study procedures.

E 31. Participants are dependent on the Sponsor or Investigator (in conjunction with Section 1.61 of the International Council for Harmonisation (ICH) good clinical practice (GCP) Ordinance E6).

E 32. Participants are employees of the clinical study site or other individuals directly involved in the conduct of the study, or immediate family members of such individuals.

E 33. Any specific situation during study implementation/course that may raise ethics considerations.

E 34. Sensitivity to any of the study interventions, or components thereof, or drug or other allergy that, in the opinion of the Investigator, contraindicates participation in the study.

5.3 LIFESTYLE CONSIDERATIONS

Not applicable.

5.4 SCREEN FAILURES

Screen failures are defined as participants who consent to participate in the clinical study but are not subsequently randomly assigned to study intervention/entered in the study. A minimal set of screen failure information is required to ensure transparent reporting of screen failure participants to meet the Consolidated Standards of Reporting Trials (CONSORT) publishing requirements and to respond to queries from regulatory authorities. Minimal information includes demography, screen failure reasons, eligibility criteria, and any SAE.

In the case of technical malfunction of equipment, the patient may be rescreened. Patients who fail the exclusion criteria may be rescreened once during the open screening period of the study. Rescreened participants will be assigned a new participant number versus the one received for the initial screening.

There is no requirement for a waiting period between the screen-failure date and the rescreening date. The interactive response technology (IRT) report will flag rescreened patients. Patients that are rescreened must sign a new consent form and all Visit 1 procedures must be repeated.

If certain dynamic laboratory tests do not meet the eligibility criteria at screening, these laboratory assessments may be repeated, at the discretion of the Investigator, if it is judged to be likely to return to acceptable range for study inclusion within the screening visit window prior to Day 1. There is no need to screen fail such participants if the test finally meets the eligibility criteria.

6 STUDY INTERVENTION

Study intervention is defined as any investigational intervention(s), marketed product(s), placebo, or medical device(s) intended to be administered to a study participant according to the study protocol.

6.1 STUDY INTERVENTION(S) ADMINISTERED

Table 2 - Overview of study interventions administered

**ARM name**	Dupilumab	Placebo
**Intervention name**	Dupilumab 300 mg	Placebo matching dupilumab 300 mg
**Type**	Biological/Vaccine	Other
**Dose formulation**	A 150 mg/mL dupilumab solution in a pre-filled syringe to deliver 300 mg in 2 mL	Identical formulation to the active 300 mg formulation without dupilumab, in a pre-filled syringe to deliver placebo in 2 mL
**Unit dose strength(s)**	300 mg	0 mg (placebo)
**Dosage level(s)**	300 mg every 14 ±3 days after an initial loading dose of 600 mg	0 mg every 14 ±3 days with a loading dose of 0 mg
**Route of administration**	Subcutaneous[a]	Subcutaneous[a]
**IMP and NIMP**	IMP	IMP
**Packaging and labeling**	One glass pre-filled syringe packed in a patient kit box. Both the glass pre-filled syringe and the box will be labeled as required per country requirement.	One glass pre-filled syringe packed in a patient kit box. Both glass pre-filled the syringe and the box will be labeled as required per country requirement

a Subcutaneous injection sites should alternate between the upper thighs, 4 quadrants of the abdomen or the upper arms, so that the same site is not injected twice during consecutive administrations. Injection in the upper arms can only be done by a trained person (parent/legally authorized representative/caregiver trained by Investigator or Delegate) or health care professional but not the participant themselves.

IMP: investigational medicinal product; NIMP: noninvestigational medicinal product.

The Investigator or delegate will train the patient (or caregiver) how to prepare and inject IMP at Visit 2. He/she will inject the first of the two injections. The participant (or caregiver) will perform the second injection under the supervision of the Investigator or delegate. Patient should also be trained by the site staff to recognize potential signs and symptoms of hypersensitivity reaction in order to self-monitor at home for at least 30 minutes (or longer per country specific or local site-specific requirements) following injection. In case of hypersensitivity symptoms the patient should contact healthcare provider/emergency. The training must be documented in the participant's study file.

When the participant has a study visit, the IMP will be administered following clinical procedures and blood collection. Patients should be monitored for at least 30 minutes. The monitoring period may be extended as per country specific or local site-specific requirements.

Between the protocol-scheduled on-site visits, participants are allowed to self-inject IMP at home. Participants who prefer to have a healthcare professional administer the IMP may choose to have injections administered at home by a nurse or at the study site.

Between the protocol-scheduled on-site visits, interim visits may be required for IMP dispensing. As an alternative to these visits, dupilumab 300 mg or matching placebo may be supplied from the site to the participant via a Sponsor-approved courier company where allowed by local regulations and approved by the participant.

6.1.1 Noninvestigational medicinal products

Participants will be required to apply moisturizers (emollients) once or twice daily for at least the 7 consecutive days immediately before Day 1 and continue until Week 36. However, to allow adequate assessment of skin dryness, moisturizers should not be applied on the area(s) of skin designated for such assessments for at least 8 hours before each clinic visit. All types of moisturizers are permitted, but patients may not initiate new treatment with prescription moisturizers or over-the-counter moisturizers containing additives during the screening period or during the intervention period. Patients may continue using stable doses of such moisturizers if initiated before the screening visit.

If participants are on a stable regimen of low to medium potency TCS or TCI at the screening visit, they can continue their topical steroid application once daily without tapering from Screening to Week 24. If specific lesions resolve, the participant can stop applying steroids to those sites but are permitted to continue applying to persistent lesions. If participants are on stable regimens of high potency or superpotent steroids, participants should decrease potency to medium potency TCS and continue to apply daily from screening to Week 24. Occlusion is not allowed from Screening to Week 24.

It is recommended that patients use triamcinolone acetonide 0.1% cream or fluocinolone acetonide 0.025% ointment for medium potency, and hydrocortisone 1% cream for low potency. If rescue with TCS is needed, it is recommended that patients use either betamethasone dipropionate 0.05% optimized ointment for high potency TCS or clobetasol propionate 0.05% cream for super high potency TCS. If patients have tolerance issues with any of these steroids or if they are not commercially available in some countries, they may substitute with products of the same potency from the list provided by the Sponsor.

On areas treated with TCS, moisturizers must be applied once daily only at the time when TCS is not applied (ie, do not use moisturizers and TCS on the same areas at the same time during the day). For example, if TCS are applied in the evening, moisturizers will not be used in the evening on areas treated with TCS, but will be applied to those areas in the morning. On areas not treated with TCS, moisturizers will be applied twice daily - morning and evening.

Rescue therapy is described in Section 6.5.1.

6.2 PREPARATION/HANDLING/STORAGE/ACCOUNTABILITY

6.2.1 Storage and handling

1. The Investigator or designee must confirm appropriate temperature conditions have been maintained during transit for all study intervention received and any discrepancies are reported and resolved before use of the study intervention.
2. Only participants enrolled in the study may receive study intervention and only authorized site staff may supply or administer study intervention. All study intervention must be stored in a secure, environmentally controlled, and monitored (manual or automated) area in accordance with the labeled storage conditions with access limited to the Investigator and authorized site staff.
3. The Investigator, institution, or the head of the medical institution (where applicable) is responsible for study intervention accountability, reconciliation, and record maintenance (ie, receipt, reconciliation, and final disposition records).

6.2.2 Responsibilities

Any quality issue noticed with the receipt or use of an IMP (deficiency in condition, appearance, pertaining documentation, labeling, expiration date, etc) must be promptly notified to the Sponsor. Some deficiencies may be recorded through a complaint procedure (see Section 8.3.6).

A potential defect in the quality of IMP may be subject to initiation of a recall procedure by the Sponsor. In this case, the Investigator will be responsible for promptly addressing any request made by the Sponsor, in order to recall the IMP and eliminate potential hazards.

Under no circumstances will the Investigator supply IMP to a third party (except for direct-to-patient [DTP] shipment, for which a courier company has been approved by the Sponsor), allow the IMP to be used other than as directed by this clinical trial protocol, or dispose of IMP in any other manner.

6.3 MEASURES TO MINIMIZE BIAS: RANDOMIZATION AND BLINDING

All participants will be centrally assigned to randomized study intervention using an IRT. Before the study is initiated, the telephone number and call-in directions for the IVRS and/or the log in information & directions for the IWRS will be provided to each site.

At screening (Visit 1), the Investigator or designee will contact the IRT system to receive the participant number. If a patient who had previously failed screening is approached for re-screening, a new ICF must be signed. In such case, a new patient number will be assigned by IRT.

Methods of assigning patients to treatment group

The randomized intervention kit number list is generated centrally by Sanofi and IMPs are packaged in accordance with this list. The randomization and intervention allocation are performed centrally by an IRT. The IRT generates the participant randomization list and allocates the intervention number and the corresponding intervention kits to the participants according to it.

Participants will be randomized in 1:1 ratio to treatment arms described in Table 2.

Randomization will be stratified by the following factors:

- documented history of atopy (atopic or non-atopic)
  - Atopic: Patients with a physician-documented history of atopic comorbidities defined as AD, allergic rhinitis/rhinoconjunctivits, asthma, or food allergy, or a current diagnosis of at least one of these atopic comorbidities, per investigator judgement. NOTE: enrollment of patients with a current diagnosis of active mild AD will be capped at up to 10% of the participants in this subgroup
  - Non-atopic: Patients without a physician-documented history of atopic comorbidities defined as AD, allergic rhinitis/rhinoconjunctivitis, asthma or food allergy, and without a current diagnosis of at least one of such atopic comorbidities, per investigator judgement
- stable use of TCS/TCI (yes or no)
- country/territory code

A randomized participant is defined as a participant who has been allocated to a randomized intervention regardless whether the intervention kit was used or not (ie, participant registered by the IRT).

A participant cannot be randomized more than once in the study.

Study intervention will be dispensed at the study visits summarized in SoA (see Section 1.3). Returned study intervention should not be re-dispensed to the participants.

Methods of blinding

- Dupilumab 300 mg and placebo matching dupilumab 300 mg will be provided in identically matched 2 mL pre-filled syringes that are visually indistinguishable. Syringes and box will be labeled with a treatment kit number.
- The unblinding plan is described in Section 9.5.2.

Randomization code breaking during the study

The IRT will be programmed with blind-breaking instructions. In case of an emergency, the Investigator has the sole responsibility for determining if unblinding of a participant's treatment assignment is warranted (eg, in case of available antidote). Participant safety must always be the first consideration in making such a determination. If the Investigator decides that unblinding is warranted, the Investigator should make every effort to contact the Sponsor prior to unblinding a participant's treatment assignment unless this could delay emergency treatment of the participant. If a participant's treatment assignment is unblinded, the Sponsor must be notified within 24 hours after breaking the blind. The date and reason that the blind was broken must be recorded in the source documentation and case report form (CRF), as applicable.

6.4 STUDY INTERVENTION COMPLIANCE

Investigator or his/her delegate must ensure that IMP will be administered to each participant according to the labeling instructions.

IMP accountability:

- Intervention units are returned by the participant at each visit. In case of DTP process, the intervention units can be returned by the carrier (if defined in the contract).
- The Investigator counts the number of remaining kit/pre-filled syringe, and fills in the IMP accountability and inventory forms.
- The Investigator or his/her delegate records the dosing information on the appropriate page(s) of the eCRF.
- The monitor in charge of the study then checks the eCRF data by comparing them with the IMP which he/she has retrieved and source documents.

Participant compliance with study intervention will be assessed at each visit. Compliance will be assessed by counting returned kit/pre-filled syringe. Deviation(s) from the prescribed dosage regimen should be recorded in the eCRF.

6.5 CONCOMITANT THERAPY

Any medication or vaccine (including over-the-counter or prescription medicines, vitamins, and/or herbal supplements) that the participant is receiving at the time of enrollment or receives during the study must be recorded along with:

- Reason for use.
- Dates of administration including start and end dates.
- Dosage information including dose and frequency.

The concomitant use of the following therapies is prohibited during the entire study. Study treatment will need to be discontinued in participants receiving these treatments:

- Systemic immunosuppressive/immunomodulating drugs (eg, systemic corticosteroids, cyclosporine, mycophenolate-mofetil, interferon gamma, Janus kinase inhibitors, azathioprine, methotrexate, hydroxychloroquine, dapsone, sulfasalazine, colchine, etc).
- Other biologics.
- Phototherapy, including tanning beds.
- Naltrexone or other opioid antagonist.
- Gabapentin, pregabalin, and thalidomide.

The concomitant use of the following therapies is also prohibited during the entire study, but study treatment will not need to be discontinued in participants receiving the treatments listed below.

- Intralesional corticosteroid injections and cryotherapy.
- Paroxetine, fluvoxamine, or other SSRIs.
- SNRIs.
- Amitriptyline or other tricyclic antidepressants.

6.5.1 Rescue medicine

The following rescue medications may be used:

- Dermatological preparations of high potency or superpotent TCS and TCI.

Although the use of rescue medications is allowed at any time during the study, the use of rescue medications should be delayed, if possible, for at least 14 days following the initiation of the investigational treatment. The date and time of rescue medication administration as well as the name and dosage regimen of the rescue medication must be recorded in the eCRF.

6.6 DOSE MODIFICATION

No change in IMP dose is allowed.

6.7 INTERVENTION AFTER THE END OF THE STUDY

The Sponsor will not be responsible for intervention after the EOS Visit. Intervention after the EOS Visit will be at the discretion of investigator or treating physician.

7 DISCONTINUATION OF STUDY INTERVENTION AND PARTICIPANT DISCONTINUATION/WITHDRAWAL

7.1 DISCONTINUATION OF STUDY INTERVENTION

7.1.1 Definitive discontinuation

In rare instances, it may be necessary for a participant to permanently discontinue study intervention. If study intervention is permanently discontinued, the participant will remain in the study to be evaluated for safety. See the SoA (Section 1.3) for data to be collected at the time of discontinuation of study intervention.

The participants may withdraw from treatment with the IMP if he or she decides to do so, at any time and irrespective of the reason, or this may be the investigator's decision. All efforts should be made to document the reason(s) for treatment discontinuation and this should be documented in the eCRF.

Participants must be permanently withdrawn from the study treatment for the following reasons:

- At their own request or at the request of their legally authorized representative (legally authorized representative means an individual or judicial or other body authorized under applicable law to consent on behalf of a prospective participant to the patient's participation in the procedure(s) involved in the research).
- If, in the investigator's opinion, continuation in the study would be detrimental to the participant's well-being.
- At the specific request of the Sponsor.
- If they are treated with the specific prohibited medications mentioned in Section 6.5.
- If they miss 2 consecutive IMP doses.
- In the event of a protocol deviation, at the discretion of the investigator or the Sponsor.
- Any code broken at the requested of the investigator.
- Pregnancy.
- Anaphylactic reactions or systemic allergic reactions that are related to IMP and require treatment (see Section 10.8).
- Diagnosis of a malignancy during study, excluding carcinoma in situ of the cervix, or squamous or basal cell carcinoma of the skin.
- Any opportunistic infection or other infections whose nature or course may suggest an immunocompromised status (see Section 10.9).
- Serum ALT >3 ULN and Total Bilirubin >2 ULN (see Section 10.6).
- Serum ALT >5 ULN if baseline ALT ≤2 ULN or ALT >8 ULN if baseline ALT >2 ULN (see Section 10.6).

If a clinically significant finding is identified (including, but not limited to changes from baseline in QT interval corrected using Fridericia's formula [QTcF] after enrollment), the Investigator or qualified designee will determine if the participant can continue in the study and if any change in participant management is needed. This review of the ECG printed at the time of collection must be documented. Any new clinically relevant finding should be reported as an AE.

Any abnormal laboratory value or ECG parameter will be immediately rechecked for confirmation within a reasonable timeframe as assessed by the Investigator before making a decision of definitive discontinuation of the IMP for the concerned participant.

Handling of participants after definitive intervention discontinuation

Participants will be followed-up according to the study procedures specified in this protocol up to the scheduled date of study completion, or up to recovery or stabilization of any AE to be followed-up as specified in this protocol, whichever comes last.

Participants who discontinue the study intervention prematurely (prior to completing the 24-week treatment period) will perform, as soon as possible, the early termination visit with all assessments normally planned for the EOT Visit (Visit 6), to assure a complete clinical assessment in close temporal proximity to the premature termination of study treatment is available.

In addition, and to allow assessment of participant outcomes over the stipulated study period, patients will be asked and encouraged to complete all remaining study treatment visits, and participate in all assessments according to the visit schedule with a ±3 day window. Under exceptional circumstances when a participant cannot come to the site for a scheduled visit, a phone contact can be made. During the phone contact, at least information about AEs, concomitant medication and status of PN should be collected.

All cases of definitive intervention discontinuation must be recorded by the Investigator in the appropriate pages of the eCRF when considered as confirmed.

7.1.2 Temporary discontinuation

Temporary intervention discontinuation may be considered by the Investigator because of suspected AEs.

In addition, the following conditions will be causes for temporary discontinuation of study intervention:

- Infections or infestations that do not respond to medical treatment
- Any laboratory abnormality that meets temporary treatment discontinuation criteria as per Section 10.6

Temporary intervention discontinuation decided by the Investigator corresponds to more than 1 dose not administered to the participant. Following a temporary interruption or missed dose, the treatment should be reinitiated at the next scheduled administration, maintaining the planned dose. If 2 consecutive IMP doses have been missed, the study treatment will be definitively discontinued (see Section 7.1.1).

7.1.2.1 Rechallenge

Reinitiation of intervention with the IMP will be done under close and appropriate clinical/and or laboratory monitoring once the Investigator will have considered according to his/her best medical judgment that the responsibility of the IMP(s) in the occurrence of the concerned event was unlikely and if the selection criteria for the study are still met (refer to Section 5).

7.2 PARTICIPANT DISCONTINUATION/WITHDRAWAL FROM THE STUDY

- A participant may withdraw from the study at any time at his/her own request, or may be withdrawn at any time at the discretion of the Investigator for safety, behavioral, compliance, or administrative reasons. This is expected to be uncommon.
- At the time of discontinuing from the study, if possible, an early discontinuation visit should be conducted, as shown in the SoA (Section 1.3). See SoA for data to be collected at the time of study discontinuation and follow-up and for any further evaluations that need to be completed.
- The participant will be permanently discontinued both from the study intervention and from the study at that time.
- If the participant withdraws consent for disclosure of future information, the Sponsor may retain and continue to use any data collected before such a withdrawal of consent.
- If a participant withdraws from the study, he/she may request destruction of any samples taken and not tested, and the Investigator must document this in the site study records.

If participants no longer wish to take the IMP, they will be encouraged to remain in the study.

The Investigators should discuss with them key visits to attend. The value of all their study data collected during their continued involvement will be emphasized as important to the public health value of the study.

Participants who withdraw from the study intervention should be explicitly asked about the contribution of possible AEs to their decision, and any AE information elicited must be documented.

All study withdrawals should be recorded by the Investigator in the appropriate screens of the eCRF and in the participant's medical records. In the medical record, at least the date of the withdrawal and the reason should be documented.

In addition, a participant may withdraw his/her consent to stop participating in the study. Withdrawal of consent for intervention should be distinguished from withdrawal of consent for follow-up visits and from withdrawal of consent for non-participant contact follow-up, eg, medical record checks. The site should document any case of withdrawal of consent.

Participants who have withdrawn from the study cannot be rerandomized (treated) in the study. Their inclusion and intervention numbers must not be reused.

7.3 LOST TO FOLLOW UP

A participant will be considered lost to follow-up if he or she repeatedly fails to return for scheduled visits and is unable to be contacted by the study site.

The following actions must be taken if a participant fails to return to the clinic for a required study visit:

- The site must attempt to contact the participant and reschedule the missed visit as soon as possible and counsel the participant on the importance of maintaining the assigned visit schedule and ascertain whether or not the participant wishes to and/or should continue in the study.
- Before a participant is deemed lost to follow up, the Investigator or designee must make every effort to regain contact with the participant (where possible, 3 telephone calls and, if necessary, a certified letter to the participant's last known mailing address or local equivalent methods). These contact attempts should be documented in the participant's medical record.
- Should the participant continue to be unreachable, he/she will be considered to have withdrawn from the study.
- Discontinuation of specific sites or of the study as a whole are handled as part of Section 10.1.

8 STUDY ASSESSMENTS AND PROCEDURES

- Study procedures and their timing are summarized in the SoA (see Section 1.3). Protocol waivers or exemptions are not allowed.
- Adherence to the study design requirements, including those specified in the SoA (see Section 1.3), is essential and required for study conduct.
- All screening evaluations must be completed and reviewed to confirm that potential participants meet all eligibility criteria. The Investigator will maintain a screening log to record details of all participants screened and to confirm eligibility or record reasons for screening failure, as applicable.
- Procedures conducted as part of the participant's routine clinical management (eg, blood count) and obtained before signing of the ICF may be utilized for screening or baseline purposes provided the procedures met the protocol-specified criteria and were performed within the time frame defined in the SoA (see Section 1.3).
- Patient-Reported Outcome questionnaires including NRS should be completed by the participants before the consultation and/or clinical tests, in a quiet place. The questionnaires should be completed by the participants themselves, independently from their physician, the study nurse or any other medical personnel and without any help from friends or relatives.

8.1 EFFICACY ASSESSMENTS

Efficacy data will be collected via electronic devices.

The e-diary is used for daily recording of participant's answers to the WI-NRS, pain-NRS, and sleep-NRS questionnaires. This device will be dispensed at screening visit (Visit 1), including instructions for use. Participants will be instructed on the use of the device.

Recorded information will be downloaded from this device daily. At EOS Visit, the e-diary will be downloaded and returned to the site.

On regular basis, the site staff should review on vendor's website the information downloaded from participants' e-diary. They should particularly check status of the disease reviewing WI-NRS, as well as compliance to background therapy and overall e-diary compliance. The site should follow up with the participant as appropriate.

Participants will fill in the DLQI, HADS, EQ-5D-5L, PGIC, PGIS, and missed school/work days questionnaires during their site visit on a tablet that will be provided to the site. This device is kept at the site during the study.

8.1.1 Worst-itch numeric rating scale

Worst-itch numerical rating scale (WI-NRS) is a PRO comprised of a single item rated on a scale from 0 (“No itch”) to 10 (“Worst imaginable itch”). Participants are asked to rate the intensity of their worst pruritus (itch) over the past 24 hours using this scale.

Participants will complete the WI-NRS daily according to the SoA (see Section 1.3). The assessment tool is provided in Section 10.7.1.

8.1.2 Investigator’s global assessment for prurigo nodularis

Investigator’s global assessment for prurigo nodularis (IGA PN) is a clinician-reported outcome (ClinRO) that allows clinicians to assess the activity of PN (IGA PN-A) using a 5-point scale from 0 (clear) to 4 (severe); and the stage of the disease (IGA PN-S) using a 5-point scale from 0 (clear) to 4 (severe).

Clinicians will complete the IGA PN as described in the SoA (see Section 1.3). The assessment tool is provided in Section 10.7.2.

8.1.3 Prurigo activity score

The PAS is a ClinRO measurement. The original PAS questionnaire Version 0.9 consists of 7 items, developed by expert clinicians in PN (26). The items of the PAS evaluate the pruriginous lesions in terms of:

- Type (visible lesions: Item 1a; predominant lesions: Item 1b);
- Estimated number (Item 2);
- Distribution (Item 3, 4);
- Size (biggest lesion: Item 6a; representative lesion: Item 6b).

Other items evaluate the representative body area and exact number of lesions (Item 5), the activity in terms of percentage of pruriginous lesions with excoriations/crusts on top (reflecting active scratching; Item 7a) and the percentage of healed pruriginous lesions (reflecting healing of chronic prurigo; Item 7b).

A 5-item simplified version of the PAS will be used in the current study. In particular, Item 3 (lesion distribution) and Item 6 (lesion monitoring) of the original PAS were removed, and the response options slightly modified and refined.

Clinicians will complete the screening/baseline version at screening and baseline visits, and will complete the follow-up version of the modified PAS at the other visits, as reported in the SoA (see Section 1.3). The assessment tool is provided in Section 10.7.3.

8.1.4 Dermatology life quality index

The DLQI is a PRO developed to measure dermatology-specific HRQoL in adult patients (27). The instrument comprises 10 items assessing the impact of skin disease on patients' HRQoL over the previous week. The items cover symptoms, leisure activities, work/school or holiday time, personal relationships including intimate, the side effects of treatment, and emotional reactions to having a skin disease. It is a validated questionnaire used in clinical practice and clinical trials (25). Response scale is a 4-point Likert scale (0 = "not at all" and 3 = "very much") for nine items. The remaining one item about work/studying asks whether work/study has been prevented and then (if "No") to what degree the skin condition has been a problem at work/study; the item is rated on a 3-point Likert scale ("Not at all" to "A lot"). Overall scoring ranges from 0 to 30, with a high score indicative of a poor HRQoL.

Participants will complete the questionnaire at time points according to SoA (see Section 1.3). The assessment tool is provided in Section 10.7.4.

8.1.5 Pain and sleep numeric rating scales

Participants will be asked to rate their worst skin pain in the past 24 hours using a 0 to 10 NRS, with 0 = No pain to 10 = Worst pain possible.

In addition, participants will be asked to rate their sleep quality on their past night upon awakening, using a 0 to 10 NRS, with 0 = Worst possible sleep and 10 = Best possible sleep.

Participants will complete the skin pain NRS and sleep quality NRS once a day.

The assessment tools are provided in Section 10.7.7 and Section 10.7.8.

8.1.6 Hospital anxiety and depression scale

The HADS is a PRO instrument for screening anxiety and depression in non-psychiatric populations; repeated administration also provides information about changes to a patient's emotional state (28, 29). The HADS consists of 14 items, 7 each for anxiety and depression symptoms; possible scores range from 0 to 21 for each subscale. The following cut-off scores are recommended for both subscales:

- 0 to 7: normal
- 8 to 10: borderline abnormal (borderline case)
- 11 to 21: abnormal

Participants will complete the questionnaire as described in the SoA (see Section 1.3). The assessment tool is provided in Section 10.7.5.

8.1.7 Patient Global Impression of Change of disease and Patient Global Impression of Severity

The PGIC is a one-item questionnaire that asks patients to provide the overall self-assessment of change in their PN overall on a 7-point scale, compared to just before patient started taking the study injection. Response choices are: 0 = “Very much better”, 1 = “Moderately better”, 2 = “A little better”, 3 = “No change”, 4 = “A little worse”, 5 = “Moderately worse”, 6 = “Very much worse”.

The PGIS is a one-item questionnaire that asks patients to provide the overall self-assessment of their disease severity on a 4-point scale for the past week. Response choices are: 1 = “none”, 2 = “Mild”, 3 = “Moderate”, 4 = “Severe”.

Participants will complete the 2 items as described in the SoA (see Section 1.3). The items are provided in Section 10.7.9 and Section 10.7.10.

8.1.8 Euroqol 5 dimensions questionnaire

The Euroqol-5 dimensions (EQ-5D) is a standardized PRO measure of health status developed by the EuroQOL Group in order to provide a simple, generic measure of health for clinical and economic appraisal (30). The EQ-5D consists of 2 parts: the descriptive system and the EQ visual analog scale (VAS). The EQ-5D descriptive system comprises the following 5 dimensions: mobility, self-care, usual activities, pain/discomfort, and anxiety/depression. Each dimension has 5 levels of perceived problems: “no problem”, “slight problems”, “moderate problems”, “severe problems” and “inability to do the activity”. The respondent is asked to indicate his/her health state by ticking (or placing a cross) in the box against the most appropriate statement in each of the 5 dimensions; this results in a 1-digit number expressing the level for that dimension. The digits for 5 dimensions can be combined in a 5-digit number describing the respondent’s health state. The EQ VAS records the respondent’s self-rated health on a vertical VAS where the endpoints are labeled “best imaginable health state (100)” and “worst imaginable health state (0)”. This information can be used as a quantitative measure of health outcome as judged by the individual respondents.

Participants will complete the questionnaire as described in the SoA (see Section 1.3). The assessment tool is provided in Section 10.7.6.

8.1.9 Missed school/work days

Participants who are employed or enrolled in school will be asked to report the number of sick leave/missed school days since the last study assessment.

Participants will complete the questionnaire as described in the SoA (see Section 1.3). The assessment tool is provided in Section 10.7.11.

8.2 SAFETY ASSESSMENTS

Planned time points for all safety assessments are provided in the SoA (see Section 1.3).

8.2.1 Physical examinations

- A complete physical examination will include, at a minimum, assessments of the skin (full body skin exam), nasal cavities, eyes, ears, respiratory, cardiovascular, gastrointestinal, neurological, lymphatic, and musculoskeletal systems.
- Participants must be disrobed and provided with a hospital gown before the skin examination.
- Investigators should pay special attention to clinical signs related to previous serious illnesses.
- Any new finding or worsening of previous finding should be reported as a new AE

8.2.2 Vital signs

- Vital signs will be measured in a semi-supine or sitting position after 5 minutes rest and will include body temperature, SBP and DBP, and pulse and respiratory rate. Blood pressure and pulse measurements should be assessed using the same arm with a completely automated device. Manual techniques will be used only if an automated device is not available.
- Body weight (kg) will be measured at screening (Visit 1), EOT, and EOS visits. Height will be measured at screening visit (Visit 1). Height and weight should be measured with indoor clothing but without shoes.

8.2.3 Electrocardiograms

- Single 12-lead ECG will be obtained as outlined in the SoA (see Section 1.3) using an ECG machine that automatically calculates the heart rate and measures PR, QRS, QT, and QTcF intervals. Refer to Section 7 for QTcF withdrawal criteria and any additional QTcF readings that may be necessary. The ECG should be recorded after 10 minutes of rest in the supine position.

8.2.4 Clinical safety laboratory assessments

- See Appendix 2 (Section 10.2) for the list of clinical laboratory tests to be performed and to the SoA (see Section 1.3) for the timing and frequency.
- The Investigator must review the laboratory report, document this review, and record any clinically relevant changes occurring during the study in the AE section of the CRF. The laboratory reports must be filed with the source documents. Clinically significant abnormal laboratory findings are those which are not associated with the underlying disease, unless judged by the Investigator to be more severe than expected for the participant's condition.
- All laboratory tests with values considered clinically significantly abnormal during participation in the study or within 12 weeks after the last dose of study intervention should be repeated until the values return to normal or baseline or are no longer considered clinically significant by the Investigator or medical monitor.

- If such values do not return to normal/baseline within a period of time judged reasonable by the Investigator, the etiology should be identified and the Sponsor notified.
- All protocol-required laboratory assessments, as defined in Appendix 2 (Section 10.2), must be conducted in accordance with the laboratory manual and the SoA (see Section 1.3).
- If laboratory values from non-protocol specified laboratory assessments performed at the institution's local laboratory require a change in participant management or are considered clinically significant by the Investigator (eg, SAE or AE or dose modification), then the results must be recorded in the CRF.

8.3 ADVERSE EVENTS AND SERIOUS ADVERSE EVENTS

Adverse event of special interest

An AESI is an AE (serious or nonserious) of scientific and medical concern specific to the Sponsor's product or program, for which ongoing monitoring and immediate notification by the Investigator to the Sponsor is required. Such events may require further investigation in order to characterize and understand them. Adverse events of special interest may be added, modified or removed during a study by protocol amendment.

For these AESIs, the Sponsor will be informed immediately (ie, within 24 hours), per SAE notification described in Section 10.3, even if not fulfilling a seriousness criterion, using the corresponding pages in the CRF (to be sent) or screens in the eCRF. If an SAE or any AESI of anaphylactic reactions or systemic allergic reactions that are related to IMP and require treatment, or severe injection site reactions lasting longer than 24 hours, occurs in a participant, blood samples should be collected for determination of functional dupilumab concentration, and ADA assessment at or near the onset and completion of the occurrence of the event, if possible.

- Anaphylactic reactions
- Systemic hypersensitivity reactions
- Helminthic infections
- Any type of conjunctivitis or blepharitis (severe or serious)
- Keratitis
- Clinically symptomatic eosinophilia (or eosinophilia associated with clinical symptoms)
- Pregnancy of a female subject entered in a study as well as pregnancy occurring in a female partner of a male subject entered in a study with IMP/NIMP;
  - Pregnancy occurring in a female participant entered in the clinical trial or in a female partner of a male participant entered in the clinical trial. It will be qualified as an SAE only if it fulfills one of the seriousness criteria.
  - In the event of pregnancy in a female participant, IMP should be discontinued.

- Follow-up of the pregnancy in a female participant or in a female partner of a male participant is mandatory until the outcome has been determined
- Abnormal pregnancy outcomes (eg, spontaneous abortion, fetal death, stillbirth, congenital anomalies, ectopic pregnancy) are considered SAEs

- Significant ALT elevation
  - ALT >5 x ULN in participants with baseline ALT ≤2 x ULN;

    OR
  - ALT >8 x ULN if baseline ALT >2 x ULN
- Symptomatic overdose (serious or nonserious) with IMP/NIMP
  - An overdose (accidental or intentional) with the IMP is an event suspected by the Investigator or spontaneously notified by the participant and defined as at least twice the intended dose during an interval of less than 11 days. The circumstances (ie, accidental or intentional) should be clearly specified in the verbatim and symptoms, if any, entered on separate AE forms.
  - An overdose (accidental or intentional) with any NIMP is an event suspected by the Investigator or spontaneously notified by the participant and defined as at least twice the maximum prescribed daily dose, within the intended therapeutic interval. The circumstances (ie, accidental or intentional) should be clearly specified in the overdose form.

The definitions of an AE or SAE can be found in Appendix 3 (Section 10.3).

Adverse events will be reported by the participant (or, when appropriate, by a caregiver, surrogate, or the participant's legally authorized representative).

The Investigator and any qualified designees are responsible for detecting, documenting, and recording events that meet the definition of an AE or SAE and remain responsible for following up AEs that are serious, considered related to the study intervention or study procedures, or that caused the participant to discontinue the study intervention (see Section 7).

8.3.1 Time period and frequency for collecting AE and SAE information

All AEs, serious or nonserious, will be collected from the signing of the ICF until the EOS visit at the time points specified in the SoA (Section 1.3).

All SAEs and AESI will be recorded and reported to the Sponsor or designee immediately and under no circumstance should this exceed 24 hours, as indicated in Appendix 3 (Section 10.3). The Investigator will submit any updated SAE data to the Sponsor within 24 hours of it being available.

Investigators are not obligated to actively seek AE or SAE after conclusion of the study participation. However, if the Investigator learns of any SAE, including a death, at any time after a participant has been discharged from the study, and he/she considers the event to be reasonably related to the study intervention or study participation, the Investigator must promptly notify the Sponsor.

8.3.2 Method of detecting AEs and SAEs

The method of recording, evaluating, and assessing causality of AE and SAE and the procedures for completing and transmitting SAE reports are provided in Appendix 3 (Section 10.3).

Care will be taken not to introduce bias when detecting AEs and/or SAEs. Open-ended and non-leading verbal questioning of the participant is the preferred method to inquire about AE occurrences.

8.3.3 Follow-up of AEs and SAEs

After the initial AE/AESI/SAE report, the Investigator is required to proactively follow each participant at subsequent visits/contacts. At the pre-specified study end-date, all SAEs and nonserious AESI (as defined in Section 8.3) will be followed until resolution, stabilization, the event is otherwise explained, or the participant is lost to follow-up (as defined in Section 7.3). Further information on follow-up procedures is given in Appendix 3 (Section 10.3).

8.3.4 Regulatory reporting requirements for SAEs

- Prompt notification by the Investigator to the Sponsor of a SAE is essential so that legal obligations and ethical responsibilities towards the safety of participants and the safety of a study intervention under clinical investigation are met.
- The Sponsor has a legal responsibility to notify both the local regulatory authority and other regulatory agencies about the safety of a study intervention under clinical investigation. The Sponsor will comply with country-specific regulatory requirements relating to safety reporting to the regulatory authority, Institutional Review Boards (IRB:)/Independent Ethics Committees (IEC), and Investigators.
- Investigator safety reports must be prepared for suspected unexpected serious adverse reactions (SUSAR) according to local regulatory requirements and Sponsor policy and forwarded to Investigators as necessary.
- Adverse events that are considered expected will be specified in the reference safety information.
- An Investigator who receives an Investigator safety report describing a SAE or other specific safety information (eg, summary or listing of SAEs) from the Sponsor will review and then file it along with the IB and will notify the IRB/IEC, if appropriate according to local requirements.

8.3.5 Pregnancy

- Details of all pregnancies in female participants and female partners of male participants will be collected after the start of study intervention and until the outcome has been determined.

- If a pregnancy is reported, the Investigator should inform the Sponsor within 24 hours of learning of the pregnancy and should follow the procedures outlined in Appendix 4 (Section 10.4).
- Abnormal pregnancy outcomes (eg, spontaneous abortion, fetal death, stillbirth, congenital anomalies, ectopic pregnancy) are considered SAEs.

8.3.6 Guidelines for reporting product complaints

Any defect in the IMP/NIMP/device must be reported as soon as possible by the Investigator to the monitoring team that will complete a product complaint form within required timelines.

Appropriate information (eg, samples, labels or documents like pictures or photocopies) related to product identification and to the potential deficiencies may need to be gathered. The Investigator will assess whether or not the quality issue has to be reported together with an AE or SAE.

8.4 TREATMENT OF OVERDOSE

Overdose is an AESI (defined in Section 8.3). No antidote is available for dupilumab. Sponsor does not recommend specific treatment for an overdose.

In the event of an overdose, the Investigator/treating physician should:

1. Provide symptomatic care.
2. Contact the Medical Monitor immediately.
3. Closely monitor the participant for any AE/SAE and laboratory abnormalities until dupilumab can no longer be detected systemically.
4. Obtain a plasma sample for PK analysis as soon as possible if requested by the Medical Monitor (determined on a case-by-case basis).
5. Document appropriately in the CRF.

Decisions regarding dose interruptions will be made by the Investigator in consultation with the Medical Monitor based on the clinical evaluation of the participant.

8.5 PHARMACOKINETICS

8.5.1 Systemic drug concentration and antidrug antibodies

8.5.1.1 Sampling time

Blood samples will be collected for determination of functional dupilumab and anti-dupilumab antibodies in serum as specified in the SoA (Section 1.3). Special procedures for collection, storage, and shipping of serum are described in separate operational manuals. The date of collection should be recorded in the eCRF.

8.5.1.2 Handling procedures

Special procedures for collection, storage, and shipping of serum are described in separate operational manuals. An overview of handling procedure for samples used in the determination of systemic drug concentration and ADA is provided in Table 3.

Table 3 - Summary of handling procedures

Sample type	Functional dupilumab	Anti-dupilumab antibody
Matrix	Serum	Serum
Blood sample volume	5 mL	5 mL
Anticoagulant	None	None
Blood handling procedures	See Operational Manual	See Operational Manual
Serum aliquot split	2 aliquots	2 aliquots
Storage conditions	<6 months: below -20°C<br><24 months: below -80°C (preferred)	<6 months: below -20°C<br><24 months: below -80°C (preferred)
Serum shipment condition	In dry ice	In dry ice

8.5.1.3 Bioanalytic method

Serum PK and ADA samples will be assayed using validated methods as described in Table 4.

Table 4 - Summary of bioanalytical methods for functional dupilumab and anti-dupilumab antibodies

[redacted]

Note: In the event of any SAE, any AE of severe injection site reaction lasting longer than 24 hours, or any AESI of anaphylactic reaction or systemic allergic reaction that is related to IMP and require treatment, PK and ADA samples will be collected at or near the onset of the event for any additional analysis if required or for archival purposes. The exact date and time of sample collection must be recorded and entered into the database by the central laboratory. An unscheduled systemic drug concentration page in the eCRF must be completed as well. If necessary for safety monitoring, additional PK and ADA samples may be collected after the EOS Visit until resolution of AE.

Specifically for PK, any changes in the timing or addition of time points for any planned study assessments must be documented and approved by the relevant study team member and then archived in the Sponsor and site study files, but will not constitute a protocol amendment. The IRB/IEC will be informed of any safety issues that require alteration of the safety monitoring scheme or amendment of the ICF.

8.6 PHARMACODYNAMICS

See Section 8.8 for IgE measurement. No other PD parameters will be evaluated in this study.

8.7 GENETICS

For those participants who consent to the optional pharmacogenetic/pharmacogenomic sample collection section of the ICF, blood samples for exploratory genetic analysis of DNA or RNA will be collected and stored for possible future use (see Section 10.5). Participation is optional. Participants who do not wish to participate in the genetic research may still participate in the study.

In the event of DNA extraction failure, a replacement genetic blood sample may be requested from the participant.

Details on processes for collection and shipment and destruction of these samples can be found in the laboratory manual.

8.8 BIOMARKERS

- Venous blood samples will be collected for measurement of total serum IgE. Total IgE will be measured using validated quantitative methods.
- Optional samples for biomarker research that should be collected from participants in the study with their consent include:
    - Serum and plasma: samples will be collected and stored for possible future analysis of potential biomarkers of drug response, disease activity, safety, and Type 2 inflammation.

Plasma and serum samples may be stored for a maximum of 5 years, and DNA or RNA samples for 15 years (or according to local regulations) following the last participant's last visit for the study at a facility selected by the Sponsor.

8.9 MEDICAL RESOURCE UTILIZATION AND HEALTH ECONOMICS

Number of missed school/work days collection is described in Section 8.1.9.

9 STATISTICAL CONSIDERATIONS

9.1 STATISTICAL HYPOTHESES

The statistical hypotheses for comparing dupilumab to placebo on the primary endpoint of the proportion of participants with WI-NRS reduction of ≥4 (minimally important difference) at Week 12 are as follows:

- Null hypothesis H0: No treatment difference between dupilumab and placebo
- Alternative hypothesis H1: There is a treatment difference between dupilumab and placebo

The statistical hypotheses to be tested on secondary endpoints can be specified similarly to those on the primary endpoint.

9.2 SAMPLE SIZE DETERMINATION

The primary endpoint is the proportion of participants with WI-NRS reduction of ≥4 from baseline to Week 12. By assuming the response rate is [redacted]% and [redacted]% in placebo and dupilumab respectively, 56 participants/arm will provide 90% power to detect the difference of [redacted]% between dupilumab and placebo with Fisher exact test at 2-sided level of 0.05. Assuming 15% drop out during the 12 weeks of treatment, the target is to randomize 75 participants/arm with a cap of up to 10% of participants in the atopic population having active mild AD.

Approximately 150 participants will be randomized 1:1. This corresponds to approximately 75 participants that will be randomly assigned to each intervention arm.

The sample size calculation was performed using SAS 9.4 power procedure. The assumptions were based on the effect of dupilumab versus placebo in WI-NRS reduction ≥4 at Week 16 observed in patients with moderate to severe AD as seen in studies of AD-1334 (Solo1) and AD-1416 (Solo2).

Participants will be randomized to dupilumab or placebo in 1:1 ratio with stratification factors of documented history of atopy (atopic or non-atopic, see definition in Section 6.3), stable use of TCS/TCI (yes or no), and country/territory code. Both the atopic and the non-atopic PN population will be capped at 60% of the total enrolled population.

9.3 POPULATIONS FOR ANALYSES

For purposes of analysis, the following populations are defined (Table 5):

Table 5 - Populations for analyses

Population	Description
Screened	All participants who sign the ICF.
Randomized	The randomized population includes all participants with a treatment kit number allocated and recorded in the IRT database, and regardless of whether the treatment kit was used or not.<br>Participants treated without being randomized will not be considered randomized and will not be included in any efficacy population.
Intent-to-treat (ITT)	All randomized participants analyzed according to the treatment group allocated by randomization regardless if treatment kit is used or not
Efficacy	The ITT population
Safety	All participants randomly assigned to study intervention and who take at least 1 dose of study intervention. Participants will be analyzed according to the intervention they actually received.<br>Randomized participants for whom it is unclear whether they took the study medication will be included in the safety population as randomized.<br>For participants who accidentally receive different treatment from the planned, the actual intervention allocation for as-treated analysis will be the dupilumab group.<br>The PD analyses will be performed on the safety population.
Pharmacokinetic (PK)	The PK population includes all participants in the safety population with at least one non-missing result for functional dupilumab concentration in serum after first dose of the study treatment. Participants will be analyzed according to the intervention actually received.
Antidrug antibody (ADA)	ADA population includes all participants in the safety population who have at least one non-missing ADA result after first dose of the study treatment. Participants will be analyzed according to the intervention actually received.

ADA: antidrug antibody; ICF: informed consent form; ITT: intent to treat; PD: pharmacodynamics: PK: pharmacokinetic

9.4 STATISTICAL ANALYSES

The SAP will be developed and finalized before primary database lock and will describe the participant populations to be included in the analyses, and procedures for accounting for missing, unused, and spurious data. This section is a summary of the planned statistical analyses of the primary and secondary endpoints.

9.4.1 Efficacy analyses

The multiplicity procedure is proposed to control the overall Type-I error rate for testing the primary endpoint and the key secondary endpoints. Detailed hierarchical testing procedure will be defined in the study SAP. The study is considered positive when the primary endpoint achieves statistical significance with two-sided significance level 0.05.

Table 6 - Efficacy analyses

Endpoint	Statistical analysis methods
**Primary**:	
Proportion of participants with improvement (reduction) in WI-NRS by ≥4 from baseline to Week 12	The primary analysis will be conducted by using CMH test stratifying by stratification factors of documented history of atopy (atopic or non-atopic), stable use of TCS/TCI (yes or no), and region (countries combined).<br>For participants discontinuing the study treatment before Week 12, their off-study treatment values measured up to Week 12 will be included in the analysis. Participants taking selected prohibited medications and/or rescue medications (details of selection will be provided in the SAP) prior to Week 12 or have missing data at Week 12 will be considered non-responders.<br>*Sensitivity analyses:*<br>The data collected after taking selected prohibited medications and/or rescue medications will not be censored and included in the sensitivity analysis to evaluate the robustness of the primary analysis results with respect to the method of handling data while taking selected prohibited medications. Details of the sensitivity analyses will be provided in the SAP.<br>*Subgroup analyses:*<br>To assess the consistency in treatment effects across different subgroup levels, subgroup analyses will be performed for the primary efficacy endpoint with respect to documented history of atopy (atopic or non-atopic), age group, gender, region, and other factors that will be specified in the SAP. A subgroup analysis will be performed excluding participants with a current diagnosis of AD.
**Secondary:**	
**[Key secondary endpoint]** Proportion of participants with improvement (reduction) in WI-NRS by ≥4 from baseline to Week 24.<br>Proportion of participants with WI-NRS reduction ≥4 over time until Week 24.<br>Proportion of participants with IGA PN-S 0/1 score at Week 4, Week 8, Week 12, and Week 24.<br>Proportion of participants with IGA PN-A 0/1 score at Week 4, Week 8, Week 12, and Week 24.	Secondary efficacy endpoints that measure binary responses will be analyzed in the same fashion as the primary endpoint.
Time to onset of effect on pruritus as measured by proportion of participants with improvement (reduction) in WI-NRS by ≥4 from baseline during the 24-week treatment period.	This time-to-event endpoint will be analyzed using the Cox proportional hazards model, including treatment, stratification factors (countries combined to region). The hazards ratio, its 95% confidence interval and p-value will be reported. Kaplan-Meier curves will be also provided.

VV-CLIN-0[illegible] [illegible]

Change from baseline in WI-NRS at Week 12 and Week 24.<br>Percent change from baseline in WI-NRS at Week 12 and Week 24.<br>Percent change from baseline in WI-NRS over time until Week 24.<br>Change from baseline in IGA PN-S score at Week 4, Week 8, Week 12, and Week 24.<br>Change from baseline in HRQoL, as measured by DLQI to Week 12 and Week 24<br>Onset of action in change from baseline in WI-NRS (first p <0.05 difference from placebo in the daily WI-NRS that remains significant at subsequent measurements) until Week 12.	Continuous secondary efficacy endpoints will be analyzed using a hybrid method of the WOCF and multiple imputations.<br>Data of participants taking selected prohibited medications and/or rescue medications will be set to missing after the medication usage, and the worst postbaseline value on or before the time of the medication usage will be used to impute missing endpoint value (for participants whose postbaseline values are all missing, the baseline will be used to impute).<br>Participants who discontinue the treatment prematurely are encouraged to follow the planned clinical visits and in those participants who did not take the selected prohibited medications and/or rescue medications, all data collected after treatment discontinuation will be used in the analysis. For these participants, missing data may still happen despite all efforts have been tried to collect the data after treatment discontinuation. For participants who discontinue due to lack of efficacy, all data collected after discontinuation will be used in the analysis, and a WOCF approach will be used to impute missing data if needed. For participants who discontinued not due to lack of efficacy, a multiple imputation approach will be used to impute missing endpoint value, and this multiple imputation will use all participants excluding patients who have taken the selected prohibited medications and/or rescue medications prior to timepoint of endpoint of interest and excluding participants who discontinue due to lack of efficacy.<br>Each of the imputed complete data will be analyzed by fitting an ANCOVA model with treatment group, stratification factors of documented history of atopy (atopic or non-atopic), stable use of TCS/TCI (yes or no), and region (countries combined), and relevant baseline measurement as covariates included in the model. Statistical inference obtained from all imputed data will be combined using Rubin's rule. Descriptive statistics including number of subjects, mean, standard error, and least squares (LS) mean change and standard error will be provided. In addition, difference of the dupilumab group against placebo in LS means and the corresponding 95% confidence interval will be provided along with the p-values.<br>Detailed analyses will be described in the SAP finalized before database lock.
**Tertiary/exploratory**	Will be described in the SAP finalized before database lock.

ANCOVA: analysis of covariance; CMH: Cochran-Mantel-Haenszel; DLQI: Dermatology Life Quality Index; IGA: Investigator's global assessment; LS: Least squares; NRS: numeric rating scale; PAS: prurigo activity score; WI-NRS: worst itch numeric rating scale.

9.4.2 Safety analyses

All safety analyses will be performed on the Safety Population. The summary of safety results will be presented by treatment group. The baseline value is defined generally as the last available value before randomization.

Table 7 - Safety analyses

Endpoint	Statistical analysis methods
Percentage of participants experiencing TEAEs or SAEs from baseline through Week 24	Adverse event incidence tables will present by SOC (sorted by internationally agreed order), and PT sorted in alphabetical order for each treatment group, the number (n) and percentage (%) of participants experiencing an AE. Multiple occurrences of the same event in the same participant will be counted only once in the tables within a treatment phase. The denominator for computation of percentages is the safety population within each treatment group.<br>Proportion of participants with at least one TEAE, treatment emergent SAE, TEAE leading to death, and TEAE leading to permanent treatment discontinuation will be tabulated by treatment group. In addition TEAEs will be described according to maximum intensity and relation to the study intervention. Serious AEs and AEs leading to study discontinuation that occur outside the treatment-emergent period will be summarized separately.

AE: adverse event; PT: preferred term; SOC: system organ class; SAE: serious adverse event; TEAE: treatment-emergent adverse event

9.4.3 Other analyses

Table 8 - Other analyses

Endpoint	Statistical analysis methods
Incidence of treatment-emergent ADA against dupilumab over time	The ADA analysis will be conducted on ADA population. ADA variables including treatment-emergent ADA will be summarized using descriptive statistics by treatment group. Drug concentration data will be examined and the influence of ADAs on individual concentration-time profiles will be evaluated. Assessment of the potential impact of ADA on safety and efficacy may be provided.

ADA: antidrug antibodies

Other PK, PD, biomarker, and outcome measures exploratory analyses will be described in the SAP finalized before database lock. The population PK analysis and PD analyses will be presented separately from the main clinical study report (CSR).

9.5 INTERIM ANALYSES

No interim analysis is planned.

A primary database lock will be performed when all randomized participants in this study have completed their 24-week treatment phase. Final analyses in the CSR will be based on this database.

The database will be updated at the end of the study for all participants to include the post-treatment follow-up information and updates for the events previously ongoing at the time of the primary lock. Additional data between this database lock and last participant completing last visit will be summarized in a CSR addendum.

More details will be described in the SAP.

9.5.1 Data Monitoring Committee (DMC)

Due to extensive safety record of the post marked IMP (dupilumab), it is not planned to have a DMC for this study.

9.5.2 Unblinding plan

The unblinding plan will be detailed in the SAP or in a separate unblinding plan.

10 SUPPORTING DOCUMENTATION AND OPERATIONAL CONSIDERATIONS

10.1 APPENDIX 1: REGULATORY, ETHICAL, AND STUDY OVERSIGHT CONSIDERATIONS

10.1.1 Regulatory and Ethical Considerations

- This study will be conducted in accordance with the protocol and with the following:
  - Consensus ethical principles derived from international guidelines including the Declaration of Helsinki and the applicable amendments and Council for International Organizations of Medical Sciences (CIOMS) International Ethical Guidelines.
  - Applicable ICH GCP Guidelines.
  - Applicable laws and regulations.
- The protocol, protocol amendments, ICF, Investigator Brochure, and other relevant documents (eg, advertisements) must be submitted to an IRB/IEC by the Investigator and reviewed and approved by the IRB/IEC before the study is initiated.
- Any amendments to the protocol will require IRB/IEC approval before implementation of changes made to the study design, except for changes necessary to eliminate an immediate hazard to study participants.
- The Investigator will be responsible for the following:
  - Providing written summaries of the status of the study to the IRB/IEC annually or more frequently in accordance with the requirements, policies, and procedures established by the IRB/IEC.
  - Notifying the IRB/IEC of SAEs or other significant safety findings as required by IRB/IEC procedures.
  - Providing oversight of the conduct of the study at the site and adherence to requirements of 21 Code of Federal Regulations (CFR), ICH guidelines, the IRB/IEC, European regulation 536/2014 for clinical studies (if applicable), and all other applicable local regulations.

10.1.2 Financial disclosure

Investigators and sub-Investigators will provide the Sponsor with sufficient, accurate financial information as requested to allow the Sponsor to submit complete and accurate financial certification or disclosure statements to the appropriate regulatory authorities. Investigators are responsible for providing information on financial interests during the course of the study and for 1 year after completion of the study.

10.1.3 Informed consent process

- The Investigator or his/her representative will explain the nature of the study to the participant or his/her legally authorized representative and answer all questions regarding the study.
- Participants must be informed that their participation is voluntary. Participants will be required to sign a statement of informed consent that meets the requirements of 21 CFR 50, local regulations, ICH guidelines, Health Insurance Portability and Accountability Act requirements, where applicable, and the IRB/IEC or study center.
- The medical record must include a statement that written informed consent was obtained before the participant was enrolled in the study and the date the written consent was obtained. The authorized person obtaining the informed consent must also sign the ICF.
- Participants must be re-consented to the most current version of the ICF(s) during their participation in the study.
- A copy of the ICF(s) must be provided to the participant or the participant's legally authorized representative.

Participants who are rescreened are required to sign a new ICF.

The ICF will contain a separate section that addresses the use of remaining mandatory samples as well as additional serum/plasma samples for optional exploratory research. The Investigator or authorized designee will explain to each participant the objectives of the exploratory research. Participants will be told that they are free to refuse to participate and may withdraw their consent at any time and for any reason during the storage period. A separate signature will be required to document a participant's agreement to allow any remaining specimens to be used for exploratory research. Participants who decline to participate in this optional research will not provide this separate signature.

The ICF will also contains a separate section concerning the other optional procedure (genetic testing [DNA/RNA sampling]). A separate signature will be required to document a participant's agreement to this procedure. Participants who decline to participate will not provide this separate signature. A specific consent will be provided for HIV testing if specific consent is locally required.

10.1.4 Data protection

All personal data collected related to participants, Investigators, or any person involved in the study, which may be included in the Sponsor's databases, shall be treated in compliance with all applicable laws and regulations including the Global Data Protection Regulation.

Data collected must be adequate, relevant and not excessive, in relation to the purposes for which they are collected. Each category of data must be properly justified and in line with the study objective.

Participant race and ethnicity will be collected in this study because these data are required by regulatory agencies (eg, for reporting effectiveness data by racial subgroup in marketing authorization applications for FDA, or to report on the Japanese population for the Pharmaceuticals and Medical Devices Agency in Japan). In addition, race has been identified to influence PN incidence (15).

- Participants will be assigned a unique identifier by the Sponsor. Any participant records or datasets that are transferred to the Sponsor will contain the identifier only; participant names or any information which would make the participant identifiable will not be transferred.
- The participant must be informed that his/her personal study-related data will be used by the Sponsor in accordance with local data protection law. The level of disclosure must also be explained to the participant.
- The participant must be informed that his/her medical records may be examined by Clinical Quality Assurance auditors or other authorized personnel appointed by the Sponsor, by appropriate IRB/IEC members, and by inspectors from regulatory authorities.
- When archiving or processing personal data pertaining to the Investigator and/or to the participants, the Sponsor shall take all appropriate measures to safeguard and prevent access to this data by any unauthorized third party.

10.1.5 Committees structure

There will be no study committees.

10.1.6 Dissemination of clinical study data

Sanofi shares information about clinical trials and results on publically accessible websites, based on company commitments, international and local legal and regulatory requirements, and other clinical trial disclosure commitments established by pharmaceutical industry associations. These websites include clinicaltrials.gov, EU clinicaltrialregister (eu.ctr), and sanofi.com, as well as some national registries.

In addition, results from clinical trials in patients are required to be submitted to peer-reviewed journals following internal company review for accuracy, fair balance and intellectual property. For those journals that request sharing of the analyzable data sets that are reported in the publication, interested researchers are directed to submit their request to clinicalstudydatarequest.com.

Individual participant data and supporting clinical documents are available for request at clinicalstudydatarequest.com. While making information available we continue to protect the privacy of participants in our clinical trials. Details on data sharing criteria and process for requesting access can be found at this web address: clinicalstudydatarequest.com.

10.1.7 Data quality assurance

- All participant data relating to the study will be recorded on printed or electronic CRF unless transmitted to the Sponsor or designee electronically (eg, laboratory data). The Investigator is responsible for verifying that data entries are accurate and correct by physically or electronically signing the CRF.
- The Investigator must maintain accurate documentation (source data) that supports the information entered in the CRF.
- The Investigator must permit study-related monitoring, audits, IRB/IEC review, and regulatory agency inspections and provide direct access to source data documents.
- Monitoring details describing strategy (eg, risk-based initiatives in operations and quality such as Risk Management and Mitigation Strategies and Analytical Risk-Based Monitoring), methods, responsibilities and requirements, including handling of noncompliance issues and monitoring techniques (central, remote, or on-site monitoring) are provided in separate study documents.
- The Sponsor or designee is responsible for the data management of this study including quality checking of the data.
- The Sponsor assumes accountability for actions delegated to other individuals (eg, Contract Research Organizations).
- Study monitors will perform ongoing source data verification to confirm that data entered into the CRF by authorized site personnel are accurate, complete, and verifiable from source documents; that the safety and rights of participants are being protected; and that the study is being conducted in accordance with the currently approved protocol and any other study agreements, ICH GCP, and all applicable regulatory requirements.
- Records and documents, including signed ICFs, pertaining to the conduct of this study must be retained by the Investigator for 25 years after the signature of the final study report unless local regulations or institutional policies require a longer retention period. No records may be destroyed during the retention period without the written approval of the Sponsor. No records may be transferred to another location or party without written notification to the Sponsor.

10.1.8 Source documents

- Source documents provide evidence for the existence of the participant and substantiate the integrity of the data collected. Source documents are filed at the Investigator's site.
- Data reported on the CRF or entered in the eCRF that are transcribed from source documents must be consistent with the source documents or the discrepancies must be explained. The Investigator may need to request previous medical records or transfer records, depending on the study. Also, current medical records must be available.

- Source data is all information in original records and certified copies of original records of clinical findings, observations, or other activities in a clinical trial necessary for the reconstruction and evaluation of the trial. Source data are contained in source documents. Source documents are original documents, data and records such as hospital records, clinic and office charts, laboratory notes, memoranda, pharmacy dispensing records, recorded data from automated instruments, etc.

10.1.9 Study and site closure

The Sponsor or designee reserves the right to close the study site or terminate the study at any time for any reason at the sole discretion of the Sponsor. Study sites will be closed upon study completion. A study site is considered closed when all required documents and study supplies have been collected and a study-site closure visit has been performed.

The Investigator may initiate study-site closure at any time, provided there is reasonable cause and sufficient notice is given in advance of the intended termination.

Reasons for study termination by the Sponsor, as well as reasons for the early closure of a study site by the Sponsor or Investigator may include but are not limited to:

- For study termination:
  - Information on the product leads to doubt as to the benefit/risk ratio
  - Discontinuation of further study intervention development
- For site termination:
  - Failure of the Investigator to comply with the protocol, the requirements of the IRB/IEC or local health authorities, the Sponsor’s procedures, or GCP guidelines
  - Inadequate or no recruitment (evaluated after a reasonable amount of time) of participants by the Investigator
  - Total number of participants included earlier than expected

10.1.10 Publication policy

- The results of this study may be published or presented at scientific meetings. If this is foreseen, the Investigator agrees to submit all manuscripts or abstracts to the Sponsor before submission. This allows the Sponsor to protect proprietary information and to provide comments.
- The Sponsor will comply with the requirements for publication of study results. In accordance with standard editorial and ethical practice, the Sponsor will generally support publication of multicenter studies only in their entirety and not as individual site data. In this case, a coordinating Investigator will be designated by mutual agreement.
- Authorship will be determined by mutual agreement and in line with International Committee of Medical Journal Editors authorship requirements.

10.2 APPENDIX 2: CLINICAL LABORATORY TESTS

The tests detailed in Table 9 will be performed by the central laboratory.

- Local laboratory results are only required in the event that the central laboratory results are not available in time for either study intervention administration and/or response evaluation. If a local sample is required, it is important that the sample for central analysis is obtained at the same time. Additionally, if the local laboratory results are used to make either a study intervention decision or response evaluation, the results must be entered into the CRF.
- Protocol-specific requirements for inclusion or exclusion of participants are detailed in Section 5 of the protocol.
- Additional tests may be performed at any time during the study as determined necessary by the Investigator or required by local regulations.
- Additional serum or urine pregnancy tests may be performed, as determined necessary by the investigator or required by local regulation, to establish the absence of pregnancy at any time during the subject's participation in the study.

Table 9 - Protocol-required laboratory assessments

Laboratory assessments	Parameters
Hematology	
	Platelet count
	RBC count
	Hemoglobin
	Hematocrit
	WBC count with differential:
	Neutrophils
	Lymphocytes
	Monocytes
	Eosinophils
	Basophils
	CD4 T cell count (for participants with HIV history only)
Clinical chemistry[a]	
	BUN
	Creatinine
	Glucose
	Lactate dehydrogenase
	Uric acid
	Total cholesterol
	Potassium

Laboratory assessments	Parameters
	Sodium<br>Chloride<br>Bicarbonate<br>AST/SGOT<br>ALT/SGPT<br>Alkaline phosphatase<br>Creatine phosphokinase<br>Total bilirubin<br>Total protein<br>Albumin
Routine urinalysis	• Specific gravity<br>• pH, glucose, protein, blood, ketones, bilirubin, urobilinogen, nitrite, leukocyte esterase by dipstick<br>• Creatinine and leukotriene (central laboratory)<br>• Microscopic examination (if blood or protein is abnormal)
Other screening tests	• Highly sensitive serum (at screening) or urine (at other timepoints) hCG pregnancy test (as needed for WOCBP)[b]<br>• Serology[c]: HBs Ag, HBs Ab, HBc Ab, HCV Ab, HIV screen (Anti-HIV-1 and HIV-2 antibodies); HIV viral load test (for participants with HIV history only)<br>• Tuberculosis test<br>• All study-required laboratory assessments will be performed by a central laboratory, with the exception of TB test, urine pregnancy test, and routine urinalysis.

NOTES:

a Details of liver chemistry stopping criteria and required actions and follow-up assessments after liver stopping or monitoring event are given in Section 7.1 and Section 10.6. All events which may indicate severe liver injury (meeting Hy's Law laboratory criteria; ALT or AST >3 x ULN and total bilirubin >2 x ULN) must be reported as an SAE.

b After screening, local urine testing will be standard for the protocol unless serum testing is required by local regulation or IRB/IEC.

c In case of results showing HBs Ag (negative), HBs Ab (negative) and HBc Ab (positive), an HBV DNA testing may be performed prior to randomization to rule out a false positivity if the Investigator believes the patient is a false positive, or to clarify the serological status if the Investigator finds it unclear to interpret in absence of known HBV infection. In case of results showing HCV Ab (positive), an HCV RNA testing may be performed to rule out a false positivity, if the Investigator believes the patient is a false positive.

ALT: alanine aminotransferase; AST: aspartate aminotransferase; BUN: blood urea nitrogen; DNA: deoxyribonucleic acidHBc Ab: hepatitis B core antibody; HBs Ab: hepatitis B surface antibody; HBs Ag: hepatitis B surface antigen; HBV: hepatitis B virus; hCG: human chorionic gonadotropin; HCV: hepatitis C virus; HCV Ab: hepatitis C virus antibody; HIV: human immunodeficiency virus; IEC: Independent Ethics Committee; IRB: Institutional Review Board; RBC: red blood cells; RNA: ribonucleic acid; SAE: serious adverse event; SGOT: serum glutamic-oxaloacetic transaminase; SGPT: serum glutamic-pyruvic transaminase; TB: tuberculosis; ULN: upper limit of normal; WBC: white blood cells; WOCBP: woman of childbearing potential

Investigators must document their review of each laboratory safety report.

Laboratory/analyte results that could unblind the study will not be reported to investigative sites or other blinded personnel until the study has been unblinded.

10.3 APPENDIX 3: ADVERSE EVENTS: DEFINITIONS AND PROCEDURES FOR RECORDING, EVALUATING, FOLLOW-UP, AND REPORTING

DEFINITION OF AE

AE definition

- An AE is any untoward medical occurrence in a patient or clinical study participant, temporally associated with the use of study intervention, whether or not considered related to the study intervention.

  NOTE: An AE can therefore be any unfavorable and unintended sign (including an abnormal laboratory finding), symptom, or disease (new or exacerbated) temporally associated with the use of study intervention.

Events meeting the AE definition

- Any abnormal laboratory test results (hematology, clinical chemistry, or urinalysis) or other safety assessments (eg, ECG, radiological scans, vital signs measurements), including those that worsen from baseline, considered clinically significant in the medical and scientific judgment of the Investigator (ie, not related to progression of underlying disease).
- Exacerbation of a chronic or intermittent pre-existing condition including either an increase in frequency and/or intensity of the condition.
- New conditions detected or diagnosed after study intervention administration even though it may have been present before the start of the study.
- Signs, symptoms, or the clinical sequelae of a suspected drug-drug interaction.
- Signs, symptoms, or the clinical sequelae of a suspected overdose of either study intervention or a concomitant medication.
- "Lack of efficacy" or "failure of expected pharmacological action" per se will not be reported as an AE or SAE. Such instances will be captured in the efficacy assessments. However, the signs, symptoms, and/or clinical sequelae resulting from lack of efficacy will be reported as AE or SAE if they fulfil the definition of an AE or SAE.

Events NOT meeting the AE definition

- Any clinically significant abnormal laboratory findings or other abnormal safety assessments which are associated with the underlying disease, unless judged by the Investigator to be more severe than expected for the participant's condition.
- The disease/disorder being studied or expected progression, signs, or symptoms of the disease/disorder being studied, unless more severe than expected for the participant's condition.
- Medical or surgical procedure (eg, endoscopy, appendectomy): the condition that leads to the procedure is the AE.

- Situations in which an untoward medical occurrence did not occur (social and/or convenience admission to a hospital).
- Anticipated day-to-day fluctuations of pre-existing disease(s) or condition(s) present or detected at the start of the study that do not worsen.

DEFINITION OF SAE

If an event is not an AE per definition above, then it cannot be an SAE even if serious conditions are met (eg, hospitalization for signs/symptoms of the disease under study, death due to progression of disease).

A SAE is defined as any untoward medical occurrence that, at any dose:

a) **Results in death**

b) **Is life-threatening**

The term “life-threatening” in the definition of “serious” refers to an event in which the participant was at risk of death at the time of the event. It does not refer to an event, which hypothetically might have caused death, if it were more severe.

c) **Requires inpatient hospitalization or prolongation of existing hospitalization**

In general, hospitalization signifies that the participant has been detained (usually involving at least an overnight stay) at the hospital or emergency ward for observation and/or treatment that would not have been appropriate in the physician’s office or outpatient setting. Complications that occur during hospitalization are AEs. If a complication prolongs hospitalization or fulfills any other serious criteria, the event is serious. When in doubt as to whether “hospitalization” occurred or was necessary, the AE should be considered serious.

Hospitalization for elective treatment of a pre-existing condition that did not worsen from baseline is not considered an AE.

d) **Results in persistent disability/incapacity**

- The term disability means a substantial disruption of a person’s ability to conduct normal life functions.
- This definition is not intended to include experiences of relatively minor medical significance such as uncomplicated headache, nausea, vomiting, diarrhea, influenza, and accidental trauma (eg, sprained ankle) which may interfere with or prevent everyday life functions but do not constitute a substantial disruption.

e) **Is a congenital anomaly/birth defect**

f) **Other situations:**

- Medical or scientific judgment should be exercised in deciding whether SAE reporting is appropriate in other situations such as important medical events that may not be immediately life-threatening or result in death or hospitalization but may jeopardize the participant or may require medical or surgical intervention to prevent one of the other outcomes listed in the above definition. These events should usually be considered serious.

- Examples of such events include invasive or malignant cancers, intensive treatment in an emergency room or at home for allergic bronchospasm, blood dyscrasias or convulsions that do not result in hospitalization, or development of drug dependency or drug abuse.

RECORDING AND FOLLOW-UP OF AE AND/OR SAE

AE and SAE recording

- When an AE/SAE occurs, it is the responsibility of the Investigator to review all documentation (eg, hospital progress notes, laboratory reports, and diagnostics reports) related to the event.
- The Investigator will then record all relevant AE/SAE information in the CRF.
- It is **not** acceptable for the Investigator to send photocopies of the participant's medical records to the Sponsor's representative in lieu of completion of the AE/SAE CRF page.
- There may be instances when copies of medical records for certain cases are requested by the Sponsor's representative. In this case, all participant identifiers, with the exception of the participant number, will be redacted on the copies of the medical records before submission to the Sponsor's representative.
- The Investigator will attempt to establish a diagnosis of the event based on signs, symptoms, and/or other clinical information. Whenever possible, the diagnosis (not the individual signs/symptoms) will be documented as the AE/SAE.

Assessment of intensity

The Investigator will make an assessment of intensity for each AE and SAE reported during the study and assign it to 1 of the following categories:

- Mild: An event that is easily tolerated by the participant, causing minimal discomfort and not interfering with everyday activities.
- Moderate: An event that causes sufficient discomfort and interferes with normal everyday activities.
- Severe: An event that prevents normal everyday activities. An AE that is assessed as severe should not be confused with a SAE. Severe is a category utilized for rating the intensity of an event; and both AEs and SAEs can be assessed as severe.

  An event is defined as "serious" when it meets at least 1 of the predefined outcomes as described in the definition of an SAE, NOT when it is rated as severe.

Assessment of causality

- The Investigator is obligated to assess the relationship between study intervention and each occurrence of each AE/SAE.
- A "reasonable possibility" of a relationship conveys that there are facts, evidence, and/or arguments to suggest a causal relationship, rather than that a relationship cannot be ruled out.

- The Investigator will use clinical judgment to determine whether or not the relationship is causal.
- Alternative causes, such as underlying disease(s), concomitant therapy, and other risk factors, as well as the temporal relationship of the event to study intervention administration will be considered and investigated.
- The Investigator will also consult the IB and/or Product Information, for marketed products, in his/her assessment.
- For each AE/SAE, the Investigator **must** document in the medical notes that he/she has reviewed the AE/SAE and has provided an assessment of causality.
- There may be situations in which an SAE has occurred and the Investigator has minimal information to include in the initial report to the Sponsor's representative. However, **it is very important that the Investigator always make an assessment of causality for every event before the initial transmission of the SAE data to the Sponsor's representative.**
- The Investigator may change his/her opinion of causality in light of follow-up information and send a SAE follow-up report with the updated causality assessment.
- The causality assessment is one of the criteria used when determining regulatory reporting requirements.

Follow-up of AEs and SAEs

- The Investigator is obligated to perform or arrange for the conduct of supplemental measurements and/or evaluations as medically indicated or as requested by the Sponsor's representative to elucidate the nature and/or causality of the AE or SAE as fully as possible. This may include additional laboratory tests or investigations, histopathological examinations, or consultation with other health care professionals.
- If a participant dies during participation in the study or during a recognized follow-up period, the Investigator will provide the Sponsor's representative with a copy of any post-mortem findings including histopathology.
- New or updated information will be recorded in the originally completed CRF.
- The Investigator will submit any updated SAE data to the Sponsor within 24 hours of receipt of the information.

REPORTING OF SAES

SAE reporting to the Sponsor via an electronic data collection tool

- The primary mechanism for reporting an SAE to the Sponsor's representative will be the electronic data collection tool.
- If the electronic system is unavailable, then the site will use the paper SAE data collection tool (see next section) in order to report the event within 24 hours.
- The site will enter the SAE data into the electronic system as soon as it becomes available.

- After the study is completed at a given site, the electronic data collection tool will be taken off-line to prevent the entry of new data or changes to existing data.
- If a site receives a report of a new SAE from a study participant or receives updated data on a previously reported SAE after the electronic data collection tool has been taken off-line, then the site can report this information on a paper SAE form (see next section) or to the Sponsor's representative by telephone.
- Contacts for SAE reporting can be found in the protocol.

SAE reporting to the Sponsor's representative via paper CRF

- Facsimile transmission of the SAE paper CRF is the preferred method to transmit this information to the Sponsor's representative.
- In rare circumstances and in the absence of facsimile equipment, notification by telephone is acceptable with a copy of the SAE data collection tool sent by overnight mail or courier service.
- Initial notification via telephone does not replace the need for the Investigator to complete and sign the SAE CRF pages within the designated reporting time frames.
- Contacts for SAE reporting can be found in the protocol.

10.4 APPENDIX 4: CONTRACEPTIVE GUIDANCE AND COLLECTION OF PREGNANCY INFORMATION

DEFINITIONS:

Woman of childbearing potential (WOCBP)

A woman is considered fertile following menarche and until becoming post-menopausal unless permanently sterile (see below).

If fertility is unclear (eg, amenorrhea in adolescents or athletes) and a menstrual cycle cannot be confirmed before first dose of study intervention, additional evaluation should be considered.

Women in the following categories are not considered WOCBP

1. Premenarchal
2. Premenopausal female with 1 of the following:
   - Documented hysterectomy
   - Documented bilateral salpingectomy
   - Documented bilateral oophorectomy

   For individuals with permanent infertility due to an alternate medical cause other than the above, (eg, mullerian agenesis, androgen insensitivity), Investigator discretion should be applied to determining study entry.

Note: Documentation can come from the site personnel's: review of the participant's medical records, medical examination, or medical history interview.

3. Postmenopausal female
    - A postmenopausal state is defined as no menses for 12 consecutive months without an alternative medical cause.
    - Females on hormone replacement therapy (HRT) and whose menopausal status is in doubt will be required to use one of the non-estrogen hormonal highly effective contraception methods if they wish to continue their HRT during the study.

CONTRACEPTION GUIDANCE

Female participants of childbearing potential are eligible to participate if they agree to use a highly effective method of contraception consistently and correctly as described below.

Contraceptives[a] allowed during the study include:

Highly effective methods[b] that have low user dependency
Failure rate of <1% per year when used consistently and correctly.

- Implantable progestogen-only hormone contraception associated with inhibition of ovulation[b]
    - Intrauterine device (IUD)
    - Intrauterine hormone-releasing system (IUS)[b]
- Bilateral tubal occlusion

Vasectomized partner

(Vasectomized partner is a highly effective contraceptive method provided that the partner is the sole sexual partner of the WOCBP and the absence of sperm has been confirmed. If not, an additional highly effective method of contraception should be used. Spermatogenesis cycle is approximately 90 days.)

Highly effective methods[b] that are user dependent
Failure rate of <1% per year when used consistently and correctly.

- Combined (estrogen- and progestogen-containing) hormonal contraception associated with inhibition of ovulation[c]
    - oral
    - intravaginal
    - transdermal
    - injectable
- Progestogen-only hormone contraception associated with inhibition of ovulation[c]
    - oral
    - injectable

Sexual abstinence

Sexual abstinence is considered a highly effective method only if defined as refraining from heterosexual intercourse during the entire period of risk associated with the study intervention. The reliability of sexual abstinence needs to be evaluated in relation to the duration of the study and the preferred and usual lifestyle of the participant.)

Acceptable methods[d]

- Progestogen-only oral hormonal contraception where inhibition of ovulation is not the primary mode of action
- Male or female condom with or without spermicide[e]
- Cervical cap, diaphragm, or sponge with spermicide
- A combination of male condom with either cervical cap, diaphragm, or sponge with spermicide (double-barrier methods)[c]

a Contraceptive use by men or women should be consistent with local regulations regarding the use of contraceptive methods for those participating in clinical studies.

b Failure rate of <1% per year when used consistently and correctly. Typical use failure rates differ from those when used consistently and correctly.

c If locally required, in accordance with Clinical Trial Facilitation Group (CTFG) guidelines, acceptable contraceptive methods are limited to those which inhibit ovulation as the primary mode of action.

d Considered effective, but not highly effective - failure rate of ≥1% per year. Periodic abstinence (calendar, symptothermal, post-ovulation methods), withdrawal (coitus interruptus), spermicides only, and lactational amenorrhoea method (LAM) are not acceptable methods of contraception.

e Male condom and female condom should not be used together (due to risk of failure with friction).

WOCBP: woman of childbearing potential

COLLECTION OF PREGNANCY INFORMATION:

Male participants with partners who become pregnant

- The Investigator will attempt to collect pregnancy information on any male participant's female partner who becomes pregnant while the male participant is in this study. This applies only to male participants who receive dupilumab.
- After obtaining the necessary signed informed consent from the pregnant female partner directly, the Investigator will record pregnancy information on the appropriate form and submit it to the Sponsor within 24 hours of learning of the partner's pregnancy. The female partner will also be followed to determine the outcome of the pregnancy. Information on the status of the mother and child will be forwarded to the Sponsor. Generally, the follow-up will be no longer than 6 to 8 weeks following the estimated delivery date. Any termination of the pregnancy will be reported regardless of fetal status (presence or absence of anomalies) or indication for the procedure.

Female participants who become pregnant

- The Investigator will collect pregnancy information on any female participant who becomes pregnant while participating in this study. Information will be recorded on the appropriate form and submitted to the Sponsor within 24 hours of learning of a participant's pregnancy. The participant will be followed to determine the outcome of the pregnancy. The Investigator will collect follow-up information on the participant and the neonate and the information will be forwarded to the Sponsor. Generally, follow-up will not be required for longer than 6 to 8 weeks beyond the estimated delivery date. Any termination of pregnancy will be reported, regardless of fetal status (presence or absence of anomalies) or indication for the procedure.
- Any pregnancy complication or elective termination of a pregnancy will be reported as an AE or SAE. A spontaneous abortion is always considered to be an SAE and will be reported as such. Any post-study pregnancy related SAE considered reasonably related to the study intervention by the Investigator will be reported to the Sponsor as described in Section 8.3.4 of the protocol. While the Investigator is not obligated to actively seek this information in former study participants, he or she may learn of an SAE through spontaneous reporting.
- Any female participant who becomes pregnant while participating in the study will discontinue study intervention.

10.5 APPENDIX 5: GENETICS

Use/Analysis of DNA and RNA

- Genetic variation may impact a participant's response to study intervention, susceptibility to, and severity and progression of disease. Variable response to study intervention may be due to genetic determinants that impact drug absorption, distribution, metabolism, and excretion; mechanism of action of the drug; safety, disease etiology; and/or molecular subtype of the disease being treated. Therefore, where local regulations and IRB/IEC allow, a blood sample will be collected for DNA analysis from consenting participants.
- DNA and RNA samples will be used for research related to dupilumab or PN and related diseases. They may also be used to develop tests/assays including diagnostic tests related to dupilumab or related drugs and atopic/allergic diseases. Genetic research may consist of the analysis of one or more candidate genes or the analysis of genetic markers throughout the genome including whole-exome sequencing, whole-genome sequencing, and DNA copy number variation. Transcriptome sequencing (or other methods for quantitating RNA expression) may also be performed.
- The samples may be analyzed as part of a multi-study assessment of genetic factors involved in the response to dupilumab or study interventions of this class to understand study disease or related conditions.
- The results of genetic analyses will not be reported in the CSR.
- The Sponsor will store the DNA samples in a secure storage space with adequate measures to protect confidentiality.
- The samples will be retained no longer than 15 years or other period as per local requirements.

10.6 APPENDIX 6: LIVER AND OTHER SAFETY: SUGGESTED ACTIONS AND FOLLOW-UP ASSESSMENTS

INCREASE IN ALT

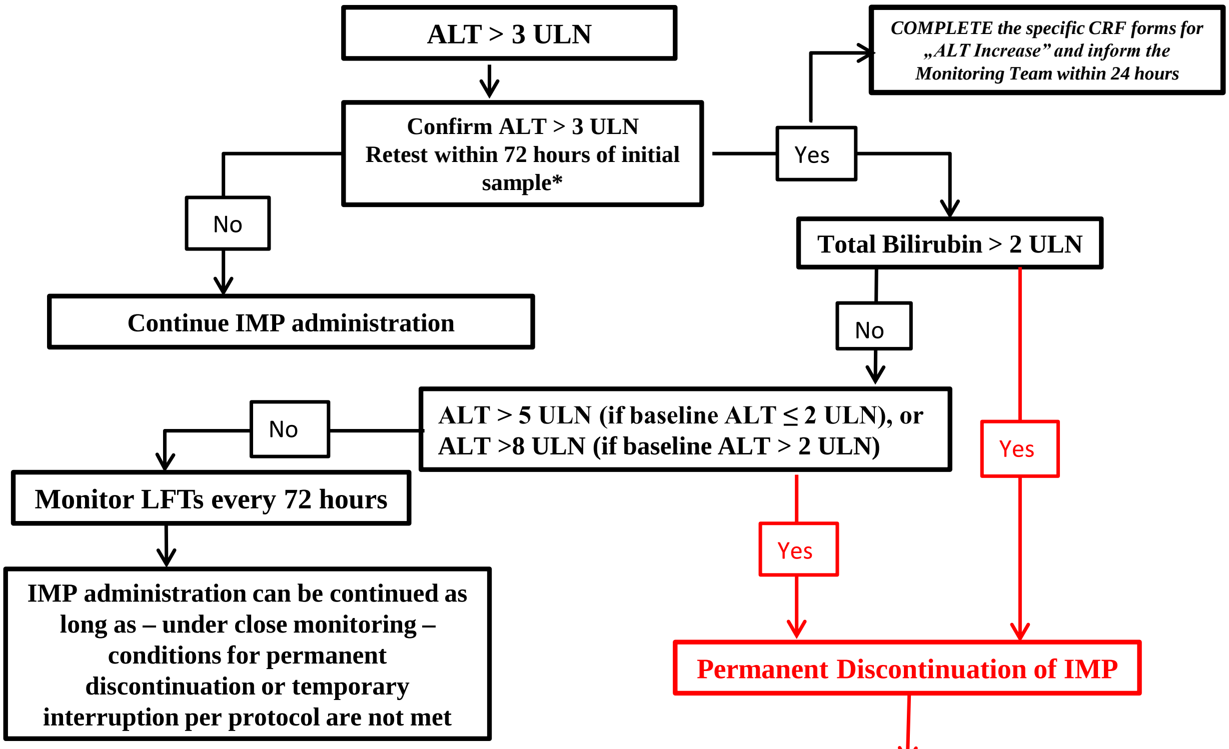

In ANY CASE, FOLLOW the instructions listed in the box below:

1. **INFORM** the Site Monitor who will forward the information to the Study Manager
2. **INVESTIGATE** specifically for malaise with or without loss of consciousness, dizziness, and/or hypotension and/or episode of arrhythmia in the previous 72 hours; rule out muscular injury
3. **PERFORM** the following tests:
   - LFTs: AST, ALT, alkaline phosphatase, total and conjugated bilirubin and prothrombin time / INR
   - CPK, serum creatinine, complete blood count
   - Anti-HAV IgM, anti-HBc IgM (HBV-DNA if clinically indicated), anti-HCV and HCV RNA, anti-CMV IgM and anti-HEV IgM antibodies
   - Depending on the clinical context, check for recent infection with EBV, herpes viruses, and toxoplasma
   - Hepatobiliary ultrasonography (or other imaging investigations if needed)
4. **CONSIDER** Auto-antibodies: antinuclear, anti-DNA, anti-smooth muscle, anti-LKM
5. **CONSIDER** consulting with hepatologist
6. **CONSIDER** patient hospitalisation if INR>2 (or PT<50%) and/or central nervous system disburbances suggesting hepatic encephalopathy
7. **MONITOR LFTs after discontinuation of IMP:**
   - As *closely as possible* (or **every 48 hours)** until stabilization, then every 2 weeks until return to normal/baseline or clinical resolution.
8. **FREEZE** serum sample (5ml x 2)
9. **In case of SUSPICION of GILBERT Syndrome,** a DNA diagnostic test should be done

*If unable to retest in 72 hours, use original lab results to decide on further reporting/monitoring/discontinuation.

Note:

"Baseline" refers to ALT sampled at baseline visit; or if baseline value unavailable, to the latest ALT sampled before the baseline visit. The algorithm does not apply to the instances of increase in ALT during screening.

See Section 10.3 for guidance on safety reporting.

Normalization is defined as ≤ULN or baseline value, if baseline value is >ULN.

INCREASE IN SERUM CREATININE in patients with normal baseline (creatininemia between 45 µmol/L and 84 µmol/L)

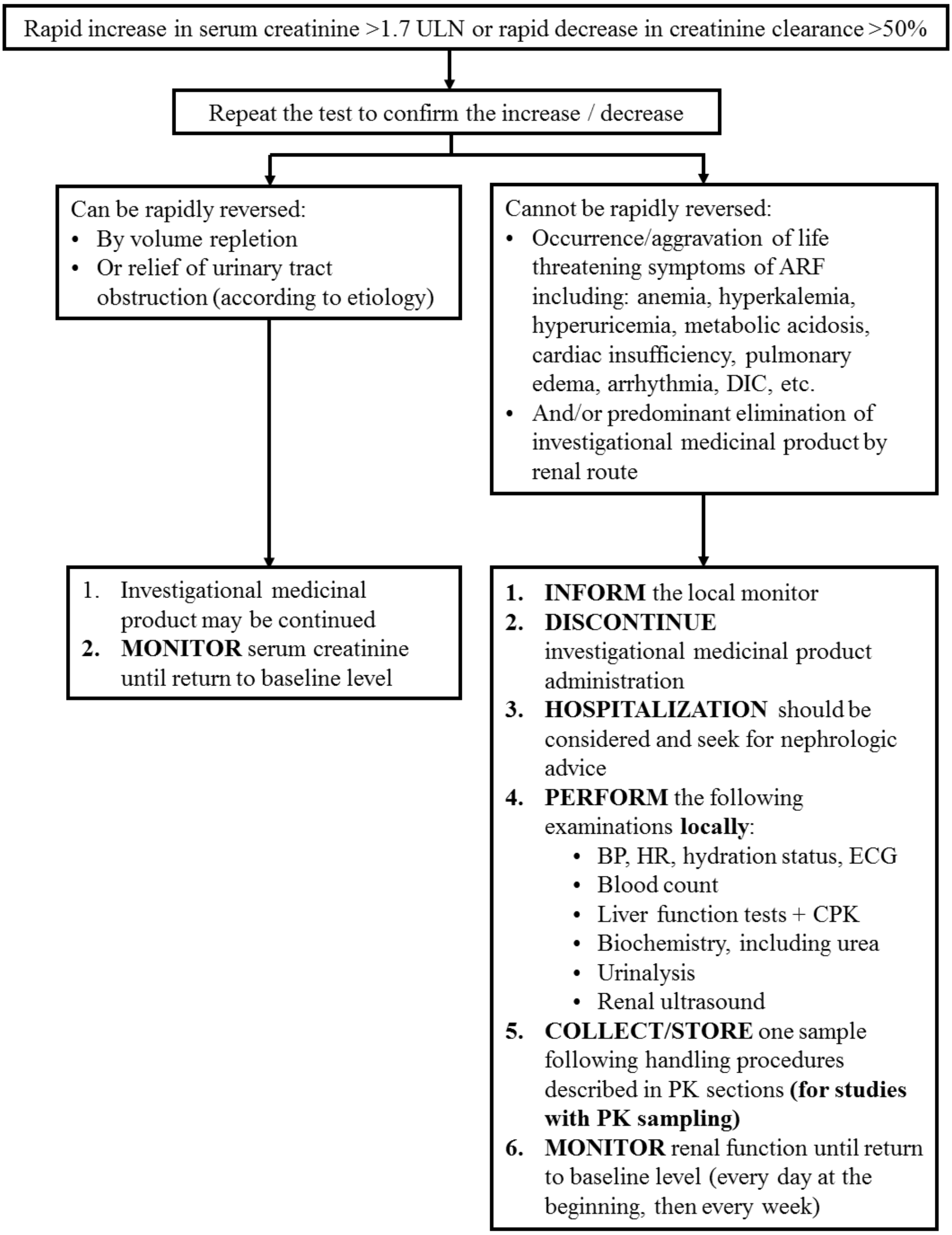

Increase in serum creatinine is to be recorded as an AE only if at least 1 of the criteria listed in the general guidelines for reporting AEs in Section 10.3 is met.

INCREASE IN CPK OF NON-CARDIAC ORIGIN AND NOT RELATED TO INTENSIVE PHYSICAL ACTIVITY

If Increase in CPK > 10 ULN

↓

Repeat immediately locally the count.
If confirmed, inform the local monitor and

↓

INVESTIGATE for the origin:
- **INTERVIEW** the patient about a recent intensive muscular effort, trauma, convulsions, electrical injury, or stress to the skeletal muscle, multiple intramuscular injections, recent surgery, concomitant medications, consumption of alcohol, morphine, cocaine…
- **PERFORM locally**:
  - ECG, CK-MB , Troponin if not previously done
  - CK-MM
  - Creatinine
  - Iono (K+)
  - Transaminases + Total and conjugated bilirubin
  - And any other parameters considered as relevant per medical judgement (e.g. Ca^{2+}, Myoglobin)
- **COLLECT/STORE** one sample following handling procedures described in PK sections **(for studies with PK sampling)**
- **SEARCH** for the cause

↓

1. Hospitalization should be considered depending on clinical context and profile of the patient
2. Consider to discontinue investigational medicinal product administration
3. Monitor biological parameters as appropriate within the next days/weeks/months until return to baseline

Increase in CPK is to be recorded as an AE only if at least 1 of the criteria in the general guidelines for reporting AEs in Section 10.3 is met.

10.7 APPENDIX 7: CLINICIANS-REPORTED OUTCOMES AND PATIENT-REPORTED OUTCOMES

10.7.1 Worst itch numeric rating scale (WI-NRS)

WWU MÜNSTER

UKM Universitätsklinikum Münster

[redacted]

GB ZD . 01/2019

UKM: AöR, Prof. Dr. Martin Schulze Schwienhorst (Aufsichtsratsvorsitzender),
Univ.-Prof. Dr. med. Dr. phil. Robert Nitsch (Vorstandsvorsitzender, Ärztlicher Direktor),
Dr. rer. pol. Christoph Hoppenheit (stellv. Vorstandsvorsitzender, Kaufmännischer Direktor),
Univ.-Prof. Dr. med. Mathias Herrmann (Dekan), Thomas van den Hooven (Pflegedirektor),
Univ.-Prof. Dr. med. Claudia Rössig (stellv. Ärztliche Direktorin)

KTQ-Zertifikat

10.7.2 Investigator’s global assessment of prurigo nodularis (IGA PN)

[redacted]

10.7.3 Prurigo activity score (PAS)

[redacted]

[redacted]

10.7.4 Dermatology life quality index (DLQI)

[redacted]

10.7.5 Hospital anxiety and depression scale (HADS)

[redacted]

10.7.6 EQ-5D-5L

[redacted]

VV-CLIN-0649751 2.0

10.7.7 Pain numeric rating scale

Worst Skin Pain Numerical Rating Scale (Generic)

How would you rate your skin pain at its **worst** in the past 24 hours?

0	1	2	3	4	5	6	7	8	6	10
No pain										Worst pain possible

10.7.8 Sleep numeric rating scale

Sleep Quality Numerical Rating Scale (Generic)

Instructions:

Please complete the following questions upon awakening for the day.

Select the number that best describes the quality of your sleep last night.

0	1	2	3	4	5	6	7	8	9	10
Worst possible sleep										Best possible sleep

10.7.9 Patient Global Impression of Change of disease (PGIC)

Patient Global Impression of Change (PGIC) of PN Disease

Please choose the response below that best describes the overall change in your prurigo nodularis since you started taking the study medication.

☐ Very much better

☐ Moderately better

☐ A little better

☐ No change

☐ A little worse

☐ Moderately worse

☐ Very much worse

10.7.10 Patient Global Impression of Severity (PGIS)

Patient Global Impression of Severity (PGIS) of PN Disease

Please choose the response below that best describes the severity of your prurigo nodularis over the past week.

☐ None

☐ Mild

☐ Moderate

☐ Severe

10.7.11 Missed school/work days

Patients with Prurigo Nodularis *(PN)*

Missed School/Work days
BASELINE Visit

Site Number: __________ Patient Study Number: ___________________________

Visit Date (dd/mmm/yyyy): ______/______/______Time of Completion: ___:_____am/pm

Employment status

1. Have you been working?

   □ Yes – I am working full time (≥4 days/week)
   □ Yes – I am working part time (<4 days/week)
   □ No – I am not working (unemployed, retired)

For those working (full time or part time):

2. How many days have you missed from work because of your Prurigo Nodularis in the last 4 weeks prior to the visit today?

   Please do not include time you missed from work for taking part in this research.

   Specify: __ __ __ Days

Student status

1. Have you been in school?

   □ Yes – I am in school full time (≥4 days/week)
   □ Yes – I am in school part time (<4 days/week)
   □ No – I am not in school

For those in school (full time or part time):

2. How many days have you missed from school because of your Prurigo Nodularis in the last 4 weeks prior to the visit today?

   Please do not include time you missed from school for taking part in this research.

   Specify: __ __ __ Days

NOTES:

Please make sure the instructions are read to the patient BEFORE they start the questionnaire.

Assessment periods (i.e., time interval from the date of last assessment through the current date) must be continuous. Leave no gaps. Cumulatively, these periods must cover the entire study duration.

Avoid duplicate reporting. If the patient missed school/work because of his/her PN on the day of

assessment, this day should be recorded only once.

Record only days on which patient's PN prevented him/her to go to school/work. Otherwise, do not record the days on which the patient missed school/work only to participate in study visits.

Print Name (First/Last) of Investigator Completing Assessment	Signature	Date

R668-XXX-Missed School/work Days Version 1.0 BASELINE; *date*

Page 1 of 1

Patients with *Prurigo Nodularis (PN)*

Missed School/work days

POST Baseline Visits

Site Number: ______________Patient Study Number: ______________

Visit Date (dd/mmm/yyyy): ____/_____/_____Time of Completion: ___:____am/pm

Employment status

1. Have you been working since your last assessment?

   □ Yes – I am working full time (≥4 days/week)
   □ Yes – I am working part time (<4 days/week)
   □ No – I am not working (unemployed, retired)

For those working (full time or part time):

2. How many days have you missed from work because of your Prurigo Nodularis since the day of your last assessment, including today?

   Please do not include time you missed from work for taking part in this research.

   Specify: __ __ __ Days

Student status

1. Have you been in school since your last assessment?

   □ Yes – I am in school full time (≥4 days/week)
   □ Yes – I am in school part time (<4 days/week)
   □ No – I am not in school

For those in school (full time or part time):

2. How many days have you missed from school because of your Prurigo Nodularis since the day of your last assessment, including today?

   Please do not include time you missed from school for taking part in this research.

   Specify: __ __ __ Days

NOTES:

Please make sure the instructions are read to the patient BEFORE they start the questionnaire.

Assessment periods (i.e., time interval from the date of last assessment through the current date) must be continuous. Leave no gaps. Cumulatively, these periods must cover the entire study duration.

Avoid duplicate reporting. If the patient missed school/work because of his/her PN on the day of assessment, this day should be recorded only once.

Record only days on which patient's PN prevented him/her to go to school/work. Otherwise, do not record the days on which the patient missed school/work only to participate in study visits.

Print Name (First/Last) of Investigator Completing Assessment	Signature	Date

R668-XXX Missed School/work Days Version 2.0; date

Page 1 of 1

10.8 APPENDIX 8: DEFINITION OF ANAPHYLAXIS

"Anaphylaxis is a serious allergic reaction that is rapid in onset and may cause death (31)."

Clinical criteria for diagnosing anaphylaxis

Anaphylaxis is highly likely when any one of the following 3 criteria are fulfilled:

1. Acute onset of an illness (minutes to several hours) with involvement of the skin, mucosal tissue, or both (eg, generalized hives, pruritus or flushing, swollen lips-tongue-uvula)
   AND AT LEAST ONE OF THE FOLLOWING
   a. Respiratory compromise (eg, dyspnea, wheeze-bronchospasm, stridor, reduced PEF, hypoxemia)
   b. Reduced BP or associated symptoms of end-organ dysfunction (eg, hypotonia [collapse], syncope, incontinence)
2. Two or more of the following that occur rapidly after exposure *to a likely allergen for that patient* (minutes to several hours):
   a. Involvement of the skin-mucosal tissue (eg, generalized hives, itch-flush, swollen lips-tongue-uvula)
   b. Respiratory compromise (eg, dyspnea, wheeze-bronchospasm, stridor, reduced PEF, hypoxemia)
   c. Reduced BP or associated symptoms (eg, hypotonia [collapse], syncope, incontinence)
   d. Persistent gastrointestinal symptoms (eg, crampy abdominal pain, vomiting)
3. Reduced BP after exposure to *known allergen for that patient* (minutes to several hours):
   a. Infants and children: low systolic BP (age specific) or greater than 30% decrease in systolic BP*
   b. Adults: systolic BP of less than 90 mm Hg or greater than 30% decrease from that person's baseline

PEF, Peak expiratory flow; *BP*, blood pressure.
*Low systolic blood pressure for children is defined as less than 70 mm Hg from 1 month to 1 year, less than (70 mm Hg + [2 × age]) from 1 to 10 years, and less than 90 mm Hg from 11 to 17 years.

10.9 APPENDIX 9: LIST OF OPPORTUNISTIC INFECTIONS

- Aspergillosis
- Blastomyces dermatitidis (endemic in the south-eastern and south-central states US, along Mississippi and Ohio Rivers)
- Candidiasis - only systemic or extensive mucosal or cutaneous candidiasis
- Coccidioides immitis (endemic south-western US and Central and South America)
- Cryptococcus
- Cytomegalovirus
- Herpes Simplex (disseminated)
- Herpes Zoster (disseminated; ophthalmic; involvement of 2 or more dermatomes)
- Histoplasmosis (pulmonary or disseminated; most common tropical areas Tennessee-Ohio-Mississippi river basins)
- Listeriosis
- Mycobacterium avium
- Nontuberculosis mycobacteria
- Pneumocystis pneumonia

This list is indicative and not exhaustive.

10.10 APPENDIX 10: COUNTRY-SPECIFIC REQUIREMENTS

Not applicable.

10.11 APPENDIX 11: ABBREVIATIONS

ACE:	angiotensin converting enzyme
AD:	atopic dermatitis
ADA:	antidrug antibody
ADR:	adverse drug reaction
AE:	adverse event
AESI:	adverse event of special interest
ALT:	alanine aminotransferase
ANCOVA:	analysis of covariance
AST:	aspartate aminotransferase
BUN:	blood urea nitrogen
CFR:	Code of Federal Regulations
CIOMS:	Council for International Organizations of Medical Sciences
ClinRO:	clinician-reported outcome
CMH:	Cochran-Mantel-Haenszel
CONSORT:	Consolidated Standards of Reporting Trials
CRF:	case report form
CRSwNP:	chronic rhinosinusitis with nasal polyposis
CSR:	clinical study report
DBP:	diastolic blood pressure
DLQI:	dermatology life quality index
DMC:	data monitoring committee
DNA:	deoxyribonucleic acid
DTP:	direct-to-patient
EADV:	European Academy of Dermatology and Venereology
EASI:	eczema area and severity index
ECG:	electrocardiogram
eCRF:	electronic case report form
ELISA:	enzyme-linked immunosorbent assay
EMA:	European Medicines Agency
EoE:	eosinophilic esophagitis
EOS:	end of study
EOT:	end of treatment
EQ-5D:	Euroqol 5 dimensions
EQ-5D-5L:	Euroqol 5 dimensions 5 levels
EU:	European Union
FDA:	Food and Drug Administration
GCP:	Good Clinical Practice
HADS:	hospital anxiety and depression scale
HBc Ab:	hepatitis B core antibody

HBs Ab:	hepatitis B surface antibody
HBs Ag:	hepatitis B surface antigen
HBV:	hepatitis B virus
hCG:	human chorionic gonadotropin
HCV:	hepatitis C virus
HCV Ab:	hepatitis C virus antibody
HIV:	human immunodeficiency virus
HRQoL:	health-related quality of life
HRT:	hormone replacement therapy
IB:	investigator's brochure
ICF:	informed consent form
ICH:	International Council for Harmonisation
IEC:	Independent Ethics Committee
Ig:	immunoglobulin
IGA:	investigator's global assessment
IGA PN:	investigator's global assessment for prurigo nodularis
IGA PN-A:	IGA PN-Activity
IGA PN-S:	IGA PN-Stage
IL:	interleukin
IMP:	investigational medicinal product
IRB:	Institutional Review Board
IRT:	interactive response technology
ITT:	intent to treat
IVRS:	interactive voice response system
IWRS:	interactive web response system
LS:	least squares
NIMP:	noninvestigational medicinal product
NRS:	numeric rating scale
PAS:	prurigo activity score
PD:	pharmacodynamic
PGA:	Physician Global Assessment
PGIC:	Patient Global Impression of Change
PGIS:	Patient Global Impression of Severity
PK:	pharmacokinetic
PN:	prurigo nodularis
PRO:	patient reported outcome
PT:	preferred term
Q2W:	every 2 weeks
QTcF:	QT interval corrected using Fridericia's formula
RBC:	red blood cells
RNA:	ribonucleic acid
SAE:	serious adverse event
SAP:	statistical analysis plan
SBP:	systolic blood pressure
SC:	subcutaneous
SCORAD:	scoring atopic dermatitis

SGOT:	serum glutamic-oxaloacetic transaminase
SGPT:	serum glutamic-pyruvic transaminase
SNRI:	serotonin and norepinephrin reuptake inhibitor
SoA:	schedule of activities
SOC:	system organ class
SSRI:	selective serotonin reuptake inhibitor
SUSAR:	suspected unexpected serious adverse reaction
TB:	tuberculosis
TCI:	topical calcineurin inhibitors
TCS:	topical corticosteroids
TEAE:	treatment-emergent adverse event
ULN:	upper limit of normal
US:	United States
UVB:	ultraviolet B
VAS:	visual analog scale
WBC:	white blood cells
WI-NRS:	worst-itch numeric rating scale
WOCBP:	woman of childbearing potential
WOCF:	worst observation carried forward

11 REFERENCES

1. Zeidler C, Athanasios T, Pereira M, Stander H, Yosipovitch G, Stander S. Chronic prurigo of nodular type: A Review. Acta Derm Venereol 2018;98(2):173-9.

2. Iking A, Grundmann S, Chatzigeorgakidis E, Phan NQ, Klein D, Ständer S. Prurigo as a symptom of atopic and non-atopic diseases: aetiological survey in a consecutive cohort of 108 patients. J Eur Acad Dermatol Venereol. 2013;27(5):550-7.

3. Mollanazar NK, Elgash M, Weaver L, Valdes-Rodriguez R, Hsu S. Reduced itch associated with dupilumab treatment in 4 patients with prurigo nodularis. JAMA Dermatol. 2019;155(1):121-2.

4. Beck KM, Yang EJ, Sekhon S, Bhutani T, Liao W. Dupilumab treatment for generalized prurigo nodularis. JAMA Dermatol. 2019;155(1):118-20.

5. Rambhia PH, Levitt JO. Recalcitrant prurigo nodularis treated successfully with dupilumab. JAAD Case Rep. 2019;5(5):471-3.

6. Calugareanu A, Jachiet M, Lepelletier C, De Masson A, Rybojad M, Bagot M, et al. Dramatic improvement of generalized prurigo nodularis with dupilumab. J Eur Acad Dermatol Venereol. 2019; 33(8):e303-4.

7. Zhai LL, Savage KT, Qiu CC, Jin A, Valdes-Rodriguez R, Mollanazar NK. Chronic Pruritus Responding to Dupilumab-A Case Series. Medicines (Basel). 2019;6(3):E72.

8. Weigelt N, Metze D, Stander S. Prurigo nodularis: systematic analysis of 58 histological criteria in 136 patients. J Cutan Pathol. 2010;37(5):578-86

9. Yang TB, Kim BS. Pruritus in allergy and immunology. J Allergy Clin Immunol. 2019;144(2):353-60.

10. Pereira MP, Steinke S, Zeidler C, Forner C, Riepe C, Augustin M, et al. European academy of dermatology and venereology European prurigo project: expert consensus on the definition, classification and terminology of chronic prurigo. J Eur Acad Dermatol Venereol. 2018;32(7):1059-65.

11. Zeidler C, Ständer S. The pathogenesis of Prurigo nodularis--'Super-Itch' in exploration. Eur J Pain. 2016;20(1):37-40.

12. Fukushi S, Yamasaki K, Aiba S. Nuclear localization of activated STAT6 and STAT3 in epidermis of prurigo nodularis. Br J Dermatol. 2011;165(5):990-6.

13. Tokura Y, Yagi H, Hanaoka K, Ito T, Towyama K, Sachi Y, et al. Subacute and chronic prurigo effectively treated with recombination interferon-gamma: implications for

participation of Th2 cells in the pathogenesis of prurigo. Acta Derm Venereol. 1997;77(3):231-4.

14. Oetjen LK, Mack MR, Feng J, Whelan TM, Niu H, Guo CJ, et al. Sensory neurons co-opt classical immune signaling pathways to mediate chronic itch. Cell. 2017;171(1):217-28.

15. Boozalis E, Tang O, Patel S, Semenov YR, Pereira MP, Stander S, et al. Ethnic differences and comorbidities of 909 prurigo nodularis patients. J Am Acad Dermatol. 2018;79(4):714-9.

16. Steinke S, Zeidler C, Riepe C, Bruland P, Soto-Rey I, Storck M, et al. Humanistic burden of chronic pruritus in patients with inflammatory dermatoses: Results of the European Academy of Dermatology and Venereology Network on Assessment of Severity and Burden of Pruritus (PruNet) cross-sectional trial. J Am Acad Dermatol. 2018;79(3):457-63.

17. Tanaka M, Aiba S, Matsumura N, Aoyama H, Tagami H. Prurigo nodularis consists of two distinct forms: early-onset atopic and late-onset non-atopic. Dermatology. 1995;190(4):269-76.

18. Kowalski EH, Kneiber D, Valdebran M, Patel U, Amber KT. Treatment-resistant prurigo nodularis: challenges and solutions. Clin Cosmet Investig Dermatol. 2019;12:163-72.

19. Novartis Laboratories. NEORAL Soft Gelatin Capsules (cyclosporine capsules, USP) MODIFIED NEORAL Oral Solution (cyclosporine oral solution, USP) MODIFIED. Prescribing information. 2009.

20. Ständer S, Kwon P, Hirman J, Perlman AJ, Weisshaar E, Metz M, et al. Serlopitant reduced pruritus in patients with prurigo nodularis in a phase 2, randomized, placebo-controlled trial. J Am Acad Dermatol. 2019;80(5):1395-402.

21. Verweyen E, Ständer S, Kreitz K, Höben I, Osada N, Gernart M, et al. Validation of a Comprehensive Set of Pruritus Assessment Instruments: The Chronic Pruritus Tools Questionnaire PRURITOOLS. Acta Derm Venereol. 2019;99(7):657-63.

22. Reich A, Bożek A, Janiszewska K, Szepietowski JC. 12-Item Pruritus Severity Scale: Development and Validation of New Itch Severity Questionnaire. Biomed Res Int. 2017;2017:3896423.

23. Reich A, Riepe C, Anastasiadou Z, Mędrek K, Augustin M, Szepietowski JC, et al. Itch Assessment with Visual Analogue Scale and Numerical Rating Scale: Determination of Minimal Clinically Important Difference in Chronic Itch. Acta Derm Venereol. 2016;96(7):978-80.

24. Phan NQ, Blome C, Fritz F, Gerss J, Reich A, Ebata T, et al. Assessment of pruritus intensity: prospective study on validity and reliability of the visual analogue scale, numerical rating scale and verbal rating scale in 471 patients with chronic pruritus. Acta Derm Venereol. 2012;92(5):502-7.

25. Chernyshov PV. The evolution of quality of life assessment and use in dermatology. Dermatology. 2019;235(3):167-74.

26. Pölking J, Zeidler C, Schedel F, Osada N, Augustin M, Metze D, et al. Prurigo Activity Score (PAS): validity and reliability of a new instrument to monitor chronic prurigo. J Eur Acad Dermatol Venereol. 2018;32(10):1754-60.

27. Finlay AY, Khan GK. Dermatology Life Quality Index (DLQI)--a simple practical measure for routine clinical use. Clin Exp Dermatol. 1994;19(3):210-6.

28. Zigmond AS, Snaith RP. The hospital anxiety and depression scale. Acta Psychiatr Scand. 1983;67(6):361-70.

29. Herrmann C. International experiences with the Hospital Anxiety and Depression Scale--a review of validation data and clinical results. J Psychosom Res. 1997;42(1):17-41.

30. Herdman M, Gudex C, Lloyd A, Janssen M, Kind P, Parkin D, et al. Development and preliminary testing of the new five-level version of EQ-5D (EQ-5D-5L). Qual Life Res. 2011;20(10):1727-36.

31. Sampson HA, Munoz-Furlong A, Campbell RL, Adkinson NF, Bock SA, Branum A, et al. Second symposium on the definition and management of anaphylaxis: Summary report - Second National Institute of Allergy and Infectious Disease/Food Allergy and Anaphylaxis Network symposium. J Allergy Clin Immunol. 2006;117(2):391-7.

Signature Page for VV-CLIN-0549725 v2.0
efc16460-16-1-1-protocol

Approve & eSign	[redacted]

Approve & eSign	[redacted]

STATISTICAL ANALYSIS PLAN

**Protocol title:**	**A randomized, double blind, placebo-controlled, multi-center, parallel group study to evaluate the efficacy and safety of dupilumab in patients with prurigo nodularis who are inadequately controlled on topical prescription therapies or when those therapies are not advisable**
**Protocol number:**	**EFC16460**
**Compound number (INN/Trademark):**	**SAR231893/REGN668 dupilumab/Dupixent**
**Study phase:**	**Phase 3**
**Short title:**	**Study of dupilumab for the treatment of patients with prurigo nodularis, inadequately controlled on topical prescription therapies or when those therapies are not advisable**<br>**LIBERTY-PN PRIME 2**
**Statistician:**	[redacted]
**Statistical project leader:**	
**Date of issue:**	**04-Feb-2021**

Regulatory agency identifier number(s):

IND:	IND107969
EudraCT:	2019-003801-90
NCT:	NCT04202679
WHO:	U1111-1241-8174
Other:	Not applicable

Total number of pages: 62

Any and all information presented in this document shall be treated as confidential and shall remain the exclusive property of Sanofi (or any of its affiliated companies). The use of such confidential information must be restricted to the recipient for the agreed purpose and must not be disclosed, published or otherwise communicated to any unauthorized persons, for any reason, in any form whatsoever without the prior written consent of Sanofi (or the concerned affiliated company); 'affiliated company' means any corporation, partnership or other entity which at the date of communication or afterwards (i) controls directly or indirectly Sanofi, (ii) is directly or indirectly controlled by Sanofi, with 'control' meaning direct or indirect ownership of more than 50% of the capital stock or the voting rights in such corporation, partnership or other entity

TABLE OF CONTENTS

STATISTICAL ANALYSIS PLAN 1

TABLE OF CONTENTS 2

LIST OF TABLES 4

VERSION HISTORY 5

1 INTRODUCTION 6

1.1 STUDY DESIGN 6

1.2 OBJECTIVE AND ENDPOINTS 6

1.2.1 Estimands 9

2 SAMPLE SIZE DETERMINATION 13

3 ANALYSIS POPULATIONS 14

4 STATISTICAL ANALYSES 16

4.1 GENERAL CONSIDERATIONS 16

4.2 PARTICIPANT DISPOSITIONS 16

4.3 PRIMARY ENDPOINT(S) ANALYSIS 17

4.3.1 Definition of endpoint(s) 17

4.3.2 Main analytical approach 19

4.3.3 Sensitivity analysis 20

4.3.4 Supplementary analyses 20

4.3.5 Subgroup analyses 20

4.4 SECONDARY ENDPOINT(S) ANALYSIS 21

4.4.1 Key/Confirmatory secondary endpoint(s) 21

4.4.1.1 Definition of endpoint(s) 21

4.4.1.2 Main analytical approach 22

4.4.2 Supportive secondary endpoint(s) 22

4.5 TERTIARY/EXPLORATORY ENDPOINT(S) ANALYSIS 24

4.5.1 Definition of endpoint(s) 24

4.5.2 Main analytical approach 27

4.6 MULTIPLICITY ISSUES 27

4.7 SAFETY ANALYSES 28
4.7.1 Extent of exposure 28
4.7.2 Adverse events 29
4.7.3 Additional safety assessments....................34
4.7.3.1 Laboratory variables, vital signs and electrocardiograms (ECGs)....................34
4.8 OTHER ANALYSES....................36
4.8.1 Pharmacokinetic analyses 36
4.8.2 Immunogenicity analyses....................36
4.8.3 Pharmacodynamic/genomics endpoints 39
4.9 INTERIM ANALYSES 40
5 SUPPORTING DOCUMENTATION 41
5.1 APPENDIX 1 LIST OF ABBREVIATIONS 41
5.2 APPENDIX 2 CHANGES TO PROTOCOL-PLANNED ANALYSES 43
5.3 APPENDIX 3 DEMOGRAPHICS AND BASELINE CHARACTERISTICS, PRIOR OR CONCOMITANT MEDICATIONS/PROCEDURES....................44
5.4 APPENDIX 4 DATA HANDLING CONVENTIONS 48
5.5 APPENDIX 5 SAMPLE SAS CODE....................55
5.6 APPENDIX 6 SELECTION CRITERIA FOR AE/MEDICATION GROUPINGS 57
5.7 APPENDIX 7 MEDICATION/PROCEDURE AJUDICATION ALGORITHM 59
6 REFERENCES....................62

LIST OF TABLES

Table 1 - Objectives and endpoints....6
Table 2 - Summary of primary estimand for main endpoints....10
Table 3 - Populations for analyses....14
Table 4 - Selected prohibited medications/procedures and rescue medications that impact on efficacy....18
Table 5 - Sorting of AE tables....30
Table 6 - Analyses of adverse events....31
Table 7 - Selections for AESIs and other AEs of interest....32
Table 8 - Major statistical changes in protocol amendment(s)....43
Table 9 - Time window for eDiary efficacy variables....49
Table 10 - Time window for efficacy variables....51
Table 11 - Time window for safety endpoints....53
Table 12 - Time window for pharmacokinetics/pharmacodynamics variables....54
Table 13 - List of PTs or medications for CMQs/CDGs....57

VERSION HISTORY

This Statistical Analysis Plan (SAP) for study EFC16460 is based on the Protocol Amendment 1 dated 20-May-2020. There are no major changes to the statistical analysis features in this SAP, but the changes to the statistical analysis features from the original protocol to Amendment 1 are described in Appendix 2.

The first participant was randomized on 2020-02-03.

1 INTRODUCTION

1.1 STUDY DESIGN

This study is a multi-center, 24-week treatment, parallel group, double-blind, randomized, placebo-controlled study to evaluate the use of dupilumab in participants with prurigo nodularis (PN) inadequately controlled on topical prescription therapies or when those therapies are not advisable. The study will assess the effect of dupilumab on itch improvement as well as its effect on PN lesions, on participants' health-related quality of life (HRQoL), anxiety and depression, sleep quality, skin pain, and overall health status.

After 2-4 weeks of screening, participants will be centrally randomized using a permuted block randomization schedule via Interactive Voice Response System/Interactive Web Response System (IVRS/IWRS) in a 1:1 randomization ratio to dupilumab 300 mg q2w or matching placebo. Randomization will be stratified by documented history of atopy (atopic or non-atopic), stable use of topical corticosteroids (TCS)/ topical calcineurin inhibitors (TCI) (yes or no), and country/territory code.

A total of approximately 150 participants will be randomized to two treatment arms (75 participants/arm). The number of participants with active mild AD upon study entry will represent up to 10% of the atopic participants. Both the atopic and the non-atopic PN populations will each be capped at 60% of the total enrolled population.

The study duration consists of the following periods:

- Screening period (2-4 weeks)
- Randomized IMP intervention period (24 weeks)
- Follow-up period (12 weeks)

1.2 OBJECTIVE AND ENDPOINTS

Table 1 - Objectives and endpoints

Objectives	Endpoints
**Primary**	
• To demonstrate the efficacy of dupilumab on itch response in participants with PN, inadequately controlled on topical prescription therapies or when those therapies are not advisable.	• Proportion of participants with improvement (reduction) in worst-itch numeric rating scale (WI-NRS) by ≥4 from baseline to Week 12.
**Secondary**	
• To demonstrate the efficacy of dupilumab on additional itch endpoints in participants with PN, inadequately controlled on topical prescription therapies or when those therapies are not advisable.	• Proportion of participants with improvement (reduction) in WI-NRS by ≥4 from baseline to Week 24 **[Key secondary endpoint]**.<br>• Time to onset of effect on pruritus as measured by proportion of participants with an improvement

Objectives	Endpoints
	(reduction) in WI-NRS by ≥4 from baseline during the 24-week treatment period.<br>• Change from baseline in WI-NRS at Week 24.<br>• Change from baseline in WI-NRS at Week 12.<br>• Percent change from baseline in WI-NRS at Week 24.<br>• Percent change from baseline in WI-NRS at Week 12.<br>• Percent change from baseline in WI-NRS at Week 4.<br>• Percent change from baseline in WI-NRS at Week 2.<br>• Percent change from baseline in WI-NRS over time until Week 24.<br>• Proportion of participants with WI-NRS reduction ≥4 at Week 4.<br>• Proportion of participants with WI-NRS reduction ≥4 over time until Week 24[a].<br>• Onset of action in change from baseline in WI-NRS (first p <0.05 difference from placebo in the daily WI-NRS that remains significant at subsequent measurements) until Week 12[b].
• To demonstrate efficacy of dupilumab on skin lesions of PN.	• Proportion of participants with Investigator's Global Assessment 0 or 1 score for PN-Stage (IGA PN-S) at Week 24 **[Key secondary endpoint]**.<br>• Proportion of participants with IGA PN-S 0 or 1 score at Week 12.<br>• Proportion of participants with IGA PN-S 0 or 1 score at Week 8[c].<br>• Proportion of participants with IGA PN-S 0 or 1 score at Week 4[c].<br>• Change from baseline in IGA PN-S score at Week 24.<br>• Change from baseline in IGA PN-S score at Week 12.<br>• Change from baseline in IGA PN-S score at Week 8.<br>• Change from baseline in IGA PN-S score at Week 4.<br>• Proportion of participants with Investigator's Global Assessment 0 or 1 score for PN-Activity (IGA PN-A) at Week 24.<br>• Proportion of participants with IGA PN-A 0 or 1 score at Week 12.<br>• Proportion of participants with IGA PN-A 0 or 1 score at Week 8.<br>• Proportion of participants with IGA PN-A 0 or 1 score at Week 4.
• To demonstrate the improvement in health-related quality of life (HRQoL).	• Change from baseline in HRQoL, as measured by Dermatology Life Quality Index (DLQI) to Week 24.<br>• Change from baseline in HRQoL, as measured by DLQI to Week 12.

Objectives	Endpoints
• To evaluate safety outcome measures.	• Percentage of participants experiencing treatment-emergent adverse events (TEAEs) or serious adverse events (SAEs) from baseline through Week 24.
• To evaluate immunogenicity of dupilumab.	• Incidence of treatment-emergent antidrug antibodies (ADA) against dupilumab over time.
**Tertiary/exploratory**	
• To demonstrate a reduction in the use of rescue medication and systemic immunosuppressant	• Use of high potency or superpotent TCS rescue medication through Week 24<br>• Use of systemic immunosuppressants through Week 24, constituting treatment failure
• To evaluate exploratory outcome measures	• Change from baseline in Hospital Anxiety and Depression Scale (HADS) total score to Week 24.<br>• Change from baseline in EQ5D-5L to Week 24.<br>• Change from baseline in Pain numeric rating scale (NRS) to Week 4, Week 8, Week 12, and Week 24, respectively.<br>• Change from baseline in Sleep NRS to Week 4, Week 8, Week 12, and Week 24, respectively.<br>• Missed school/work days through Week 24.<br>• Incidence of skin-infection TEAEs (excluding herpetic infections) through Week 24.
• To evaluate the efficacy of dupilumab on skin lesions using a modified PAS 5-item questionnaire	• Proportion of participants who achieve ≥75% healed lesions from Prurigo Activity Score (PAS) at Week 4, Week 8, Week 12, and Week 24, respectively.<br>• Change from baseline in exact number of lesions in representative area (as determined from PAS) at Week 4, Week 8, Week 12, and Week 24, respectively.
• To evaluate the efficacy of dupilumab on other PN endpoints	• Change from baseline in Participant Global Impression of Severity (PGIS) of PN to Week 4, Week 8, Week 12, and Week 24, respectively.<br>• Participant Global Impression of Change (PGIC) of PN at Week 4, Week 8, Week 12, and Week 24, respectively.
**PK**	
• To evaluate pharmacokinetic (PK) and pharmacodynamic (PD) outcome measures	• Serum functional dupilumab concentrations and PK profile.<br>• Pharmacodynamic response for selected biomarkers (total IgE).

a The early onset for significant change in the proportion of responders with WI-NRS reduction ≥4 between dupilumab and placebo will be claimed at Week xx if the p-value for Week xx is <0.05 and the remaining timepoints are also statistically significant.

b The early onset for significant change in WI-NRS from baseline between dupilumab and placebo will be claimed at Week xx if the p-value for Week xx is <0.05 and the remaining timepoints are also statistically significant.

c The early onset for reaching IGA PN-S(0,1) between dupilumab and placebo will be claimed at Week xx if the p-value for Week xx is <0.05 and the remaining timepoints are also statistically significant.

1.2.1 Estimands

The primary estimands defined for main endpoints are summarized in below Table 2. More details are provided in Section 4.

Table 2 - Summary of primary estimand for main endpoints

Endpoint Category	Estimands			
	**Endpoint(s)**[a]	**Population**	**Intercurrent event(s) strategy and missing data handling**	**Population-level summary**
**Primary objective: To demonstrate the efficacy of dupilumab on itch response in participants with PN, inadequately controlled on topical prescription therapies or when those therapies are not advisable.**				
Primary endpoint	Proportion of participants with improvement (reduction) in worst-itch numeric rating scale (WI-NRS) by ≥4 from baseline to Week 12	ITT	The intercurrent events will be handled as follows:<br>• Discontinuation of study treatment before Week 12: Off-study treatment data up to Week 12 will be included in the analysis (treatment policy strategy).<br>• Taking selected prohibited medications/procedures and/or rescue medications[b] prior to Week 12: Participants will be considered as non-responders (composite strategy).<br>In addition, the missing data imputation rules are as follows:<br>• Having missing data at Week 12: Participants will be considered as non-responders.	CMH test adjusted by documented history of atopy (atopic or non-atopic), stable use of TCS/TCI (yes or no), region (countries combined), and baseline antidepressant use (yes or no).

Endpoint Category	Estimands			
	Endpoint(s)[a]	Population	Intercurrent event(s) strategy and missing data handling	Population-level summary
**Secondary objective: To demonstrate the efficacy of dupilumab on additional itch endpoints in participants with PN, inadequately controlled on topical prescription therapies or when those therapies are not advisable.**				
Secondary endpoint	Time to onset of effect on pruritus as measured by proportion of participants with an improvement (reduction) in WI-NRS by ≥4 from baseline during the 24-week treatment period	ITT	The intercurrent events will be handled as follows:<br>• Discontinuation of study treatment before Week 24: Off-study treatment data up to Week 24 will be included in the analysis (treatment policy strategy).<br>• Taking selected prohibited medications/procedures and/or rescue medications[b] prior to Week 24: Analyses will be censored at Week 24 (composite strategy).<br>In addition, the missing data imputation rules are as follows:<br>• Discontinuing the study follow-up before Week 24: Analyses will be censored at the time of last WI-NRS assessment.	This time-to-event endpoint will be analyzed using the Cox proportional hazards model, including intervention group, documented history of atopy (atopic or non-atopic), stable use of TCS/TCI (yes or no), region (countries combined), and baseline antidepressant use (yes or no). The hazards ratio, its 95% confidence interval and p-value will be reported. Kaplan-Meier curves will be also provided.

Endpoint Category	Estimands			
	Endpoint(s)[a]	Population	Intercurrent event(s) strategy and missing data handling	Population-level summary
Secondary endpoint	Change from baseline in WI-NRS at Week 24	ITT	The intercurrent events will be handled as follows:<br>• Discontinuing the study treatment: all data collected following schedule after treatment discontinuation will be used in the analysis (treatment policy strategy).<br>• Taking selected prohibited medications/procedures and/or rescue medications[b] prior to Week 24: data will be set to missing values after the medication usage, and the participant's worst postbaseline value on or before the time of the medication usage will be used to impute missing endpoint value (for participants whose postbaseline values are all missing, the participant's baseline will be used to impute the missing endpoint value) (hypothetical strategy)<br><br>In addition, the missing data imputation rules are as follows:<br>• After discontinuation of the study treatment due to lack of efficacy prior to Week 24: WOCF approach will be used to impute missing data if needed[2].<br>• After discontinuation of the study treatment due to reasons other than lack of efficacy prior to Week 24: multiple imputation (MI) approach will be used to impute missing endpoint value, and this multiple imputation will use all participants excluding participants who have taken the selected prohibited medications and/or rescue medications prior to Week 24 and excluding participants who discontinue due to lack of efficacy prior to Week 24.	ANCOVA model with intervention group, documented history of atopy (atopic or non-atopic), stable use of TCS/TCI (yes or no), region (countries combined), baseline antidepressant use (yes or no), and relevant baseline measurement as covariates is used. Statistical inference obtained from all imputed data by ANCOVA model will be combined using Rubin's rule.

a Additional secondary objectives/endpoints are not included in this table but would be handled with a similar strategy as the endpoint type (ie continuous, proportion, time-to-event) at other weeks
b Selected prohibited medications and/or rescue medications are listed in Table 4.

2 SAMPLE SIZE DETERMINATION

The primary endpoint is the proportion of participants with WI-NRS reduction of ≥4 from baseline to Week 12. By assuming the response rate is [redacted]% and [redacted]% in the placebo and dupilumab arms, respectively, 56 participants/arm will provide 90% power to detect the difference of [redacted]% between dupilumab and placebo with Fisher exact test at 2-sided level of 0.05. Assuming 15% drop out during the 12 weeks of treatment, the target is to randomize 75 participants/arm with a cap of up to 10% of participants in the atopic population having active mild AD.

The assumptions were based on the effect of dupilumab versus placebo in WI-NRS reduction ≥4 at Week 16 observed in participants with moderate to severe AD as seen in studies of AD-1334 (Solo1) and AD 1416 (Solo2).

Approximately 150 participants will be randomized to dupilumab or placebo in a 1:1 ratio with stratification factors of documented history of atopy (atopic or non-atopic), stable use of TCS/TCI (yes or no), and country/territory code. Both the atopic and the non-atopic PN population will be capped at 60% of the total enrolled population.

The sample size calculation was performed using SAS 9.4 power procedure.

3 ANALYSIS POPULATIONS

The following populations for analyses are defined:

Table 3 - Populations for analyses

Population	Description
Screened	All participants who sign the ICF.
Randomized	The randomized population includes all participants with a treatment kit number allocated and recorded in the IRT database, and regardless of whether the treatment kit was used or not.<br>Participants treated without being randomized will not be considered randomized and will not be included in any efficacy population.
Intent-to-treat (ITT)	All randomized participants analyzed according to the intervention group allocated by randomization regardless if treatment kit is used or not
Efficacy	The ITT population
Safety	All participants randomly assigned to study intervention and who take at least 1 dose of study intervention. Participants will be analyzed according to the intervention they actually received.<br>Randomized participants for whom it is unclear whether they took the study medication will be included in the safety population as randomized.<br>For participants who accidentally receive different treatment from the planned, the actual intervention allocation for as-treated analysis will be the dupilumab group.<br>The PD analyses will be performed on the safety population.
Pharmacokinetic (PK)	The PK population includes all participants in the safety population with at least one non-missing result for functional dupilumab concentration in serum after first dose of the study treatment. Participants will be analyzed according to the intervention actually received.
Antidrug antibody (ADA)	ADA population includes all participants in the safety population who have at least one non-missing ADA result after first dose of the study treatment. Participants will be analyzed according to the intervention actually received.

ADA: antidrug antibody; ICF: informed consent form; ITT: intent to treat; PD: pharmacodynamics: PK: pharmacokinetic.

Participants exposed to study intervention before or without being randomized will not be considered randomized and will not be included in any analysis population. The safety experience of these participants will be reported separately.

Randomized participants for whom it is unclear whether they took the study intervention will be considered as exposed and will be included in the safety population as randomized.

For any participant randomized more than once, only the data associated with the first randomization will be used in any analysis population. The safety experience associated with any later randomization will be reported separately.

For participants receiving more than one study intervention (placebo and dupilumab) during the study, the intervention group for as-treated analysis will be the dupilumab group.

If >10% participants are impacted by the COVID-19 pandemic, additional summaries by COVID-19 subgroups will be provided. Participants impacted by the COVID-19 pandemic are defined as randomized participants with any critical or major deviation related to COVID-19 or who permanently discontinued study intervention or study due to COVID-19.

4 STATISTICAL ANALYSES

4.1 GENERAL CONSIDERATIONS

In general, continuous data will be summarized using the number of observations available, mean, standard deviation (SD), median, minimum, and maximum. Categorical and ordinal data will be summarized using the count and percentage of participants.

The baseline value of efficacy parameters is defined as the last available value before randomization and prior to the first dose of study medication unless below eDiary data (WI-NRS, Pain-NRS and Sleep-NRS) score or otherwise specified.

The baseline for weekly average WI-NRS (Pain-NRS and Sleep-NRS) score is defined as the average of daily non-missing scores obtained during the 7 days prior to randomization.

The baseline value of the other parameters is defined as the last available value prior to the first dose of investigational medicinal product (IMP) if the participant is treated, or the last available value up to randomization if the participant is not exposed to IMP.

Observation period

The observation period will be divided into 4 segments:

- The **pre-treatment period** is defined as the period up to first IMP administration.
- The **treatment-emergent (TE) period** is defined as the period from the first IMP administration to the last IMP administration + 98 days. The treatment-emergent period includes the following 2 periods:
  - The **on-treatment period** is defined as the period from the first IMP administration to the last administration of the IMP + 14 days
  - The **residual treatment period** is defined as the period from the end of the on-treatment period to the end of the treatment-emergent period.
- The **post-treatment period** is defined as the period from the end of the treatment-emergent period.

The on-study observation period is defined as the time from start of intervention until the end of the study defined as the status date collected on e-CRF page “Completion of End of Study”.

4.2 PARTICIPANT DISPOSITIONS

The number (%) of participants included in each of the analysis populations listed in Table 3 will be summarized.

Screen failures are defined as participants who consent to participate in the study but are not subsequently randomized. The number (%) of screen failures and reasons for screen failures will be provided in the screened population.

The number (%) of participants in the following categories will be provided:

- Randomized participants
- Randomized but not exposed participants
- Randomized and exposed participants
- Participants who completed the study treatment period as per protocol
- Participants who did not complete the study treatment period as per protocol and discontinued study treatment prior to Week 12 by main reason for permanent intervention discontinuation including due to COVID-19
- Participants who did not complete the study treatment period as per protocol and discontinued study treatment prior to Week 24 by main reason for permanent intervention discontinuation including due to COVID-19
- Participants who completed the study period as per protocol
- Participants who did not complete the study period as per protocol and discontinued study by main reason for study discontinuation including due to COVID-19.
- Vital status at last study contact

The number (%) of exposed and not randomized participants will also be summarized.

In addition, the number (%) of participants screened, screened-failed, randomized, with permanent intervention discontinuation and with early study discontinuation will be provided by country and site.

Protocol deviations

Critical and major protocol deviations (automatic or manual) will be summarized in the randomized population and according to COVID-19 impact (i.e. deviations related to the COVID-19 pandemic and deviations not related to the COVID-19 pandemic). In addition, deviations potentially impacting the primary endpoint analysis will be summarized.

4.3 PRIMARY ENDPOINT(S) ANALYSIS

4.3.1 Definition of endpoint(s)

The primary endpoint for this study is the proportion of participants with improvement (reduction) in worst-itch numeric rating scale (WI-NRS) by ≥4 from baseline to Week 12.

WI-NRS is a patient-reported outcome (PRO) comprised of a single item rated on a scale from 0 (“No itch”) to 10 (“Worst imaginable itch”). Participants are asked to rate the intensity of their worst pruritus (itch) over the past 24 hours using this scale.

The weekly average WI-NRS score at each week, which is defined as the average of daily non-missing scores within the week window of each week (see Section 5.4), will be used for analyses.

For efficacy analysis, Table 4 presents the prohibited and rescue medications/procedures which will be considered as intercurrent events if the last column indicates as “Yes” and therefore be handled in the estimands for endpoints defined in Table 2.

Table 4 - Selected prohibited medications/procedures and rescue medications that impact on efficacy

Medication/procedure	Comment	Data to be set as non-responder after taking medication in the main statistical analysis (Yes/No) [a]/ Selection criteria
**Prohibited medications/procedures**		
Systemic immunosuppressive/immunomodulating drugs (eg, systemic corticosteroids, cyclosporine, mycophenolate-mofetil, interferon gamma, Janus kinase inhibitors, azathioprine, methotrexate, hydroxychloroquine, dapsone, sulfasalazine, colchine, etc)	IMP to be discontinued	Yes (CDG[b] Immunosuppressant drugs Narrow and SDG[b]Corticosteroids Narrow)
Other monoclonal antibodies (that are biological response modifiers).	IMP to be discontinued	Yes (SDG[b] Monoclonal antibodies Narrow)
Phototherapy, including tanning beds.	IMP to be discontinued	Yes (CMQ[b] HLT Phototherapies, CMQ[b] Tanning_single PT)
Naltrexone or other opioid antagonist	IMP to be discontinued	Yes (SDG[b] Analgesia producing opioids NARROW)
Gabapentin, pregabalin, and thalidomide	IMP to be discontinued	Yes (CDG[b] Gabapentin mono and multi ingredients, Pregabalin mono and multi ingredients, or Thalidomide mono and multi ingredients)
Paroxetine, fluvoxamine or other SSRIs.	No IMP discontinuation, see requirement in the footnote	Yes[c,d] (CDG[b] N06AB selective serotonin reuptake inhibitors)
SNRIs.	No IMP discontinuation, see requirement in the footnote	Yes[c,d] (CDG[b] serotonin and norepinephrine reuptake inhibitors)
Amitriptyline or other tricyclic or tetracyclic antidepressants	No IMP discontinuation, see requirement in the footnote	Yes[c,d] (CDG[b] N06AA non-selective monoamine reuptake inhibitors)
Intralesional corticosteroid injections and cryotherapy.	No IMP discontinuation	Yes[d] (CMQ[b] Injection_single pt, cryotherapy or skin cryotherapy PT)
Sedating antihistamine	No IMP discontinuation	Yes[d,e] (CDG[b] sedating antihistamines)
Non-sedating antihistamine if used specifically for the treatment of itch secondary to AD or PN	No IMP discontinuation	Yes[d,e] (CDG[b] non-sedating antihistamines)

Medication/procedure	Comment	Data to be set as non-responder after taking medication in the main statistical analysis (Yes/No) [a]/ Selection criteria
**Rescue medications**		
Dermatological preparations of high potency or superpotent TCS and TCI	No IMP discontinuation	Yes[d] (CDG[b] calcineurin inhibitors and corticosteroids narrow, Ticked 'RESCUE THERAPY' in CRF page)

a. When yes, the estimand for the intercurrent event handling strategy will be as follows: hypothetical for continuous endpoints, and composite for responder and time-to-event endpoints. When no, a treatment policy strategy will be applied.
b. CDG=company drug groupings, CMQ=company MedDRA query, SDG= Standardized drug groupings.
c. Only if the antidepressant is initiated during the study or its dose is increased from baseline provided that medication was taken at least 3 months prior to screening.
d. As per medical adjudication, see Appendix 7 for details.
e. Only if the medication is initiated at Week 12 or Week 24, or the dose is increased from Week 11 to Week 12 or from Week 23 to Week 24.

Blinded review of prohibited/rescue treatment (medication or procedure) based on Table 4 and a pre-specified algorithm (Appendix 7) will be implemented before database locks by considering the type of medication or procedure, indication, timing, frequency and the potential impact of the use of the prohibited medications/procedures and rescue medications.

4.3.2 Main analytical approach

The primary estimand for the primary endpoint is the treatment policy/composite approach as defined in Table 2.

The primary analysis population for the efficacy endpoints will be the ITT population.

The following null hypothesis H0 and alternative hypothesis H1 will be tested for dupilumab against placebo:

- H0: No treatment difference between dupilumab and placebo.
- H1: There is a treatment difference between dupilumab and placebo

The primary analysis will be conducted by using Cochran–Mantel–Haenszel test (CMH) test adjusted by documented history of atopy (atopic or non-atopic), stable use of TCS/TCI (yes or no), region, and baseline anti-depressant use (yes or no). Comparisons of the response rates between dupilumab and placebo will be derived. In addition, odds ratio and response rate difference as well as the corresponding 95% confidence intervals (CI) will be provided along with the p-values.

As defined in Table 2 for participants discontinuing the study treatment before Week 12, their off-study treatment values measured up to Week 12 will be included in the analysis. Participants taking selected prohibited medications/procedures and/or rescue medications (see Table 4) prior to Week 12 or having missing data at Week 12 will be considered non-responders.

4.3.3 Sensitivity analysis

For the primary estimand for the primary endpoint, no sensitivity analysis will be performed. However, two supplementary analyses will be performed as described in the section below.

4.3.4 Supplementary analyses

The following supplementary analyses will be performed:

As-observed analysis (including all data after taking selected prohibited and/or rescue medications)

The data collected after taking all prohibited medications and/or rescue medications will be included in the supplementary analysis to evaluate the robustness of the primary analysis results with respect to the method of handling data while taking selected prohibited medications (e.g. treatment policy strategy). In addition, for participants discontinuing the study treatment before Week 12, their off-study treatment values measured up to Week 12 will be included in the analysis. The patients having missing data at Week 12 regardless of reason(s) will be considered non-responders at that timepoint.

Hybrid method analysis (the worst-observation carried forward (WOCF) and multiple imputation (MI))

In the primary analysis of change from baseline in WI-NRS (continuous variable) at Week 12, the hybrid method of the WOCF and MI will be used (see Section 4.4.2). Similar to the continuous variable, the same imputation method will be used in the analysis of the proportion of participants with improvement (reduction) in WI-NRS by ≥4 from baseline to Week 12, which is consistent for the intercurrent event strategy and missing data handling in the binary variables and continuous variable. That is, after the imputation of continuous WI-NRS data at Week 12 using the hybrid method of the WOCF and MI (see Section 4.4.2), responders will be defined as patients with improvement in WI-NRS by ≥4 from baseline to Week 12 in each of the imputed datasets with about 40 imputations, and then the CMH test adjusted by documented history of atopy (atopic or non-atopic), stable use of TCS/TCI (yes or no), region, and baseline anti-depressant use (yes or no) will be used. Statistical inference obtained from all imputed data will be combined using Rubin’s rule.

4.3.5 Subgroup analyses

To assess the homogeneity of the treatment effect across various subgroups, analyses will be performed on the primary endpoint across the following subgroups (categories with fewer than 5 participants may be combined with other categories):

- Participants without a current diagnosis of AD
- Age group (<65, ≥65 years)

- Gender (Male, Female)
- Region
- Territory
- Race (Caucasian/White, Black/of African descent, Asian/Oriental, Others)
- Ethnicity (Hispanic or Latino, Not Hispanic or Latino)
- Baseline weight (<60, ≥60- < 90, ≥ 90 kg)
- Baseline BMI (<25, ≥25- <30, ≥30 kg/m^2)
- History of atopy (atopic or non-atopic)
- Stable use of TCS/TCI (yes or no)
- Antidepressant use (yes or no) at baseline
- HIV (positive vs. negative)
- Baseline IGA PN-S moderate vs severe (3 vs. 4)

To test the interaction between intervention and subgroup factor, a logistic regression model incorporating subgroup-by-treatment interaction will be built for each subgroup factor except the subgroup of participants without a current diagnosis of AD (very few AD participants will be excluded). The model will include all the covariates in the main statistical model plus the subgroup variable and the subgroup-by-treatment interaction. A p-value for the test of interaction will be provided.

In each subgroup, the treatment effects for the primary endpoint will be provided, as well as the corresponding 95% CI, using the same method as applied to the primary analysis. Forest plots will be provided.

4.4 SECONDARY ENDPOINT(S) ANALYSIS

The weekly average Pain-NRS or Sleep-NRS score at each week, which is defined as the average of non-missing daily scores within the week window of each week (see Section 5.4), will be used for analyses.

4.4.1 Key/Confirmatory secondary endpoint(s)

4.4.1.1 Definition of endpoint(s)

There are two key secondary endpoints:

- Proportion of participants with improvement (reduction) in WI-NRS by ≥4 from baseline to Week 24, and
- Proportion of participants with IGA PN-S 0 or 1 score at Week 24.

Investigator's global assessment for prurigo nodularis (IGA PN)

The IGA PN is a clinician-reported outcome (ClinRO) that allows clinicians to assess the activity of PN (IGA PN-A) using a 5-point scale from 0 (clear) to 4 (severe); and the stage of the disease (IGA PN-S) using a 5-point scale from 0 (clear) to 4 (severe).

4.4.1.2 Main analytical approach

For both key secondary efficacy endpoints, the analysis will be conducted by using CMH test adjusted by documented history of atopy (atopic or non-atopic), stable use of TCS/TCI (yes or no), region, and baseline anti-depressant use (yes or no). The same estimand, i.e., intercurrent event strategy and missing data handling method, as the primary endpoint, by replacing Week 12 with Week 24, is used.

4.4.2 Supportive secondary endpoint(s)

The other secondary endpoints are as follows:

- Proportion of participants with WI-NRS reduction ≥4 over time until Week 24.
- Proportion of participants with WI-NRS reduction ≥4 at Week 4
- Proportion of participants with IGA PN-S 0 or 1 score at Week 12
- Proportion of participants with IGA PN-S 0 or 1 score at Week 8
- Proportion of participants with IGA PN-S 0 or 1 score at Week 4
- Proportion of participants with IGA PN-A 0 or 1 score at Week 24
- Proportion of participants with IGA PN-A 0 or 1 score at Week 12
- Proportion of participants with IGA PN-A 0 or 1 score at Week 8
- Proportion of participants with IGA PN-A 0 or 1 score at Week 4
- Time to onset of effect on pruritus as measured by proportion of participants with an improvement (reduction) in WI-NRS by ≥4 from baseline during the 24-week treatment period
- Change from baseline in WI-NRS at Week 24
- Change from baseline in WI-NRS at Week 12
- Percent change from baseline in WI-NRS at Week 24
- Percent change from baseline in WI-NRS at Week 12
- Percent change from baseline in WI-NRS at Week 4
- Percent change from baseline in WI-NRS at Week 2
- Percent change from baseline in WI-NRS over time until Week 24.

- Onset of action in change from baseline in WI-NRS (first $p < 0.05$ difference from placebo in the daily WI-NRS that remains significant at subsequent measurements) until Week 12
- Change from baseline in IGA PN-S score at Week 24
- Change from baseline in IGA PN-S score at Week 12
- Change from baseline in IGA PN-S score at Week 8
- Change from baseline in IGA PN-S score at Week 4
- Change from baseline in HRQoL, as measured by Dermatology Life Quality Index (DLQI) to Week 24
- Change from baseline in HRQoL, as measured by Dermatology Life Quality Index (DLQI) to Week 12

Dermatology life quality index (DLQI)

The DLQI is a PRO developed to measure dermatology-specific HRQoL in adult participants. The instrument comprises 10 items assessing the impact of skin disease on participants' HRQoL over the previous week. The items cover symptoms, leisure activities, work/school or holiday time, personal relationships including intimate, the side effects of treatment, and emotional reactions to having a skin disease. It is a validated questionnaire used in clinical practice and clinical trials. Response scale is a 4-point Likert scale (0 = "not at all" and 3 = "very much") for nine items. The remaining one item about work/studying asks whether work/study has been prevented and then (if "No") to what degree the skin condition has been a problem at work/study; the item is rated on a 3-point Likert scale ("Not at all" to "A lot"). Overall scoring ranges from 0 to 30, with a high score indicative of a poor HRQoL.

Time-to-event secondary efficacy endpoint

Time to first onset of effect on pruritus defined as an improvement (reduction) in WI-NRS by ≥4 from baseline during the 24-week treatment period will be analyzed using the Cox proportional hazards model, including treatment, documented history of atopy (atopic or non-atopic), stable use of TCS/TCI (yes or no), region, and baseline anti-depressant use (yes or no). The hazards ratio, its 95% confidence interval and p-value will be reported. Kaplan-Meier curves will be also provided.

For time to first WI-NRS response (WI-NRS by ≥4 from baseline), participants who receive selected prohibited medications and/or rescue medications (see Table 4), data prior to start of the medications will be used, but after medication start, the participants' data will not be used and they will be censored at Week 24 (i.e. Day 172). For other participants, all available data up to Week 24 (i.e. Day 172) including those collected during the off-treatment period will be used. Participants without events will be censored at Day 172 or their last WI-NRS assessment date if discontinued from the study, whichever is earlier.

Secondary efficacy endpoints that measure binary responses

Secondary efficacy endpoints that measure binary responses will be analyzed in the same fashion as the primary endpoint using primary statistical model.

Continuous secondary efficacy endpoints

The primary estimand for continuous secondary efficacy endpoints is defined in Table 2 and will be analyzed using an analysis of covariance (ANCOVA) model with intervention group, documented history of atopy (atopic or non-atopic), stable use of TCS/TCI (yes or no), region (countries combined), baseline antidepressant use (yes or no), and relevant baseline measurement as covariate, with intercurrent event strategy and missing data handling as defined as in Table 2. Specifically, data of participants taking selected prohibited medications/procedures and/or rescue medications will be set to missing after the medication usage, and the worst postbaseline value on or before the time of the medication usage will be used to impute missing endpoint value (for participants whose postbaseline values are all missing, the baseline will be used to impute). Participants who discontinue the treatment prematurely are encouraged to follow the planned clinical visits and in those participants who did not take the selected prohibited medications/procedures and/or rescue medications, all data collected after treatment discontinuation will be used in the analysis. For these participants, missing data may still happen despite all efforts have been tried to collect the data after treatment discontinuation. For participants who discontinue due to lack of efficacy, all data collected after discontinuation will be used in the analysis, and a WOCF approach will be used to impute missing data if needed. For participants who discontinued not due to lack of efficacy, a multiple imputation (MI) approach will be used to impute missing endpoint value, and this MI method will use all participants excluding participants who have taken the selected prohibited medications/procedures and/or rescue medications prior to timepoint of endpoint of interest and excluding patients who discontinue due to lack of efficacy.

Each of the imputed complete data will be analyzed by fitting an ANCOVA model as described above. The imputation number will be about 40. Statistical inference obtained from all imputed data will be combined using Rubin's rule. Descriptive statistics including number of participants, mean, standard error, and least squares (LS) mean changes (and standard error) will be provided. In addition, difference of the dupilumab group against placebo in LS means and the corresponding 95% confidence intervals (CI) will be provided along with the p-values.

See Section 5.5 for the sample SAS code for the imputation and the analysis.

4.5 TERTIARY/EXPLORATORY ENDPOINT(S) ANALYSIS

4.5.1 Definition of endpoint(s)

The exploratory endpoints are as follows:

- Use of high potency or superpotent TCS rescue medication through Week 24
- Use of systemic immunosuppressant through Week 24, constituting treatment failure

- Change from baseline in Hospital Anxiety and Depression Scale (HADS) total score to Week 24
- Change from baseline in EQ-5D-5L Single Index score to Week 24
- Change from baseline in EQ-5D visual analog scale (VAS) to Week 24
- Change from baseline in Pain-NRS to Week 4, Week 8, Week 12, and Week 24, respectively
- Change from baseline in Sleep-NRS to Week 4, Week 8, Week 12, and Week 24, respectively
- Missed school/work days through Week 24
- Incidence of skin-infection TEAEs (excluding herpetic infections) through Week 24
- Proportion of participants who achieve ≥75% healed lesions from PAS at Week 4, Week 8, Week 12, and Week 24, respectively
- Change from baseline in exact number of lesions in representative area (as determined from PAS) at Week 4, Week 8, Week 12, and Week 24, respectively
- Change from baseline in Participant Global Impression of Severity (PGIS) of PN to Week 4, Week 8, Week 12, and Week 24 respectively
- Proportion of participants with PGIS score of "none" at Week 4, Week 8, Week 12, and Week 24 respectively
- Proportion of participants with PGIS score of "non" or "mild" at Week 4, Week 8, Week 12, and Week 24 respectively
- Participant Global Impression of Change (PGIC) of PN at Week 4, Week 8, Week 12, and Week 24, respectively
- Proportion of participants with PGIC score of "very much better" at Week 4, Week 8, Week 12, and Week 24 respectively
- Proportion of participants with PGIC score of "very much better" or "moderately better" at Week 4, Week 8, Week 12, and Week 24 respectively

<u>Hospital anxiety and depression scale (HADS)</u>

The HADS is a PRO instrument for screening anxiety and depression in non-psychiatric populations; repeated administration also provides information about changes to a participant's emotional state. The HADS consists of 14 items, 7 each for anxiety and depression symptoms; possible scores range from 0 to 21 for each subscale. The following cut-off scores are recommended for both subscales:

- 0 to 7: normal
- 8 to 10: borderline abnormal (borderline case)
- 11 to 21: abnormal

Euroqol 5 dimensions questionnaire (EQ-5D)

The Euroqol-5 dimensions (EQ-5D) is a standardized PRO measure of health status developed by the EuroQOL Group in order to provide a simple, generic measure of health for clinical and economic appraisal. The EQ-5D consists of 2 parts: the descriptive system and the EQ visual analog scale (VAS). The EQ-5D descriptive system comprises the following 5 dimensions: mobility, self-care, usual activities, pain/discomfort, and anxiety/depression. Each dimension has 5 levels of perceived problems: “no problem”, “slight problems”, “moderate problems”, “severe problems” and “inability to do the activity”. The respondent is asked to indicate his/her health state by ticking (or placing a cross) in the box against the most appropriate statement in each of the 5 dimensions; this results in a 1-digit number expressing the level for that dimension. The digits for 5 dimensions can be combined in a 5-digit number describing the respondent’s health state. The EQ VAS records the respondent’s self-rated health on a vertical VAS where the endpoints are labeled “best imaginable health state (100)” and “worst imaginable health state (0)”. This information can be used as a quantitative measure of health outcome as judged by the individual respondents.

Pain and sleep numeric rating scales (Pain-NRS, Sleep-NRS)

Participants will be asked to rate their worst skin pain in the past 24 hours using a 0 to 10 NRS, with 0 = No pain to 10 = Worst pain possible.

In addition, participants will be asked to rate their sleep quality on their past night upon awakening, using a 0 to 10 NRS, with 0 = Worst possible sleep and 10 = Best possible sleep.

Participants will complete the skin pain NRS and sleep quality NRS once a day.

Prurigo activity score (PAS)

The PAS is a ClinRO measurement. The original PAS questionnaire Version 0.9 consists of 7 items, developed by expert clinicians in PN.

A 5-item simplified version of the PAS will be used in the current study. Item 4 (exact number of pruriginous lesions in representative area) and Item 5b (percentage of healed prurigo lesions in all pruriginous lesions) will be used for analyses. In addition, Item 2 (number of prurigo lesions) may be used for analysis too.

Participant Global Impression of Change of disease (PGIC) and Participant Global Impression of Severity (PGIS)

The PGIC is a one-item questionnaire that asks participants to provide the overall self-assessment of change in their PN overall on a 7-point scale, compared to just before participant started taking the study injection. Response choices are: 0 = “Very much better”, 1 = “Moderately better”, 2 = “A little better”, 3 = “No change”, 4 = “A little worse”, 5 = “Moderately worse”, 6 = “Very much worse”.

The PGIS is a one-item questionnaire that asks participants to provide the overall self-assessment of their disease severity on a 4-point scale for the past week. Response choices are: 1 = “none”, 2 = “Mild”, 3 = “Moderate”, 4 = “Severe”.

Missed school/work days

Participants who are employed or enrolled in school will be asked to report the number of sick leave/missed school days since the last study assessment.

4.5.2 Main analytical approach

Exploratory efficacy endpoints will be analyzed using the same methodology as secondary efficacy for similar data (continuous or proportion) except the endpoints below.

- Use of high potency or superpotent TCS rescue medication through Week 24
- Use of systemic immunosuppressant through Week 24, constituting treatment failure
- Missed school/work days through Week 24
- Incidence of skin-infection TEAEs (excluding herpetic infections) through Week 24

The number of participants and percentage with the events for these endpoints will be provided in observed cases. In addition, the time (week) to first event for those participants will also be analyzed using Kaplan-Meier method and the duration of the rescue medication use may be summarized if applicable.

An additional exploratory endpoint of time to first select prohibited/rescue medication that impact efficacy will be provided. This includes medications where WOCF will be applied (see Table 4).

4.6 MULTIPLICITY ISSUES

The multiplicity procedure is proposed to control the overall type-I error rate for testing the primary and selected secondary endpoints. The overall alpha is 0.05. The comparisons with placebo will be tested based on the hierarchical order below at 2-sided $\alpha = 0.05$:

Primary efficacy endpoints:

- Proportion of participants with improvement (reduction) in WI-NRS by ≥4 from baseline to Week 12.

Secondary efficacy endpoints:

- Proportion of participants with improvement (reduction) in WI-NRS by ≥4 from baseline to Week 24
- Proportion of participants with IGA PN-S 0 or 1 score at Week 24

Other secondary efficacy endpoints

- Percent change from baseline in WI-NRS at Week 24
- Proportion of participants with IGA PN-S 0 or 1 score at Week 12
- Change from baseline in HRQoL, as measured by Dermatology Life Quality Index (DLQI) to Week 24
- Change from baseline in Pain-NRS to Week 24
- Change from baseline in Sleep-NRS to Week 24
- Change from baseline in Hospital Anxiety and Depression Scale (HADS) total score to Week 24

The study is considered positive when the primary endpoint achieves statistical significance.

4.7 SAFETY ANALYSES

All safety analyses will be performed on the safety population as defined in Section 3, unless otherwise specified, using the following common rules:

- The analysis of the safety variables will be descriptive, and no testing is planned.
- Safety data in participants who do not belong to the safety population (e.g., exposed but not randomized) will be provided separately.

4.7.1 Extent of exposure

The extent of IMP exposure will be assessed by the duration of IMP exposure and compliance and summarized within the safety population.

Duration of IMP exposure

Duration of IMP exposure is defined as last dose date – first dose date + 14 days, regardless of unplanned intermittent discontinuations. Duration of IMP exposure will be summarized descriptively as a quantitative variable (number, mean, SD, median, minimum, and maximum). In addition, duration of treatment exposure will also be summarized categorically by numbers and percentages for each of the following categories and cumulatively according to these categories:

- >0 and ≤2 weeks
- >2 and ≤4 weeks
- >4 and ≤8 weeks
- >8 and ≤12 weeks
- >12 and ≤16 weeks
- >16 and ≤20 weeks
- >20 and ≤24 weeks

- >24 weeks and ≤ 24 weeks + 3 days
- > 24 weeks + 3 days

Additionally, the cumulative duration of treatment exposure will be provided, defined as the sum of the duration of treatment exposure for all participants, and will be expressed in participant years.

Treatment compliance

A given administration will be considered noncompliant if the participant did not take the planned dose as required by the protocol. No imputation will be made for participants with missing or incomplete data.

Percentage of treatment compliance for a participant will be defined as the number of administrations that the participant was compliant divided by the total number of administrations that the participant was planned to take from the first administration of IMP up to the actual last administration of IMP.

Treatment compliance will be summarized quantitatively and categorically: <80%, ≥80%.

Cases of overdose (defined as at least twice the intended dose during an interval of less than 11 days) will constitute SAEs and will be listed as such.

4.7.2 Adverse events

General common rules for adverse events

All adverse events (AEs) will be coded to a lower-level term (LLT), preferred term (PT), high-level term (HLT), high-level group term (HLGT), and associated primary system organ class (SOC) using the Medical Dictionary for Regulatory Activities (MedDRA) version currently in effect at Sanofi at the time of database lock.

The AEs will be analyzed in the following 3 categories:

- Pre-treatment AEs: AEs that developed, worsened or became serious during the pre-treatment period.
- Treatment-emergent adverse events (TEAE)s: AEs that developed, worsened or became serious during the treatment-emergent period
- Post-treatment AEs: AEs that developed, worsened or became serious during the post-treatment period

Similarly, the deaths will be analyzed in the pre-treatment, treatment-emergent and post-treatment periods.

The primary focus of AE reporting will be on TEAEs. Pre-treatment and post-treatment AEs will be described separately.

An AE with incomplete or missing date/time of onset (occurrence, worsening, or becoming serious) will be classified as a TEAE unless there is definitive information to determine it is a pre-treatment or a post-treatment AE.

If the assessment of the relationship to IMP is missing for an AE, this AE will be assumed as related to IMP. If the severity is missing for 1 of the treatment-emergent occurrences of an AE, the severity will be imputed with the maximal severity of the other occurrences. If the severity is missing for all the occurrences, the severity will be left as missing.

Multiple occurrences of the same event in the same participant will be counted only once in the tables within a treatment phase.

The AE tables will be sorted as indicated in Table 5.

Table 5 - Sorting of AE tables

AE presentation	Sorting rules
SOC, HLGT, HLT and PT	By the internationally agreed SOC order and by alphabetic order of HLGTs, HLTs and PTs.
SOC, HLT and PT	By the internationally agreed SOC order and by alphabetic order of HLTs and PTs.
SOC and PT	By the internationally agreed SOC order and decreasing frequency of PTs[a, b]
SMQ/CMQ and PT	By decreasing frequency of SMQs/CMQs and PTs[a]
PT	By decreasing frequency of PTs[a]

a Sorting will be based on the SAR231893 dupilumab group
b The table of all TEAEs presented by SOC and PT will define the presentation order for all other tables (eg, treatment-emergent SAE) presented by SOC and PT, unless otherwise specified.

<u>Analysis of all adverse events</u>

The overview of TEAE with the details below will be generated:

- Any TEAE
- Any treatment emergent SAE
- TEAE leading to death
- Any TEAE leading to permanent intervention discontinuation

The AE summaries of Table 6 will be generated with number (%) of participants experiencing at least one event.

Table 6 - Analyses of adverse events

Type of AE	MedDRA levels
All TEAE	Primary SOC, HLGT, HLT and PT
	Primary SOC and PT
	PT
	Primary and secondary SOC, HLGT, HLT and PT
Common TEAE (≥2% and 5% in any group)	Primary SOC and PT
TEAE related to IMP as per Investigator's judgment	Primary SOC, HLGT, HLT and PT
	Primary SOC and PT
TEAE by maximal intensity	Primary SOC and PT
Treatment emergent SAE	Primary SOC, HLGT, HLT and PT
	Primary SOC and PT
Treatment emergent SAE related to IMP as per Investigator's judgment	Primary SOC, HLGT, HLT and PT
TEAE leading to permanent intervention discontinuation	Primary SOC, HLGT, HLT and PT
	Primary SOC and PT
TEAE leading to death (death as an outcome of the AE as reported by the Investigator in the AE page)	Primary SOC, HLGT, HLT and PT
Pretreatment AE	Overview[a]
	Primary SOC and PT

a Will include the following AE categories: any AEs, any serious AEs, any AEs leading to death, any AEs leading to permanent intervention discontinuation

Analysis of deaths

In addition to the analyses of deaths included in Table 5 the number (%) of participants in the following categories will be provided:

- Deaths during the treatment-emergent and post-treatment periods
- Deaths in non-randomized or randomized but not treated participants

Analysis of adverse events of special interest (AESIs) and other AEs of interest

Adverse events of special interest (AESIs) and other AEs of interest will be selected for analyses as indicated in Table 7. Number (%) of participants experiencing at least one event will be provided for each event of interest. Tables will be sorted as indicated in Table 5.

Table 7 - Selections for AESIs and other AEs of interest

AE Grouping	Criteria
**AESI**	
Anaphylactic reaction	Anaphylactic reaction algorithmic approach (Introductory Guide for Standardised MedDRA Queries (SMQs) Version 18.1): includes anaphylactic reaction narrow SMQ (20000021) terms and programmatic identification of cases based on occurrence of at least two preferred terms meeting the algorithm criteria occurring within 24 hours of each other. The latter cases identified using the algorithm will undergo blinded medical review taking into account the timing of events relative to each other and to IMP administration for final determination of an anaphylactic reaction or not.
Systemic hypersensitivity reactions	SMQ [20000214] hypersensitivity narrow search and [AE corrective treatment/therapy='Y' or Action taken with IMP='Drug withdrawn' or Action taken with IMP='Drug interrupted'] followed by blinded medical review (documented process) for selection of relevant systemic hypersensitivity events
Helminthic infections	CMQ10544 based on HLGT as "Helminthic disorder"
Any severe type of conjunctivitis	CMQ10498 based on PTs (See Section 5.6)[a] and "Severe" ticked in Adverse Events eCRF page
Any severe type of blepharitis	CMQ10497 based on HLT as "Lid, lash and lacrimal infections, irritations and inflammations" and "Severe" ticked in Adverse Events eCRF page
Keratitis	CMQ10642 based on the following PTs [keratitis, allergic keratitis, ulcerative keratitis, atopic keratoconjunctivitis, herpes ophthalmic, ophthalmic herpes simplex, corneal infection][a]
Clinically symptomatic eosinophilia (or eosinophilia associated with clinical symptoms)[b]	CMQ10641 based on HLT = Eosinophilic disorders or PT=Eosinophil count increased
Pregnancy of a female participants entered in a study as well as pregnancy occurring in a female partner of a male participant entered in a study with IMP/NIMP	"Pregnancy" or "Partner Pregnancy" checked on the Pregnancy eCRF page as reported by the investigator
Significant ALT elevation	ALT >5 x ULN in participants with baseline ALT ≤2 x ULN;<br>OR ALT >8 x ULN if baseline ALT >2 x ULN
Symptomatic overdose with IMP	Symptomatic Overdose is answered Yes, with Overdose of IMP answered Yes on AE eCRF.
Symptomatic overdose with NIMP	Symptomatic Overdose is answered Yes, with Overdose of NIMP answered Yes on AE eCRF.
**Other selected AE Grouping**	
Serious injection site reactions or severe injection site reactions that last longer than 24 hours	HLT = 'Injection site reaction' and either with serious status, or with severe status and (AE end date/time - AE start date/time) ≥ 24 hours or ongoing

AE Grouping	Criteria
Severe or serious infection	Primary SOC = 'Infections and infestations' and with severe or serious status
Drug-related hepatic disorder	SMQ [20000006] Drug-related hepatic disorders- narrow
Injection site reaction	HLT = 'Injection site reaction'
Malignancy	SMQ [20000091]- Malignant or unspecified tumors narrow
Suicidal behavior	CMQ10639 based on the following PTs [Completed suicide, Suicidal ideation, Depression suicidal, Suicidal behavior, Suicide attempt][a]
Conjunctivitis (narrow)	CMQ10644 based on the following PTs [Conjunctivitis, Conjunctivitis allergic, Conjunctivitis bacterial, Conjunctivitis viral, Atopic keratoconjunctivitis][a]
Conjunctivitis (broad)	CMQ10645 based on the following PTs [Conjunctivitis, Conjunctivitis allergic, Conjunctivitis bacterial, Conjunctivitis viral, Atopic keratoconjunctivitis, Blepharitis, Dry eye, Eye irritation, Eye pruritus, Lacrimation increased, Eye discharge, Foreign body sensation in eyes, Photophobia, Xerophthalmia, Ocular hyperaemia, Conjunctival hyperaemia][a]
Conjunctivitis (FDA)	CMQ10643 based on the following PTs [Conjunctivitis, Conjunctivitis allergic, Conjunctivitis bacterial, Conjunctivitis viral, Eye irritation, Eye inflammation, Giant papillary conjunctivitis][a]

a The list of terms may be adjusted according to MedDRA version changes

b All cases of Eosinophilia will be included in the analysis, where cases associated with clinical symptoms will be further described in the CSR

The following summaries will be provided:

- All TEAEs, by selected standardized MedDRA query (SMQ)/Customized MedDRA query (CMQ) and PT or by laboratory values (as in alanine aminotransferase (ALT) elevation), showing the number (%) of participants with at least 1 PT,
- For each AESI and other selected AE groupings,
  - Number (%) of participants with any specific TEAE
  - Number (%) of participants with any specific serious AE (regardless of treatment emergent status)
  - Number (%) of participants with any specific treatment emergent serious AE
  - Number (%) of participants with any specific AE leading to death
  - Number (%) of participants with any specific TEAE leading to permanent study drug discontinuation
  - Number (%) of participants with any specific TEAE related to IMP reported by investigator

- Number (%) of participants with any specific TEAE by maximum intensity, corrective treatment, and final outcome
- Number of any specific TEAE adjusted by the exposure duration
- Number of participants with any specific TEAE adjusted by the exposure duration at risk. For each specific TEAE, Kaplan-Meier estimates of cumulative incidence at Week 12, and 24 and K-M plot may be provided to depict the course of onset over time if the number of events is large enough.
- Number (%) of participants with injection site reactions by IMP injection.
- Number (%) of participants with different number of injection site reactions.

- In addition, AESIs reported by the investigator in eCRF will be summarized separately.

4.7.3 Additional safety assessments

4.7.3.1 Laboratory variables, vital signs and electrocardiograms (ECGs)

The following laboratory variables, vital signs and electrocardiogram (ECG) variables will be analyzed. They will be converted into standard international units.

- Hematology:
  - Red blood cells and platelets and coagulation: hemoglobin, hematocrit, red blood cell count, platelet count
  - White blood cells: white blood cell count, neutrophils, lymphocytes, monocytes, basophils, eosinophils
- Clinical chemistry:
  - Metabolism: glucose, total cholesterol, total protein, creatine phosphokinase
  - Electrolytes: sodium, potassium, chloride, calcium, bicarbonate
  - Renal function: creatinine, blood urea nitrogen, uric acid
  - Liver function: alanine aminotransferase, aspartate aminotransferase, alkaline phosphatase, lactate dehydrogenase, total bilirubin, albumin
  - Pregnancy test: Serum β-human chorionic gonadotropin (all female participants) will be performed at screening (V1) in women of childbearing potential, and a urine pregnancy test will be performed at V2 and every 4 weeks thereafter.
  - Hepatitis screen: hepatitis B surface antigen (HBs Ag), hepatitis B surface antibody (HBs Ab), hepatitis B core antibody (HBc Ab), hepatitis C virus antibodies (HCV Ab) will be tested at screening (V1). In case of results showing HBs Ag (negative) and HBc Ab (positive), an hepatitis B virus (HBV) deoxyribonucleic acid (DNA) testing will be performed and should be confirmed negative prior to randomization. In case of results showing HCV Ab (positive), an HCV ribonucleic acid (RNA) testing will be performed and should be confirmed negative prior to randomization.
  - HIV screen: Anti-HIV-1 and HIV-2 antibodies will be tested at Visit 1.

- TB test (performed locally if required and results noted in the eCRF).

- Urinalysis:
  - Urinalysis will include specific gravity, pH, glucose, ketones, blood, protein, nitrate, leukocyte esterase, urobilinogen and bilirubin. In case the urine dipstick test result is abnormal, a urine sample should be sent into the central laboratory for microscopic analysis. Creatinine and leukotriene are tested by the central laboratory.
- Vital signs: pulse rate (beats per minute), systolic and diastolic blood pressure (mmHg) in a semi-supine or sitting position after 5 minutes, weight, respiratory rate (breaths per minute), temperature (degrees Celsius) and height (screening only).
- ECG variables: heart rate, PR, QRS, QT, and QTcF intervals after 10 minutes of rest in the supine position. Data are locally collected and read at screening (V1) and Week 24 (V6).

Data below the lower limit of quantitation/detection limit (LLOQ) will be replaced by half of the LLOQ, data above the upper limit of quantification will be replaced by ULOQ value.

Quantitative analyses

For all laboratory variables, vital signs and ECG variables above, descriptive statistics for results and changes from baseline will be provided for each analysis window, the last value and the worst value during the on-treatment period. These analyses will be performed using central measurements only (when available) for laboratory variables.

For all parameters, mean changes from baseline with the corresponding standard error will be plotted over time.

Analyses according to PCSA

Analysis of potentially clinically significant abnormality (PCSA) will be performed based on the PCSA list in effect at Sanofi at the time of the database lock. For parameters for which no PCSA criteria are defined, similar analyses will be done using the normal range, if applicable.

Analyses according to PCSA will be performed based on the worst value during the treatment-emergent period, using all measurements (either local or central, either scheduled, nonscheduled or repeated).

For laboratory variables, vital signs and ECG variables above, the incidence of participants with at least one PCSA during the treatment-emergent period will be summarized regardless of the baseline level and according to the following baseline status categories:

- Normal/missing
- Abnormal according to PCSA criterion or criteria

Additional analyses for suspect drug-induced liver injury

The following additional analyses will be performed for drug-induced liver injury:

- Time to onset of the initial ALT or aspartate aminotransferase (AST) elevation (>3 x ULN) and total bilirubin elevation (>2 x ULN) during the treatment-emergent period will be analyzed using Kaplan-Meier method.
- A graph of the distribution of peak values of ALT versus peak values of total bilirubin during the treatment-emergent period will be provided.
- For each liver function test (eg, ALT), participants having a PCSA (eg, ALT >5 ULN) will be summarized using the following categories: Returned to baseline PCSA status (or returned to value ≤ ULN in case of missing baseline) before last IMP dose, Returned to baseline PCSA status after last IMP dose, Never returned to baseline PCSA status, No assessment after elevation. This summary will be performed by categories of elevation (ALT >3, >5, >10, >20 ULN).

4.8 OTHER ANALYSES

4.8.1 Pharmacokinetic analyses

Predose serum dupilumab concentrations at Visit 2 (Day 1), dupilumab trough levels at Week 4, Week 8, Week 12, Week 24/EOT Visit and posttreatment serum dupilumab at Week 36/EOS Visit will be provided.

Serum concentrations of SAR231893 (REGN668) will be summarized in the PK population using arithmetic and geometric means, SD, standard error of the mean (SEM), coefficient of variation (CV), minimum, median and maximum per sampling time. If date and/or time of the drug injection and/or sampling is missing then the concentration will not be taken into account. For drug-treated participants, where concentration values are below the lower limit of quantification (LLOQ), one-half of the LLOQ will be used. Values will be expressed in the tables with no more than three significant figures. For participants in the placebo group, concentration values that are below the LLOQ will be taken into account with a plasma concentration considered equal to 0.

4.8.2 Immunogenicity analyses

Anti-drug antibody (ADA) status to dupilumab (negative or titer value, if positive in the ADA assay) at Visit 2 (Day 1), Week 12, Week 24/EOT and follow up at Week 36 will be provided. The neutralizing antibody status for ADA positive samples will be provided.

Incidence will be provided for the following ADA response categories:

Pre-existing immunoreactivity is defined as:

An ADA positive response in the assay at baseline with all post first dose ADA results negative, OR an ADA positive response at baseline with all post first dose ADA responses less than 4-fold over baseline titer levels.

Treatment-emergent ADA response is defined as:

A positive response in the ADA assay post first dose, when baseline results are negative or missing.

Treatment-emergent ADA responses are further classified as Persistent, Indeterminate or Transient

A) Persistent Response- defined as a treatment-emergent ADA response with two or more consecutive ADA positive sampling time points, separated by greater than (>) 12-week period (84 days), with no ADA negative samples in between.

B) Indeterminate Response- defined as a treatment-emergent response with only the last collected sample positive in the ADA assay

C) Transient Response - defined as a treatment-emergent response that is not considered persistent OR indeterminate

Treatment-boosted response is defined as:

An ADA positive response in the assay post first dose that is greater-than or equal to 4-fold over baseline titer levels, when baseline results are positive.

Titer values (Titer value category)

- Low (Titer <1000)
- Moderate (1,000 ≤ Titer ≤10,000)
- High (Titer >10,000)

The following summary will be provided based on ADA population:

- Number (%) of participants with pre-existing immunoreactivity
- Number (%) of participants with treatment-emergent ADA
- The summary statistics (including number, median, Q1, Q3, minimum and maximum) of the peak post-baseline titer for participants with treatment-emergent ADA, and participants with persistent, indeterminate and transient ADA response
- Number (%) of participant with transient treatment-emergent ADA
- Number (%) of participants with persistent treatment-emergent ADA
- Number (%) of participants with indeterminate treatment-emergent ADA

- Number (%) of participants with treatment-boosted ADA
- The summary statistics (including number, median, Q1, Q3, minimum and maximum) of the peak post-baseline titer for participants with treatment-boosted ADA
- The summary statistics (including number, mean, SD, median, Q1, Q3, minimum and maximum) of the ratio of peak post-baseline titer to baseline titer for participants with treatment-boosted ADA
- Listing of ADA peak titer levels and neutralizing antibody status
- Number (%) of participants with neutralizing antibody status

Kinetics of treatment-emergent ADA response

Number (%) of participants with treatment-emergent ADA positive response at each visit will be summarized, including titer categories (lower, moderate, and high titer), by each intervention group.

A plot of percentage of participants with treatment-emergent ADA positive response at each visit will be provided by each intervention group.

Association of Immunogenicity with Exposure, Safety and Efficacy

The safety and efficacy analyses mentioned below will be conducted using the following categories:

- ADA positive patients: Patients with treatment-emergent or treatment-boosted response.
- ADA negative patients: Patients with pre-existing immunoreactivity or negative in the ADA assay at all time points.

Association of ADA with PK

Associations between ADA variables (eg, ADA peak titers, neutralizing antibody status, treatment-emergent, persistent, indeterminate and transient response, treatment-boosted) and serum concentration of dupilumab may be explored for each dupilumab dose group. A plot of serum concentration of functional dupilumab versus visit will be provided by ADA classifications for the dupilumab dose group. Individual participant plots of dupilumab concentration according to ADA status will be provided to assess the impact of ADA on PK.

Association of ADA with clinical efficacy endpoints

Associations between the ADA variables (eg, ADA peak titers, neutralizing antibody status, treatment-emergent, persistent and treatment-boosted) and the primary efficacy endpoint may be explored for the dupilumab dosed group.

Association of ADA with clinical safety endpoints

Association of safety versus ADA status may be analyzed in the ADA population. The safety assessment may focus on the following events:

- Severe injection site reactions last longer than 24 hours or serious injection site reactions
- Hypersensitivity reactions (SMQ (20000214) hypersensitivity narrow search confirmed by medical review)
- Anaphylactic reactions (SMQ (20000021) anaphylactic reaction narrow search)

Associations between ADA variables (eg, ADA peak titers, neutralizing antibody status, treatment-emergent, persistent and treatment-boosted) and safety may be explored.

4.8.3 Pharmacodynamic/genomics endpoints

Venous blood samples will be collected at Visit 2 (Week 0), Visit 3 (Week 4), Visit 4 (Week 8), Visit 5 (Week 12), Visit 6 (Week 24), and Visit 7 (Week 36), for measurement of total serum IgE. Total IgE will be measured using validated quantitative methods.

For those participants who consent to the optional pharmacogenetic/pharmacogenomic sample collection section of the ICF, serum/plasma for archival samples for possible future analysis of potential biomarkers of drug response, disease activity, safety, and the type 2 inflammation pathway, and blood samples for exploratory genetic analysis of DNA or RNA will be collected and stored for possible future use. Participation is optional. Participants who do not wish to participate in the genetic research may still participate in the study.

Total IgE will be summarized in the safety population defined as participants who actually received at least 1 dose or part of a dose of the IMP. Baseline values will be the last value collected prior to the first IMP. Descriptive statistics (including number, mean, SD, median, Q1, Q3, min, max) of biomarkers at baseline will be summarized. In addition, for patients who take selected prohibited medications/procedures or rescue medications defined in Table 4, the data after the medications will be censored and then last observation carried forward (LOCF) method will be implemented for missing data imputation in the analysis..

Summary plots (mean +/- standard error of the mean) on values at each visit, absolute changes from baseline and percent changes from baseline will be provided for the total IgE by intervention group and visit.

Exploratory analysis of DNA/RNA will be addressed in a separate document.

4.9 INTERIM ANALYSES

No interim analysis is planned.

A primary database lock will be performed when all randomized participants in this study have completed their 24-week treatment phase. Final analyses in the CSR will be based on this database.

The database will be updated at the end of the study for all participants to include the post-treatment follow-up information and updates for the events previously ongoing at the time of the primary lock. Additional data between this database lock and last participant completing last visit will be summarized in a CSR addendum.

5 SUPPORTING DOCUMENTATION

5.1 APPENDIX 1 LIST OF ABBREVIATIONS

ADA:	anti-drug antibody
AE:	adverse event
AESIs:	adverse events of special interest
ALT:	alanine aminotransferase
ANCOVA:	analysis of covariance
AST:	aspartate aminotransferase
ATC:	anatomic category
CI:	confidence interval
CLcr:	creatinine clearance
CMH:	Cochran-Mantel-Haenszel
CMQ:	customized MedDRA query
CSR:	clinical study report
DLQI:	Dermatology Life Quality Index
DNA:	deoxyribonucleic acid
ECG:	electrocardiogram
eCRF:	electronic case report form
EMA:	European Medicines Agency
EOT:	end of treatment
EQ-5D-5L:	5-level EuroQol 5-dimensional questionnaire
FDA:	Food and Drug Administration
HADS:	hospital anxiety and depression scale
HBc Ab:	hepatitis B core antibody
HBs Ab:	hepatitis B surface antibody
HBs Ag:	hepatitis B surface antigen
HBV:	hepatitis B virus
HCV Ab:	hepatitis C virus antibodies
HLGT:	high level group term
HLT:	high level term
HRQoL:	health-related quality-of-life
IGA PN:	Investigator's global assessment for prurigo nodularis
IMP:	investigational medicinal product
IRT:	interactive response technology
ITT:	intent-to-treat
LLT:	lower-level term
LS:	least squares
MedDRA:	Medical Dictionary for Regulatory Activities
PCSA:	potentially clinically significant abnormality
PGIC:	Participant Global Impression of Change
PGIS:	Global Impression of Severity
PK:	pharmacokinetic

PN:	prurigo nodularis
PT:	preferred term
RNA:	ribonucleic acid
SAE:	serious adverse event
SAP:	statistical analysis plan
SD:	standard deviation
SDG:	standardized drug grouping
SMQ:	standardized MedDRA query
SOC:	system organ class
TEAE:	treatment-emergent adverse event
ULN:	upper limit of normal
WHO-DD:	World Health Organization-Drug Dictionary
WI-NRS:	worst-itch numeric rating scale
WOCF:	worst-observation carried forward

5.2 APPENDIX 2 CHANGES TO PROTOCOL-PLANNED ANALYSES

This SAP for study EFC16460 is based on the Protocol Amendment 1 dated 20-May-2020. There are no major changes to the statistical analysis features in this SAP, this section summarizes major statistical changes in the protocol amendment.

Table 8 - Major statistical changes in protocol amendment(s)

Amendment Number	Approval Date	Changes	Rationale
1	20-May-2020	To add "proportion of participants with Investigator's Global Assessment 0 or 1 score for PN-Stage (IGA PN-S) at Week 24" as another key secondary endpoint	To include a lesion-related key secondary endpoint according to the health authority's recommendations
		To remove the endpoint "Change from baseline in PAS total score at Week 4, Week 8, Week 12, and Week 24" in the exploratory endpoint and modify the exploratory endpoint regarding the healed lesions from PAS questionnaire analysis	To clarify the analysis on efficacy evaluation of dupilumab on skin lesions using a modified prurigo activity score (PAS) 5-item questionnaire
		To break out the secondary endpoints with multiple measuring timepoints into individual endpoints.	To clearly define the timepoints of each endpoint according to health authority's recommendation
		To add the sensitivity analysis for secondary endpoints information, and to separate key secondary endpoints in a different row	To evaluate the robustness of the missing data imputation assumption by sensitivity analyses, and to clarify the endpoints analyses
		To add the covariate "baseline anti-depressant use (yes or no)" to primary and secondary endpoint analyses	To adjust for potential impact of anti-depressant use on the treatment effect in primary and secondary efficacy analyses
		The following wording was added: "Data collected regarding the impact of the COVID-19 or other pandemics, on the participants will be summarized (eg, discontinuation due to COVID-19). Any additional analyses and methods required to investigate the impact of COVID-19 or other pandemics requiring public health emergency on the efficacy (eg, missing data due to COVID-19) and safety will be detailed in the SAP".	To describe alternative temporary mechanism that can be implemented in the study conduct in case of pandemic requiring public health emergency eg, COVID-19

5.3 APPENDIX 3 DEMOGRAPHICS AND BASELINE CHARACTERISTICS, PRIOR OR CONCOMITANT MEDICATIONS/PROCEDURES

Demographics, baseline characteristics, medical surgical history

The following demographics and baseline characteristics, medical and surgical history and disease characteristics at baseline will be summarized using descriptive statistics in the randomized population.

Demographic variables are

- Age in years (quantitative and qualitative variable: 18-<40, 40–<65, 65-<75, and ≥75 years),
- Gender (Male, Female),
- Race (White, Black or African American, Asian, American Indian or Alaska Native, Native Hawaiian or other Pacific Island, unknown),
- Ethnicity (Hispanic or Latino, Not Hispanic or Latino, Unknown)
- Region (Asia: Taiwan, South Korea; East Europe: Hungary; Latin America: Chile; Western Countries: USA, Canada, France, Italy, Portugal, Spain, and UK)
- Territory (North America: USA, Canada; European Union: France, Italy, Portugal, Spain, Hungary, and UK; Rest of World: Taiwan, South Korea, Chile)
- Weight in kg (quantitative and qualitative variable: (<60, ≥60- <90, ≥90 kg)
- BMI in kg/m^2 (quantitative and qualitative variable: <25, ≥25- <30, ≥30 kg/m^2)

Baseline safety and efficacy parameters (apart from those listed above) will be presented along with the safety and efficacy summaries.

Medical (or surgical) history includes all the relevant medical (or surgical) history during the lifetime of the participant.

This information will be coded using the version of MedDRA currently in effect at Sanofi at the time of database lock.

Comorbidity will be summarized separately. The following comorbid diseases will be summarized from electronic case report form (eCRF) pages which were filled in by investigators based on participant reporting.

- Prurigo nodularis (Yes, Ongoing condition)
- Atopic dermatitis (Yes, Ongoing condition)
- Allergic rhinitis history (Yes, Ongoing condition)
- Allergic rhinoconjunctitvitis (Yes, Ongoing condition)
- Asthma history (Yes, Ongoing condition)

- Food allergy history (Yes, Ongoing condition)
- Eosinophilic esophagitis history (Yes, Ongoing condition)

Disease characteristics at baseline

The following baseline disease characteristics will be summarized by intervention group:

- Duration of PN and grouping (years; <3 and ≥3) to be derived as
  - (Year of randomization – Year of first diagnosis of PN) + (month of randomization – month of first diagnosis of PN)/12
- Age of onset of PN (years)
- History of atopy (atopic or non-atopic)
  - Number of mild AD participants under atopic
- Stable use of TCS/TCI (yes or no)
- Baseline WI-NRS score
- Baseline IGA PN-S score (summary and frequency by each score level)
- Baseline IGA PN-A score (summary and frequency by each score level)
- Baseline Pain-NRS score
- Baseline Sleep-NRS score
- Baseline PGIS score
- PAS (number of prurigo lesions in a total and in representative area, and healed prurigo lesions) at baseline
- Baseline Hospital Anxiety and Depression Scale (HADS) total score
  - Baseline Hospital Anxiety score
  - Baseline Depression Scale score
- Baseline Dermatology Life Quality Index (DLQI) score
- Baseline EuroQol five dimensions questionnaire (ED-5D-5L) (single index score and VAS)
- Frequency of alcohol drinking in the past 12 months (never, occasional, at least monthly, at least weekly, at least daily) and number of drinks on a typical day (1 or 2, >2)
- Antidepressant use (yes or no) at baseline
- HIV (positive vs. negative)

Prior or concomitant medications and procedure

All medications will be coded using the World Health Organization-Drug Dictionary (WHO-DD) using the version currently in effect at Sanofi at the time of database lock.

All procedures will be coded to a PT and associated primary SOC using the version of MedDRA currently in effect at Sanofi at the time of database lock.

- Prior medications/procedures are those the participant used prior to first investigational medicinal product (IMP) injection. Prior medications/procedures can be discontinued before first administration or can be ongoing during treatment phase.
- Concomitant medications/procedures are any interventions received by the participant concomitantly to the IMP, from first administration of IMP to last IMP intake + 98 days.
- Post-treatment medications/procedures are those the participant took in the period running from the end of the concomitant medications period up to the end of the study.
- A given medication/procedure can be classified as a prior medication/procedure and/or as a concomitant medication/procedure and/or as post-treatment medication/procedure. If it cannot be determined whether a given medication/procedure was taken prior or concomitantly or post, it will be considered as prior, concomitant, and post-treatment medication/procedure.

The prior and concomitant medications/procedure will be summarized for the randomized population.

Medications will be summarized by intervention group according to the WHO-DD dictionary, considering the first digit of the anatomic category (ATC) class (anatomic category) and the first 3 digits of the ATC class (therapeutic category). All ATC codes corresponding to a medication will be summarized, and participants will be counted once in each ATC category (anatomic or therapeutic) linked to the medication. Therefore participants may be counted several time for the same medication.

The table for prior medications will be sorted by decreasing frequency of ATC followed by all other therapeutic classes based on the overall incidence across intervention groups. In case of equal frequency regarding ATCs (anatomic or therapeutic categories), alphabetical order will be used.

Concomitant medication received during first IMP to last IMP +14 days and concomitant medication received during first IMP to last IMP +98 days will be summarized separately. The tables for concomitant medications will be sorted by decreasing frequency of ATC followed by all other therapeutic classes based on the incidence in the dupilumab group. In case of equal frequency regarding ATCs (anatomic or therapeutic categories), alphabetical order will be used.

Medications will also be summarized by generic name sorted by decreasing frequency based on the incidence in the dupilumab group.

Procedures will be summarized by intervention group by primary SOC (sorted by internationally agreed order) and PT (sorted in alphabetical order), sorting is based on the overall incidence across intervention groups.

Background intervention

Participants will be required to apply moisturizers (emollients) once or twice daily for at least the 7 consecutive days immediately before baseline (Day 1) and continue until Week 36.

The compliance of moisturizers (emollients) used from 7 days before the baseline visit to Week 36 (or end of study), which is defined as the (number of days moisturizers used during the period) / (number of days within the period) x 100%, will be summarized by intervention group.

Similarly, the compliance of moisturizers (emollients) used from the baseline visit to Week 12 and Week 24 will be summarized by intervention group respectively.

In addition, the participants who take TCS/TCI during the study will be summarized by visit.

Prohibited medications/procedures

The concomitant use of the following therapies is prohibited during the entire study. Study treatment will need to be discontinued in participants receiving these treatments:

- Systemic immunosuppressive/immunomodulating drugs (eg, systemic corticosteroids, cyclosporine, mycophenolate-mofetil, interferon gamma, Janus kinase inhibitors, azathioprine, methotrexate, hydroxychloroquine, dapsone, sulfasalazine, colchine, etc)
- Other monoclonal antibodies (which are biological modifiers)
- Phototherapy, including tanning beds
- Naltrexone or other opioid antagonist
- Gabapentin, pregabalin, and thalidomide

The concomitant use of the following therapies is prohibited except if the dose has been stable for at least 3 months prior to screening, but study treatment will not need to be discontinued in participants receiving the treatments listed below. The dose should also remain stable (can be reduced or discontinued if medically indicated) and should not be increased during the study.

- Paroxetine, fluvoxamine, or other SSRIs
- SNRIs
- Amitriptyline or other tricyclic or tetracyclic antidepressants

The concomitant use of the following therapies is also prohibited during the entire study, but study treatment will not need to be discontinued in participants receiving the treatments listed below:

- Intralesional corticosteroid injections and cryotherapy
- Sedating antihistamine
- Non-sedating antihistamine if used specifically for the treatment of itch secondary to AD or PN

The number and percentage of participants who take the prohibited medications/procedures will be provided. In addition, the time (week) of first prohibited medication/procedure taken will also be analyzed using Kaplan-Meier method.

Rescue medications

The following rescue medications may be used:

- Dermatological preparations of high potency or superpotent TCS and TCI.

If medically necessary (i.e., to control intolerable PN symptoms), rescue treatment for PN may be provided to study participants at the discretion of the Investigator.

Although the use of rescue medications is allowed at any time during the study, the use of rescue medications should be delayed, if possible, for at least 14 days following the initiation of the investigational treatment. The date and time of rescue medication administration as well as the name and dosage regimen of the rescue medication must be recorded in the eCRF.

- For the purpose of the efficacy responder analysis, a pre-specified algorithm will be used to classify rescue. In addition, a blinded review of all post-baseline medications to adjudicate rescue treatment, based on medical judgment, will be performed to adjudicate rescue. Participants who receive rescue treatment as per this adjudication during the study will be considered treatment failures.

The rescue medications will be analyzed by the same method as that used for prohibited medications/procedures.

5.4 APPENDIX 4 DATA HANDLING CONVENTIONS

Demographic formulas

Age of onset of PN is calculated as:

Year of PN diagnosis – Year of birth

BMI is calculated as:

$$\text{Weight in kg} / (\text{height}^2 \text{ in meters})$$

Renal function formulas

For adults, creatinine clearance (CLcr) value will be derived using the equation of Cockroft and Gault:

$$\text{CLcr (ml/min)} = (140 - \text{age}) \times \text{weight (kg)} \times (1 - 0.15 \times \text{sex (0-M, 1-F)}) / (0.814 \times \text{creatinine} (\mu\text{mol/l}))$$

CLcr will be calculated using the last weight measurement on or before the visit of the creatinine measurement and age at the lab sampling day. Here age is calculated as following:

Age = age collected at screening + integer part of (lab sampling analysis day/365.25)

Analysis windows for time points

Efficacy assessment

For the efficacy assessment, the reference date for the derivation of relative days of events or findings will be the randomization day. If a participant receives IMP prior to the randomization by mistake, the reference date of efficacy assessment will be the date of the first IMP administration for that participant.

For daily eDiary data (WI-NRS, Pain-NRS, Sleep-NRS), all available values of daily measurements will be assigned to each week window according to Table 9, and then weekly average score will be calculated. Randomization day is used as the reference day (Day 1).

Table 9 - Time window for eDiary efficacy variables

Time	Target day	Day range for calculating weekly score
Baseline (Week 0)	1	-7- <1
Week 1	8	1-11
Week 2	15	12-18
Week 3	22	19-25
Week 4	29	26-32
Week 5	36	33-39
Week 6	43	40-46
Week 7	50	47-53
Week 8	57	54-60
Week 9	64	61-67
Week 10	71	68-74
Week 11	78	75-81
Week 12	85	82-88
Week 13	92	89-95
Week 14	99	96-102
Week 15	106	103-109
Week 16	113	110-116
Week 17	120	117-123
Week 18	127	124-130
Week 19	134	131-137

Time	Target day	Day range for calculating weekly score
Week 20	141	138-144
Week 21	148	145-151
Week 22	155	152-158
Week 23	162	159-165
Week 24	169	166-172
Week 25	176	173-179
Week 26	183	180-186
Week 27	190	187-193
Week 28	197	194-200
Week 29	204	201-207
Week 30	211	208-214
Week 31	218	215-221
Week 32	225	222-228
Week 33	232	229-235
Week 34	239	236-242
Week 35	246	243-249
Week 36	253	250-256

For other efficacy variables, all available values of scheduled measurements will be assigned to the appropriate visit window according to Table 10. In the event of multiple measurements of the same test in the same window, the one closest to the targeted visit date will be used for the by-visit summary. If they are at the same number of days away from the target day, the latest one will be used.

Table 10 - Time window for efficacy variables

Visit	Target Day	Time windows for				
		**DLQI, IGA PN-A/PN-S**	**HADS, EQ-5D-5L, Missed school/work days**	**PGIS**	**PGIC**	**PAS**
Visit 1 (Week -4 to -2)	<1			<-14		<-14
Visit 2 (Week 0)	1	1^-	1^-	-14-1^-		-14-1^-
Visit 3 (Week 4)	29	1^+-42		1^+-42	1^+-42	1^+-42
Visit 4 (Week 8)	57	43-70		43-70	43-70	43-70
Visit 5 (Week 12)	85	71-126	1^+-126	71-126	71-126	71-126
Visit 6 (Week 24)	169	127-210	127-210	>126	>126	127-210
Visit 7 (Week 36)	253	>210	>210			>210

1-: up to randomization and before 1st dose date/time; 1+: after randomization or 1st dose date/time

Safety assessment

For the safety assessment, the reference date for the derivation of relative days of events or findings will be the date of first IMP administration. Selected safety variables will be summarized by the analysis window define in Table 11 for the by visit descriptive analysis. All available values from central lab will be assigned to the appropriate visit window. In the event of multiple measurements of the same test in the same window, the one closest to the targeted visit date will be used for the by-visit summary. If they are at the same number of days away from the target day, the latest one will be used. For procedures planned on Visit 2, if it is done on the same date as the first IMP injection but the performance time is missing, it will belong to Visit 2 time window.

Table 11 - Time window for safety endpoints

		Time windows for							
Visit	Target Day	Vital signs	Hematology, biochemistry	Urinalysis	CD4 T cell count and HIV test	Serum Pregnancy test	Urine Pregnancy test	Physical examination	ECG
Visit 1 (Week -4 to -2)	<1	<-14	<-14	<-14	1^{-}	<-14		<-14	1^{-}
Visit 2 (Week 0)	1	$-14\text{-}1^{-}$	$-14\text{-}1^{-}$	$-14\text{-}1^{-}$			$-14\text{-}1^{-}$	$-14\text{-}1^{-}$	
Visit 3 (Week 4)	29	1^{+}-42	1^{+}-42	1^{+}-42			1^{+}-42		
Visit 4 (Week 8)	57	43-70	43-70	43-70			43-70		
Visit 5 (Week 12)	85	71-126	71-126	71-126			71-126		
Visit 6 (Week 24)	169	127-210	127-210	>126	1^{+}		127-210	1^{+}-210	1^{+}
Visit 7 (Week 36)	253	>210	>210				>210	>210	

1-: up to 1st dose date/time; 1+: after 1st dose date/time;

Pharmacokinetics/pharmacodynamics assessment

For the pharmacokinetics/pharmacodynamics variables summary, the reference date for the derivation of relative days of measurements will be the date of first IMP administration if the participant is treated with study treatment, or the randomization date if the participant is not treated. Pharmacokinetics /pharmacodynamics variables will be summarized by the analysis window defined in Table 12 for the by visit descriptive analyses. All available values of measurements will be assigned to the appropriate visit window. In the event of multiple measurements of the same test in the same window, the one closest to the targeted visit date will be used for the by-visit summary. If they are at the same distance to the target day, the latest one will be used. For procedures planned on Visit 2, if it is done on the same date as the first IMP injection but the performance time is missing, it will belong to Visit 2 time window.

Table 12 - Time window for pharmacokinetics/pharmacodynamics variables

Visit	Target day	Time windows for		
		Serum dupilumab concentration	Anti-drug antibodies	Total IgE
Visit 1 (Week -4)	<1			
Visit 2 (Week 0)	1	1^{-}	1^{-}	1^{-}
Visit 3 (Week 4)	29	1^{+}-42		1^{+}-42
Visit 4 (Week 8)	57	43-70		43-70
Visit 5 (Week 12)	85	71-126	1^{+}-126	71-126
Visit 6 (Week 24)	169	127-210	127-210	127-210
Visit 7(Week 36)	253	>210	>210	>210

1-: up to 1st dose date/time or randomization if participant is not treated; 1+: after 1st dose date/time or randomization date if participant is not treated;

Unscheduled visits

Unscheduled visit measurements of laboratory data, vital signs, and ECG will be used for computation of baseline, the last on-treatment value, analysis according to PCSAs, and the shift summaries for safety. They will also be included in the by-visit summaries if they are re-allocated to scheduled visits.

5.5 APPENDIX 5 SAMPLE SAS CODE

The multiple imputation and analysis model for the primary analysis of change from baseline in WI-NRS at Week 12 will be built with the following sample SAS code.

1. 40 datasets with a monotone missing pattern will be obtained, induced by Markov Chain Monte Carlo (MCMC) method on participants who have not taken selected prohibited medications and/or rescue medications or have discontinued study treatment due to lack of efficacy prior to Week 12.

```
proc mi data=dat_etd seed=16461 nimpute=40 out=dat_mc;
    mcmc impute=monotone;
    var atopicyn stableyn region antidblyn trt01p winrsbl chg1winrs …
    chg24winrs;
run;
```

2. For each of the imputed dataset with monotone missing pattern in step 1, the remaining missing data will be imputed using the regression method for the monotone pattern with adjustment for covariates including intervention groups, documented history of atopy (atopic or non-atopic), stable use of TCS/TCI (yes or no), region, baseline anti-depressant use (yes or no), and baseline value of the response variable.

```
proc mi data=dat_mc nimpute=1 seed=16461 out=dat_mi;
    by _imputation_;
    class atopicyn stableyn region antidblyn trt01p;
    monotone method=reg;
    var atopicyn stableyn region antidblyn trt01p winrsbl chg1winrs …
    chg24winrs;
run;
```

3. Each of the 40 imputed datasets will be merged with the one dataset imputed by WOCF approach, and then be analyzed using the main statistical model. These 40 imputed datasets will be saved.

```
%macro w1;
     %do i=1%to 40;
     data wocf&i.;
     set wocf;
     _imputation_=&i.;
     run;
     %end;
     data wocf_all;
     set %do j=1 %to 40; wocf&j. %end;;
     run;
%mend w1;
```

```
%w1

data dat_imp;
     set dat_mi  wocf_all;
Run;

proc sort data=dat_imp;
     by _imputation_;
run;

proc glm data= dat_imp;
     by _imputation_;
     class atopicyn stableyn region antidblyn trt01p;
     model  chg24winrs = atopicyn stableyn region antidblyn
                trt01p winrsbl;
     lsmeans trt01p / stderr;
     estimate 'Diff Dupilumab vs Placebo' trt01p -1 1;
     ods output LSMeans=implsmeans Estimates=implsmeandiff;
run;
```

4. Applying Rubin's rule to combine analysis results (point estimates and standard errors) from 40 imputations using PROC MIANALYZE for the LS means and difference in LS means between dupilumab and placebo. Sample code:

```
proc sort data=implsMeans; by trt01pn _imputation_;run;

proc mianalyze data= implsmeans;
     by trt01pn;
     modeleffects lsmean;
     stderr stderr;
     ods output ParameterEstimates=lsmeans;
run;

proc mianalyze data=implsmeandiff;
     modeleffects estimate;
     stderr stderr;
     ods output ParameterEstimates=lsmeandiff;

run;
```

5.6 APPENDIX 6 SELECTION CRITERIA FOR AE/MEDICATION GROUPINGS

Table 13 - List of PTs or medications for CMQs/CDGs

Grouping	Preferred Term/Medication Code	Preferred Term/Medication
Conjunctivitis	10001257	Adenoviral conjunctivitis
Conjunctivitis	10010725	Conjunctival irritation
Conjunctivitis	10010726	Conjunctival oedema
Conjunctivitis	10010736	Conjunctival ulcer
Conjunctivitis	10010741	Conjunctivitis
Conjunctivitis	10010744	Conjunctivitis allergic
Conjunctivitis	10010745	Conjunctivitis chlamydial
Conjunctivitis	10010749	Conjunctivitis gonococcal neonatal
Conjunctivitis	10010754	Conjunctivitis tuberculous
Conjunctivitis	10010755	Conjunctivitis viral
Conjunctivitis	10018258	Giant papillary conjunctivitis
Conjunctivitis	10021629	Inclusion conjunctivitis
Conjunctivitis	10030861	Ophthalmia neonatorum
Conjunctivitis	10048908	Seasonal allergy
Conjunctivitis	10049458	Herpes simplex virus conjunctivitis neonatal
Conjunctivitis	10051625	Conjunctival hyperaemia
Conjunctivitis	10053991	Inclusion conjunctivitis neonatal
Conjunctivitis	10061784	Conjunctivitis bacterial
Conjunctivitis	10062889	Pingueculitis
Conjunctivitis	10063669	Photoelectric conjunctivitis
Conjunctivitis	10067317	Oculorespiratory syndrome
Conjunctivitis	10067817	Acute haemorrhagic conjunctivitis
Conjunctivitis	10069166	Blebitis
Conjunctivitis	10071570	Ligneous conjunctivitis
Conjunctivitis	10074701	Noninfective conjunctivitis
Conjunctivitis	10075264	Oculoglandular syndrome
Conjunctivitis	10080825	Conjunctivitis fungal
Conjunctivitis	10084034	Conjunctival suffusion
Intravenous immunoglobulin therapy	CAS 8000012671	IMMUNOGLOBULIN HUMAN NORMAL
Intravenous immunoglobulin therapy	CAS 8000050682	IMMUNOGLOBULIN, PORCINE
Intravenous immunoglobulin therapy	CAS 8000056919	IMMUNOGLOBULIN G HUMAN

Grouping	Preferred Term/Medication Code	Preferred Term/Medication
Intravenous immunoglobulin therapy	CAS 8600000563	IMMUNOGLOBULINS NOS
Intravenous immunoglobulin therapy	CAS 8600001670	IMMUNOGLOBULIN HUMAN NORMAL SLRA
Intravenous immunoglobulin therapy	CAS 8600001671	IMMUNOGLOBULIN HUMAN NORMAL IFAS
Intravenous immunoglobulin therapy	RECNO 900708	OTHER IMMUNOGLOBULINS
Intravenous immunoglobulin therapy	RECNO 900722	IMMUNE SERA AND IMMUNOGLOBULINS
Intravenous immunoglobulin therapy	RECNO 900728	IMMUNOGLOBULINS
Intravenous immunoglobulin therapy	RECNO 900914	SPECIFIC IMMUNOGLOBULINS
Intravenous immunoglobulin therapy	RECNO 901112	IMMUNOGLOBULINS, NORMAL HUMAN

Abbreviations: CAS : Chemical Abstract Service Registry Number RECNO : Drug Record Number

5.7 APPENDIX 7 MEDICATION/PROCEDURE AJUDICATION ALGORITHM

Algorithm for determining whether treatment with following medications or procedures constitutes treatment failure resulting in setting data as non-responder after taking medication or undergoing procedure in the main statistical analysis

1. Not required to adjudicate post-baseline medications (WHODD-coded) or procedures (CMQ-coded) as these will be considered treatment failures if used at any time[1].

   a) Always considered treatment failure

   - ATC2 = CORTICOSTEROIDS FOR SYSTEMIC USE
   - ATC2=IMMUNOSUPPRESSANTS (systemic, e.g. oral or parenteral route)
   - Preferred Drug Name = Ciclosporin
   - Preferred Drug Name = Methotrexate
   - Preferred Drug Name = Mycophenolate sodium
   - Preferred Drug Name = Mycophenolic acid
   - Preferred Drug Name = Azathioprine
   - Preferred Drug Name = Gabapentin
   - Preferred Drug Name = Pregabalin
   - Preferred Drug Name = Thalidomide
   - Preferred Drug Name = Lenalidomide
   - Preferred Drug Name = Baricitinib
   - Preferred Drug Name = Ruxolitinib
   - Preferred Drug Name = Tofacitinib
   - Preferred Drug Name = AbrocitinibPreferred Drug Name = Delgocitinib
   - Preferred Drug Name = Nalbuphine
   - Preferred Drug Name = Naltrexone
   - Preferred Drug Name = Naloxone
   - Preferred Drug Name = Vixarelimab
   - Preferred Drug Name = Nemolizumab
   - Preferred Drug Name = Serlopitant
   - Preferred Drug Name = Aprepitant
   - CMQb HLT Phototherapies
   - CMQ Tanning_single PT

b) Never considered treatment failure:

- ATC2 = EMOLLIENTS AND PROTECTIVES
- ATC2 = GENERAL NUTRIENTS
- ATC2 = VITAMINS
- ATC2 = ANTIVIRALS FOR SYSTEMIC USE
- ATC2 = ANTIFUNGALS FOR DERMATOLOGICAL USE
- ATC2 = ANTISEPTICS AND DISINFECTANTS
- ATC2 = ANTIBIOTICS AND CHEMOTHERAPEUTICS FOR DERMATOLOGICAL USE
- ATC2 = ANTI-ACNE PREPARATIONS, excluding D10AA Corticosteroids, combinations for treatment of acne
- ATC2 = OPHTHALMOLOGICALS
- ATC1 = ANTIINFECTIVES FOR SYSTEMIC USE, excluding D07C Corticosteroids, combinations with antibiotics
- ATC1 = BLOOD AND BLOOD FORMING ORGANS
- ATC1 = ALIMENTARY TRACT AND METABOLISM
- ATC1 = MUSCULO-SKELETAL SYSTEM, excluding M01BA Antiinflammatory/antirheumatic agents in combination with corticosteroids
- ATC2 = COUGH AND COLD PREPARATIONS
- ATC2= PSYCHOLEPTICS, excluding N05BB Diphenylmethane derivatives and N05C HYPNOTICS AND SEDATIVES

1 A blinded review of all post-baseline medications or procedures to adjudicate whether treatment constitutes treatment failure, based on medical judgment, may be performed in addition. A listing of treatments or procedures classified as treatment failure in a manner inconsistent with the classification under #1 will be provided, along with supporting rationale.

2. **Require to adjudicate medications or procedures that may constitute treatment failure**

- All other medications and procedures listed in the protocol and Table 4 of the SAP (not noted in 1. above) given for indications consistent with PN[2]
- Considerations in determining treatment failure include the criteria already set forth in Table 4 of the SAP in addition to the type of medication or procedure, indication, dose, route of administration, timing, frequency and the potential impact of the use of the prohibited medications/procedures and rescue medications on the primary and key secondary efficacy endpoints.

2 Below is a list of indications consistent with PN based on PT level from concomitant medication/procedure data using MedDRA dictionary

System Organ Class	High Level Term	Preferred Term	Preferred Term Code
Skin and subcutaneous tissue disorders	Dermatitis and eczema	Neurodermatitis	10029263
Infections and infestations	Bacterial infections NEC	Eczema impetiginous	10051890
Infections and infestations	Skin structures and soft tissue infections	Dermatitis infected	10012470
Infections and infestations	Skin structures and soft tissue infections	Eczema infected	10014199
Skin and subcutaneous tissue disorders	Dermatitis and eczema	Dermatitis	10012431
Skin and subcutaneous tissue disorders	Dermatitis and eczema	Dermatitis atopic	10012438
Skin and subcutaneous tissue disorders	Dermatitis and eczema	Eczema	10014184

6 REFERENCES

1. Carpenter JR, Roger JH, Kenward MG. Analysis of longitudinal trials with protocol deviation: a framework for relevant, accessible assumptions, and inference via multiple imputation. J Biopharm Stat. 2013;23(6):1352-71.

Signature Page for VV-CLIN-0602730 v1.0
efc16460-16-1-9-sap

Approve & eSign	[redacted]

Approve & eSign	[redacted]

AMENDED CLINICAL TRIAL PROTOCOL 01

**Protocol title:**	**A randomized, double blind, placebo-controlled, multi-center, parallel group study to evaluate the efficacy and safety of dupilumab in patients with prurigo nodularis who are inadequately controlled on topical prescription therapies or when those therapies are not advisable**
**Protocol number:**	**EFC16460**
**Amendment number:**	**01**
**Compound number (INN/Trademark):**	**SAR231893/REGN668 dupilumab/Dupixent**
**Study phase:**	**Phase 3**
**Short title:**	**Study of dupilumab for the treatment of patients with prurigo nodularis, inadequately controlled on topical prescription therapies or when those therapies are not advisable**<br>**LIBERTY-PN PRIME2**
**Sponsor name:**	
**Legal registered address:**	
**Monitoring Team's Representative Name and Contact Information**	

Regulatory agency identifier number(s):

IND:	IND107969
EudraCT:	2019-003801-90
NCT:	NCT04202679
WHO:	U1111-1241-8174
Other:	Not applicable

Approval Date: 20-May-2020 Total number of pages: 106

Any and all information presented in this document shall be treated as confidential and shall remain the exclusive property of Sanofi (or any of its affiliated companies). The use of such confidential information must be restricted to the recipient for the agreed purpose and must not be disclosed, published or otherwise communicated to any unauthorized persons, for any reason, in any form whatsoever without the prior written consent of Sanofi (or the concerned affiliated company); 'affiliated company' means any corporation, partnership or other entity which at the date of communication or afterwards (i) controls directly or indirectly Sanofi, (ii) is directly or indirectly controlled by Sanofi, with 'control' meaning direct or indirect ownership of more than 50% of the capital stock or the voting rights in such corporation, partnership or other entity

PROTOCOL AMENDMENT SUMMARY OF CHANGES

DOCUMENT HISTORY

Document	Country/countries impacted by amendment	Date, version
Amended Clinical Trial Protocol 01	All	20 May 2020, version 1 (electronic 1.0)
Original Protocol		30 October 2019, version 1 (electronic 2.0)

Amended protocol 01 (20 May 2020)

This amended protocol (amendment 01) is considered to be substantial based on the criteria set forth in Article 10(a) of Directive 2001/20/EC of the European Parliament and the Council of the European Union.

OVERALL RATIONALE FOR THE AMENDMENT

The proportion of participants with Investigator's Global Assessment 0 or 1 score for PN-Stage (IGA PN-S) at Week 24 has been indicated as an additional key secondary endpoint. This is to include a lesion-related key secondary endpoint based on the recommendations from health authorities.

Protocol amendment summary of changes table

Section # and Name	Description of Change	Brief Rationale
1.1 Synopsis: Objectives and endpoints<br>3 Objectives and Endpoints<br>3.1 Appropriateness of measurements<br>9.4.1 Efficacy analyses	To add "proportion of participants with Investigator's Global Assessment 0 or 1 score for PN-Stage (IGA PN-S) at Week 24" as another key secondary endpoint	To include a lesion-related key secondary endpoint according to the health authority's recommendations
1.3 Schedule of activities	To correct rescue medication use start date to Day 1 (Visit 2)	To correct an error on the rescue medication use
3 Objectives and Endpoints	To remove the endpoint "Change from baseline in PAS total score at Week 4, Week 8, Week 12, and Week 24" in the exploratory endpoint and modify the exploratory endpoint regarding the healed lesions from PAS questionnaire analysis	To clarify the analysis on efficacy evaluation of dupilumab on skin lesions using a modified prurigo activity score (PAS) 5-item questionnaire
1.1 Synopsis (Objectives and endpoints)<br>3 Objectives and Endpoints<br>9.4.1 Efficacy analyses	To break out the secondary endpoints with multiple measuring timepoints into individual endpoints	To clearly define the timepoints of each endpoint according to health authority's recommendation

Section # and Name	Description of Change	Brief Rationale
5.1 Inclusion Criteria	Change of I05 “medium-to-high potency TCS” to “medium-to-superpotent TCS”	To enroll patients on medium to superpotent topical corticosteroids (TCS) according to health authority’s recommendations
1.1 Synopsis: Noninvestigational medicinal products<br>5.1 Inclusion Criteria<br>6.1.1 Noninvestigational medicinal products	Change of topical emollient (moisturizer) application in Synopsis and I06 from “once or twice daily for at least the 7 consecutive days immediately before Day 1” to “once or twice daily for at least 5 out of the 7 consecutive days immediately before Day 1”	To allow some flexibility in adherence to the moisturizer usage by the patient during screening
5.2 Exclusion Criteria	To update E04 to “within 6 months before the screening visit, or documented diagnosis of moderate to severe AD from screening visit to randomization visit”	To specify the exclusion criteria of patients with documented atopic dermatitis severity moderate to severe
5.2 Exclusion Criteria<br>1.3 Schedule of Activities<br>10.2 Appendix 2: Clinical laboratory tests	To update E05 “hemoglobin A1c ≥9%” to “hemoglobin A1c ≥9% according to the laboratory results within 3 months before screening visit”	To clarify the exclusion criteria for patients with diabetes history is according to the hemoglobin A1c results within 3 months before screening visit
5.2 Exclusion Criteria	To update E10 from “within 2 weeks before the screening visit” to “within 2 weeks before screening visit and during the screening period”	To exclude the patients with active chronic or acute infection (except HIV infection) requiring treatment with systemic antibiotics, antivirals, antiprotozoals, or antifungals during screening period
5.2 Exclusion Criteria	To modify E18 to allow prurigo nodularis (PN) patients to use stable dose of some anti-depressant medicines for at least 3 months prior to screening	To enroll PN patients with stable dose of anti-depressant
5.2 Exclusion Criteria	To add menthol and polidocanol as one of the moisturizer additives in E20	To clarify that the patients who initiate treatment with moisturizers containing menthol and polidocanol will be excluded
5.2 Exclusion Criteria<br>6.1.1 Noninvestigational medicinal products	To add to E22 a stable regimen of TCS/TCI "maintain same medicine, same dose from 2 weeks prior to screening visit"	To clarify the definition of stable regimen of TCS/TCI
5.2 Exclusion Criteria	To add “treated in the past with dupilumab” in E25	To exclude the patients previously treated with dupilumab
5.2 Exclusion Criteria	To describe the participants with or without history of HIV infection in E26	To clarify the exclusion criteria about HIV infection and HIV viral load, and that the CD4+ test will only be done in the patient with existing HIV history
5.2 Exclusion Criteria<br>1.3 Schedule of Activities: footnote c<br>10.2 Appendix 2: Clinical laboratory tests: Table 9 footnote c	To clarify to E27 the positive HBc Ab and HCV Ab confirmation method	To clarify that the positive HBc Ab and HCV Ab will be confirmed by HBV DNA and HCV RNA, respectively

Section # and Name	Description of Change	Brief Rationale
6.1.1 Noninvestigational medicinal products	To clarify the use of noninvestigational medicinal products	To prohibit topical medications that have anti-itch properties
6.5 Concomitant Therapy	To clarify the permitted and prohibited concomitant medications	To clarify the requirement of antihistamine used concomitantly, and antidepressant dose should also remain stable (can be reduced or discontinued if medically indicated), but should not be initiated or increased throughout the study
6.5.1 Rescue Medicine	To clarify the use of rescue medicine	To add criteria for rescue therapy
6.5.1 Rescue Medicine	To add that a pre-specified algorithm will be used to classify rescue and a blinded review of all post-baseline medications, based on medical judgment, will be performed to adjudicate rescue	To clarify the review of adjudicate rescue treatment
7.1.1 Definitive discontinuation<br>7.1.2 Temporary discontinuation	To clarify the treatment discontinuation criteria regarding the missing doses	To clarify that patients with missing more than 2 consecutive IMP doses should be permanently withdrawn from the study treatment
8 Study assessments and procedures	The following wording was added: "In light of the public health emergency related to Coronavirus disease 2019 (COVID-19) (or in case of any other pandemic requiring public health emergency), the continuity of clinical study conduct and oversight may require implementation of temporary or alternative mechanisms eg, phone contact, virtual visits, online meetings, use of local clinic or laboratory locations, and home visits by skilled staff. Implementation of such mechanisms may differ country by country, depending on country regulations and local business continuity plans. Additionally, no waivers to deviate from protocol enrollment criteria due to COVID-19 (or any other pandemic) will be granted. All temporary mechanisms utilized, and deviations from planned study procedures are to be documented as being related to COVID-19 (or any other pandemic) and will remain in effect only for the duration of the public health emergency."	To describe alternative temporary mechanism that can be implemented in the study conduct in case of pandemic requiring public health emergency eg, COVID-19
8.3 Adverse events and serious adverse events	To update the adverse event of special interest (AESI) "any type of conjunctivitis or blepharitis (severe or serious)" to "any severe type of conjunctivitis or blepharitis"	To update the AESI list to harmonize with the most recent list
9.4.1 Efficacy analyses	To add the sensitivity analysis for secondary endpoints information, and to separate key secondary endpoints in a different row	To evaluate the robustness of the missing data imputation assumption by sensitivity analyses, and to clarify the endpoints analyses

Section # and Name	Description of Change	Brief Rationale
9.4.1 Efficacy analyses<br>1.1 Synopsis (Statistical considerations)	To add the covariate "baseline anti-depressant use (yes or no)" to primary and secondary endpoint analyses	To adjust for potential impact of anti-depressant use on the treatment effect in primary and secondary efficacy analyses
9.4.3 Other analyses	The following wording was added: "Data collected regarding the impact of the COVID-19 or other pandemics, on the patients will be summarized (eg, discontinuation due to COVID-19). Any additional analyses and methods required to investigate the impact of COVID-19 or other pandemics requiring public health emergency on the efficacy (eg, missing data due to COVID-19) and safety will be detailed in the SAP".	To describe alternative temporary mechanism that can be implemented in the study conduct in case of pandemic requiring public health emergency eg COVID-19
9.5.2 Unblinding plan	To update the unblinding plan to "Unblinding plan is not applicable for this study"	Unblinding plan is not applicable for this study.
1.3 Schedule of activities<br>10.2 Appendix 2: Clinical laboratory tests	To remove the description about false positivity	To clarify that the testing will be performed if considered that the patient is immune after a natural infection
1.3 Schedule of activities<br>10.2 Appendix 2: Clinical laboratory tests	To add "tetranor PGDM" into urinalysis	Tetranor PGDM will be tested in urinalysis since it is a Type 2 inflammation biomarker.
10.3 Appendix 3: Adverse events: Definitions and procedures for recording, evaluating, follow-up, and reporting	To update the AE and SAE recording	To minimize the transfer of personal data to be compliant with the General Data Protection Regulation (GDPR) and harmonize processes across countries.
10.4 Appendix 4: Contraceptive guidance and collection of pregnancy information	To remove the "Acceptable methods" in contraception guidance	To clarify the contraceptive methods can be used during the study.
10.7.2 Investigator's global assessment of prurigo nodularis (IGA PN)<br>10.7.3 Prurigo activity score (PAS)	To add the copyright information	To add copyright provided by the author for the IGA PN, PAS baseline and PAS Follow-up versions
	Minor editorial and formatting changes were done throughout the document.	To correct minor errors or formatting issues.

TABLE OF CONTENTS

AMENDED CLINICAL TRIAL PROTOCOL 01 1

PROTOCOL AMENDMENT SUMMARY OF CHANGES 2

TABLE OF CONTENTS 6

LIST OF TABLES 10

1 PROTOCOL SUMMARY 11

1.1 SYNOPSIS 11

1.2 SCHEMA 17

1.3 SCHEDULE OF ACTIVITIES (SOA) 18

2 INTRODUCTION 23

2.1 STUDY RATIONALE 23

2.2 BACKGROUND 23

2.3 BENEFIT/RISK ASSESSMENT 25

3 OBJECTIVES AND ENDPOINTS 28

3.1 APPROPRIATENESS OF MEASUREMENTS 29

4 STUDY DESIGN 31

4.1 OVERALL DESIGN 31

4.2 SCIENTIFIC RATIONALE FOR STUDY DESIGN 31

4.3 JUSTIFICATION FOR DOSE 32

4.4 END OF STUDY DEFINITION 32

5 STUDY POPULATION 33

5.1 INCLUSION CRITERIA 33

5.2 EXCLUSION CRITERIA 35

5.3 LIFESTYLE CONSIDERATIONS 39

5.4 SCREEN FAILURES 39

6 STUDY INTERVENTION 40

6.1 STUDY INTERVENTION(S) ADMINISTERED 40

6.1.1 Noninvestigational medicinal products 41

6.2 PREPARATION/HANDLING/STORAGE/ACCOUNTABILITY....................42
6.2.1 Storage and handling....................42
6.2.2 Responsibilities....................42
6.3 MEASURES TO MINIMIZE BIAS: RANDOMIZATION AND BLINDING....................42
6.4 STUDY INTERVENTION COMPLIANCE....................44
6.5 CONCOMITANT THERAPY....................44
6.5.1 Rescue medicine....................45
6.6 DOSE MODIFICATION....................46
6.7 INTERVENTION AFTER THE END OF THE STUDY....................46
7 DISCONTINUATION OF STUDY INTERVENTION AND PARTICIPANT DISCONTINUATION/WITHDRAWAL....................47
7.1 DISCONTINUATION OF STUDY INTERVENTION....................47
7.1.1 Definitive discontinuation....................47
7.1.2 Temporary discontinuation....................48
7.1.2.1 Rechallenge....................49
7.2 PARTICIPANT DISCONTINUATION/WITHDRAWAL FROM THE STUDY....................49
7.3 LOST TO FOLLOW UP....................50
8 STUDY ASSESSMENTS AND PROCEDURES....................51
8.1 EFFICACY ASSESSMENTS....................51
8.1.1 Worst-itch numeric rating scale....................52
8.1.2 Investigator's global assessment for prurigo nodularis....................52
8.1.3 Prurigo activity score....................52
8.1.4 Dermatology life quality index....................53
8.1.5 Pain and sleep numeric rating scales....................53
8.1.6 Hospital anxiety and depression scale....................53
8.1.7 Patient Global Impression of Change of disease and Patient Global Impression of Severity....................54
8.1.8 Euroqol 5 dimensions questionnaire....................54
8.1.9 Missed school/work days....................54
8.2 SAFETY ASSESSMENTS....................55
8.2.1 Physical examinations....................55
8.2.2 Vital signs....................55
8.2.3 Electrocardiograms....................55
8.2.4 Clinical safety laboratory assessments....................55

8.3 ADVERSE EVENTS AND SERIOUS ADVERSE EVENTS....56
8.3.1 Time period and frequency for collecting AE and SAE information....57
8.3.2 Method of detecting AEs and SAEs....58
8.3.3 Follow-up of AEs and SAEs....58
8.3.4 Regulatory reporting requirements for SAEs....58
8.3.5 Pregnancy....58
8.3.6 Guidelines for reporting product complaints....59
8.4 TREATMENT OF OVERDOSE....59
8.5 PHARMACOKINETICS....59
8.5.1 Systemic drug concentration and antidrug antibodies....59
8.5.1.1 Sampling time....59
8.5.1.2 Handling procedures....60
8.5.1.3 Bioanalytic method....60
8.6 PHARMACODYNAMICS....61
8.7 GENETICS....61
8.8 BIOMARKERS....61
8.9 MEDICAL RESOURCE UTILIZATION AND HEALTH ECONOMICS....61
9 STATISTICAL CONSIDERATIONS....62
9.1 STATISTICAL HYPOTHESES....62
9.2 SAMPLE SIZE DETERMINATION....62
9.3 POPULATIONS FOR ANALYSES....63
9.4 STATISTICAL ANALYSES....63
9.4.1 Efficacy analyses....63
9.4.2 Safety analyses....65
9.4.3 Other analyses....66
9.5 INTERIM ANALYSES....66
9.5.1 Data Monitoring Committee (DMC)....67
9.5.2 Unblinding plan....67
10 SUPPORTING DOCUMENTATION AND OPERATIONAL CONSIDERATIONS....68
10.1 APPENDIX 1: REGULATORY, ETHICAL, AND STUDY OVERSIGHT CONSIDERATIONS....68
10.1.1 Regulatory and Ethical Considerations....68
10.1.2 Financial disclosure....68
10.1.3 Informed consent process....69

10.1.4 Data protection 69
10.1.5 Committees structure 70
10.1.6 Dissemination of clinical study data 70
10.1.7 Data quality assurance 71
10.1.8 Source documents 71
10.1.9 Study and site closure 72
10.1.10 Publication policy 72

10.2 APPENDIX 2: CLINICAL LABORATORY TESTS 73

10.3 APPENDIX 3: ADVERSE EVENTS: DEFINITIONS AND PROCEDURES FOR RECORDING, EVALUATING, FOLLOW-UP, AND REPORTING 75

10.4 APPENDIX 4: CONTRACEPTIVE GUIDANCE AND COLLECTION OF PREGNANCY INFORMATION 79

10.5 APPENDIX 5: GENETICS 81

10.6 APPENDIX 6: LIVER AND OTHER SAFETY: SUGGESTED ACTIONS AND FOLLOW-UP ASSESSMENTS 83

10.7 APPENDIX 7: CLINICIANS-REPORTED OUTCOMES AND PATIENT-REPORTED OUTCOMES 86
10.7.1 Worst itch numeric rating scale (WI-NRS) 86
10.7.2 Investigator's global assessment of prurigo nodularis (IGA PN) 87
10.7.3 Prurigo activity score (PAS) 88
10.7.4 Dermatology life quality index (DLQI) 90
10.7.5 Hospital anxiety and depression scale (HADS) 91
10.7.6 EQ-5D-5L 92
10.7.7 Pain numeric rating scale 93
10.7.8 Sleep numeric rating scale 94
10.7.9 Patient Global Impression of Change of disease (PGIC) 94
10.7.10 Patient Global Impression of Severity (PGIS) 95
10.7.11 Missed school/work days 96

10.8 APPENDIX 8: DEFINITION OF ANAPHYLAXIS 100

10.9 APPENDIX 9: LIST OF OPPORTUNISTIC INFECTIONS 100

10.10 APPENDIX 10: COUNTRY-SPECIFIC REQUIREMENTS 101

10.11 APPENDIX 11: ABBREVIATIONS 101

10.12 APPENDIX 12: PROTOCOL AMENDMENT HISTORY 103

11 REFERENCES 104

LIST OF TABLES

Table 1 - Objectives and endpoints 28
Table 2 - Overview of study interventions administered 40
Table 3 - Summary of handling procedures 60
Table 4 - Summary of bioanalytical methods for functional dupilumab and anti-dupilumab antibodies 60
Table 5 - Populations for analyses 63
Table 6 - Efficacy analyses 64
Table 7 - Safety analyses 66
Table 8 - Other analyses 66
Table 9 - Protocol-required laboratory assessments 73

1 PROTOCOL SUMMARY

1.1 SYNOPSIS

Protocol title: A randomized, double blind, placebo-controlled, multi-center, parallel group study to evaluate the efficacy and safety of dupilumab in patients with prurigo nodularis who are inadequately controlled on topical prescription therapies or when those therapies are not advisable

Short title: Study of dupilumab for the treatment of patients with prurigo nodularis, inadequately controlled on topical prescription therapies or when those therapies are not advisable

LIBERTY-PN PRIME2 (PRurigo Nodularis Itch Minimization Evaluation 2)

Rationale:

Prurigo nodularis (PN) is a skin disease characterized by multiple, intensely itchy skin eruptions in symmetrically distributed areas of the extremities (1). The main symptom is prolonged, repetitive and uncontrollable rubbing, scratching and uncontrollable itching which leads to hyperkeratotic eroding papules and nodules on the skin. It is difficult to treat and entails a high disease burden. Approximately 50% of patients have either past or current history of atopic dermatitis (AD) or other atopic disorders (2). While case reports suggest that dupilumab may successfully treat patients with PN regardless of a preexisting atopic background (3, 4, 5, 6, 7), it remains unclear to what extent Type 2 cytokines play a role in the pathogenesis in those PN lesions not associated with atopy (8).

There are no United States (US) Food and Drug Administration (FDA) or European Medicines Agency (EMA) approved targeted therapies indicated for the treatment of PN, whether atopic or non-atopic forms. Topical corticosteroids (TCS) and topical calcineurin inhibitors (TCI) are often used initially. While there is a mechanistic rationale for their use, no rigorous clinical studies confirming their efficacy were identified.

Dupilumab is a human monoclonal IgG4 antibody that inhibits interleukin (IL)-4 and IL-13 signaling by specifically binding to the IL-4Rα subunit shared by the IL-4 and IL-13 receptor complexes. Dupilumab inhibits IL-4 signaling via the Type I receptor and both IL-4 and IL-13 signaling through the Type II receptor.

Dupilumab has shown clinical efficacy in multiple diseases with underlying Type 2 inflammation such as AD, asthma, chronic rhinosinusitis with nasal polyposis (CRSwNP), and eosinophilic esophagitis (EoE).

Recently, case series were published that suggest dupilumab may also be effective in the treatment of PN (3, 4, 5, 6, 7).

Type 2 cytokine involvement is implicated to some extent in all forms of PN. It is postulated that dupilumab may play a fundamental role in pruritus based upon its ability to block IL4R activity at the level of the sensory dorsal root ganglion (9). The present protocol will evaluate the efficacy and safety of dupilumab in PN.

Objectives and endpoints

Objectives	Endpoints
**Primary**	
• To demonstrate the efficacy of dupilumab on itch response in patients with PN, inadequately controlled on topical prescription therapies or when those therapies are not advisable.	• Proportion of participants with improvement (reduction) in worst-itch numeric rating scale (WI-NRS) by ≥4 from baseline to Week 12.
**Secondary**	
• To demonstrate the efficacy of dupilumab on additional itch endpoints in patients with PN, inadequately controlled on topical prescription therapies or when those therapies are not advisable.	• Proportion of participants with improvement (reduction) in WI-NRS by ≥4 from baseline to Week 24 **[Key secondary endpoint]**.<br>• Time to onset of effect on pruritus as measured by proportion of participants with an improvement (reduction) in WI-NRS by ≥4 from baseline during the 24-week treatment period.<br>• Change from baseline in WI-NRS at Week 24.<br>• Change from baseline in WI-NRS at Week 12.<br>• Percent change from baseline in WI-NRS at Week 24.<br>• Percent change from baseline in WI-NRS at Week 12.<br>• Percent change from baseline in WI-NRS at Week 4.<br>• Percent change from baseline in WI-NRS at Week 2.<br>• Percent change from baseline in WI-NRS over time until Week 24.<br>• Proportion of participants with WI-NRS reduction ≥4 at Week 4.<br>• Proportion of participants with WI-NRS reduction ≥4 over time until Week 24.<br>• Onset of action in change from baseline in WI-NRS (first p <0.05 difference from placebo in the daily WI-NRS that remains significant at subsequent measurements) until Week 12.
• To demonstrate efficacy of dupilumab on skin lesions of PN.	• Proportion of participants with Investigator's Global Assessment 0 or 1 score for PN-Stage (IGA PN-S) at Week 24 **[Key secondary endpoint]**.<br>• Proportion of participants with IGA PN-S 0 or 1 score at Week 12.<br>• Proportion of participants with IGA PN-S 0 or 1 score at Week 8.<br>• Proportion of participants with IGA PN-S 0 or 1 score at Week 4.<br>• Change from baseline in IGA PN-S score at Week 24.<br>• Change from baseline in IGA PN-S score at Week 12.<br>• Change from baseline in IGA PN-S score at Week 8.<br>• Change from baseline in IGA PN-S score at Week 4.<br>• Proportion of participants with Investigator's Global Assessment 0 or 1 score for PN-Activity (IGA PN-A) at Week 24.<br>• Proportion of participants with IGA PN-A 0 or 1 score at Week 12.<br>• Proportion of participants with IGA PN-A 0 or 1 score at Week 8.<br>• Proportion of participants with IGA PN-A 0 or 1 score at Week 4.

Objectives	Endpoints
• To demonstrate the improvement in health-related quality of life (HRQoL).	• Change from baseline in HRQoL, as measured by Dermatology Life Quality Index (DLQI) to Week 24.<br>• Change from baseline in HRQoL, as measured by DLQI to Week 12.
• To evaluate safety outcome measures.	• Percentage of participants experiencing treatment-emergent adverse events (TEAEs) or serious adverse events (SAEs) from baseline through Week 24.
• To evaluate immunogenicity of dupilumab.	• Incidence of treatment-emergent antidrug antibodies (ADA) against dupilumab over time.

Overall design:

This study is a multi-center, 24-week treatment, parallel, double-blind, randomized, placebo-controlled study to evaluate the use of dupilumab in patients with PN inadequately controlled on topical prescription therapies or when those therapies are not advisable. The study will assess the effect of dupilumab on itch improvement as well as its effect on PN lesions, on patients' HRQoL, anxiety and depression, sleep quality and skin pain, and overall health status.

Disclosure Statement: This is a Parallel, Treatment study, with 2 arms, that is blinded/masked for participants and investigators.

Number of participants:

Approximately 150 participants will be randomized 1:1. This corresponds to approximately 75 participants who will be randomly assigned to each intervention arm.

Intervention groups and duration:

Participants who satisfy the inclusion and exclusion criteria will be randomized (1:1) to one of the following investigational medicinal product (IMP) treatment groups:

- Dupilumab 300 mg
- Matched placebo

Duration of study period (per participant)

- Screening period (2-4 weeks)
- Randomized IMP intervention period (24 weeks)
- Follow-up period (12 weeks)

Study interventions

Investigational medicinal product:

Dupilumab 300 mg and placebo matching dupilumab 300 mg supplied in prefilled syringes that are visually indistinguishable.

Dupilumab

- Formulation: dupilumab 300 mg: a 150 mg/mL dupilumab solution in a pre-filled syringe to deliver 300 mg in a 2 mL injection.
- Route of administration: subcutaneous (SC) injection.
- Dose regimen: 300 mg every 2 weeks (Q2W) after an initial loading dose of 600 mg (2 injections of 300 mg) on Day 1.

Placebo

- Formulation: identical formulation to the active 300 mg formulation without dupilumab, in a pre-filled syringe to deliver placebo in a 2 mL injection.
- Route of administration: SC injection.
- Dose regimen: 1 injection Q2W after an initial loading dose (2 injections) on Day 1.

Noninvestigational medicinal products

Participants will be required to apply moisturizers (emollients) once or twice daily for at least 5 out of the 7 consecutive days immediately before Day 1 and continue until Week 36.

If participants are on a stable regimen of low to medium potency TCS or TCI at the screening visit, they can continue their topical steroid application once daily without tapering from Screening to Week 24. If specific lesions resolve, the participant can stop applying steroids to those sites but are permitted to continue applying to persistent lesions. If participants are on stable regimens of high potency or superpotent steroids, participants should decrease potency to medium potency TCS and continue to apply daily from screening to Week 24. Occlusion is not allowed from Screening to Week 24.

Participants can be rescued with high potency or superpotent TCS/TCI as needed throughout the study.

Post-trial access to study medication

The sponsor does not plan to provide post-trial access to the study medication.

Statistical considerations:

- **Randomization**
  - The participants will be randomized to dupilumab or placebo in 1:1 ratio with stratification factors of documented history of atopy (atopic or non-atopic), stable use of TCS/TCI (yes or no), and country/territory code. Number of participants with active mild AD upon study entry will represent up to 10% of the atopic participants. Both the atopic and the non-atopic PN population will be capped at 60% of the total enrolled population.

- **Sample size calculation**
  - The primary endpoint is the proportion of participants with a WI-NRS reduction of ≥4 from baseline to Week 12. Assuming a response rate of [redacted] in placebo and dupilumab respectively, 56 participants/arm will provide 90% power to detect the difference of [redacted] between dupilumab and placebo with Fisher exact test at 2-sided level of 0.05. Assuming 15% drop out during the 12 weeks of treatment, the target is to randomize 75 participants/arm to allow for up to 10% of atopic patients having active mild AD upon study entry.
- **Primary analysis**
  - The analysis population for the efficacy endpoints will be the intent-to-treat (ITT) population defined as all randomized participants analyzed according to the treatment group allocated by randomization regardless if treatment kit is used or not.
  - The primary analysis on WI-NRS reduction ≥4 at Week 12 will be conducted by using Cochran-Mantel-Haenszel (CMH) test stratifying by stratification factors (documented history of atopy [atopic or non-atopic], stable use of TCS/TCI [yes or no], and region [countries combined]) and covariate of baseline anti-depressant use (yes or no). For participants discontinuing the study treatment before Week 12, their off-study treatment values measured up to Week 12 will be included in the analysis. Participants taking selected prohibited medications and/or rescue medications (details of selection will be specified in the statistical analysis plan [SAP]) prior to Week 12 or have missing data at Week 12 will be considered non-responders.
  - Sensitivity analyses using alternative methods will be performed to handle missing data and/or data collected after participants taking selected prohibited medications and/or rescue medications. A subgroup analysis will be performed excluding participants with a current diagnosis of AD. More details of the sensitivity and subgroup analyses will be specified in the SAP.
- **Analysis of secondary endpoints**
  - Secondary efficacy endpoints that measure binary responses will be analyzed in the same fashion as the primary endpoint.
  - Time-to-event secondary efficacy endpoint will be analyzed using the Cox proportional hazards model, including treatment, stratification factors (countries combined to region), and covariate of baseline anti-depressant use (yes or no). The hazards ratio, its 95% confidence interval and p-value will be reported. Kaplan-Meier curves will be also provided.
  - Continuous secondary efficacy endpoints will be analyzed using a hybrid method of the worst observation carried forward (WOCF) and multiple imputations. Data of participants taking selected prohibited medications and/or rescue medications will be set to missing after the medication usage, and the worst postbaseline value on or before the time of the medication usage will be used to impute missing endpoint value (for participants whose postbaseline values are all missing, the baseline will be used to impute). Participants who discontinue the treatment prematurely are encouraged to follow the planned clinical visits and in those participants who did not take the selected prohibited medications and/or rescue medications, all data collected after treatment

discontinuation will be used in the analysis. For these participants, missing data may still happen despite all efforts have been tried to collect the data after treatment discontinuation. For participants who discontinue due to lack of efficacy, all data collected after discontinuation will be used in the analysis, and a WOCF approach will be used to impute missing data if needed. For participants who discontinued not due to lack of efficacy, a multiple imputation approach will be used to impute missing endpoint value, and this multiple imputation will use all participants excluding participants who have taken the selected prohibited medications and/or rescue medications prior to timepoint of endpoint of interest and excluding patients who discontinue due to lack of efficacy. Each of the imputed complete data will be analyzed by fitting an analysis of covariance (ANCOVA) model with treatment group, stratification factors (countries combined into region), baseline anti-depressant use (yes or no), and relevant baseline measurement as covariates in the model. Statistical inference obtained from all imputed data will be combined using Rubin's rule.

- The safety variables will be summarized using descriptive statistics.
- Antidrug antibodies variables including treatment-emergent ADA will be summarized using descriptive statistics by treatment group.

- **Multiplicity considerations**
  - The multiplicity procedure is proposed to control the overall Type-I error rate for testing the primary endpoint and the key secondary endpoints. Detailed hierarchical testing procedure will be defined in the study SAP. The study is considered positive when the primary endpoint achieves statistical significance.
- **Planned database lock date**
  - A primary database lock will be performed when all randomized participants have completed their 24-week treatment phase.
  - The database will be updated at the end of the study for all participants to include the post-treatment follow-up information and updates for the events previously ongoing at the time of the primary lock.
- **Unblinding plan**
  - The unblinding plan will be detailed in the SAP or in a separate unblinding plan.

Data Monitoring Committee: No

1.2 SCHEMA

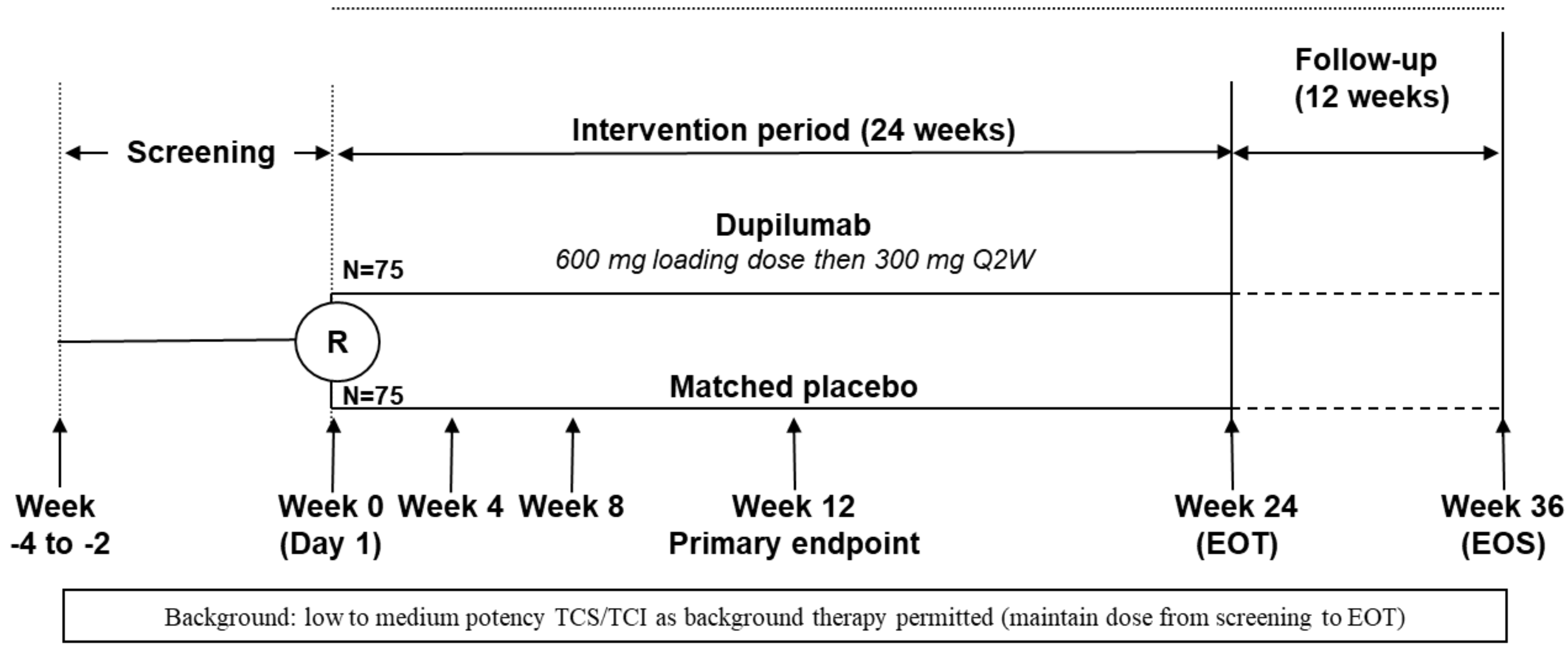

Dupilumab 300 mg Q2W, administered as 1 SC injection of dupilumab 300 mg (2 mL)
A 600 mg loading dose will be administered on Day 1.
Matched placebo is prepared in the same formulation without the addition of protein (ie, the active substance)

EOS: end of study; EOT: end of treatment; PN: prurigo nodularis; Q2W: every 2 weeks; R: randomization; SC: subcutaneous; TCI: topical calcineurin inhibitors; TCS: topical corticosteroids.

1.3 SCHEDULE OF ACTIVITIES (SOA)

Procedure	Screening (2 to 4 weeks before Day 1)	Intervention period (Weeks)					Follow-up (12 weeks)	Notes
		0 (Day 1)	4	8	12	24		
**Visit**	1	2[a]	3	4	5	6[b] (EOT)	7 (EOS)	Visit window: ±3 days for Visits 3, 4, 5, 6, and 7.
**Screening and baseline**								
Informed consent	X							Separate consent to be obtained for optional procedures (DNA and RNA sampling, and serum/plasma sampling for archival; HIV test if specific consent locally required).
Medical history	X							
Prior and concomitant medication	X	X	X	X	X	X	X	Concomitant medication will be collected throughout the study.
Demography	X							
Inclusion and exclusion criteria	X	X						
Hepatitis[c], HIV serology, TB test	X							HBs Ag, HBs Ab, HBc Ab, HCV Ab, HIV screen (Anti-HIV-1 and HIV-2 antibodies), TB test (performed locally if required and results noted in the eCRF).
Randomization		X						
**Study intervention[d]**								
Dispense or download e-diary	X	X	X	X	X	X	X	Device will be dispensed at Screening (including instructions for use). At EOS, the e-diary will be downloaded and returned to the site.
Participant e-diary training	X	X						
Call IVRS/IWRS	X	X	X	X	X	X	X	At screening visit, IVRS/IWRS will be called after medical history check.

Procedure	Screening (2 to 4 weeks before Day 1)	Intervention period (Weeks)					Follow-up (12 weeks)	Notes
		0 (Day 1)	4	8	12	24		
**Visit**	**1**	**2**[a]	**3**	**4**	**5**	**6**[b] **(EOT)**	**7 (EOS)**	Visit window: ±3 days for Visits 3, 4, 5, 6, and 7.
IMP administration		X	X	X	X			IMP administration Q2W (±3 days) from Week 0 until Week 22. Between visits, the participants will self-inject IMP at home or may choose to have injections administered at home by a caregiver or at the study site.
**Safety**[d]								
Physical examination	X	X				X	X	Including skin (full body skin exam), nasal cavities, eyes, ears, respiratory, cardiovascular, gastrointestinal, neurological, lymphatic, and musculoskeletal systems.
Vital signs	X	X	X	X	X	X	X	Including SBP and DBP (mmHg), pulse rate (beats per minute), body temperature (°C), and respiratory rate. Body weight will be recorded at Visits 1, 6, and 7 only. Height will be recorded at Visit 1 only.
12-lead ECG	X					X		Locally collected and read.
Hematology, biochemistry[e]	X	X	X	X	X	X	X	Including hemoglobin, hematocrit, platelet count, total WBC count, differential count, total RBC count, creatinine, BUN, glucose, lactate dehydrogenase, uric acid, total cholesterol, total protein, albumin, total bilirubin, ALT, AST, alkaline phosphatase, electrolytes (sodium, potassium, chloride), bicarbonate, creatine phosphokinase, and hemoglobin A1c (only for diabetic patients without hemoglobin A1c laboratory results within 3 months before screening visit).

Procedure	Screening (2 to 4 weeks before Day 1)	Intervention period (Weeks)					Follow-up (12 weeks)	Notes
		0 (Day 1)	4	8	12	24		
**Visit**	**1**	**2**[a]	**3**	**4**	**5**	**6**[b] **(EOT)**	**7 (EOS)**	Visit window: ±3 days for Visits 3, 4, 5, 6, and 7.
Urinalysis	X	X	X	X	X	X		Dipstick analysis including specific gravity, pH, glucose, ketones, blood, protein, nitrite, leukocyte esterase, urobilinogen and bilirubin. If positive for protein and/or red blood cells, microscopic analysis will be performed by the central laboratory. Creatinine, leukotriene and tetranor PGDM will be tested by the central laboratory. Urine sample has to be collected at the same time (±1 hour) established at V2 in a given patient, if possible.
CD4 T cell count and HIV viral load	X					X		For participants with HIV history only.
Pregnancy test (WOCBP only)[g]	Serum	Urine	Urine	Urine	Urine	Urine	Urine	In between visit urine pregnancy tests will be performed at home (Weeks 16, 20, 28, 32).
AE reporting, including SAEs	X	X	X	X	X	X	X	
Rescue medication use		←---→						
**PK and ADA**[d, f]								
Serum PK samples for dupilumab concentration		X	X	X	X	X	X	
Anti-dupilumab antibody		X			X	X	X	
**Biomarkers**[d]								
Total serum IgE		X	X	X	X	X	X	
Optional serum/plasma for archival samples		X	X	X	X	X	X	Archive serum and plasma samples will be collected and stored for possible future analysis of potential biomarkers of drug response, disease activity, safety, and the Type 2 inflammation pathway.

Procedure	Screening (2 to 4 weeks before Day 1)	Intervention period (Weeks)					Follow-up (12 weeks)	Notes
		0 (Day 1)	4	8	12	24		
**Visit**	1	2[a]	3	4	5	6[b] (EOT)	7 (EOS)	Visit window: ±3 days for Visits 3, 4, 5, 6, and 7.
Optional DNA (whole blood) sample[h]		X						
Optional RNA (whole blood) sample[h]		X				X		
**Efficacy**[d]								
WI-NRS	NRS: once a day from Screening to EOS							To be recorded once a day in e-diary.
Pain-NRS								
Sleep-NRS								
DLQI		X	X	X	X	X	X	Entered by participant during site visit on a tablet provided to the site.
HADS		X			X	X	X	
EQ-5D-5L		X			X	X	X	
PGIS	X	X	X	X	X	X		
PGIC			X	X	X	X		
Missed school/work days (baseline version)		X						
Missed school/work days (postbaseline version)					X	X	X	
Modified PAS (screening-baseline version)	X	X						Investigator's assessment to be entered on a tablet provided to the site.
Modified PAS (follow-up version)			X	X	X	X	X	
IGA PN-A and IGA PN-S		X	X	X	X	X	X	

a All assessments at Visit 2 (Day 1) are to be conducted pre-IMP dose with the exception of the assessment of local tolerability of SC injections.

b Participants who discontinue the study treatment prematurely (prior to completing the 24-week treatment period) will perform the EOT assessments at the time of discontinuation to assure a complete clinical assessment in close temporal proximity to the premature termination of study treatment. In addition, to allow assessment of participant outcomes over the stipulated study period, participants will be asked and encouraged to complete all remaining study visits and participate in all assessments according to the visit schedule.

c In case of results showing HBs Ag (negative) and HBc Ab (positive), an HBV DNA testing will be performed and should be confirmed negative prior to randomization. In case of results showing HCV Ab (positive), HCV RNA testing will be performed and should be confirmed negative prior to randomization.

d Assessments/procedures should be conducted in the following order: PRO, investigator assessments, safety and laboratory assessments (including sample collection for ADA, PK, biomarker, and optional DNA and RNA), and administration of IMP.

e Refer to Section 10.2 and central lab manual for collection details.

f PK and ADA samples to be collected prior to the administration of the drug. In the event of any SAE, any AE of severe injection site reaction lasting longer than 24 hours, or any AESI of anaphylactic reaction or systemic allergic reaction that is related to IMP and require treatment, PK and ADA samples will be collected at or near the onset of the event for any additional analysis if required or for archival purposes.

g Pregnancy will lead to definitive treatment discontinuation in all cases. Female (WOCBP) participants will be supplied with urine dipsticks for use between visits and complete the Home pregnancy diary.

h DNA sample should be collected at the Day 1 visit, but can be collected at any visit during the study. RNA samples must be collected before the administration of the first dose of study drug and at Week 24.

ADA: antidrug antibodies; AE: adverse event; AESI: adverse event of special interest; ALT: alanine aminotransferase; AST: aspartate aminotransferase; BUN: blood urea nitrogen; DBP: diastolic blood pressure; DLQI: Dermatology Life Quality Index; DNA: deoxyribonucleic acid; ECG: electrocardiogram; eCRF: electronic case report form; EOS: end of study; EOT: end of treatment; EQ-5D-5L: Euroqol 5 dimensions 5 levels; HADS: Hospital Anxiety and Depression Scale; HBc Ab: hepatitis B core antibody; HBs Ab: hepatitis B surface antibody; HBs Ag: hepatitis B surface antigen; HBV: hepatitis B virus; HCV: hepatitis C virus; HCV Ab: hepatitis C virus antibody; HIV: human immunodeficiency virus; Ig: immunoglobulin; IGA PN: Investigator's Global Assessment for prurigo nodularis; IGA PN-A: IGA PN-Activity; IGA PN-S: IGA PN-Stage; IMP: Investigational Medicinal Product; IVRS: interactive voice response system; IWRS: interactive web response system; NRS: numeric rating scale; PAS: prurigo activity score; PGIC: Patient Global Impression of Change; PGIS: Patient Global Impression of Severity; PK: pharmacokinetic; PRO: patient reported outcome; Q2W: every 2 weeks; RBC: red blood cells; RNA: ribonucleic acid; SAE: serious adverse event; SBP: systolic blood pressure; SC: subcutaneous; TB: tuberculosis; WBC: white blood cells; WI-NRS: worst itch numeric rating scale; WOCBP: woman of childbearing potential.

2 INTRODUCTION

Dupilumab is a human monoclonal IgG4 antibody that inhibits IL-4 and IL-13 signaling by specifically binding to the IL-4Rα subunit shared by the IL-4 and IL-13 receptor complexes. Dupilumab inhibits IL-4 signaling via the Type I receptor and both IL-4 and IL-13 signaling through the Type II receptor. Blocking IL-4Rα with dupilumab inhibits IL-4 and IL-13, key cytokines that drive Type 2 inflammatory responses, including the release of proinflammatory cytokines, chemokines, nitric oxide, and IgE.

Dupilumab is approved by major regulatory agencies for use in AD and in asthma in adults and adolescents, and in the US for CRSwNP in adults.

2.1 STUDY RATIONALE

Prurigo nodularis is a skin disease characterized by multiple, intensely itchy skin eruptions in symmetrically distributed areas of the extremities (1). It is difficult to treat and entails a high disease burden.

Dupilumab has shown efficacy in multiple diseases with underlying Type 2 inflammation such as AD, asthma, CRSwNP and EoE. In light of the published research suggesting a principal role for Type 2 cytokines in the pathogenesis of PN (see Section 2.2), the Sponsor proposes that selective and simultaneous inhibition of IL-4 and IL-13 signaling such as that achieved with dupilumab treatment may provide clinical benefit in relieving the signs and symptoms of PN, especially in the high unmet need population of patients inadequately treated with, or ineligible for, TCS.

Recently, case series were published that suggest that dupilumab may also be effective in the treatment of PN (3, 4, 5, 6). Type 2 cytokine involvement is implicated in all forms of PN. This is consistent with a recent case series of nine patients without an atopic history who benefitted from dupilumab treatment (7).

The primary hypothesis of the study is that dupilumab will be effective in reducing itch and the secondary hypothesis is that it will reduce and promote the healing of lesions of PN, in patients who are inadequate responders to topical prescription therapies or when those therapies are not advisable.

2.2 BACKGROUND

Prurigo nodularis is a skin disease characterized by multiple, intensely itchy skin eruptions in symmetrically distributed areas of the extremities (1). The main symptom is prolonged, repetitive and uncontrollable rubbing, scratching and uncontrollable itching which leads to hyperkeratotic eroding papules and nodules on the skin. A broadly accepted definition for chronic prurigo has been published by the European Academy of Dermatology and Venereology (EADV) (10). The convened experts agreed that chronic prurigo should be used as an umbrella term for the range of clinical manifestations (eg, papular, nodular, plaque or umbilicated types). Prurigo nodularis is

considered a distinct disease defined by the presence of chronic pruritus for ≥6 weeks, history and/or signs of repeated scratching and multiple localized/generalized pruriginous skin lesions (whitish, hyperpigmented, or pink papules, nodules and/or plaques).

Pathophysiological studies in PN patients reveal increased dermal nerve fiber density and changes in many types of skin cells. These skin cells cause inflammation and pruritus (severe, constant itching) through the release of cytokine IL-31, tryptase, prostaglandins, eosinophil cationic protein, histamine, and neuropeptides, such as substance P, calcitonin gene-related peptide and nerve growth factor (11). The itch caused by primary dermatoses or systemic disorders is postulated to cause an itch scratch cycle which leads to neuronal hypersensitization. This neuronal hypersensitization is the final common pathway which drives the symptomatology of PN (1). Fukushi et al. studied 22 PN skin biopsies and found that 86% of them showed activation of STAT6, a marker for Type 2 inflammatory pathway activation downstream of the Type 2 cytokines IL-4 and IL-13 (12). Consistent with this, IL-4 and IL-5 messenger RNA expression is upregulated in the skin of PN patients (13). Importantly, these two studies did not mention whether these skin biopsy specimens came from patients with or without atopy. The etiology of the profound pruritus in this disease is thought to be driven by high Type 2 inflammation driving activity at the level of the sensory neuron. Human sensory neurons in skin that transmit the sensation of itch are known to express IL- 4Rα, IL-13Ra1, and IL-31RA (14). On testing for functional stimulation, IL-4 was shown to enhance responsiveness to other pruritogens like histamine (14). These findings support that Type 2 cytokines may play a principal role in the pathogenesis of PN.

Due to the central manifestation of itch, PN carries a significant burden of disease (10, 15). The effect on quality of life due to PN has been reported to be higher than other common skin disorders like AD and psoriasis (16). Patients report chronic sleep loss due to constant itching; constant burning, stinging, and pain at affected area; and chronic depression, anxiety, anger, disgust, and shame; and hence overall experience a great impact on their quality of life. According to a 5 year cross-sectional study on 909 adult PN patients in the John Hopkins hospital system (15), PN is a key contributing factor to mood disorders such as anxiety and depression. Of all patients with chronic pruritus, 70% have some type of psychiatric comorbidity, and the severity of depression in patients with chronic pruritus correlates with intensity of itch. The concomitant psychologic component of PN likely contributes to the challenging nature of treating this disease.

Data on the epidemiology of PN are limited. Most studies show a predominance of older patients with a median age of more than 50 years (2, 10, 15). Prurigo nodularis is only occasionally observed in younger patients, in whom it is often associated with atopic conditions (17).

There are no FDA or EMA approved targeted therapies indicated for the treatment of PN, whether atopic or non-atopic forms; the lesions of atopic and non-atopic PN are clinically indistinguishable and treatment options remain the same. Topical corticosteroids and TCI are often used initially. While there is a mechanistic rationale for their use, no rigorous clinical studies confirming their efficacy were identified. Anecdotal feedback obtained from dermatological experts indicate variable, modest efficacy. For thicker lesions, TCS is also administered intralesionally. Lesional cryotherapy is another available topical treatment; case reports indicate temporary relief. Antihistamines and antileukotrienes are occasionally used; their efficacy, however, is not supported by well conducted, randomized clinical trials and is rated low

by patients (18). Phototherapy, in particular narrowband ultraviolet B (UVB), is occasionally added in patients not responding to topical pharmacotherapy. While some improvements with phototherapy have been reported, they remain usually partial and complete remissions are uncommon.

Oral immunosuppressants such as methotrexate and cyclosporine have been used off-label with some success as reported based on case reports and retrospective (18). Use of cyclosporine in PN is limited by commonly recognized toxicities including hypertension, impaired renal and hepatic function, and potential for increased susceptibility to infections and cancer, particularly skin cancer, due to decreased cancer immunosurveillance (19). Methotrexate has well established toxicities, in particular, myelosuppression and hepatotoxicity. In addition, the broad immunosuppression caused by all these drugs carries an increased risk of developing serious bacterial, fungal, viral, and mycobacterial infections.

Neuromodulatory agents such as gabapentin and anti-inflammatory agents such as thalidomide have been used in PN with varying degrees of success (18), but have also considerable adverse effects. Adverse effects of thalidomide include peripheral neuropathies, sedation, dizziness and teratogenicity, while adverse effects of gabapentin include headache, sedation and dizziness.

Overall, despite the use of multiple treatments, many patients with PN remain uncontrolled, and some of the available therapies are associated with serious potential adverse reactions. Given the lack of targeted treatments and the suboptimal efficacy associated with currently available therapies, there remains a significant unmet need in patients with PN.

Dupilumab has shown efficacy in multiple diseases with underlying Type 2 inflammation such as AD, asthma, CRSwNP, and EoE. The most compelling data suggesting that PN is driven by Type 2 inflammation are the many case series of PN patients successfully treated with dupilumab. Recently, five case series were published that suggest that dupilumab may also be effective in the treatment of PN: Mollanazar et al. (4 patients, 3 of those without history of AD) (3), Beck et al. (3 patients) (4), Rambhia et al. (2 patients) (5), Calugareanu et al. (one patient) (6), and Zhai et al. (9 patients, all without history of AD) (7). These case series reported major improvements in treatment-refractory cases of PN after treatment with dupilumab 300 mg Q2W (after an initial loading dose of 600 mg). These published case reports show rapid and substantial effects of dupilumab on itch, and somewhat less well documented effects also on lesions in patients with PN refractory to standard treatment.

2.3 BENEFIT/RISK ASSESSMENT

Dupilumab solution for injection is currently authorized:

- In over 40 countries worldwide including the US, European Union (EU) (Centralised Procedure), and Japan for the treatment of adults with inadequately controlled moderate-to-severe AD. In the US and EU, it has also been authorized for use in adolescent patients (≥12 years) with inadequately controlled moderate-to-severe AD.

- In the US for use in moderate-to-severe eosinophilic or oral steroid dependent asthma, in the EU for severe Type 2 asthma in adults and adolescents, and in Japan for use in adults and adolescents (≥12 years) with severe or refractory bronchial asthma,
- In the US for use in adults with inadequately controlled CRSwNP.

Dupilumab has shown clinically relevant benefit in several Type 2 driven immunological disorders such as AD, bronchial asthma, CRSwNP, and EoE. In asthma and AD indications, studies were also conducted in adolescents and similar benefit to adults was observed.

While the mechanism of PN is not well-known, there is some evidence of a Type 2 immunologic signature in prurigo nodule skin biopsy specimens and activation of Type 2 transcription factors STAT3 and STAT6 in the epidermis (12, 13). Therefore, it is hypothesized that dupilumab may show significant clinical benefit in the treatment of PN. This hypothesis is supported by a series of case reports that show dupilumab treatment resulting in a significant reduction in pruritus in 19 PN patients (3, 4, 5, 6, 7).

No tissue targets or specific hazards to humans were identified in nonclinical general and reproductive toxicology studies.

Dupilumab has an extensive safety database. As of the 28 March 2019 (data lock point), 8798 subjects were enrolled into the development program for dupilumab and are included in the safety population treated: 242 as healthy volunteers, 4227 from AD studies, 3377 from asthma studies, 782 from CRSwNP studies, 53 from EoE studies, 117 from grass allergy and peanut allergy studies. The number of subjects exposed to dupilumab in clinical studies was 7781 (218 in healthy volunteer studies, 3931 in AD studies, 3073 in asthma studies, 470 in CRSwNP studies, 26 in EoE studies, and 63 in grass allergy and peanut allergy studies). Based on the sales figure retrieved from Intercontinental Marketing Services Health and using the World Health Organization’s defined average daily dose for dupilumab of 21.4 mg, the cumulative post marketing exposure to dupilumab is estimated to be 38 816 patient years (01 January 2017 through 31 December 2018).

Dupilumab was generally well tolerated in all populations tested in clinical development programs. The adverse drug reactions (ADRs) identified to date for dupilumab include injection site reactions, conjunctivitis, oral herpes, conjunctivitis allergic, conjunctivitis bacterial, herpes simplex, blepharitis, dry eye, eye pruritus, and eosinophilia. These ADRs occur with relatively low frequency with dupilumab treatment, and were generally mild or moderate, transient, and manageable. More significant serious allergic reactions were very rare. Importantly, no increased overall infection risk was observed in patients treated with dupilumab.

Systemic hypersensitivity has been established as an important identified risk with dupilumab. As protein therapeutics, all monoclonal antibodies are potentially immunogenic. Rare serious and systemic hypersensitivity reactions have been observed in the dupilumab program including serum sickness/serum sickness-like reaction in the adult AD program and anaphylaxis related to dupilumab in the adult asthma clinical trials.

It is hypothesized that dupilumab in patients with PN will have a favorable safety profile as observed across other Type 2-driven immunological disorders.

The safety data available to date, in conjunction with the risk monitoring and mitigation strategies in the study protocol, and the clinical benefit of dupilumab demonstrated in multiple Type 2 indications (AD, asthma, CRSwNP) and case reports and series in PN so far, support a favorable benefit-risk profile for dupilumab.

A risk-benefit statement with respect to the overall development program is provided in the investigator’s brochure (IB).

3 OBJECTIVES AND ENDPOINTS

Table 1 - Objectives and endpoints

Objectives	Endpoints
**Primary**	
• To demonstrate the efficacy of dupilumab on itch response in patients with PN, inadequately controlled on topical prescription therapies or when those therapies are not advisable.	• Proportion of participants with improvement (reduction) in worst-itch numeric rating scale (WI-NRS) by ≥4 from baseline to Week 12.
**Secondary**	
• To demonstrate the efficacy of dupilumab on additional itch endpoints in patients with PN, inadequately controlled on topical prescription therapies or when those therapies are not advisable.	• Proportion of participants with improvement (reduction) in WI-NRS by ≥4 from baseline to Week 24 **[Key secondary endpoint]**.<br>• Time to onset of effect on pruritus as measured by proportion of participants with an improvement (reduction) in WI-NRS by ≥4 from baseline during the 24-week treatment period.<br>• Change from baseline in WI-NRS at Week 24.<br>• Change from baseline in WI-NRS at Week 12.<br>• Percent change from baseline in WI-NRS at Week 24.<br>• Percent change from baseline in WI-NRS at Week 12.<br>• Percent change from baseline in WI-NRS at Week 4.<br>• Percent change from baseline in WI-NRS at Week 2.<br>• Percent change from baseline in WI-NRS over time until Week 24.<br>• Proportion of participants with WI-NRS reduction ≥4 at Week 4.<br>• Proportion of participants with WI-NRS reduction ≥4 over time until Week 24.<br>• Onset of action in change from baseline in WI-NRS (first $p < 0.05$ difference from placebo in the daily WI-NRS that remains significant at subsequent measurements) until Week 12.
• To demonstrate efficacy of dupilumab on skin lesions of PN.	• Proportion of participants with Investigator's Global Assessment 0 or 1 score for PN-Stage (IGA PN-S) at Week 24 **[Key secondary endpoint]**.<br>• Proportion of participants with IGA PN-S 0 or 1 score at Week 12.<br>• Proportion of participants with IGA PN-S 0 or 1 score at Week 8.<br>• Proportion of participants with IGA PN-S 0 or 1 score at Week 4.<br>• Change from baseline in IGA PN-S score at Week 24.<br>• Change from baseline in IGA PN-S score at Week 12.<br>• Change from baseline in IGA PN-S score at Week 8.<br>• Change from baseline in IGA PN-S score at Week 4.<br>• Proportion of participants with Investigator's Global Assessment 0 or 1 score for PN-Activity (IGA PN-A) at Week 24.<br>• Proportion of participants with IGA PN-A 0 or 1 score at Week 12.<br>• Proportion of participants with IGA PN-A 0 or 1 score at Week 8.<br>• Proportion of participants with IGA PN-A 0 or 1 score at Week 4.
• To demonstrate the improvement in health-related quality of life (HRQoL).	• Change from baseline in HRQoL, as measured by Dermatology Life Quality Index (DLQI) to Week 24.<br>• Change from baseline in HRQoL, as measured by DLQI to Week 12.

Objectives	Endpoints
• To evaluate safety outcome measures.	• Percentage of participants experiencing treatment-emergent adverse events (TEAEs) or serious adverse events (SAEs) from baseline through Week 24.
• To evaluate immunogenicity of dupilumab.	• Incidence of treatment-emergent antidrug antibodies (ADA) against dupilumab over time.
**Tertiary/exploratory**	
• To demonstrate a reduction in the use of rescue medication and systemic immunosuppressant	• Use of high potency TCS or superpotent TCS rescue medication through Week 24<br>• Use of systemic immunosuppressant through Week 24, constituting treatment failure
• To evaluate exploratory outcome measures	• Change from baseline in Hospital Anxiety and Depression Scale (HADS) total score to Week 24.<br>• Change from baseline in EQ5D-5L to Week 24.<br>• Change from baseline in Pain numeric rating scale (NRS) to Week 4, Week 8, Week 12, and Week 24, respectively.<br>• Change from baseline in Sleep NRS to Week 4, Week 8, Week 12, and Week 24, respectively.<br>• Missed school/work days through Week 24.<br>• Incidence of skin-infection TEAEs (excluding herpetic infections) through Week 24.
• To evaluate efficacy of dupilumab on skin lesions using a modified PAS 5-item questionnaire	• Proportion of patients who achieve ≥75% healed lesions from PAS at Week 4, Week 8, Week 12, and Week 24, respectively.<br>• Change from baseline in exact number of lesions in representative area (as determined from PAS) at Week 4, Week 8, Week 12, and Week 24, respectively.
• To evaluate efficacy of dupilumab on other PN endpoints	• Change from baseline in Patient Global Impression of Severity (PGIS) of PN to Week 4, Week 8, Week 12, and Week 24, respectively.<br>• Patient Global Impression of Change (PGIC) of PN at Week 4, Week 8, Week 12, and Week 24, respectively.
**PK**	
• To evaluate pharmacokinetic (PK) and pharmacodynamic (PD) outcome measures	• Serum functional dupilumab concentrations and PK profile.<br>• Pharmacodynamic response for selected biomarkers (total IgE).

3.1 APPROPRIATENESS OF MEASUREMENTS

The study endpoints will include both assessments of itch and assessments of the PN lesions. As discussed in Section 2.2, the severe itch observed in this disease, the fact that lesions are typically observed in areas that are within reach for scratching, and the importance of itch-scratch cycles in the pathophysiology of the disease support itch as a reasonable primary endpoint. This choice is in line with the study design publicly available for other drug classes such as NK1-antagonists (20). Amongst standard measurement instruments for evaluation of chronic pruritus, NRS assessing worst itch in the past 24 hours features high reliability and concurrent validity and is a popular choice for all patients due to its simple format (21, 22, 23, 24). The proposed WI-NRS is currently being used in Phase 3 trial assessing serlopitant efficacy in PN (20). A similar pruritus NRS (peak pruritus) has been validated for AD. Additional validation work covering PN as additional indication will be conducted in the context of the current study. The primary assessment will be a

responder analysis based on an at least 4 points improvement of the WI-NRS at Week 12, similar to the assessment widely used in AD studies, and similar to the value proposed for assessment of clinical improvement in the WI-NRS as measures of response in the Phase 2 trial assessing serlopitant efficacy and safety in PN, and currently used in the Phase 3 trial (20). The itch response occurs typically faster than the lesion response but since improvement of both may occur prior to Week 12, responder analyses assessments will be performed at earlier time points as well, starting at Week 2 for itch, and Week 4 for lesions. A key secondary endpoint will be the responder analyses of itch improvement of at least 4 points at Week 24.

As another key secondary endpoint, PN lesions will be assessed by an IGA PN-Stage, which is a scale that measures the approximate number of nodules. The estimated number of lesions correlates well with increasing DLQI scores (ie, worsened quality of life), and demonstrates very good inter-rater reliability and test-retest reliability (25). The IGA scale is widely used in several dermatological indications (eg, as Physician Global Assessment [PGA] in psoriasis). A modified PAS will be used as an exploratory measure. Both instruments are PN-specific and have been developed by experts in the field. These are the only two disease-specific instruments currently available for the lesion assessment. The validation of both instruments for use in PN will be conducted in the context of the Phase 3 trials.

Secondary endpoints will also include HRQoL assessments using the skin-disease specific DLQI in recognition of the significant impacts on quality of life experienced by patients with PN. Even though not validated in PN specifically, the DLQI is well established and widely used to assess HRQoL in patients with skin conditions in clinical trials (26).

4 STUDY DESIGN

4.1 OVERALL DESIGN

This study is a multi-center, 24-week treatment, parallel, double-blind, randomized, placebo-controlled study to evaluate the use of dupilumab in patients with PN inadequately controlled on topical prescription therapies or when those therapies are not advisable.

The study will assess the effect of dupilumab on itch improvement as well as its effect on PN lesions, on patients' health-related quality of life, anxiety and depression, sleep quality and skin pain, and overall health status.

Participants who satisfy the inclusion and exclusion criteria will be randomized (1:1) to one of the following IMP treatment groups:

- Dupilumab 300 mg
- Matched placebo

Duration of study period (per participant)

- Screening period (2-4 weeks)
- Randomized IMP intervention period (24 weeks)
- Follow-up period (12 weeks)

4.2 SCIENTIFIC RATIONALE FOR STUDY DESIGN

This study is a multi-center, 24-week treatment, double-blind, randomized, placebo-controlled study to evaluate the use of dupilumab in patients with PN inadequately controlled on topical prescription therapies or when those therapies are not advisable. Based on mechanistic data (see Section 2), Type 2 inflammation may play a principal role in the pathogenesis of PN in all patients.

The double-blind design intends to mitigate potential bias and placebo control is necessary to assess the net effect of active dupilumab treatment.

The patient population to be studied in this trial is intended to include patients with underlying systemic medical conditions known to be associated with PN. Given that better control of severe manifestations of underlying systemic illnesses can confound the primary itch endpoint, we will enroll 40% to 60% of non-atopic PN patients that have medically well-controlled systemic conditions. Since the literature suggests that approximately 20%-60% of PN patients have a history of atopy (defined as having a medical history of AD, allergic rhinitis/rhinoconjunctivits, asthma, or food allergy), our study design will require that the remaining PN patients have a history or a current diagnosis of atopy. Because active AD can confound the primary endpoint, we will exclude moderate-to-severe AD patients and will institute a cap of up to 10% of the atopic patient subgroup having active mild AD. It is the goal of this design to mirror the overall PN patient population consistent with real-world estimates of overall prevalence while trying to minimize potential confounders on the primary endpoint.

The primary endpoint, WI-NRS, is a validated measurement of the highest PN burden, ie, extreme itching.

Dupilumab treatment has shown clinical efficacy prior to Week 24 in all Phase 3 trials examined across all indications. In addition, available biomarker data of disease response, including TARC and eotaxin-3, plateau after Week 16 but before Week 24 in prior AD trials, including the 52-week AD study (R668-AD-1224; CHRONOS). The Sponsor considers that a 24-week treatment period is an appropriate duration to observe dupilumab's effect in PN to ensure an adequate evaluation of PN lesions as the effect on pruritus is expected to plateau by Week 12 similar to that observed in the atopic dermatitis studies. Weeks 12 to Week 24 will then allow for evaluation of the maintenance of effect on pruritus. The duration of the 12-week follow-up period is based on the time expected for drug concentration to reach zero (below the lower limit of quantification) in most patients after the last dose of dupilumab. This follow-up period will allow assessment of the need for chronic treatment with dupilumab for this disease by an assessment of return of PN symptoms post-treatment.

The study will not include adolescents because PN is rare in adolescent patients.

4.3 JUSTIFICATION FOR DOSE

The selected dosing regimen is dupilumab 300 mg Q2W with a loading dose of 600 mg.

This dose regimen is expected to achieve serum concentrations that saturate the target-mediated clearance pathway rapidly with the loading dose and maintain saturation thereafter. The PK of dupilumab is consistent across populations of patients with AD, asthma, CRSwNP, and EoE, as well as healthy volunteers. Furthermore, the sources of variability of dupilumab PK identified in each population and the magnitude of the covariate effects indicate that body weight is the most influential factor, whereas other covariates identified as being statistically significant have no meaningful impact on dupilumab PK. The immunogenicity of dupilumab is also comparable in these populations (see IB).

Dosing regimens of 300 mg Q2W after a loading dose of 600 mg were used in the PN case studies, which resulted in marked improvement in itch within three months and subsequently a notable improvement of the skin lesions (see Section 2).

The 300 mg Q2W dose, with loading dose, is the approved dose for AD and is an approved dose for asthma in the US and the EU.

Therefore, based on the totality of the data, dupilumab 300 mg Q2W (with loading dose) regimen is adequate for patients with PN and a further dose ranging study is not necessary to select a Phase 3 dose regimen.

4.4 END OF STUDY DEFINITION

A participant is considered to have completed the study if he/she has completed all phases of the study including the last visit.

The end of the study is defined as the date of the last visit of the last participant in the study.

5 STUDY POPULATION

Prospective approval of protocol deviations to recruitment and enrollment criteria, also known as protocol waivers or exemptions, is not permitted.

5.1 INCLUSION CRITERIA

Participants are eligible to be included in the study only if all of the following criteria apply:

Age

I 01. Participants must be 18 to 80 years of age, at the time of signing the informed consent.

Type of participant and disease characteristics

Patients with a clinical diagnosis of PN, as defined by all of the following:

I 02. Diagnosed by a dermatologist for at least 3 months before the Screening visit.

I 03. On the WI-NRS ranging from 0 to 10, patients must have an average worst itch score of ≥7 in the 7 days prior to Day 1.

NOTE: Baseline pruritus NRS average score for maximum itch intensity will be determined based on the average of daily NRS scores for maximum intensity (the daily score ranges from 0 to 10) during the 7 days immediately preceding randomization. A minimum of 4 daily scores out of the 7 days is required to calculate the baseline average score. For patients who do not have at least 4 daily scores reported during the 7 days immediately preceding the planned randomization date, randomization should be postponed until this requirement is met, but without exceeding the 28-day maximum duration of the screening period.

I 04. Patients must have a minimum of 20 PN lesions in total on both legs, and/or both arms and/or trunk, at Screening visit and on Day 1.

NOTE: Patients need to have bilaterally symmetrical lesions on the extremities. The presence of lesions on at least 2 body surface areas is required.

I 05. History of failing a 2-week course of medium-to-superpotent TCS or when TCS are not medically advisable.

NOTE: Failure is defined as patients who are unable to achieve and/or maintain remission and low disease activity (similar to IGA PN-S score of ≤2 [≤19 nodules]) despite treatment with a daily regimen of medium-to-superpotent TCS (±TCI as appropriate), applied for at least 14 days, or for the maximum duration recommended by the product prescribing information, whichever is shorter.

I 06. Have applied a stable dose of topical emollient (moisturizer) once or twice daily for at least 5 out of the 7 consecutive days immediately before Day 1 (NOTE: See exclusion criterion E 20 for limitations regarding emollients; see background treatment with topical emollient in Section 6.1.1).

I 07. Participants must be willing and able to complete a daily symptom eDiary for the duration of the study.

Sex

I 08. Male or Female

Contraceptive use by women should be consistent with local regulations regarding the methods of contraception for those participating in clinical studies.

a) Female participants

- A female participant is eligible to participate if she is not pregnant or breastfeeding, and at least one of the following conditions applies:
  - Is not a WOCBP.

  OR

  - Is a WOCBP and agrees to use a contraceptive method as described in Appendix 4 (Section 10.4) during the study (at a minimum until 12 weeks after the last dose of study intervention).
  - A WOCBP must have a negative highly sensitive (Section 10.2) pregnancy test (urine or serum as required by local regulations) on Day 1 before the first dose of study intervention.
  - If a urine test cannot be confirmed as negative (eg, an ambiguous result), a serum pregnancy test is required. In such cases, the participant must be excluded from participation if the serum pregnancy result is positive.
- Additional details can be found in Appendix 4 (Section 10.4).
- The investigator is responsible for review of medical history, menstrual history, and recent sexual activity to decrease the risk for inclusion of a woman with an early undetected pregnancy.

Informed Consent

I 09. Capable of giving signed informed consent as described in Appendix 1 (Section 10.1) of the protocol which includes compliance with the requirements and restrictions listed in the informed consent form (ICF) and in this protocol. In countries where legal age of majority is above 18 years, a specific ICF must also be signed by the participant’s legally authorized representative.

5.2 EXCLUSION CRITERIA

Participants are excluded from the study if any of the following criteria apply:

Medical conditions

E 01. Presence of skin morbidities other than PN and mild AD that may interfere with the assessment of the study outcomes. Conditions such as, but not limited to, the following: scabies, insect bite, lichen simplex chronicus, psoriasis, acne, folliculitis, habitual picking, lymphomatoid papulosis, chronic actinic dermatitis, dermatitis herpetiformis, sporotrichosis, bullous disease.

NOTE: patients with mild active AD will represent up to 10% of the atopic PN study population.

E 02. PN secondary to medications (eg, opioids, angiotensin converting enzyme [ACE] inhibitors).

E 03. PN secondary to medical conditions such as neuropathy or psychiatric disease (eg, notalgia paresthetica, brachioradial pruritus, neurotic excoriations, obsessive compulsive disorder, delusions of parasitosis, etc).

E 04. Patients with a documented AD severity moderate to severe within 6 months before the screening visit, or documented diagnosis of moderate to severe AD from screening visit to randomization visit (eg, IGA AD of 3 or 4, eczema area and severity index [EASI] ≥16, scoring atopic dermatitis [SCORAD] ≥25).

E 05. Severe concomitant illness(es) under poor control that, in the investigator’s judgment, would adversely affect the patient’s participation in the study. Examples include, but are not limited to patients with life expectancy shorter than 1 year, patients with uncontrolled diabetes (hemoglobin A1c ≥9% according to the laboratory results within 3 months before screening visit), patients with cardiovascular conditions (eg, Class III or IV heart failure according to the New York Heart Association classification), hepato-biliary conditions (eg, Child-Pugh Class B or C), neurological conditions (eg, demyelinating diseases), active major autoimmune diseases (eg, lupus, inflammatory bowel disease, rheumatoid arthritis, etc), other severe endocrinological, gastrointestinal, metabolic, pulmonary, or lymphatic diseases. The specific justification for patients excluded under this criterion will be noted in study documents (chart notes, eCRF, etc).

E 06. Severe renal conditions (eg, patients with uremia and/or on dialysis).

E 07. Participants with uncontrolled thyroid disease.

E 08. Active TB or non-tuberculous mycobacterial infection, or a history of incompletely treated TB will be excluded from the study unless it is well documented by a specialist that the participant has been adequately treated and can now start treatment with dupilumab in the medical judgment of the investigator and/or infectious disease specialist. Tuberculosis testing will be performed on a country-by-country basis, according to local guidelines if required by regulatory authorities or ethics boards.

E 09. Diagnosed active endoparasitic infections; suspected or high risk of endoparasitic infection, unless clinical and (if necessary) laboratory assessment have ruled out active infection before randomization.

E 10. Active chronic or acute infection (except HIV infection) requiring treatment with systemic antibiotics, antivirals, antiprotozoals, or antifungals within 2 weeks before screening visit or during the screening period.

E 11. Known or suspected immunodeficiency, including history of invasive opportunistic infections (eg, TB, histoplasmosis, listeriosis, coccidioidomycosis, pneumocystosis, aspergillosis) despite infection resolution, or otherwise recurrent infections of abnormal frequency or prolonged duration suggesting an immune-compromised status, as judged by the investigator.

E 12. Active malignancy or history of malignancy within 5 years before the baseline visit, except completely treated in situ carcinoma of the cervix, completely treated and resolved non-metastatic squamous or basal cell carcinoma of the skin.

E 13. History of systemic hypersensitivity or anaphylaxis to any biologic therapy, including any excipients.

E 14. Any other medical or psychological condition including relevant laboratory abnormalities at screening that, in the opinion of the investigator, suggest a new and/or insufficiently understood disease, may present an unreasonable risk to the study patient as a result of his/her participation in this clinical trial, may make patient’s participation unreliable, or may interfere with study assessments. The specific justification for patients excluded under this criterion will be noted in study documents (chart notes, eCRF, etc).

E 15. History of substance and/or alcohol abuse.

E 16. Planned major surgical procedure during the patient’s participation in this study.

Prior/concomitant therapy

E 17. Exposure to another systemic or topical investigative drug (monoclonal antibodies as well as small molecules) within a certain time period prior to Visit 1 (screening), as follows: an interval of less than 6 months or <5 PK half-lives for investigative monoclonal antibodies, whichever is longer, and an interval of less than 30 days or <5 PK half-lives, whichever is longer, for investigative small molecules.

E 18. Having used any of the following treatments within 4 weeks before the screening visit

- Systemic immunosuppressive/immunomodulating drugs (eg, systemic corticosteroids, cyclosporine, mycophenolate-mofetil, interferon gamma, Janus kinase inhibitors, azathioprine, methotrexate, hydroxychloroquine, dapsone, sulfasalazine, colchine, etc).
- Intralesional corticosteroid injections and cryotherapy.
- Phototherapy, including tanning beds.

- Naltrexone or other opioid antagonist.
- Gabapentin, pregabalin, and thalidomide.

Or starting to use the following treatments or changed the dose of the following treatments in 3 months before the screening visit or expected the dose of the following treatments will be changed throughout the study:

- Paroxetine, fluvoxamine, or other selective serotonin reuptake inhibitors (SSRIs).
- Serotonin and norepinephrine reuptake inhibitors (SNRIs)
- Amitriptyline or other tricyclic or tetracyclic antidepressants.

E 19. Previous treatment with biologic medicines within the following timeframe:

- Any cell-depleting agents including but not limited to rituximab: within 6 months before the screening visit.
- Omalizumab: within 5 months before screening visit.
- Other immunomodulatory biologics: within 5 half-lives (if known) or 16 weeks before the screening visit, whichever is longer.

E 20. Initiation of treatment with prescription moisturizers or moisturizers containing additives such as ceramide, hyaluronic acid, urea, menthol, polidocanol, or filaggrin degradation products during the screening period (patients may continue using stable doses of such moisturizers if initiated before the Screening visit).

E 21. Initiation of treatment with TCS/TCI (any potency) during the screening period or treatment with high potency or superpotent TCS/TCI during the screening period.

E 22. For participants who were on a stable regimen of TCS/TCI (maintain same medicine, same dose from 2 weeks prior to screening visit) at the screening visit:

- Application of TCS/TCI on fewer than 6 days during the 7 days immediately preceding randomization.
- Application of TCS/TCI of incorrect potency within 7 days before Day 1 according to the requirements of Section 6.1.1, ie, low potency if on low potency at screening visit and medium potency if on medium or higher potency at screening visit.

E 23. Treatment with a live (attenuated) vaccine within 4 weeks before the screening visit.

NOTE: For patients who have vaccination with live, attenuated vaccines planned during the course of the study (based on national vaccination schedule/local guidelines), it will be determined, after consultation with a physician, whether the administration of vaccine can be postponed until after the end of study, or preponed to before the start of the study, without compromising the health of the patient:

- Patient for whom administration of live (attenuated) vaccine can be safely postponed would be eligible to enroll into the study.
- Patients who have their vaccination preponed can enroll in the study only after a gap of 4 weeks following administration of the vaccine.

E 24. Planned or anticipated use of any prohibited medications and procedures during screening and study treatment period.

Prior/concurrent clinical study experience

E 25. Participation in prior dupilumab clinical study; treated in the past with dupilumab; prior use of biologics for PN.

Diagnostic assessments

E 26. For participants without history of HIV infection before screening visit, positive HIV serology at screening.

For participants with history of HIV infection with CD4+ counts ≤300 cells/µL and/or detectable HIV viral load at screening.

E 27. Participants with any of the following result at screening:

- Positive (or indeterminate) HBs Ag or,
- Positive total HBc Ab confirmed by positive HBV DNA or,
- Positive HCV Ab confirmed by positive HCV RNA.

Other exclusions

E 28. Individuals accommodated in an institution because of regulatory or legal order; prisoners or subjects who are legally institutionalized.

E 29. Any country-related specific regulation that would prevent the subject from entering the study - see Section 10.10 (country-specific requirements).

E 30. Participant not suitable for participation, whatever the reason, as judged by the Investigator, including medical or clinical conditions, or participants potentially at risk of noncompliance to study procedures.

E 31. Participants are dependent on the Sponsor or Investigator (in conjunction with Section 1.61 of the International Council for Harmonisation (ICH) good clinical practice (GCP) Ordinance E6).

E 32. Participants are employees of the clinical study site or other individuals directly involved in the conduct of the study, or immediate family members of such individuals.

E 33. Any specific situation during study implementation/course that may raise ethics considerations.

E 34. Sensitivity to any of the study interventions, or components thereof, or drug or other allergy that, in the opinion of the Investigator, contraindicates participation in the study.

5.3 LIFESTYLE CONSIDERATIONS

Not applicable.

5.4 SCREEN FAILURES

Screen failures are defined as participants who consent to participate in the clinical study but are not subsequently randomly assigned to study intervention/entered in the study. A minimal set of screen failure information is required to ensure transparent reporting of screen failure participants to meet the Consolidated Standards of Reporting Trials (CONSORT) publishing requirements and to respond to queries from regulatory authorities. Minimal information includes demography, screen failure reasons, eligibility criteria, and any SAE.

In the case of technical malfunction of equipment, the patient may be rescreened. Patients who fail the exclusion criteria may be rescreened once during the open screening period of the study. Rescreened participants will be assigned a new participant number versus the one received for the initial screening.

There is no requirement for a waiting period between the screen-failure date and the rescreening date. The interactive response technology (IRT) report will flag rescreened patients. Patients that are rescreened must sign a new consent form and all Visit 1 procedures must be repeated.

If certain dynamic laboratory tests do not meet the eligibility criteria at screening, these laboratory assessments may be repeated, at the discretion of the Investigator, if it is judged to be likely to return to acceptable range for study inclusion within the screening visit window prior to Day 1. There is no need to screen fail such participants if the test finally meets the eligibility criteria.

6 STUDY INTERVENTION

Study intervention is defined as any investigational intervention(s), marketed product(s), placebo, or medical device(s) intended to be administered to a study participant according to the study protocol.

6.1 STUDY INTERVENTION(S) ADMINISTERED

Table 2 - Overview of study interventions administered

**ARM name**	Dupilumab	Placebo
**Intervention name**	Dupilumab 300 mg	Placebo matching dupilumab 300 mg
**Type**	Biological/Vaccine	Other
**Dose formulation**	A 150 mg/mL dupilumab solution in a pre-filled syringe to deliver 300 mg in 2 mL	Identical formulation to the active 300 mg formulation without dupilumab, in a pre-filled syringe to deliver placebo in 2 mL
**Unit dose strength(s)**	300 mg	0 mg (placebo)
**Dosage level(s)**	300 mg every 14 ±3 days after an initial loading dose of 600 mg	0 mg every 14 ±3 days with a loading dose of 0 mg
**Route of administration**	Subcutaneous[a]	Subcutaneous[a]
**IMP and NIMP**	IMP	IMP
**Packaging and labeling**	One glass pre-filled syringe packed in a patient kit box. Both the glass pre-filled syringe and the box will be labeled as required per country requirement.	One glass pre-filled syringe packed in a patient kit box. Both glass pre-filled the syringe and the box will be labeled as required per country requirement

a Subcutaneous injection sites should alternate between the upper thighs, 4 quadrants of the abdomen or the upper arms, so that the same site is not injected twice during consecutive administrations. Injection in the upper arms can only be done by a trained person (parent/legally authorized representative/caregiver trained by Investigator or Delegate) or health care professional but not the participant themselves.

IMP: investigational medicinal product; NIMP: noninvestigational medicinal product.

The Investigator or delegate will train the patient (or caregiver) how to prepare and inject IMP at Visit 2. He/she will inject the first of the two injections. The participant (or caregiver) will perform the second injection under the supervision of the Investigator or delegate. Patient should also be trained by the site staff to recognize potential signs and symptoms of hypersensitivity reaction in order to self-monitor at home for at least 30 minutes (or longer per country specific or local site-specific requirements) following injection. In case of hypersensitivity symptoms the patient should contact healthcare provider/emergency. The training must be documented in the participant’s study file.

When the participant has a study visit, the IMP will be administered following clinical procedures and blood collection. Patients should be monitored for at least 30 minutes. The monitoring period may be extended as per country specific or local site-specific requirements.

Between the protocol-scheduled on-site visits, participants are allowed to self-inject IMP at home. Participants who prefer to have a healthcare professional administer the IMP may choose to have injections administered at home by a nurse or at the study site.

Between the protocol-scheduled on-site visits, interim visits may be required for IMP dispensing. As an alternative to these visits, dupilumab 300 mg or matching placebo may be supplied from the site to the participant via a Sponsor-approved courier company where allowed by local regulations and approved by the participant.

6.1.1 Noninvestigational medicinal products

Starting from the screening visit, participants will be instructed to use their daily moisturizer, if it is not containing any compound with known anti-itch effect (such as menthol, polidocanol, pramoxine, lidocaine, prilocaine, capsaicin, naltrexone, N-palmitoylethanolamine, etc). It is not authorized changing emollients or moisturizers or applying products for itching relief during the course of the study.

Participants will be required to apply moisturizers (emollients) once or twice daily for at least 5 out of the 7 consecutive days immediately before Day 1 and continue until Week 36. All types of moisturizers are permitted, but patients may not initiate new treatment with prescription moisturizers or over-the-counter moisturizers containing additives during the screening period or during the intervention period. Patients may continue using stable doses of such moisturizers if initiated before the screening visit.

If participants are on a stable regimen of low to medium potency TCS or TCI at the screening visit, they can continue their topical steroid application once daily without tapering from Screening to Week 24. If specific lesions resolve, the participant can stop applying steroids to those sites but are permitted to continue applying to persistent lesions. If participants are on stable regimens of high potency or superpotent steroids, participants should decrease potency to medium potency TCS and continue to apply daily from screening to Week 24. Stable regimen for TCS is maintaining the same medicine (low to medium potency TCS), and maintaining the same frequency of treatment (once or twice daily) used from 2 weeks prior to screening. Stable regimen for TCI is maintaining the same medicine of TCI and the treatment frequency (once or twice daily) used from 2 weeks prior to screening. If participant’s prior regimen was applying once daily, the participant will maintain daily application during study and for participants who had twice daily prior to screening, participation will maintain twice daily regimen during study. Ifspecific lesions resolve, the participant can stop applying steroids to those sites but are permitted to continue applying to persistent lesions. Occlusion is not allowed from Screening to Week 24.

It is recommended that patients use triamcinolone acetonide 0.1% cream or fluocinolone acetonide 0.025% ointment for medium potency, and hydrocortisone 1% cream for low potency. If rescue with TCS is needed, it is recommended that patients use either betamethasone dipropionate 0.05% optimized ointment for high potency TCS or clobetasol propionate 0.05% cream for super high potency TCS. If patients have tolerance issues with any of these steroids or if they are not commercially available in some countries, they may substitute with products of the same potency from the list provided by the Sponsor.

On areas treated with TCS, moisturizers must be applied once daily only at the time when TCS is not applied (ie, do not use moisturizers and TCS on the same areas at the same time during the day). For example, if TCS are applied in the evening, moisturizers will not be used in the evening on areas treated with TCS, but will be applied to those areas in the morning. On areas not treated with TCS, moisturizers will be applied twice daily - morning and evening.

Rescue therapy is described in Section 6.5.1.

6.2 PREPARATION/HANDLING/STORAGE/ACCOUNTABILITY

6.2.1 Storage and handling

1. The Investigator or designee must confirm appropriate temperature conditions have been maintained during transit for all study intervention received and any discrepancies are reported and resolved before use of the study intervention.
2. Only participants enrolled in the study may receive study intervention and only authorized site staff may supply or administer study intervention. All study intervention must be stored in a secure, environmentally controlled, and monitored (manual or automated) area in accordance with the labeled storage conditions with access limited to the Investigator and authorized site staff.
3. The Investigator, institution, or the head of the medical institution (where applicable) is responsible for study intervention accountability, reconciliation, and record maintenance (ie, receipt, reconciliation, and final disposition records).

6.2.2 Responsibilities

Any quality issue noticed with the receipt or use of an IMP (deficiency in condition, appearance, pertaining documentation, labeling, expiration date, etc) must be promptly notified to the Sponsor. Some deficiencies may be recorded through a complaint procedure (see Section 8.3.6).

A potential defect in the quality of IMP may be subject to initiation of a recall procedure by the Sponsor. In this case, the Investigator will be responsible for promptly addressing any request made by the Sponsor, in order to recall the IMP and eliminate potential hazards.

Under no circumstances will the Investigator supply IMP to a third party (except for direct-to-patient [DTP] shipment, for which a courier company has been approved by the Sponsor), allow the IMP to be used other than as directed by this clinical trial protocol, or dispose of IMP in any other manner.

6.3 MEASURES TO MINIMIZE BIAS: RANDOMIZATION AND BLINDING

All participants will be centrally assigned to randomized study intervention using an IRT. Before the study is initiated, the telephone number and call-in directions for the IVRS and/or the log in information & directions for the IWRS will be provided to each site.

At screening (Visit 1), the Investigator or designee will contact the IRT system to receive the participant number. If a patient who had previously failed screening is approached for re-screening, a new ICF must be signed. In such case, a new patient number will be assigned by IRT.

Methods of assigning patients to treatment group

The randomized intervention kit number list is generated centrally by Sanofi and IMPs are packaged in accordance with this list. The randomization and intervention allocation are performed centrally by an IRT. The IRT generates the participant randomization list and allocates the intervention number and the corresponding intervention kits to the participants according to it.

Participants will be randomized in 1:1 ratio to treatment arms described in Table 2.

Randomization will be stratified by the following factors:

- documented history of atopy (atopic or non-atopic)
  - Atopic: Patients with a physician-documented history of atopic comorbidities defined as AD, allergic rhinitis/rhinoconjunctivits, asthma, or food allergy, or a current diagnosis of at least one of these atopic comorbidities, per investigator judgement. NOTE: enrollment of patients with a current diagnosis of active mild AD will be capped at up to 10% of the participants in this subgroup
  - Non-atopic: Patients without a physician-documented history of atopic comorbidities defined as AD, allergic rhinitis/rhinoconjunctivitis, asthma or food allergy, and without a current diagnosis of at least one of such atopic comorbidities, per investigator judgement
- stable use of TCS/TCI (yes or no)
- country/territory code

A randomized participant is defined as a participant who has been allocated to a randomized intervention regardless whether the intervention kit was used or not (ie, participant registered by the IRT).

A participant cannot be randomized more than once in the study.

Study intervention will be dispensed at the study visits summarized in SoA (see Section 1.3). Returned study intervention should not be re-dispensed to the participants.

Methods of blinding

- Dupilumab 300 mg and placebo matching dupilumab 300 mg will be provided in identically matched 2 mL pre-filled syringes that are visually indistinguishable. Syringes and box will be labeled with a treatment kit number.
- The unblinding plan is described in Section 9.5.2.

Randomization code breaking during the study

The IRT will be programmed with blind-breaking instructions. In case of an emergency, the Investigator has the sole responsibility for determining if unblinding of a participant's treatment assignment is warranted (eg, in case of available antidote). Participant safety must always be the first consideration in making such a determination. If the Investigator decides that unblinding is warranted, the Investigator should make every effort to contact the Sponsor prior to unblinding a participant's treatment assignment unless this could delay emergency treatment of the participant. If a participant's treatment assignment is unblinded, the Sponsor must be notified within 24 hours after breaking the blind. The date and reason that the blind was broken must be recorded in the source documentation and case report form (CRF), as applicable.

6.4 STUDY INTERVENTION COMPLIANCE

Investigator or his/her delegate must ensure that IMP will be administered to each participant according to the labeling instructions.

IMP accountability:

- Intervention units are returned by the participant at each visit. In case of DTP process, the intervention units can be returned by the carrier (if defined in the contract).
- The Investigator counts the number of remaining kit/pre-filled syringe, and fills in the IMP accountability and inventory forms.
- The Investigator or his/her delegate records the dosing information on the appropriate page(s) of the eCRF.
- The monitor in charge of the study then checks the eCRF data by comparing them with the IMP which he/she has retrieved and source documents.

Participant compliance with study intervention will be assessed at each visit. Compliance will be assessed by counting returned kit/pre-filled syringe. Deviation(s) from the prescribed dosage regimen should be recorded in the eCRF.

6.5 CONCOMITANT THERAPY

Any medication or vaccine (including over-the-counter or prescription medicines, vitamins, and/or herbal supplements) that the participant is receiving at the time of enrollment or receives during the study must be recorded along with:

- Reason for use.
- Dates of administration including start and end dates.
- Dosage information including dose and frequency.

The concomitant use of non-sedating antihistamine administration is allowed during the study except for treatment of AD or PN, but dose change of non-sedating antihistamine is not allowed both from Week 11 to Week 12 and from Week 23 to Week 24.

The concomitant use of the following therapies is prohibited during the entire study. Study treatment will need to be discontinued in participants receiving these treatments:

- Systemic immunosuppressive/immunomodulating drugs (eg, systemic corticosteroids, cyclosporine, mycophenolate-mofetil, interferon gamma, Janus kinase inhibitors, azathioprine, methotrexate, hydroxychloroquine, dapsone, sulfasalazine, colchine, etc).
- Other monoclonal antibodies (which are biological modifiers).
- Phototherapy, including tanning beds.
- Naltrexone or other opioid antagonist.
- Gabapentin, pregabalin, and thalidomide.

The concomitant use of the following therapies is prohibited except if the dose has been stable for at least 3 months prior to screening, but study treatment will not need to be discontinued in participants receiving the treatments listed below. The dose should also remain stable (can be reduced or discontinued if medically indicated), but should not be initiated or increased throughout the study.

- Paroxetine, fluvoxamine, or other SSRIs.
- SNRIs.
- Amitriptyline or other tricyclic or tetracyclic antidepressants.

The concomitant use of the following therapies is also prohibited during the entire study, but study treatment will not need to be discontinued in participants receiving the treatments listed below.

- Intralesional corticosteroid injections and cryotherapy.
- Sedating antihistamine.
- Non-sedating antihistamine used specifically for the treatment of itch secondary to AD or PN.

6.5.1 Rescue medicine

The following rescue medications may be used:

- Dermatological preparations of high potency or superpotent TCS and TCI.

If medically necessary (ie, to control intolerable PN symptoms), rescue treatment for PN may be provided to study patients at the discretion of the Investigator.

Although the use of rescue medications is allowed at any time during the study, the use of rescue medications should be delayed, if possible, for at least 14 days following the initiation of the investigational treatment. The date and time of rescue medication administration as well as the name and dosage regimen of the rescue medication must be recorded in the eCRF.

For the purpose of the efficacy responder analysis, a pre-specified algorithm will be used to classify rescue (details in the SAP). In addition, a blinded review of all post-baseline medications to adjudicate rescue treatment, based on medical judgment, will be performed to adjudicate rescue. Patients who receive rescue treatment as per this adjudication during the study will be considered treatment failures.

6.6 DOSE MODIFICATION

No change in IMP dose is allowed.

6.7 INTERVENTION AFTER THE END OF THE STUDY

The Sponsor will not be responsible for intervention after the EOS Visit. Intervention after the EOS Visit will be at the discretion of investigator or treating physician.

7 DISCONTINUATION OF STUDY INTERVENTION AND PARTICIPANT DISCONTINUATION/WITHDRAWAL

7.1 DISCONTINUATION OF STUDY INTERVENTION

7.1.1 Definitive discontinuation

In rare instances, it may be necessary for a participant to permanently discontinue study intervention. If study intervention is permanently discontinued, the participant will remain in the study to be evaluated for safety. See the SoA (Section 1.3) for data to be collected at the time of discontinuation of study intervention.

The participants may withdraw from treatment with the IMP if he or she decides to do so, at any time and irrespective of the reason, or this may be the investigator's decision. All efforts should be made to document the reason(s) for treatment discontinuation and this should be documented in the eCRF.

Participants must be permanently withdrawn from the study treatment for the following reasons:

- At their own request or at the request of their legally authorized representative (legally authorized representative means an individual or judicial or other body authorized under applicable law to consent on behalf of a prospective participant to the patient's participation in the procedure(s) involved in the research).
- If, in the investigator's opinion, continuation in the study would be detrimental to the participant's well-being.
- At the specific request of the Sponsor.
- If they are treated with the specific prohibited medications mentioned in Section 6.5.
- If they miss more than 2 consecutive IMP doses.
- In the event of a protocol deviation, at the discretion of the investigator or the Sponsor.
- Any code broken at the requested of the investigator.
- Pregnancy.
- Anaphylactic reactions or systemic allergic reactions that are related to IMP and require treatment (see Section 10.8).
- Diagnosis of a malignancy during study, excluding carcinoma in situ of the cervix, or squamous or basal cell carcinoma of the skin.
- Any opportunistic infection or other infections whose nature or course may suggest an immunocompromised status (see Section 10.9).
- Serum ALT >3 ULN and Total Bilirubin >2 ULN (see Section 10.6).
- Serum ALT >5 ULN if baseline ALT ≤2 ULN or ALT >8 ULN if baseline ALT >2 ULN (see Section 10.6).

If a clinically significant finding is identified (including, but not limited to changes from baseline in QT interval corrected using Fridericia's formula [QTcF] after enrollment), the Investigator or qualified designee will determine if the participant can continue in the study and if any change in participant management is needed. This review of the ECG printed at the time of collection must be documented. Any new clinically relevant finding should be reported as an AE.

Any abnormal laboratory value or ECG parameter will be immediately rechecked for confirmation within a reasonable timeframe as assessed by the Investigator before making a decision of definitive discontinuation of the IMP for the concerned participant.

Handling of participants after definitive intervention discontinuation

Participants will be followed-up according to the study procedures specified in this protocol up to the scheduled date of study completion, or up to recovery or stabilization of any AE to be followed-up as specified in this protocol, whichever comes last.

Participants who discontinue the study intervention prematurely (prior to completing the 24-week treatment period) will perform, as soon as possible, the early termination visit with all assessments normally planned for the EOT Visit (Visit 6), to assure a complete clinical assessment in close temporal proximity to the premature termination of study treatment is available.

In addition, and to allow assessment of participant outcomes over the stipulated study period, patients will be asked and encouraged to complete all remaining study treatment visits, and participate in all assessments according to the visit schedule with a ±3 day window. Under exceptional circumstances when a participant cannot come to the site for a scheduled visit, a phone contact can be made. During the phone contact, at least information about AEs, concomitant medication and status of PN should be collected.

All cases of definitive intervention discontinuation must be recorded by the Investigator in the appropriate pages of the eCRF when considered as confirmed.

7.1.2 Temporary discontinuation

Temporary intervention discontinuation may be considered by the Investigator because of suspected AEs.

In addition, the following conditions will be causes for temporary discontinuation of study intervention:

- Infections or infestations that do not respond to medical treatment
- Any laboratory abnormality that meets temporary treatment discontinuation criteria as per Section 10.6

Temporary intervention discontinuation decided by the Investigator corresponds to more than 1 dose not administered to the participant. Following a temporary interruption or missed dose, the treatment should be reinitiated at the next scheduled administration, maintaining the planned dose. If more than 2 consecutive IMP doses have been missed, the study treatment will be permanently discontinued (see Section 7.1.1).

7.1.2.1 Rechallenge

Reinitiation of intervention with the IMP will be done under close and appropriate clinical/and or laboratory monitoring once the Investigator will have considered according to his/her best medical judgment that the responsibility of the IMP(s) in the occurrence of the concerned event was unlikely and if the selection criteria for the study are still met (refer to Section 5).

7.2 PARTICIPANT DISCONTINUATION/WITHDRAWAL FROM THE STUDY

- A participant may withdraw from the study at any time at his/her own request, or may be withdrawn at any time at the discretion of the Investigator for safety, behavioral, compliance, or administrative reasons. This is expected to be uncommon.
- At the time of discontinuing from the study, if possible, an early discontinuation visit should be conducted, as shown in the SoA (Section 1.3). See SoA for data to be collected at the time of study discontinuation and follow-up and for any further evaluations that need to be completed.
- The participant will be permanently discontinued both from the study intervention and from the study at that time.
- If the participant withdraws consent for disclosure of future information, the Sponsor may retain and continue to use any data collected before such a withdrawal of consent.
- If a participant withdraws from the study, he/she may request destruction of any samples taken and not tested, and the Investigator must document this in the site study records.

If participants no longer wish to take the IMP, they will be encouraged to remain in the study.

The Investigators should discuss with them key visits to attend. The value of all their study data collected during their continued involvement will be emphasized as important to the public health value of the study.

Participants who withdraw from the study intervention should be explicitly asked about the contribution of possible AEs to their decision, and any AE information elicited must be documented.

All study withdrawals should be recorded by the Investigator in the appropriate screens of the eCRF and in the participant's medical records. In the medical record, at least the date of the withdrawal and the reason should be documented.

In addition, a participant may withdraw his/her consent to stop participating in the study. Withdrawal of consent for intervention should be distinguished from withdrawal of consent for follow-up visits and from withdrawal of consent for non-participant contact follow-up, eg, medical record checks. The site should document any case of withdrawal of consent.

Participants who have withdrawn from the study cannot be rerandomized (treated) in the study. Their inclusion and intervention numbers must not be reused.

7.3 LOST TO FOLLOW UP

A participant will be considered lost to follow-up if he or she repeatedly fails to return for scheduled visits and is unable to be contacted by the study site.

The following actions must be taken if a participant fails to return to the clinic for a required study visit:

- The site must attempt to contact the participant and reschedule the missed visit as soon as possible and counsel the participant on the importance of maintaining the assigned visit schedule and ascertain whether or not the participant wishes to and/or should continue in the study.
- Before a participant is deemed lost to follow up, the Investigator or designee must make every effort to regain contact with the participant (where possible, 3 telephone calls and, if necessary, a certified letter to the participant's last known mailing address or local equivalent methods). These contact attempts should be documented in the participant's medical record.
- Should the participant continue to be unreachable, he/she will be considered to have withdrawn from the study.
- Discontinuation of specific sites or of the study as a whole are handled as part of Section 10.1.

8 STUDY ASSESSMENTS AND PROCEDURES

- Study procedures and their timing are summarized in the SoA (see Section 1.3). Protocol waivers or exemptions are not allowed.
- Adherence to the study design requirements, including those specified in the SoA (see Section 1.3), is essential and required for study conduct.
- All screening evaluations must be completed and reviewed to confirm that potential participants meet all eligibility criteria. The Investigator will maintain a screening log to record details of all participants screened and to confirm eligibility or record reasons for screening failure, as applicable.
- Procedures conducted as part of the participant's routine clinical management (eg, blood count) and obtained before signing of the ICF may be utilized for screening or baseline purposes provided the procedures met the protocol-specified criteria and were performed within the time frame defined in the SoA (see Section 1.3).
- Patient-Reported Outcome questionnaires including NRS should be completed by the participants before the consultation and/or clinical tests, in a quiet place. The questionnaires should be completed by the participants themselves, independently from their physician, the study nurse or any other medical personnel and without any help from friends or relatives.
- In light of the public health emergency related to Coronavirus disease 2019 (COVID-19) (or in case of any other pandemic requiring public health emergency), the continuity of clinical study conduct and oversight may require implementation of temporary or alternative mechanisms eg, phone contact, virtual visits, online meetings, use of local clinic or laboratory locations, and home visits by skilled staff. Implementation of such mechanisms may differ country by country, depending on country regulations and local business continuity plans. Additionally, no waivers to deviate from protocol enrollment criteria due to COVID-19 (or any other pandemic) will be granted. All temporary mechanisms utilized, and deviations from planned study procedures are to be documented as being related to COVID-19 (or any other pandemic) and will remain in effect only for the duration of the public health emergency.

8.1 EFFICACY ASSESSMENTS

Efficacy data will be collected via electronic devices.

The e-diary is used for daily recording of participant's answers to the WI-NRS, pain-NRS, and sleep-NRS questionnaires. This device will be dispensed at screening visit (Visit 1), including instructions for use. Participants will be instructed on the use of the device.

Recorded information will be downloaded from this device daily. At EOS Visit, the e-diary will be downloaded and returned to the site.

On regular basis, the site staff should review on vendor's website the information downloaded from participants' e-diary. They should particularly check status of the disease reviewing

WI-NRS, as well as compliance to background therapy and overall e-diary compliance. The site should follow up with the participant as appropriate.

Participants will fill in the DLQI, HADS, EQ-5D-5L, PGIC, PGIS, and missed school/work days questionnaires during their site visit on a tablet that will be provided to the site. This device is kept at the site during the study.

8.1.1 Worst-itch numeric rating scale

Worst-itch numerical rating scale (WI-NRS) is a PRO comprised of a single item rated on a scale from 0 (“No itch”) to 10 (“Worst imaginable itch”). Participants are asked to rate the intensity of their worst pruritus (itch) over the past 24 hours using this scale.

Participants will complete the WI-NRS daily according to the SoA (see Section 1.3). The assessment tool is provided in Section 10.7.1.

8.1.2 Investigator’s global assessment for prurigo nodularis

Investigator’s global assessment for prurigo nodularis (IGA PN) is a clinician-reported outcome (ClinRO) that allows clinicians to assess the activity of PN (IGA PN-A) using a 5-point scale from 0 (clear) to 4 (severe); and the stage of the disease (IGA PN-S) using a 5-point scale from 0 (clear) to 4 (severe).

Clinicians will complete the IGA PN as described in the SoA (see Section 1.3). The assessment tool is provided in Section 10.7.2.

8.1.3 Prurigo activity score

The PAS is a ClinRO measurement. The original PAS questionnaire Version 0.9 consists of 7 items, developed by expert clinicians in PN (25). The items of the PAS evaluate the pruriginous lesions in terms of:

- Type (visible lesions: Item 1a; predominant lesions: Item 1b);
- Estimated number (Item 2);
- Distribution (Item 3, 4);
- Size (biggest lesion: Item 6a; representative lesion: Item 6b).

Other items evaluate the representative body area and exact number of lesions (Item 5), the activity in terms of percentage of pruriginous lesions with excoriations/crusts on top (reflecting active scratching; Item 7a) and the percentage of healed pruriginous lesions (reflecting healing of chronic prurigo; Item 7b).

A 5-item simplified version of the PAS will be used in the current study. In particular, Item 3 (lesion distribution) and Item 6 (lesion monitoring) of the original PAS were removed, and the response options slightly modified and refined.

Clinicians will complete the screening/baseline version at screening and baseline visits, and will complete the follow-up version of the modified PAS at the other visits, as reported in the SoA (see Section 1.3). The assessment tool is provided in Section 10.7.3.

8.1.4 Dermatology life quality index

The DLQI is a PRO developed to measure dermatology-specific HRQoL in adult patients (27). The instrument comprises 10 items assessing the impact of skin disease on patients' HRQoL over the previous week. The items cover symptoms, leisure activities, work/school or holiday time, personal relationships including intimate, the side effects of treatment, and emotional reactions to having a skin disease. It is a validated questionnaire used in clinical practice and clinical trials (26). Response scale is a 4-point Likert scale (0 = "not at all" and 3 = "very much") for nine items. The remaining one item about work/studying asks whether work/study has been prevented and then (if "No") to what degree the skin condition has been a problem at work/study; the item is rated on a 3-point Likert scale ("Not at all" to "A lot"). Overall scoring ranges from 0 to 30, with a high score indicative of a poor HRQoL.

Participants will complete the questionnaire at time points according to SoA (see Section 1.3). The assessment tool is provided in Section 10.7.4.

8.1.5 Pain and sleep numeric rating scales

Participants will be asked to rate their worst skin pain in the past 24 hours using a 0 to 10 NRS, with 0 = No pain to 10 = Worst pain possible.

In addition, participants will be asked to rate their sleep quality on their past night upon awakening, using a 0 to 10 NRS, with 0 = Worst possible sleep and 10 = Best possible sleep.

Participants will complete the skin pain NRS and sleep quality NRS once a day.

The assessment tools are provided in Section 10.7.7 and Section 10.7.8.

8.1.6 Hospital anxiety and depression scale

The HADS is a PRO instrument for screening anxiety and depression in non-psychiatric populations; repeated administration also provides information about changes to a patient's emotional state (28, 29). The HADS consists of 14 items, 7 each for anxiety and depression symptoms; possible scores range from 0 to 21 for each subscale. The following cut-off scores are recommended for both subscales:

- 0 to 7: normal
- 8 to 10: borderline abnormal (borderline case)
- 11 to 21: abnormal

Participants will complete the questionnaire as described in the SoA (see Section 1.3). The assessment tool is provided in Section 10.7.5.

8.1.7 Patient Global Impression of Change of disease and Patient Global Impression of Severity

The PGIC is a one-item questionnaire that asks patients to provide the overall self-assessment of change in their PN overall on a 7-point scale, compared to just before patient started taking the study injection. Response choices are: 0 = “Very much better”, 1 = “Moderately better”, 2 = “A little better”, 3 = “No change”, 4 = “A little worse”, 5 = “Moderately worse”, 6 = “Very much worse”.

The PGIS is a one-item questionnaire that asks patients to provide the overall self-assessment of their disease severity on a 4-point scale for the past week. Response choices are: 1 = “none”, 2 = “Mild”, 3 = “Moderate”, 4 = “Severe”.

Participants will complete the 2 items as described in the SoA (see Section 1.3). The items are provided in Section 10.7.9 and Section 10.7.10.

8.1.8 Euroqol 5 dimensions questionnaire

The Euroqol-5 dimensions (EQ-5D) is a standardized PRO measure of health status developed by the EuroQOL Group in order to provide a simple, generic measure of health for clinical and economic appraisal (30). The EQ-5D consists of 2 parts: the descriptive system and the EQ visual analog scale (VAS). The EQ-5D descriptive system comprises the following 5 dimensions: mobility, self-care, usual activities, pain/discomfort, and anxiety/depression. Each dimension has 5 levels of perceived problems: “no problem”, “slight problems”, “moderate problems”, “severe problems” and “inability to do the activity”. The respondent is asked to indicate his/her health state by ticking (or placing a cross) in the box against the most appropriate statement in each of the 5 dimensions; this results in a 1-digit number expressing the level for that dimension. The digits for 5 dimensions can be combined in a 5-digit number describing the respondent’s health state. The EQ VAS records the respondent’s self-rated health on a vertical VAS where the endpoints are labeled “best imaginable health state (100)” and “worst imaginable health state (0)”. This information can be used as a quantitative measure of health outcome as judged by the individual respondents.

Participants will complete the questionnaire as described in the SoA (see Section 1.3). The assessment tool is provided in Section 10.7.6.

8.1.9 Missed school/work days

Participants who are employed or enrolled in school will be asked to report the number of sick leave/missed school days since the last study assessment.

Participants will complete the questionnaire as described in the SoA (see Section 1.3). The assessment tool is provided in Section 10.7.11.

8.2 SAFETY ASSESSMENTS

Planned time points for all safety assessments are provided in the SoA (see Section 1.3).

8.2.1 Physical examinations

- A complete physical examination will include, at a minimum, assessments of the skin (full body skin exam), nasal cavities, eyes, ears, respiratory, cardiovascular, gastrointestinal, neurological, lymphatic, and musculoskeletal systems.
- Participants must be disrobed and provided with a hospital gown before the skin examination.
- Investigators should pay special attention to clinical signs related to previous serious illnesses.
- Any new finding or worsening of previous finding should be reported as a new AE.

8.2.2 Vital signs

- Vital signs will be measured in a semi-supine or sitting position after 5 minutes rest and will include body temperature, SBP and DBP, and pulse and respiratory rate. Blood pressure and pulse measurements should be assessed using the same arm with a completely automated device. Manual techniques will be used only if an automated device is not available.
- Body weight (kg) will be measured at screening (Visit 1), EOT, and EOS visits. Height will be measured at screening visit (Visit 1). Height and weight should be measured with indoor clothing but without shoes.

8.2.3 Electrocardiograms

- Single 12-lead ECG will be obtained as outlined in the SoA (see Section 1.3) using an ECG machine that automatically calculates the heart rate and measures PR, QRS, QT, and QTcF intervals. Refer to Section 7 for QTcF withdrawal criteria and any additional QTcF readings that may be necessary. The ECG should be recorded after 10 minutes of rest in the supine position.

8.2.4 Clinical safety laboratory assessments

- See Appendix 2 (Section 10.2) for the list of clinical laboratory tests to be performed and to the SoA (see Section 1.3) for the timing and frequency.
- The Investigator must review the laboratory report, document this review, and record any clinically relevant changes occurring during the study in the AE section of the CRF. The laboratory reports must be filed with the source documents. Clinically significant abnormal laboratory findings are those which are not associated with the underlying disease, unless judged by the Investigator to be more severe than expected for the participant's condition.
- All laboratory tests with values considered clinically significantly abnormal during participation in the study or within 12 weeks after the last dose of study intervention

should be repeated until the values return to normal or baseline or are no longer considered clinically significant by the Investigator or medical monitor.

- If such values do not return to normal/baseline within a period of time judged reasonable by the Investigator, the etiology should be identified and the Sponsor notified.
- All protocol-required laboratory assessments, as defined in Appendix 2 (Section 10.2), must be conducted in accordance with the laboratory manual and the SoA (see Section 1.3).
- If laboratory values from non-protocol specified laboratory assessments performed at the institution's local laboratory require a change in participant management or are considered clinically significant by the Investigator (eg, SAE or AE or dose modification), then the results must be recorded in the CRF.

8.3 ADVERSE EVENTS AND SERIOUS ADVERSE EVENTS

Adverse event of special interest

An AESI is an AE (serious or nonserious) of scientific and medical concern specific to the Sponsor's product or program, for which ongoing monitoring and immediate notification by the Investigator to the Sponsor is required. Such events may require further investigation in order to characterize and understand them. Adverse events of special interest may be added, modified or removed during a study by protocol amendment.

For these AESIs, the Sponsor will be informed immediately (ie, within 24 hours), per SAE notification described in Section 10.3, even if not fulfilling a seriousness criterion, using the corresponding pages in the CRF (to be sent) or screens in the eCRF. If an SAE or any AESI of anaphylactic reactions or systemic allergic reactions that are related to IMP and require treatment, or severe injection site reactions lasting longer than 24 hours, occurs in a participant, blood samples should be collected for determination of functional dupilumab concentration, and ADA assessment at or near the onset and completion of the occurrence of the event, if possible.

- Anaphylactic reactions
- Systemic hypersensitivity reactions
- Helminthic infections
- Any severe type of conjunctivitis or blepharitis
- Keratitis
- Clinically symptomatic eosinophilia (or eosinophilia associated with clinical symptoms)
- Pregnancy of a female subject entered in a study as well as pregnancy occurring in a female partner of a male subject entered in a study with IMP/NIMP;
    - Pregnancy occurring in a female participant entered in the clinical trial or in a female partner of a male participant entered in the clinical trial. It will be qualified as an SAE only if it fulfills one of the seriousness criteria.

- In the event of pregnancy in a female participant, IMP should be discontinued.
- Follow-up of the pregnancy in a female participant or in a female partner of a male participant is mandatory until the outcome has been determined.
- Abnormal pregnancy outcomes (eg, spontaneous abortion, fetal death, stillbirth, congenital anomalies, ectopic pregnancy) are considered SAEs.

- Significant ALT elevation
  - ALT >5 x ULN in participants with baseline ALT ≤2 x ULN;

    OR
  - ALT >8 x ULN if baseline ALT >2 x ULN
- Symptomatic overdose (serious or nonserious) with IMP/NIMP
  - An overdose (accidental or intentional) with the IMP is an event suspected by the Investigator or spontaneously notified by the participant and defined as at least twice the intended dose during an interval of less than 11 days. The circumstances (ie, accidental or intentional) should be clearly specified in the verbatim and symptoms, if any, entered on separate AE forms.
  - An overdose (accidental or intentional) with any NIMP is an event suspected by the Investigator or spontaneously notified by the participant and defined as at least twice the maximum prescribed daily dose, within the intended therapeutic interval. The circumstances (ie, accidental or intentional) should be clearly specified in the overdose form.

The definitions of an AE or SAE can be found in Appendix 3 (Section 10.3).

Adverse events will be reported by the participant (or, when appropriate, by a caregiver, surrogate, or the participant's legally authorized representative).

The Investigator and any qualified designees are responsible for detecting, documenting, and recording events that meet the definition of an AE or SAE and remain responsible for following up AEs that are serious, considered related to the study intervention or study procedures, or that caused the participant to discontinue the study intervention (see Section 7).

8.3.1 Time period and frequency for collecting AE and SAE information

All AEs, serious or nonserious, will be collected from the signing of the ICF until the EOS visit at the time points specified in the SoA (Section 1.3).

All SAEs and AESI will be recorded and reported to the Sponsor or designee immediately and under no circumstance should this exceed 24 hours, as indicated in Appendix 3 (Section 10.3). The Investigator will submit any updated SAE data to the Sponsor within 24 hours of it being available.

Investigators are not obligated to actively seek AE or SAE after conclusion of the study participation. However, if the Investigator learns of any SAE, including a death, at any time after a participant has been discharged from the study, and he/she considers the event to be reasonably related to the study intervention or study participation, the Investigator must promptly notify the Sponsor.

8.3.2 Method of detecting AEs and SAEs

The method of recording, evaluating, and assessing causality of AE and SAE and the procedures for completing and transmitting SAE reports are provided in Appendix 3 (Section 10.3).

Care will be taken not to introduce bias when detecting AEs and/or SAEs. Open-ended and non-leading verbal questioning of the participant is the preferred method to inquire about AE occurrences.

8.3.3 Follow-up of AEs and SAEs

After the initial AE/AESI/SAE report, the Investigator is required to proactively follow each participant at subsequent visits/contacts. At the pre-specified study end-date, all SAEs and nonserious AESI (as defined in Section 8.3) will be followed until resolution, stabilization, the event is otherwise explained, or the participant is lost to follow-up (as defined in Section 7.3). Further information on follow-up procedures is given in Appendix 3 (Section 10.3).

8.3.4 Regulatory reporting requirements for SAEs

- Prompt notification by the Investigator to the Sponsor of a SAE is essential so that legal obligations and ethical responsibilities towards the safety of participants and the safety of a study intervention under clinical investigation are met.
- The Sponsor has a legal responsibility to notify both the local regulatory authority and other regulatory agencies about the safety of a study intervention under clinical investigation. The Sponsor will comply with country-specific regulatory requirements relating to safety reporting to the regulatory authority, Institutional Review Boards (IRB)/Independent Ethics Committees (IEC), and Investigators.
- Investigator safety reports must be prepared for suspected unexpected serious adverse reactions (SUSAR) according to local regulatory requirements and Sponsor policy and forwarded to Investigators as necessary.
- Adverse events that are considered expected will be specified in the reference safety information.
- An Investigator who receives an Investigator safety report describing a SAE or other specific safety information (eg, summary or listing of SAEs) from the Sponsor will review and then file it along with the IB and will notify the IRB/IEC, if appropriate according to local requirements.

8.3.5 Pregnancy

- Details of all pregnancies in female participants and female partners of male participants will be collected after the start of study intervention and until the outcome has been determined.

- If a pregnancy is reported, the Investigator should inform the Sponsor within 24 hours of learning of the pregnancy and should follow the procedures outlined in Appendix 4 (Section 10.4).
- Abnormal pregnancy outcomes (eg, spontaneous abortion, fetal death, stillbirth, congenital anomalies, ectopic pregnancy) are considered SAEs.

8.3.6 Guidelines for reporting product complaints

Any defect in the IMP/NIMP/device must be reported as soon as possible by the Investigator to the monitoring team that will complete a product complaint form within required timelines.

Appropriate information (eg, samples, labels or documents like pictures or photocopies) related to product identification and to the potential deficiencies may need to be gathered. The Investigator will assess whether or not the quality issue has to be reported together with an AE or SAE.

8.4 TREATMENT OF OVERDOSE

Overdose is an AESI (defined in Section 8.3). No antidote is available for dupilumab. Sponsor does not recommend specific treatment for an overdose.

In the event of an overdose, the Investigator/treating physician should:

1. Provide symptomatic care.
2. Contact the Medical Monitor immediately.
3. Closely monitor the participant for any AE/SAE and laboratory abnormalities until dupilumab can no longer be detected systemically.
4. Obtain a plasma sample for PK analysis as soon as possible if requested by the Medical Monitor (determined on a case-by-case basis).
5. Document appropriately in the CRF.

Decisions regarding dose interruptions will be made by the Investigator in consultation with the Medical Monitor based on the clinical evaluation of the participant.

8.5 PHARMACOKINETICS

8.5.1 Systemic drug concentration and antidrug antibodies

8.5.1.1 Sampling time

Blood samples will be collected for determination of functional dupilumab and anti-dupilumab antibodies in serum as specified in the SoA (Section 1.3). Special procedures for collection, storage, and shipping of serum are described in separate operational manuals. The date of collection should be recorded in the eCRF.

8.5.1.2 Handling procedures

Special procedures for collection, storage, and shipping of serum are described in separate operational manuals. An overview of handling procedure for samples used in the determination of systemic drug concentration and ADA is provided in Table 3.

Table 3 - Summary of handling procedures

Sample type	Functional dupilumab	Anti-dupilumab antibody
Matrix	Serum	Serum
Blood sample volume	5 mL	5 mL
Anticoagulant	None	None
Blood handling procedures	See Operational Manual	See Operational Manual
Serum aliquot split	2 aliquots	2 aliquots
Storage conditions	<6 months: below -20°C<br><24 months: below -80°C (preferred)	<6 months: below -20°C<br><24 months: below -80°C (preferred)
Serum shipment condition	In dry ice	In dry ice

8.5.1.3 Bioanalytic method

Serum PK and ADA samples will be assayed using validated methods as described in Table 4.

[redacted]

Note: In the event of any SAE, any AE of severe injection site reaction lasting longer than 24 hours, or any AESI of anaphylactic reaction or systemic allergic reaction that is related to IMP and require treatment, PK and ADA samples will be collected at or near the onset of the event for any additional analysis if required or for archival purposes. The exact date and time of sample collection must be recorded and entered into the database by the central laboratory. An unscheduled systemic drug concentration page in the eCRF must be completed as well. If necessary for safety monitoring, additional PK and ADA samples may be collected after the EOS Visit until resolution of AE.

Specifically for PK, any changes in the timing or addition of time points for any planned study assessments must be documented and approved by the relevant study team member and then archived in the Sponsor and site study files, but will not constitute a protocol amendment. The IRB/IEC will be informed of any safety issues that require alteration of the safety monitoring scheme or amendment of the ICF.

8.6 PHARMACODYNAMICS

See Section 8.8 for IgE measurement. No other PD parameters will be evaluated in this study.

8.7 GENETICS

For those participants who consent to the optional pharmacogenetic/pharmacogenomic sample collection section of the ICF, blood samples for exploratory genetic analysis of DNA or RNA will be collected and stored for possible future use (see Section 10.5). Participation is optional. Participants who do not wish to participate in the genetic research may still participate in the study.

In the event of DNA extraction failure, a replacement genetic blood sample may be requested from the participant.

Details on processes for collection and shipment and destruction of these samples can be found in the laboratory manual.

8.8 BIOMARKERS

- Venous blood samples will be collected for measurement of total serum IgE. Total IgE will be measured using validated quantitative methods.
- Optional samples for biomarker research that should be collected from participants in the study with their consent include:
  - Serum and plasma: samples will be collected and stored for possible future analysis of potential biomarkers of drug response, disease activity, safety, and Type 2 inflammation.

Plasma and serum samples may be stored for a maximum of 5 years, and DNA or RNA samples for 15 years (or according to local regulations) following the last participant's last visit for the study at a facility selected by the Sponsor.

8.9 MEDICAL RESOURCE UTILIZATION AND HEALTH ECONOMICS

Number of missed school/work days collection is described in Section 8.1.9.

9 STATISTICAL CONSIDERATIONS

9.1 STATISTICAL HYPOTHESES

The statistical hypotheses for comparing dupilumab to placebo on the primary endpoint of the proportion of participants with WI-NRS reduction of ≥4 (minimally important difference) at Week 12 are as follows:

- Null hypothesis H0: No treatment difference between dupilumab and placebo
- Alternative hypothesis H1: There is a treatment difference between dupilumab and placebo

The statistical hypotheses to be tested on secondary endpoints can be specified similarly to those on the primary endpoint.

9.2 SAMPLE SIZE DETERMINATION

The primary endpoint is the proportion of participants with WI-NRS reduction of ≥4 from baseline to Week 12. By assuming the response rate is [redacted] in placebo and dupilumab respectively, 56 participants/arm will provide 90% power to detect the difference of [redacted] between dupilumab and placebo with Fisher exact test at 2-sided level of 0.05. Assuming 15% drop out during the 12 weeks of treatment, the target is to randomize 75 participants/arm with a cap of up to 10% of participants in the atopic population having active mild AD.

Approximately 150 participants will be randomized 1:1. This corresponds to approximately 75 participants that will be randomly assigned to each intervention arm.

The sample size calculation was performed using SAS 9.4 power procedure. The assumptions were based on the effect of dupilumab versus placebo in WI-NRS reduction ≥4 at Week 16 observed in patients with moderate to severe AD as seen in studies of R668-AD-1334 (Solo1) and R668-AD-1416 (Solo2).

Participants will be randomized to dupilumab or placebo in 1:1 ratio with stratification factors of documented history of atopy (atopic or non-atopic, see definition in Section 6.3), stable use of TCS/TCI (yes or no), and country/territory code. Both the atopic and the non-atopic PN population will be capped at 60% of the total enrolled population.

9.3 POPULATIONS FOR ANALYSES

For purposes of analysis, the following populations are defined (Table 5):

Table 5 - Populations for analyses

Population	Description
Screened	All participants who sign the ICF.
Randomized	The randomized population includes all participants with a treatment kit number allocated and recorded in the IRT database, and regardless of whether the treatment kit was used or not.<br>Participants treated without being randomized will not be considered randomized and will not be included in any efficacy population.
Intent-to-treat (ITT)	All randomized participants analyzed according to the treatment group allocated by randomization regardless if treatment kit is used or not
Efficacy	The ITT population
Safety	All participants randomly assigned to study intervention and who take at least 1 dose of study intervention. Participants will be analyzed according to the intervention they actually received.<br>Randomized participants for whom it is unclear whether they took the study medication will be included in the safety population as randomized.<br>For participants who accidentally receive different treatment from the planned, the actual intervention allocation for as-treated analysis will be the dupilumab group.<br>The PD analyses will be performed on the safety population.
Pharmacokinetic (PK)	The PK population includes all participants in the safety population with at least one non-missing result for functional dupilumab concentration in serum after first dose of the study treatment. Participants will be analyzed according to the intervention actually received.
Antidrug antibody (ADA)	ADA population includes all participants in the safety population who have at least one non-missing ADA result after first dose of the study treatment. Participants will be analyzed according to the intervention actually received.

ADA: antidrug antibody; ICF: informed consent form; ITT: intent to treat; PD: pharmacodynamics: PK: pharmacokinetic

9.4 STATISTICAL ANALYSES

The SAP will be developed and finalized before primary database lock and will describe the participant populations to be included in the analyses, and procedures for accounting for missing, unused, and spurious data. This section is a summary of the planned statistical analyses of the primary and secondary endpoints.

9.4.1 Efficacy analyses

The multiplicity procedure is proposed to control the overall Type-I error rate for testing the primary endpoint and the key secondary endpoints. Detailed hierarchical testing procedure will be defined in the study SAP. The study is considered positive when the primary endpoint achieves statistical significance with two-sided significance level 0.05.

Table 6 - Efficacy analyses

Endpoint	Statistical analysis methods
**Primary**:	
Proportion of participants with improvement (reduction) in WI-NRS by ≥4 from baseline to Week 12	The primary analysis will be conducted by using CMH test stratifying by stratification factors (documented history of atopy [atopic or non-atopic], stable use of TCS/TCI [yes or no], and region [countries combined]) and covariate of baseline anti-depressant use (yes or no).<br>For participants discontinuing the study treatment before Week 12, their off-study treatment values measured up to Week 12 will be included in the analysis. Participants taking selected prohibited medications and/or rescue medications (details of selection will be provided in the SAP) prior to Week 12 or have missing data at Week 12 will be considered non-responders.<br>*Sensitivity analyses:*<br>The data collected after taking selected prohibited medications and/or rescue medications will not be censored and included in the sensitivity analysis to evaluate the robustness of the primary analysis results with respect to the method of handling data while taking selected prohibited medications. Details of the sensitivity analyses will be provided in the SAP.<br>*Subgroup analyses:*<br>To assess the consistency in treatment effects across different subgroup levels, subgroup analyses will be performed for the primary efficacy endpoint with respect to documented history of atopy (atopic or non-atopic), age group, gender, region, and other factors that will be specified in the SAP. A subgroup analysis will be performed excluding participants with a current diagnosis of AD.
**Key secondary:**<br>Proportion of participants with improvement (reduction) in WI-NRS by ≥4 from baseline to Week 24.<br>Proportion of participants with IGA PN-S 0 or 1 score at Week 24.	Key secondary efficacy endpoints that measure binary responses will be analyzed in the same fashion as the primary endpoint.
**Secondary:**	
Proportion of participants with WI-NRS reduction ≥4 over time until Week 24.<br>Proportion of participants with IGA PN-S 0 or 1 score at Week 12.<br>Proportion of participants with IGA PN-S 0 or 1 score at Week 8.<br>Proportion of participants with IGA PN-S 0 or 1 score at Week 4.<br>Proportion of participants with IGA PN-A 0 or 1 score at Week 24.<br>Proportion of participants with IGA PN-A 0 or 1 score at Week 12.<br>Proportion of participants with IGA PN-A 0 or 1 score at Week 8.<br>Proportion of participants with IGA PN-A 0 or 1 score at Week 4.<br>Proportion of participants with WI-NRS reduction ≥4 at Week 4.	Secondary efficacy endpoints that measure binary responses will be analyzed in the same fashion as the primary endpoint.

Endpoint	Statistical analysis methods
Time to onset of effect on pruritus as measured by proportion of participants with improvement (reduction) in WI-NRS by ≥4 from baseline during the 24-week treatment period.	This time-to-event endpoint will be analyzed using the Cox proportional hazards model, including treatment, stratification factors (countries combined to region), and covariate of baseline anti-depressant use (yes or no). The hazards ratio, its 95% confidence interval and p-value will be reported. Kaplan-Meier curves will be also provided.
Change from baseline in WI-NRS at Week 24.<br>Change from baseline in WI-NRS at Week 12.<br>Percent change from baseline in WI-NRS at Week 24.<br>Percent change from baseline in WI-NRS at Week 12.<br>Percent change from baseline in WI-NRS at Week 4.<br>Percent change from baseline in WI-NRS at Week 2.<br>Percent change from baseline in WI-NRS over time until Week 24.<br>Change from baseline in IGA PN-S score at Week 24.<br>Change from baseline in IGA PN-S score at Week 12.<br>Change from baseline in IGA PN-S score at Week 8.<br>Change from baseline in IGA PN-S score at Week 4.<br>Change from baseline in HRQoL, as measured by DLQI to Week 24.<br>Change from baseline in HRQoL, as measured by DLQI to Week 12.<br>Onset of action in change from baseline in WI-NRS (first $p < 0.05$ difference from placebo in the daily WI-NRS that remains significant at subsequent measurements) until Week 12.	Continuous secondary efficacy endpoints will be analyzed using a hybrid method of the WOCF and multiple imputations.<br>Data of participants taking selected prohibited medications and/or rescue medications will be set to missing after the medication usage, and the worst postbaseline value on or before the time of the medication usage will be used to impute missing endpoint value (for participants whose postbaseline values are all missing, the baseline will be used to impute).<br>Participants who discontinue the treatment prematurely are encouraged to follow the planned clinical visits and in those participants who did not take the selected prohibited medications and/or rescue medications, all data collected after treatment discontinuation will be used in the analysis. For these participants, missing data may still happen despite all efforts have been tried to collect the data after treatment discontinuation. For participants who discontinue due to lack of efficacy, all data collected after discontinuation will be used in the analysis, and a WOCF approach will be used to impute missing data if needed. For participants who discontinued not due to lack of efficacy, a multiple imputation approach will be used to impute missing endpoint value, and this multiple imputation will use all participants excluding patients who have taken the selected prohibited medications and/or rescue medications prior to timepoint of endpoint of interest and excluding participants who discontinue due to lack of efficacy.<br>Each of the imputed complete data will be analyzed by fitting an ANCOVA model with treatment group, stratification factors (documented history of atopy [atopic or non-atopic], stable use of TCS/TCI [yes or no], and region [countries combined]), baseline antidepressant use (yes or no), and relevant baseline measurement as covariates included in the model. Statistical inference obtained from all imputed data will be combined using Rubin's rule. Descriptive statistics including number of subjects, mean, standard error, and least squares (LS) mean change and standard error will be provided. In addition, difference of the dupilumab group against placebo in LS means and the corresponding 95% confidence interval will be provided along with the p-values.<br>Detailed analyses will be described in the SAP finalized before database lock. The sensitivity analyses will be also detailed in the SAP to evaluate the robustness of the hybrid method of the WOCF and multiple imputations handling intercurrent events such as taking prohibited medication, rescue medication, or missing data due to study treatment withdrawal.
<u>**Tertiary/exploratory**</u>	Will be described in the SAP finalized before database lock.

ANCOVA: analysis of covariance; CMH: Cochran-Mantel-Haenszel; DLQI: Dermatology Life Quality Index; IGA: Investigator's global assessment; LS: Least squares; NRS: numeric rating scale; PAS: prurigo activity score; WI-NRS: worst itch numeric rating scale.

9.4.2 Safety analyses

All safety analyses will be performed on the Safety Population. The summary of safety results will be presented by treatment group. The baseline value is defined generally as the last available value before randomization.

Table 7 - Safety analyses

Endpoint	Statistical analysis methods
Percentage of participants experiencing TEAEs or SAEs from baseline through Week 24	Adverse event incidence tables will present by SOC (sorted by internationally agreed order), and PT sorted in alphabetical order for each treatment group, the number (n) and percentage (%) of participants experiencing an AE. Multiple occurrences of the same event in the same participant will be counted only once in the tables within a treatment phase. The denominator for computation of percentages is the safety population within each treatment group.<br>Proportion of participants with at least one TEAE, treatment emergent SAE, TEAE leading to death, and TEAE leading to permanent treatment discontinuation will be tabulated by treatment group. In addition TEAEs will be described according to maximum intensity and relation to the study intervention. Serious AEs and AEs leading to study discontinuation that occur outside the treatment-emergent period will be summarized separately.

AE: adverse event; PT: preferred term; SOC: system organ class; SAE: serious adverse event; TEAE: treatment-emergent adverse event

9.4.3 Other analyses

Table 8 - Other analyses

Endpoint	Statistical analysis methods
Incidence of treatment-emergent ADA against dupilumab over time	The ADA analysis will be conducted on ADA population. ADA variables including treatment-emergent ADA will be summarized using descriptive statistics by treatment group. Drug concentration data will be examined and the influence of ADAs on individual concentration-time profiles will be evaluated. Assessment of the potential impact of ADA on safety and efficacy may be provided.

ADA: antidrug antibodies

Other PK, PD, biomarker, and outcome measures exploratory analyses will be described in the SAP finalized before database lock. The population PK analysis and PD analyses will be presented separately from the main clinical study report (CSR).

Data collected regarding the impact of the COVID-19 or other pandemics, on the patients will be summarized (eg, discontinuation due to COVID-19). Any additional analyses and methods required to investigate the impact of COVID-19 or other pandemics requiring public health emergency on the efficacy (eg; missing data due to COVID-19) and safety will be detailed in the SAP.

9.5 INTERIM ANALYSES

No interim analysis is planned.

A primary database lock will be performed when all randomized participants in this study have completed their 24-week treatment phase. Final analyses in the CSR will be based on this database.

The database will be updated at the end of the study for all participants to include the post-treatment follow-up information and updates for the events previously ongoing at the time of

the primary lock. Additional data between this database lock and last participant completing last visit will be summarized in a CSR addendum.

More details will be described in the SAP.

9.5.1 Data Monitoring Committee (DMC)

Due to extensive safety record of the post marketed IMP (dupilumab), it is not planned to have a DMC for this study.

9.5.2 Unblinding plan

Unblinding plan is not applicable for this study.

10 SUPPORTING DOCUMENTATION AND OPERATIONAL CONSIDERATIONS

10.1 APPENDIX 1: REGULATORY, ETHICAL, AND STUDY OVERSIGHT CONSIDERATIONS

10.1.1 Regulatory and Ethical Considerations

- This study will be conducted in accordance with the protocol and with the following:
  - Consensus ethical principles derived from international guidelines including the Declaration of Helsinki and the applicable amendments and Council for International Organizations of Medical Sciences (CIOMS) International Ethical Guidelines.
  - Applicable ICH GCP Guidelines.
  - Applicable laws and regulations.
- The protocol, protocol amendments, ICF, Investigator Brochure, and other relevant documents (eg, advertisements) must be submitted to an IRB/IEC by the Investigator and reviewed and approved by the IRB/IEC before the study is initiated.
- Any amendments to the protocol will require IRB/IEC approval before implementation of changes made to the study design, except for changes necessary to eliminate an immediate hazard to study participants.
- The Investigator will be responsible for the following:
  - Providing written summaries of the status of the study to the IRB/IEC annually or more frequently in accordance with the requirements, policies, and procedures established by the IRB/IEC.
  - Notifying the IRB/IEC of SAEs or other significant safety findings as required by IRB/IEC procedures.
  - Providing oversight of the conduct of the study at the site and adherence to requirements of 21 Code of Federal Regulations (CFR), ICH guidelines, the IRB/IEC, European regulation 536/2014 for clinical studies (if applicable), and all other applicable local regulations.

10.1.2 Financial disclosure

Investigators and sub-Investigators will provide the Sponsor with sufficient, accurate financial information as requested to allow the Sponsor to submit complete and accurate financial certification or disclosure statements to the appropriate regulatory authorities. Investigators are responsible for providing information on financial interests during the course of the study and for 1 year after completion of the study.

10.1.3 Informed consent process

- The Investigator or his/her representative will explain the nature of the study to the participant or his/her legally authorized representative and answer all questions regarding the study.
- Participants must be informed that their participation is voluntary. Participants will be required to sign a statement of informed consent that meets the requirements of 21 CFR 50, local regulations, ICH guidelines, Health Insurance Portability and Accountability Act requirements, where applicable, and the IRB/IEC or study center.
- The medical record must include a statement that written informed consent was obtained before the participant was enrolled in the study and the date the written consent was obtained. The authorized person obtaining the informed consent must also sign the ICF.
- Participants must be re-consented to the most current version of the ICF(s) during their participation in the study.
- A copy of the ICF(s) must be provided to the participant or the participant's legally authorized representative.

Participants who are rescreened are required to sign a new ICF.

The ICF will contain a separate section that addresses the use of remaining mandatory samples as well as additional serum/plasma samples for optional exploratory research. The Investigator or authorized designee will explain to each participant the objectives of the exploratory research. Participants will be told that they are free to refuse to participate and may withdraw their consent at any time and for any reason during the storage period. An optional consent will be required to document a participant's agreement to allow any remaining specimens to be used for exploratory research. Participants who decline to participate in this optional research will not provide this optional consent.

The ICF will also contains a separate section concerning the other optional procedure (genetic testing [DNA/RNA sampling]). An optional consent will be required to document a participant's agreement to each of this procedure. Participants who decline to participate will not provide this optional consent. A specific consent will be provided for HIV testing if specific consent is locally required.

10.1.4 Data protection

All personal data collected related to participants, Investigators, or any person involved in the study, which may be included in the Sponsor's databases, shall be treated in compliance with all applicable laws and regulations including the Global Data Protection Regulation.

Data collected must be adequate, relevant and not excessive, in relation to the purposes for which they are collected. Each category of data must be properly justified and in line with the study objective.

Participant race and ethnicity will be collected in this study because these data are required by regulatory agencies (eg, for reporting effectiveness data by racial subgroup in marketing authorization applications for FDA, or to report on the Japanese population for the Pharmaceuticals and Medical Devices Agency in Japan). In addition, race has been identified to influence PN incidence (15).

- Participants will be assigned a unique identifier by the Sponsor. Any participant records or datasets that are transferred to the Sponsor will contain the identifier only; participant names or any information which would make the participant identifiable will not be transferred.
- The participant must be informed that his/her personal study-related data will be used by the Sponsor in accordance with local data protection law. The level of disclosure must also be explained to the participant.
- The participant must be informed that his/her medical records may be examined by Clinical Quality Assurance auditors or other authorized personnel appointed by the Sponsor, by appropriate IRB/IEC members, and by inspectors from regulatory authorities.
- When archiving or processing personal data pertaining to the Investigator and/or to the participants, the Sponsor shall take all appropriate measures to safeguard and prevent access to this data by any unauthorized third party.

10.1.5 Committees structure

There will be no study committees.

10.1.6 Dissemination of clinical study data

Sanofi shares information about clinical trials and results on publically accessible websites, based on company commitments, international and local legal and regulatory requirements, and other clinical trial disclosure commitments established by pharmaceutical industry associations. These websites include clinicaltrials.gov, EU clinicaltrialregister (eu.ctr), and sanofi.com, as well as some national registries.

In addition, results from clinical trials in patients are required to be submitted to peer-reviewed journals following internal company review for accuracy, fair balance and intellectual property. For those journals that request sharing of the analyzable data sets that are reported in the publication, interested researchers are directed to submit their request to clinicalstudydatarequest.com.

Individual participant data and supporting clinical documents are available for request at clinicalstudydatarequest.com. While making information available we continue to protect the privacy of participants in our clinical trials. Details on data sharing criteria and process for requesting access can be found at this web address: clinicalstudydatarequest.com.

10.1.7 Data quality assurance

- All participant data relating to the study will be recorded on printed or electronic CRF unless transmitted to the Sponsor or designee electronically (eg, laboratory data). The Investigator is responsible for verifying that data entries are accurate and correct by physically or electronically signing the CRF.
- The Investigator must maintain accurate documentation (source data) that supports the information entered in the CRF.
- The Investigator must permit study-related monitoring, audits, IRB/IEC review, and regulatory agency inspections and provide direct access to source data documents.
- Monitoring details describing strategy (eg, risk-based initiatives in operations and quality such as Risk Management and Mitigation Strategies and Analytical Risk-Based Monitoring), methods, responsibilities and requirements, including handling of noncompliance issues and monitoring techniques (central, remote, or on-site monitoring) are provided in separate study documents.
- The Sponsor or designee is responsible for the data management of this study including quality checking of the data.
- The Sponsor assumes accountability for actions delegated to other individuals (eg, Contract Research Organizations).
- Study monitors will perform ongoing source data verification to confirm that data entered into the CRF by authorized site personnel are accurate, complete, and verifiable from source documents; that the safety and rights of participants are being protected; and that the study is being conducted in accordance with the currently approved protocol and any other study agreements, ICH GCP, and all applicable regulatory requirements.
- Records and documents, including signed ICFs, pertaining to the conduct of this study must be retained by the Investigator for 25 years after the signature of the final study report unless local regulations or institutional policies require a longer retention period. No records may be destroyed during the retention period without the written approval of the Sponsor. No records may be transferred to another location or party without written notification to the Sponsor.

10.1.8 Source documents

- Source documents provide evidence for the existence of the participant and substantiate the integrity of the data collected. Source documents are filed at the Investigator's site.
- Data reported on the CRF or entered in the eCRF that are transcribed from source documents must be consistent with the source documents or the discrepancies must be explained. The Investigator may need to request previous medical records or transfer records, depending on the study. Also, current medical records must be available.

- Source data is all information in original records and certified copies of original records of clinical findings, observations, or other activities in a clinical trial necessary for the reconstruction and evaluation of the trial. Source data are contained in source documents. Source documents are original documents, data and records such as hospital records, clinic and office charts, laboratory notes, memoranda, pharmacy dispensing records, recorded data from automated instruments, etc.

10.1.9 Study and site closure

The Sponsor or designee reserves the right to close the study site or terminate the study at any time for any reason at the sole discretion of the Sponsor. Study sites will be closed upon study completion. A study site is considered closed when all required documents and study supplies have been collected and a study-site closure visit has been performed.

The Investigator may initiate study-site closure at any time, provided there is reasonable cause and sufficient notice is given in advance of the intended termination.

Reasons for study termination by the Sponsor, as well as reasons for the early closure of a study site by the Sponsor or Investigator may include but are not limited to:

- For study termination:
  - Information on the product leads to doubt as to the benefit/risk ratio.
  - Discontinuation of further study intervention development.
- For site termination:
  - Failure of the Investigator to comply with the protocol, the requirements of the IRB/IEC or local health authorities, the Sponsor’s procedures, or GCP guidelines.
  - Inadequate or no recruitment (evaluated after a reasonable amount of time) of participants by the Investigator.
  - Total number of participants included earlier than expected.

10.1.10 Publication policy

- The results of this study may be published or presented at scientific meetings. If this is foreseen, the Investigator agrees to submit all manuscripts or abstracts to the Sponsor before submission. This allows the Sponsor to protect proprietary information and to provide comments.
- The Sponsor will comply with the requirements for publication of study results. In accordance with standard editorial and ethical practice, the Sponsor will generally support publication of multicenter studies only in their entirety and not as individual site data. In this case, a coordinating Investigator will be designated by mutual agreement.
- Authorship will be determined by mutual agreement and in line with International Committee of Medical Journal Editors authorship requirements.

10.2 APPENDIX 2: CLINICAL LABORATORY TESTS

The tests detailed in Table 9 will be performed by the central laboratory.

- Local laboratory results are only required in the event that the central laboratory results are not available in time for either study intervention administration and/or response evaluation. If a local sample is required, it is important that the sample for central analysis is obtained at the same time. Additionally, if the local laboratory results are used to make either a study intervention decision or response evaluation, the results must be entered into the CRF.
- Protocol-specific requirements for inclusion or exclusion of participants are detailed in Section 5 of the protocol.
- Additional tests may be performed at any time during the study as determined necessary by the Investigator or required by local regulations.
- Additional serum or urine pregnancy tests may be performed, as determined necessary by the investigator or required by local regulation, to establish the absence of pregnancy at any time during the subject's participation in the study.

Table 9 - Protocol-required laboratory assessments

Laboratory assessments	Parameters
Hematology	
	Platelet count
	RBC count
	Hemoglobin
	Hematocrit
	WBC count with differential:
	Neutrophils
	Lymphocytes
	Monocytes
	Eosinophils
	Basophils
	CD4 T cell count (for participants with HIV history only)
Clinical chemistry[a]	
	BUN
	Creatinine
	Glucose
	Lactate dehydrogenase
	Uric acid
	Total cholesterol
	Potassium

Laboratory assessments	Parameters
	Sodium<br>Chloride<br>Bicarbonate<br>AST/SGOT<br>ALT/SGPT<br>Alkaline phosphatase<br>Creatine phosphokinase<br>Total bilirubin<br>Total protein<br>Albumin<br>Hemoglobin A1c (only for diabetic patients without hemoglobin A1c laboratory results within 3 months before screening visit)
Urinalysis	• Specific gravity<br>• pH, glucose, protein, blood, ketones, bilirubin, urobilinogen, nitrite, leukocyte esterase by dipstick<br>• Creatinine and leukotriene (central laboratory)<br>• Microscopic examination (if blood or protein is abnormal)<br>• Tetranor PGDM (central laboratory)
Other screening tests	• Highly sensitive serum (at screening) or urine (at other timepoints) hCG pregnancy test (as needed for WOCBP)[b]<br>• Serology[c]: HBs Ag, HBs Ab, HBc Ab, HCV Ab, HIV screen (Anti-HIV-1 and HIV-2 antibodies); HIV viral load test (for participants with HIV history only)<br>• Tuberculosis test<br>• All study-required laboratory assessments will be performed by a central laboratory, with the exception of TB test, urine pregnancy test, and routine urinalysis.

NOTES:

a Details of liver chemistry stopping criteria and required actions and follow-up assessments after liver stopping or monitoring event are given in Section 7.1 and Section 10.6. All events which may indicate severe liver injury (meeting Hy's Law laboratory criteria; ALT or AST >3 x ULN and total bilirubin >2 x ULN) must be reported as an SAE.

b After screening, local urine testing will be standard for the protocol unless serum testing is required by local regulation or IRB/IEC.

c In case of results showing HBs Ag (negative) and HBc Ab (positive), an HBV DNA testing will be performed and should be confirmed negative prior to randomization. In case of results showing HCV Ab (positive), HCV RNA testing will be performed and should be confirmed negative prior to randomization.

ALT: alanine aminotransferase; AST: aspartate aminotransferase; BUN: blood urea nitrogen; DNA: deoxyribonucleic acidHBc Ab: hepatitis B core antibody; HBs Ab: hepatitis B surface antibody; HBs Ag: hepatitis B surface antigen; HBV: hepatitis B virus; hCG: human chorionic gonadotropin; HCV: hepatitis C virus; HCV Ab: hepatitis C virus antibody; HIV: human immunodeficiency virus; IEC: Independent Ethics Committee; IRB: Institutional Review Board; RBC: red blood cells; RNA: ribonucleic acid; SAE: serious adverse event; SGOT: serum glutamic-oxaloacetic transaminase; SGPT: serum glutamic-pyruvic transaminase; TB: tuberculosis; ULN: upper limit of normal; WBC: white blood cells; WOCBP: woman of childbearing potential

Investigators must document their review of each laboratory safety report.

Laboratory/analyte results that could unblind the study will not be reported to investigative sites or other blinded personnel until the study has been unblinded.

10.3 APPENDIX 3: ADVERSE EVENTS: DEFINITIONS AND PROCEDURES FOR RECORDING, EVALUATING, FOLLOW-UP, AND REPORTING

DEFINITION OF AE

AE definition

- An AE is any untoward medical occurrence in a patient or clinical study participant, temporally associated with the use of study intervention, whether or not considered related to the study intervention.

  NOTE: An AE can therefore be any unfavorable and unintended sign (including an abnormal laboratory finding), symptom, or disease (new or exacerbated) temporally associated with the use of study intervention.

Events meeting the AE definition

- Any abnormal laboratory test results (hematology, clinical chemistry, or urinalysis) or other safety assessments (eg, ECG, radiological scans, vital signs measurements), including those that worsen from baseline, considered clinically significant in the medical and scientific judgment of the Investigator (ie, not related to progression of underlying disease).
- Exacerbation of a chronic or intermittent pre-existing condition including either an increase in frequency and/or intensity of the condition.
- New conditions detected or diagnosed after study intervention administration even though it may have been present before the start of the study.
- Signs, symptoms, or the clinical sequelae of a suspected drug-drug interaction.
- Signs, symptoms, or the clinical sequelae of a suspected overdose of either study intervention or a concomitant medication.
- “Lack of efficacy” or “failure of expected pharmacological action” per se will not be reported as an AE or SAE. Such instances will be captured in the efficacy assessments. However, the signs, symptoms, and/or clinical sequelae resulting from lack of efficacy will be reported as AE or SAE if they fulfil the definition of an AE or SAE.

Events NOT meeting the AE definition

- Any clinically significant abnormal laboratory findings or other abnormal safety assessments which are associated with the underlying disease, unless judged by the Investigator to be more severe than expected for the participant’s condition.
- The disease/disorder being studied or expected progression, signs, or symptoms of the disease/disorder being studied, unless more severe than expected for the participant’s condition.
- Medical or surgical procedure (eg, endoscopy, appendectomy): the condition that leads to the procedure is the AE.

- Situations in which an untoward medical occurrence did not occur (social and/or convenience admission to a hospital).
- Anticipated day-to-day fluctuations of pre-existing disease(s) or condition(s) present or detected at the start of the study that do not worsen.

DEFINITION OF SAE

If an event is not an AE per definition above, then it cannot be an SAE even if serious conditions are met (eg, hospitalization for signs/symptoms of the disease under study, death due to progression of disease).

A SAE is defined as any untoward medical occurrence that, at any dose:

a) **Results in death**

b) **Is life-threatening**

The term "life-threatening" in the definition of "serious" refers to an event in which the participant was at risk of death at the time of the event. It does not refer to an event, which hypothetically might have caused death, if it were more severe.

c) **Requires inpatient hospitalization or prolongation of existing hospitalization**

In general, hospitalization signifies that the participant has been detained (usually involving at least an overnight stay) at the hospital or emergency ward for observation and/or treatment that would not have been appropriate in the physician's office or outpatient setting. Complications that occur during hospitalization are AEs. If a complication prolongs hospitalization or fulfills any other serious criteria, the event is serious. When in doubt as to whether "hospitalization" occurred or was necessary, the AE should be considered serious.

Hospitalization for elective treatment of a pre-existing condition that did not worsen from baseline is not considered an AE.

d) **Results in persistent disability/incapacity**

- The term disability means a substantial disruption of a person's ability to conduct normal life functions.
- This definition is not intended to include experiences of relatively minor medical significance such as uncomplicated headache, nausea, vomiting, diarrhea, influenza, and accidental trauma (eg, sprained ankle) which may interfere with or prevent everyday life functions but do not constitute a substantial disruption.

e) **Is a congenital anomaly/birth defect**

f) **Other situations:**

- Medical or scientific judgment should be exercised in deciding whether SAE reporting is appropriate in other situations such as important medical events that may not be immediately life-threatening or result in death or hospitalization but may jeopardize the participant or may require medical or surgical intervention to prevent one of the other outcomes listed in the above definition. These events should usually be considered serious.

- Examples of such events include invasive or malignant cancers, intensive treatment in an emergency room or at home for allergic bronchospasm, blood dyscrasias or convulsions that do not result in hospitalization, or development of drug dependency or drug abuse.

RECORDING AND FOLLOW-UP OF AE AND/OR SAE

AE and SAE recording

- When an AE/SAE occurs, it is the responsibility of the Investigator to review all documentation (eg, hospital progress notes, laboratory reports, and diagnostics reports) related to the event.
- The Investigator will then record all relevant AE/SAE information in the CRF.
- It is **not** acceptable for the Investigator to send photocopies of the participant's medical records to the Sponsor's representative in lieu of completion of the AE/SAE CRF page.
- There may be instances when copies of medical records for certain cases are requested by the Sponsor's representative. In such case, care should be taken to ensure that the patient's identity is protected and the patient's identifiers in the study are properly mentioned on any copy of a source document provided to the Sponsor.
- The Investigator will attempt to establish a diagnosis of the event based on signs, symptoms, and/or other clinical information. Whenever possible, the diagnosis (not the individual signs/symptoms) will be documented as the AE/SAE.

Assessment of intensity

The Investigator will make an assessment of intensity for each AE and SAE reported during the study and assign it to 1 of the following categories:

- Mild: An event that is easily tolerated by the participant, causing minimal discomfort and not interfering with everyday activities.
- Moderate: An event that causes sufficient discomfort and interferes with normal everyday activities.
- Severe: An event that prevents normal everyday activities. An AE that is assessed as severe should not be confused with a SAE. Severe is a category utilized for rating the intensity of an event; and both AEs and SAEs can be assessed as severe.

  An event is defined as "serious" when it meets at least 1 of the predefined outcomes as described in the definition of an SAE, NOT when it is rated as severe.

Assessment of causality

- The Investigator is obligated to assess the relationship between study intervention and each occurrence of each AE/SAE.
- A "reasonable possibility" of a relationship conveys that there are facts, evidence, and/or arguments to suggest a causal relationship, rather than that a relationship cannot be ruled out.

- The Investigator will use clinical judgment to determine whether or not the relationship is causal.
- Alternative causes, such as underlying disease(s), concomitant therapy, and other risk factors, as well as the temporal relationship of the event to study intervention administration will be considered and investigated.
- The Investigator will also consult the IB and/or Product Information, for marketed products, in his/her assessment.
- For each AE/SAE, the Investigator **must** document in the medical notes that he/she has reviewed the AE/SAE and has provided an assessment of causality.
- There may be situations in which an SAE has occurred and the Investigator has minimal information to include in the initial report to the Sponsor's representative. However, **it is very important that the Investigator always make an assessment of causality for every event before the initial transmission of the SAE data to the Sponsor's representative.**
- The Investigator may change his/her opinion of causality in light of follow-up information and send a SAE follow-up report with the updated causality assessment.
- The causality assessment is one of the criteria used when determining regulatory reporting requirements.

Follow-up of AEs and SAEs

- The Investigator is obligated to perform or arrange for the conduct of supplemental measurements and/or evaluations as medically indicated or as requested by the Sponsor's representative to elucidate the nature and/or causality of the AE or SAE as fully as possible. This may include additional laboratory tests or investigations, histopathological examinations, or consultation with other health care professionals.
- If a participant dies during participation in the study or during a recognized follow-up period, the Investigator will provide the Sponsor's representative with a copy of any post-mortem findings including histopathology.
- New or updated information will be recorded in the originally completed CRF.
- The Investigator will submit any updated SAE data to the Sponsor within 24 hours of receipt of the information.

REPORTING OF SAES

SAE reporting to the Sponsor via an electronic data collection tool

- The primary mechanism for reporting an SAE to the Sponsor's representative will be the electronic data collection tool.
- If the electronic system is unavailable, then the site will use the paper SAE data collection tool (see next section) in order to report the event within 24 hours.
- The site will enter the SAE data into the electronic system as soon as it becomes available.

- After the study is completed at a given site, the electronic data collection tool will be taken off-line to prevent the entry of new data or changes to existing data.
- If a site receives a report of a new SAE from a study participant or receives updated data on a previously reported SAE after the electronic data collection tool has been taken off-line, then the site can report this information on a paper SAE form (see next section) or to the Sponsor's representative by telephone.
- Contacts for SAE reporting can be found in the protocol.

SAE reporting to the Sponsor's representative via paper CRF

- Facsimile transmission of the SAE paper CRF is the preferred method to transmit this information to the Sponsor's representative.
- In rare circumstances and in the absence of facsimile equipment, notification by telephone is acceptable with a copy of the SAE data collection tool sent by overnight mail or courier service.
- Initial notification via telephone does not replace the need for the Investigator to complete and sign the SAE CRF pages within the designated reporting time frames.
- Contacts for SAE reporting can be found in the protocol.

10.4 APPENDIX 4: CONTRACEPTIVE GUIDANCE AND COLLECTION OF PREGNANCY INFORMATION

DEFINITIONS:

Woman of childbearing potential (WOCBP)

A woman is considered fertile following menarche and until becoming post-menopausal unless permanently sterile (see below).

If fertility is unclear (eg, amenorrhea in adolescents or athletes) and a menstrual cycle cannot be confirmed before first dose of study intervention, additional evaluation should be considered.

Women in the following categories are not considered WOCBP

1. Premenarchal
2. Premenopausal female with 1 of the following:
   - Documented hysterectomy
   - Documented bilateral salpingectomy
   - Documented bilateral oophorectomy

   For individuals with permanent infertility due to an alternate medical cause other than the above, (eg, mullerian agenesis, androgen insensitivity), Investigator discretion should be applied to determining study entry.

Note: Documentation can come from the site personnel's: review of the participant's medical records, medical examination, or medical history interview.

3. Postmenopausal female
   - A postmenopausal state is defined as no menses for 12 consecutive months without an alternative medical cause.
   - Females on hormone replacement therapy (HRT) and whose menopausal status is in doubt will be required to use one of the non-estrogen hormonal highly effective contraception methods if they wish to continue their HRT during the study.

CONTRACEPTION GUIDANCE

Female participants of childbearing potential are eligible to participate if they agree to use a highly effective method of contraception consistently and correctly as described below.

**Contraceptives[a] allowed during the study include:**
**Highly effective methods[b] that have low user dependency**<br>*Failure rate of <1% per year when used consistently and correctly.*
• Implantable progestogen-only hormone contraception associated with inhibition of ovulation[b]<br>- Intrauterine device (IUD)<br>- Intrauterine hormone-releasing system (IUS)[b]<br>• Bilateral tubal occlusion
Vasectomized partner<br>*(Vasectomized partner is a highly effective contraceptive method provided that the partner is the sole sexual partner of the WOCBP and the absence of sperm has been confirmed. If not, an additional highly effective method of contraception should be used. Spermatogenesis cycle is approximately 90 days.)*
**Highly effective methods[b] that are user dependent**<br>*Failure rate of <1% per year when used consistently and correctly.*
• Combined (estrogen- and progestogen-containing) hormonal contraception associated with inhibition of ovulation[c]<br>- oral<br>- intravaginal<br>- transdermal<br>- injectable<br>• Progestogen-only hormone contraception associated with inhibition of ovulation[c]<br>- oral<br>- injectable
**Sexual abstinence**<br>*Sexual abstinence is considered a highly effective method only if defined as refraining from heterosexual intercourse during the entire period of risk associated with the study intervention. The reliability of sexual abstinence needs to be evaluated in relation to the duration of the study and the preferred and usual lifestyle of the participant.)*

a Contraceptive use by men or women should be consistent with local regulations regarding the use of contraceptive methods for those participating in clinical studies.

b Failure rate of <1% per year when used consistently and correctly. Typical use failure rates differ from those when used consistently and correctly.

c If locally required, in accordance with Clinical Trial Facilitation Group (CTFG) guidelines, acceptable contraceptive methods are limited to those which inhibit ovulation as the primary mode of action.

WOCBP: woman of childbearing potential

COLLECTION OF PREGNANCY INFORMATION:

Male participants with partners who become pregnant

- The Investigator will attempt to collect pregnancy information on any male participant's female partner who becomes pregnant while the male participant is in this study. This applies only to male participants who receive dupilumab.
- After obtaining the necessary signed informed consent from the pregnant female partner directly, the Investigator will record pregnancy information on the appropriate form and submit it to the Sponsor within 24 hours of learning of the partner's pregnancy. The female partner will also be followed to determine the outcome of the pregnancy. Information on the status of the mother and child will be forwarded to the Sponsor. Generally, the follow-up will be no longer than 6 to 8 weeks following the estimated delivery date. Any termination of the pregnancy will be reported regardless of fetal status (presence or absence of anomalies) or indication for the procedure.

Female participants who become pregnant

- The Investigator will collect pregnancy information on any female participant who becomes pregnant while participating in this study. Information will be recorded on the appropriate form and submitted to the Sponsor within 24 hours of learning of a participant's pregnancy. The participant will be followed to determine the outcome of the pregnancy. The Investigator will collect follow-up information on the participant and the neonate and the information will be forwarded to the Sponsor. Generally, follow-up will not be required for longer than 6 to 8 weeks beyond the estimated delivery date. Any termination of pregnancy will be reported, regardless of fetal status (presence or absence of anomalies) or indication for the procedure.
- Any pregnancy complication or elective termination of a pregnancy will be reported as an AE or SAE. A spontaneous abortion is always considered to be an SAE and will be reported as such. Any post-study pregnancy related SAE considered reasonably related to the study intervention by the Investigator will be reported to the Sponsor as described in Section 8.3.4 of the protocol. While the Investigator is not obligated to actively seek this information in former study participants, he or she may learn of an SAE through spontaneous reporting.
- Any female participant who becomes pregnant while participating in the study will discontinue study intervention.

10.5 APPENDIX 5: GENETICS

Use/Analysis of DNA and RNA

- Genetic variation may impact a participant's response to study intervention, susceptibility to, and severity and progression of disease. Variable response to study intervention may be due to genetic determinants that impact drug absorption, distribution, metabolism, and excretion; mechanism of action of the drug; safety, disease etiology; and/or molecular

subtype of the disease being treated. Therefore, where local regulations and IRB/IEC allow, a blood sample will be collected for DNA analysis from consenting participants.

- DNA and RNA samples will be used for research related to dupilumab or PN and related diseases. They may also be used to develop tests/assays including diagnostic tests related to dupilumab or related drugs and atopic/allergic diseases. Genetic research may consist of the analysis of one or more candidate genes or the analysis of genetic markers throughout the genome including whole-exome sequencing, whole-genome sequencing, and DNA copy number variation. Transcriptome sequencing (or other methods for quantitating RNA expression) may also be performed.
- The samples may be analyzed as part of a multi-study assessment of genetic factors involved in the response to dupilumab or study interventions of this class to understand study disease or related conditions.
- The results of genetic analyses will not be reported in the CSR.
- The Sponsor will store the DNA samples in a secure storage space with adequate measures to protect confidentiality.
- The samples will be retained no longer than 15 years or other period as per local requirements.

10.6 APPENDIX 6: LIVER AND OTHER SAFETY: SUGGESTED ACTIONS AND FOLLOW-UP ASSESSMENTS

INCREASE IN ALT

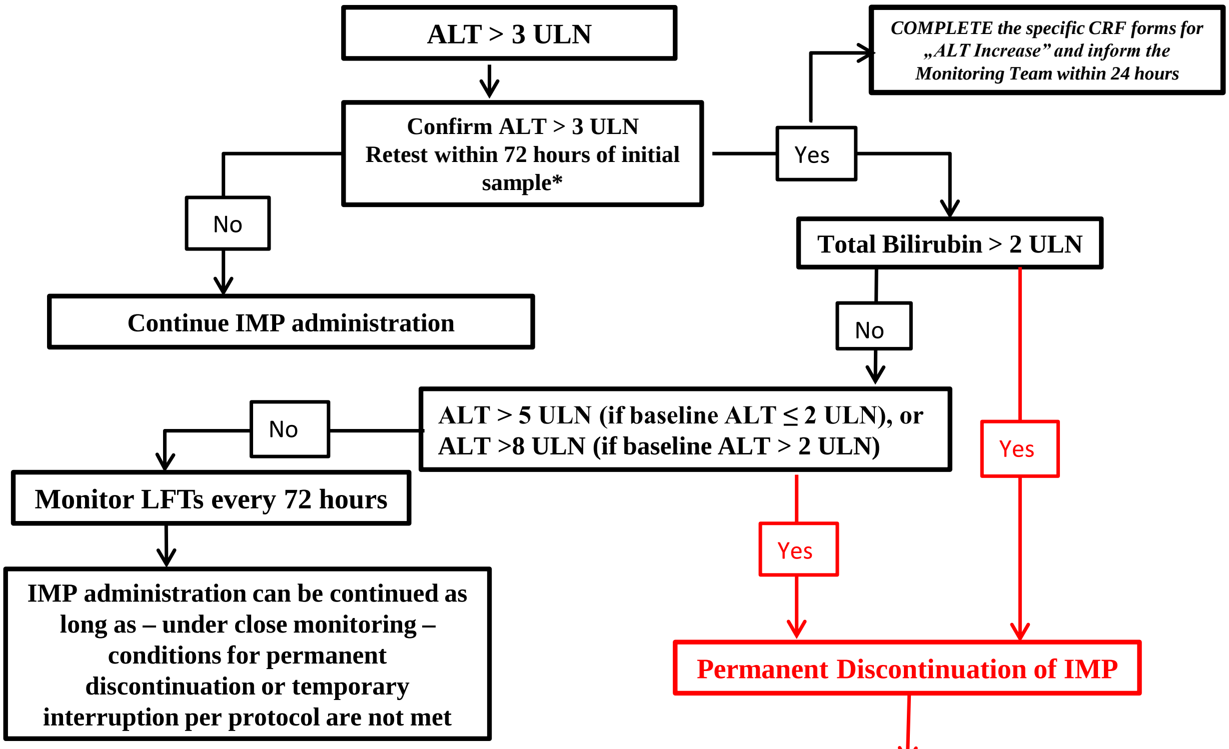

In ANY CASE, FOLLOW the instructions listed in the box below:

1. **INFORM** the Site Monitor who will forward the information to the Study Manager
2. **INVESTIGATE** specifically for malaise with or without loss of consciousness, dizziness, and/or hypotension and/or episode of arrhythmia in the previous 72 hours; rule out muscular injury
3. **PERFORM** the following tests:
   - LFTs: AST, ALT, alkaline phosphatase, total and conjugated bilirubin and prothrombin time / INR
   - CPK, serum creatinine, complete blood count
   - Anti-HAV IgM, anti-HBc IgM (HBV-DNA if clinically indicated), anti-HCV and HCV RNA, anti-CMV IgM and anti-HEV IgM antibodies
   - Depending on the clinical context, check for recent infection with EBV, herpes viruses, and toxoplasma
   - Hepatobiliary ultrasonography (or other imaging investigations if needed)
4. **CONSIDER** Auto-antibodies: antinuclear, anti-DNA, anti-smooth muscle, anti-LKM
5. **CONSIDER** consulting with hepatologist
6. **CONSIDER** patient hospitalisation if INR>2 (or PT<50%) and/or central nervous system disburbances suggesting hepatic encephalopathy
7. **MONITOR LFTs after discontinuation of IMP:**
   - As *closely as possible* (or **every 48 hours)** until stabilization, then every 2 weeks until return to normal/baseline or clinical resolution.
8. **FREEZE** serum sample (5ml x 2)
9. **In case of SUSPICION of GILBERT Syndrome,** a DNA diagnostic test should be done

*If unable to retest in 72 hours, use original lab results to decide on further reporting/monitoring/discontinuation.

Note:

“Baseline” refers to ALT sampled at baseline visit; or if baseline value unavailable, to the latest ALT sampled before the baseline visit. The algorithm does not apply to the instances of increase in ALT during screening.

See Section 10.3 for guidance on safety reporting.

Normalization is defined as ≤ULN or baseline value, if baseline value is >ULN.

INCREASE IN SERUM CREATININE in patients with normal baseline (creatininemia between 45 µmol/L and 84 µmol/L)

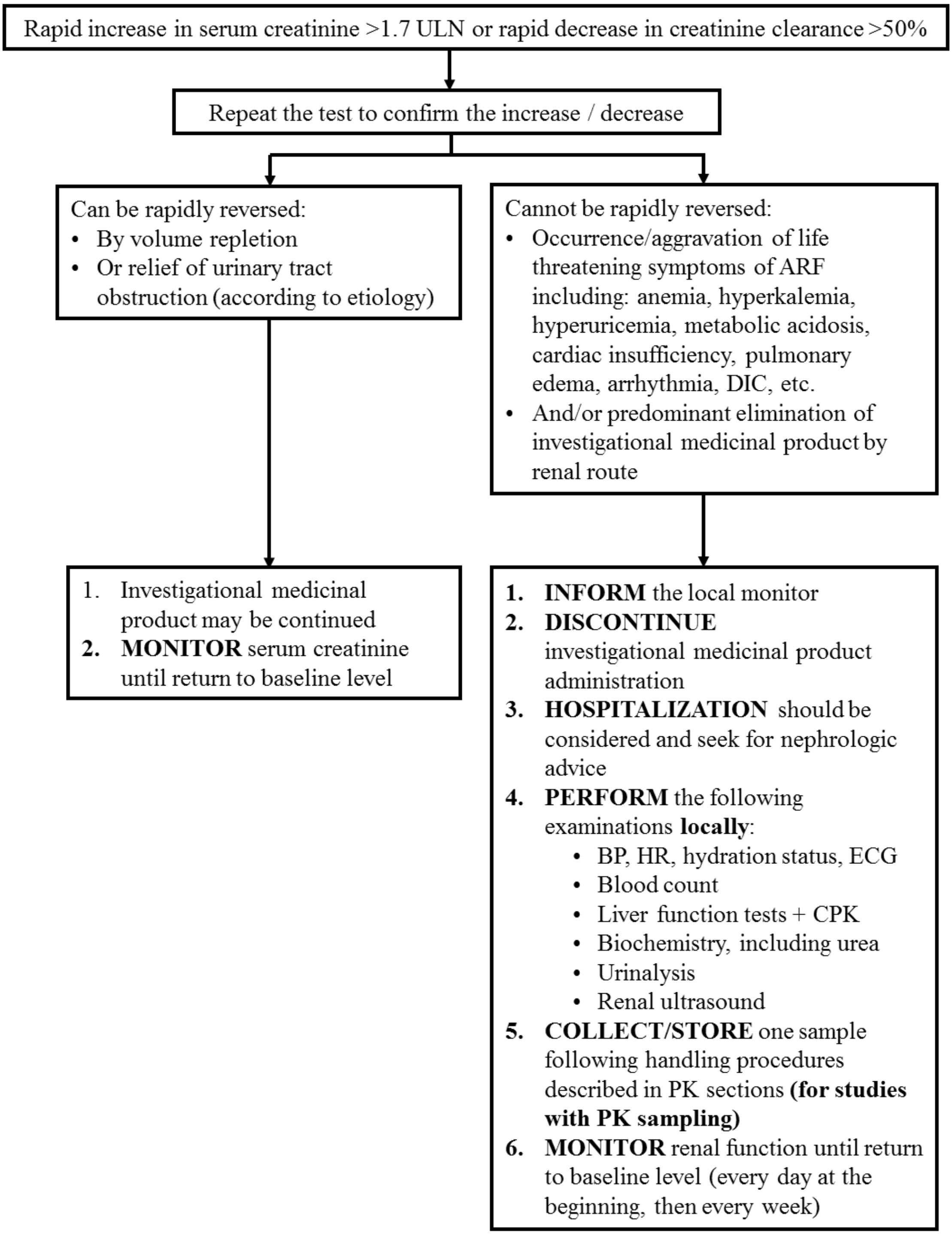

Increase in serum creatinine is to be recorded as an AE only if at least 1 of the criteria listed in the general guidelines for reporting AEs in Section 10.3 is met.

INCREASE IN CPK OF NON-CARDIAC ORIGIN AND NOT RELATED TO INTENSIVE PHYSICAL ACTIVITY

If Increase in CPK > 10 ULN

↓

Repeat immediately locally the count.
If confirmed, inform the local monitor and

↓

INVESTIGATE for the origin:

- **INTERVIEW** the patient about a recent intensive muscular effort, trauma, convulsions, electrical injury, or stress to the skeletal muscle, multiple intramuscular injections, recent surgery, concomitant medications, consumption of alcohol, morphine, cocaine…
- **PERFORM locally**:
  - ECG, CK-MB , Troponin if not previously done
  - CK-MM
  - Creatinine
  - Iono (K+)
  - Transaminases + Total and conjugated bilirubin
  - And any other parameters considered as relevant per medical judgement (e.g. Ca^{2}+, Myoglobin)
- **COLLECT/STORE** one sample following handling procedures described in PK sections **(for studies with PK sampling)**
- **SEARCH** for the cause

↓

1. Hospitalization should be considered depending on clinical context and profile of the patient
2. Consider to discontinue investigational medicinal product administration
3. Monitor biological parameters as appropriate within the next days/weeks/months until return to baseline

Increase in CPK is to be recorded as an AE only if at least 1 of the criteria in the general guidelines for reporting AEs in Section 10.3 is met.

10.7 APPENDIX 7: CLINICIANS-REPORTED OUTCOMES AND PATIENT-REPORTED OUTCOMES

10.7.1 Worst itch numeric rating scale (WI-NRS)

[redacted]

GB ZD . 01/2019

UKM: AöR, Prof. Dr. Martin Schulze Schwienhorst (Aufsichtsratsvorsitzender),
Univ.-Prof. Dr. med. Dr. phil. Robert Nitsch (Vorstandsvorsitzender, Ärztlicher Direktor),
Dr. rer. pol. Christoph Hoppenheit (stellv. Vorstandsvorsitzender, Kaufmännischer Direktor),
Univ.-Prof. Dr. med. Mathias Herrmann (Dekan), Thomas van den Hooven (Pflegedirektor),
Univ.-Prof. Dr. med. Claudia Rössig (stellv. Ärztliche Direktorin)

KTQ-Zertifikat

10.7.2 Investigator’s global assessment of prurigo nodularis (IGA PN)

[redacted]

Copyright @ Center for Chronic Pruritus, University Hospital Münster Under license. For permission to use refer to: KCPadministration@ukmuenster.de

10.7.3 Prurigo activity score (PAS)

[redacted]

[redacted]

Copyright @ Center for Chronic Pruritus, University Hospital Münster Under license. For permission to use refer to: KCPadministration@ukmuenster.de

10.7.4 Dermatology life quality index (DLQI)

[redacted]

©AY Finlay, GK Khan, April 1992, This must not be copied without the permission of the authors.

10.7.5 Hospital anxiety and depression scale (HADS)

[redacted]

HADS - United States/English - Version of 30 Jan 17 - Mapi.
ID058041 / HADS_AU5.0_eng-US.doc

10.7.6 EQ-5D-5L

[redacted]

Disclaimer: This is a preview of the EQ-5D instrument. It demonstrates the text, questions and response options included in this version. This preview does not represent the final product and should not be used as an official EQ-5D instrument.

10.7.7 Pain numeric rating scale

Worst Skin Pain Numerical Rating Scale (Generic)

How would you rate your skin pain at its **worst** in the past 24 hours?

0	1	2	3	4	5	6	7	8	6	10
No pain										Worst pain possible

10.7.8 Sleep numeric rating scale

Sleep Quality Numerical Rating Scale (Generic)

Instructions:

Please complete the following questions upon awakening for the day.

Select the number that best describes the quality of your sleep last night.

0	1	2	3	4	5	6	7	8	9	10
Worst possible sleep										Best possible sleep

10.7.9 Patient Global Impression of Change of disease (PGIC)

Patient Global Impression of Change (PGIC) of PN Disease

Please choose the response below that best describes the overall change in your prurigo nodularis since you started taking the study medication.

☐ Very much better

☐ Moderately better

☐ A little better

☐ No change

☐ A little worse

☐ Moderately worse

☐ Very much worse

10.7.10 Patient Global Impression of Severity (PGIS)

Patient Global Impression of Severity (PGIS) of PN Disease

Please choose the response below that best describes the severity of your prurigo nodularis over the past week.

☐ None

☐ Mild

☐ Moderate

☐ Severe

10.7.11 Missed school/work days

Patients with Prurigo Nodularis *(PN)*

Missed School/Work days
BASELINE Visit

Site Number: __________ Patient Study Number: ___________________________

Visit Date (dd/mmm/yyyy): ______/______/______Time of Completion: ___:_____am/pm

Employment status

1. Have you been working?

   □ Yes – I am working full time (≥4 days/week)
   □ Yes – I am working part time (<4 days/week)
   □ No – I am not working (unemployed, retired)

For those working (full time or part time):

2. How many days have you missed from work because of your Prurigo Nodularis in the last 4 weeks prior to the visit today?

   Please do not include time you missed from work for taking part in this research.

   Specify: __ __ __ Days

Student status

1. Have you been in school?

   □ Yes – I am in school full time (≥4 days/week)
   □ Yes – I am in school part time (<4 days/week)
   □ No – I am not in school

For those in school (full time or part time):

2. How many days have you missed from school because of your Prurigo Nodularis in the last 4 weeks prior to the visit today?

   Please do not include time you missed from school for taking part in this research.

   Specify: __ __ __ Days

NOTES:

Please make sure the instructions are read to the patient BEFORE they start the questionnaire.

Assessment periods (i.e., time interval from the date of last assessment through the current date) must be continuous. Leave no gaps. Cumulatively, these periods must cover the entire study duration.

Avoid duplicate reporting. If the patient missed school/work because of his/her PN on the day of

assessment, this day should be recorded only once.

Record only days on which patient's PN prevented him/her to go to school/work. Otherwise, do not record the days on which the patient missed school/work only to participate in study visits.

Print Name (First/Last) of Investigator Completing Assessment	Signature	Date

R668-XXX-Missed School/work Days Version 1.0 BASELINE; *date*

Page 1 of 1

Patients with *Prurigo Nodularis (PN)*

Missed School/work days
POST Baseline Visits

Site Number: ______________Patient Study Number: ______________

Visit Date (dd/mmm/yyyy): ____/_____/_____Time of Completion: ___:_____am/pm

Employment status

1. Have you been working since your last assessment?

 ◻ Yes – I am working full time (≥4 days/week)
 ◻ Yes – I am working part time (<4 days/week)
 ◻ No – I am not working (unemployed, retired)

For those working (full time or part time):

2. How many days have you missed from work because of your Prurigo Nodularis since the day of your last assessment, including today?

 Please do not include time you missed from work for taking part in this research.

 Specify: __ __ __ Days

Student status

1. Have you been in school since your last assessment?

 ◻ Yes – I am in school full time (≥4 days/week)
 ◻ Yes – I am in school part time (<4 days/week)
 ◻ No – I am not in school

For those in school (full time or part time):

2. How many days have you missed from school because of your Prurigo Nodularis since the day of your last assessment, including today?

 Please do not include time you missed from school for taking part in this research.

 Specify: __ __ __ Days

NOTES:

Please make sure the instructions are read to the patient BEFORE they start the questionnaire.

Assessment periods (i.e., time interval from the date of last assessment through the current date) must be continuous. Leave no gaps. Cumulatively, these periods must cover the entire study duration.

Avoid duplicate reporting. If the patient missed school/work because of his/her PN on the day of assessment, this day should be recorded only once.

Record only days on which patient's PN prevented him/her to go to school/work. Otherwise, do not record the days on which the patient missed school/work only to participate in study visits.

Print Name (First/Last) of Investigator Completing Assessment	Signature	Date

R668-XXX Missed School/work Days Version 2.0; date

Page 1 of 1

10.8 APPENDIX 8: DEFINITION OF ANAPHYLAXIS

"Anaphylaxis is a serious allergic reaction that is rapid in onset and may cause death (31)."

Clinical criteria for diagnosing anaphylaxis

Anaphylaxis is highly likely when any one of the following 3 criteria are fulfilled:

1. Acute onset of an illness (minutes to several hours) with involvement of the skin, mucosal tissue, or both (eg, generalized hives, pruritus or flushing, swollen lips-tongue-uvula)
   AND AT LEAST ONE OF THE FOLLOWING
   a. Respiratory compromise (eg, dyspnea, wheeze-bronchospasm, stridor, reduced PEF, hypoxemia)
   b. Reduced BP or associated symptoms of end-organ dysfunction (eg, hypotonia [collapse], syncope, incontinence)
2. Two or more of the following that occur rapidly after exposure *to a likely allergen for that patient* (minutes to several hours):
   a. Involvement of the skin-mucosal tissue (eg, generalized hives, itch-flush, swollen lips-tongue-uvula)
   b. Respiratory compromise (eg, dyspnea, wheeze-bronchospasm, stridor, reduced PEF, hypoxemia)
   c. Reduced BP or associated symptoms (eg, hypotonia [collapse], syncope, incontinence)
   d. Persistent gastrointestinal symptoms (eg, crampy abdominal pain, vomiting)
3. Reduced BP after exposure to *known allergen for that patient* (minutes to several hours):
   a. Infants and children: low systolic BP (age specific) or greater than 30% decrease in systolic BP*
   b. Adults: systolic BP of less than 90 mm Hg or greater than 30% decrease from that person's baseline

PEF, Peak expiratory flow; *BP*, blood pressure.
*Low systolic blood pressure for children is defined as less than 70 mm Hg from 1 month to 1 year, less than (70 mm Hg + [2 × age]) from 1 to 10 years, and less than 90 mm Hg from 11 to 17 years.

10.9 APPENDIX 9: LIST OF OPPORTUNISTIC INFECTIONS

- Aspergillosis
- Blastomyces dermatitidis (endemic in the south-eastern and south-central states US, along Mississippi and Ohio Rivers)
- Candidiasis - only systemic or extensive mucosal or cutaneous candidiasis
- Coccidioides immitis (endemic south-western US and Central and South America)
- Cryptococcus
- Cytomegalovirus
- Herpes Simplex (disseminated)
- Herpes Zoster (disseminated; ophthalmic; involvement of 2 or more dermatomes)
- Histoplasmosis (pulmonary or disseminated; most common tropical areas Tennessee-Ohio-Mississippi river basins)
- Listeriosis
- Mycobacterium avium
- Nontuberculosis mycobacteria
- Pneumocystis pneumonia

This list is indicative and not exhaustive.

10.10 APPENDIX 10: COUNTRY-SPECIFIC REQUIREMENTS

Not applicable.

10.11 APPENDIX 11: ABBREVIATIONS

ACE:	angiotensin converting enzyme
AD:	atopic dermatitis
ADA:	antidrug antibody
ADR:	adverse drug reaction
AE:	adverse event
AESI:	adverse event of special interest
ALT:	alanine aminotransferase
ANCOVA:	analysis of covariance
AST:	aspartate aminotransferase
BUN:	blood urea nitrogen
CFR:	Code of Federal Regulations
CIOMS:	Council for International Organizations of Medical Sciences
ClinRO:	clinician-reported outcome
CMH:	Cochran-Mantel-Haenszel
CONSORT:	Consolidated Standards of Reporting Trials
COVID-19:	coronavirus disease 2019
CRF:	case report form
CRSwNP:	chronic rhinosinusitis with nasal polyposis
CSR:	clinical study report
DBP:	diastolic blood pressure
DLQI:	dermatology life quality index
DMC:	data monitoring committee
DNA:	deoxyribonucleic acid
DTP:	direct-to-patient
EADV:	European Academy of Dermatology and Venereology
EASI:	eczema area and severity index
ECG:	electrocardiogram
eCRF:	electronic case report form
ELISA:	enzyme-linked immunosorbent assay
EMA:	European Medicines Agency
EoE:	eosinophilic esophagitis
EOS:	end of study
EOT:	end of treatment
EQ-5D:	Euroqol 5 dimensions
EQ-5D-5L:	Euroqol 5 dimensions 5 levels
EU:	European Union
FDA:	Food and Drug Administration
GCP:	Good Clinical Practice
HADS:	hospital anxiety and depression scale

HBc Ab:	hepatitis B core antibody
HBs Ab:	hepatitis B surface antibody
HBs Ag:	hepatitis B surface antigen
HBV:	hepatitis B virus
hCG:	human chorionic gonadotropin
HCV:	hepatitis C virus
HCV Ab:	hepatitis C virus antibody
HIV:	human immunodeficiency virus
HRQoL:	health-related quality of life
HRT:	hormone replacement therapy
IB:	investigator's brochure
ICF:	informed consent form
ICH:	International Council for Harmonisation
IEC:	Independent Ethics Committee
Ig:	immunoglobulin
IGA:	investigator's global assessment
IGA PN:	investigator's global assessment for prurigo nodularis
IGA PN-A:	IGA PN-Activity
IGA PN-S:	IGA PN-Stage
IL:	interleukin
IMP:	investigational medicinal product
IRB:	Institutional Review Board
IRT:	interactive response technology
ITT:	intent to treat
IVRS:	interactive voice response system
IWRS:	interactive web response system
LS:	least squares
NIMP:	noninvestigational medicinal product
NRS:	numeric rating scale
PAS:	prurigo activity score
PD:	pharmacodynamic
PGA:	physician global assessment
PGIC:	Patient Global Impression of Change
PGIS:	Patient Global Impression of Severity
PK:	pharmacokinetic
PN:	prurigo nodularis
PRO:	patient reported outcome
PT:	preferred term
Q2W:	every 2 weeks
QTcF:	QT interval corrected using Fridericia's formula
RBC:	red blood cells
RNA:	ribonucleic acid
SAE:	serious adverse event
SAP:	statistical analysis plan
SBP:	systolic blood pressure
SC:	subcutaneous

SCORAD:	scoring atopic dermatitis
SGOT:	serum glutamic-oxaloacetic transaminase
SGPT:	serum glutamic-pyruvic transaminase
SNRI:	serotonin and norepinephrin reuptake inhibitor
SoA:	schedule of activities
SOC:	system organ class
SSRI:	selective serotonin reuptake inhibitor
SUSAR:	suspected unexpected serious adverse reaction
TB:	tuberculosis
TCI:	topical calcineurin inhibitors
TCS:	topical corticosteroids
TEAE:	treatment-emergent adverse event
ULN:	upper limit of normal
US:	United States
UVB:	ultraviolet B
VAS:	visual analog scale
WBC:	white blood cells
WI-NRS:	worst-itch numeric rating scale
WOCBP:	woman of childbearing potential
WOCF:	worst observation carried forward

10.12 APPENDIX 12: PROTOCOL AMENDMENT HISTORY

The Protocol Amendment Summary of Changes Table for the current amendment is located directly before the Table of Contents (TOC).

11 REFERENCES

1. Zeidler C, Athanasios T, Pereira M, Stander H, Yosipovitch G, Stander S. Chronic prurigo of nodular type: A Review. Acta Derm Venereol 2018;98(2):173-9.

2. Iking A, Grundmann S, Chatzigeorgakidis E, Phan NQ, Klein D, Ständer S. Prurigo as a symptom of atopic and non-atopic diseases: aetiological survey in a consecutive cohort of 108 patients. J Eur Acad Dermatol Venereol. 2013;27(5):550-7.

3. Mollanazar NK, Elgash M, Weaver L, Valdes-Rodriguez R, Hsu S. Reduced itch associated with dupilumab treatment in 4 patients with prurigo nodularis. JAMA Dermatol. 2019;155(1):121-2.

4. Beck KM, Yang EJ, Sekhon S, Bhutani T, Liao W. Dupilumab treatment for generalized prurigo nodularis. JAMA Dermatol. 2019;155(1):118-20.

5. Rambhia PH, Levitt JO. Recalcitrant prurigo nodularis treated successfully with dupilumab. JAAD Case Rep. 2019;5(5):471-3.

6. Calugareanu A, Jachiet M, Lepelletier C, De Masson A, Rybojad M, Bagot M, et al. Dramatic improvement of generalized prurigo nodularis with dupilumab. J Eur Acad Dermatol Venereol. 2019; 33(8):e303-4.

7. Zhai LL, Savage KT, Qiu CC, Jin A, Valdes-Rodriguez R, Mollanazar NK. Chronic Pruritus Responding to Dupilumab-A Case Series. Medicines (Basel). 2019;6(3):E72.

8. Weigelt N, Metze D, Stander S. Prurigo nodularis: systematic analysis of 58 histological criteria in 136 patients. J Cutan Pathol. 2010;37(5):578-86

9. Yang TB, Kim BS. Pruritus in allergy and immunology. J Allergy Clin Immunol. 2019;144(2):353-60.

10. Pereira MP, Steinke S, Zeidler C, Forner C, Riepe C, Augustin M, et al. European academy of dermatology and venereology European prurigo project: expert consensus on the definition, classification and terminology of chronic prurigo. J Eur Acad Dermatol Venereol. 2018;32(7):1059-65.

11. Zeidler C, Ständer S. The pathogenesis of Prurigo nodularis--'Super-Itch' in exploration. Eur J Pain. 2016;20(1):37-40.

12. Fukushi S, Yamasaki K, Aiba S. Nuclear localization of activated STAT6 and STAT3 in epidermis of prurigo nodularis. Br J Dermatol. 2011;165(5):990-6.

13. Tokura Y, Yagi H, Hanaoka K, Ito T, Towyama K, Sachi Y, et al. Subacute and chronic prurigo effectively treated with recombination interferon-gamma: implications for participation of Th2 cells in the pathogenesis of prurigo. Acta Derm Venereol. 1997;77(3):231-4.

14. Oetjen LK, Mack MR, Feng J, Whelan TM, Niu H, Guo CJ, et al. Sensory neurons co-opt classical immune signaling pathways to mediate chronic itch. Cell. 2017;171(1):217-28.

15. Boozalis E, Tang O, Patel S, Semenov YR, Pereira MP, Stander S, et al. Ethnic differences and comorbidities of 909 prurigo nodularis patients. J Am Acad Dermatol. 2018;79(4):714-9.

16. Steinke S, Zeidler C, Riepe C, Bruland P, Soto-Rey I, Storck M, et al. Humanistic burden of chronic pruritus in patients with inflammatory dermatoses: Results of the European Academy of Dermatology and Venereology Network on Assessment of Severity and Burden of Pruritus (PruNet) cross-sectional trial. J Am Acad Dermatol. 2018;79(3):457-63.

17. Tanaka M, Aiba S, Matsumura N, Aoyama H, Tagami H. Prurigo nodularis consists of two distinct forms: early-onset atopic and late-onset non-atopic. Dermatology. 1995;190(4):269-76.

18. Kowalski EH, Kneiber D, Valdebran M, Patel U, Amber KT. Treatment-resistant prurigo nodularis: challenges and solutions. Clin Cosmet Investig Dermatol. 2019;12:163-72.

19. Novartis Laboratories. NEORAL Soft Gelatin Capsules (cyclosporine capsules, USP) MODIFIED NEORAL Oral Solution (cyclosporine oral solution, USP) MODIFIED. Prescribing information. 2009.

20. Ständer S, Kwon P, Hirman J, Perlman AJ, Weisshaar E, Metz M, et al. Serlopitant reduced pruritus in patients with prurigo nodularis in a phase 2, randomized, placebo-controlled trial. J Am Acad Dermatol. 2019;80(5):1395-402.

21. Verweyen E, Ständer S, Kreitz K, Höben I, Osada N, Gernart M, et al. Validation of a Comprehensive Set of Pruritus Assessment Instruments: The Chronic Pruritus Tools Questionnaire PRURITOOLS. Acta Derm Venereol. 2019;99(7):657-63.

22. Reich A, Bożek A, Janiszewska K, Szepietowski JC. 12-Item Pruritus Severity Scale: Development and Validation of New Itch Severity Questionnaire. Biomed Res Int. 2017;2017:3896423.

23. Reich A, Riepe C, Anastasiadou Z, Mędrek K, Augustin M, Szepietowski JC, et al. Itch Assessment with Visual Analogue Scale and Numerical Rating Scale: Determination of Minimal Clinically Important Difference in Chronic Itch. Acta Derm Venereol. 2016;96(7):978-80.

24. Phan NQ, Blome C, Fritz F, Gerss J, Reich A, Ebata T, et al. Assessment of pruritus intensity: prospective study on validity and reliability of the visual analogue scale, numerical rating scale and verbal rating scale in 471 patients with chronic pruritus. Acta Derm Venereol. 2012;92(5):502-7.

25. Pölking J, Zeidler C, Schedel F, Osada N, Augustin M, Metze D, et al. Prurigo Activity Score (PAS): validity and reliability of a new instrument to monitor chronic prurigo. J Eur Acad Dermatol Venereol. 2018;32(10):1754-60.

VV-CLIN-0579818 1.0

26. Chernyshov PV. The evolution of quality of life assessment and use in dermatology. Dermatology. 2019;235(3):167-74.

27. Finlay AY, Khan GK. Dermatology Life Quality Index (DLQI)--a simple practical measure for routine clinical use. Clin Exp Dermatol. 1994;19(3):210-6.

28. Zigmond AS, Snaith RP. The hospital anxiety and depression scale. Acta Psychiatr Scand. 1983;67(6):361-70.

29. Herrmann C. International experiences with the Hospital Anxiety and Depression Scale--a review of validation data and clinical results. J Psychosom Res. 1997;42(1):17-41.

30. Herdman M, Gudex C, Lloyd A, Janssen M, Kind P, Parkin D, et al. Development and preliminary testing of the new five-level version of EQ-5D (EQ-5D-5L). Qual Life Res. 2011;20(10):1727-36.

31. Sampson HA, Munoz-Furlong A, Campbell RL, Adkinson NF, Bock SA, Branum A, et al. Second symposium on the definition and management of anaphylaxis: Summary report - Second National Institute of Allergy and Infectious Disease/Food Allergy and Anaphylaxis Network symposium. J Allergy Clin Immunol. 2006;117(2):391-7.

Signature Page for VV-CLIN-0579818 v1.0
efc16460-16-1-1-amended-protocol01

Approve & eSign	[redacted]
Approve & eSign	

SANOFI

STATISTICAL ANALYSIS PLAN

**Protocol title:**	**A randomized, double blind, placebo-controlled, multi-center, parallel group study to evaluate the efficacy and safety of dupilumab in patients with prurigo nodularis who are inadequately controlled on topical prescription therapies or when those therapies are not advisable**
**Protocol number:**	**EFC16460**
**Compound number (INN/Trademark):**	**SAR231893/REGN668 dupilumab/Dupixent**
**Study phase:**	**Phase 3**
**Short title:**	**Study of dupilumab for the treatment of patients with prurigo nodularis, inadequately controlled on topical prescription therapies or when those therapies are not advisable**<br>**LIBERTY-PN PRIME 2**
**Statistician:**	[redacted]
**Statistical project leader:**	[redacted]
**Date of issue:**	**08-Aug-2021**

Regulatory agency identifier number(s):

IND:	IND107969
EudraCT:	2019-003801-90
NCT:	NCT04202679
WHO:	U1111-1241-8174
Other:	Not applicable

Total number of pages: 64

Any and all information presented in this document shall be treated as confidential and shall remain the exclusive property of Sanofi (or any of its affiliated companies). The use of such confidential information must be restricted to the recipient for the agreed purpose and must not be disclosed, published or otherwise communicated to any unauthorized persons, for any reason, in any form whatsoever without the prior written consent of Sanofi (or the concerned affiliated company); 'affiliated company' means any corporation, partnership or other entity which at the date of communication or afterwards (i) controls directly or indirectly Sanofi, (ii) is directly or indirectly controlled by Sanofi, with 'control' meaning direct or indirect ownership of more than 50% of the capital stock or the voting rights in such corporation, partnership or other entity

TABLE OF CONTENTS

STATISTICAL ANALYSIS PLAN 1

TABLE OF CONTENTS 2

LIST OF TABLES 4

VERSION HISTORY 5

1 INTRODUCTION 7

1.1 STUDY DESIGN 7

1.2 OBJECTIVE AND ENDPOINTS 7
1.2.1 Estimands 10

2 SAMPLE SIZE DETERMINATION 14

3 ANALYSIS POPULATIONS 15

4 STATISTICAL ANALYSES 17

4.1 GENERAL CONSIDERATIONS 17

4.2 PARTICIPANT DISPOSITIONS 17

4.3 PRIMARY ENDPOINT(S) ANALYSIS 18
4.3.1 Definition of endpoint(s) 18
4.3.2 Main analytical approach 20
4.3.3 Sensitivity analysis 21
4.3.4 Supplementary analyses 21
4.3.5 Subgroup analyses 22

4.4 SECONDARY ENDPOINT(S) ANALYSIS 23
4.4.1 Key/Confirmatory secondary endpoint(s) 23
4.4.1.1 Definition of endpoint(s) 23
4.4.1.2 Main analytical approach 23
4.4.2 Supportive secondary endpoint(s) 24

4.5 TERTIARY/EXPLORATORY ENDPOINT(S) ANALYSIS 26
4.5.1 Definition of endpoint(s) 26
4.5.2 Main analytical approach 29

4.6 MULTIPLICITY ISSUES 29

4.7 SAFETY ANALYSES 30
4.7.1 Extent of exposure 30
4.7.2 Adverse events 31
4.7.3 Additional safety assessments....................36
4.7.3.1 Laboratory variables, vital signs and electrocardiograms (ECGs)....................36
4.8 OTHER ANALYSES....................38
4.8.1 Pharmacokinetic analyses 38
4.8.2 Immunogenicity analyses....................39
4.8.3 Pharmacodynamic/genomics endpoints 41
4.9 INTERIM ANALYSES 42
5 SUPPORTING DOCUMENTATION 43
5.1 APPENDIX 1 LIST OF ABBREVIATIONS 43
5.2 APPENDIX 2 CHANGES TO PROTOCOL-PLANNED ANALYSES 45
5.3 APPENDIX 3 DEMOGRAPHICS AND BASELINE CHARACTERISTICS, PRIOR OR CONCOMITANT MEDICATIONS/PROCEDURES....................46
5.4 APPENDIX 4 DATA HANDLING CONVENTIONS 50
5.5 APPENDIX 5 SAMPLE SAS CODE....................57
5.6 APPENDIX 6 SELECTION CRITERIA FOR AE/MEDICATION GROUPINGS 59
5.7 APPENDIX 7 MEDICATION/PROCEDURE ADJUDICATION ALGORITHM....................61
6 REFERENCES....................64

LIST OF TABLES

Table 1 - Major changes in statistical analysis plan 5
Table 2 - Objectives and endpoints 7
Table 3 - Summary of primary estimand for main endpoints 11
Table 4 - Populations for analyses 15
Table 5 - Prohibited medications/procedures and rescue medications that impact efficacy 19
Table 6 - Sorting of AE tables 32
Table 7 - Analyses of adverse events 33
Table 8 - Selections for AESIs and other AEs of interest 34
Table 9 - Major statistical changes in protocol amendment(s) 45
Table 10 - Time window for eDiary efficacy variables 51
Table 11 - Time window for efficacy variables 53
Table 12 - Time window for safety endpoints 55
Table 13 - Time window for pharmacokinetics/pharmacodynamics variables 56
Table 14 - List of PTs or Medications for CMQs/CDGs 59

VERSION HISTORY

This Statistical Analysis Plan (SAP) for study EFC16460 is based on the Protocol Amendment 1 dated 20-May-2020 and a health authority's feedback on EFC16459 Protocol Amendment 2 dated 14-Apr-2021 (See EFC16459 SAP for details). This section summarizes major changes to the statistical analysis features in the SAP. All changes to the statistical analysis features from the original protocol to Amendment 1 are described in Appendix 2.

Table 1 - Major changes in statistical analysis plan

SAP Version	Approval Date	Changes	Rationale
1.0	04-Feb-2021	Not Applicable	Original version
2.0	Current version	Add objective "To demonstrate efficacy of dupilumab on both itch as well as skin lesions within the same participant" and multicomponent endpoint (Proportion of participants with both an improvement (reduction) in WI-NRS by ≥4 from baseline to Week 24 and an IGA PN-S 0 or 1 score at Week 24) as a key secondary endpoint for the US and US reference countries hierarchy.	To add this multicomponent endpoint as another measure of treatment success based on feedback from a health authority.
		Add supplementary analysis (tipping point analyses) for primary and key secondary endpoints	This additional supplementary analysis was recommended by a health authority to assess whether the estimate, and the inference thereof, was robust to departure from the strategies used in primary analysis for handling intercurrent events and missing data.
		Add subgroup analyses for key secondary endpoints.	To evaluate whether the treatment effect is consistent across pre-specified sub-groups on the key secondary endpoints in addition to the primary endpoint.
		Add subgroup analyses for participants who have been impacted (or not) by COVID-19	To address a health authority's request to assess the impact of the treatment effect by COVID-19.
		Modify hierarchical order for multiplicity procedure for US and US reference countries	To include the multicomponent endpoint in the hierarchy to address the health authority's feedback requesting assessment of this endpoint as another measure of treatment success.
		Add as-observed plus multiple imputation supplementary analysis for the secondary endpoint (percent change from baseline in WI-NRS at Week 24)	To explore the robustness of the estimate, and the inference thereof, with a different strategy for handling intercurrent events and missing data
		Remove "selected" in the text including "selected prohibited and/or rescue medication"	Since a participant who takes any protocol specified prohibited medications/procedures and/or rescue

SAP Version	Approval Date	Changes	Rationale
			medications is considered as a non-responder after medical adjudication/confirmation conducted in a blinded fashion, use of the word "selected" is confusing.
		Add additional exposure-adjusted adverse event incidence rate tables that will provide the number of patients with at least 1 event per 100 patient-years.	As requested by a health authority to include exposure-adjusted incidence rate and patient years in the summaries for TEAEs and AESIs.
		Added “Keratitis FDA” to other AE groupings	For consistency with other dupilumab studies

The first participant was randomized on 2020-02-03.

1 INTRODUCTION

1.1 STUDY DESIGN

This study is a multi-center, 24-week treatment, parallel group, double-blind, randomized, placebo-controlled study to evaluate the use of dupilumab in participants with prurigo nodularis (PN) inadequately controlled on topical prescription therapies or when those therapies are not advisable. The study will assess the effect of dupilumab on itch improvement as well as its effect on PN lesions, on participants' health-related quality of life (HRQoL), anxiety and depression, sleep quality, skin pain, and overall health status.

After 2-4 weeks of screening, participants will be centrally randomized using a permuted block randomization schedule via Interactive Voice Response System/Interactive Web Response System (IVRS/IWRS) in a 1:1 randomization ratio to dupilumab 300 mg q2w or matching placebo. Randomization will be stratified by documented history of atopy (atopic or non-atopic), stable use of topical corticosteroids (TCS)/ topical calcineurin inhibitors (TCI) (yes or no), and country/territory code.

A total of approximately 150 participants will be randomized to two treatment arms (75 participants/arm). The number of participants with active mild AD upon study entry will represent up to 10% of the atopic participants. Both the atopic and the non-atopic PN populations will each be capped at 60% of the total enrolled population.

The study duration consists of the following periods:

- Screening period (2-4 weeks)
- Randomized IMP intervention period (24 weeks)
- Follow-up period (12 weeks)

1.2 OBJECTIVE AND ENDPOINTS

Table 2 - Objectives and endpoints

Objectives	Endpoints
**Primary**	
• To demonstrate the efficacy of dupilumab on itch response in participants with PN, inadequately controlled on topical prescription therapies or when those therapies are not advisable.	• Proportion of participants with improvement (reduction) in worst-itch numeric rating scale (WI-NRS) by ≥4 from baseline to Week 12.
**Secondary**	
• To demonstrate the efficacy of dupilumab on additional itch endpoints in participants with PN, inadequately controlled on topical prescription therapies or when those therapies are not advisable.	• Proportion of participants with improvement (reduction) in WI-NRS by ≥4 from baseline to Week 24 **[Key secondary endpoint]**.<br>• Time to onset of effect on pruritus as measured by proportion of participants with an improvement (reduction) in WI-NRS by ≥4 from baseline during the 24-week treatment period.

Objectives	Endpoints
	• Change from baseline in WI-NRS at Week 24.<br>• Change from baseline in WI-NRS at Week 12.<br>• Percent change from baseline in WI-NRS at Week 24.<br>• Percent change from baseline in WI-NRS at Week 12.<br>• Percent change from baseline in WI-NRS at Week 4.<br>• Percent change from baseline in WI-NRS at Week 2.<br>• Percent change from baseline in WI-NRS over time until Week 24.<br>• Proportion of participants with WI-NRS reduction ≥4 at Week 4.<br>• Proportion of participants with WI-NRS reduction ≥4 over time until Week 24[a].<br>• Onset of action in change from baseline in WI-NRS (first p <0.05 difference from placebo in the daily WI-NRS that remains significant at subsequent measurements) until Week 12[b].
• To demonstrate efficacy of dupilumab on skin lesions of PN.	• Proportion of participants with Investigator's Global Assessment 0 or 1 score for PN-Stage (IGA PN-S) at Week 24 **[Key secondary endpoint]**.<br>• Proportion of participants with IGA PN-S 0 or 1 score at Week 12.<br>• Proportion of participants with IGA PN-S 0 or 1 score at Week 8[c].<br>• Proportion of participants with IGA PN-S 0 or 1 score at Week 4[c].<br>• Change from baseline in IGA PN-S score at Week 24.<br>• Change from baseline in IGA PN-S score at Week 12.<br>• Change from baseline in IGA PN-S score at Week 8.<br>• Change from baseline in IGA PN-S score at Week 4.<br>• Proportion of participants with Investigator's Global Assessment 0 or 1 score for PN-Activity (IGA PN-A) at Week 24.<br>• Proportion of participants with IGA PN-A 0 or 1 score at Week 12.<br>• Proportion of participants with IGA PN-A 0 or 1 score at Week 8.<br>• Proportion of participants with IGA PN-A 0 or 1 score at Week 4.
• To demonstrate efficacy of dupilumab on both itch as well as skin lesions within the same participant	• Proportion of participants with both an improvement (reduction) in WI-NRS by ≥4 from baseline to Week 24 and an IGA PN-S 0 or 1 score at Week 24 **(key secondary endpoint** for US and US reference countries only)
• To demonstrate the improvement in health-related quality of life (HRQoL).	• Change from baseline in HRQoL, as measured by Dermatology Life Quality Index (DLQI) to Week 24.

Objectives	Endpoints
	• Change from baseline in HRQoL, as measured by DLQI to Week 12.
• To evaluate safety outcome measures.	• Percentage of participants experiencing treatment-emergent adverse events (TEAEs) or serious adverse events (SAEs) from baseline through Week 24.
• To evaluate immunogenicity of dupilumab.	• Incidence of treatment-emergent antidrug antibodies (ADA) against dupilumab over time.
**Tertiary/exploratory**	
• To demonstrate a reduction in the use of rescue medication and systemic immunosuppressant	• Use of high potency or superpotent TCS rescue medication through Week 24<br>• Use of systemic immunosuppressants through Week 24, constituting treatment failure
• To evaluate exploratory outcome measures	• Change from baseline in Hospital Anxiety and Depression Scale (HADS) total score to Week 24.<br>• Change from baseline in EQ5D-5L to Week 24.<br>• Change from baseline in skin Pain numeric rating scale (NRS) to Week 4, Week 8, Week 12, and Week 24, respectively.<br>• Change from baseline in Sleep NRS to Week 4, Week 8, Week 12, and Week 24, respectively.<br>• Missed school/work days through Week 24.<br>• Incidence of skin-infection TEAEs (excluding herpetic infections) through Week 24.
• To evaluate the efficacy of dupilumab on skin lesions using a modified PAS 5-item questionnaire	• Proportion of participants who achieve ≥75% healed lesions from Prurigo Activity Score (PAS) at Week 4, Week 8, Week 12, and Week 24, respectively.<br>• Change from baseline in exact number of lesions in representative area (as determined from PAS) at Week 4, Week 8, Week 12, and Week 24, respectively.
• To evaluate the efficacy of dupilumab on other PN endpoints	• Change from baseline in Participant Global Impression of Severity (PGIS) of PN to Week 4, Week 8, Week 12, and Week 24, respectively.<br>• Participant Global Impression of Change (PGIC) of PN at Week 4, Week 8, Week 12, and Week 24, respectively.
**PK**	
• To evaluate pharmacokinetic (PK) and pharmacodynamic (PD) outcome measures	• Serum functional dupilumab concentrations and PK profile.<br>• Pharmacodynamic response for selected biomarkers (total IgE).

a The early onset for significant change in the proportion of responders with WI-NRS reduction ≥4 between dupilumab and placebo will be claimed at Week xx if the p-value for Week xx is <0.05 and the remaining timepoints also have nominal p-value ≤0.05 until Week 12.

b The early onset for significant change in WI-NRS from baseline between dupilumab and placebo will be claimed at Week xx if the p-value for Week xx is <0.05 and the remaining timepoints also have nominal p-value ≤0.05 until Week 12.

c The early onset for reaching IGA PN-S(0,1) between dupilumab and placebo will be claimed at Week xx if the p-value for Week xx is <0.05 and the remaining timepoints also have nominal p-value ≤0.05 until Week 12.

1.2.1 Estimands

The primary estimands defined for main endpoints are summarized in below Table 3. More details are provided in Section 4.

Table 3 - Summary of primary estimand for main endpoints

Endpoint Category	Estimands			
	**Endpoint(s)[a]**	**Population**	**Intercurrent event(s) strategy and missing data handling**	**Population-level summary**
**Primary objective: To demonstrate the efficacy of dupilumab on itch response in participants with PN, inadequately controlled on topical prescription therapies or when those therapies are not advisable.**				
Primary endpoint	Proportion of participants with improvement (reduction) in worst-itch numeric rating scale (WI-NRS) by ≥4 from baseline to Week 12	ITT	The intercurrent events will be handled as follows:<br>• Discontinuation of study treatment before Week 12: Off-study treatment data up to Week 12 will be included in the analysis (treatment policy strategy).<br>• Taking the prohibited medications/procedures and/or rescue medications[b] prior to Week 12: Participants will be considered as non-responders (composite strategy).<br>In addition, the missing data imputation rules are as follows:<br>• Having missing data at Week 12: Participants will be considered as non-responders.	CMH test adjusted by documented history of atopy (atopic or non-atopic), stable use of TCS/TCI (yes or no), region (countries combined), and baseline antidepressant use (yes or no).

Endpoint Category	Estimands			
	Endpoint(s)[a]	Population	Intercurrent event(s) strategy and missing data handling	Population-level summary
**Secondary objective: To demonstrate the efficacy of dupilumab on additional itch endpoints in participants with PN, inadequately controlled on topical prescription therapies or when those therapies are not advisable.**				
Secondary endpoint	Time to onset of effect on pruritus as measured by proportion of participants with an improvement (reduction) in WI-NRS by ≥4 from baseline during the 24-week treatment period	ITT	The intercurrent events will be handled as follows:<br>• Discontinuation of study treatment before Week 24: Off-study treatment data up to Week 24 will be included in the analysis (treatment policy strategy).<br>• Taking the prohibited medications/procedures and/or rescue medications[b] prior to Week 24: Analyses will be censored at Week 24 (composite strategy).<br>In addition, the missing data imputation rules are as follows:<br>• Discontinuing the study follow-up before Week 24: Analyses will be censored at the time of last WI-NRS assessment.	This time-to-event endpoint will be analyzed using the Cox proportional hazards model, including intervention group, documented history of atopy (atopic or non-atopic), stable use of TCS/TCI (yes or no), region (countries combined), and baseline antidepressant use (yes or no). The hazards ratio, its 95% confidence interval and p-value will be reported. Kaplan-Meier curves will be also provided.

Endpoint Category	Estimands			
	Endpoint(s)[a]	Population	Intercurrent event(s) strategy and missing data handling	Population-level summary
Secondary endpoint	Change from baseline in WI-NRS at Week 24	ITT	The intercurrent events will be handled as follows:<br>• Discontinuing the study treatment: all data collected following schedule after treatment discontinuation will be used in the analysis (treatment policy strategy).<br>• Taking the prohibited medications/procedures and/or rescue medications[b] prior to Week 24: data will be set to missing values after the medication usage, and the participant's worst postbaseline value on or before the time of the medication usage will be used to impute missing endpoint value (for participants whose postbaseline values are all missing, the participant's baseline will be used to impute the missing endpoint value) (hypothetical strategy)<br><br>In addition, the missing data imputation rules are as follows:<br>• After discontinuation of the study treatment due to lack of efficacy prior to Week 24: WOCF approach will be used to impute missing data if needed[2].<br>• After discontinuation of the study treatment due to reasons other than lack of efficacy prior to Week 24: multiple imputation (MI) approach will be used to impute missing endpoint value, and this multiple imputation will use all participants excluding participants who have taken the prohibited medications and/or rescue medications prior to Week 24 and excluding participants who discontinue due to lack of efficacy prior to Week 24.	ANCOVA model with intervention group, documented history of atopy (atopic or non-atopic), stable use of TCS/TCI (yes or no), region (countries combined), baseline antidepressant use (yes or no), and relevant baseline measurement as covariates is used. Statistical inference obtained from all imputed data by ANCOVA model will be combined using Rubin's rule.

a Additional secondary objectives/endpoints are not included in this table but would be handled with a similar strategy as the endpoint type (ie continuous, proportion, time-to-event) at other weeks

b Prohibited medications and/or rescue medications are listed in Table 5.

2 SAMPLE SIZE DETERMINATION

The primary endpoint is the proportion of participants with WI-NRS reduction of ≥4 from baseline to Week 12. By assuming the response rate is [redacted] in the placebo and dupilumab arms, respectively, 56 participants/arm will provide 90% power to detect the difference of [redacted] between dupilumab and placebo with Fisher exact test at 2-sided level of 0.05. Assuming 15% drop out during the 12 weeks of treatment, the target is to randomize 75 participants/arm with a cap of up to 10% of participants in the atopic population having active mild AD.

The assumptions were based on the effect of dupilumab versus placebo in WI-NRS reduction ≥4 at Week 16 observed in participants with moderate to severe AD as seen in studies of AD-1334 (Solo1) and AD-1416 (Solo2).

Approximately 150 participants will be randomized to dupilumab or placebo in a 1:1 ratio with stratification factors of documented history of atopy (atopic or non-atopic), stable use of TCS/TCI (yes or no), and country/territory code. Both the atopic and the non-atopic PN population will be capped at 60% of the total enrolled population.

The sample size calculation was performed using SAS 9.4 power procedure.

VV-CLIN-0603750 2.0

3 ANALYSIS POPULATIONS

The following populations for analyses are defined:

Table 4 - Populations for analyses

Population	Description
Screened	All participants who sign the ICF.
Randomized	The randomized population includes all participants with a treatment kit number allocated and recorded in the interactive response technology (IRT) database, and regardless of whether the treatment kit was used or not.<br>Participants treated without being randomized will not be considered randomized and will not be included in any efficacy population.
Intent-to-treat (ITT)	All randomized participants analyzed according to the intervention group allocated by randomization regardless if treatment kit is used or not
Efficacy	The ITT population
Safety	All participants randomly assigned to study intervention and who take at least 1 dose of study intervention. Participants will be analyzed according to the intervention they actually received.<br>Randomized participants for whom it is unclear whether they took the study medication will be included in the safety population as randomized.<br>For participants who accidentally receive different treatment from the planned, the actual intervention allocation for as-treated analysis will be the dupilumab group.<br>The PD analyses will be performed on the safety population.
Pharmacokinetic (PK)	The PK population includes all participants in the safety population with at least one non-missing result for functional dupilumab concentration in serum after first dose of the study treatment. Participants will be analyzed according to the intervention actually received.
Antidrug antibody (ADA)	ADA population includes all participants in the safety population who have at least one non-missing ADA result after first dose of the study treatment. Participants will be analyzed according to the intervention actually received.

ADA: antidrug antibody; ICF: informed consent form; ITT: intent to treat; PD: pharmacodynamics: PK: pharmacokinetic.

Participants exposed to study intervention before or without being randomized will not be considered randomized and will not be included in any analysis population. The safety experience of these participants will be reported separately.

Randomized participants for whom it is unclear whether they took the study intervention will be considered as exposed and will be included in the safety population as randomized.

For any participant randomized more than once, only the data associated with the first randomization will be used in any analysis population. The safety experience associated with any later randomization will be reported separately.

For participants receiving more than one study intervention (placebo and dupilumab) during the study, the intervention group for as-treated analysis will be the dupilumab group.

Regarding the COVID-19 pandemic, additional summaries by COVID-19 subgroups (i.e., impacted by the COVID-19 pandemic and NOT impacted by the COVID-19 pandemic) will be provided to assess the impact of COVID-19 on treatment effect. Participants impacted by the COVID-19 pandemic are defined as randomized participants with any critical or major deviation related to COVID-19 or who permanently discontinued study intervention or study due to COVID-19.

4 STATISTICAL ANALYSES

4.1 GENERAL CONSIDERATIONS

In general, continuous data will be summarized using the number of observations available, mean, standard deviation (SD), median, minimum, and maximum. Categorical and ordinal data will be summarized using the count and percentage of participants.

The baseline value of efficacy parameters is defined as the last available value before randomization and prior to the first dose of study medication unless below eDiary data (WI-NRS, skin Pain-NRS and Sleep-NRS) score or otherwise specified.

The baseline for weekly average WI-NRS (skin Pain-NRS and Sleep-NRS) score is defined as the average of daily non-missing scores obtained during the 7 days prior to randomization.

The baseline value of the other parameters is defined as the last available value prior to the first dose of investigational medicinal product (IMP) if the participant is treated, or the last available value up to randomization if the participant is not exposed to IMP.

Observation period

The observation period will be divided into 4 segments:

- The **pre-treatment period** is defined as the period up to first IMP administration.
- The **treatment-emergent (TE) period** is defined as the period from the first IMP administration to the last IMP administration + 98 days. The treatment-emergent period includes the following 2 periods:
  - The **on-treatment period** is defined as the period from the first IMP administration to the last administration of the IMP + 14 days
  - The **residual treatment period** is defined as the period from the end of the on-treatment period to the end of the treatment-emergent period.
- The **post-treatment period** is defined as the period from the end of the treatment-emergent period.

The on-study observation period is defined as the time from start of intervention until the end of the study defined as the status date collected on e-CRF page "Completion of End of Study".

4.2 PARTICIPANT DISPOSITIONS

The number (%) of participants included in each of the analysis populations listed in Table 4 will be summarized.

Screen failures are defined as participants who consent to participate in the study but are not subsequently randomized. The number (%) of screen failures and reasons for screen failures will be provided in the screened population.

The number (%) of participants in the following categories will be provided:

- Randomized participants
- Randomized but not exposed participants
- Randomized and exposed participants
- Participants who completed the study treatment period as per protocol
- Participants who did not complete the study treatment period as per protocol and discontinued study treatment prior to Week 12 by main reason for permanent intervention discontinuation including due to COVID-19
- Participants who did not complete the study treatment period as per protocol and discontinued study treatment prior to Week 24 by main reason for permanent intervention discontinuation including due to COVID-19
- Participants who completed the study period as per protocol
- Participants who did not complete the study period as per protocol and discontinued study by main reason for study discontinuation including due to COVID-19.
- Vital status at last study contact

The number (%) of exposed and not randomized participants will also be summarized.

In addition, the number (%) of participants screened, screened-failed, randomized, with permanent intervention discontinuation and with early study discontinuation will be provided by country and site.

Protocol deviations

Critical and major protocol deviations (automatic or manual) will be summarized in the randomized population and according to COVID-19 impact (i.e. deviations related to the COVID-19 pandemic and deviations not related to the COVID-19 pandemic). In addition, deviations potentially impacting the primary endpoint analysis will be summarized.

4.3 PRIMARY ENDPOINT(S) ANALYSIS

4.3.1 Definition of endpoint(s)

The primary endpoint for this study is the proportion of participants with improvement (reduction) in worst-itch numeric rating scale (WI-NRS) by ≥4 from baseline to Week 12.

WI-NRS is a patient-reported outcome (PRO) comprised of a single item rated on a scale from 0 (“No itch”) to 10 (“Worst imaginable itch”). Participants are asked to rate the intensity of their worst pruritus (itch) over the past 24 hours using this scale.

The weekly average WI-NRS score at each week, which is defined as the average of daily non-missing scores within the week window of each week (see Section 5.4), will be used for analyses.

For efficacy analysis, Table 5 presents the prohibited and rescue medications/procedures which will be considered as intercurrent events if the last column indicates as "Yes" and therefore be handled in the estimands for endpoints defined in Table 3.

Table 5 - Prohibited medications/procedures and rescue medications that impact efficacy

Medication/procedure	Comment	Data to be set as non-responder after taking medication in the main statistical analysis (Yes/No) [a]/ Selection criteria
**Prohibited medications/procedures**		
Systemic immunosuppressive/immunomodulating drugs (eg, systemic corticosteroids, cyclosporine, mycophenolate-mofetil, interferon gamma, Janus kinase inhibitors, azathioprine, methotrexate, hydroxychloroquine, dapsone, sulfasalazine, colchine, etc)	IMP to be discontinued	Yes (CDG[b] Immunosuppressant drugs Narrow and SDG[b]Corticosteroids Narrow)
Other monoclonal antibodies (that are biological response modifiers).	IMP to be discontinued	Yes (SDG[b] Monoclonal antibodies Narrow)
Phototherapy, including tanning beds.	IMP to be discontinued	Yes (CMQ[b] HLT Phototherapies, CMQ[b] Tanning_single PT)
Naltrexone or other opioid antagonist	IMP to be discontinued	Yes (SDG[b] Analgesia producing opioids NARROW)
Gabapentin, pregabalin, and thalidomide	IMP to be discontinued	Yes (CDG[b] Gabapentin mono and multi ingredients, Pregabalin mono and multi ingredients, or Thalidomide mono and multi ingredients)
Paroxetine, fluvoxamine or other SSRIs.	No IMP discontinuation, see requirement in the footnote	Yes[c,d] (CDG[b] N06AB selective serotonin reuptake inhibitors)
SNRIs.	No IMP discontinuation, see requirement in the footnote	Yes[c,d] (CDG[b] serotonin and norepinephrine reuptake inhibitors)
Amitriptyline or other tricyclic or tetracyclic antidepressants	No IMP discontinuation, see requirement in the footnote	Yes[c,d] (CDG[b] N06AA non-selective monoamine reuptake inhibitors)
Intralesional corticosteroid injections and cryotherapy.	No IMP discontinuation	Yes[d] (CMQ[b] Injection_single pt, cryotherapy or skin cryotherapy PT)
Sedating antihistamine	No IMP discontinuation	Yes[d,e] (CDG[b] sedating antihistamines)
Non-sedating antihistamine if used specifically for the treatment of itch secondary to AD or PN	No IMP discontinuation	Yes[d,e] (CDG[b] non-sedating antihistamines)

Medication/procedure	Comment	Data to be set as non-responder after taking medication in the main statistical analysis (Yes/No) [a]/ Selection criteria
**Rescue medications**		
Dermatological preparations of high potency or superpotent TCS and TCI	No IMP discontinuation	Yes[d] (CDG[b] calcineurin inhibitors and corticosteroids narrow, Ticked 'RESCUE THERAPY' in CRF page)

a. When yes, the estimand for the intercurrent event handling strategy will be as follows: hypothetical for continuous endpoints, and composite for responder and time-to-event endpoints. When no, a treatment policy strategy will be applied.
b. CDG=company drug groupings, CMQ=company MedDRA query, SDG= Standardized drug groupings.
c. Only if the antidepressant is initiated during the study or its dose is increased from baseline provided that medication was taken at least 3 months prior to screening.
d. As per medical adjudication, see Appendix 7 for details.
e. Only if the medication is initiated at Week 12 or Week 24, or the dose is increased from Week 11 to Week 12 or from Week 23 to Week 24.

Blinded review of prohibited/rescue treatment (medication or procedure) based on Table 5 and a pre-specified algorithm (Appendix 7) will be implemented before database locks by considering the type of medication or procedure, indication, timing, frequency and the potential impact of the use of the prohibited medications/procedures and rescue medications.

4.3.2 Main analytical approach

The primary estimand for the primary endpoint is the treatment policy/composite approach as defined in Table 3.

The primary analysis population for the efficacy endpoints will be the ITT population.

The following null hypothesis H0 and alternative hypothesis H1 will be tested for dupilumab against placebo:

- H0: No treatment difference between dupilumab and placebo.
- H1: There is a treatment difference between dupilumab and placebo

The primary analysis will be conducted by using Cochran–Mantel–Haenszel test (CMH) test adjusted by documented history of atopy (atopic or non-atopic), stable use of TCS/TCI (yes or no), region, and baseline anti-depressant use (yes or no). Comparisons of the response rates between dupilumab and placebo will be derived. In addition, odds ratio and response rate difference as well as the corresponding 95% confidence intervals (CI) will be provided along with the p-values.

As defined in Table 3 for participants discontinuing the study treatment before Week 12, their off-study treatment values measured up to Week 12 will be included in the analysis. Participants taking the prohibited medications/procedures and/or rescue medications (see Table 5) prior to Week 12 or having missing data at Week 12 will be considered non-responders.

4.3.3 Sensitivity analysis

For the primary estimand for the primary endpoint, no sensitivity analysis will be performed. However, three supplementary analyses will be performed as described in the section below.

4.3.4 Supplementary analyses

The following supplementary analyses will be performed:

As-observed analysis (including all data after taking the prohibited and/or rescue medications)

The data collected after taking all prohibited medications and/or rescue medications will be included in the supplementary analysis to evaluate the robustness of the primary analysis results with respect to the method of handling data while taking the prohibited medications (e.g. treatment policy strategy). In addition, for participants discontinuing the study treatment before Week 12, their off-study treatment values measured up to Week 12 will be included in the analysis. The participants having missing data at Week 12 regardless of reason(s) will be considered non-responders at that timepoint.

Hybrid method analysis (the worst-observation carried forward (WOCF) and multiple imputation (MI))

In the primary analysis of change from baseline in WI-NRS (continuous variable) at Week 12, the hybrid method of the WOCF and MI will be used (see Section 4.4.2). Similar to the continuous variable, the same imputation method will be used in the analysis of the proportion of participants with improvement (reduction) in WI-NRS by ≥4 from baseline to Week 12, which is consistent for the intercurrent event strategy and missing data handling in the binary variables and continuous variable. That is, after the imputation of continuous WI-NRS data at Week 12 using the hybrid method of the WOCF and MI (see Section 4.4.2), responders will be defined as participants with improvement in WI-NRS by ≥4 from baseline to Week 12 in each of the imputed datasets with about 40 imputations, and then the CMH test adjusted by documented history of atopy (atopic or non-atopic), stable use of TCS/TCI (yes or no), region, and baseline anti-depressant use (yes or no) will be used. Statistical inference obtained from all imputed data will be combined using Rubin’s rule.

Tipping point analysis

The intercurrent events will be handled as follows:

- Discontinuation of study treatment before Week 12: Off-study treatment data up to Week 12 will be included in the analysis (treatment policy strategy).
- Taking the prohibited medications/procedures and/or rescue medications prior to Week 12: Data after the intercurrent events will be censored and then imputed with the below tipping point method

In addition, the missing data imputation rules are as follows:

- Having missing data at Week 12: Data will be imputed with the following tipping point method.

Tipping point method:

- A sequence of analyses will be performed with the adjustment to artificially decrease the response rate in the dupilumab group and increase the response rate in the placebo group with a fixed and definite set of values for data imputation.
- For each combination of increasing response rate in placebo and decreasing response rate in dupilumab, multiple imputed datasets will be generated and analyzed using CMH test. The results obtained from multiple imputed datasets will be combined to generate statistical inference, i.e. p-value and treatment difference between 2 treatment groups.
- A “tipping point” will be identified while the result is no longer statistically significant (i.e. p-value >0.05).

4.3.5 Subgroup analyses

To assess the homogeneity of the treatment effect across various subgroups, analyses will be performed on the primary endpoint across the following subgroups (categories with fewer than 5 participants may be combined with other categories):

- Age group (<65, ≥65 years)
- Gender (Male, Female)
- Region
- Territory
- Race (Caucasian/White, Black/of African descent, Asian/Oriental, Others)
- Ethnicity (Hispanic or Latino, Not Hispanic or Latino)
- Baseline weight (<60, ≥60- < 90, ≥ 90 kg)
- Baseline BMI (<25, ≥25- <30, ≥30 kg/m^2)
- Participants without a current diagnosis of AD
- History of atopy (atopic or non-atopic)
- Stable use of TCS/TCI (yes or no)
- Antidepressant use (yes or no) at baseline
- Baseline IGA PN-S moderate versus severe (3 vs. 4)
- Participants who have not been impacted by COVID-19 vs impacted by COVID-19 (for participants who have been impacted by the COVID-19, the efficacy data will be descriptive only if the number of participants is not enough to perform statistical tests. Participants impacted by the COVID-19 pandemic are defined as randomized participants with any critical or major deviation related to COVID-19 or who permanently discontinued study intervention or study due to COVID-19.)

To test the interaction between intervention and subgroup factor, a logistic regression model incorporating subgroup-by-treatment interaction will be built for each subgroup factor except the subgroup of participants without a current diagnosis of AD (very few AD participants will be excluded). The model will include all the covariates in the main statistical model plus the subgroup variable and the subgroup-by-treatment interaction. A p-value for the test of interaction will be provided.

In each subgroup, the treatment effects for the primary endpoint will be provided, as well as the corresponding 95% CI, using the same method as applied to the primary analysis. Forest plots will be provided.

4.4 SECONDARY ENDPOINT(S) ANALYSIS

The weekly average skin Pain-NRS or Sleep-NRS score at each week, which is defined as the average of non-missing daily scores within the week window of each week (see Section 5.4), will be used for analyses.

4.4.1 Key/Confirmatory secondary endpoint(s)

4.4.1.1 Definition of endpoint(s)

The key secondary endpoints are:

- Proportion of participants with improvement (reduction) in WI-NRS by ≥4 from baseline to Week 24, and
- Proportion of participants with IGA PN-S 0 or 1 score at Week 24.

In addition, for US and US reference countries only, there is another key secondary endpoint:

- Proportion of participants with both an improvement (reduction) in WI-NRS by ≥4 from baseline to Week 24 and an IGA PN-S 0 or 1 score at Week 24.

<u>Investigator's global assessment for prurigo nodularis (IGA PN)</u>

The IGA PN is a clinician-reported outcome (ClinRO) that allows clinicians to assess the activity of PN (IGA PN-A) using a 5-point scale from 0 (clear) to 4 (severe); and the stage of the disease (IGA PN-S) using a 5-point scale from 0 (clear) to 4 (severe).

4.4.1.2 Main analytical approach

For the key secondary efficacy endpoints, the analysis will be conducted by using CMH test adjusted by documented history of atopy (atopic or non-atopic), stable use of TCS/TCI (yes or no), region, and baseline anti-depressant use (yes or no). The same estimand, i.e., intercurrent event strategy and missing data handling method, as the primary endpoint, by replacing Week 12 with Week 24, is used.

The same supplementary and subgroup analyses used for the primary endpoint will also be performed for the key secondary efficacy endpoints.

4.4.2 Supportive secondary endpoint(s)

The other secondary endpoints are as follows:

- Proportion of participants with WI-NRS reduction $\geq$4 over time until Week 24.
- Proportion of participants with WI-NRS reduction $\geq$4 at Week 4
- Proportion of participants with IGA PN-S 0 or 1 score at Week 12
- Proportion of participants with IGA PN-S 0 or 1 score at Week 8
- Proportion of participants with IGA PN-S 0 or 1 score at Week 4
- Proportion of participants with IGA PN-A 0 or 1 score at Week 24
- Proportion of participants with IGA PN-A 0 or 1 score at Week 12
- Proportion of participants with IGA PN-A 0 or 1 score at Week 8
- Proportion of participants with IGA PN-A 0 or 1 score at Week 4
- Time to onset of effect on pruritus as measured by proportion of participants with an improvement (reduction) in WI-NRS by $\geq$4 from baseline during the 24-week treatment period
- Change from baseline in WI-NRS at Week 24
- Change from baseline in WI-NRS at Week 12
- Percent change from baseline in WI-NRS at Week 24
- Percent change from baseline in WI-NRS at Week 12
- Percent change from baseline in WI-NRS at Week 4
- Percent change from baseline in WI-NRS at Week 2
- Percent change from baseline in WI-NRS over time until Week 24.
- Onset of action in change from baseline in WI-NRS (first $p < 0.05$ difference from placebo in the daily WI-NRS that remains significant at subsequent measurements) until Week 12
- Change from baseline in IGA PN-S score at Week 24
- Change from baseline in IGA PN-S score at Week 12
- Change from baseline in IGA PN-S score at Week 8
- Change from baseline in IGA PN-S score at Week 4
- Change from baseline in HRQoL, as measured by Dermatology Life Quality Index (DLQI) to Week 24

- Change from baseline in HRQoL, as measured by Dermatology Life Quality Index (DLQI) to Week 12

Dermatology life quality index (DLQI)

The DLQI is a PRO developed to measure dermatology-specific HRQoL in adult participants. The instrument comprises 10 items assessing the impact of skin disease on participants' HRQoL over the previous week. The items cover symptoms, leisure activities, work/school or holiday time, personal relationships including intimate, the side effects of treatment, and emotional reactions to having a skin disease. It is a validated questionnaire used in clinical practice and clinical trials. Response scale is a 4-point Likert scale (0 = "not at all" and 3 = "very much") for nine items. The remaining one item about work/studying asks whether work/study has been prevented and then (if "No") to what degree the skin condition has been a problem at work/study; the item is rated on a 3-point Likert scale ("Not at all" to "A lot"). Overall scoring ranges from 0 to 30, with a high score indicative of a poor HRQoL.

Time-to-event secondary efficacy endpoint

Time to first onset of effect on pruritus defined as an improvement (reduction) in WI-NRS by ≥4 from baseline during the 24-week treatment period will be analyzed using the Cox proportional hazards model, including treatment, documented history of atopy (atopic or non-atopic), stable use of TCS/TCI (yes or no), region, and baseline anti-depressant use (yes or no). The hazards ratio, its 95% confidence interval and p-value will be reported. Kaplan-Meier curves will be also provided.

For time to first WI-NRS response (WI-NRS by ≥4 from baseline), participants who receive the prohibited medications and/or rescue medications (see Table 5), data prior to start of the medications will be used, but after medication start, the participants' data will not be used and they will be censored at Week 24 (i.e. Day 172). For other participants, all available data up to Week 24 (i.e. Day 172) including those collected during the off-treatment period will be used. Participants without events will be censored at Day 172 or their last WI-NRS assessment date if discontinued from the study, whichever is earlier.

Secondary efficacy endpoints that measure binary responses

Secondary efficacy endpoints that measure binary responses will be analyzed in the same fashion as the primary endpoint using primary statistical model.

Continuous secondary efficacy endpoints

The primary estimand for continuous secondary efficacy endpoints is defined in Table 3 and will be analyzed using an analysis of covariance (ANCOVA) model with intervention group, documented history of atopy (atopic or non-atopic), stable use of TCS/TCI (yes or no), region (countries combined), baseline antidepressant use (yes or no), and relevant baseline measurement as covariate, with intercurrent event strategy and missing data handling as defined as in Table 3. Specifically, data of participants taking the prohibited medications/procedures and/or rescue medications will be set to missing after the medication usage, and the worst postbaseline value on

or before the time of the medication usage will be used to impute missing endpoint value (for participants whose postbaseline values are all missing, the baseline will be used to impute). Participants who discontinue the treatment prematurely are encouraged to follow the planned clinical visits and in those participants who did not take the prohibited medications/procedures and/or rescue medications, all data collected after treatment discontinuation will be used in the analysis. For these participants, missing data may still happen despite all efforts have been tried to collect the data after treatment discontinuation. For participants who discontinue due to lack of efficacy, all data collected after discontinuation will be used in the analysis, and a WOCF approach will be used to impute missing data if needed. For participants who discontinued not due to lack of efficacy, a multiple imputation (MI) approach will be used to impute missing endpoint value, and this MI method will use all participants excluding participants who have taken the prohibited medications/procedures and/or rescue medications prior to timepoint of endpoint of interest and excluding participants who discontinue due to lack of efficacy.

Each of the imputed complete data will be analyzed by fitting an ANCOVA model as described above. The imputation number will be about 40. Statistical inference obtained from all imputed data will be combined using Rubin's rule. Descriptive statistics including number of participants, mean, standard error, and least squares (LS) mean changes (and standard error) will be provided. In addition, difference of the dupilumab group against placebo in LS means and the corresponding 95% confidence intervals (CI) will be provided along with the p-values.

See Section 5.5 for the sample SAS code for the imputation and the analysis.

For the endpoint of percent change from baseline in WI-NRS at week 24, we will also perform an as-observed plus multiple imputation supplementary analysis as described below.

The data collected after taking all prohibited medications and/or rescue medications will be included in the supplementary analysis to evaluate the robustness of the primary analysis results with respect to the method of handling data while taking the prohibited medications (e.g. treatment policy strategy). In addition, for participants discontinuing the study treatment before Week 24, their off-study treatment values measured up to Week 24 will be included in the analysis. For participants having missing data at Week 24 regardless of reason(s), a multiple imputation (MI) approach will be used to impute the missing endpoint value.

4.5 TERTIARY/EXPLORATORY ENDPOINT(S) ANALYSIS

4.5.1 Definition of endpoint(s)

The exploratory endpoints are as follows:

- Use of high potency or superpotent TCS rescue medication through Week 24
- Use of systemic immunosuppressant through Week 24, constituting treatment failure
- Change from baseline in Hospital Anxiety and Depression Scale (HADS) total score to Week 24
- Change from baseline in EQ-5D-5L Single Index score to Week 24

- Change from baseline in EQ-5D visual analog scale (VAS) to Week 24
- Change from baseline in skin Pain-NRS to Week 4, Week 8, Week 12, and Week 24, respectively
- Change from baseline in Sleep-NRS to Week 4, Week 8, Week 12, and Week 24, respectively
- Missed school/work days through Week 24
- Incidence of skin-infection TEAEs (excluding herpetic infections) through Week 24
- Proportion of participants who achieve ≥75% healed lesions from PAS at Week 4, Week 8, Week 12, and Week 24, respectively
- Change from baseline in exact number of lesions in representative area (as determined from PAS) at Week 4, Week 8, Week 12, and Week 24, respectively
- Change from baseline in Participant Global Impression of Severity (PGIS) of PN to Week 4, Week 8, Week 12, and Week 24 respectively
- Proportion of participants with PGIS score of "none" at Week 4, Week 8, Week 12, and Week 24 respectively
- Proportion of participants with PGIS score of "none" or "mild" at Week 4, Week 8, Week 12, and Week 24 respectively
- Participant Global Impression of Change (PGIC) of PN at Week 4, Week 8, Week 12, and Week 24, respectively
- Proportion of participants with PGIC score of "very much better" at Week 4, Week 8, Week 12, and Week 24 respectively
- Proportion of participants with PGIC score of "very much better" or "moderately better" at Week 4, Week 8, Week 12, and Week 24 respectively

Hospital anxiety and depression scale (HADS)

The HADS is a PRO instrument for screening anxiety and depression in non-psychiatric populations; repeated administration also provides information about changes to a participant's emotional state. The HADS consists of 14 items, 7 each for anxiety and depression symptoms; possible scores range from 0 to 21 for each subscale. The following cut-off scores are recommended for both subscales:

- 0 to 7: normal
- 8 to 10: borderline abnormal (borderline case)
- 11 to 21: abnormal

Euroqol 5 dimensions questionnaire (EQ-5D)

The Euroqol-5 dimensions (EQ-5D) is a standardized PRO measure of health status developed by the EuroQOL Group in order to provide a simple, generic measure of health for clinical and

economic appraisal. The EQ-5D consists of 2 parts: the descriptive system and the EQ visual analog scale (VAS). The EQ-5D descriptive system comprises the following 5 dimensions: mobility, self-care, usual activities, pain/discomfort, and anxiety/depression. Each dimension has 5 levels of perceived problems: “no problem”, “slight problems”, “moderate problems”, “severe problems” and “inability to do the activity”. The respondent is asked to indicate his/her health state by ticking (or placing a cross) in the box against the most appropriate statement in each of the 5 dimensions; this results in a 1-digit number expressing the level for that dimension. The digits for 5 dimensions can be combined in a 5-digit number describing the respondent’s health state. The EQ VAS records the respondent’s self-rated health on a vertical VAS where the endpoints are labeled “best imaginable health state (100)” and “worst imaginable health state (0)”. This information can be used as a quantitative measure of health outcome as judged by the individual respondents.

Skin Pain and sleep numeric rating scales (skin Pain-NRS, Sleep-NRS)

Participants will be asked to rate their worst skin pain in the past 24 hours using a 0 to 10 NRS, with 0 = No pain to 10 = Worst pain possible.

In addition, participants will be asked to rate their sleep quality on their past night upon awakening, using a 0 to 10 NRS, with 0 = Worst possible sleep and 10 = Best possible sleep.

Participants will complete the skin pain NRS and sleep quality NRS once a day.

Prurigo activity score (PAS)

The PAS is a clinician-reported outcome (ClinRO) measurement. The original PAS questionnaire Version 0.9 consists of 7 items, developed by expert clinicians in PN.

A 5-item simplified version of the PAS will be used in the current study. Item 4 (exact number of pruriginous lesions in representative area) and Item 5b (percentage of healed prurigo lesions in all pruriginous lesions) will be used for analyses. In addition, Item 2 (number of prurigo lesions) may be used for analysis too.

Participant Global Impression of Change of disease (PGIC) and Participant Global Impression of Severity (PGIS)

The PGIC is a one-item questionnaire that asks participants to provide the overall self-assessment of change in their PN overall on a 7-point scale, compared to just before participant started taking the study injection. Response choices are: 0 = “Very much better”, 1 = “Moderately better”, 2 = “A little better”, 3 = “No change”, 4 = “A little worse”, 5 = “Moderately worse”, 6 = “Very much worse”.

The PGIS is a one-item questionnaire that asks participants to provide the overall self-assessment of their disease severity on a 4-point scale for the past week. Response choices are: 1 = “none”, 2 = “Mild”, 3 = “Moderate”, 4 = “Severe”.

Missed school/work days

Participants who are employed or enrolled in school will be asked to report the number of sick leave/missed school days since the last study assessment.

4.5.2 Main analytical approach

Exploratory efficacy endpoints will be analyzed using the same methodology as secondary efficacy for similar data (continuous or proportion) except the endpoints below.

- Use of high potency or superpotent TCS rescue medication through Week 24
- Use of systemic immunosuppressant through Week 24, constituting treatment failure
- Missed school/work days through Week 24
- Incidence of skin-infection TEAEs (excluding herpetic infections) through Week 24

The number of participants and percentage with the events for these endpoints will be provided in observed cases. In addition, the time (week) to first event for those participants will also be analyzed using Kaplan-Meier method and the duration of the rescue medication use may be summarized if applicable.

An additional exploratory endpoint of time to first select prohibited/rescue medication that impact efficacy will be provided. This includes medications where WOCF will be applied (see Table 5).

4.6 MULTIPLICITY ISSUES

The multiplicity procedure is proposed to control the overall type-I error rate for testing the primary and selected secondary endpoints. The overall alpha is 0.05. The comparisons with placebo will be tested based on the hierarchical order below at 2-sided $\alpha = 0.05$:

In US and US reference countries:

- Proportion of participants with improvement (reduction) in WI-NRS by ≥4 from baseline to Week 12.
- Proportion of participants with improvement (reduction) in WI-NRS by ≥4 from baseline to Week 24
- Proportion of participants with IGA PN-S 0 or 1 score at Week 24
- Proportion of participants with both an improvement (reduction) in WI-NRS by ≥4 from baseline to Week 24 and an IGA PN-S 0 or 1 score at Week 24
- Proportion of participants with IGA PN-S 0 or 1 score at Week 12
- Percent change from baseline in WI-NRS at Week 24
- Change from baseline in HRQoL, as measured by Dermatology Life Quality Index (DLQI) to Week 24
- Change from baseline in skin Pain-NRS to Week 24

- Change from baseline in Sleep-NRS to Week 24
- Change from baseline in Hospital Anxiety and Depression Scale (HADS) total score to Week 24

In all other countries (including EU and EU reference countries, as well as China and Japan):

- Proportion of participants with improvement (reduction) in WI-NRS by ≥4 from baseline to Week 12.
- Proportion of participants with improvement (reduction) in WI-NRS by ≥4 from baseline to Week 24
- Proportion of participants with IGA PN-S 0 or 1 score at Week 24
- Percent change from baseline in WI-NRS at Week 24
- Proportion of participants with IGA PN-S 0 or 1 score at Week 12
- Change from baseline in HRQoL, as measured by Dermatology Life Quality Index (DLQI) to Week 24
- Change from baseline in skin Pain-NRS to Week 24
- Change from baseline in Sleep-NRS to Week 24
- Change from baseline in Hospital Anxiety and Depression Scale (HADS) total score to Week 24

The study is considered positive when the primary endpoint achieves statistical significance.

4.7 SAFETY ANALYSES

All safety analyses will be performed on the safety population as defined in Section 3, unless otherwise specified, using the following common rules:

- The analysis of the safety variables will be descriptive, and no testing is planned.
- Safety data in participants who do not belong to the safety population (e.g., exposed but not randomized) will be provided separately.

4.7.1 Extent of exposure

The extent of IMP exposure will be assessed by the duration of IMP exposure and compliance and summarized within the safety population.

Duration of IMP exposure

Duration of IMP exposure is defined as last dose date – first dose date + 14 days, regardless of unplanned intermittent discontinuations. Duration of IMP exposure will be summarized descriptively as a quantitative variable (number, mean, SD, median, minimum, and maximum). In addition, duration of treatment exposure will also be summarized categorically by numbers and percentages for each of the following categories and cumulatively according to these categories:

- >0 and ≤2 weeks
- >2 and ≤4 weeks
- >4 and ≤8 weeks
- >8 and ≤12 weeks
- >12 and ≤16 weeks
- >16 and ≤20 weeks
- >20 and ≤24 weeks
- >24 weeks and ≤ 24 weeks + 3 days
- > 24 weeks + 3 days

Additionally, the cumulative duration of treatment exposure will be provided, defined as the sum of the duration of treatment exposure for all participants, and will be expressed in participant years.

Treatment compliance

A given administration will be considered noncompliant if the participant did not take the planned dose as required by the protocol. No imputation will be made for participants with missing or incomplete data.

Percentage of treatment compliance for a participant will be defined as the number of administrations that the participant was compliant divided by the total number of administrations that the participant was planned to take from the first administration of IMP up to the actual last administration of IMP.

Treatment compliance will be summarized quantitatively and categorically: <80%, ≥80%.

Cases of overdose (defined as at least twice the intended dose during an interval of less than 11 days) will be considered an AESI per dupilumab clinical programs and will be listed as such.

4.7.2 Adverse events

General common rules for adverse events

All adverse events (AEs) will be coded to a lower-level term (LLT), preferred term (PT), high-level term (HLT), high-level group term (HLGT), and associated primary system organ class (SOC) using the Medical Dictionary for Regulatory Activities (MedDRA) version currently in effect at Sanofi at the time of database lock.

The AEs will be analyzed in the following 3 categories:

- Pre-treatment AEs: AEs that developed, worsened or became serious during the pre-treatment period.

- Treatment-emergent adverse events (TEAE)s: AEs that developed, worsened or became serious during the treatment-emergent period
- Post-treatment AEs: AEs that developed, worsened or became serious during the post-treatment period

Similarly, the deaths will be analyzed in the pre-treatment, treatment-emergent and post-treatment periods.

The primary focus of AE reporting will be on TEAEs. Pre-treatment and post-treatment AEs will be described separately.

An AE with incomplete or missing date/time of onset (occurrence, worsening, or becoming serious) will be classified as a TEAE unless there is definitive information to determine it is a pre-treatment or a post-treatment AE.

If the assessment of the relationship to IMP is missing for an AE, this AE will be assumed as related to IMP. If the severity is missing for 1 of the treatment-emergent occurrences of an AE, the severity will be imputed with the maximal severity of the other occurrences. If the severity is missing for all the occurrences, the severity will be left as missing.

Multiple occurrences of the same event in the same participant will be counted only once in the tables within a treatment phase.

The AE tables will be sorted as indicated in Table 6.

Table 6 - Sorting of AE tables

AE presentation	Sorting rules
SOC, HLGT, HLT and PT	By the internationally agreed SOC order and by alphabetic order of HLGTs, HLTs and PTs.
SOC, HLT and PT	By the internationally agreed SOC order and by alphabetic order of HLTs and PTs.
SOC and PT	By the internationally agreed SOC order and decreasing frequency of PTs[a, b]
SMQ/CMQ and PT	By decreasing frequency of SMQs/CMQs and PTs[a]
PT	By decreasing frequency of PTs[a]

a Sorting will be based on the SAR231893 dupilumab group

b The table of all TEAEs presented by SOC and PT will define the presentation order for all other tables (eg, treatment-emergent SAE) presented by SOC and PT, unless otherwise specified.

Analysis of all adverse events

The overview of TEAE with the details below will be generated:

- Any TEAE
- Any severe TEAE
- Any treatment emergent SAE
- TEAE leading to death

- Any TEAE leading to permanent intervention discontinuation
- Any treatment emergent AESI
- Any treatment emergent other AE of interest grouping
- Any TEAE related to IMP

The AE summaries of Table 7 will be generated with number (%) of participants experiencing at least one event.

Table 7 - Analyses of adverse events

Type of AE	MedDRA levels
All TEAE	Primary SOC, HLGT, HLT and PT
	Primary SOC and PT
	PT
	Primary and secondary SOC, HLGT, HLT and PT
Common TEAE (≥2% and 5% in any group)	Primary SOC and PT
TEAE related to IMP as per Investigator's judgment	Primary SOC, HLGT, HLT and PT
	Primary SOC and PT
TEAE by maximal intensity	Primary SOC and PT
Treatment emergent SAE	Primary SOC, HLGT, HLT and PT
	Primary SOC and PT
Treatment emergent SAE related to IMP as per Investigator's judgment	Primary SOC, HLGT, HLT and PT
TEAE leading to permanent intervention discontinuation	Primary SOC, HLGT, HLT and PT
	Primary SOC and PT
TEAE leading to death (death as an outcome of the AE as reported by the Investigator in the AE page)	Primary SOC, HLGT, HLT and PT
Pretreatment AE	Overview[a]
	Primary SOC and PT

a Will include the following AE categories: any AEs, any serious AEs, any AEs leading to death, any AEs leading to permanent intervention discontinuation

<u>Analysis of deaths</u>

In addition to the analyses of deaths included in Table 6 the number (%) of participants in the following categories will be provided:

- Deaths during the treatment-emergent and post-treatment periods
- Deaths in non-randomized or randomized but not treated participants

Analysis of adverse events of special interest (AESIs) and other AEs of interest

Adverse events of special interest (AESIs) and other AEs of interest will be selected for analyses as indicated in Table 8. Number (%) of participants experiencing at least one event will be provided for each event of interest. Tables will be sorted as indicated in Table 6.

Table 8 - Selections for AESIs and other AEs of interest

AE Grouping	Criteria
**AESI**	
Anaphylactic reaction	Anaphylactic reaction algorithmic approach (Introductory Guide for Standardised MedDRA Queries (SMQs) Version 18.1): includes anaphylactic reaction narrow SMQ (20000021) terms and programmatic identification of cases based on occurrence of at least two preferred terms meeting the algorithm criteria occurring within 24 hours of each other. The latter cases identified using the algorithm will undergo blinded medical review taking into account the timing of events relative to each other and to IMP administration for final determination of an anaphylactic reaction or not.
Systemic hypersensitivity reactions	SMQ [20000214] hypersensitivity narrow search and [AE corrective treatment/therapy='Y' or Action taken with IMP='Drug withdrawn' or Action taken with IMP='Drug interrupted'] followed by blinded medical review (documented process) for selection of relevant systemic hypersensitivity events
Helminthic infections	CMQ10544 based on HLGT as "Helminthic disorder"
Any severe type of conjunctivitis	CMQ10498 based on PTs (See Section 5.6)[a] and "Severe" ticked in Adverse Events eCRF page
Any severe type of blepharitis	CMQ10497 based on HLT as "Lid, lash and lacrimal infections, irritations and inflammations" and "Severe" ticked in Adverse Events eCRF page
Keratitis	CMQ10642 based on the following PTs [keratitis, allergic keratitis, ulcerative keratitis, atopic keratoconjunctivitis, herpes ophthalmic, ophthalmic herpes simplex, corneal infection][a]
Clinically symptomatic eosinophilia (or eosinophilia associated with clinical symptoms)[b]	CMQ10641 based on HLT = Eosinophilic disorders or PT=Eosinophil count increased
Pregnancy of a female participants entered in a study as well as pregnancy occurring in a female partner of a male participant entered in a study with IMP/NIMP	"Pregnancy" or "Partner Pregnancy" checked on the Pregnancy eCRF page as reported by the investigator
Significant ALT elevation	"ALT increase" and AESI answer "Yes" checked on AE eCRF as reported by the investigator (ALT >5 x ULN in participants with baseline ALT ≤2 x ULN; OR ALT >8 x ULN if baseline ALT >2 x ULN)
Symptomatic overdose with IMP	Symptomatic Overdose is answered Yes, with Overdose of IMP answered Yes on AE eCRF.

AE Grouping	Criteria
Symptomatic overdose with NIMP	Symptomatic Overdose is answered Yes, with Overdose of NIMP answered Yes on AE eCRF.
**Other selected AE Grouping**	
Serious injection site reactions or severe injection site reactions that last longer than 24 hours	HLT = 'Injection site reaction' and either with serious status, or with severe status and (AE end date/time - AE start date/time) ≥ 24 hours or ongoing
Severe or serious infection	Primary SOC = 'Infections and infestations' and with severe or serious status
Drug-related hepatic disorder	SMQ [20000006] Drug-related hepatic disorders- narrow
Injection site reaction	HLT = 'Injection site reaction'
Malignancy	SMQ [20000091]- Malignant or unspecified tumors narrow
Suicidal behavior	CMQ10639 based on the following PTs [Completed suicide, Suicidal ideation, Depression suicidal, Suicidal behavior, Suicide attempt][a]
Conjunctivitis (narrow)	CMQ10644 based on the following PTs [Conjunctivitis, Conjunctivitis allergic, Conjunctivitis bacterial, Conjunctivitis viral, Atopic keratoconjunctivitis][a]
Conjunctivitis (broad)	CMQ10645 based on the following PTs [Conjunctivitis, Conjunctivitis allergic, Conjunctivitis bacterial, Conjunctivitis viral, Atopic keratoconjunctivitis, Blepharitis, Dry eye, Eye irritation, Eye pruritus, Lacrimation increased, Eye discharge, Foreign body sensation in eyes, Photophobia, Xerophthalmia, Ocular hyperaemia, Conjunctival hyperaemia][a]
Conjunctivitis (FDA)[c]	CMQ10643 based on the following PTs [Conjunctivitis, Conjunctivitis allergic, Conjunctivitis bacterial, Conjunctivitis viral, Eye irritation, Eye inflammation, Giant papillary conjunctivitis][a]
Keratitis (FDA)[c]	CMQ30102 based on the following PTs in (keratitis, ulcerative keratitis, allergic keratitis, atopic keratoconjunctivitis, and ophthalmic herpes simplex)

a The list of terms may be adjusted according to MedDRA version changes
b All cases of Eosinophilia will be included in the analysis, where cases associated with clinical symptoms will be further described in the CSR
c FDA requested for US labeling

The following summaries will be provided:

- All TEAEs, by selected standardized MedDRA query (SMQ)/Customized MedDRA query (CMQ) and PT or by laboratory values (as in alanine aminotransferase (ALT) elevation), showing the number (%) of participants with at least 1 PT,
- For each AESI and other selected AE groupings,
  - Number (%) of participants with any specific TEAE

  - Number (%) of participants with any specific serious AE (regardless of treatment emergent status)
  - Number (%) of participants with any specific treatment emergent serious AE
  - Number (%) of participants with any specific AE leading to death
  - Number (%) of participants with any specific TEAE leading to permanent study drug discontinuation
  - Number (%) of participants with any specific TEAE related to IMP reported by investigator
  - Number (%) of participants with any specific TEAE by maximum intensity, corrective treatment, and final outcome
  - Number of any specific TEAE adjusted by the exposure duration
  - Number of participants with any specific TEAE adjusted by the exposure duration at risk. For each specific TEAE, Kaplan-Meier estimates of cumulative incidence at Week 12, and 24 and K-M plot may be provided to depict the course of onset over time if the number of events is large enough.
  - Number (%) of participants with injection site reactions by IMP injection.
  - Number (%) of participants with different number of injection site reactions.
- In addition, AESIs reported by the investigator in eCRF will be summarized separately.

In addition, the exposure-adjusted adverse event incidence rate tables will provide the number of patients with at least 1 event per 100 patient-years for the summaries of TEAEs, treatment emergent SAE, TEAE leading to permanent intervention discontinuation, and AESIs and other selected AE groups in Table 8

4.7.3 Additional safety assessments

4.7.3.1 Laboratory variables, vital signs and electrocardiograms (ECGs)

The following laboratory variables, vital signs and electrocardiogram (ECG) variables will be analyzed. They will be converted into standard international units.

- Hematology:
  - Red blood cells and platelets and coagulation: hemoglobin, hematocrit, red blood cell count, platelet count
  - White blood cells: white blood cell count, neutrophils, lymphocytes, monocytes, basophils, eosinophils
- Clinical chemistry:
  - Metabolism: glucose, total cholesterol, total protein, creatine phosphokinase
  - Electrolytes: sodium, potassium, chloride, bicarbonate

- Renal function: creatinine, blood urea nitrogen, uric acid
- Liver function: alanine aminotransferase, aspartate aminotransferase, alkaline phosphatase, lactate dehydrogenase, total bilirubin, albumin
- Pregnancy test: Serum β-human chorionic gonadotropin (all female participants) will be performed at screening (V1) in women of childbearing potential, and a urine pregnancy test will be performed at V2 and every 4 weeks thereafter.
- Hepatitis screen: hepatitis B surface antigen (HBs Ag), hepatitis B surface antibody (HBs Ab), hepatitis B core antibody (HBc Ab), hepatitis C virus antibodies (HCV Ab) will be tested at screening (V1). In case of results showing HBs Ag (negative) and HBc Ab (positive), an hepatitis B virus (HBV) deoxyribonucleic acid (DNA) testing will be performed and should be confirmed negative prior to randomization. In case of results showing HCV Ab (positive), an HCV ribonucleic acid (RNA) testing will be performed and should be confirmed negative prior to randomization.
- HIV screen: Anti-HIV-1 and HIV-2 antibodies will be tested at Visit 1.
- TB test (performed locally if required and results noted in the eCRF).

- Urinalysis:
  - Urinalysis will include specific gravity, pH, glucose, ketones, blood, protein, nitrate, leukocyte esterase, urobilinogen and bilirubin. If positive for protein and/or red blood cells, microscopic analysis will be performed by the central laboratory. Creatinine, leukotriene and tetranor PGDM will be tested by the central laboratory.
- Vital signs: pulse rate (beats per minute), systolic and diastolic blood pressure (mmHg) in a semi-supine or sitting position after 5 minutes, weight, respiratory rate (breaths per minute), temperature (degrees Celsius) and height (screening only).
- ECG variables: heart rate, PR, QRS, QT, and QTcF intervals after 10 minutes of rest in the supine position. Data are locally collected and read at screening (V1) and Week 24 (V6).

Data below the lower limit of quantitation/detection limit (LLOQ) will be replaced by half of the LLOQ, data above the upper limit of quantification will be replaced by ULOQ value.

Quantitative analyses

For all laboratory variables, vital signs and ECG variables above, descriptive statistics for results and changes from baseline will be provided for each analysis window, the last value and the worst value during the on-treatment period. These analyses will be performed using central measurements only (when available) for laboratory variables.

For all parameters, mean changes from baseline with the corresponding standard error will be plotted over time.

Analyses according to PCSA

Analysis of potentially clinically significant abnormality (PCSA) will be performed based on the PCSA list in effect at Sanofi at the time of the database lock. For parameters for which no PCSA criteria are defined, similar analyses will be done using the normal range, if applicable.

Analyses according to PCSA will be performed based on the worst value during the treatment-emergent period, using all measurements (either local or central, either scheduled, nonscheduled or repeated).

For laboratory variables, vital signs and ECG variables above, the incidence of participants with at least one PCSA during the treatment-emergent period will be summarized regardless of the baseline level and according to the following baseline status categories:

- Normal/missing
- Abnormal according to PCSA criterion or criteria

Additional analyses for suspect drug-induced liver injury

The following additional analyses will be performed for drug-induced liver injury:

- Time to onset of the initial ALT or aspartate aminotransferase (AST) elevation (>3 x ULN) and total bilirubin elevation (>2 x ULN) during the treatment-emergent period will be analyzed using Kaplan-Meier method.
- A graph of the distribution of peak values of ALT versus peak values of total bilirubin during the treatment-emergent period will be provided.
- For each liver function test (eg, ALT), participants having a PCSA (eg, ALT >5 ULN) will be summarized using the following categories: Returned to baseline PCSA status (or returned to value ≤ ULN in case of missing baseline) before last IMP dose, Returned to baseline PCSA status after last IMP dose, Never returned to baseline PCSA status, No assessment after elevation. This summary will be performed by categories of elevation (ALT >3, >5, >10, >20 ULN).

4.8 OTHER ANALYSES

4.8.1 Pharmacokinetic analyses

Predose dupilumab concentrations in serum at Visit 2 (Day 1), dupilumab trough concentrations at Week 4, Week 8, Week 12, Week 24/EOT Visit and post-treatment dupilumab concentrations at Week 36/EOS Visit will be provided.

Concentrations of dupilumab in serum will be summarized in the PK population using arithmetic and geometric means, SD, standard error of the mean (SEM), coefficient of variation (CV), minimum, median and maximum per sampling time. If date and/or time of the drug injection and/or sampling is missing then the concentration will not be taken into account. For drug-treated participants, where concentration values are below the lower limit of quantification (LLOQ),

one-half of the LLOQ will be used. Values will be expressed in the tables with no more than three significant figures. For participants in the placebo group, concentration values that are below the LLOQ will be taken into account with a plasma concentration considered equal to 0.

4.8.2 Immunogenicity analyses

Anti-drug antibody (ADA) status to dupilumab (negative or titer value, if positive in the ADA assay) at Visit 2 (Day 1), Week 12, Week 24/EOT and follow up at Week 36 will be provided. The neutralizing antibody status for ADA positive samples will be provided.

Incidence will be provided for the following ADA response categories:

Pre-existing immunoreactivity is defined as:

An ADA positive response in the assay at baseline with all post first dose ADA results negative, OR an ADA positive response at baseline with all post first dose ADA responses less than 4-fold over baseline titer levels.

Treatment-emergent ADA response is defined as:

A positive response in the ADA assay post first dose, when baseline results are negative or missing.

Treatment-emergent ADA responses are further classified as Persistent, Indeterminate or Transient

a) Persistent Response- defined as a treatment-emergent ADA response with two or more consecutive ADA positive sampling time points, separated by greater than (>) 12-week period (84 days), with no ADA negative samples in between.
b) Indeterminate Response- defined as a treatment-emergent response with only the last collected sample positive in the ADA assay
c) Transient Response - defined as a treatment-emergent response that is not considered persistent OR indeterminate

Treatment-boosted response is defined as:

An ADA positive response in the assay post first dose that is greater-than or equal to 4-fold over baseline titer levels, when baseline results are positive.

Titer values (Titer value category)

- Low (Titer <1000)
- Moderate (1,000 ≤ Titer ≤10,000)
- High (Titer >10,000)

The following summary will be provided based on ADA population:

- Number (%) of participants with pre-existing immunoreactivity
- Number (%) of participants with treatment-emergent ADA
- The summary statistics (including number, median, Q1, Q3, minimum and maximum) of the peak post-baseline titer for participants with treatment-emergent ADA, and participants with persistent, indeterminate and transient ADA response
- Number (%) of participant with transient treatment-emergent ADA
- Number (%) of participants with persistent treatment-emergent ADA
- Number (%) of participants with indeterminate treatment-emergent ADA
- Number (%) of participants with treatment-boosted ADA
- The summary statistics (including number, median, Q1, Q3, minimum and maximum) of the peak post-baseline titer for participants with treatment-boosted ADA
- The summary statistics (including number, mean, SD, median, Q1, Q3, minimum and maximum) of the ratio of peak post-baseline titer to baseline titer for participants with treatment-boosted ADA
- Listing of ADA peak titer levels and neutralizing antibody status
- Number (%) of participants with neutralizing antibody status

Kinetics of treatment-emergent ADA response

Number (%) of participants with treatment-emergent ADA positive response at each visit will be summarized, including titer categories (lower, moderate, and high titer), by each intervention group.

A plot of percentage of participants with treatment-emergent ADA positive response at each visit will be provided by each intervention group.

Association of Immunogenicity with Exposure, Safety and Efficacy

The safety and efficacy analyses mentioned below will be conducted using the following categories:

- ADA positive participants: Participants with treatment-emergent or treatment-boosted response.
- ADA negative participants: Participants with pre-existing immunoreactivity or negative in the ADA assay at all time points.

Association of ADA with PK

Associations between ADA variables (eg, ADA peak titers, neutralizing antibody status, treatment-emergent, persistent, indeterminate and transient response, treatment-boosted) and concentration of dupilumab in serum may be explored for each dupilumab dose group. A plot of

concentration of functional dupilumab versus visit will be provided by ADA classifications for the dupilumab dose group. Individual participant plots of dupilumab concentration according to ADA status will be provided to assess the impact of ADA on PK.

Association of ADA with clinical efficacy endpoints

Associations between the ADA variables (eg, ADA peak titers, neutralizing antibody status, treatment-emergent, persistent and treatment-boosted) and the primary efficacy endpoint may be explored for the dupilumab dosed group.

Association of ADA with clinical safety endpoints

Association of safety versus ADA status may be analyzed in the ADA population. The safety assessment may focus on the following events:

- Severe injection site reactions last longer than 24 hours or serious injection site reactions
- Hypersensitivity reactions (SMQ (20000214) hypersensitivity narrow search confirmed by medical review)
- Anaphylactic reactions (SMQ (20000021) anaphylactic reaction narrow search)

Associations between ADA variables (eg, ADA peak titers, neutralizing antibody status, treatment-emergent, persistent and treatment-boosted) and safety may be explored.

4.8.3 Pharmacodynamic/genomics endpoints

Venous blood samples will be collected at Visit 2 (Week 0), Visit 3 (Week 4), Visit 4 (Week 8), Visit 5 (Week 12), Visit 6 (Week 24), and Visit 7 (Week 36), for measurement of total serum IgE. Total IgE will be measured using validated quantitative methods.

For those participants who consent to the optional pharmacogenetic/pharmacogenomic sample collection section of the ICF, blood (serum/plasma) for possible future analysis of potential biomarkers of drug response, disease activity, safety, and the type 2 inflammation pathway, and blood samples for exploratory genetic analysis of DNA or RNA will be collected and stored for possible future use. Participation is optional. Participants who do not wish to participate in the genetic research may still participate in the study.

Total IgE will be summarized in the safety population defined as participants who actually received at least 1 dose or part of a dose of the IMP. Baseline values will be the last value collected prior to the first IMP. Descriptive statistics (including number, mean, SD, median, Q1, Q3, min, max) of biomarkers at baseline will be summarized. In addition, for participants who take the prohibited medications/procedures or rescue medications defined in Table 5, the data after the medications will be censored and then last observation carried forward (LOCF) method will be implemented for missing data imputation in the analysis..

Summary plots (median with interquartile range) on values at each visit, absolute changes from baseline and percent changes from baseline will be provided for the total IgE by intervention group and visit.

Exploratory analysis of DNA, RNA and urine biomarkers will be addressed in a separate document.

4.9 INTERIM ANALYSES

No interim analysis is planned.

A primary database lock will be performed when all randomized participants in this study have completed their 24-week treatment phase. Final analyses in the CSR will be based on this database.

The database will be updated at the end of the study for all participants to include the post-treatment follow-up information and updates for the events previously ongoing at the time of the primary lock. Additional data between this database lock and last participant completing last visit will be summarized in a CSR addendum.

5 SUPPORTING DOCUMENTATION

5.1 APPENDIX 1 LIST OF ABBREVIATIONS

ADA:	anti-drug antibody
AE:	adverse event
AESIs:	adverse events of special interest
ALT:	alanine aminotransferase
ANCOVA:	analysis of covariance
AST:	aspartate aminotransferase
ATC:	anatomic category
CI:	confidence interval
CLcr:	creatinine clearance
CMH:	Cochran-Mantel-Haenszel
CMQ:	customized MedDRA query
CSR:	clinical study report
DLQI:	Dermatology Life Quality Index
DNA:	deoxyribonucleic acid
ECG:	electrocardiogram
eCRF:	electronic case report form
EMA:	European Medicines Agency
EOT:	end of treatment
EQ-5D-5L:	5-level EuroQol 5-dimensional questionnaire
FDA:	Food and Drug Administration
HADS:	hospital anxiety and depression scale
HBc Ab:	hepatitis B core antibody
HBs Ab:	hepatitis B surface antibody
HBs Ag:	hepatitis B surface antigen
HBV:	hepatitis B virus
HCV Ab:	hepatitis C virus antibodies
HLGT:	high level group term
HLT:	high level term
HRQoL:	health-related quality-of-life
IGA PN:	Investigator's global assessment for prurigo nodularis
IMP:	investigational medicinal product
ITT:	intent-to-treat
LLT:	lower-level term
LS:	least squares
MedDRA:	Medical Dictionary for Regulatory Activities
PCSA:	potentially clinically significant abnormality
PGIC:	Participant Global Impression of Change
PGIS:	Global Impression of Severity
PK:	pharmacokinetic
PN:	prurigo nodularis

PT:	preferred term
RNA:	ribonucleic acid
SAE:	serious adverse event
SAP:	statistical analysis plan
SD:	standard deviation
SDG:	standardized drug grouping
SMQ:	standardized MedDRA query
SOC:	system organ class
TEAE:	treatment-emergent adverse event
ULN:	upper limit of normal
WHO-DD:	World Health Organization-Drug Dictionary
WI-NRS:	worst-itch numeric rating scale
WOCF:	worst-observation carried forward

5.2 APPENDIX 2 CHANGES TO PROTOCOL-PLANNED ANALYSES

This section summarizes major statistical changes in the protocol amendment.

Table 9 - Major statistical changes in protocol amendment(s)

Amendment Number	Approval Date	Changes	Rationale
1	20-May-2020	To add "proportion of participants with Investigator's Global Assessment 0 or 1 score for PN-Stage (IGA PN-S) at Week 24" as another key secondary endpoint	To include a lesion-related key secondary endpoint according to the health authority's recommendations
		To remove the endpoint "Change from baseline in PAS total score at Week 4, Week 8, Week 12, and Week 24" in the exploratory endpoint and modify the exploratory endpoint regarding the healed lesions from PAS questionnaire analysis	To clarify the analysis on efficacy evaluation of dupilumab on skin lesions using a modified prurigo activity score (PAS) 5-item questionnaire
		To break out the secondary endpoints with multiple measuring timepoints into individual endpoints.	To clearly define the timepoints of each endpoint according to health authority's recommendation
		To add the sensitivity analysis for secondary endpoints information, and to separate key secondary endpoints in a different row	To evaluate the robustness of the missing data imputation assumption by sensitivity analyses, and to clarify the endpoints analyses
		To add the covariate "baseline anti-depressant use (yes or no)" to primary and secondary endpoint analyses	To adjust for potential impact of anti-depressant use on the treatment effect in primary and secondary efficacy analyses
		The following wording was added: "Data collected regarding the impact of the COVID-19 or other pandemics, on the participants will be summarized (eg, discontinuation due to COVID-19). Any additional analyses and methods required to investigate the impact of COVID-19 or other pandemics requiring public health emergency on the efficacy (eg, missing data due to COVID-19) and safety will be detailed in the SAP".	To describe alternative temporary mechanism that can be implemented in the study conduct in case of pandemic requiring public health emergency eg, COVID-19

5.3 APPENDIX 3 DEMOGRAPHICS AND BASELINE CHARACTERISTICS, PRIOR OR CONCOMITANT MEDICATIONS/PROCEDURES

Demographics, baseline characteristics, medical surgical history

The following demographics and baseline characteristics, medical and surgical history and disease characteristics at baseline will be summarized using descriptive statistics in the randomized population.

Demographic variables are

- Age in years (quantitative and qualitative variable: 18-<40, 40–<65, 65-<75, and ≥75 years),
- Gender (Male, Female),
- Race (White, Black or African American, Asian, American Indian or Alaska Native, Native Hawaiian or other Pacific Island, unknown),
- Ethnicity (Hispanic or Latino, Not Hispanic or Latino, Unknown)
- Region (Asia: Taiwan, South Korea; East Europe: Hungary; Latin America: Chile; Western Countries: USA, Canada, France, Italy, Portugal, Spain, and UK)
- Territory (North America: USA, Canada; European Union: France, Italy, Portugal, Spain, Hungary, and UK; Rest of World: Taiwan, South Korea, Chile)
- Weight in kg (quantitative and qualitative variable: (<60, ≥60- <90, ≥90 kg)
- BMI in kg/m^2 (quantitative and qualitative variable: <25, ≥25- <30, ≥30 kg/m^2)

Baseline safety and efficacy parameters (apart from those listed above) will be presented along with the safety and efficacy summaries.

Medical (or surgical) history includes all the relevant medical (or surgical) history during the lifetime of the participant.

This information will be coded using the version of MedDRA currently in effect at Sanofi at the time of database lock.

Comorbidity will be summarized separately. The following comorbid diseases will be summarized from electronic case report form (eCRF) pages which were filled in by investigators based on participant reporting.

- Prurigo nodularis (Yes, Ongoing condition)
- Atopic dermatitis (Yes, Ongoing condition)
- Allergic rhinitis history (Yes, Ongoing condition)
- Allergic rhinoconjunctitvitis (Yes, Ongoing condition)
- Asthma history (Yes, Ongoing condition)

- Food allergy history (Yes, Ongoing condition)
- Eosinophilic esophagitis history (Yes, Ongoing condition)

Disease characteristics at baseline

The following baseline disease characteristics will be summarized by intervention group:

- Duration of PN and grouping (years; <3 and ≥3) to be derived as
  - (Year of randomization – Year of first diagnosis of PN) + (month of randomization – month of first diagnosis of PN)/12
- Age of onset of PN (years)
- History of atopy (atopic or non-atopic)
  - Number of mild AD participants under atopic
- Stable use of TCS/TCI (yes or no)
- Baseline WI-NRS score
- Baseline IGA PN-S score (summary and frequency by each score level)
- Baseline IGA PN-A score (summary and frequency by each score level)
- Baseline skin Pain-NRS score
- Baseline Sleep-NRS score
- Baseline PGIS score
- PAS (number of prurigo lesions in a total and in representative area, and healed prurigo lesions) at baseline
- Baseline Hospital Anxiety and Depression Scale (HADS) total score
  - Baseline Hospital Anxiety score
  - Baseline Depression Scale score
- Baseline Dermatology Life Quality Index (DLQI) score
- Baseline EuroQol five dimensions questionnaire (ED-5D-5L) (single index score and VAS)
- Frequency of alcohol drinking in the past 12 months (never, occasional, at least monthly, at least weekly, at least daily) and number of drinks on a typical day (1 or 2, >2)
- Antidepressant use (yes or no) at baseline
- HIV (positive versus negative)

Prior or concomitant medications and procedure

All medications will be coded using the World Health Organization-Drug Dictionary (WHO-DD) using the version currently in effect at Sanofi at the time of database lock.

All procedures will be coded to a PT and associated primary SOC using the version of MedDRA currently in effect at Sanofi at the time of database lock.

- Prior medications/procedures are those the participant used prior to first investigational medicinal product (IMP) injection. Prior medications/procedures can be discontinued before first administration or can be ongoing during treatment phase.
- Concomitant medications/procedures are any interventions received by the participant concomitantly to the IMP, from first administration of IMP to last IMP intake + 98 days.
- Post-treatment medications/procedures are those the participant took in the period running from the end of the concomitant medications period up to the end of the study.
- A given medication/procedure can be classified as a prior medication/procedure and/or as a concomitant medication/procedure and/or as post-treatment medication/procedure. If it cannot be determined whether a given medication/procedure was taken prior or concomitantly or post, it will be considered as prior, concomitant, and post-treatment medication/procedure.

The prior and concomitant medications/procedure will be summarized for the randomized population.

Medications will be summarized by intervention group according to the WHO-DD dictionary, considering the first digit of the anatomic category (ATC) class (anatomic category) and the first 3 digits of the ATC class (therapeutic category). All ATC codes corresponding to a medication will be summarized, and participants will be counted once in each ATC category (anatomic or therapeutic) linked to the medication. Therefore participants may be counted several time for the same medication.

The table for prior medications will be sorted by decreasing frequency of ATC followed by all other therapeutic classes based on the overall incidence across intervention groups. In case of equal frequency regarding ATCs (anatomic or therapeutic categories), alphabetical order will be used.

Concomitant medication received during first IMP to last IMP +14 days and concomitant medication received during first IMP to last IMP +98 days will be summarized separately. The tables for concomitant medications will be sorted by decreasing frequency of ATC followed by all other therapeutic classes based on the incidence in the dupilumab group. In case of equal frequency regarding ATCs (anatomic or therapeutic categories), alphabetical order will be used.

Medications will also be summarized by generic name sorted by decreasing frequency based on the incidence in the dupilumab group.

Procedures will be summarized by intervention group by primary SOC (sorted by internationally agreed order) and PT (sorted in alphabetical order), sorting is based on the overall incidence across intervention groups.

Background intervention

Participants will be required to apply moisturizers (emollients) once or twice daily for at least the 7 consecutive days immediately before baseline (Day 1) and continue until Week 36.

The compliance of moisturizers (emollients) used from 7 days before the baseline visit to Week 36 (or end of study), which is defined as the (number of days moisturizers used during the period) / (number of days within the period) x 100%, will be summarized by intervention group.

Similarly, the compliance of moisturizers (emollients) used from the baseline visit to Week 12 and Week 24 will be summarized by intervention group respectively.

In addition, the compliance of use of TCS/TCI from the baseline visit to Week 12 and Week 24 for participants with the stratification of stable use of TCS/TCI (yes) will be summarized by intervention group respectively.

.

Prohibited medications/procedures

The concomitant use of the following therapies is prohibited during the entire study. Study treatment will need to be discontinued in participants receiving these treatments:

- Systemic immunosuppressive/immunomodulating drugs (eg, systemic corticosteroids, cyclosporine, mycophenolate-mofetil, interferon gamma, Janus kinase inhibitors, azathioprine, methotrexate, hydroxychloroquine, dapsone, sulfasalazine, colchine, etc)
- Other monoclonal antibodies (which are biological modifiers)
- Phototherapy, including tanning beds
- Naltrexone or other opioid antagonist
- Gabapentin, pregabalin, and thalidomide

The concomitant use of the following therapies is prohibited except if the dose has been stable for at least 3 months prior to screening, but study treatment will not need to be discontinued in participants receiving the treatments listed below. The dose should also remain stable (can be reduced or discontinued if medically indicated) and should not be increased during the study.

- Paroxetine, fluvoxamine, or other SSRIs
- SNRIs
- Amitriptyline or other tricyclic or tetracyclic antidepressants

The concomitant use of the following therapies is also prohibited during the entire study, but study treatment will not need to be discontinued in participants receiving the treatments listed below:

- Intralesional corticosteroid injections and cryotherapy

- Sedating antihistamine
- Non-sedating antihistamine if used specifically for the treatment of itch secondary to AD or PN

The number and percentage of participants who take the prohibited medications/procedures will be provided. In addition, the time (week) of first prohibited medication/procedure taken will also be analyzed using Kaplan-Meier method.

Rescue medications

The following rescue medications may be used:

- Dermatological preparations of high potency or superpotent TCS and TCI.

If medically necessary (i.e., to control intolerable PN symptoms), rescue treatment for PN may be provided to study participants at the discretion of the Investigator.

Although the use of rescue medications is allowed at any time during the study, the use of rescue medications should be delayed, if possible, for at least 14 days following the initiation of the investigational treatment. The date and time of rescue medication administration as well as the name and dosage regimen of the rescue medication must be recorded in the eCRF.

- For the purpose of the efficacy responder analysis, a pre-specified algorithm will be used to classify rescue. In addition, a blinded review of all post-baseline medications to adjudicate rescue treatment, based on medical judgment, will be performed to adjudicate rescue. Participants who receive rescue treatment as per this adjudication during the study will be considered treatment failures.

The rescue medications will be analyzed by the same method as that used for prohibited medications/procedures.

5.4 APPENDIX 4 DATA HANDLING CONVENTIONS

Demographic formulas

Age of onset of PN is calculated as:

Year of PN diagnosis – Year of birth

BMI is calculated as:

Weight in kg / (height^2 in meters)

Renal function formulas

For adults, creatinine clearance (CLcr) value will be derived using the equation of Cockroft and Gault:

$$\text{CLcr (ml/min)} = (140 - \text{age}) \times \text{weight (kg)} \times (1 - 0.15 \times \text{sex (0-M, 1-F)}) / (0.814 \times \text{creatinine } (\mu\text{mol/l}))$$

CLcr will be calculated using the last weight measurement on or before the visit of the creatinine measurement and age at the lab sampling day. Here age is calculated as following:

Age = age collected at screening + integer part of (lab sampling analysis day/365.25)

Analysis windows for time points

Efficacy assessment

For the efficacy assessment, the reference date for the derivation of relative days of events or findings will be the randomization day. If a participant receives IMP prior to the randomization by mistake, the reference date of efficacy assessment will be the date of the first IMP administration for that participant.

For daily eDiary data (WI-NRS, skin Pain-NRS, Sleep-NRS), all available values of daily measurements will be assigned to each week window according to Table 10, and then weekly average score will be calculated. Randomization day is used as the reference day (Day 1).

Table 10 - Time window for eDiary efficacy variables

Time	Target day	Day range for calculating weekly score
Baseline (Week 0)	1	-7- <1
Week 1	8	1-11
Week 2	15	12-18
Week 3	22	19-25
Week 4	29	26-32
Week 5	36	33-39
Week 6	43	40-46
Week 7	50	47-53
Week 8	57	54-60
Week 9	64	61-67
Week 10	71	68-74
Week 11	78	75-81

Time	Target day	Day range for calculating weekly score
Week 12	85	82-88
Week 13	92	89-95
Week 14	99	96-102
Week 15	106	103-109
Week 16	113	110-116
Week 17	120	117-123
Week 18	127	124-130
Week 19	134	131-137
Week 20	141	138-144
Week 21	148	145-151
Week 22	155	152-158
Week 23	162	159-165
Week 24	169	166-172
Week 25	176	173-179
Week 26	183	180-186
Week 27	190	187-193
Week 28	197	194-200
Week 29	204	201-207
Week 30	211	208-214
Week 31	218	215-221
Week 32	225	222-228
Week 33	232	229-235
Week 34	239	236-242
Week 35	246	243-249
Week 36	253	250-256

For other efficacy variables, all available values of scheduled measurements will be assigned to the appropriate visit window according to Table 11. In the event of multiple measurements of the same test in the same window, the one closest to the targeted visit date will be used for the by-visit summary. If they are at the same number of days away from the target day, the latest one will be used.

Table 11 - Time window for efficacy variables

		Time windows for				
**Visit**	**Target Day**	**DLQI, IGA PN-A/PN-S**	**HADS, EQ-5D-5L, Missed school/work days**	**PGIS**	**PGIC**	**PAS**
Visit 1 (Week -4 to -2)	<1			<-14		<-14
Visit 2 (Week 0)	1	1^-	1^-	-14-1^-		-14-1^-
Visit 3 (Week 4)	29	1^+-42		1^+-42	1^+-42	1^+-42
Visit 4 (Week 8)	57	43-70		43-70	43-70	43-70
Visit 5 (Week 12)	85	71-126	1^+-126	71-126	71-126	71-126
Visit 6 (Week 24)	169	127-210	127-210	>126	>126	127-210
Visit 7 (Week 36)	253	>210	>210			>210

1-: up to randomization and before 1st dose date/time; 1+: after randomization or 1st dose date/time

Safety assessment

For the safety assessment, the reference date for the derivation of relative days of events or findings will be the date of first IMP administration. Selected safety variables will be summarized by the analysis window define in Table 12 for the by visit descriptive analysis. All available values from central lab will be assigned to the appropriate visit window. In the event of multiple measurements of the same test in the same window, the one closest to the targeted visit date will be used for the by-visit summary. If they are at the same number of days away from the target day, the latest one will be used. For procedures planned on Visit 2, if it is done on the same date as the first IMP injection but the performance time is missing, it will belong to Visit 2 time window.

Table 12 - Time window for safety endpoints

		Time windows for							
Visit	Target Day	Vital signs	Hematology, biochemistry	Urinalysis	CD4 T cell count and HIV test	Serum Pregnancy test	Urine Pregnancy test	Physical examination	ECG
Visit 1 (Week -4 to -2)	<1	<-14	<-14	<-14	1^-	<-14		<-14	1^-
Visit 2 (Week 0)	1	-14-1^-	-14-1^-	-14-1^-			-14-1^-	-14-1^-	
Visit 3 (Week 4)	29	1^+-42	1^+-42	1^+-42			1^+-42		
Visit 4 (Week 8)	57	43-70	43-70	43-70			43-70		
Visit 5 (Week 12)	85	71-126	71-126	71-126			71-126		
Visit 6 (Week 24)	169	127-210	127-210	>126	1^+		127-210	1^+-210	1^+
Visit 7 (Week 36)	253	>210	>210				>210	>210	

1-: up to 1st dose date/time; 1+: after 1st dose date/time;

Pharmacokinetics/pharmacodynamics assessment

For the pharmacokinetics/pharmacodynamics variables summary, the reference date for the derivation of relative days of measurements will be the date of first IMP administration if the participant is treated with study treatment, or the randomization date if the participant is not treated. Pharmacokinetics /pharmacodynamics variables will be summarized by the analysis window defined in Table 13 for the by visit descriptive analyses. All available values of measurements will be assigned to the appropriate visit window. In the event of multiple measurements of the same test in the same window, the one closest to the targeted visit date will be used for the by-visit summary. If they are at the same distance to the target day, the latest one will be used. For procedures planned on Visit 2, if it is done on the same date as the first IMP injection but the performance time is missing, it will belong to Visit 2 time window.

Table 13 - Time window for pharmacokinetics/pharmacodynamics variables

Visit	Target day	Time windows for		
		**Serum dupilumab concentration**	**Anti-drug antibodies**	**Total IgE**
Visit 1 (Week -4)	<1			
Visit 2 (Week 0)	1	1^{-}	1^{-}	1^{-}
Visit 3 (Week 4)	29	1^{+}-42		1^{+}-42
Visit 4 (Week 8)	57	43-70		43-70
Visit 5 (Week 12)	85	71-126	1^{+}-126	71-126
Visit 6 (Week 24)	169	127-210	127-210	127-210
Visit 7(Week 36)	253	>210	>210	>210

1-: up to 1st dose date/time or randomization if participant is not treated; 1+: after 1st dose date/time or randomization date if participant is not treated;

Unscheduled visits

Unscheduled visit measurements of laboratory data, vital signs, and ECG will be used for computation of baseline, the last on-treatment value, analysis according to PCSAs, and the shift summaries for safety. They will also be included in the by-visit summaries if they are re-allocated to scheduled visits.

5.5 APPENDIX 5 SAMPLE SAS CODE

The multiple imputation and analysis model for the primary analysis of change from baseline in WI-NRS at Week 12 will be built with the following sample SAS code.

1. 40 datasets with a monotone missing pattern will be obtained, induced by Markov Chain Monte Carlo (MCMC) method on participants who have not taken the prohibited medications and/or rescue medications or have discontinued study treatment due to lack of efficacy prior to Week 12.

```
proc mi data=dat_etd seed=16460 nimpute=40 out=dat_mc;
    mcmc impute=monotone;
    var atopicyn stableyn region antidblyn trt01p winrsbl chg1winrs …
    chg24winrs;
run;
```

2. For each of the imputed dataset with monotone missing pattern in step 1, the remaining missing data will be imputed using the regression method for the monotone pattern with adjustment for covariates including intervention groups, documented history of atopy (atopic or non-atopic), stable use of TCS/TCI (yes or no), region, baseline anti-depressant use (yes or no), and baseline value of the response variable.

```
proc mi data=dat_mc nimpute=1 seed=16461 out=dat_mi;
    by _imputation_;
    class atopicyn stableyn region antidblyn trt01p;
    monotone method=reg;
    var atopicyn stableyn region antidblyn trt01p winrsbl chg1winrs …
    chg24winrs;
run;
```

3. Each of the 40 imputed datasets will be merged with the one dataset imputed by WOCF approach, and then be analyzed using the main statistical model. These 40 imputed datasets will be saved.

```
%macro w1;
      %do i=1%to 40;
      data wocf&i.;
      set wocf;
      _imputation_=&i.;
      run;
      %end;
      data wocf_all;
      set %do j=1 %to 40; wocf&j. %end;;
      run;
%mend w1;
```

```
%w1

data dat_imp;
     set dat_mi  wocf_all;
Run;

proc sort data=dat_imp;
     by _imputation_;
run;

proc glm data= dat_imp;
     by _imputation_;
     class atopicyn stableyn region antidblyn trt01p;
     model  chg24winrs = atopicyn stableyn region antidblyn
              trt01p winrsbl;
     lsmeans trt01p / stderr;
     estimate 'Diff Dupilumab vs Placebo' trt01p -1 1;
     ods output LSMeans=implsmeans Estimates=implsmeandiff;
run;
```

4. Applying Rubin’s rule to combine analysis results (point estimates and standard errors) from 40 imputations using PROC MIANALYZE for the LS means and difference in LS means between dupilumab and placebo. Sample code:

```
proc sort data=implsMeans; by trt01pn _imputation_;run;

proc mianalyze data= implsmeans;
     by trt01pn;
     modeleffects lsmean;
     stderr stderr;
     ods output ParameterEstimates=lsmeans;
run;

proc mianalyze data=implsmeandiff;
     modeleffects estimate;
     stderr stderr;
     ods output ParameterEstimates=lsmeandiff;

run;
```

5.6 APPENDIX 6 SELECTION CRITERIA FOR AE/MEDICATION GROUPINGS

Table 14 - List of PTs or Medications for CMQs/CDGs

Grouping	Preferred Term/Medication Code	Preferred Term/Medication
Conjunctivitis	10001257	Adenoviral conjunctivitis
Conjunctivitis	10010725	Conjunctival irritation
Conjunctivitis	10010726	Conjunctival oedema
Conjunctivitis	10010736	Conjunctival ulcer
Conjunctivitis	10010741	Conjunctivitis
Conjunctivitis	10010744	Conjunctivitis allergic
Conjunctivitis	10010745	Conjunctivitis chlamydial
Conjunctivitis	10010749	Conjunctivitis gonococcal neonatal
Conjunctivitis	10010754	Conjunctivitis tuberculous
Conjunctivitis	10010755	Conjunctivitis viral
Conjunctivitis	10018258	Giant papillary conjunctivitis
Conjunctivitis	10021629	Inclusion conjunctivitis
Conjunctivitis	10030861	Ophthalmia neonatorum
Conjunctivitis	10048908	Seasonal allergy
Conjunctivitis	10049458	Herpes simplex virus conjunctivitis neonatal
Conjunctivitis	10051625	Conjunctival hyperaemia
Conjunctivitis	10053991	Inclusion conjunctivitis neonatal
Conjunctivitis	10061784	Conjunctivitis bacterial
Conjunctivitis	10062889	Pingueculitis
Conjunctivitis	10063669	Photoelectric conjunctivitis
Conjunctivitis	10067317	Oculorespiratory syndrome
Conjunctivitis	10067817	Acute haemorrhagic conjunctivitis
Conjunctivitis	10069166	Blebitis
Conjunctivitis	10071570	Ligneous conjunctivitis
Conjunctivitis	10074701	Noninfective conjunctivitis
Conjunctivitis	10075264	Oculoglandular syndrome
Conjunctivitis	10080825	Conjunctivitis fungal
Conjunctivitis	10084034	Conjunctival suffusion
Intravenous immunoglobulin therapy	CAS 8000012671	IMMUNOGLOBULIN HUMAN NORMAL
Intravenous immunoglobulin therapy	CAS 8000050682	IMMUNOGLOBULIN, PORCINE
Intravenous immunoglobulin therapy	CAS 8000056919	IMMUNOGLOBULIN G HUMAN

Grouping	Preferred Term/Medication Code	Preferred Term/Medication
Intravenous immunoglobulin therapy	CAS 8600000563	IMMUNOGLOBULINS NOS
Intravenous immunoglobulin therapy	CAS 8600001670	IMMUNOGLOBULIN HUMAN NORMAL SLRA
Intravenous immunoglobulin therapy	CAS 8600001671	IMMUNOGLOBULIN HUMAN NORMAL IFAS
Intravenous immunoglobulin therapy	RECNO 900708	OTHER IMMUNOGLOBULINS
Intravenous immunoglobulin therapy	RECNO 900722	IMMUNE SERA AND IMMUNOGLOBULINS
Intravenous immunoglobulin therapy	RECNO 900728	IMMUNOGLOBULINS
Intravenous immunoglobulin therapy	RECNO 900914	SPECIFIC IMMUNOGLOBULINS
Intravenous immunoglobulin therapy	RECNO 901112	IMMUNOGLOBULINS, NORMAL HUMAN

Abbreviations: CAS : Chemical Abstract Service Registry Number RECNO : Drug Record Number

5.7 APPENDIX 7 MEDICATION/PROCEDURE ADJUDICATION ALGORITHM

Algorithm for determining whether treatment with following medications or procedures constitutes treatment failure resulting in setting data as non-responder after taking medication or undergoing procedure in the main statistical analysis

1. Not required to adjudicate post-baseline medications (WHODD-coded) or procedures (CMQ-coded) as these will be considered treatment failures if used at any time[1].

   a) Always considered treatment failure

   - ATC2 = CORTICOSTEROIDS FOR SYSTEMIC USE
   - ATC2 = IMMUNOSUPPRESSANTS (systemic, e.g. oral or parenteral route)
   - Preferred Drug Name = Ciclosporin
   - Preferred Drug Name = Methotrexate
   - Preferred Drug Name = Mycophenolate sodium
   - Preferred Drug Name = Mycophenolic acid
   - Preferred Drug Name = Azathioprine
   - Preferred Drug Name = Gabapentin
   - Preferred Drug Name = Pregabalin
   - Preferred Drug Name = Thalidomide
   - Preferred Drug Name = Lenalidomide
   - Preferred Drug Name = Baricitinib
   - Preferred Drug Name = Ruxolitinib
   - Preferred Drug Name = Tofacitinib
   - Preferred Drug Name = AbrocitinibPreferred Drug Name = Delgocitinib
   - Preferred Drug Name = Nalbuphine
   - Preferred Drug Name = Naltrexone
   - Preferred Drug Name = Naloxone
   - Preferred Drug Name = Vixarelimab
   - Preferred Drug Name = Nemolizumab
   - Preferred Drug Name = Serlopitant
   - Preferred Drug Name = Aprepitant
   - CMQb HLT Phototherapies
   - CMQ Tanning_single PT

b) Never considered treatment failure:

- ATC2 = EMOLLIENTS AND PROTECTIVES
- ATC2 = GENERAL NUTRIENTS
- ATC2 = VITAMINS
- ATC2 = ANTIVIRALS FOR SYSTEMIC USE
- ATC2 = ANTIFUNGALS FOR DERMATOLOGICAL USE
- ATC2 = ANTISEPTICS AND DISINFECTANTS
- ATC2 = ANTIBIOTICS AND CHEMOTHERAPEUTICS FOR DERMATOLOGICAL USE
- ATC2 = ANTI-ACNE PREPARATIONS, excluding D10AA Corticosteroids, combinations for treatment of acne
- ATC2 = OPHTHALMOLOGICALS
- ATC1 = ANTIINFECTIVES FOR SYSTEMIC USE, excluding D07C Corticosteroids, combinations with antibiotics
- ATC1 = BLOOD AND BLOOD FORMING ORGANS
- ATC1 = ALIMENTARY TRACT AND METABOLISM
- ATC1 = MUSCULO-SKELETAL SYSTEM, excluding M01BA Antiinflammatory/antirheumatic agents in combination with corticosteroids
- ATC2 = COUGH AND COLD PREPARATIONS
- ATC2= PSYCHOLEPTICS, excluding N05BB Diphenylmethane derivatives and N05C HYPNOTICS AND SEDATIVES

1 A blinded review of all post-baseline medications or procedures to adjudicate whether treatment constitutes treatment failure, based on medical judgment, may be performed in addition. A listing of treatments or procedures classified as treatment failure in a manner inconsistent with the classification under #1 will be provided, along with supporting rationale.

2. Require to adjudicate medications or procedures that may constitute treatment failure

- All other medications and procedures listed in the protocol and Table 5 of the SAP (not noted in 1. above) given for indications consistent with PN[2]
- Considerations in determining treatment failure include the criteria already set forth in Table 5 of the SAP in addition to the type of medication or procedure, indication, dose, route of administration, timing, frequency and the potential impact of the use of the prohibited medications/procedures and rescue medications on the primary and key secondary efficacy endpoints.

2 Below is a list of indications consistent with PN based on PT level from concomitant medication/procedure data using MedDRA dictionary

System Organ Class	High Level Term	Preferred Term	Preferred Term Code
Skin and subcutaneous tissue disorders	Dermatitis and eczema	Neurodermatitis	10029263
Infections and infestations	Bacterial infections NEC	Eczema impetiginous	10051890
Infections and infestations	Skin structures and soft tissue infections	Dermatitis infected	10012470
Infections and infestations	Skin structures and soft tissue infections	Eczema infected	10014199
Skin and subcutaneous tissue disorders	Dermatitis and eczema	Dermatitis	10012431
Skin and subcutaneous tissue disorders	Dermatitis and eczema	Dermatitis atopic	10012438
Skin and subcutaneous tissue disorders	Dermatitis and eczema	Eczema	10014184

6 REFERENCES

1. Carpenter JR, Roger JH, Kenward MG. Analysis of longitudinal trials with protocol deviation: a framework for relevant, accessible assumptions, and inference via multiple imputation. J Biopharm Stat. 2013;23(6):1352-71.

Signature Page for VV-CLIN-0602730 v2.0
efc16460-16-1-9-sap

Approve & eSign	[redacted]
Approve & eSign	[redacted]

Supplementary Data 2

Institutional Review Board (IRB)/Independent Ethics Committee (IEC) list

Type of Center (1 or 2)	Study Center No.	IRB/IEC Name and Address	
		Local IRB/IEC	National IRB/IEC
1	0320001	Comite de Etica en Farmacologia Clinica<br>Paraguay 2041 9no H<br>C1121ABE Caba<br>Buenos Aires<br>Argentina	
1	0320002	Comite de Etica en Investigacion INAER<br>Arenales 3146 1A<br>C1425BEN Caba<br>Buenos Aires<br>Argentina	
1	0320003	Comite de Etica en Investigacion INAER<br>Arenales 3146 1A<br>C1425BEN Caba<br>Buenos Aires<br>Argentina	
1	0320004	Comite de Etica en Investigacion INAER<br>Arenales 3146 1A<br>C1425BEN Caba<br>Buenos Aires<br>Argentina	

Type of Center (1 or 2)	Study Center No.	IRB/IEC Name and Address	
		Local IRB/IEC	National IRB/IEC
1	0320005	Comite de Etica en Investigacion INAER<br>Arenales 3146 1A<br>C1425BEN Caba<br>Buenos Aires<br>Argentina	
1	0320006	CEI en investigación clínica Dr. Carlos Barclay<br>Larrea 1381 3° A<br>C1117ABK Caba<br>Buenos Aires<br>Argentina	
1	0320007	Comite de Etica en Investigacion INAER<br>Arenales 3146 1A<br>C1425BEN Caba<br>Buenos Aires<br>Argentina	
1	0320008	CEI en investigación clínica Dr. Carlos Barclay<br>Larrea 1381 3° A<br>C1117ABK Caba<br>Buenos Aires<br>Argentina	
1	0320009	CEI en investigación clínica Dr. Carlos Barclay<br>Larrea 1381 3° A<br>C1117ABK Caba<br>Buenos Aires<br>Argentina	

Type of Center (1 or 2)	Study Center No.	IRB/IEC Name and Address	
		Local IRB/IEC	National IRB/IEC
1	1560001	West China Hospital of Sichuan University<br>37 Guoxue lane,<br>610041, Chengdu,<br>Sichuan<br>Mainland China	
1	1560002	Ethics Committee of First People's Hospital of Hangzhou<br>No.261, Huansha Road, Shangcheng District,<br>310000, Hangzhou,<br>Mainland China	
1	1560003	Ethics Committee of Second People's Hospital of Wuxi<br>No. 68 Zhongshan Road,<br>214000, Wuxi<br>Mainland China	
1	1560004	Bioethics Committee, Beijing Friendship Hospital, Capital Medical University<br>No.95, Yong’an Road,<br>Xicheng Distrct,<br>100000, Beijing<br>Mainland China	
1	2500005		CPP Sud-Ouest et Outre-Mer 2<br>Agence Régionale de Santé Occitanie<br>10, chemin du raisin<br>31050 Toulouse Cedex 9<br>France

Type of Center (1 or 2)	Study Center No.	IRB/IEC Name and Address	
		Local IRB/IEC	National IRB/IEC
1	3920001	Institutional Review Board of NTT Medical Center Tokyo<br>5-9-22, Higashi-Gotanda<br>141-8625 Shinagawa-Ku<br>Tokyo<br>Japan	
1	3920002	(Current)<br>The Central Institutional Review Board for the Fujita Health University Hospitals<br>1-98 Dengakugakubo, Kutsukake-cho<br>Toyoake-shi,<br>Aichi 470-1192<br>Japan<br>(Former)<br>Fujita Health University Bantane Hospital IRB<br>3-6-10 Otobashi, Nakagawa-ku<br>Nagoya-city,<br>Aichi 454-8509<br>Japan	
1	3920003	National Defense Medical College Hospital IRB<br>3-2 Namiki<br>359-8513 Tokorozawa-Shi<br>Saitama<br>Japan	

Type of Center (1 or 2)	Study Center No.	IRB/IEC Name and Address	
		Local IRB/IEC	National IRB/IEC
1	3920004	Tokyo Medical And Dental University, Medical Hospital Institutional Review Board<br>1-5-45 Yushima<br>113-8519 Bunkyo-Ku<br>Tokyo<br>Japan	
1	3920005	Kyoto University Hospital Institutional Review Board<br>54 Shogoin Kawahara-cho, Sakyo-ku<br>606-8507 Kyoto-Shi<br>Kyoto<br>Japan	
1	3920006	Nihon University Hospitals' Joint Institutional Review Board<br>30-1, Oyaguchi,. Kami-cho<br>173-8610 Itabashi-Ku<br>Tokyo<br>Japan	
1	3920007	Nagasaki University Hospital Institutional Review Board<br>1-7-1 Sakamoto<br>852-8501 Nagasaki-Shi<br>Nagasaki<br>Japan	

Type of Center (1 or 2)	Study Center No.	IRB/IEC Name and Address	
		Local IRB/IEC	National IRB/IEC
1	3920009	IRB of Yokohama City University Hospital<br>3-9 Fukuura, Kanazawa-ku<br>236-0004 Yokohama-Shi<br>Kanagawa<br>Japan	
1	3920010	Shimane University Hospital, Institutional Review Board<br>89-1 Enya-cho<br>693-8501 Izumo-Shi<br>Shimane<br>Japan	
1	4100001	Gachon University Gil Medical Center Institutional Review Board(GUGMCIRB)<br>21,Namdong-daero,774 beon-gil, Namdong-gu,<br>21565 Incheon<br>Republic of Korea	
1	4100002	Seoul National University Hospital Institutional Review Board<br>101, Daehak-ro, Jongno-gu,<br>03080 Seoul, ,<br>Republic of Korea	
1	4100003	Severance Hospital Institutional Review Board<br>50-1, Yonsei-ro, Seodaemun-gu,<br>03722 Seoul<br>Republic of Korea	

Type of Center (1 or 2)	Study Center No.	IRB/IEC Name and Address	
		Local IRB/IEC	National IRB/IEC
1	4100004	Hallym University Kangnam Sacred Heart Hospital Institutional Review Board<br>1, Singil-ro, Yeongdeungpo-gu,<br>07441 Seoul<br>Republic of Korea	
1	4100005	The Catholic University Of Korea, Incheon St. Mary's Hospital Institutional Review Board<br>56, Dongsu-ro, Bupyeong-gu,<br>21431 Incheon<br>Republic of Korea	
1	4100007	Seoul National University Bundang Hospital Institutional Review Board<br>Gumi-ro 173 Beon-gil 82, Bundang-gu,<br>Seongnam-si,<br>13620 Gyeonggi-do<br>Republic of Korea	

Type of Center<br>(1 or 2)	Study Center No.	IRB/IEC Name and Address	
		Local IRB/IEC	National IRB/IEC
1	4840001	M CEI Hospital Universitario "DR. Jose Eleuterio Gonzalez"<br>AVE. Francisco I.Madero y AVE. Gonzalitos S/N Mitras Centro<br>64460 Monterrey<br>Nuevo León<br>Mexico<br><br>CI Hospital Universitario "Dr. Jose Eleuterio Gonzalez"<br>Av. Francisco I. Madero y Av. Gonzalitos S/N Col. Mitras Centro<br>64460 Monterrey<br>Nuevo León<br>Mexico	

Type of Center (1 or 2)	Study Center No.	IRB/IEC Name and Address	
		Local IRB/IEC	National IRB/IEC
1	4840002	CEI Instituto Jalisciense de Investigacion Clinica S.AdeC.V.<br>Penitenciaria No. 20 Col. Centro<br>44100 Guadalajara<br>Jalisco<br>Mexico<br><br>CI Instituto Jalisciense de Investigacion Clinica S.AdeC.V.<br>Penitenciaria No. 20, Col. Centro,<br>44100Guadalajara<br>Jalisco<br>Mexico	
1	4840003	M CEI Medica Rio Mayo S.C.<br>Av. Rio Mayo No. 1306, Col. Vista Hermosa<br>62290 Cuernavaca<br>Morelos<br>Mexico<br><br>M CI Medica Rio Mayo S.C.<br>Av. Rio Mayo No. 1306 - 104. Col. Vista Hermosa<br>62290 Cuernavaca<br>Morelos<br>Mexico	

Type of Center (1 or 2)	Study Center No.	IRB/IEC Name and Address	
		Local IRB/IEC	National IRB/IEC
1	4840005	CI Hospital La Mision, S.A. de C.V.<br>Av. del Hospital No. 112 Primer Piso Col. Sertoma Monterrey Nuevo Leon<br>64718 Monterrey<br>Nuevo León<br>Mexico<br><br>CEI del Hospital La Mision<br>Av. del Hospital No. 112 Int. 1 y 2 piso Col. Sertoma<br>64718 Monterrey<br>Nuevo León<br>Mexico	
1	4840006	CI Unidad Clinica de Bioequivalencia, S. de R.L. de C.V.<br>Av. Alemania No. 1361 Col. Moderna<br>44190 Guadalajara<br>Jalisco<br>Mexico<br><br>CEI Unidad Clinica de Bioequivalencia, S. de R.L. de C.V.<br>Av. Alemania No. 1361 Col. Moderna<br>44190 Guadalajara<br>Mexico	

Type of Center<br>(1 or 2)	Study Center No.	IRB/IEC Name and Address	
		Local IRB/IEC	National IRB/IEC
1	6430001	LEC of Stavropol Regional Clinical Hospital<br>1, Semashko str.,<br>355030 Stavropol<br>Russian Federation	
1	6430002	LEC of St Petersburg State Medical University n.a. Pavlov<br>6-8, Lev Tolstoy Str.<br>197022 St Petersburg<br>Russian Federation	
1	6430005	LEC of State Scientific Center of Dermatovenerology and Cosmetology<br>bld.3, 6 Korolenko str.,<br>107076 Moscow<br>Russian Federation	
1	6430006	Local Ethics Committee of Russian Medical Academy of Postgra<br>2/1, Barrikadnaya str.,<br>125993 Moscow<br>Russian Federation	
1	6430007	LEC of Clinical dermatovenerogic dispensary<br>179, Rashpilevskaya str.,<br>350020 Krasnodar<br>Russian Federation	

Type of Center (1 or 2)	Study Center No.	IRB/IEC Name and Address	
		Local IRB/IEC	National IRB/IEC
1	6430008	LEC of Chelyabinsk Regional Dermatologic Dispensary<br>24, Yablochkina str.,<br>454048 Chelyabinsk<br>Russian Federation	
1	6430009	LEC of the Saratov State Medical University<br>137, Bolshaya Sadovaya Str.,<br>410054 Saratov,<br>Russian Federation	
1	6430010	LEC of the Institute of Immunology<br>24, Kashirskoe shosse,<br>115522 Moscow<br>Russian Federation	
1	8400001	Copernicus Group IRB<br>One Triangle Drive<br>Suite 100<br>27713 Research Triangle Park<br>North Carolina<br>United States	
1	8400002	Copernicus Group IRB<br>One Triangle Drive<br>Suite 100<br>27713 Research Triangle Park<br>North Carolina<br>United States	

Type of Center (1 or 2)	Study Center No.	IRB/IEC Name and Address	
		Local IRB/IEC	National IRB/IEC
1	8400003	Copernicus Group IRB<br>One Triangle Drive<br>Suite 100<br>27713 Research Triangle Park<br>North Carolina<br>United States	
1	8400004	Copernicus Group IRB<br>One Triangle Drive<br>Suite 100<br>27713 Research Triangle Park<br>North Carolina<br>United States	
1	8400005	Copernicus Group IRB<br>One Triangle Drive<br>Suite 100<br>27713 Research Triangle Park<br>North Carolina<br>United States	
1	8400006	Copernicus Group IRB<br>One Triangle Drive<br>Suite 100<br>27713 Research Triangle Park<br>North Carolina<br>United States	

Type of Center (1 or 2)	Study Center No.	IRB/IEC Name and Address	
		Local IRB/IEC	National IRB/IEC
1	8400007	Copernicus Group IRB<br>One Triangle Drive<br>Suite 100<br>27713 Research Triangle Park<br>North Carolina<br>United States	
1	8400009	Copernicus Group IRB<br>One Triangle Drive<br>Suite 100<br>27713 Research Triangle Park<br>North Carolina<br>United States	
1	8400010	Copernicus Group IRB<br>One Triangle Drive<br>Suite 100<br>27713 Research Triangle Park<br>North Carolina<br>United States	
1	8400011	Copernicus Group IRB<br>One Triangle Drive<br>Suite 100<br>27713 Research Triangle Park<br>North Carolina<br>United States	

Type of Center (1 or 2)	Study Center No.	IRB/IEC Name and Address	
		Local IRB/IEC	National IRB/IEC
1	8400013	Western Institutional Review Board<br>1019 39th Avenue SE<br>98324 Puyallup<br>Washington<br>United States	
1	8400014	University of Miami<br>1500 NW 12th Avenue<br>Suite 1002<br>33136 Miami<br>Florida<br>United States	
1	8400015	Copernicus Group IRB<br>One Triangle Drive<br>Suite 100<br>27713 Research Triangle Park<br>North Carolina<br>United States	
1	8400016	Copernicus Group IRB<br>One Triangle Drive<br>Suite 100<br>27713 Research Triangle Park<br>North Carolina<br>United States	

Type of Center (1 or 2)	Study Center No.	IRB/IEC Name and Address	
		Local IRB/IEC	National IRB/IEC
1	8400017	Copernicus Group IRB<br>5000 CentreGreen Way<br>Suite 200<br>27513 Cary<br>North Carolina<br>United States	
1	8400018	Copernicus Group IRB<br>One Triangle Drive<br>Suite 100<br>27713 Research Triangle Park<br>North Carolina<br>United States	
1	8400019	Copernicus Group IRB<br>5000 CentreGreen Way<br>Suite 200<br>27513 Cary<br>North Carolina<br>United States	
1	8400022	Copernicus Group IRB<br>One Triangle Drive<br>Suite 100<br>27713 Research Triangle Park<br>North Carolina<br>United States	

Type of Center (1 or 2)	Study Center No.	IRB/IEC Name and Address	
		Local IRB/IEC	National IRB/IEC
1	8400024	Copernicus Group IRB<br>5000 CentreGreen Way<br>Suite 200<br>27513 Cary<br>North Carolina<br>United States	
1	8400025	Copernicus Group IRB<br>5000 CentreGreen Way<br>Suite 200<br>27513 Cary<br>North Carolina<br>United States	
1	8400026	Copernicus Group IRB<br>5000 CentreGreen Way<br>Suite 200<br>27513 Cary<br>North Carolina<br>United States	
2	1240007	Advarra<br>372 Hollandview Trail, Suite 300<br>L4G 0A5 Aurora<br>Ontario<br>Canada	

Type of Center (1 or 2)	Study Center No.	IRB/IEC Name and Address	
		Local IRB/IEC	National IRB/IEC
2	2500001		CPP Sud-Ouest et Outre-Mer 2<br>Agence Régionale de Santé Occitanie<br>10, chemin du raisin<br>31050 Toulouse Cedex 9<br>France
2	4100006	Asan Medical Center Institutional Review Board<br>88, Olympic-ro 43-gil, Songpa-gu,<br>05505 Seoul,<br>Republic of Korea	

Institutional Review Board (IRB)/Independent Ethics Committee (IEC) list

Type of Center (1 or 2)	Study Center No.	IRB/IEC Name and Address	
		Local IRB/IEC	National IRB/IEC
1	1240001	Advarra<br>372 Hollandview Trail, Suite 300<br>L4G 0A5 Aurora<br>Ontario<br>Canada	
1	1240002	Health Research Ethics Board of Alberta<br>Clinical Trials Committee<br>1500, 10104 - 103 Avenue NW<br>Edmonton, Alberta, T5J 0H8<br>Canada	
1	1240006	Advarra<br>372 Hollandview Trail, Suite 300<br>L4G 0A5 Aurora<br>Ontario<br>Canada	
1	1240007	Advarra<br>372 Hollandview Trail, Suite 300<br>L4G 0A5 Aurora<br>Ontario<br>Canada	

Type of Center<br>(1 or 2)	Study Center No.	IRB/IEC Name and Address	
		Local IRB/IEC	National IRB/IEC
1	1240008	Advarra<br>372 Hollandview Trail, Suite 300<br>L4G 0A5 Aurora<br>Ontario<br>Canada	
1	1240009	Veritas IRB Inc (current)<br>3551 St. Charles Blvd., Suite 501<br>Kirkland, Quebec<br>H9H 3C4<br><br>Advarra (former)<br>372 Hollandview Trail, Suite 300<br>L4G 0A5 Aurora<br>Ontario<br>Canada	
1	1520001	Comite de Etica de la Investigacion S.S.M Central<br>Victoria subercaseaux 381, 5° piso, Santiago Centro<br>8360160 Santiago<br>Reg Metropolitana de Santiago<br>Chile	

Type of Center (1 or 2)	Study Center No.	IRB/IEC Name and Address	
		Local IRB/IEC	National IRB/IEC
1	1520002	Comite de Etica de la Investigacion S.S.M. Norte<br>San Jose # 1053<br>Independencia<br>8380755 Santiago<br>Reg Metropolitana de Santiago<br>Chile	
1	1520003	Comite de Etica de la Investigacion S.S.M. Norte<br>San Jose # 1053<br>Independencia<br>8380755 Santiago<br>Reg Metropolitana de Santiago<br>Chile	
1	1520004	Comite de Etica de la Investigacion S.S.M Central<br>Santa Rosa 1234<br>8360160 Santiago<br>Reg Metropolitana de Santiago<br>Chile	
1	1520005	Comite Etico de Investigacion Servicio de Salud Valdivia<br>Vicente Perez Rosales #560 of 307<br>Edificio Prales<br>5110537 Valdivia<br>Chile	

Type of Center (1 or 2)	Study Center No.	IRB/IEC Name and Address	
		Local IRB/IEC	National IRB/IEC
1	1520006	Comite Etico de Investigacion Servicio de Salud Valdivia<br>Vicente Perez Rosales #560 of 307<br>Edificio Prales<br>5110537 Valdivia<br>Chile	
1	1580001	Research Ethics Committee, National Taiwan University Hospital<br>No.7, Chung Shan S. Rd,<br>Zhongzheng Dist.<br>10002 Taipei<br>Taiwan	
1	1580002	Institutional Review Board, Mackay Memorial Hospital<br>No. 92, Sec. 2, Zhongshan N. Rd.,<br>10449 Taipei<br>Taiwan	
1	1580005	Institional Reivew Board of National Taiwan University Hospital Hsin-Chu Branch,<br>No.25, Lane 442, Jingguo Road,<br>30059 Hsinchu City<br>Taiwan	

Type of Center<br>(1 or 2)	Study Center No.	IRB/IEC Name and Address	
		Local IRB/IEC	National IRB/IEC
1	1580006	Chang Gung Memorial Foundation Institutional Review Board, No. 199, Tung Hwa<br>North Road,<br>10507 Taipei<br>Taiwan	
1	1580008	The Institional Reivew Board , Chung Shan Medical University Hospital<br>No. 110, Sec. 1, Chien Kuo N. Rd.,<br>South Dist.,<br>40201 Taichung<br>Taiwan	
1	2500001		CPP Ouest 3<br>CHU de Poitiers<br>Pavillon Le Blaye - Porte 9<br>2, rue de la Milètrie<br>86021 Poitiers Cedex<br>France
1	2500002		CPP Ouest 3<br>CHU de Poitiers<br>Pavillon Le Blaye - Porte 9<br>2, rue de la Milètrie<br>86021 Poitiers Cedex<br>France

Type of Center (1 or 2)	Study Center No.	IRB/IEC Name and Address	
		Local IRB/IEC	National IRB/IEC
1	2500003		CPP Ouest 3<br>CHU de Poitiers<br>Pavillon Le Blaye - Porte 9<br>2, rue de la Milètrie<br>86021 Poitiers Cedex<br>France
1	2500004		CPP Ouest 3<br>CHU de Poitiers<br>Pavillon Le Blaye - Porte 9<br>2, rue de la Milètrie<br>86021 Poitiers Cedex<br>France
1	2500005		CPP Ouest 3<br>CHU de Poitiers<br>Pavillon Le Blaye - Porte 9<br>2, rue de la Milètrie<br>86021 Poitiers Cedex<br>France
1	2500006		CPP Ouest 3<br>CHU de Poitiers<br>Pavillon Le Blaye - Porte 9<br>2, rue de la Milètrie<br>86021 Poitiers Cedex<br>France

Type of Center (1 or 2)	Study Center No.	IRB/IEC Name and Address	
		Local IRB/IEC	National IRB/IEC
1	2500007		CPP Ouest 3<br>CHU de Poitiers<br>Pavillon Le Blaye - Porte 9<br>2, rue de la Milètrie<br>86021 Poitiers Cedex<br>France
1	2500008		CPP Ouest 3<br>CHU de Poitiers<br>Pavillon Le Blaye - Porte 9<br>2, rue de la Milètrie<br>86021 Poitiers Cedex<br>France
1	3480002	Orosházi Kórház Intézményi Etikai Bizottság<br>Könd utca 59.<br>5900 Orosháza<br>Hungary	Egészségügyi Tudományos Tanács<br>Klinikai Farmakológiai Etikai Bizottság<br>Alkotmány utca 25.<br>1054 Budapest<br>Hungary
1	3480003	Szegedi Tudományegyetem Szent-Györgyi Albert Orvos- és Gyógyszerésztudományi Centrum Regionális Tudományos és Kutatásetikai Bizottsága<br>Korányi fasor 8-10.<br>6720 Szeged<br>Hungary	Egészségügyi Tudományos Tanács<br>Klinikai Farmakológiai Etikai Bizottság<br>Alkotmány utca 25.<br>1054 Budapest<br>Hungary

Type of Center<br>(1 or 2)	Study Center No.	IRB/IEC Name and Address	
		Local IRB/IEC	National IRB/IEC
1	3480004	Debreceni Egyetem Klinikai Központ<br>Regionális Tudományos és Kutatásetikai Bizottsága<br>Nagyerdei krt. 98.<br>4032 Debrecen<br>Hungary	Egészségügyi Tudományos Tanács<br>Klinikai Farmakológiai Etikai Bizottság<br>Alkotmány utca 25.<br>1054 Budapest<br>Hungary
1	3480005	Pécsi Tudományegyetem Klinikai Központ<br>Regionális Kutatásetikai Bizottság<br>Rákóczi u. 2.<br>7623 Pécs<br>Hungary	Egészségügyi Tudományos Tanács<br>Klinikai Farmakológiai Etikai Bizottság<br>Alkotmány utca 25.<br>1054 Budapest<br>Hungary
1	3800001	Comitato Etico Indipendente Istituto Clinico Humanitas<br>Via Alessandro Manzoni, 56<br>20089 Rozzano (MI)<br>Italy	
1	3800002	Comitato Etico Milano Area 2<br>Fondazione IRCCS Ca' Granda Ospedale Maggiore Policlinico<br>Via Francesco Sforza 28<br>20122 Milano<br>Italy	

Type of Center (1 or 2)	Study Center No.	IRB/IEC Name and Address	
		Local IRB/IEC	National IRB/IEC
1	3800003	Comitato Etico Regione Calabria Sezione Area Centro<br>Via Tommaso Campanella N. 115<br>88100, Catanzaro (CZ)<br>Italy	
1	3800004	Comitato Etico Regionale (C.E.R.) delle Marche<br>AOU Ospedali Riuniti Umberto I, G.M. Lancisi, G. Salesi di Ancona<br>Via Conca, 71<br>60126 Torrette di Ancona<br>Italy	
1	4100001	Institutional Review Board(IRB),Chung-Ang University Hospital<br>102, Heukseok-ro, Dongjak-Gu, Seoul 06973,<br>Republic of Korea	
1	4100002	Pusan National University Hospital Institutional Review Board, 179, Gudeok-ro, Seo-gu, Busan-si, 49241,<br>Republic of Korea	
1	4100003	Soonchunhyang University Hospital Institutional Review Board, 170, Jomaru-ro, Bucheon-si, Gyeonggi-do, 14584,<br>Republic of Korea	

Type of Center (1 or 2)	Study Center No.	IRB/IEC Name and Address	
		Local IRB/IEC	National IRB/IEC
1	4100005	Severance Hospital Institutional Review Board<br>50-1, Yonsei-ro, Seodaemun-gu,<br>03722 Seoul,<br>Republic of Korea	
1	4100006	Hallym University Kangnam Sacred Heart Hospital Institutional Review Board<br>1, Singil-ro, Yeongdeungpo-gu,<br>07441 Seoul<br>Republic of Korea	
1	4100007	The Catholic University Of Korea, Incheon St. Mary's Hospital Institutional Review Board<br>56, Dongsu-ro, Bupyeong-gu,<br>21431 Incheon<br>Republic of Korea	
1	6200001		Comissão de Ética para a Investigação Clínica<br>Parque da Saúde de Lisboa, Pav. 17-A,<br>Avenida do Brasil nº 53,<br>1749-004 Lisboa<br>Portugal
1	6200002		Comissão de Ética para a Investigação Clínica<br>Parque da Saúde de Lisboa, Pav. 17-A,<br>Avenida do Brasil nº 53,<br>1749-004 Lisboa<br>Portugal

Type of Center (1 or 2)	Study Center No.	IRB/IEC Name and Address	
		Local IRB/IEC	National IRB/IEC
1	6200003		Comissão de Ética para a Investigação Clínica<br>Parque da Saúde de Lisboa, Pav. 17-A,<br>Avenida do Brasil nº 53,<br>1749-004 Lisboa<br>Portugal
1	7240001		CEIm Provincial de Sevilla<br>Avda. Dr. Fedriani, 3 – Unidad de Investigación 2ª planta,<br>41009 Sevilla<br>Spain
1	7240002		CEIm Provincial de Sevilla<br>Avda. Dr. Fedriani, 3 – Unidad de Investigación 2ª planta,<br>41009 Sevilla<br>Spain
1	7240003		CEIm Provincial de Sevilla<br>Avda. Dr. Fedriani, 3 – Unidad de Investigación 2ª planta,<br>41009 Sevilla<br>Spain
1	7240004		CEIm Provincial de Sevilla<br>Avda. Dr. Fedriani, 3 – Unidad de Investigación 2ª planta,<br>41009 Sevilla<br>Spain

Type of Center (1 or 2)	Study Center No.	IRB/IEC Name and Address	
		Local IRB/IEC	National IRB/IEC
1	7240007		CEIm Provincial de Sevilla<br>Avda. Dr. Fedriani, 3 – Unidad de Investigación 2ª planta,<br>41009 Sevilla<br>Spain
1	7240008		CEIm Provincial de Sevilla<br>Avda. Dr. Fedriani, 3 – Unidad de Investigación 2ª planta,<br>41009 Sevilla<br>Spain
1	7240009		CEIm Provincial de Sevilla<br>Avda. Dr. Fedriani, 3 – Unidad de Investigación 2ª planta,<br>41009 Sevilla<br>Spain
1	8260001		North East - Newcastle & North Tyneside 2 Research Ethics Committee<br>NHS BT Blood Donor Centre<br>Holland Drive<br>Newcastle upon Tyne<br>NE2 4NQ Tyne and Wear<br>United Kingdom

Type of Center (1 or 2)	Study Center No.	IRB/IEC Name and Address	
		Local IRB/IEC	National IRB/IEC
1	8400001	Copernicus Group IRB<br>One Triangle Drive<br>Suite 100<br>27713 Research Triangle Park<br>North Carolina<br>United States	
1	8400002	Copernicus Group IRB<br>One Triangle Drive<br>Suite 100<br>27713 Research Triangle Park<br>North Carolina<br>United States	
1	8400003	Johns Hopkins IRB<br>Reed Hall B-130<br>1620 McElderry Street<br>21205 Baltimore<br>Maryland<br>United States	
1	8400005	Copernicus Group IRB<br>5000 CentreGreen Way<br>Suite 200<br>27513 Cary<br>North Carolina<br>United States	

Type of Center (1 or 2)	Study Center No.	IRB/IEC Name and Address	
		Local IRB/IEC	National IRB/IEC
1	8400006	Copernicus Group IRB<br>One Triangle Drive<br>Suite 100<br>27713 Research Triangle Park<br>North Carolina<br>United States	
1	8400008	Copernicus Group Independent Review Board<br>5000 CentreGreen Way<br>Suite 200<br>27513 Cary<br>United States	
1	8400054	Copernicus Group IRB<br>One Triangle Drive<br>Suite 100<br>27713 Research Triangle Park<br>North Carolina<br>United States	
2	4100004	Gachon University Gil Medical Center<br>Institutional Review Board(GUGMCIRB)<br>21, Namdong-daero,774 beon-gil,<br>Namdong-gu,<br>21565 Incheon,<br>Republic of Korea	

Type of Center (1 or 2)	Study Center No.	IRB/IEC Name and Address	
		Local IRB/IEC	National IRB/IEC
2	7240005		CEIm Provincial de Sevilla<br>Avda. Dr. Fedriani, 3 – Unidad de Investigación 2ª planta,<br>41009 Sevilla<br>Spain
2	8400007	Copernicus Group IRB<br>One Triangle Drive<br>Suite 100<br>27713 Research Triangle Park<br>North Carolina<br>United States	
