# Supplementary material for: Dupilumab in patients with prurigo nodularis: two randomized, double-blind, placebo-controlled phase 3 trials
Source: Nat Med. 2023 May 4;29(5):1180–90. doi: 10.1038/s41591-023-02320-9 (PMC10202800; doi:10.1038/s41591-023-02320-9)
Supplement: Supplementary file 2 — Reporting Summary [file 41591_2023_2320_MOESM2_ESM.pdf]

nature portfolio

Corresponding author(s): John T. O'Malley

Last updated by author(s): Mar 22, 2023

Reporting Summary

Nature Portfolio wishes to improve the reproducibility of the work that we publish. This form provides structure for consistency and transparency in reporting. For further information on Nature Portfolio policies, see our Editorial Policies and the Editorial Policy Checklist.

Statistics

For all statistical analyses, confirm that the following items are present in the figure legend, table legend, main text, or Methods section.

n/a	Confirmed	
☐	☒	The exact sample size (*n*) for each experimental group/condition, given as a discrete number and unit of measurement
☒	☐	A statement on whether measurements were taken from distinct samples or whether the same sample was measured repeatedly
☐	☒	The statistical test(s) used AND whether they are one- or two-sided<br>*Only common tests should be described solely by name; describe more complex techniques in the Methods section.*
☐	☒	A description of all covariates tested
☐	☒	A description of any assumptions or corrections, such as tests of normality and adjustment for multiple comparisons
☐	☒	A full description of the statistical parameters including central tendency (e.g. means) or other basic estimates (e.g. regression coefficient) AND variation (e.g. standard deviation) or associated estimates of uncertainty (e.g. confidence intervals)
☐	☒	For null hypothesis testing, the test statistic (e.g. *F*, *t*, *r*) with confidence intervals, effect sizes, degrees of freedom and *P* value noted<br>*Give P values as exact values whenever suitable.*
☒	☐	For Bayesian analysis, information on the choice of priors and Markov chain Monte Carlo settings
☐	☒	For hierarchical and complex designs, identification of the appropriate level for tests and full reporting of outcomes
☒	☐	Estimates of effect sizes (e.g. Cohen's *d*, Pearson's *r*), indicating how they were calculated

Our web collection on statistics for biologists contains articles on many of the points above.

Software and code

Policy information about availability of computer code

Data collection	MEDIDATA-RAVE v2020.3 was used for data collection, and ERT syndication application was used to capture patient digital diary data
Data analysis	SAS software version 9.4 (Cary, NC, USA)

For manuscripts utilizing custom algorithms or software that are central to the research but not yet described in published literature, software must be made available to editors and reviewers. We strongly encourage code deposition in a community repository (e.g. GitHub). See the Nature Portfolio guidelines for submitting code & software for further information.

Data

Policy information about availability of data

All manuscripts must include a data availability statement. This statement should provide the following information, where applicable:

- Accession codes, unique identifiers, or web links for publicly available datasets
- A description of any restrictions on data availability
- For clinical datasets or third party data, please ensure that the statement adheres to our policy

Scientific and medical researchers may request access to study documents (including the clinical study report, study protocol with any amendments, blank case report form, and statistical analysis plan) that support the methods and findings reported in this manuscript. Individual anonymized participant data will be considered for sharing once the product and indication has been approved by major health authorities (eg, US Food and Drug Administration, European Medicines

Agency, Pharmaceuticals and Medical Devices Agency), if there is legal authority to share the data and there is not a reasonable likelihood of participant re-identification. Requests should be submitted to https://vivli.org/ and will be addressed within 60 days.

Human research participants

Policy information about studies involving human research participants and Sex and Gender in Research.

Reporting on sex and gender	Both male and female patients were included in this study. Demographic information about the number of males and females is provided in Table 1.
Population characteristics	Patient demographic and clinical characteristic data are presented in Table 1.
Recruitment	The sponsor engaged stakeholders as equal partners in the process of protocol writing and ensured the relevance of the scientific question to stakeholders. The sponsor took feedback from sites for designing the protocol. Sponsors selected appropriate sites based on evidence-based trial feasibility analysis, and chose sites with investigators that had required experience, adequate site infrastructure, institutional resources, and target population access. The use of institutional ethics committee-approved recruitment strategies were employed. These recruitment strategies minimized self-selection bias and are unlikely to impact results.
Ethics oversight	The local institutional review board or ethics committee at each study center oversaw trial conduct and documentation. Due to the extent of this information, details on the institutes/organizations provided in supplementary data 2.

Note that full information on the approval of the study protocol must also be provided in the manuscript.

Field-specific reporting

Please select the one below that is the best fit for your research. If you are not sure, read the appropriate sections before making your selection.

☒ Life sciences ☐ Behavioural & social sciences ☐ Ecological, evolutionary & environmental sciences

For a reference copy of the document with all sections, see nature.com/documents/nr-reporting-summary-flat.pdf

Life sciences study design

All studies must disclose on these points even when the disclosure is negative.

Sample size	A sample size of 150 patients for PRIME and PRIME2 each was estimated to provide 90% power to detect a 28% difference in the primary endpoint between dupilumab and placebo with a Fisher exact test at a two-sided alpha of 0.05, assuming response rates of 39% and 11%, respectively.
Data exclusions	There were no data exclusions to report.
Replication	The analyses include an as-observed analysis, a hybrid method analysis, and a tipping point analysis. Sensitivity analyses of the primary and key secondary are presented in Extended Data Table 2. The results were consistent with that from the primary analysis.
Randomization	Randomization was performed centrally using a permuted block randomization schedule via Interactive Voice Response System/Interactive Web Response System and was stratified by documented history of atopy (atopic or nonatopic), stable use of TCS/TCI (yes or no), and country/territory code.
Blinding	Study was double blinded.

Reporting for specific materials, systems and methods

We require information from authors about some types of materials, experimental systems and methods used in many studies. Here, indicate whether each material, system or method listed is relevant to your study. If you are not sure if a list item applies to your research, read the appropriate section before selecting a response.

Materials & experimental systems

n/a	Involved in the study
☒	☐ Antibodies
☒	☐ Eukaryotic cell lines
☒	☐ Palaeontology and archaeology
☒	☐ Animals and other organisms
☐	☒ Clinical data
☒	☐ Dual use research of concern

Methods

n/a	Involved in the study
☒	☐ ChIP-seq
☒	☐ Flow cytometry
☒	☐ MRI-based neuroimaging

Clinical data

Policy information about clinical studies

All manuscripts should comply with the ICMJE guidelines for publication of clinical research and a completed CONSORT checklist must be included with all submissions.

Clinical trial registration

NCT04183335, NCT04202679

Study protocol

Scientific and medical researchers may request access to study documents (including the clinical study report, study protocol with any amendments, blank case report form, and statistical analysis plan) that support the methods and findings reported in this manuscript. Individual anonymized participant data will be considered for sharing once the product and indication has been approved by major health authorities (eg, US Food and Drug Administration, European Medicines Agency, Pharmaceuticals and Medical Devices Agency), if there is legal authority to share the data and there is not a reasonable likelihood of participant re-identification. Requests should be submitted to https://vivli.org/ and will be addressed within 60 days.

Data collection

Patients were enrolled in 16 countries in North and South America, Europe, and Asia, from December 12, 2019, to February 3, 2022 (PRIME) and January 16, 2020, to November 22, 2021 (PRIME2). Due to the extent of this information, all the study centers and investigators list are available on the supplementary appendix.

Outcomes

The primary endpoint was the proportion of patients with a ≥4-point reduction in WI-NRS score (range 0 [“no itch”] to 10 [“worst imaginable itch”]) at week 24 (PRIME) or week 12 (PRIME2). WI-NRS is validated in PN, with research to date supporting a 4-point reduction as clinically meaningful.
Key secondary endpoints were the proportion of patients with a ≥4-point reduction in WI-NRS at week 24 (PRIME2); reduction in skin lesion number to an IGA PN-S (range 0 [“clear, no nodules”] to 4 [“severe, ≥100 nodules”])score of 0 or 1 at week 24 (both trials); and a composite endpoint comprising proportion of patients concomitantly achieving a ≥4-point reduction in WI-NRS with an IGA PN-S of 0 or 1 at week 24 (both trials). IGA PN-S is also validated in PN. Other multiplicity-controlled secondary endpoints, which also included assessment of QoL, skin pain, sleep, and mental health, are listed in Extended Data Table 1.
The full list of prespecified endpoints is provided in page 30.
